# Population Structure of *Staphylococcus aureus* from Trinidad & Tobago

**DOI:** 10.1371/journal.pone.0089120

**Published:** 2014-02-19

**Authors:** Stefan Monecke, Bettina Stieber, Rashida Roberts, Patrick Eberechi Akpaka, Peter Slickers, Ralf Ehricht

**Affiliations:** 1 Institute for Medical Microbiology and Hygiene, Technical University of Dresden, Dresden, Germany; 2 Alere Technologies GmbH, Jena, Germany; 3 Department of Para-Clinical Sciences, The University of the West Indies, St. Augustine, Trinidad and Tobago; Columbia University, United States of America

## Abstract

It has been shown previously that high rates of methicillin- and mupirocin-resistant *Staphylococcus aureus* exist in the Caribbean islands of Trinidad and Tobago, as well as a high prevalence of Panton-Valentine leukocidin-positive *S. aureus*. Beyond these studies, limited typing data have been published. In order to obtain insight into the population structure not only of MRSA but also of methicillin-susceptible *S. aureus*, 294 clinical isolates collected in 2012/2013 were typed by microarray hybridisation. A total of 15.31% of the tested isolates were MRSA and 50.00% were PVL-positive. The most common MSSA strains were PVL-positive CC8-MSSA (20.41% of all isolates tested), PVL-positive CC152-MSSA (9.52%) and PVL-positive CC30-MSSA (8.84%) while the most common MRSA were ST239-MRSA-III&SCC*mer* (9.18%) and ST8-MRSA-IV, “USA300” (5.78%). 2.38% of characterised isolates belonged to distinct strains likely to be related to “*Staphylococcus argenteus*” lineages. The population structure of *S. aureus* isolates suggests an importation of strains from Africa, endemicity of PVL-positive MSSA (mainly CC8) and of ST239-MRSA-III, and a recent emergence of the PVL-positive CC8-MRSA-IV strain “USA300”.

## Introduction

In Trinidad and Tobago, methicillin-resistant *Staphylococcus aureus* (MRSA) poses a public health risk [1], [2], [3], [4]. MRSA prevalence is high and there is no strict isolation of patients with suspected MRSA-infection, as practised in hospitals elsewhere. A limited number of studies have been conducted on *S. aureus* for which the main focus has generally been MRSA. Swanston [5] performed a study in the Port of Spain General Hospital between June 1995 and May 1996 where the prevalence of MRSA was found to be 4.6%. Akpaka *et al.* [2] conducted a study of 1912 *S. aureus* isolates collected from major hospitals in the country between 2000 and 2001 and found that 12.8% of the isolates were methicillin-/oxacillin-resistant. In another study by Orrett [4] isolates were collected from various hospital and communal facilities in Trinidad between January 1, 1999 and December 31, 2004. It was found that the prevalence of MRSA from surgical/burn wounds, urine and pus/abscess were 60.1%, 15.5% and 6.6%, respectively. The major sources of MSSA were surgical/burn wounds, pus/abscess samples and upper respiratory tract specimens with rates of 32.9%, 17.1% and 14.3%, respectively. The vast majority of MRSA isolates from Trinidad and Tobago belong to the hospital-associated strain ST239-MRSA-III. Other strains were rare although the emerging “community-acquired” MRSA (CA-MRSA) strain “USA300” (ST8-MRSA-IV [PVL+/ACME+]) was also found [3]. Of special interest with regard to infection control is a high rate of mupirocin resistance among MRSA isolates [3], [6].

Another issue is the frequent carriage of Panton-Valentine leukocidin genes, lukF-PV and lukS-PV, in S. aureus isolates from Trinidad and Tobago. Following a case of a child who died hours after admission despite being managed in the best intensive care unit of the country [7], further studies [8] revealed a high prevalence of PVL genes in isolates from skin and soft tissue infections, as well as a high proportion of PVL-positive, methicillin-susceptible S. aureus (MSSA) belonging to clonal complex (CC) 8.

In order to obtain an overview not only on MRSA but also on MSSA, clinical isolates from Trinidad and Tobago were collected in 2012/2013 and characterised by microarray hybridisation and, partially, by multi locus sequence typing (MLST).

## Materials and Methods

### Study sites

There are five main regional hospitals in Trinidad and Tobago, namely, Port of Spain General Hospital, Eric Williams Medical Sciences Complex (EWMSC), San Fernando General Hospital (SFGH), Sangre Grande Hospital and Scarborough General Hospital (SGH). Additionally, there are nine district health facilities and 97 health centres throughout the country besides several privately run hospitals. For the purpose of this study, isolates were collected at three major hospitals. Two of them (EWMSC and SFGH) are located on the island of Trinidad, while the third one (SGH) is situated on Tobago.

The population served by EWMSC is about 600,000 and 19,029 admissions were recorded for the year 2011. Approximately 450 *S. aureus* strains were isolated, 20% of which were MRSA. The population served by the SFGH is also about 600,000. For 2011, there were 52,137 admissions. Over 700 *S. aureus* strains were isolated, 35% of which were MRSA.

SGH is Tobago's major health facility, being the only hospital located on the island during the period of the study. Supplemented by 18 health centres it serves a population of 55,000. During the study period, the hospital was still located on Fort Street. SGH was then a 100 bed facility that provided a variety of critical facilities such as Accident and Emergency, Medical and Surgical procedures. It has since been relocated to Signal Hill where a state of the art health care facility has been constructed.

### Isolates and origin

*S. aureus* isolates for this study were recovered from routine clinical samples processed at the hospitals. The isolates were included in the study after being initially identified at the medical microbiological laboratories of the participating hospitals. Prior to shipping the isolates to Dresden, further tests were carried out at the laboratory of the Department of Para-Clinical Sciences to confirm the species of the isolates and determine their antibiotic susceptibilities. Standard laboratory methods (including the automated Microscan walkaway 96 system (Siemens, USA)) were used to identify these bacterial isolates according to Clinical and Laboratory Standards Institute (CLSI) guidelines.

### Microarray procedures

The Alere StaphyType DNA microarray (Alere Technologies GmbH, Jena, Germany) was used for the characterisation of the isolates. The array allows the detection of 333 genes including species markers, typing markers, resistance genes and exotoxins encoding sequences as well as an assignment to clonal complexes and strains. The protocols and procedures were described in detail in previous work [9], [10].

In short, bacteria were grown on Columbia blood agar and incubated overnight at 37°C. The harvested staphylococci were suspended to enzymatic lysis by lysostaphin, lysozyme and RNAse A and digested using Proteinase K. Then, the extracted DNA was purified using an automated system (EZ1, Qiagen, Hilden, Germany).

The purified DNA was used as template for an iterated linear primer elongation using one primer per target. During linear amplification, all targets were labelled by internal incorporation of biotin-16-dUTP. Labelled and amplified single-stranded DNA was hybridised to the array, followed by washing and blocking. Horseradish-peroxidase-streptavidin was added which bound to biotin-labelled DNA. Positive reactions were visualised by the local precipitation of a dye by the peroxidase. An image of the microarray was taken and automatically analysed using dedicated reader and software (ArrayMate and Iconoclust, Alere Technologies).

### MLST

Multilocus sequence typing (MLST) was performed by amplification and sequencing of housekeeping genes *arcC*, *aroE*, *glpF*, *gmk*, *pta*, *tpi*, and *yqiL* using primers and reaction conditions that have been described previously [11]. However, alternative primers were used for the *aroE* gene in order to better fit a formerly published sequence (GenBank FR821777) of “*Staphylococcus argenteus*”-like strains [12]. These primer sequences were aro_fw_agt: GTCCAATTGAGCATTCTTTATCA and aro_rv_agt: CATACCTGCCGGAGTAGT. Resulting sequences were analysed using the software tools on the MLST website (http://saureus.mlst.net/).

### Tree reconstruction

**Figure 1:**
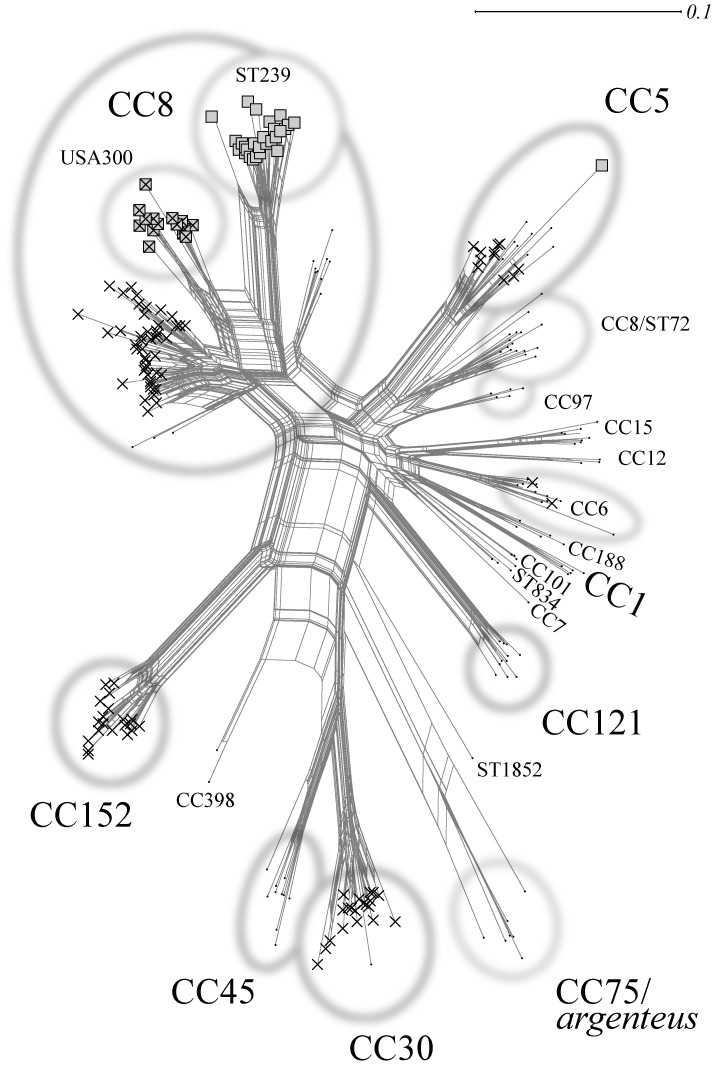
SplitsTree graph visualising strain assignments and similarities between isolates. Isolates were numbered as in file S1. *mecA* positives are indicated with squares, PVL-positives with crosses.

In order to visualise similarities (although not necessarily true phylogenetic relationships) between the hybridisation profiles, a network tree (Figure 1) was constructed using SplitsTree software [13]. Array hybridisation profiles of the tested isolates (see File S1) were converted into a series of ‘sequences’. Each position in this ‘sequence’, *i.e.*, each target gene or allele, could have a value of ‘positive’ (‘C’), ‘negative’ (‘G’) or ‘ambiguous’ (‘A’). These ‘sequences’ were used with SplitsTree version 4.11.3 on default settings (characters transformation: uncorrected P/ignore ambiguous states, distance transformation: Neighbour-Net, and variance: ordinary least squares).

## Results

294 clinical isolates collected in 2012/2013 were typed by microarray hybridisation. Demographic data as well as full array hybridisation profiles are provided as File S1.

### Resistance markers

An overview on abundances of resistance genes is given in Table 1.

**Table 1:** Genes associated with antimicrobial resistance.

Gene	Gene product/explanation	All isolates	MSSA only	MRSA only
***mecA***	Alternate penicillin binding protein 2, defining MRSA	45 (15.31%)	0	45 (100%)
***SCCmec I***	Cassette chromosome elements SCC*mec*	0	0	0
***SCCmec II***		0	0	0
***SCCmec III***		25 (8.5%)	0	25 (55.56%)
***SCCmec III+ccrA/B-4***		2 (0.68%)	0	2 (4.44%)
***SCCmec IV***		15 (5.10%)	0	15 (33.33%)
***SCCmec IV*** ** (trunc.)**		2 (0.68%)	0	2 (4.44%)
***SCCmec V***		1 (0.34%)	0	1 (2.22%)
***merA/B***	Mercury resistance operon	31 (10.54%)	4 (1.61%)	27 (60%)
***blaZ/I/R***	Beta-lactamase operon	254 (86.39%)	211 (84.74%)	43 (95.56%)
***erm*** **(A)**	Methyltransferases, erythromycin/clindamycin resistance	29 (9.86%)	2 (0.80%)	27 (60%)
***erm*** **(B)**		0	0	0
***erm*** **(C)**		0	0	0
***lnu*** **(A)**	Lincosamid-nucleotidyltransferase	1 (0.34%)	1 (0.4%)	0
***mef*** **(A)**	Macrolide efflux protein A	0	0	0
***mph*** **(C)**	Probable lysylphosphatidylglycerol synthetase	21 (7.14%)	4 (1.61%)	17 (37.78%)
***msr*** **(A)**	Energy dependent efflux of erythromycin	24 (8.16%)	7 (2.81%)	17 (37.78%)
***aacA-aphD***	Bifunctional enzyme Aac/Aph, gentamicin resistance	28 (9.52%)	0	28 (62.22%)
***aadD***	Aminoglycoside adenyltransferase, tobramycin resistance	12 (4.08%)	7 (2.81%)	5 (11.11%)
***aphA3***	Aminoglycoside phosphotransferase, neo-/kanamycin resist.	45 (15.31%)	2 (0.80%)	43 (95.56%)
***fusB***	Fusidic acid resistance gene ( = ***far1***)	1 (0.34%)	1 (0.4%)	0
***fusC***	Fusidic acid resistance gene from “SCC*fus*” elements	2 (0.68%)	2 (0.8%)	0
***mupA***	Mupirocin resistance protein	17 (5.78%)	9 (3.61%)	8 (17.78%)
***tet*** **(K)**	Tetracycline resistance	29 (9.86%)	6 (2.41%)	23 (51.11%)
***tet*** **(M)**	Tetracycline resistance	30 (10.20%)	3 (1.20%)	27 (60%)
***cat***	Chloramphenicol acetyltransferase	2 (0.68%)	1 (0.40%)	1 (2.22%)
***fexA***	Chloramphenicol/florfenicol exporter	0	0	0
***cfr***	Linezolid resistance	0	0	0
***dfrS1***	Dihydrofolate reductase type 1	1 (0.34%)	1 (0.4%)	0
***qacA***	Quaternary ammonium compound resistance protein A	22 (7.48%)	3 (1.2%)	19 (42.22%)
***qacC***	Quaternary ammonium compound resistance protein C	9 (3.06%)	6 (2.41%)	3 (6.67%)
***sat***	Streptothricine-acetyltransferase	45 (15.31%)	2 (0.80%)	43 (95.56%)
***vanA***	Vancomycin resistance gene	0	0	0

A total of 15.31% of the tested isolates proved to be *mecA*-positive. The occurrence of *mecA* across the different lineages is shown in Figure 1. SCC*mec* III was the most common type of SCC*mec* element, found in 27 isolates (all belonging to ST239). All these isolates were also positive for *merA/B*, indicating the presence of a composite SCC*mec* III/SCC*mer* element as previously described [14]. Two of these isolates harboured additional *ccrA/B-4* genes. SCC*mec* IV was detected in 15 isolates that were assigned to the “USA300” strain. Two additional isolates were identical except for a lack of *ccr* genes. SCC*mec* V was found only once in a CC5 strain.

Other common resistance markers were the beta-lactamase operon (*blaZ/I/R*; in 86.39% of isolates), *erm*(A) (in 9.86%, mostly ST239-MRSA-III), *msr*(A)/*mph*(C) (in 8.16% and 7.14%, respectively; mostly associated with “USA300”) and *aphA3/sat* (in 15.31%, largely associated with ST239-MRSA-III and “USA300”). The gentamicin/tobramycin resistance gene *aacA-aphD* occurred in 9.52% of isolates that all belonged to ST239-MRSA-III or “USA300”. A gene associated with mupirocin resistance, *mupA*, was detected in 5.78% of isolates. Among MSSA, the rate of *mupA*-positives was 3.61% (9 out of 249 isolates), while for MRSA it was 17.78% (8 out of 45). Other resistance markers were rare; *vanA* (vancomycin resistance) and *cfr* (linezolid resistance) were not found.

### Virulence genes

An overview on abundances of virulence-associated genes is given in Table 2. Most notably, half (50.00%) of the tested isolates were PVL-positive (Figure 1).

**Table 2:** Genes associated with virulence.

Gene	Gene product/explanation	All isolates	MSSA only	MRSA only
***agrI***	Accessory gene regulator allele I	206 (70.07%)	162 (65.06%)	44 (97.78%)
***agrII***	Accessory gene regulator allele II	36 (12.24%)	35 (14.06%)	1 (2.22%)
***agrIII***	Accessory gene regulator allele III	33 (11.22%)	33 (13.25%)	0
***agrIV***	Accessory gene regulator allele IV	12 (4.08%)	12 (4.82%)	0
***agr -***	Unidentified *agr* allele or negatives	7 (2.38%)	7 (2.81%)	0
***tst1***	Toxic shock syndrome toxin 1	5 (1.70%)	5 (2.01%)	0
***sea***	Enterotoxin A	36 (12.24%)	15 (6.02%)	21 (46.67%)
***sea (N315)/sep***	Enterotoxin A ( = P), allele from N315	14 (4.76%)	14 (5.62%)	0
***seb***	Enterotoxin B	24 (8.16%)	24 (9.64%)	0
***sec/l***	Enterotoxin C and L	20 (6.80%)	20 (8.03%)	0
***sed***	Enterotoxin D	63 (21.43%)	63 (25.30%)	0 (0%)
***see***	Enterotoxin E	0	0	0
***seh***	Enterotoxin H	5 (1.70%)	5 (2.01%)	0
***sej***	Enterotoxin J	64 (21.77%)	64 (25.70%)	0
***sek/q***	Enterotoxin K and Q	110 (37.41%)	67 (26.91%)	43 (95.56%)
***ser***	Enterotoxin R	63 (21.43%)	63 (25.30%)	0
***egc***	*egc* cluster (*seg, sei, selm, seln, selo, selu*)	90 (30.61%)	89 (35.74%)	1 (2.22%)
**ORF CM14**	Enterotoxin homologue	15 (5.10%)	15 (6.02%)	0
***lukF/S*** **-PV**	Panton-Valentine leukocidin	147 (50%)	130 (52.21%)	17 (37.78%)
***sak***	Staphylokinase	255 (86.73%)	218 (87.55%)	37 (82.22%)
***chp***	Chemotaxis inhibiting protein (CHIPS)	158 (53.74%)	142 (57.03%)	16 (35.56%)
***scn***	Staphyl. complement inhibitor	266 (90.48%)	229 (91.97%)	37 (82.22%)
***etA***	Exfoliative toxin serotype A	11 (3.74%)	11 (4.42%)	0
***etB***	Exfoliative toxin serotype B	5 (1.70%)	5 (2.01%)	0
***etD***	Exfoliative toxin D	0	0	0
***edinA***	Epidermal cell differentiation inhibitor	13 (4.42%)	13 (5.22%)	0
***edinB***	Epidermal cell differentiation inhibitor B	27 (9.18%)	27 (10.84%)	0
***edinC***	Epidermal cell differentiation inhibitor C	4 (1.36%)	4 (1.61%)	0
**ACME**	ACME locus	18 (6.12%)	0	18 (40%)
***cna***	Collagen adhesin	136 (46.26%)	109 (43.78%)	27 (60%)
***sasG***	Staphylococcal protein G	198 (67.35%)	153 (61.45%)	45 (100%)
***cap5***	Capsule type 8 alleles	166 (56.46%)	148 (59.44%)	18 (40%)
***cap8***	Capsule type 5 alleles	122 (41.50%)	95 (38.15%)	27 (60%)
***cap -***	Unidentified capsule type or negatives	6 (2.04%)	6 (2.41%)	0

### Typing data

An overview on typing data is provided in Table 3 and in Figure 1. Therefore, clonal complexes and strains as well as their abundances are only discussed shortly here.

**Table 3:** Typing data.

CC	Strain	N (%)
**CC1**	CC1-MSSA	5 (1.70%)
**CC5**	CC5-MSSA	8 (2.72%)
	CC5-MSSA [PVL+]	14 (4.76%)
	CC5-MRSA-V [ACME+]	1 (0.34%)
**CC6**	CC6-MSSA	12 (4.08%)
	CC6-MSSA [PVL+]	2 (0.68%)
**CC7**	CC7-MSSA	4 (1.36%)
**CC8**	CC8-MSSA	13 (4.42%)
	CC8-MSSA [PVL+]	60 (20.41%)
	ST8-MRSA-IV [PVL+/ACME+]	15 (5.10%)
	ST8-MRSA-IV [PVL+/ACME+, truncated SCC*mec*]	2 (0.68%)
	ST72-MSSA	14 (4.76%)
	ST72-MSSA-SCC*fus*	1 (0.34%)
	ST72-MSSA-SCC*fus/kdp*	1 (0.34%)
	ST239-MRSA-III&SCC*mer*	25 (8.50%)
	ST239-MRSA-III+*ccrA/B4*	2 (0.68%)
**CC9**	ST834-MSSA	1 (0.34%)
**CC12**	CC12-MSSA	3 (1.02%)
**CC15**	CC15-MSSA	10 (3.40%)
**CC20**	CC20-MSSA	1 (0.34%)
**CC30**	CC30-MSSA	1 (0.34%)
	CC30-MSSA [PVL+]	26 (8.84%)
**CC45**	CC45-MSSA	10 (3.40%)
**CC97**	CC97-MSSA	5 (1.70%)
**CC101**	CC101-MSSA	3 (1.02%)
**CC121**	CC121-MSSA	12 (4.08%)
**CC152**	CC152-MSSA [PVL+]	28 (9.52%)
**CC188**	CC188-MSSA	5 (1.70%)
**CC398**	CC398-MSSA	2 (0.68%)
**ST1852**	ST1852-MSSA	1 (0.34%)
**“Alien”/**	CC1223/1594-MSSA	1 (0.34%)
**CC75-like strains**	ST2250/2277-MSSA	5 (1.70%)
	ST2596dlv-MSSA	1 (0.34%)

#### Clonal Complex 5 Strains

Twenty-three isolates belonged to CC5. Most (n = 14) were PVL-positive MSSA; one was an ACME-positive MRSA harbouring a SCC*mec* V element.

#### Clonal Complex 8 Strains

CC8 was the most common lineage and it largely contributed to the high prevalence of PVL in the study group. 133 (45.24%; ST72 and ST239 included) of tested isolates belonged to this clonal complex and 20.41% of all isolates tested were PVL-positive CC8-MSSA. These isolates were virtually identical to each other. Out of the 60 isolates, 44 were indistinguishable using the current array, others differed only in some genes known to be localised on mobile elements (*e.g.*, *sed/j/r*, beta-lactamase operon and other resistance markers, genes associated with haemolysin beta-converting phages). Another 15 isolates (5.10%) were assigned to the “USA300” strain (ST8-MRSA-IV [PVL+/ACME+]). Two isolates (0.68%) were closely related to this strain but lacked *ccrA/B-2* genes. 9.18% of isolates belonged to ST239-MRSA-III, or, respectively, to a variant that additionally carried *ccrA/B-4* recombinase genes. The remaining CC8 isolates were either PVL-negative CC8 MSSA *sensu strictu*, or belonged to ST72. ST72 is a distinct lineage that differs from other CC8 strains in several traits such as in the presence of the *egc* enterotoxin gene cluster. All ST72 strains were MSSA although one harboured genes associated with a “SCC*fus*” element (*ccrA/B-1, fusC*), whilst another contained these genes plus the SCC-associated *kdp* (potassium translocating) locus.

#### Clonal complex 152

Roughly one-tenth of the collection, 28 isolates, was assigned to this CC. All were PVL-positive, and all were MSSA. Three of them carried enterotoxin genes *sek* and *seq*, which seems to be an unusual feature in CC152 that has not been noted in previously characterised isolates [8], [9], [15], [16].

#### “Alien”, “Staphylococcus argenteus”-like strains

Seven isolates with deviant hybridisation profiles clustered into three distinct groups that were also confirmed by MLST. One group comprising five isolates yielded MLST sequences as well as hybridisation profiles similar to ST2250/2277. Capsule genes, *agr* genes and the coagulase gene *coA* were not detected. Other species-specific genes such as *spa*, *sbi* or *nuc1* were present as well as some adhesion factors including *sasG*. PVL or enterotoxin genes were absent. All isolates were *mecA*-negative although *blaZ* and *tet*(K) were found occasionally. A further alternate pattern was demonstrated for one isolate that yielded a hybridisation signal with one *agrB* III probe and with probes for *egc* enterotoxin cluster genes. It lacked *sasG* and its hybridisation profile was identical to a previously characterised single locus variant of ST1223 [8]. A third group comprised one isolate displaying hybridisations with probes for *agrB* III and capsule type 5 associated genes. It apparently lacked *coA*, *nuc1*, enterotoxin genes and *sasG*. Its MLST profile was a double locus variant of ST2596 (151-269-20-34-256-261-49).

## Discussion

Typing of pathogenic bacteria is a prerequisite to understanding their population structure and dynamics, and thus to controlling them. This study gives a first insight in the population structure not only of MRSA but also of MSSA for a country for which only few typing data are currently available. Limitations are mainly caused by the need to retrospectively use isolates from diagnostic procedures. It was unfortunately not yet possible to collect prospectively and to obtain a collection of isolates from healthy carriers that could serve as a control group in order to analyse the frequencies of certain virulence markers with regard to clinical presentations. It was also not possible to retrieve full clinical and supporting epidemiological information; this was due to the lack of an electronic system to manage laboratory information in this area.

Generally, the population of *S. aureus* on Trinidad and Tobago might fall into four epidemiological categories that likely originate from different epidemic waves in the past.

First, there are MSSA strains that are related to Western African strains. These include ST72, CC152, and possibly others for which not few data are currently available (such as ST1852), as well as the “alien” or “*S. argenteus*”-like lineages. ST72 has been already found in West Africa [17] and, as MRSA, in the Caribbean [18] and in Latin America [9]. CC152 is known to exist in Gabon [19], Mali [20] and Nigeria [21]. ST1852 has been found in Gabon according to the MLST database (http://saureus.mlst.net/sql/fulldetails.asp?id=3702&send=240). The group of “alien” or “*S. argenteus*”-like lineages were most similar to a cluster of strains observed mainly in Australia that were assigned to MLST lineages ST75, ST883, ST1303 and ST1304. It has been proposed to merge the Australian strains to a new species “*S. argenteus*” [12] based on their lack of the “golden” pigment, or at least to a distinct subspecies. Other notable differences of this lineage compared to “normal” *S. aureus* include the lack of signals for the *agr* regulatory locus used on the array caused by an unknown sequence related to *agr* I and IV alleles [22], although some of the Trinidad isolates yielded *agrB* III signals. Other “alien” or “*S. argenteus*”-like strains have been found in Cambodia, in sporadic (possibly, travel associated) cases from Europe, in French Guayana and, in animals (Straw-Coloured Fruit Bat, *Eidolon helvum*) from Western Africa [23]. Historically, an importation of Western African strains appears plausible given that a large proportion of the population of Trinidad and Tobago has African roots, and that the forced transportation of Africans into the New World during previous centuries also resulted in a transfer of pathogens and diseases, such as malaria, leprosy, smallpox, measles or yellow fever virus [24] into the Americas.

The second group are lineages that are globally spread, *i.e.*, that could have been imported at anytime and from anywhere. Their geographic background and routes of dissemination might in future be elucidated by massive genome sequencing. CC5-, CC8-, CC30- and CC121-MSSA fall in this category; and these lineages have already been observed at Caribbean islands [18]. Most notable is the high prevalence of PVL-positive CC8-MSSA on Trinidad and Tobago. PVL-positive CC8-MSSA were very common on the islands, as noted previously [8], such that the causative agent from a recently reported fatal case of PVL-associated disease [7] was a rather typical specimen. As previously observed, the high prevalence of this strain in the Caribbean and its rarity in other parts of the world might indicate that the pandemic CC8 CA-MRSA strain “USA300” emerged in this region [8], [25]. However, it is not yet clear whether the abundance of PVL-positive CC8-MSSA in Trinidad and Tobago is paralleled on other West Indian/Caribbean islands and in Latin America or if it indicates a local outbreak situation. The extremely high homogeneity of all isolates described in this study (see above) as well as in two previous studies [7], [8] might support the latter theory.

A third group comprises ST239-MRSA-III and its variants. This is a globally spread healthcare-associated strain. It is very common in Trinidad and Tobago and is, or used to be, common in many other countries. Overall, it appears the MRSA rate has been rather constant in Trinidad and Tobago for the last decade, being 12.8% for isolates collected in 2001/2002 [2] and 15.2% in this study. The abundance of one single strain and its remarkable homogeneity suggests an outbreak scenario, *i.e.*, transmission is sustained following an importation event that may have occurred several years ago. This was, for the same strain, observed also in Ireland where ST239-MRSA-III became the predominant strain in the 1980s [26], after repatriation of a single infected patient [27]. Since this strain is strictly hospital-associated, the implementation of stringent hospital infection control measures could substantially reduce the burden of MRSA to the healthcare systems on Trinidad and Tobago [3].

Finally, a forth group consists of recently emerging, possibly community-acquired, MRSA. In Trinidad and Tobago this is almost exclusively the PVL/ACME-positive ST8-MRSA-IV strain “USA300”. Interestingly, on other Caribbean islands, other strains might fulfil this role. A recent study shows the presence of typical “French” CA-MRSA clones on French West Indian islands [28]. This emphasises the importance of travel in the intercontinental transfer of epidemic clones. As mentioned above, it has been suspected that “USA300” emerged in this part of the world. However it is not yet clear where and when the SCC*mec* and the ACME elements were acquired. While ACME-negative PVL-positive ST8-MRSA-IV are often associated with Spain or Latin America, all isolates from Trinidad and Tobago were ACME-positives, *i.e.*, more likely to be linked to the U.S. or other regions of origin. In order to assess the significance of ACME as an epidemiological marker and its possible correlation with geographic background, more data are still needed, especially from other Caribbean countries.

Open questions for further studies are whether CA-MRSA, *i.e.*, “USA300”, can replace the related PVL-positive CC8-MSSA in direct competition under community conditions. This could be of high relevance for public health because such a development could render standard treatments ineffective. Another issue for future study is if “USA300” can compete with hospital-associated MRSA strains in hospitals and if it can spread in healthcare settings. In Northern America this was apparently the case, while in Germany or the U.K. one predominant strain (CC22-MRSA-IV, “Barnim Epidemic Strain/UK-EMRSA-15”) is by now so firmly rooted in healthcare facilities that it apparently hinders the spread of other strains including “USA300”. Currently, the rate of “USA300” in Trinidad and Tobago appears to be increasing. In a previous study, only three out of 80 MRSA isolates (3.75%) belonged to that strain [3]. In the present study, 17 out of the 45 MRSA isolates (37.78%) belonged to “USA300” (or to a *ccrA/B-2*-negative variant thereof). This warrants further studies and on-going surveillance. Future epidemiological studies should also focus on the population outside of healthcare facilities because a high burden of infections with both, MSSA and MRSA, can be expected in community settings in Trinidad and Tobago.

## Supporting Information

File S1 **Full demographic data and hybridisation profiles.**
(PDF)
