# Supplementary material for: Population Structure of Staphylococcus aureus from Trinidad & Tobago
Source: PLoS One. 2014 Feb 19;9(2):e89120. doi: 10.1371/journal.pone.0089120 (PMC3929661; doi:10.1371/journal.pone.0089120)
Supplement: File S1 — Full demographic data and hybridisation profiles. (PDF) [file pone.0089120.s001.pdf]

Strain / Isolates		MLST profile	Clinical / demographic data		Domain 1 of 23S-rRNA	glyceraldehyde 3-phosphate dehydrogenase locus 1	katalase A	coagulase	thermostable extracellular nuclease	staphylococcal protein A	IgG-binding protein	staphylococcal accessory regulator A	histidine protein kinase, sae locus	sensor protein	accessory gene regulator allele I				accessory gene regulator allele II				accessory gene regulator allele III				accessory gene regulator allele IV			haemolysin delta	alternate penicillin binding protein 2, defining MRSA	truncated signal transducer protein MecR1	glycerophosphoryl diester phosphodiesterase, associated with mecA	cassette chromosome recombinase genes A/B-1	
			Patient	Sample type, Diagnosis	rrnD1 (S. aureus)	gapA	katA	coA	nuc1	spa	sbi	sarA	saeS	vraS	agrI (total)	agrB-I	agrC-I	agrD-I	agrII (total)	agrB-II	agrC-II	agrD-II	agrIII (total)	agrB-III	agrC-III	agrD-III	agrIV (total)	agrB-IV	agrC-IV	hld	mecA	delta_mecR	ugpQ	ccrA-1	ccrB-1
**CC1-MSSA**	**CC1-MSSA**				N= 5		%= 1.70																												
2012_257_Z3544 (3216/12)	2012-257		4 years old, MALE	FEVER, COUGH, RT SIDE CHEST PAIN BLOOD CULTURE	POS	POS	POS	POS	POS	POS	POS	POS	POS	POS	NEG	NEG	NEG	NEG	NEG	NEG	NEG	NEG	POS	POS	POS	POS	NEG	NEG	NEG	POS	NEG	NEG	NEG	NEG	NEG
2012_281_Z3568 (4696/12)	2012-281		age unknown, MALE	PERIANAL & GROIN ABSCESS	POS	POS	POS	POS	POS	POS	POS	POS	POS	POS	NEG	NEG	NEG	NEG	NEG	NEG	NEG	NEG	POS	POS	POS	POS	NEG	NEG	NEG	POS	NEG	NEG	NEG	NEG	NEG
2012_324_Z3611 (7180/12)	2012-324		age unknown, MALE	SWAB	POS	POS	POS	POS	POS	POS	POS	POS	POS	POS	NEG	NEG	NEG	NEG	NEG	NEG	NEG	NEG	POS	POS	POS	POS	NEG	NEG	NEG	POS	NEG	NEG	NEG	NEG	NEG
2012_327_Z3614 (7347/12)	2012-327		12 years old, MALE	BLOOD CULTURE	POS	POS	POS	POS	POS	POS	POS	POS	POS	POS	NEG	NEG	NEG	NEG	NEG	NEG	NEG	NEG	POS	POS	POS	POS	NEG	NEG	NEG	POS	NEG	NEG	NEG	NEG	NEG
2012_351_Z3638 (8273B/12)	2012-351		36 years old, MALE with HTN/ESRD/DM	SEPSISBLOOD CULTURE	POS	POS	POS	POS	POS	POS	POS	POS	POS	POS	NEG	NEG	NEG	NEG	NEG	NEG	NEG	NEG	POS	POS	POS	POS	NEG	NEG	NEG	POS	NEG	NEG	NEG	NEG	NEG
**CC5-MSSA**	**CC5-MSSA**				N= 8		%= 2.72																												
2012_188_Z3505 (503/12)	2012-188		age unknown, gender not recorded	URINE CULTURE	POS	POS	POS	POS	POS	POS	POS	POS	POS	POS	NEG	NEG	NEG	NEG	POS	POS	POS	POS	NEG	NEG	NEG	NEG	NEG	NEG	NEG	POS	NEG	NEG	NEG	NEG	NEG
2012_219_Z3518 (1437/12)	2012-219		age unknown, MALE	ASPIRATE	POS	POS	POS	POS	POS	POS	POS	POS	POS	POS	NEG	NEG	NEG	NEG	POS	POS	POS	POS	NEG	NEG	NEG	NEG	NEG	NEG	NEG	POS	NEG	NEG	NEG	NEG	NEG
2012_329_Z3616 (7577/12)	2012-329		1 week old, gender not recorded	FEVER, SEPSIS UMBILICAL SWAB	POS	POS	POS	POS	POS	POS	POS	POS	POS	POS	NEG	NEG	NEG	NEG	POS	POS	POS	POS	NEG	NEG	NEG	NEG	NEG	NEG	NEG	POS	NEG	NEG	NEG	NEG	NEG
2012_377_Z3437 (6242)	2012-377		age unknown, FEMALE	BLOOD	POS	POS	POS	POS	POS	POS	POS	POS	POS	POS	NEG	NEG	NEG	NEG	POS	POS	POS	POS	NEG	NEG	NEG	NEG	NEG	NEG	NEG	POS	NEG	NEG	NEG	NEG	NEG
2013_PA04_Z3987	2013-004		76 years old, MALE	WS DEVICE EXTRUSION SITE	POS	POS	POS	POS	POS	POS	POS	POS	POS	POS	NEG	NEG	NEG	NEG	POS	POS	POS	POS	NEG	NEG	NEG	NEG	NEG	NEG	NEG	POS	NEG	NEG	NEG	NEG	NEG
2013_PA32_Z4015	2013-032			BLOOD	POS	POS	POS	POS	POS	POS	POS	POS	POS	POS	NEG	NEG	NEG	NEG	POS	POS	POS	POS	NEG	NEG	NEG	NEG	NEG	NEG	NEG	POS	NEG	NEG	NEG	NEG	NEG
2013_PA41_Z4024	2013-041		11 years old, MALE	WS ABSCESS	POS	POS	POS	POS	POS	POS	POS	POS	POS	POS	NEG	NEG	NEG	NEG	POS	POS	POS	POS	NEG	NEG	NEG	NEG	NEG	NEG	NEG	POS	NEG	NEG	NEG	NEG	NEG
2013_PA44_Z4027	2013-044		10 years old, MALE	SWAB RIGHT THIGH, Post mortem	POS	POS	POS	POS	POS	POS	POS	POS	POS	POS	NEG	NEG	NEG	NEG	POS	POS	POS	POS	NEG	NEG	NEG	NEG	NEG	NEG	NEG	POS	NEG	NEG	NEG	NEG	NEG
**CC5-MSSA [PVL+]**	**CC5-MSSA [PVL+]**				N= 14		%= 4.76																												
2012_175_Z3497 (112/12)	2012-175		9 months old, FEMALE	MULTIPLE SWAB ABSCESS SWABS	POS	POS	POS	POS	POS	POS	POS	POS	POS	POS	NEG	NEG	NEG	NEG	POS	POS	POS	POS	NEG	NEG	NEG	NEG	NEG	NEG	NEG	POS	NEG	NEG	NEG	NEG	NEG
2012_193_Z3458 (617/12)	2012-193		19 years old, MALE	LEFT POSTERIOR THIGH ABSCESS SWAB	POS	POS	POS	POS	POS	POS	POS	POS	POS	POS	NEG	NEG	NEG	NEG	POS	POS	POS	POS	NEG	NEG	NEG	NEG	NEG	NEG	NEG	POS	NEG	NEG	NEG	NEG	NEG
2012_215_Z3474 (1341/12)	2012-215		6 years old, MALE	SWAB	POS	POS	POS	POS	POS	POS	POS	POS	POS	POS	NEG	NEG	NEG	NEG	POS	POS	POS	POS	NEG	NEG	NEG	NEG	NEG	NEG	NEG	POS	NEG	NEG	NEG	NEG	NEG
2012_216_Z3475 (1370/12)	2012-216		age unknown, FEMALE	WOUND SWAB FROM CARBUNCLE	POS	POS	POS	POS	POS	POS	POS	POS	POS	POS	NEG	NEG	NEG	NEG	POS	POS	POS	POS	NEG	NEG	NEG	NEG	NEG	NEG	NEG	POS	NEG	NEG	NEG	NEG	NEG
2012_297_Z3584 (5140/12)	2012-297		7 years old, gender not recorded	LT AXILLARY ABS SWAB	POS	POS	POS	POS	POS	POS	POS	POS	POS	POS	NEG	NEG	NEG	NEG	POS	POS	POS	POS	NEG	NEG	NEG	NEG	NEG	NEG	NEG	POS	NEG	NEG	NEG	NEG	NEG
2012_331_Z3618 (7644/12)	2012-331		age unknown, MALE	WOUND SWAB, FACIAL ABSCESS	POS	POS	POS	POS	POS	POS	POS	POS	POS	POS	NEG	NEG	NEG	NEG	POS	POS	POS	POS	NEG	NEG	NEG	NEG	NEG	NEG	NEG	POS	NEG	NEG	NEG	NEG	NEG
2012_379_Z3439 (4126)	2012-379		10 years old, FEMALE	POST AURICULAR ABSCESSWOUND SWAB	POS	POS	POS	POS	POS	POS	POS	POS	POS	POS	NEG	NEG	NEG	NEG	POS	POS	POS	POS	NEG	NEG	NEG	NEG	NEG	NEG	NEG	POS	NEG	NEG	NEG	NEG	NEG
2012_381_Z3441	2012-381				POS	POS	POS	POS	POS	POS	POS	POS	POS	POS	NEG	NEG	NEG	NEG	POS	POS	POS	POS	NEG	NEG	NEG	NEG	NEG	NEG	NEG	POS	NEG	NEG	NEG	NEG	NEG
2013_PA39_Z4022	2013-039		8 years old, MALE	PUS FROM RIGHT FOREARM ABSCESS	POS	POS	POS	POS	POS	POS	POS	POS	POS	POS	NEG	NEG	NEG	NEG	POS	POS	POS	POS	NEG	NEG	NEG	NEG	NEG	NEG	NEG	POS	NEG	NEG	NEG	NEG	NEG
2013_PA47_Z4030	2013-047		FEMALE	SWAB PUS AXILLARY ABSCESS	POS	POS	POS	POS	POS	POS	POS	POS	POS	POS	NEG	NEG	NEG	NEG	POS	POS	POS	POS	NEG	NEG	NEG	NEG	NEG	NEG	NEG	POS	NEG	NEG	NEG	NEG	NEG
2013_PA61_I_Z4044	2013-061		52 years old, FEMALE	SWAB - ABD WALL PUS	POS	POS	POS	POS	POS	POS	POS	POS	POS	POS	NEG	NEG	NEG	NEG	POS	POS	POS	POS	NEG	NEG	NEG	NEG	NEG	NEG	NEG	POS	NEG	NEG	NEG	NEG	NEG
2013_PA62_I_Z4045	2013-062		14 years old, MALE	SWAB LEFT LEG ABSCESS	POS	POS	POS	POS	POS	POS	POS	POS	POS	POS	NEG	NEG	NEG	NEG	POS	POS	POS	POS	NEG	NEG	NEG	NEG	NEG	NEG	NEG	POS	NEG	NEG	NEG	NEG	NEG
2013_PA74_Z4057	2013-074		28 years old, FEMALE	SWAB LEFT ARM SWELLING	POS	POS	POS	POS	POS	POS	POS	POS	POS	POS	NEG	NEG	NEG	NEG	POS	POS	POS	POS	NEG	NEG	NEG	NEG	NEG	NEG	NEG	POS	NEG	NEG	NEG	NEG	NEG
2013_PA75_Z4058	2013-075				POS	POS	POS	POS	POS	POS	POS	POS	POS	POS	NEG	NEG	NEG	NEG	POS	POS	POS	POS	NEG	NEG	NEG	NEG	NEG	NEG	NEG	POS	NEG	NEG	NEG	NEG	NEG
**CC5-MRSA-V [ACME+]**	**CC5-MRSA-V [ACME+]**				N= 1		%= 0.34																												
2012_373_Z3433 (1204-17312)	2012-373		age unknown, MALE	WOUND SWAB	POS	POS	POS	POS	POS	POS	POS	POS	POS	POS	NEG	NEG	NEG	NEG	POS	POS	POS	POS	NEG	NEG	NEG	NEG	NEG	NEG	NEG	POS	POS	NEG	POS	NEG	NEG
**CC6-MSSA**	**CC6-MSSA**				N= 12		%= 4.08																												
2012_173_Z3450 (98A/12)	2012-173		56 years old, MALE	BLOOD CULTURE	POS	POS	POS	POS	POS	POS	POS	POS	POS	POS	POS	POS	POS	POS	NEG	NEG	NEG	NEG	NEG	NEG	NEG	NEG	AMB	POS	NEG	POS	NEG	NEG	NEG	NEG	NEG
2012_176_Z3498	2012-176				POS	POS	POS	POS	POS	POS	POS	POS	POS	POS	POS	POS	POS	POS	NEG	NEG	NEG	NEG	NEG	NEG	NEG	NEG	AMB	POS	NEG	POS	NEG	NEG	NEG	NEG	NEG
2012_181_Z3452	2012-181				POS	POS	POS	POS	POS	POS	POS	POS	POS	POS	POS	POS	POS	POS	NEG	NEG	NEG	NEG	NEG	NEG	NEG	NEG	AMB	POS	NEG	POS	NEG	NEG	NEG	NEG	NEG
2012_259_Z3546 (3279/12)	2012-259		46 years old, FEMALE with NEUROFIBROSIS	WOUND SWAB	POS	POS	POS	POS	POS	POS	POS	POS	POS	POS	POS	POS	POS	POS	NEG	NEG	NEG	NEG	NEG	NEG	NEG	NEG	POS	POS	NEG	POS	NEG	NEG	NEG	NEG	NEG
2012_264_Z3551 (3407B/12)	2012-264		20-29 years old, FEMALE with Sickle Cell Anaemia, H	UTI, URINE	POS	POS	POS	POS	POS	POS	POS	POS	POS	POS	POS	POS	POS	POS	NEG	NEG	NEG	NEG	NEG	NEG	NEG	NEG	AMB	POS	NEG	POS	NEG	NEG	NEG	NEG	NEG
2012_273_Z3560 (3844/12)	2012-273		age unknown, MALE	SWAB	POS	POS	POS	POS	POS	POS	POS	POS	POS	POS	POS	POS	POS	POS	NEG	NEG	NEG	NEG	NEG	NEG	NEG	NEG	POS	POS	NEG	POS	NEG	NEG	NEG	NEG	NEG
2012_312_Z3599 (6815/12)	2012-312		2 months old, MALE	EYE SWAB	POS	POS	POS	POS	POS	POS	POS	POS	POS	POS	POS	POS	POS	POS	NEG	NEG	NEG	NEG	NEG	NEG	NEG	NEG	AMB	POS	NEG	POS	NEG	NEG	NEG	NEG	NEG
2012_338_Z3625	2012-338				POS	POS	POS	POS	POS	POS	POS	POS	POS	POS	POS	POS	POS	POS	NEG	NEG	NEG	NEG	NEG	NEG	NEG	NEG	AMB	POS	NEG	POS	NEG	NEG	NEG	NEG	NEG
2012_352_Z3639	2012-352				POS	POS	POS	POS	POS	POS	POS	POS	POS	POS	POS	POS	POS	POS	NEG	NEG	NEG	NEG	NEG	NEG	NEG	NEG	POS	POS	NEG	POS	NEG	NEG	NEG	NEG	NEG
2012_368_Z3428	2012-368				POS	POS	POS	POS	POS	POS	POS	POS	POS	POS	POS	POS	POS	POS	NEG	NEG	NEG	NEG	NEG	NEG	NEG	NEG	AMB	POS	NEG	POS	NEG	NEG	NEG	NEG	NEG
2013_PA12_Z3995	2013-012		24 years old, FEMALE	WOUND SWAB	POS	POS	POS	POS	POS	POS	POS	POS	POS	POS	POS	POS	POS	POS	NEG	NEG	NEG	NEG	NEG	NEG	NEG	NEG	AMB	POS	NEG	POS	NEG	NEG	NEG	NEG	NEG
2013_PA28_Z4011	2013-028		53 years old, FEMALE	WOUND SWAB RIGHT FOOT	POS	POS	POS	POS	POS	POS	POS	POS	POS	POS	POS	POS	POS	POS	NEG	NEG	NEG	NEG	NEG	NEG	NEG	NEG	AMB	POS	NEG	POS	NEG	NEG	NEG	NEG	NEG
**CC6-MSSA [PVL+]**	**CC6-MSSA [PVL+]**				N= 2		%= 0.68																												
2012_375_Z3435	2012-375				POS	POS	POS	POS	POS	POS	POS	POS	POS	POS	POS	POS	POS	POS	NEG	NEG	NEG	NEG	NEG	NEG	NEG	NEG	AMB	POS	NEG	POS	NEG	NEG	NEG	NEG	NEG
2013_PA77_Z4060	2013-077				POS	POS	POS	POS	POS	POS	POS	POS	POS	POS	POS	POS	POS	POS	NEG	NEG	NEG	NEG	NEG	NEG	NEG	NEG	AMB	POS	NEG	POS	NEG	NEG	NEG	NEG	NEG
**CC7-MSSA**	**CC7-MSSA**				N= 4		%= 1.36																												
2012_248_Z3532 (2864/12)	2012-248		age unknown, FEMALE	LOWER RESPIRATORY TRACT SPUTUM	POS	POS	POS	POS	POS	POS	POS	POS	POS	POS	POS	POS	POS	POS	NEG	NEG	NEG	NEG	NEG	NEG	NEG	NEG	POS	POS	NEG	POS	NEG	NEG	NEG	NEG	NEG
2012_265_Z3552 (3518/12)	2012-265		9 weeks old, MALE	SWAB	POS	POS	POS	POS	POS	POS	POS	POS	POS	POS	POS	POS	POS	POS	NEG	NEG	NEG	NEG	NEG	NEG	NEG	NEG	POS	POS	NEG	POS	NEG	NEG	NEG	NEG	NEG
2013_PA53_Z4036	2013-053		1 week old, MALE	RIGHT EYE SWAB	POS	POS	POS	POS	POS	POS	POS	POS	POS	POS	POS	POS	POS	POS	NEG	NEG	NEG	NEG	NEG	NEG	NEG	NEG	AMB	POS	NEG	POS	NEG	NEG	NEG	NEG	AMB
2013_PA83_Z4066	2013-083				POS	POS	POS	POS	POS	POS	POS	POS	POS	POS	POS	POS	POS	POS	NEG	NEG	NEG	NEG	NEG	NEG	NEG	NEG	AMB	POS	NEG	POS	NEG	NEG	NEG	NEG	NEG
**CC8-MSSA**	**CC8-MSSA**				N= 13		%= 4.42																												
2012_243_Z3528 (2587/12)	2012-243		15 years old, gender not recorded	WOUND SWAB	POS	POS	POS	POS	POS	POS	POS	POS	POS	POS	POS	POS	POS	POS	NEG	NEG	NEG	NEG	NEG	NEG	NEG	NEG	AMB	POS	NEG	POS	NEG	NEG	NEG	NEG	NEG
2012_344_Z3631 (8605/12)	2012-344		3 months old, MALE	UMBILICAL SWAB	POS	POS	POS	POS	POS	POS	POS	POS	POS	POS	POS	POS	POS	POS	NEG	NEG	NEG	NEG	NEG	NEG	NEG	NEG	AMB	POS	NEG	POS	NEG	NEG	NEG	NEG	NEG
2012_346_Z3633 (8074/12)	2012-346		age unknown, gender not recorded	SWAB	POS	POS	POS	POS	POS	POS	POS	POS	POS	POS	POS	POS	POS	POS	NEG	NEG	NEG	NEG	NEG	NEG	NEG	NEG	AMB	POS	NEG	POS	NEG	NEG	NEG	NEG	NEG
2012_255_Z3538 (3205B/12)	2012-255		age unknown, MALE	BLOOD CULTURE	POS	POS	POS	POS	POS	POS	POS	POS	POS	POS	POS	POS	POS	POS	NEG	NEG	NEG	NEG	NEG	NEG	NEG	NEG	AMB	POS	NEG	POS	NEG	NEG	NEG	NEG	NEG
2012_280_Z3567 (4601/12)	2012-280		48 years old, FEMALE	URINE CULTURE	POS	POS	POS	POS	POS	POS	POS	POS	POS	POS	POS	POS	POS	POS	NEG	NEG	NEG	NEG	NEG	NEG	NEG	NEG	POS	POS	NEG	POS	NEG	NEG	NEG	NEG	NEG
Trinidad&Tobago_2012_267 (3642/12)	2012-267		1 week old, gender not recorded	EYE SWAB	POS	POS	POS	POS	POS	POS	POS	POS	POS	POS	POS	POS	POS	POS	NEG	NEG	NEG	NEG	NEG	NEG	NEG	NEG	NEG	POS	NEG	POS	NEG	NEG	NEG	NEG	NEG
2012_365_Z3425 (1204-15838)	2012-365		age unknown, gender not recorded	EYE SWAB	POS	POS	POS	POS	POS	POS	POS	POS	POS	POS	POS	POS	POS	POS	NEG	NEG	NEG	NEG	NEG	NEG	NEG	NEG	AMB	POS	NEG	POS	NEG	NEG	NEG	NEG	NEG
2012_296_Z3583 (5118/12)	2012-296		13 years old, MALE	SWAB	POS	POS	POS	POS	POS	POS	POS	POS	POS	POS	POS	POS	POS	POS	NEG	NEG	NEG	NEG	NEG	NEG	NEG	NEG	AMB	POS	NEG	POS	NEG	NEG	NEG	NEG	NEG
2012_318_Z3605 (6960/12)	2012-318		75 years old, MALE	BLOOD CULTURE	POS	POS	POS	POS	POS	POS	POS	POS	POS	POS	POS	POS	POS	POS	NEG	NEG	NEG	NEG	NEG	NEG	NEG	NEG	AMB	POS	NEG	POS	NEG	NEG	NEG	NEG	NEG
2012_306_Z3593 (5861/12)	2012-306		25 years old, FEMALE	HIGH VAGINAL SWAB	POS	POS	POS	POS	POS	POS	POS	POS	POS	POS	POS	POS	POS	POS	NEG	NEG	NEG	NEG	NEG	NEG	NEG	NEG	POS	POS	NEG	POS	NEG	NEG	NEG	NEG	NEG
2013_PA37_Z4020	2013-037		45 years old, FEMALE	SWAB CATH SITE	POS	POS	POS	POS	POS	POS	POS	POS	POS	POS	POS	POS	POS	POS	NEG	NEG	NEG	NEG	NEG	NEG	NEG	NEG	AMB	POS	NEG	POS	NEG	NEG	NEG	NEG	NEG
2013_PA38_Z4021	2013-038		44 years old, FEMALE	PLEURAL FLUID	POS	POS	POS	POS	POS	POS	POS	POS	POS	POS	POS	POS	AMB	POS	NEG	NEG	NEG	NEG	NEG	NEG	NEG	NEG	NEG	NEG	NEG	POS	NEG	NEG	NEG	NEG	NEG
2013_PA52_Z4035	2013-052		6 years old, FEMALE	SWAB PUSTULES FROM LIMBS	POS	POS	POS	POS	POS	POS	POS	POS	POS	POS	POS	POS	POS	POS	NEG	NEG	NEG	NEG	NEG	NEG	NEG	NEG	NEG	AMB	NEG	POS	NEG	NEG	NEG	NEG	NEG
**CC8-MSSA [PVL+]**	**CC8-MSSA [PVL+]**				N= 60		%= 20.41																												
2012_156_Z3489 (25917/11)	2012-156		age unknown, gender not recorded	BLOOD CULTURE	POS	POS	POS	POS	POS	POS	POS	POS	POS	POS	POS	POS	POS	POS	NEG	NEG	NEG	NEG	NEG	NEG	NEG	NEG	AMB	POS	NEG	POS	NEG	NEG	NEG	NEG	NEG
2012_159_Z3492 (26310/11)	2012-159		age unknown, MALE	SWAB	POS	POS	POS	POS	POS	POS	POS	POS	POS	POS	POS	POS	POS	POS	NEG	NEG	NEG	NEG	NEG	NEG	NEG	NEG	AMB	POS	NEG	POS	NEG	NEG	NEG	NEG	NEG
2012_161_Z3445 (26430/11)	2012-161		age unknown, MALE	SWAB	POS	POS	POS	POS	POS	POS	POS	POS	POS	POS	POS	POS	POS	POS	NEG	NEG	NEG	NEG	NEG	NEG	NEG	NEG	AMB	POS	NEG	POS	NEG	NEG	NEG	NEG	NEG
2012_162_Z3512 (26433/11)	2012-162		age unknown, gender not recorded	SWAB	POS	POS	POS	POS	POS	POS	POS	POS	POS	POS	POS	POS	POS	POS	NEG	NEG	NEG	NEG	NEG	NEG	NEG	NEG	AMB	POS	NEG	POS	NEG	NEG	NEG	NEG	NEG
2012_169_Z3495 (27194/11)	2012-169		47 years old, MALE	RIGHT MIDDLE ABSCESS	POS	POS	POS	POS	POS	POS	POS	POS	POS	POS	POS	POS	POS	POS	NEG	NEG	NEG	NEG	NEG	NEG	NEG	NEG	AMB	POS	NEG	POS	NEG	NEG	NEG	NEG	NEG
2012_170_Z3496 (35/12)	2012-170		2 years old, FEMALE	LEFT GLUTEAL ABSCESS	POS	POS	POS	POS	POS	POS	POS	POS	POS	POS	POS	POS	POS	POS	NEG	NEG	NEG	NEG	NEG	NEG	NEG	NEG	AMB	POS	NEG	POS	NEG	NEG	NEG	NEG	NEG
2012_171_Z3448 (47/12)	2012-171		1 months old, FEMALE	EYE DISCHARGE SWAB	POS	POS	POS	POS	POS	POS	POS	POS	POS	POS	POS	POS	POS	POS	NEG	NEG	NEG	NEG	NEG	NEG	NEG	NEG	AMB	POS	NEG	POS	NEG	NEG	NEG	NEG	NEG
2012_177_Z3499 (184/12)	2012-177		age unknown, FEMALE	ABSCESS SWAB	POS	POS	POS	POS	POS	POS	POS	POS	POS	POS	POS	POS	POS	POS	NEG	NEG	NEG	NEG	NEG	NEG	NEG	NEG	AMB	POS	NEG	POS	NEG	NEG	NEG	NEG	NEG
2012_179_Z3515 (231/12)	2012-179		2 weeks old, gender not recorded	RO EARLY ABSCESS	POS	POS	POS	POS	POS	POS	POS	POS	POS	POS	POS	POS	POS	POS	NEG	NEG	NEG	NEG	NEG	NEG	NEG	NEG	AMB	POS	NEG	POS	NEG	NEG	NEG	NEG	NEG
2012_185_Z3502 (437/12)	2012-185		2 weeks old, gender not recorded	RT INGUINAL ABSCESS	POS	POS	POS	POS	POS	POS	POS	POS	POS	POS	POS	POS	POS	POS	NEG	NEG	NEG	NEG	NEG	NEG	NEG	NEG	AMB	POS	NEG	POS	NEG	NEG	NEG	NEG	NEG
2012_191_Z3456 (563/12)	2012-191		4 months old, MALE	WOUND SWAB FROM CHIN ABSCESS	POS	POS	POS	POS	POS	POS	POS	POS	POS	POS	POS	POS	POS	POS	NEG	NEG	NEG	NEG	NEG	NEG	NEG	NEG	AMB	POS	NEG	POS	NEG	NEG	NEG	NEG	NEG
2012_194_Z3459	2012-194				POS	POS	POS	POS	POS	POS	POS	POS	POS	POS	POS	POS	POS	POS	NEG	NEG	NEG	NEG	NEG	NEG	NEG	NEG	AMB	POS	NEG	POS	NEG	NEG	NEG	NEG	NEG
2012_200_Z3460 (820/12)	2012-200		48 years old, MALE	SWAB	POS	POS	POS	POS	POS	POS	POS	POS	POS	POS	POS	POS	POS	POS	NEG	NEG	NEG	NEG	NEG	NEG	NEG	NEG	POS	POS	NEG	POS	AMB	NEG	NEG	NEG	NEG
2012_207_Z3467 (1010/12)	2012-207		15 months old, MALE	WOUND SWAB	POS	POS	POS	POS	POS	POS	POS	POS	POS	POS	POS	POS	POS	POS	NEG	NEG	NEG	NEG	NEG	NEG	NEG	NEG	POS	POS	NEG	POS	NEG	NEG	NEG	NEG	NEG
2012_212_Z3471 (1261/12)	2012-212		age unknown, gender not recorded	BLOOD CULTURE	POS	POS	POS	POS	POS	POS	POS	POS	POS	POS	POS	POS	POS	POS	NEG	NEG	NEG	NEG	NEG	NEG	NEG	NEG	AMB	POS	NEG	POS	NEG	NEG	NEG	NEG	NEG
2012_220_Z3477 (1466/12)	2012-220		age unknown, FEMALE	SWAB FROM EXIT SITE PD CATHE	POS	POS	POS	POS	POS	POS	POS	POS	POS	POS	POS	POS	POS	POS	NEG	NEG	NEG	NEG	NEG	NEG	NEG	NEG	AMB	POS	NEG	POS	NEG	NEG	NEG	NEG	NEG
2012_224_Z3481 (1522/12)	2012-224		11 years old, FEMALE	RT AXILLARY SWAB	POS	POS	POS	POS	POS	POS	POS	POS	POS	POS	POS	POS	POS	POS	NEG	NEG	NEG	NEG	NEG	NEG	NEG	NEG	POS	POS	NEG	POS	NEG	NEG	NEG	NEG	NEG
2012_226_Z3483 (1662/12)	2012-226		8 years old, MALE	SEPSIS, FEVER, BLOOD CULTURE	POS	POS	POS	POS	POS	POS	POS	POS	POS	POS	POS	POS	POS	POS	NEG	NEG	NEG	NEG	NEG	NEG	NEG	NEG	POS	POS	NEG	POS	NEG	NEG	NEG	NEG	NEG
2012_242_Z3527 (2581/12)	2012-242		8 years old, FEMALE	SWAB	POS	POS	POS	POS	POS	POS	POS	POS	POS	POS	POS	POS	POS	POS	NEG	NEG	NEG	NEG	NEG	NEG	NEG	NEG	AMB	POS	NEG	POS	NEG	NEG	NEG	NEG	NEG
2012_245_Z3529 (2687/12)	2012-245		1 years old, FEMALE	FLUID	POS	POS	POS	POS	POS	POS	POS	POS	POS	POS	POS	POS	POS	POS	NEG	NEG	NEG	NEG	NEG	NEG	NEG	NEG	AMB	POS	NEG	POS	NEG	NEG	NEG	NEG	NEG
2012_253_Z3536 (3074/12)	2012-253		60-69 years old, MALE with DM	CELLULITIS, WOUND SWAB	POS	POS	POS	POS	POS	POS	POS	POS	POS	POS	POS	POS	POS	POS	NEG	NEG	NEG	NEG	NEG	NEG	NEG	NEG	POS	POS	NEG	POS	NEG	NEG	NEG	NEG	NEG
2012_258_Z3545 (3231/12)	2012-258		28 years old, FEMALE	STEVENS JOHNSON SYNDROME WOUND SWAB	POS	POS	POS	POS	POS	POS	POS	POS	POS	POS	POS	POS	POS	POS	NEG	NEG	NEG	NEG	NEG	NEG	NEG	NEG	POS	POS	NEG	POS	NEG	NEG	NEG	NEG	NEG
2012_261_Z3548 (3298/12)	2012-261		19 years old, FEMALE	PERIANAL ABS SWAB	POS	POS	POS	POS	POS	POS	POS	POS	POS	POS	POS	POS	POS	POS	NEG	NEG	NEG	NEG	NEG	NEG	NEG	NEG	POS	POS	NEG	POS	NEG	NEG	NEG	NEG	NEG
2012_270_Z3557 (3798/12)	2012-270		35 years old, MALE	CELLULITIS ELBOW, PUS SWAB	POS	POS	POS	POS	POS	POS	POS	POS	POS	POS	POS	POS	POS	POS	NEG	NEG	NEG	NEG	NEG	NEG	NEG	NEG	POS	POS	NEG	POS	NEG	NEG	NEG	NEG	NEG
2012_274_Z3561 (3861/12)	2012-274		14 years old, MALE	BLOOD CULTURE	POS	POS	POS	POS	POS	POS	POS	POS	POS	POS	POS	POS	POS	POS	NEG	NEG	NEG	NEG	NEG	NEG	NEG	NEG	POS	POS	NEG	POS	NEG	NEG	NEG	NEG	NEG
2012_282_Z3569 (4698/12)	2012-282		8 years old, MALE	GLUTEAL ABS SWAB	POS	POS	POS	POS	POS	POS	POS	POS	POS	POS	POS	POS	POS	POS	NEG	NEG	NEG	NEG	NEG	NEG	NEG	NEG	AMB	POS	NEG	POS	NEG	NEG	NEG	NEG	NEG
2012_286_Z3573 (4805/12)	2012-286		4 years old, MALE	WOUND SWAB	POS	POS	POS	POS	POS	POS	POS	POS	POS	POS	POS	POS	POS	POS	NEG	NEG	NEG	NEG	NEG	NEG	NEG	NEG	AMB	POS	NEG	POS	NEG	NEG	NEG	NEG	NEG
2012_293_Z3580 (5007/12)	2012-293		17 months old, MALE	ANTERIOR ABS SWAB	POS	POS	POS	POS	POS	POS	POS	POS	POS	POS	POS	POS	POS	POS	NEG	NEG	NEG	NEG	NEG	NEG	NEG	NEG	AMB	POS	NEG	POS	NEG	NEG	NEG	NEG	NEG
2012_294_Z3581	2012-294				POS	POS	POS	POS	POS	POS	POS	POS	POS	POS	POS	POS	POS	POS	NEG	NEG	NEG	NEG	NEG	NEG	NEG	NEG	AMB	POS	NEG	POS	NEG	NEG	NEG	NEG	NEG
2012_307_Z3594 (5918/12)	2012-307		9 years old, gender not recorded	PUS DRAINING R FOOT SWAB	POS	POS	POS	POS	POS	POS	POS	POS	POS	POS	POS	POS	POS	POS	NEG	NEG	NEG	NEG	NEG	NEG	NEG	NEG	POS	POS	NEG	POS	NEG	NEG	NEG	NEG	NEG
2012_333_Z3620 (8101/12)	2012-333		age unknown, FEMALE	VP SHUNT	POS	POS	POS	POS	POS	POS	POS	POS	POS	POS	POS	POS	POS	POS	NEG	NEG	NEG	NEG	NEG	NEG	NEG	NEG	AMB	POS	NEG	POS	NEG	NEG	NEG	NEG	NEG
2012_343_Z3630 (8195/12)	2012-343		age unknown, FEMALE	SWAB	POS	POS	POS	POS	POS	POS	POS	POS	POS	POS	POS	POS	POS	POS	NEG	NEG	NEG	NEG	NEG	NEG	NEG	NEG	AMB	POS	NEG	POS	NEG	NEG	NEG	NEG	NEG
2012_349_Z3636 (7999A/12)	2012-349		age unknown, gender not recorded	VEIN BLOOD CULTURE	POS	POS	POS	POS	POS	POS	POS	POS	POS	POS	POS	POS	POS	POS	NEG	NEG	NEG	NEG	NEG	NEG	NEG	NEG	POS	POS	NEG	POS	AMB	AMB	AMB	NEG	NEG
2012_353_Z3640 (8575/12)	2012-353		8 years old, MALE	LEFT FOOT ABSCESS, SWAB	POS	POS	POS	POS	POS	POS	POS	POS	POS	POS	POS	POS	POS	POS	NEG	NEG	NEG	NEG	NEG	NEG	NEG	NEG	POS	POS	NEG	POS	NEG	NEG	NEG	NEG	NEG
2012_354_Z3641 (8408/12)	2012-354		age unknown, MALE	LEFT EYE PUS, EYE SWAB	POS	POS	POS	POS	POS	POS	POS	POS	POS	POS	POS	POS	POS	POS	NEG	NEG	NEG	NEG	NEG	NEG	NEG	NEG	POS	POS	NEG	POS	AMB	NEG	NEG	NEG	NEG
2012_355_Z3642 (1112-58348)	2012-355		age unknown, FEMALE	SEPSIS BLOOD CULTURE	POS	POS	POS	POS	POS	POS	POS	POS	POS	POS	POS	POS	POS	POS	NEG	NEG	NEG	NEG	NEG	NEG	NEG	NEG	AMB	POS	NEG	POS	NEG	NEG	NEG	NEG	NEG
2012_357_Z3417 (1202-6360)	2012-357		age unknown, FEMALE	WOUND SWAB	POS	POS	POS	POS	POS	POS	POS	POS	POS	POS	POS	POS	POS	POS	NEG	NEG	NEG	NEG	NEG	NEG	NEG	NEG	AMB	POS	NEG	POS	NEG	NEG	NEG	NEG	NEG
2012_364_Z3424 (1112-60417)	2012-364		age unknown, gender not recorded	SKIN SWAB	POS	POS	POS	POS	POS	POS	POS	POS	POS	POS	POS	POS	POS	POS	NEG	NEG	NEG	NEG	NEG	NEG	NEG	NEG	AMB	POS	NEG	POS	NEG	NEG	NEG	NEG	NEG
2012_376_Z3436 (6256)	2012-376		1,5 years old, FEMALE	NASAL	POS	POS	POS	POS	POS	POS	POS	POS	POS	POS	POS	POS	POS	POS	NEG	NEG	NEG	NEG	NEG	NEG	NEG	NEG	AMB	POS	NEG	POS	NEG	NEG	NEG	NEG	NEG
2012_383_Z3443	2012-383				POS	POS	POS	POS	POS	POS	POS	POS	POS	POS	POS	POS	POS	POS	NEG	NEG	NEG	NEG	NEG	NEG	NEG	NEG	POS	POS	NEG	POS	NEG	NEG	NEG	NEG	NEG
2013_PA01_Z3984	2013-001		1 year 11 months years old, MALE	WOUND SWAB LEFT GLUTEAL ABSCESS	POS	POS	POS	POS	POS	POS	POS	POS	POS	POS	POS	POS	POS	POS	NEG	NEG	NEG	NEG	NEG	NEG	NEG	NEG	AMB	POS	NEG	POS	NEG	NEG	NEG	NEG	NEG
2013_PA08_Z3991	2013-008		5 years old, FEMALE	SWAB SCALP ABSCESS	POS	POS	POS	POS	POS	POS	POS	POS	POS	POS	POS	POS	POS	POS	NEG	NEG	NEG	NEG	NEG	NEG	NEG	NEG	AMB	POS	NEG	POS	NEG	NEG	NEG	NEG	NEG
2013_PA10_Z3993	2013-010		36 years old, MALE	SWAB LEFT ELBOW ABSCESS	POS	POS	POS	POS	POS	POS	POS	POS	POS	POS	POS	POS	POS	POS	NEG	NEG	NEG	NEG	NEG	NEG	NEG	NEG	AMB	POS	NEG	POS	NEG	NEG	NEG	NEG	NEG
2013_PA13_Z3996	2013-013		age unknown, FEMALE	WOUND SWAB	POS	POS	POS	POS	POS	POS	POS	POS	POS	POS	POS	POS	POS	POS	NEG	NEG	NEG	NEG	NEG	NEG	NEG	NEG	AMB	POS	NEG	POS	NEG	NEG	NEG	NEG	NEG
2013_PA14_Z3997	2013-014		years old, MALE	WOUND SWAB	POS	POS	POS	POS	POS	POS	POS	POS	POS	POS	POS	POS	POS	POS	NEG	NEG	NEG	NEG	NEG	NEG	NEG	NEG	AMB	POS	NEG	POS	NEG	NEG	NEG	NEG	NEG
2013_PA17_Z4000	2013-017		32 years old, MALE	WS	POS	POS	POS	POS	POS	POS	POS	POS	POS	POS	POS	POS	POS	POS	NEG	NEG	NEG	NEG	NEG	NEG	NEG	NEG	AMB	POS	NEG	POS	NEG	NEG	NEG	NEG	NEG
2013_PA26_Z4009	2013-026		22 years old, MALE	WOUND SWAB RIGHT INDEX FINGER	POS	POS	POS	POS	POS	POS	POS	POS	POS	POS	POS	POS	POS	POS	NEG	NEG	NEG	NEG	NEG	NEG	NEG	NEG	AMB	POS	NEG	POS	NEG	NEG	NEG	NEG	NEG
2013_PA27_Z4010	2013-027		? years old, FEMALE	WOUND ABSCESS	POS	POS	POS	POS	POS	POS	POS	POS	POS	POS	POS	POS	POS	POS	NEG	NEG	NEG	NEG	NEG	NEG	NEG	NEG	AMB	POS	NEG	POS	NEG	NEG	NEG	NEG	NEG
2013_PA42_Z4025	2013-042		22 years old, MALE	SWAB FINGER	POS	POS	POS	POS	POS	POS	POS	POS	POS	POS	POS	POS	POS	POS	NEG	NEG	NEG	NEG	NEG	NEG	NEG	NEG	AMB	POS	NEG	POS	NEG	NEG	NEG	NEG	NEG
2013_PA43_Z4026	2013-043		6 MONTHS old, MALE	SWAB LEFT DELTOID ABSCESS	POS	POS	POS	POS	POS	POS	POS	POS	POS	POS	POS	POS	POS	POS	NEG	NEG	NEG	NEG	NEG	NEG	NEG	NEG	NEG	AMB	NEG	POS	NEG	NEG	NEG	NEG	NEG
2013_PA45_Z4028	2013-045		2 years old, MALE	LEFT EAR SWAB	POS	POS	POS	POS	POS	POS	POS	POS	POS	POS	POS	POS	POS	POS	NEG	NEG	NEG	NEG	NEG	NEG	NEG	NEG	AMB	POS	NEG	POS	NEG	NEG	NEG	NEG	NEG
2013_PA56_Z4039	2013-056		18 months old, FEMALE	SWAB SUPRAPUBIC ABSCESS	POS	POS	POS	POS	POS	POS	POS	POS	POS	POS	POS	POS	POS	POS	NEG	NEG	NEG	NEG	NEG	NEG	NEG	NEG	AMB	POS	NEG	POS	NEG	NEG	NEG	NEG	NEG
2013_PA58_Z4041	2013-058		35 years old, MALE	SWAB PALMER ABSCESS	POS	POS	POS	POS	POS	POS	POS	POS	POS	POS	POS	POS	POS	POS	NEG	NEG	NEG	NEG	NEG	NEG	NEG	NEG	POS	POS	NEG	POS	NEG	NEG	NEG	NEG	NEG
2013_PA64_Z4047	2013-064		1 week old, MALE	SWAB RIGHT MID THIGH ABSCESS	POS	POS	POS	POS	POS	POS	POS	POS	POS	POS	POS	POS	POS	POS	NEG	NEG	NEG	NEG	NEG	NEG	NEG	NEG	AMB	POS	NEG	POS	NEG	NEG	NEG	NEG	NEG
2013_PA66_Z4049	2013-066		40 years old, MALE	SWAB LEFT UPPER LIMB	POS	POS	POS	POS	POS	POS	POS	POS	POS	POS	POS	POS	POS	POS	NEG	NEG	NEG	NEG	NEG	NEG	NEG	NEG	AMB	POS	NEG	POS	NEG	NEG	NEG	NEG	NEG
2013_PA67_Z4050	2013-067		1 years old, MALE	SWAB EYELID ABSCESS	POS	POS	POS	POS	POS	POS	POS	POS	POS	POS	POS	POS	POS	POS	NEG	NEG	NEG	NEG	NEG	NEG	NEG	NEG	AMB	POS	NEG	POS	NEG	NEG	NEG	NEG	NEG
2013_PA69_Z4052	2013-069		19 days old, FEMALE	SWAB BREAST ABSCESS	POS	POS	POS	POS	POS	POS	POS	POS	POS	POS	POS	POS	POS	POS	NEG	NEG	NEG	NEG	NEG	NEG	NEG	NEG	AMB	POS	NEG	POS	NEG	NEG	NEG	NEG	NEG
2013_PA72_Z4055	2013-072		2 years old, FEMALE	SWAB SCALP ABSCESS	POS	POS	POS	POS	POS	POS	POS	POS	POS	POS	POS	POS	POS	POS	NEG	NEG	NEG	NEG	NEG	NEG	NEG	NEG	AMB	POS	NEG	POS	NEG	NEG	NEG	NEG	NEG
2013_PA76_Z4059	2013-076				POS	POS	POS	POS	POS	POS	POS	POS	POS	POS	POS	POS	POS	POS	NEG	NEG	NEG	NEG	NEG	NEG	NEG	NEG	AMB	POS	NEG	POS	NEG	NEG	NEG	NEG	NEG
2013_PA80_Z4063	2013-080				POS	POS	POS	POS	POS	POS	POS	POS	POS	POS	POS	POS	POS	POS	NEG	NEG	NEG	NEG	NEG	NEG	NEG	NEG	AMB	POS	NEG	POS	NEG	NEG	NEG	NEG	NEG

Strain / Isolates		MLST profile	Clinical / demographic data		Domain 1 of 23S-rRNA	glyceraldehyde 3-phosphate dehydrogenase locus 1	katalase A	coagulase	thermostable extracellular nuclease	staphylococcal protein A	IgG-binding protein	staphylococcal accessory regulator A	histidine protein kinase, sae locus	sensor protein	accessory gene regulator allele I				accessory gene regulator allele II				accessory gene regulator allele III				accessory gene regulator allele IV			haemolysin delta	alternate penicillin binding protein 2, defining MRSA	truncated signal transducer protein MecR1	glycerophosphoryl diester phosphodiesterase, associated with mecA	cassette chromosome recombinase genes A/B-1	
			Patient	Sample type, Diagnosis	rrnD1 (S. aureus)	gapA	katA	coA	nuc1	spa	sbi	sarA	saeS	vraS	agrI (total)	agrB-I	agrC-I	agrD-I	agrII (total)	agrB-II	agrC-II	agrD-II	agrIII (total)	agrB-III	agrC-III	agrD-III	agrIV (total)	agrB-IV	agrC-IV	hld	mecA	delta_mecR	ugpQ	ccrA-1	ccrB-1
ST8-MRSA-IV, USA300	ST8-MRSA-IV [PVL+/ACME+],				N= 15		%= 5.10																												
2012_158_Z3491 (26049A/11)	2012-158		age unknown, MALE with LYMPHADENOPATHY/DM	SEPSISUTIBLOOD CULTURE	POS	POS	POS	POS	POS	POS	POS	POS	POS	POS	POS	POS	POS	POS	NEG	NEG	NEG	NEG	NEG	NEG	NEG	NEG	AMB	POS	NEG	POS	POS	POS	POS	NEG	NEG
2012_165_Z3513 (26656/11)	2012-165		age unknown, MALE	SWAB	POS	POS	POS	POS	POS	POS	POS	POS	POS	POS	POS	POS	POS	POS	NEG	NEG	NEG	NEG	NEG	NEG	NEG	NEG	AMB	POS	NEG	POS	POS	POS	POS	NEG	NEG
2012_214_Z3473 (1322/12)	2012-214		3 years old, FEMALE	ABSCESS SWAB	POS	POS	POS	POS	POS	POS	POS	POS	POS	POS	POS	POS	POS	POS	NEG	NEG	NEG	NEG	NEG	NEG	NEG	NEG	AMB	POS	NEG	POS	POS	POS	POS	NEG	NEG
2012_227_Z3484 (1688/12)	2012-227		age unknown, MALE	WOUND SWAB	POS	POS	POS	POS	POS	POS	POS	POS	POS	POS	POS	POS	POS	POS	NEG	NEG	NEG	NEG	NEG	NEG	NEG	NEG	POS	POS	NEG	POS	POS	POS	POS	NEG	NEG
2012_263_Z3550 (3355/12)	2012-263		82 years old, MALE	LOWER RESPIRATORY TRACT SPUTUM	POS	POS	POS	POS	POS	POS	POS	POS	POS	POS	POS	POS	POS	POS	NEG	NEG	NEG	NEG	NEG	NEG	NEG	NEG	POS	POS	NEG	POS	POS	POS	POS	NEG	NEG
2012_266_Z3553	2012-266				POS	POS	POS	POS	POS	POS	POS	POS	POS	POS	POS	POS	POS	POS	NEG	NEG	NEG	NEG	NEG	NEG	NEG	NEG	POS	POS	NEG	POS	POS	POS	POS	NEG	NEG
2012_284_Z3571 (4729/12)	2012-284		15 years old, FEMALE	LT AXILLARY ABS SWAB	POS	POS	POS	POS	POS	POS	POS	POS	POS	POS	POS	POS	POS	POS	NEG	NEG	NEG	NEG	NEG	NEG	NEG	NEG	AMB	POS	NEG	POS	POS	POS	POS	NEG	NEG
2012_298_Z3585 (5203/12)	2012-298		20-29 years old, MALE with EPILEPSY	ABSCESSWOUND SWAB	POS	POS	POS	POS	POS	POS	POS	POS	POS	POS	POS	POS	POS	POS	NEG	NEG	NEG	NEG	NEG	NEG	NEG	NEG	AMB	POS	NEG	POS	POS	POS	POS	NEG	NEG
2012_304_Z3591 (5818/12)	2012-304		1 years old, gender not recorded		POS	POS	POS	POS	POS	POS	POS	POS	POS	POS	POS	POS	POS	POS	NEG	NEG	NEG	NEG	NEG	NEG	NEG	NEG	AMB	POS	NEG	POS	POS	POS	POS	NEG	NEG
Z3595_TT308 - {EFC74B77-3925-484F-A4CF-A1F7DC	2012-308		1 month old, gender not recorded	FEVER, INFECTED WOUND WOUND SWAB	POS	POS	POS	POS	POS	POS	POS	POS	POS	POS	POS	POS	POS	POS	NEG	NEG	NEG	NEG	NEG	NEG	NEG	NEG	AMB	POS	NEG	POS	POS	POS	POS	NEG	NEG
2012_369_Z3429 (1112-58547)	2012-369		age unknown, MALE	PUS SWAB	POS	POS	POS	POS	POS	POS	POS	POS	POS	POS	POS	POS	POS	POS	NEG	NEG	NEG	NEG	NEG	NEG	NEG	NEG	AMB	POS	NEG	POS	POS	POS	POS	NEG	NEG
2012_374_Z3434 (1203-13527)	2012-374		age unknown, FEMALE	WOUND SWAB	POS	POS	POS	POS	POS	POS	POS	POS	POS	POS	POS	POS	POS	POS	NEG	NEG	NEG	NEG	NEG	NEG	NEG	NEG	AMB	POS	NEG	POS	POS	POS	POS	NEG	NEG
2013_PA06_Z3989	2013-006		12 years old, FEMALE	SWAB LEFT ELBOW ABSCESS	POS	POS	POS	POS	POS	POS	POS	POS	POS	POS	POS	POS	POS	POS	NEG	NEG	NEG	NEG	NEG	NEG	NEG	NEG	AMB	POS	NEG	POS	POS	POS	POS	NEG	NEG
2013_PA55_Z4038	2013-055		7 years old, MALE	SWAB RIGHT EYE - PERIORBITAL ABSCESS	POS	POS	POS	POS	POS	POS	POS	POS	POS	POS	POS	POS	POS	POS	NEG	NEG	NEG	NEG	NEG	NEG	NEG	NEG	POS	POS	NEG	POS	POS	POS	POS	NEG	NEG
2013_PA71_Z4054	2013-071		59 years old, MALE	SWAB RIGHT 5TH TOE WOUND	POS	POS	POS	POS	POS	POS	POS	POS	POS	POS	POS	POS	POS	POS	NEG	NEG	NEG	NEG	NEG	NEG	NEG	NEG	AMB	POS	NEG	POS	POS	POS	POS	NEG	NEG
ST8-MRSA-IV [PVL+/ACME+], US	ST8-MRSA-IV [PVL+/ACME+], USA300 [truncated/excised SCCmec]				N= 2		%= 0.68																												
2012_180_Z3501 (232/12)	2012-180		age unknown, FEMALE	ABSCESS SWAB	POS	POS	POS	POS	POS	POS	POS	POS	POS	POS	POS	POS	POS	POS	NEG	NEG	NEG	NEG	NEG	NEG	NEG	NEG	AMB	POS	NEG	POS	POS	POS	POS	NEG	NEG
2013_PA68_Z4051	2013-068		17 days old, FEMALE	FLUID RIGHT BREAST ABSCESS	POS	POS	POS	POS	POS	POS	POS	POS	POS	POS	POS	POS	POS	POS	NEG	NEG	NEG	NEG	NEG	NEG	NEG	NEG	AMB	POS	NEG	POS	POS	POS	POS	NEG	NEG
ST72-MSSA	CC8/ST72-MSSA				N= 14		%= 4.76																												
2012_174_Z3451 (102/12)	2012-174		age unknown, FEMALE	URINE CULTURE	POS	POS	POS	POS	POS	POS	POS	POS	POS	POS	POS	POS	POS	POS	NEG	NEG	NEG	NEG	NEG	NEG	NEG	NEG	AMB	POS	NEG	POS	NEG	NEG	NEG	NEG	NEG
2012_223_Z3480 (1506A/12)	2012-223		50 years old, gender not recorded	BLOOD CULTURE	POS	POS	POS	POS	POS	POS	POS	POS	POS	POS	POS	POS	POS	POS	NEG	NEG	NEG	NEG	NEG	NEG	NEG	NEG	POS	POS	NEG	POS	NEG	NEG	NEG	NEG	NEG
2012_236_Z3523 (2086/12)	2012-236		15 years old, FEMALE	RT AXILLARY ABSCESS MISCELLANEOUS	POS	POS	POS	POS	POS	POS	POS	POS	POS	POS	POS	POS	POS	POS	NEG	NEG	NEG	NEG	NEG	NEG	NEG	NEG	AMB	POS	NEG	POS	NEG	NEG	NEG	NEG	NEG
2012_239_Z3525 (2346/12)	2012-239		78 years old, gender not recorded	PLEURAL FLUID	POS	POS	POS	POS	POS	POS	POS	POS	POS	POS	POS	POS	POS	POS	NEG	NEG	NEG	NEG	NEG	NEG	NEG	NEG	AMB	POS	NEG	POS	NEG	NEG	NEG	NEG	NEG
2012_252_Z3535 (3030/12)	2012-252		age unknown, MALE		POS	POS	POS	POS	POS	POS	POS	POS	POS	POS	POS	POS	POS	POS	NEG	NEG	NEG	NEG	NEG	NEG	NEG	NEG	POS	POS	NEG	POS	NEG	NEG	NEG	NEG	NEG
2012_271_Z3558 (3799/12)	2012-271		2 months old, gender not recorded	UMBILICAL SWAB	POS	POS	POS	POS	POS	POS	POS	POS	POS	POS	POS	POS	POS	POS	NEG	NEG	NEG	NEG	NEG	NEG	NEG	NEG	POS	POS	NEG	POS	NEG	NEG	NEG	NEG	NEG
2012_276_Z3563	2012-276				POS	POS	POS	POS	POS	POS	POS	POS	POS	POS	POS	POS	POS	POS	NEG	NEG	NEG	NEG	NEG	NEG	NEG	NEG	POS	POS	NEG	POS	NEG	NEG	NEG	NEG	NEG
2012_300_Z3587 (5638/12)	2012-300		age unknown, FEMALE	SWAB	POS	POS	POS	POS	POS	POS	POS	POS	POS	POS	POS	POS	POS	POS	NEG	NEG	NEG	NEG	NEG	NEG	NEG	NEG	AMB	POS	NEG	POS	NEG	NEG	NEG	NEG	NEG
2012_316_Z3603 (6941/12)	2012-316		70+ years old, FEMALE	SEPTICAEMIAULCER SWAB	POS	POS	POS	POS	POS	POS	POS	POS	POS	POS	POS	POS	POS	POS	NEG	NEG	NEG	NEG	NEG	NEG	NEG	NEG	AMB	POS	NEG	POS	NEG	NEG	NEG	NEG	NEG
2012_319_Z3606 (7019/12)	2012-319		55 years old, MALE with DM	SEPTIC R FOOTBLOOD CULTURE	POS	POS	POS	POS	POS	POS	POS	POS	POS	POS	POS	POS	POS	POS	NEG	NEG	NEG	NEG	NEG	NEG	NEG	NEG	AMB	POS	NEG	POS	NEG	NEG	NEG	NEG	NEG
2012_371_Z3431 (1204-17577)	2012-371		age unknown, gender not recorded	EAR SWAB	POS	POS	POS	POS	POS	POS	POS	POS	POS	POS	POS	POS	POS	POS	NEG	NEG	NEG	NEG	NEG	NEG	NEG	NEG	AMB	POS	NEG	POS	NEG	NEG	NEG	NEG	NEG
2013_PA05_Z3988	2013-005		30 years old, MALE	SWAB LEFT FOOT	POS	POS	POS	POS	POS	POS	POS	POS	POS	POS	POS	POS	POS	POS	NEG	NEG	NEG	NEG	NEG	NEG	NEG	NEG	AMB	POS	NEG	POS	NEG	NEG	NEG	NEG	NEG
2013_PA65_Z4048	2013-065		15 days old, MALE	EYE SWAB	POS	POS	POS	POS	POS	POS	POS	POS	POS	POS	POS	POS	AMB	POS	NEG	NEG	NEG	NEG	NEG	NEG	NEG	NEG	NEG	AMB	NEG	POS	NEG	NEG	NEG	NEG	NEG
2013_PA73_Z4056	2013-073		1 years old, FEMALE	EYE SWAB	POS	POS	POS	POS	POS	POS	POS	POS	POS	POS	POS	POS	POS	POS	NEG	NEG	NEG	NEG	NEG	NEG	NEG	NEG	AMB	POS	NEG	POS	NEG	NEG	NEG	NEG	NEG
ST72-MSSA-SCCfus	CC8/ST72-MSSA-SCCfus				N= 1		%= 0.34																												
2013_PA16_Z3999	2013-016		51 years old, MALE	WOUND SWAB LEFT INFECTED FOOT	POS	POS	POS	POS	POS	POS	POS	POS	POS	POS	POS	POS	POS	POS	NEG	NEG	NEG	NEG	NEG	NEG	NEG	NEG	AMB	POS	NEG	POS	NEG	NEG	NEG	POS	NEG
ST72-MSSA-SCCfus/kdp	CC8/ST72-MSSA-SCCfus/kdp				N= 1		%= 0.34																												
2012_361_Z3421 (1201-1299)	2012-361		age unknown, MALE	RT EYE SWAB	POS	POS	POS	POS	POS	POS	POS	POS	POS	POS	POS	POS	POS	POS	NEG	NEG	NEG	NEG	NEG	NEG	NEG	NEG	AMB	POS	NEG	POS	NEG	NEG	NEG	NEG	NEG
ST239-MRSA-III&SCCmer	CC8/ST239-MRSA-III&SCCmer				N= 25		%= 8.50																												
2012_157_Z3490	2012-157				POS	POS	POS	POS	POS	POS	POS	POS	POS	POS	POS	POS	POS	POS	NEG	NEG	NEG	NEG	NEG	NEG	NEG	NEG	AMB	POS	NEG	POS	POS	POS	POS	NEG	POS
2012_168_Z3494 (27129/11)	2012-168		58 years old, FEMALE	RIGHT BREAST WOUND SWAB	POS	POS	POS	POS	POS	POS	POS	POS	POS	POS	POS	POS	POS	POS	NEG	NEG	NEG	NEG	NEG	NEG	NEG	NEG	AMB	POS	NEG	POS	POS	POS	POS	NEG	POS
2012_195_Z3508 (619/12)	2012-195		70+ years old, MALE with DM/HEART DISEASE	ABSCESSWOUND SWAB	POS	POS	POS	POS	POS	POS	POS	POS	POS	POS	POS	POS	POS	POS	NEG	NEG	NEG	NEG	NEG	NEG	NEG	NEG	AMB	POS	NEG	POS	POS	POS	POS	NEG	NEG
2012_222_Z3479 (1489/12)	2012-222		28 years old, MALE	WOUND SWAB FROM LEFT KNEE	POS	POS	POS	POS	POS	POS	POS	POS	POS	POS	POS	POS	POS	POS	NEG	NEG	NEG	NEG	NEG	NEG	NEG	NEG	AMB	POS	NEG	POS	POS	POS	POS	NEG	AMB
2012_233_Z3540 (1943/12)	2012-233		age unknown, MALE	TRACHEAL SECRETIONS FLUID	POS	POS	POS	POS	POS	POS	POS	POS	POS	POS	POS	POS	POS	POS	NEG	NEG	NEG	NEG	NEG	NEG	NEG	NEG	AMB	POS	NEG	POS	POS	POS	POS	NEG	POS
2012_237_Z3541	2012-237				POS	POS	POS	POS	POS	POS	POS	POS	POS	POS	POS	POS	POS	POS	NEG	NEG	NEG	NEG	NEG	NEG	NEG	NEG	AMB	POS	NEG	POS	POS	POS	POS	NEG	POS
2012_262_Z3549	2012-262				POS	POS	POS	POS	POS	POS	POS	POS	POS	POS	POS	POS	POS	POS	NEG	NEG	NEG	NEG	NEG	NEG	NEG	NEG	POS	POS	NEG	POS	POS	POS	POS	NEG	POS
2012_275_Z3562 (3893/12)	2012-275		age unknown, FEMALE	SWAB	POS	POS	POS	POS	POS	POS	POS	POS	POS	POS	POS	POS	POS	POS	NEG	NEG	NEG	NEG	NEG	NEG	NEG	NEG	POS	POS	NEG	POS	POS	POS	POS	AMB	POS
2012_291_Z3578	2012-291				POS	POS	POS	POS	POS	POS	POS	POS	POS	POS	POS	POS	POS	POS	NEG	NEG	NEG	NEG	NEG	NEG	NEG	NEG	AMB	POS	NEG	POS	POS	POS	POS	NEG	AMB
2012_314_Z3601 (6939/12)	2012-314		70+ years old, MALE with HTN/DM	OSTEOMYELITISSWAB	POS	POS	POS	POS	POS	POS	POS	POS	POS	POS	POS	POS	POS	POS	NEG	NEG	NEG	NEG	NEG	NEG	NEG	NEG	AMB	POS	NEG	POS	POS	POS	POS	NEG	POS
2012323_Z3610	2012-323				POS	POS	POS	POS	POS	POS	POS	POS	POS	POS	POS	POS	POS	POS	NEG	NEG	NEG	NEG	NEG	NEG	NEG	NEG	AMB	POS	NEG	POS	POS	POS	POS	NEG	POS
2012330_Z3617 (7605/12)	2012-330		38 years old, MALE	INTRA OP WOUND SWAB OF LT FOOT WOUND SWAB	POS	POS	POS	POS	POS	POS	POS	POS	POS	POS	POS	POS	POS	POS	NEG	NEG	NEG	NEG	NEG	NEG	NEG	NEG	AMB	POS	NEG	POS	POS	POS	POS	NEG	POS
2012332_Z3619 (8076/12)	2012-332		age unknown, FEMALE	LEFT THUMB INFECTION SWAB	POS	POS	POS	POS	POS	POS	POS	POS	POS	POS	POS	POS	POS	POS	NEG	NEG	NEG	NEG	NEG	NEG	NEG	NEG	AMB	POS	NEG	POS	POS	POS	POS	NEG	AMB
2012339_Z3626 (8558/12)	2012-339		20-29 years old, FEMALE with HTN/DM	CATHETER SWAB	POS	POS	POS	POS	POS	POS	POS	POS	POS	POS	POS	POS	POS	POS	NEG	NEG	NEG	NEG	NEG	NEG	NEG	NEG	AMB	POS	NEG	POS	POS	POS	POS	NEG	AMB
2012340_Z3627 (8523/12)	2012-340		58 years old, MALE	J VAC WOUND SWAB, POST LAP WOUND SWAB	POS	POS	POS	POS	POS	POS	POS	POS	POS	POS	POS	POS	POS	POS	NEG	NEG	NEG	NEG	NEG	NEG	NEG	NEG	AMB	POS	NEG	POS	POS	POS	POS	NEG	AMB
2012348_Z3635	2012-348				POS	POS	POS	POS	POS	POS	POS	POS	POS	POS	POS	POS	POS	POS	NEG	NEG	NEG	NEG	NEG	NEG	NEG	NEG	POS	POS	NEG	POS	POS	POS	POS	NEG	AMB
2012350_Z3637	2012-350				POS	POS	POS	POS	POS	POS	POS	POS	POS	POS	POS	POS	POS	POS	NEG	NEG	NEG	NEG	NEG	NEG	NEG	NEG	AMB	POS	NEG	POS	POS	POS	POS	NEG	AMB
2012_370_Z3430 (1112-52177)	2012-370		age unknown, MALE	NASAL SWAB	POS	POS	POS	POS	POS	POS	POS	POS	POS	POS	POS	POS	POS	POS	NEG	NEG	NEG	NEG	NEG	NEG	NEG	NEG	AMB	POS	NEG	POS	POS	POS	POS	NEG	POS
2012_378_Z3438 (4139)	2012-378		51 years old, FEMALE	WOUND SWAB	POS	POS	POS	POS	POS	POS	POS	POS	POS	POS	POS	POS	POS	POS	NEG	NEG	NEG	NEG	NEG	NEG	NEG	NEG	AMB	POS	NEG	POS	POS	POS	POS	NEG	AMB
2013_PA21_Z4004	2013-021		52 years old, FEMALE	WOUND SWAB UNDER LEFT ARM	POS	POS	POS	POS	POS	POS	POS	POS	POS	POS	POS	POS	POS	POS	NEG	NEG	NEG	NEG	NEG	NEG	NEG	NEG	AMB	POS	NEG	POS	POS	POS	POS	NEG	NEG
2013_PA22_Z4005	2013-022		43 years old, MALE	WOUND SWAB	POS	POS	POS	POS	POS	POS	POS	POS	POS	POS	POS	POS	POS	POS	NEG	NEG	NEG	NEG	NEG	NEG	NEG	NEG	POS	POS	NEG	POS	POS	POS	POS	NEG	POS
2013_PA24_Z4007	2013-024		years old, FEMALE	WOUND SWAB LEFT FOOT	POS	POS	POS	POS	POS	POS	POS	POS	POS	POS	POS	POS	POS	POS	NEG	NEG	NEG	NEG	NEG	NEG	NEG	NEG	NEG	AMB	NEG	POS	POS	POS	POS	NEG	NEG
2013_PA31_Z4014	2013-031		52 years old, FEMALE	WS CHEST WOUND	POS	POS	POS	POS	POS	POS	POS	POS	POS	POS	POS	POS	POS	POS	NEG	NEG	NEG	NEG	NEG	NEG	NEG	NEG	AMB	POS	NEG	POS	POS	POS	POS	NEG	POS
2013_PA35_Z4018	2013-035		23 years old, FEMALE	WS	POS	POS	POS	POS	POS	POS	POS	POS	POS	POS	POS	POS	NEG	POS	NEG	NEG	NEG	NEG	NEG	NEG	NEG	NEG	NEG	NEG	NEG	POS	POS	POS	POS	NEG	NEG
2013_PA81_Z4064	2013-081				POS	POS	POS	POS	POS	POS	POS	POS	POS	POS	POS	POS	POS	POS	NEG	NEG	NEG	NEG	NEG	NEG	NEG	NEG	AMB	POS	NEG	POS	POS	POS	POS	NEG	POS
ST239-MRSA-III+ccrA/B4	CC8/ST239-MRSA-III+ccrA/B4				N= 2		%= 0.68																												
2012_254_Z3537 (3124A/12)	2012-254		age unknown, gender not recorded	BLOOD CULTURE	POS	POS	POS	POS	POS	POS	POS	POS	POS	POS	POS	POS	POS	POS	NEG	NEG	NEG	NEG	NEG	NEG	NEG	NEG	AMB	POS	NEG	POS	POS	POS	POS	NEG	AMB
2013_PA25_Z4008	2013-025		20 years old, MALE	WOUND SWAB	POS	POS	POS	POS	POS	POS	POS	POS	POS	POS	POS	POS	POS	POS	NEG	NEG	NEG	NEG	NEG	NEG	NEG	NEG	AMB	POS	NEG	POS	POS	POS	POS	NEG	AMB
ST834-MSSA	CC9/ST834-MSSA				N= 1		%= 0.34																												
2012_268_Z3555 (3693/12)	2012-268		76 years old, FEMALE	ABSCESS SWAB	POS	POS	POS	POS	POS	POS	POS	POS	POS	POS	POS	POS	POS	POS	NEG	NEG	NEG	NEG	NEG	NEG	NEG	NEG	POS	POS	NEG	POS	NEG	NEG	NEG	NEG	NEG
CC12-MSSA	CC12-MSSA				N= 3		%= 1.02																												
2012_210_Z3470 (1177/12)	2012-210		41 years old, MALE	RT FOREARM ABSCESS	POS	POS	POS	POS	POS	POS	POS	POS	POS	POS	NEG	NEG	NEG	NEG	POS	POS	POS	POS	NEG	NEG	NEG	NEG	NEG	NEG	NEG	POS	NEG	NEG	NEG	NEG	NEG
2012_230_Z3487 (1797/12)	2012-230		age unknown, MALE	TRACHEAL SECRETIONS ASPIRATE	POS	POS	POS	POS	POS	POS	POS	POS	POS	POS	NEG	NEG	NEG	NEG	POS	POS	POS	POS	NEG	NEG	NEG	NEG	NEG	NEG	NEG	POS	NEG	NEG	NEG	NEG	NEG
2013_PA18_Z4001	2013-018		years old, MALE	SEPSIS	POS	POS	POS	POS	POS	POS	POS	POS	POS	POS	NEG	NEG	NEG	NEG	POS	POS	POS	POS	NEG	NEG	NEG	NEG	NEG	NEG	NEG	POS	NEG	NEG	NEG	NEG	NEG
CC15-MSSA	CC15-MSSA				N= 10		%= 3.40																												
2012_160_Z3444 (26386/11)	2012-160		age unknown, MALE	SWAB	POS	POS	POS	POS	POS	POS	POS	POS	POS	POS	NEG	NEG	NEG	NEG	POS	POS	POS	POS	NEG	NEG	NEG	NEG	NEG	NEG	NEG	POS	NEG	NEG	NEG	NEG	NEG
2012_167_Z3514 (27010/11)	2012-167		age unknown, MALE	SWAB	POS	POS	POS	POS	POS	POS	POS	POS	POS	POS	NEG	NEG	NEG	NEG	POS	POS	POS	POS	NEG	NEG	NEG	NEG	NEG	NEG	NEG	POS	NEG	NEG	NEG	NEG	NEG
2012_184_Z3455 (376/12)	2012-184		age unknown, MALE	SWAB	POS	POS	POS	POS	POS	POS	POS	POS	POS	POS	NEG	NEG	NEG	NEG	POS	POS	POS	POS	NEG	NEG	NEG	NEG	NEG	NEG	NEG	POS	NEG	NEG	NEG	NEG	NEG
2012_205_Z3465 (939/12)	2012-205		age unknown, MALE	SWAB	POS	POS	POS	POS	POS	POS	POS	POS	POS	POS	NEG	NEG	NEG	NEG	POS	POS	POS	POS	NEG	NEG	NEG	NEG	NEG	NEG	NEG	POS	NEG	NEG	NEG	NEG	NEG
2012_231_Z3519 (1831/12)	2012-231		7 years old, MALE	OTITIS MEDIAEAR SWAB	POS	POS	POS	POS	POS	POS	POS	POS	POS	POS	NEG	NEG	NEG	NEG	POS	POS	POS	POS	NEG	NEG	NEG	NEG	NEG	NEG	NEG	POS	NEG	NEG	NEG	NEG	NEG
2012_249_Z3533 (2907/12)	2012-249		58 years old, FEMALE	WOUND SWAB	POS	POS	POS	POS	POS	POS	POS	POS	POS	POS	NEG	NEG	NEG	NEG	POS	POS	POS	POS	NEG	NEG	NEG	NEG	NEG	NEG	NEG	POS	NEG	NEG	NEG	NEG	NEG
2012_305_Z3592 (5839/12)	2012-305		age unknown, MALE	SWAB	POS	POS	POS	POS	POS	POS	POS	POS	POS	POS	NEG	NEG	NEG	NEG	POS	POS	POS	POS	NEG	NEG	NEG	NEG	NEG	NEG	NEG	POS	NEG	NEG	AMB	NEG	NEG
2012_313_Z3600 (6857/12)	2012-313		60-69 years old, FEMALE with HTN/EPILEPSY/DM/G	ULCER SWAB	POS	POS	POS	POS	POS	POS	POS	POS	POS	POS	NEG	NEG	NEG	NEG	POS	POS	POS	POS	NEG	NEG	NEG	NEG	NEG	NEG	NEG	POS	NEG	NEG	NEG	NEG	NEG
2012_345_Z3632 (7966B/12)	2012-345		age unknown, MALE	VEIN BLOOD CULTURE	POS	POS	POS	POS	POS	POS	POS	POS	POS	POS	NEG	NEG	NEG	NEG	POS	POS	POS	POS	NEG	NEG	NEG	NEG	NEG	NEG	NEG	POS	NEG	NEG	NEG	NEG	NEG
2013_PA33_Z4016	2013-033		37 years old, MALE	WS RIGHT LEG	POS	POS	POS	POS	POS	POS	POS	POS	POS	POS	NEG	NEG	NEG	NEG	POS	POS	POS	POS	NEG	NEG	NEG	NEG	NEG	NEG	NEG	POS	NEG	NEG	NEG	NEG	NEG
CC20-MSSA	CC20-MSSA				N= 1		%= 0.34																												
2012_240_Z3526 (2354/12)	2012-240		age unknown, FEMALE	EYE DISCHARGE SWAB	POS	POS	POS	POS	POS	POS	POS	POS	POS	POS	POS	POS	POS	POS	NEG	NEG	NEG	NEG	NEG	NEG	NEG	NEG	AMB	POS	NEG	POS	NEG	NEG	NEG	NEG	NEG
CC30-MSSA	CC30-MSSA				N= 1		%= 0.34																												
2012_155_Z3488 (25905A/11)	2012-155		20-29 years old, FEMALE with KIDNEY/RECURRENT	UTIBLOOD CULTURE	POS	POS	POS	POS	POS	POS	POS	POS	POS	POS	NEG	NEG	NEG	NEG	NEG	NEG	NEG	NEG	POS	POS	POS	POS	NEG	NEG	NEG	POS	NEG	NEG	NEG	NEG	NEG
CC30-MSSA [PVL+]	CC30-MSSA [PVL+]				N= 26		%= 8.84																												
2012_164_Z3447 (26574/11)	2012-164		age unknown, MALE	ABSCESS SWAB	POS	POS	POS	POS	POS	POS	POS	POS	POS	POS	NEG	NEG	NEG	NEG	NEG	NEG	NEG	NEG	POS	POS	POS	POS	NEG	NEG	NEG	POS	NEG	NEG	NEG	NEG	NEG
2012_166_Z3493 (26988A/11)	2012-166		age unknown, MALE	BLOOD CULTURE	POS	POS	POS	POS	POS	POS	POS	POS	POS	POS	NEG	NEG	NEG	NEG	NEG	NEG	NEG	NEG	POS	POS	POS	POS	NEG	NEG	NEG	POS	NEG	NEG	NEG	NEG	NEG
2012_178_Z3500 (197/12)	2012-178		age unknown, gender not recorded	BLOOD CULTURE	POS	POS	POS	POS	POS	POS	POS	POS	POS	POS	NEG	NEG	NEG	NEG	NEG	NEG	NEG	NEG	POS	POS	POS	POS	NEG	NEG	NEG	POS	NEG	NEG	NEG	NEG	NEG
2012_183_Z3454	2012-183				POS	POS	POS	POS	POS	POS	POS	POS	POS	POS	NEG	NEG	NEG	NEG	NEG	NEG	NEG	NEG	POS	POS	POS	POS	NEG	NEG	NEG	POS	NEG	NEG	NEG	NEG	NEG
2012_186_Z3503 (444/12)	2012-186		23 years old, MALE	ABSCESS SWAB	POS	POS	POS	POS	POS	POS	POS	POS	POS	POS	NEG	NEG	NEG	NEG	NEG	NEG	NEG	NEG	POS	POS	POS	POS	NEG	NEG	NEG	POS	NEG	NEG	NEG	NEG	NEG
2012_189_Z3506 (542/12)	2012-189		27 years old, FEMALE	WOUND SWAB	POS	POS	POS	POS	POS	POS	POS	POS	POS	POS	NEG	NEG	NEG	NEG	NEG	NEG	NEG	NEG	POS	POS	POS	POS	NEG	NEG	NEG	POS	NEG	NEG	NEG	NEG	NEG
2012_192_Z3457 (606/12)	2012-192		2 years old, FEMALE	ABSCESS RT SIDE OF NECK	POS	POS	POS	POS	POS	POS	POS	POS	POS	POS	NEG	NEG	NEG	NEG	NEG	NEG	NEG	NEG	POS	POS	POS	POS	NEG	NEG	NEG	POS	NEG	NEG	NEG	NEG	NEG
2012_196_Z3509 (707/12)	2012-196		age unknown, MALE	ABSCESS SWAB	POS	POS	POS	POS	POS	POS	POS	POS	POS	POS	NEG	NEG	NEG	NEG	NEG	NEG	NEG	NEG	POS	POS	POS	POS	NEG	NEG	NEG	POS	NEG	NEG	NEG	NEG	NEG
2012_218_Z3476 (1432/12)	2012-218		39 years old, MALE	ABSCESS SWAB	POS	POS	POS	POS	POS	POS	POS	POS	POS	POS	NEG	NEG	NEG	NEG	NEG	NEG	NEG	NEG	POS	POS	POS	POS	NEG	NEG	NEG	POS	NEG	NEG	NEG	NEG	NEG
2012_232_Z3520 (1892/12)	2012-232		age unknown, FEMALE	EYE DISCHARGE SWAB	POS	POS	POS	POS	POS	POS	POS	POS	POS	POS	NEG	NEG	NEG	NEG	NEG	NEG	NEG	NEG	POS	POS	POS	POS	NEG	NEG	NEG	POS	NEG	NEG	NEG	NEG	NEG
2012_278_Z3565 (4397/12)	2012-278		8 years old, MALE	FURUNCULOSISGLUTEAL ABS SWAB	POS	POS	POS	POS	POS	POS	POS	POS	POS	POS	NEG	NEG	NEG	NEG	NEG	NEG	NEG	NEG	POS	POS	POS	POS	NEG	NEG	NEG	POS	NEG	NEG	NEG	NEG	NEG
2012_285_Z3572 (4802/12)	2012-285		age unknown, FEMALE	EAR SWAB	POS	POS	POS	POS	POS	POS	POS	POS	POS	POS	NEG	NEG	NEG	NEG	NEG	NEG	NEG	NEG	POS	POS	POS	POS	NEG	NEG	NEG	POS	NEG	NEG	NEG	NEG	NEG
2012_287_Z3574 (4906/12)	2012-287		5 years old, MALE	LT GLUTEAL ABS SWAB	POS	POS	POS	POS	POS	POS	POS	POS	POS	POS	NEG	NEG	NEG	NEG	NEG	NEG	NEG	NEG	POS	POS	POS	POS	NEG	NEG	NEG	POS	NEG	NEG	NEG	NEG	NEG
2012_295_Z3582 (5116/12)	2012-295		9 years old, MALE	SWAB	POS	POS	POS	POS	POS	POS	POS	POS	POS	POS	NEG	NEG	NEG	NEG	NEG	NEG	NEG	NEG	POS	POS	POS	POS	NEG	NEG	NEG	POS	NEG	NEG	NEG	NEG	NEG
2012_326_Z3613 (7321/12)	2012-326		4 months old, gender not recorded	RT BREAST ABSCESS ABSCESS SWAB	POS	POS	POS	POS	POS	POS	POS	POS	POS	POS	NEG	NEG	NEG	NEG	NEG	NEG	NEG	NEG	POS	POS	POS	POS	NEG	NEG	NEG	POS	NEG	NEG	NEG	NEG	NEG
2012_356_Z3643 (1204-15897)	2012-356		age unknown, MALE	WOUND SWAB	POS	POS	POS	POS	POS	POS	POS	POS	POS	POS	NEG	NEG	NEG	NEG	NEG	NEG	NEG	NEG	POS	POS	POS	POS	NEG	NEG	NEG	POS	NEG	NEG	NEG	NEG	NEG
2012_366_Z3426 (1204-16124)	2012-366		age unknown, MALE	WOUND SWAB	POS	POS	POS	POS	POS	POS	POS	POS	POS	POS	NEG	NEG	NEG	NEG	NEG	NEG	NEG	NEG	POS	POS	POS	POS	NEG	NEG	NEG	POS	NEG	NEG	NEG	NEG	NEG
2012_372_Z3432 (1205-19821)	2012-372		age unknown, FEMALE	SKIN SWAB	POS	POS	POS	POS	POS	POS	POS	POS	POS	POS	NEG	NEG	NEG	NEG	NEG	NEG	NEG	NEG	POS	POS	POS	POS	NEG	NEG	NEG	POS	NEG	NEG	NEG	NEG	NEG
2012_380_Z3440 (4141)	2012-380		3 years old, MALE with BURNS	ABSCESSWOUND SWAB	POS	POS	POS	POS	POS	POS	POS	POS	POS	POS	NEG	NEG	NEG	NEG	NEG	NEG	NEG	NEG	POS	POS	POS	POS	NEG	NEG	NEG	POS	NEG	NEG	NEG	NEG	NEG
2013_PA20_Z4003	2013-020		20 MONTHS old, MALE	WS NECK ABSCESS	POS	POS	POS	POS	POS	POS	POS	POS	POS	POS	NEG	NEG	NEG	NEG	NEG	NEG	NEG	NEG	POS	POS	POS	POS	NEG	NEG	NEG	POS	NEG	NEG	NEG	NEG	NEG
2013_PA36_Z4019	2013-036		56 years old, FEMALE	WS	POS	POS	POS	POS	POS	POS	POS	POS	POS	POS	NEG	NEG	NEG	NEG	NEG	NEG	NEG	NEG	POS	POS	AMB	POS	NEG	NEG	NEG	POS	NEG	NEG	NEG	NEG	NEG
2013_PA46_Z4029	2013-046		1 years old, FEMALE	SWAB SCALP INFECTED INSECT BITE	POS	POS	POS	POS	POS	POS	POS	POS	POS	POS	NEG	NEG	NEG	NEG	NEG	NEG	NEG	NEG	POS	POS	AMB	POS	NEG	NEG	NEG	POS	NEG	NEG	NEG	NEG	NEG
2013_PA50_Z4033	2013-050		7 months old, FEMALE	WS RECURRENT ABSCESS	POS	POS	POS	POS	POS	POS	POS	POS	POS	POS	NEG	NEG	NEG	NEG	NEG	NEG	NEG	NEG	POS	POS	AMB	POS	NEG	NEG	NEG	POS	NEG	NEG	NEG	NEG	NEG
2013_PA63_Z4046	2013-063		1 week old, FEMALE	SWAB LEFT PERI AREOLA ABSCESS	POS	POS	POS	POS	POS	POS	POS	POS	POS	POS	NEG	NEG	NEG	NEG	NEG	NEG	NEG	NEG	POS	POS	POS	POS	NEG	NEG	NEG	POS	NEG	NEG	NEG	NEG	NEG
2013_PA78_Z4061	2013-078				POS	POS	POS	POS	POS	POS	POS	POS	POS	POS	NEG	NEG	NEG	NEG	NEG	NEG	NEG	NEG	POS	POS	POS	POS	NEG	NEG	NEG	POS	NEG	NEG	NEG	NEG	NEG
2013_PA79_Z4062	2013-079				POS	POS	POS	POS	POS	POS	POS	POS	POS	POS	NEG	NEG	NEG	NEG	NEG	NEG	NEG	NEG	POS	POS	POS	POS	NEG	NEG	NEG	POS	NEG	NEG	NEG	NEG	NEG
CC45-MSSA	CC45-MSSA				N= 10		%= 3.40																												
2012_172_Z3449 (49/12)	2012-172		3 months old, FEMALE	WOUND SWAB FROM SCALP	POS	POS	POS	POS	POS	POS	POS	POS	POS	POS	POS	POS	POS	POS	NEG	NEG	NEG	NEG	NEG	AMB	NEG	NEG	NEG	NEG	NEG	POS	NEG	NEG	NEG	NEG	NEG
2012_208_Z3468 (1011/12)	2012-208		age unknown, gender not recorded	GASTRONOMY SITE SWAB	POS	POS	POS	POS	POS	POS	POS	POS	POS	POS	POS	POS	POS	POS	NEG	NEG	NEG	NEG	NEG	NEG	NEG	NEG	NEG	NEG	NEG	POS	NEG	NEG	NEG	NEG	NEG
2012_235_Z3522 (2085/12)	2012-235		60-69 years old, FEMALE with CATARACT	ABSCESSLEFT ELBOW ABSCESS MISCELLANEOUS	POS	POS	POS	POS	POS	POS	POS	POS	POS	POS	POS	POS	POS	POS	NEG	NEG	NEG	NEG	NEG	AMB	NEG	NEG	NEG	NEG	NEG	POS	NEG	NEG	NEG	NEG	NEG
2012_290_Z3577 (4921/12)	2012-290		age unknown, gender not recorded	URINE CULTURE	POS	POS	POS	POS	POS	POS	POS	POS	POS	POS	POS	POS	POS	POS	NEG	NEG	NEG	NEG	NEG	NEG	NEG	NEG	NEG	NEG	NEG	POS	NEG	NEG	NEG	NEG	NEG
2012_311_Z3598 (6182/12)	2012-311		2 weeks old, gender not recorded	UMBILICAL SWAB	POS	POS	POS	POS	POS	POS	POS	POS	POS	POS	POS	POS	POS	POS	NEG	NEG	NEG	NEG	NEG	NEG	NEG	NEG	NEG	NEG	NEG	POS	NEG	NEG	NEG	NEG	NEG
Z3604_TT317 - {DC558C18-2959-4049-B8F3-507365	2012-317		age unknown, gender not recorded	SWAB	POS	POS	POS	POS	POS	POS	POS	POS	POS	POS	POS	POS	POS	POS	NEG	NEG	NEG	NEG	NEG	NEG	NEG	NEG	NEG	NEG	NEG	POS	NEG	NEG	NEG	NEG	NEG
2012_363_Z3423 (1112-61058)	2012-363		age unknown, MALE	URINE	POS	POS	POS	POS	POS	POS	POS	POS	POS	POS	POS	POS	POS	POS	NEG	NEG	NEG	NEG	NEG	AMB	NEG	NEG	NEG	NEG	NEG	POS	NEG	NEG	NEG	NEG	NEG
2013_PA19_Z4002	2013-019		years old, FEMALE	SWAB LEFT EAR ABSCESS	POS	POS	POS	POS	POS	POS	POS	POS	POS	POS	POS	POS	POS	POS	NEG	NEG	NEG	NEG	NEG	NEG	NEG	NEG	NEG	NEG	NEG	POS	NEG	NEG	NEG	NEG	NEG
2013_PA54_Z4037	2013-054		1 years old, MALE	SWAB RIGHT WRIST, Post mortem	POS	POS	POS	POS	POS	POS	POS	POS	POS	POS	POS	POS	POS	POS	NEG	NEG	NEG	NEG	NEG	NEG	NEG	NEG	NEG	NEG	NEG	POS	NEG	NEG	NEG	NEG	NEG
2013_PA82_Z4065	2013-082				POS	POS	POS	POS	POS	POS	POS	POS	POS	POS	POS	POS	POS	POS	NEG	NEG	NEG	NEG	NEG	NEG	NEG	NEG	NEG	NEG	NEG	POS	NEG	NEG	NEG	NEG	NEG
CC97-MSSA	CC97-MSSA				N= 5		%= 1.70																												
2012_182_Z3453 (279A/12)	2012-182		47 years old, FEMALE with DM/SCHIZOPHRENIA	SEPSISBLOOD CULTURE	POS	POS	POS	POS	POS	POS	POS	POS	POS	POS	POS	POS	POS	POS	NEG	NEG	NEG	NEG	NEG	NEG	NEG	NEG	AMB	POS	NEG	POS	NEG	NEG	NEG	NEG	NEG
2012_187_Z3504	2012-187				POS	POS	POS	POS	POS	POS	POS	POS	POS	POS	POS	POS	POS	POS	NEG	NEG	NEG	NEG	NEG	NEG	NEG	NEG	AMB	POS	NEG	POS	NEG	NEG	NEG	NEG	NEG
2012_197_Z3510 (757/12)	2012-197		age unknown, MALE	URINE CULTURE	POS	POS	POS	POS	POS	POS	POS	POS	POS	POS	POS	POS	POS	POS	NEG	NEG	NEG	NEG	NEG	NEG	NEG	NEG	POS	POS	NEG	POS	NEG	NEG	NEG	NEG	NEG
2012_199_Z3516 (785/12)	2012-199		age unknown, MALE	SWAB	POS	POS	POS	POS	POS	POS	POS	POS	POS	POS	POS	POS	POS	POS	NEG	NEG	NEG	NEG	NEG	NEG	NEG	NEG	AMB	POS	NEG	POS	NEG	NEG	NEG	NEG	NEG
2013_PA15_Z3998	2013-015		56 years old, FEMALE	WS RECTAL ABSCESS	POS	POS	POS	POS	POS	POS	POS	POS	POS	POS	POS	POS	POS	POS	NEG	NEG	NEG	NEG	NEG	NEG	NEG	NEG	AMB	POS	NEG	POS	NEG	NEG	NEG	NEG	NEG
CC101-MSSA	CC101-MSSA				N= 3		%= 1.02																												
2012_283_Z3570 (4725/12)	2012-283		age unknown, gender not recorded	WOUND SWAB	POS	POS	POS	POS	POS	POS	POS	POS	POS	POS	POS	POS	POS	POS	NEG	NEG	NEG	NEG	NEG	NEG	NEG	NEG	AMB	POS	NEG	POS	NEG	NEG	NEG	NEG	NEG
2012_247_Z3531 (2788/12)	2012-247		30 years old, MALE	POSTULES ON BACK OF NECK WOUND SWAB	POS	POS	POS	POS	POS	POS	POS	POS	POS	POS	POS	POS	POS	POS	NEG	NEG	NEG	NEG	NEG	NEG	NEG	NEG	AMB	POS	NEG	POS	NEG	NEG	NEG	NEG	NEG
2012_279_Z3566 (4506/12)	2012-279		18 years old, FEMALE	HIGH VAGINAL SWAB	POS	POS	POS	POS	POS	POS	POS	POS	POS	POS	POS	POS	POS	POS	NEG	NEG	NEG	NEG	NEG	NEG	NEG	NEG	POS	POS	NEG	POS	NEG	NEG	NEG	NEG	NEG

Strain / Isolates		MLST profile	Clinical / demographic data		Domain 1 of 23S-rRNA	glyceraldehyde 3-phosphate dehydrogenase locus 1	katalase A	coagulase	thermostable extracellular nuclease	staphylococcal protein A	IgG-binding protein	staphylococcal accessory regulator A	histidine protein kinase, sae locus	sensor protein	accessory gene regulator allele I				accessory gene regulator allele II				accessory gene regulator allele III				accessory gene regulator allele IV			haemolysin delta	alternate penicillin binding protein 2, defining MRSA	truncated signal transducer protein MecR1	glycerophosphoryl diester phosphodiesterase, associated with mecA	cassette chromosome recombinase genes A/B-1	
			Patient	Sample type, Diagnosis	rrnD1 (S. aureus)	gapA	katA	coA	nuc1	spa	sbi	sarA	saeS	vraS	agrI (total)	agrB-I	agrC-I	agrD-I	agrII (total)	agrB-II	agrC-II	agrD-II	agrIII (total)	agrB-III	agrC-III	agrD-III	agrIV (total)	agrB-IV	agrC-IV	hld	mecA	delta_mecR	ugpQ	ccrA-1	ccrB-1
CC121-MSSA	CC121-MSSA				N= 12		%= 4.08																												
2012_163_Z3446 (26495/11)	2012-163		age unknown, FEMALE	TRACHEOSTOMY FLUID	POS	POS	POS	POS	POS	POS	POS	POS	POS	POS	NEG	POS	POS	NEG	NEG	NEG	NEG	NEG	NEG	NEG	NEG	NEG	POS	POS	POS	POS	NEG	NEG	NEG	NEG	NEG
2012_204_Z3464 (938/12)	2012-204		age unknown, gender not recorded	EYE DISCHARGE SWAB	POS	POS	POS	POS	POS	POS	POS	POS	POS	POS	NEG	POS	POS	NEG	NEG	NEG	NEG	NEG	NEG	NEG	NEG	NEG	POS	POS	POS	POS	NEG	NEG	NEG	NEG	NEG
2012_225_Z3482 (1563/12)	2012-225		age unknown, FEMALE	SCALP SWAB	POS	POS	POS	POS	POS	POS	POS	POS	POS	POS	NEG	POS	POS	NEG	NEG	NEG	NEG	NEG	NEG	NEG	NEG	NEG	POS	POS	POS	POS	NEG	NEG	NEG	NEG	NEG
2012_228_Z3485 (1700B/12)	2012-228		58 years old, MALE with HEART DISEASE	BLOOD CULTURE	POS	POS	POS	POS	POS	POS	POS	POS	POS	POS	NEG	POS	POS	NEG	NEG	NEG	NEG	NEG	NEG	NEG	NEG	NEG	POS	POS	POS	POS	NEG	NEG	NEG	NEG	NEG
2012_229_Z3486 (1737/12)	2012-229		13 years old, FEMALE	RT BREAST DISCHARGE SWAB	POS	POS	POS	POS	POS	POS	POS	POS	POS	POS	NEG	POS	POS	NEG	NEG	NEG	NEG	NEG	NEG	NEG	NEG	NEG	POS	POS	POS	POS	NEG	NEG	NEG	NEG	NEG
2012_302_Z3589 (5795/12)	2012-302		50-59 years old, FEMALE	CHRONIC COUGH, BRONCHOSCOPY - INFECTIVE PROCE	POS	POS	POS	POS	POS	POS	POS	POS	POS	POS	NEG	AMB	POS	NEG	NEG	NEG	NEG	NEG	NEG	NEG	NEG	NEG	POS	POS	POS	POS	NEG	NEG	NEG	NEG	NEG
2012_359_Z3419	2012-359				POS	POS	POS	POS	POS	POS	POS	POS	POS	POS	NEG	AMB	POS	NEG	NEG	NEG	NEG	NEG	NEG	NEG	NEG	NEG	POS	POS	POS	POS	NEG	NEG	NEG	NEG	NEG
2012_362_Z3422 (1201-996)	2012-362		age unknown, FEMALE	SKIN SWAB	POS	POS	POS	POS	POS	POS	POS	POS	POS	POS	NEG	POS	POS	NEG	NEG	NEG	NEG	NEG	NEG	NEG	NEG	NEG	POS	POS	POS	POS	NEG	NEG	NEG	NEG	NEG
2012_367_Z3427 (1204-16413)	2012-367		age unknown, FEMALE	MOUTHSWAB	POS	POS	POS	POS	POS	POS	POS	POS	POS	POS	AMB	POS	POS	NEG	NEG	NEG	NEG	NEG	NEG	NEG	NEG	NEG	POS	POS	POS	POS	NEG	NEG	NEG	NEG	NEG
2013_PA09_Z3992	2013-009		1 years old, MALE	EYE SWAB, BLOOD	POS	POS	POS	POS	POS	POS	POS	POS	POS	POS	NEG	POS	POS	NEG	NEG	NEG	NEG	NEG	NEG	NEG	NEG	NEG	POS	POS	POS	POS	NEG	NEG	NEG	NEG	NEG
2013_PA59_Z4042	2013-059		AW 1 years old, MALE	SWAB AXILLARY ABSCESS	POS	POS	POS	POS	POS	POS	POS	POS	POS	POS	NEG	POS	POS	NEG	NEG	NEG	NEG	NEG	NEG	NEG	NEG	NEG	POS	POS	POS	POS	NEG	NEG	NEG	NEG	NEG
2013_PA60_Z4043	2013-060		3 years old, FEMALE	SWAB, Post mortem	POS	POS	POS	POS	POS	POS	POS	POS	POS	POS	NEG	NEG	POS	NEG	NEG	NEG	NEG	NEG	NEG	NEG	NEG	NEG	POS	POS	POS	POS	NEG	NEG	NEG	NEG	NEG
CC152-MSSA [PVL+]	CC152-MSSA [PVL+]				N= 28		%= 9.52																												
2012_190_Z3507 (549/12)	2012-190		54 years old, MALE	RT FT ABSCESS SWAB	POS	POS	POS	POS	POS	POS	POS	POS	POS	POS	POS	POS	POS	POS	NEG	NEG	NEG	NEG	NEG	NEG	NEG	NEG	AMB	POS	NEG	POS	NEG	NEG	NEG	NEG	NEG
2012_201_Z3461 (823/12)	2012-201		age unknown, MALE	WOUND SWAB	POS	POS	POS	POS	POS	POS	POS	POS	POS	POS	POS	POS	POS	POS	NEG	NEG	NEG	NEG	NEG	NEG	NEG	NEG	POS	POS	NEG	POS	NEG	NEG	NEG	NEG	NEG
2012_209_Z3469 (1116/12)	2012-209		11 years old, MALE	BLOOD CULTURE	POS	POS	POS	POS	POS	POS	POS	POS	POS	POS	POS	POS	POS	POS	NEG	NEG	NEG	NEG	NEG	NEG	NEG	NEG	POS	POS	NEG	POS	NEG	NEG	NEG	NEG	NEG
2012_211_Z3517 (1238/12)	2012-211		4 years old, gender not recorded	ABSCESS ON LEFT THUMB WOUND SWAB	POS	POS	POS	POS	POS	POS	AMB	POS	POS	POS	POS	POS	POS	POS	NEG	NEG	NEG	NEG	NEG	NEG	NEG	NEG	AMB	POS	NEG	POS	NEG	NEG	NEG	NEG	NEG
2012_213_Z3472 (1320/12)	2012-213		6 years old, MALE	SWAB	POS	POS	POS	POS	POS	POS	POS	POS	POS	POS	POS	POS	POS	POS	NEG	NEG	NEG	NEG	NEG	NEG	NEG	NEG	POS	POS	NEG	POS	NEG	NEG	NEG	NEG	NEG
2012_221_Z3478	2012-221				POS	POS	POS	POS	POS	POS	POS	POS	POS	POS	POS	POS	POS	POS	NEG	NEG	NEG	NEG	NEG	NEG	NEG	NEG	POS	POS	NEG	POS	NEG	NEG	NEG	NEG	NEG
2012_234_Z3521 (1981/12)	2012-234		age unknown, FEMALE	SKIN SWAB	POS	POS	POS	POS	POS	POS	POS	POS	POS	POS	POS	POS	POS	POS	NEG	NEG	NEG	NEG	NEG	NEG	NEG	NEG	POS	POS	NEG	POS	NEG	NEG	NEG	NEG	NEG
2012_238_Z3524 (2199/12)	2012-238		age unknown, MALE	SPUTUM	POS	POS	POS	POS	POS	POS	POS	POS	POS	POS	POS	POS	POS	POS	NEG	NEG	NEG	NEG	NEG	NEG	NEG	NEG	AMB	POS	NEG	POS	NEG	NEG	NEG	NEG	NEG
2012_260_Z3547 (3290/12)	2012-260		22 years old, MALE	AXILLARY ABS SWAB	POS	POS	POS	POS	POS	POS	POS	POS	POS	POS	POS	POS	POS	POS	NEG	NEG	NEG	NEG	NEG	NEG	NEG	NEG	POS	POS	NEG	POS	AMB	NEG	NEG	NEG	NEG
2012_272_Z3559 (3802/12)	2012-272		age unknown, MALE	R PALM ABS SWAB	POS	POS	POS	POS	POS	POS	POS	POS	POS	POS	POS	POS	POS	POS	NEG	NEG	NEG	NEG	NEG	NEG	NEG	NEG	POS	POS	NEG	POS	NEG	NEG	NEG	NEG	NEG
2012_289_Z3576 (4912/12)	2012-289		age unknown, MALE	RT FOOT ABS SWAB	POS	POS	POS	POS	POS	POS	POS	POS	POS	POS	POS	POS	POS	POS	NEG	NEG	NEG	NEG	NEG	NEG	NEG	NEG	AMB	POS	NEG	POS	NEG	NEG	NEG	NEG	NEG
2012_309_Z3596	2012-309				POS	POS	POS	POS	POS	POS	POS	POS	POS	POS	POS	POS	POS	POS	NEG	NEG	NEG	NEG	NEG	NEG	NEG	NEG	AMB	POS	NEG	POS	NEG	NEG	NEG	NEG	NEG
2012_322_Z3609 (7178/12)	2012-322		age unknown, MALE	SWAB	POS	POS	POS	POS	POS	POS	POS	POS	POS	POS	POS	POS	POS	POS	NEG	NEG	NEG	NEG	NEG	NEG	NEG	NEG	AMB	POS	NEG	POS	NEG	NEG	NEG	NEG	NEG
2012_325_Z3612 (7254/12)	2012-325		14 years old, FEMALE	URINE CULTURE	POS	POS	POS	POS	POS	POS	POS	POS	POS	POS	POS	POS	POS	POS	NEG	NEG	NEG	NEG	NEG	NEG	NEG	NEG	AMB	POS	NEG	POS	NEG	NEG	NEG	NEG	NEG
2012_328_Z3615 (7456/12)	2012-328		34 years old, MALE	RT HAND INFECTED WOUND SWAB	POS	POS	POS	POS	POS	POS	POS	POS	POS	POS	POS	POS	POS	POS	NEG	NEG	NEG	NEG	NEG	NEG	NEG	NEG	AMB	POS	NEG	POS	NEG	NEG	NEG	NEG	NEG
2012_334_Z3621 (8267/12)	2012-334		age unknown, MALE	ABSCESS SWAB	POS	POS	POS	POS	POS	POS	POS	POS	POS	POS	POS	POS	POS	POS	NEG	NEG	NEG	NEG	NEG	NEG	NEG	NEG	AMB	POS	NEG	POS	NEG	NEG	NEG	NEG	NEG
2012_360_Z3420 (1201-4047)	2012-360		age unknown, FEMALE	PUS SWAB	POS	POS	POS	POS	POS	POS	POS	POS	POS	POS	POS	POS	POS	POS	NEG	NEG	NEG	NEG	NEG	NEG	NEG	NEG	AMB	POS	NEG	POS	AMB	NEG	NEG	NEG	NEG
2012_382_Z3442 (4086)	2012-382		59y ears old, MALE	WOUND SWAB	POS	POS	POS	POS	POS	POS	POS	POS	POS	POS	POS	POS	POS	POS	NEG	NEG	NEG	NEG	NEG	NEG	NEG	NEG	POS	POS	NEG	POS	NEG	NEG	NEG	NEG	NEG
2013_PA02_Z3985	2013-002		56 years old, FEMALE	WOUND SWAB, FACIAL ABSCESS	POS	POS	POS	POS	POS	POS	POS	POS	POS	POS	POS	POS	POS	POS	NEG	NEG	NEG	NEG	NEG	NEG	NEG	NEG	POS	POS	NEG	POS	NEG	NEG	NEG	NEG	NEG
2013_PA03_Z3986	2013-003		1.5 years old, MALE	WOUND SWAB RIGHT KNEE ABSCESS	POS	POS	POS	POS	POS	POS	AMB	POS	POS	POS	POS	POS	POS	POS	NEG	NEG	NEG	NEG	NEG	NEG	NEG	NEG	AMB	POS	NEG	POS	NEG	NEG	NEG	NEG	NEG
2013_PA07_Z3990	2013-007		17 years old, MALE	SWAB R INDEX FINGER ABSCESS	POS	POS	POS	POS	POS	POS	POS	POS	POS	POS	POS	POS	POS	POS	NEG	NEG	NEG	NEG	NEG	NEG	NEG	NEG	POS	POS	NEG	POS	NEG	NEG	NEG	NEG	NEG
2013_PA34_Z4017	2013-034		19 years old, MALE	WS	POS	POS	POS	POS	POS	POS	AMB	POS	POS	POS	POS	POS	POS	POS	NEG	NEG	NEG	NEG	NEG	NEG	NEG	NEG	AMB	POS	NEG	POS	NEG	NEG	NEG	NEG	NEG
2013_PA40_Z4023	2013-040		36 years old, MALE	SWAB RIGHT FOOT	POS	POS	POS	POS	POS	POS	AMB	POS	POS	POS	POS	POS	POS	POS	NEG	NEG	NEG	NEG	NEG	NEG	NEG	NEG	AMB	POS	NEG	POS	NEG	NEG	NEG	NEG	NEG
2013_PA48_Z4031	2013-048		57 years old, FEMALE	LEFT EAR SWAB	POS	POS	POS	POS	POS	POS	POS	POS	POS	POS	POS	POS	POS	POS	NEG	NEG	NEG	NEG	NEG	NEG	NEG	NEG	POS	POS	NEG	POS	NEG	NEG	NEG	NEG	NEG
2013_PA49_Z4032	2013-049		years old, FEMALE	RIGHT EAR SWAB	POS	POS	POS	POS	POS	POS	AMB	POS	POS	POS	POS	POS	POS	POS	NEG	NEG	NEG	NEG	NEG	NEG	NEG	NEG	AMB	POS	NEG	POS	NEG	NEG	NEG	NEG	NEG
2013_PA51_Z4034	2013-051		20 years old, MALE	SWAB LEFT HAND ABSCESS	POS	POS	POS	POS	POS	POS	POS	POS	POS	POS	POS	POS	POS	POS	NEG	NEG	NEG	NEG	NEG	NEG	NEG	NEG	POS	POS	NEG	POS	NEG	NEG	NEG	NEG	NEG
2013_PA70_Z4053	2013-070		5 years old, MALE	SWAB RIGHT GREAT TOE ABSCESS	POS	POS	POS	POS	POS	POS	AMB	POS	POS	POS	POS	POS	POS	POS	NEG	NEG	NEG	NEG	NEG	NEG	NEG	NEG	AMB	POS	NEG	POS	NEG	NEG	NEG	NEG	NEG
2013_PA84_Z4067	2013-084				POS	POS	POS	POS	POS	POS	AMB	POS	POS	POS	POS	POS	POS	POS	NEG	NEG	NEG	NEG	NEG	NEG	NEG	NEG	AMB	POS	NEG	POS	NEG	NEG	NEG	NEG	NEG
CC188-MSSA	CC188-MSSA				N= 5		%= 1.70																												
2012_206_Z3466 (968/12)	2012-206		70 years old, FEMALE	VOMITING ABD PAIN URINE CULTURE	POS	POS	POS	POS	POS	POS	POS	POS	POS	POS	POS	POS	POS	POS	NEG	NEG	NEG	NEG	NEG	NEG	NEG	NEG	POS	POS	NEG	POS	NEG	NEG	NEG	NEG	NEG
2012_251_Z3534 (3026/12)	2012-251		age unknown, FEMALE	INFECTED ECZEMA SKIN SWAB	POS	POS	POS	POS	POS	POS	POS	POS	POS	POS	POS	POS	POS	POS	NEG	NEG	NEG	NEG	NEG	NEG	NEG	NEG	POS	POS	NEG	POS	NEG	NEG	NEG	NEG	NEG
2012_320_Z3607 (7144/12)	2012-320		age unknown, FEMALE	SWAB	POS	POS	POS	POS	POS	POS	POS	POS	POS	POS	POS	POS	POS	POS	NEG	NEG	NEG	NEG	NEG	NEG	NEG	NEG	AMB	POS	NEG	POS	NEG	NEG	NEG	NEG	NEG
2012_321_Z3608 (7147/12)	2012-321		age unknown, MALE	MISC	POS	POS	POS	POS	POS	POS	POS	POS	AMB	POS	POS	POS	POS	POS	NEG	NEG	NEG	NEG	NEG	NEG	NEG	NEG	AMB	POS	NEG	POS	NEG	NEG	NEG	NEG	NEG
2012_358_Z3418 (1202-6332)	2012-358		age unknown, MALE	WOUND SWAB	POS	POS	POS	POS	POS	POS	POS	POS	POS	POS	POS	POS	POS	POS	NEG	NEG	NEG	NEG	NEG	NEG	NEG	NEG	AMB	POS	NEG	POS	NEG	NEG	NEG	NEG	NEG
CC398-MSSA	CC398-MSSA				N= 2		%= 0.68																												
2013_PA11_Z3994	2013-011		1 week old, FEMALE	LEFT EYE SWAB	POS	POS	POS	POS	POS	POS	POS	POS	POS	POS	POS	POS	POS	POS	NEG	NEG	NEG	NEG	NEG	NEG	NEG	NEG	NEG	NEG	NEG	POS	NEG	NEG	NEG	NEG	NEG
2013_PA57_Z4040	2013-057		82 years old, FEMALE	PERCUTANEOUS ABD DRAINAGE FLUID	POS	POS	POS	POS	POS	POS	POS	POS	POS	POS	POS	POS	POS	POS	NEG	NEG	NEG	NEG	NEG	NEG	NEG	NEG	NEG	NEG	NEG	POS	NEG	NEG	NEG	NEG	NEG
ST1852-MSSA	ST1852-MSSA				N= 1		%= 0.34																												
2012_277_Z3564 (4050/12)	2012-277	1-49-1-15-28-38-185	70+ years old, MALE with DM/ HEART DISEASE	CELLULITIS, SWAB	POS	POS	POS	POS	POS	POS	POS	POS	POS	POS	NEG	NEG	NEG	NEG	NEG	NEG	NEG	NEG	POS	POS	POS	POS	NEG	NEG	NEG	POS	NEG	NEG	NEG	NEG	NEG
CC1223/1594-MSSA	CC1223/1594-MSSA				N= 1		%= 0.34																												
2012_337_Z3624 (8070B/12)	2012-337	xx-xx-20-101-145-150-131	73 years old, MALE	LRTI RO SEPSIS BLOOD CULTURE	POS	POS	POS	NEG	POS	POS	AMB	POS	POS	POS	NEG	NEG	NEG	NEG	NEG	NEG	NEG	NEG	NEG	POS	NEG	NEG	NEG	NEG	NEG	POS	NEG	NEG	NEG	NEG	NEG
MSSA related to ST2250/2277	ST2250/2277-MSSA				N= 5		%= 1.70																												
2012_203_Z3463 (829/12)	2012-203		48 years old, MALE	SWAB	POS	POS	POS	NEG	POS	POS	POS	POS	POS	POS	AMB	NEG	AMB	AMB	NEG	NEG	NEG	NEG	NEG	NEG	NEG	NEG	NEG	NEG	NEG	POS	AMB	NEG	NEG	NEG	NEG
2012_269_Z3556 (3769A/12)	2012-269	151-325-215-34-175-xx-169	age unknown, MALE	BLOOD CULTURE	POS	POS	POS	NEG	POS	POS	POS	POS	POS	POS	NEG	NEG	NEG	NEG	NEG	NEG	NEG	NEG	NEG	NEG	NEG	NEG	NEG	NEG	NEG	POS	NEG	NEG	NEG	NEG	NEG
2012_292_Z3579	2012-292				POS	POS	POS	NEG	POS	AMB	AMB	POS	POS	POS	NEG	NEG	NEG	NEG	NEG	NEG	NEG	NEG	NEG	NEG	NEG	NEG	NEG	NEG	NEG	POS	NEG	NEG	NEG	NEG	NEG
2012_299_Z3586 (5487/12)	2012-299		1 week old, FEMALE	EYE SWAB	POS	POS	POS	NEG	POS	AMB	POS	POS	POS	POS	NEG	NEG	NEG	NEG	NEG	NEG	NEG	NEG	NEG	NEG	NEG	NEG	NEG	NEG	NEG	POS	NEG	NEG	NEG	NEG	NEG
2012_301_Z3588 (5717/12)	2012-301		age unknown, FEMALE	SWAB	POS	POS	POS	NEG	POS	AMB	AMB	POS	POS	POS	NEG	NEG	NEG	NEG	NEG	NEG	NEG	NEG	NEG	NEG	NEG	NEG	NEG	NEG	NEG	POS	NEG	NEG	NEG	NEG	NEG
ST2596dlv-MSSA	ST2596dlv-MSSA				N= 1		%= 0.34																												
2012_310_Z3597 (6034/12)	2012-310	151-269-20-34-256-261-49	1 month old, gender not recorded	WOUND SWAB	POS	POS	POS	NEG	NEG	POS	NEG	POS	POS	POS	NEG	NEG	NEG	NEG	NEG	NEG	NEG	NEG	NEG	POS	NEG	NEG	NEG	NEG	NEG	POS	NEG	NEG	NEG	NEG	NEG

Strain / Isolates	plasmin-sensitive surface protein	hypothetical protein from SCCmec elements	cassette chromosome recombinase genes A/B-2		potassium-trans-locating ATPase A, chain 2	potassium-transporting ATPase B, chain 1	potassium-trans-locating ATPase C, chain 2	sensor kinase protein	KDP operon transcrip-tional regulatory protein	methicillin-resistance regulatory protein	signal transducer protein MecR1	homolog of xylose repressor, associated with SCCmec-elements	cassette chromosome recombinase genes A/B-3		mercury resistance operon		cassette chromosome recombinase genes "ccrAA" (hypothetical) and ccrC					beta-lactamase	beta lactamase repressor (inhibitor)	beta-lactamase regulatory protein	erythromycin/ clindamycin resistance	erythromycin/ clindamycin resistance	erythromycin/ clindamycin resistance	Lincosamid-Nucleo-tidyltrans-ferase	energy-dependent efflux of erythro-mycin	macrolide efflux protein A	probable lysylphos-phatidyl-glycerol synthetase	virginiamycin A acetyl-transferase	acetyl-transferase inactivating streptogramin A	ATP binding protein, streptogramin-A-resistance	virginiamycin B hydrolase
	plsSCC (COL)	Q9XB68-dcs	ccrA-2	ccrB-2	kdpA-SCC	kdpB-SCC	kdpC-SCC	kdpD-SCC	kdpE-SCC	mecI	mecR	xylR	ccrA-3	ccrB-3	merA	merB	ccrAA (MRSAZH47) probe 1	ccrAA (MRSAZH47) probe 2	ccrC (85-2082)	ccrA-4	ccrB-4	blaZ	blaI	blaR	erm(A)	erm(B)	erm(C)	lnu(A)	msr(A)	mef(A)	mph(C)	vat(A)	vat(B)	vga(A) (BM 3327)	vgB(A)
**CC1-MSSA**																																			
2012_257_Z3544 (3216/12)	NEG	NEG	NEG	NEG	NEG	NEG	NEG	NEG	NEG	NEG	NEG	NEG	NEG	NEG	NEG	NEG	NEG	NEG	NEG	NEG	NEG	POS	POS	POS	NEG	NEG	NEG	NEG	NEG	NEG	NEG	NEG	NEG	NEG	NEG
2012_281_Z3568 (4696/12)	NEG	NEG	NEG	NEG	NEG	NEG	NEG	NEG	NEG	NEG	NEG	NEG	NEG	NEG	NEG	NEG	NEG	NEG	NEG	NEG	NEG	POS	POS	POS	POS	NEG	NEG	NEG	NEG	NEG	NEG	NEG	NEG	NEG	NEG
2012_324_Z3611 (7180/12)	NEG	NEG	NEG	NEG	NEG	NEG	NEG	NEG	NEG	NEG	NEG	NEG	NEG	NEG	NEG	NEG	NEG	NEG	NEG	NEG	NEG	NEG	NEG	NEG	NEG	NEG	NEG	NEG	NEG	NEG	NEG	NEG	NEG	NEG	NEG
2012_327_Z3614 (7347/12)	NEG	NEG	NEG	NEG	NEG	NEG	NEG	NEG	NEG	NEG	NEG	NEG	NEG	NEG	NEG	NEG	NEG	NEG	NEG	NEG	NEG	POS	POS	POS	NEG	NEG	NEG	NEG	POS	NEG	NEG	NEG	NEG	NEG	NEG
2012_351_Z3638 (8273B/12)	NEG	NEG	NEG	NEG	NEG	NEG	NEG	NEG	NEG	NEG	NEG	NEG	NEG	NEG	NEG	NEG	NEG	NEG	NEG	NEG	NEG	POS	POS	POS	NEG	NEG	NEG	NEG	POS	NEG	NEG	NEG	NEG	NEG	NEG
**CC5-MSSA**																																			
2012_188_Z3505 (503/12)	NEG	NEG	NEG	NEG	NEG	NEG	NEG	NEG	NEG	NEG	NEG	NEG	NEG	NEG	NEG	NEG	NEG	NEG	NEG	NEG	NEG	NEG	NEG	NEG	NEG	NEG	NEG	NEG	NEG	NEG	NEG	NEG	NEG	NEG	NEG
2012_219_Z3518 (1437/12)	NEG	NEG	NEG	NEG	NEG	NEG	NEG	NEG	NEG	NEG	NEG	NEG	NEG	NEG	NEG	NEG	NEG	NEG	NEG	NEG	NEG	NEG	NEG	NEG	NEG	NEG	NEG	NEG	NEG	NEG	NEG	NEG	NEG	NEG	NEG
2012_329_Z3616 (7577/12)	NEG	NEG	NEG	NEG	NEG	NEG	NEG	NEG	NEG	NEG	NEG	AMB	NEG	NEG	NEG	NEG	NEG	NEG	NEG	NEG	NEG	NEG	NEG	NEG	NEG	NEG	NEG	NEG	POS	NEG	POS	NEG	NEG	NEG	NEG
2012_377_Z3437 (6242)	NEG	NEG	NEG	NEG	NEG	NEG	NEG	NEG	NEG	NEG	NEG	NEG	NEG	NEG	POS	POS	NEG	NEG	NEG	NEG	NEG	POS	POS	POS	NEG	NEG	NEG	NEG	POS	NEG	POS	NEG	NEG	NEG	NEG
2013_PA04_Z3987	NEG	NEG	NEG	NEG	NEG	NEG	NEG	NEG	NEG	NEG	NEG	NEG	NEG	NEG	NEG	NEG	NEG	NEG	NEG	NEG	NEG	NEG	NEG	NEG	NEG	NEG	NEG	NEG	NEG	NEG	NEG	NEG	NEG	NEG	NEG
2013_PA32_Z4015	NEG	NEG	NEG	NEG	NEG	NEG	NEG	NEG	NEG	NEG	NEG	NEG	NEG	NEG	NEG	NEG	NEG	NEG	NEG	NEG	NEG	POS	POS	POS	NEG	NEG	NEG	NEG	NEG	NEG	NEG	NEG	NEG	NEG	NEG
2013_PA41_Z4024	NEG	NEG	NEG	NEG	NEG	NEG	NEG	NEG	NEG	NEG	NEG	NEG	NEG	NEG	POS	POS	NEG	NEG	NEG	NEG	NEG	POS	POS	POS	NEG	NEG	NEG	NEG	POS	NEG	POS	NEG	NEG	NEG	NEG
2013_PA44_Z4027	NEG	NEG	NEG	NEG	NEG	NEG	NEG	NEG	NEG	NEG	NEG	NEG	NEG	NEG	POS	POS	NEG	NEG	NEG	NEG	NEG	POS	POS	POS	NEG	NEG	NEG	NEG	POS	NEG	POS	NEG	NEG	NEG	NEG
**CC5-MSSA [PVL+]**																																			
2012_175_Z3497 (112/12)	NEG	NEG	NEG	NEG	NEG	NEG	NEG	NEG	NEG	NEG	NEG	NEG	NEG	NEG	NEG	NEG	NEG	NEG	NEG	NEG	NEG	POS	POS	POS	NEG	NEG	NEG	NEG	NEG	NEG	NEG	NEG	NEG	NEG	NEG
2012_193_Z3458 (617/12)	NEG	NEG	NEG	NEG	NEG	NEG	NEG	NEG	NEG	NEG	NEG	NEG	NEG	NEG	NEG	NEG	NEG	NEG	NEG	NEG	NEG	POS	POS	POS	NEG	NEG	NEG	NEG	NEG	NEG	NEG	NEG	NEG	NEG	NEG
2012_215_Z3474 (1341/12)	NEG	NEG	NEG	NEG	NEG	NEG	NEG	NEG	NEG	NEG	NEG	NEG	NEG	NEG	NEG	NEG	NEG	NEG	NEG	NEG	NEG	POS	POS	POS	NEG	NEG	NEG	NEG	NEG	NEG	NEG	NEG	NEG	NEG	NEG
2012_216_Z3475 (1370/12)	NEG	NEG	NEG	NEG	NEG	NEG	NEG	NEG	NEG	NEG	NEG	NEG	NEG	NEG	NEG	NEG	NEG	NEG	NEG	NEG	NEG	POS	POS	POS	NEG	NEG	NEG	NEG	NEG	NEG	NEG	NEG	NEG	NEG	NEG
2012_297_Z3584 (5140/12)	NEG	NEG	NEG	NEG	NEG	NEG	NEG	NEG	NEG	NEG	NEG	NEG	NEG	NEG	NEG	NEG	NEG	NEG	NEG	NEG	NEG	POS	POS	POS	NEG	NEG	NEG	NEG	NEG	NEG	NEG	NEG	NEG	NEG	NEG
2012_331_Z3618 (7644/12)	NEG	NEG	NEG	NEG	NEG	NEG	NEG	NEG	NEG	NEG	NEG	NEG	NEG	NEG	NEG	NEG	NEG	NEG	NEG	NEG	NEG	POS	POS	POS	NEG	NEG	NEG	NEG	NEG	NEG	NEG	NEG	NEG	NEG	NEG
2012_379_Z3439 (4126)	NEG	NEG	NEG	NEG	NEG	NEG	NEG	NEG	NEG	NEG	NEG	NEG	NEG	NEG	NEG	NEG	NEG	NEG	NEG	NEG	NEG	POS	POS	POS	NEG	NEG	NEG	NEG	NEG	NEG	NEG	NEG	NEG	NEG	NEG
2012_381_Z3441	NEG	NEG	NEG	NEG	NEG	NEG	NEG	NEG	NEG	NEG	NEG	AMB	NEG	NEG	NEG	NEG	NEG	NEG	NEG	NEG	NEG	POS	POS	POS	NEG	NEG	NEG	NEG	NEG	NEG	NEG	NEG	NEG	NEG	NEG
2013_PA39_Z4022	NEG	NEG	NEG	NEG	NEG	NEG	NEG	NEG	NEG	NEG	NEG	NEG	NEG	NEG	NEG	NEG	NEG	NEG	NEG	NEG	NEG	POS	POS	POS	NEG	NEG	NEG	NEG	NEG	NEG	NEG	NEG	NEG	NEG	NEG
2013_PA47_Z4030	NEG	NEG	NEG	NEG	NEG	NEG	NEG	NEG	NEG	NEG	NEG	NEG	NEG	NEG	NEG	NEG	NEG	NEG	NEG	NEG	NEG	POS	POS	POS	NEG	NEG	NEG	NEG	NEG	NEG	NEG	NEG	NEG	NEG	NEG
2013_PA61_I_Z4044	NEG	NEG	NEG	NEG	NEG	NEG	NEG	NEG	NEG	NEG	NEG	NEG	NEG	NEG	NEG	NEG	NEG	NEG	NEG	NEG	NEG	POS	POS	POS	NEG	NEG	NEG	NEG	NEG	NEG	NEG	NEG	NEG	NEG	NEG
2013_PA62_I_Z4045	NEG	NEG	NEG	NEG	NEG	NEG	NEG	NEG	NEG	NEG	NEG	NEG	NEG	NEG	NEG	NEG	NEG	NEG	NEG	NEG	NEG	POS	POS	POS	NEG	NEG	NEG	NEG	NEG	NEG	NEG	NEG	NEG	NEG	NEG
2013_PA74_Z4057	NEG	NEG	NEG	NEG	NEG	NEG	NEG	NEG	NEG	NEG	NEG	NEG	NEG	NEG	NEG	NEG	NEG	NEG	NEG	NEG	NEG	POS	POS	POS	NEG	NEG	NEG	NEG	NEG	NEG	NEG	NEG	NEG	NEG	NEG
2013_PA75_Z4058	NEG	NEG	NEG	NEG	NEG	NEG	NEG	NEG	NEG	NEG	NEG	NEG	NEG	NEG	NEG	NEG	NEG	NEG	NEG	NEG	NEG	POS	POS	POS	NEG	NEG	NEG	NEG	NEG	NEG	NEG	NEG	NEG	NEG	NEG
**CC5-MRSA-V [ACME+]**																																			
2012_373_Z3433 (1204-17312)	NEG	NEG	NEG	NEG	NEG	NEG	NEG	NEG	NEG	NEG	NEG	NEG	NEG	NEG	NEG	NEG	NEG	POS	POS	NEG	NEG	NEG	NEG	NEG	NEG	NEG	NEG	AMB	POS	NEG	POS	NEG	NEG	NEG	NEG
**CC6-MSSA**																																			
2012_173_Z3450 (98A/12)	NEG	NEG	NEG	NEG	NEG	NEG	NEG	NEG	NEG	NEG	NEG	NEG	NEG	NEG	NEG	NEG	NEG	NEG	NEG	NEG	NEG	NEG	NEG	NEG	NEG	NEG	NEG	NEG	NEG	NEG	NEG	NEG	NEG	NEG	NEG
2012_176_Z3498	NEG	NEG	NEG	NEG	NEG	NEG	NEG	NEG	NEG	NEG	NEG	NEG	NEG	NEG	NEG	NEG	NEG	NEG	NEG	NEG	NEG	NEG	NEG	NEG	NEG	NEG	NEG	NEG	NEG	NEG	NEG	NEG	NEG	NEG	NEG
2012_181_Z3452	NEG	NEG	NEG	NEG	NEG	NEG	NEG	NEG	NEG	NEG	NEG	NEG	NEG	NEG	NEG	NEG	NEG	NEG	NEG	NEG	NEG	NEG	NEG	NEG	NEG	NEG	NEG	NEG	NEG	NEG	NEG	NEG	NEG	NEG	NEG
2012_259_Z3546 (3279/12)	NEG	NEG	NEG	NEG	NEG	NEG	NEG	NEG	NEG	NEG	NEG	NEG	NEG	NEG	NEG	NEG	NEG	NEG	NEG	NEG	NEG	POS	POS	POS	NEG	NEG	NEG	NEG	NEG	NEG	NEG	NEG	NEG	NEG	NEG
2012_264_Z3551 (3407B/12)	NEG	NEG	NEG	NEG	NEG	NEG	NEG	NEG	NEG	NEG	NEG	NEG	NEG	NEG	NEG	NEG	NEG	NEG	NEG	NEG	NEG	POS	POS	POS	NEG	NEG	NEG	NEG	NEG	NEG	NEG	NEG	NEG	NEG	NEG
2012_273_Z3560 (3844/12)	NEG	NEG	NEG	NEG	NEG	NEG	NEG	NEG	NEG	NEG	NEG	NEG	NEG	NEG	NEG	NEG	NEG	NEG	NEG	NEG	NEG	POS	POS	POS	NEG	NEG	NEG	NEG	NEG	NEG	NEG	NEG	NEG	NEG	NEG
2012_312_Z3599 (6815/12)	NEG	NEG	NEG	NEG	NEG	NEG	NEG	NEG	NEG	NEG	NEG	NEG	NEG	NEG	NEG	NEG	NEG	NEG	NEG	NEG	NEG	POS	POS	POS	NEG	NEG	NEG	NEG	NEG	NEG	NEG	NEG	NEG	NEG	NEG
2012_338_Z3625	NEG	NEG	NEG	NEG	NEG	NEG	NEG	NEG	NEG	NEG	NEG	NEG	NEG	NEG	NEG	NEG	NEG	NEG	NEG	NEG	NEG	POS	POS	POS	NEG	NEG	NEG	NEG	NEG	NEG	NEG	NEG	NEG	NEG	NEG
2012_352_Z3639	NEG	NEG	NEG	NEG	NEG	NEG	NEG	NEG	NEG	NEG	NEG	NEG	NEG	NEG	NEG	NEG	NEG	NEG	NEG	NEG	NEG	POS	POS	POS	NEG	NEG	NEG	NEG	NEG	NEG	NEG	NEG	NEG	NEG	NEG
2012_368_Z3428	NEG	NEG	NEG	NEG	NEG	NEG	NEG	NEG	NEG	NEG	NEG	NEG	NEG	NEG	NEG	NEG	NEG	NEG	NEG	NEG	NEG	POS	POS	POS	NEG	NEG	NEG	NEG	NEG	NEG	NEG	NEG	NEG	NEG	NEG
2013_PA12_Z3995	NEG	NEG	NEG	NEG	NEG	NEG	NEG	NEG	NEG	NEG	NEG	NEG	NEG	NEG	NEG	NEG	NEG	NEG	NEG	NEG	NEG	POS	POS	POS	NEG	NEG	NEG	NEG	NEG	NEG	NEG	NEG	NEG	NEG	NEG
2013_PA28_Z4011	NEG	NEG	NEG	NEG	NEG	NEG	NEG	NEG	NEG	NEG	NEG	NEG	NEG	NEG	NEG	NEG	NEG	NEG	NEG	NEG	NEG	POS	POS	POS	NEG	NEG	NEG	NEG	NEG	NEG	NEG	NEG	NEG	NEG	NEG
**CC6-MSSA [PVL+]**																																			
2012_375_Z3435	NEG	NEG	NEG	NEG	NEG	NEG	NEG	NEG	NEG	NEG	NEG	NEG	NEG	NEG	NEG	NEG	NEG	NEG	NEG	NEG	NEG	POS	POS	POS	NEG	NEG	NEG	NEG	NEG	NEG	NEG	NEG	NEG	NEG	NEG
2013_PA77_Z4060	NEG	NEG	NEG	NEG	NEG	NEG	NEG	NEG	NEG	NEG	NEG	NEG	NEG	NEG	NEG	NEG	NEG	NEG	NEG	NEG	NEG	POS	POS	POS	NEG	NEG	NEG	NEG	NEG	NEG	NEG	NEG	NEG	NEG	NEG
**CC7-MSSA**																																			
2012_248_Z3532 (2864/12)	NEG	NEG	NEG	NEG	NEG	NEG	NEG	NEG	NEG	NEG	NEG	NEG	NEG	NEG	NEG	NEG	NEG	NEG	NEG	NEG	NEG	POS	POS	POS	NEG	NEG	NEG	NEG	NEG	NEG	NEG	NEG	NEG	NEG	NEG
2012_265_Z3552 (3518/12)	NEG	NEG	NEG	NEG	NEG	NEG	NEG	NEG	NEG	NEG	NEG	NEG	NEG	NEG	NEG	NEG	NEG	NEG	NEG	NEG	NEG	POS	POS	POS	NEG	NEG	NEG	POS	NEG	NEG	NEG	NEG	NEG	NEG	NEG
2013_PA53_Z4036	NEG	NEG	NEG	NEG	NEG	NEG	NEG	NEG	NEG	NEG	NEG	NEG	NEG	NEG	NEG	NEG	NEG	NEG	NEG	NEG	NEG	NEG	NEG	NEG	NEG	NEG	NEG	NEG	NEG	NEG	NEG	NEG	NEG	NEG	NEG
2013_PA83_Z4066	NEG	NEG	NEG	NEG	NEG	NEG	NEG	NEG	NEG	NEG	NEG	NEG	NEG	NEG	NEG	NEG	NEG	NEG	NEG	NEG	NEG	POS	POS	POS	NEG	NEG	NEG	NEG	NEG	NEG	NEG	NEG	NEG	NEG	NEG
**CC8-MSSA**																																			
2012_243_Z3528 (2587/12)	NEG	NEG	NEG	NEG	NEG	NEG	NEG	NEG	NEG	NEG	NEG	NEG	NEG	NEG	NEG	NEG	NEG	NEG	NEG	NEG	NEG	POS	POS	POS	NEG	NEG	NEG	NEG	NEG	NEG	NEG	NEG	NEG	NEG	NEG
2012_344_Z3631 (8605/12)	NEG	NEG	NEG	NEG	NEG	NEG	NEG	NEG	NEG	NEG	NEG	NEG	NEG	NEG	NEG	NEG	NEG	NEG	NEG	NEG	NEG	NEG	NEG	NEG	NEG	NEG	NEG	NEG	NEG	NEG	NEG	NEG	NEG	NEG	NEG
2012_346_Z3633 (8074/12)	NEG	NEG	NEG	NEG	NEG	NEG	NEG	NEG	NEG	NEG	NEG	NEG	NEG	NEG	NEG	NEG	NEG	NEG	NEG	NEG	NEG	POS	POS	POS	POS	NEG	NEG	NEG	NEG	NEG	NEG	NEG	NEG	NEG	NEG
2012_255_Z3538 (3205B/12)	NEG	NEG	NEG	NEG	NEG	NEG	NEG	NEG	NEG	NEG	NEG	NEG	NEG	NEG	NEG	NEG	NEG	NEG	NEG	NEG	NEG	POS	POS	POS	NEG	NEG	NEG	NEG	NEG	NEG	NEG	NEG	NEG	NEG	NEG
2012_280_Z3567 (4601/12)	NEG	NEG	NEG	NEG	NEG	NEG	NEG	NEG	NEG	NEG	NEG	NEG	NEG	NEG	NEG	NEG	NEG	NEG	NEG	POS	POS	POS	POS	POS	NEG	NEG	NEG	NEG	NEG	NEG	NEG	NEG	NEG	NEG	NEG
Trinidad&Tobago_2012_267 (3642/12)	NEG	NEG	NEG	NEG	NEG	NEG	NEG	NEG	NEG	NEG	NEG	NEG	NEG	NEG	NEG	NEG	NEG	NEG	NEG	NEG	NEG	POS	POS	POS	NEG	NEG	NEG	NEG	NEG	NEG	NEG	NEG	NEG	NEG	NEG
2012_365_Z3425 (1204-15838)	NEG	NEG	NEG	NEG	NEG	NEG	NEG	NEG	NEG	NEG	NEG	NEG	NEG	NEG	NEG	NEG	NEG	NEG	NEG	NEG	NEG	POS	POS	POS	NEG	NEG	NEG	NEG	NEG	NEG	NEG	NEG	NEG	NEG	NEG
2012_296_Z3583 (5118/12)	NEG	NEG	NEG	NEG	NEG	NEG	NEG	NEG	NEG	NEG	NEG	NEG	NEG	NEG	NEG	NEG	NEG	NEG	NEG	NEG	NEG	NEG	NEG	NEG	NEG	NEG	NEG	NEG	NEG	NEG	NEG	NEG	NEG	NEG	NEG
2012_318_Z3605 (6960/12)	NEG	NEG	NEG	NEG	NEG	NEG	NEG	NEG	NEG	NEG	NEG	NEG	NEG	NEG	NEG	NEG	NEG	NEG	NEG	NEG	NEG	NEG	NEG	NEG	NEG	NEG	NEG	NEG	NEG	NEG	NEG	NEG	NEG	NEG	NEG
2012_306_Z3593 (5861/12)	NEG	NEG	NEG	NEG	NEG	NEG	NEG	NEG	NEG	NEG	NEG	NEG	NEG	NEG	POS	POS	NEG	NEG	NEG	POS	POS	POS	POS	POS	NEG	NEG	NEG	NEG	NEG	NEG	NEG	NEG	NEG	NEG	NEG
2013_PA37_Z4020	NEG	NEG	NEG	NEG	NEG	NEG	NEG	NEG	NEG	NEG	NEG	NEG	NEG	NEG	NEG	NEG	NEG	NEG	NEG	NEG	NEG	POS	POS	POS	NEG	NEG	NEG	NEG	NEG	NEG	NEG	NEG	NEG	NEG	NEG
2013_PA38_Z4021	NEG	NEG	NEG	NEG	NEG	NEG	NEG	NEG	NEG	NEG	NEG	NEG	NEG	NEG	NEG	NEG	NEG	NEG	NEG	NEG	NEG	NEG	NEG	NEG	NEG	NEG	NEG	NEG	NEG	NEG	NEG	NEG	NEG	NEG	NEG
2013_PA52_Z4035	NEG	NEG	NEG	NEG	NEG	NEG	NEG	NEG	NEG	NEG	NEG	NEG	NEG	NEG	NEG	NEG	NEG	NEG	NEG	NEG	NEG	POS	POS	POS	NEG	NEG	NEG	NEG	AMB	NEG	NEG	NEG	NEG	NEG	NEG
**CC8-MSSA [PVL+]**																																			
2012_156_Z3489 (25917/11)	NEG	NEG	NEG	NEG	NEG	NEG	NEG	NEG	NEG	NEG	NEG	NEG	NEG	NEG	NEG	NEG	NEG	NEG	NEG	NEG	NEG	POS	POS	POS	NEG	NEG	NEG	NEG	NEG	NEG	NEG	NEG	NEG	NEG	NEG
2012_159_Z3492 (26310/11)	NEG	NEG	NEG	NEG	NEG	NEG	NEG	NEG	NEG	NEG	NEG	NEG	NEG	NEG	NEG	NEG	NEG	NEG	NEG	NEG	NEG	POS	POS	POS	NEG	NEG	NEG	NEG	NEG	NEG	NEG	NEG	NEG	NEG	NEG
2012_161_Z3445 (26430/11)	NEG	NEG	NEG	NEG	NEG	NEG	NEG	NEG	NEG	NEG	NEG	AMB	NEG	NEG	NEG	NEG	NEG	NEG	NEG	NEG	NEG	POS	POS	POS	NEG	NEG	NEG	NEG	NEG	NEG	NEG	NEG	NEG	NEG	NEG
2012_162_Z3512 (26433/11)	NEG	NEG	NEG	NEG	NEG	NEG	NEG	NEG	NEG	NEG	NEG	NEG	NEG	NEG	NEG	NEG	NEG	NEG	NEG	NEG	NEG	POS	POS	POS	NEG	NEG	NEG	NEG	NEG	NEG	NEG	NEG	NEG	NEG	NEG
2012_169_Z3495 (27194/11)	NEG	NEG	NEG	NEG	NEG	NEG	NEG	NEG	NEG	NEG	NEG	NEG	NEG	NEG	NEG	NEG	NEG	NEG	NEG	NEG	NEG	NEG	NEG	NEG	NEG	NEG	NEG	NEG	NEG	NEG	NEG	NEG	NEG	NEG	NEG
2012_170_Z3496 (35/12)	NEG	NEG	NEG	NEG	NEG	NEG	NEG	NEG	NEG	NEG	NEG	NEG	NEG	NEG	NEG	NEG	NEG	NEG	NEG	NEG	NEG	POS	POS	POS	NEG	NEG	NEG	NEG	NEG	NEG	NEG	NEG	NEG	NEG	NEG
2012_171_Z3448 (47/12)	NEG	NEG	NEG	NEG	NEG	NEG	NEG	NEG	NEG	NEG	NEG	NEG	NEG	NEG	NEG	NEG	NEG	NEG	NEG	NEG	NEG	POS	POS	POS	NEG	NEG	NEG	NEG	NEG	NEG	NEG	NEG	NEG	NEG	NEG
2012_177_Z3499 (184/12)	NEG	NEG	NEG	NEG	NEG	NEG	NEG	NEG	NEG	NEG	NEG	NEG	NEG	NEG	NEG	NEG	NEG	NEG	NEG	NEG	NEG	POS	POS	POS	NEG	NEG	NEG	NEG	NEG	NEG	NEG	NEG	NEG	NEG	NEG
2012_179_Z3515 (231/12)	NEG	NEG	NEG	NEG	NEG	NEG	NEG	NEG	NEG	NEG	NEG	NEG	NEG	NEG	NEG	NEG	NEG	NEG	NEG	NEG	NEG	POS	POS	POS	NEG	NEG	NEG	NEG	NEG	NEG	NEG	NEG	NEG	NEG	NEG
2012_185_Z3502 (437/12)	NEG	NEG	NEG	NEG	NEG	NEG	NEG	NEG	NEG	NEG	NEG	NEG	NEG	NEG	NEG	NEG	NEG	NEG	NEG	NEG	NEG	POS	POS	POS	NEG	NEG	NEG	NEG	NEG	NEG	NEG	NEG	NEG	NEG	NEG
2012_191_Z3456 (563/12)	NEG	NEG	NEG	NEG	NEG	NEG	NEG	NEG	NEG	NEG	NEG	NEG	NEG	NEG	NEG	NEG	NEG	NEG	NEG	NEG	NEG	POS	POS	POS	NEG	NEG	NEG	NEG	NEG	NEG	NEG	NEG	NEG	NEG	NEG
2012_194_Z3459	NEG	NEG	NEG	NEG	NEG	NEG	NEG	NEG	NEG	NEG	NEG	NEG	NEG	NEG	NEG	NEG	NEG	NEG	NEG	NEG	NEG	POS	POS	POS	NEG	NEG	NEG	NEG	NEG	NEG	NEG	NEG	NEG	NEG	NEG
2012_200_Z3460 (820/12)	NEG	NEG	NEG	NEG	NEG	NEG	NEG	NEG	NEG	NEG	NEG	AMB	NEG	NEG	NEG	NEG	NEG	NEG	NEG	NEG	NEG	POS	POS	POS	NEG	NEG	NEG	NEG	NEG	NEG	NEG	NEG	NEG	NEG	NEG
2012_207_Z3467 (1010/12)	NEG	NEG	NEG	NEG	NEG	NEG	NEG	NEG	NEG	NEG	NEG	NEG	NEG	NEG	NEG	NEG	NEG	NEG	NEG	NEG	NEG	POS	POS	POS	NEG	NEG	NEG	NEG	NEG	NEG	NEG	NEG	NEG	NEG	NEG
2012_212_Z3471 (1261/12)	NEG	NEG	NEG	NEG	NEG	NEG	NEG	NEG	NEG	NEG	NEG	NEG	NEG	NEG	NEG	NEG	NEG	NEG	NEG	NEG	NEG	POS	POS	POS	NEG	NEG	NEG	NEG	NEG	NEG	NEG	NEG	NEG	NEG	NEG
2012_220_Z3477 (1466/12)	NEG	NEG	NEG	NEG	NEG	NEG	NEG	NEG	NEG	NEG	NEG	NEG	NEG	NEG	NEG	NEG	NEG	NEG	NEG	NEG	NEG	POS	POS	POS	NEG	NEG	NEG	NEG	NEG	NEG	NEG	NEG	NEG	NEG	NEG
2012_224_Z3481 (1522/12)	NEG	NEG	NEG	NEG	NEG	NEG	NEG	NEG	NEG	NEG	NEG	NEG	NEG	NEG	NEG	NEG	NEG	NEG	NEG	NEG	NEG	POS	POS	POS	NEG	NEG	NEG	NEG	NEG	NEG	NEG	NEG	NEG	NEG	NEG
2012_226_Z3483 (1662/12)	NEG	NEG	NEG	NEG	NEG	NEG	NEG	NEG	NEG	NEG	NEG	NEG	NEG	NEG	NEG	NEG	NEG	NEG	NEG	NEG	NEG	POS	POS	POS	NEG	NEG	NEG	NEG	NEG	NEG	NEG	NEG	NEG	NEG	NEG
2012_242_Z3527 (2581/12)	NEG	NEG	NEG	NEG	NEG	NEG	NEG	NEG	NEG	NEG	NEG	NEG	NEG	NEG	NEG	NEG	NEG	NEG	NEG	NEG	NEG	POS	POS	POS	NEG	NEG	NEG	NEG	NEG	NEG	NEG	NEG	NEG	NEG	NEG
2012_245_Z3529 (2687/12)	NEG	NEG	NEG	NEG	NEG	NEG	NEG	NEG	NEG	NEG	NEG	NEG	NEG	NEG	NEG	NEG	NEG	NEG	NEG	NEG	NEG	POS	POS	POS	NEG	NEG	NEG	NEG	NEG	NEG	NEG	NEG	NEG	NEG	NEG
2012_253_Z3536 (3074/12)	NEG	NEG	NEG	NEG	NEG	NEG	NEG	NEG	NEG	NEG	NEG	NEG	NEG	NEG	NEG	NEG	NEG	NEG	NEG	NEG	NEG	POS	POS	POS	NEG	NEG	NEG	NEG	NEG	NEG	NEG	NEG	NEG	NEG	NEG
2012_258_Z3545 (3231/12)	NEG	NEG	NEG	NEG	NEG	NEG	NEG	NEG	NEG	NEG	NEG	NEG	NEG	NEG	NEG	NEG	NEG	NEG	NEG	NEG	NEG	POS	POS	POS	NEG	NEG	NEG	NEG	NEG	NEG	NEG	NEG	NEG	NEG	NEG
2012_261_Z3548 (3298/12)	NEG	NEG	NEG	NEG	NEG	NEG	NEG	NEG	NEG	NEG	NEG	NEG	NEG	NEG	NEG	NEG	NEG	NEG	NEG	NEG	NEG	POS	POS	POS	NEG	NEG	NEG	NEG	NEG	NEG	NEG	NEG	NEG	NEG	NEG
2012_270_Z3557 (3798/12)	NEG	NEG	NEG	NEG	NEG	NEG	NEG	NEG	NEG	NEG	NEG	NEG	NEG	NEG	NEG	NEG	NEG	NEG	NEG	NEG	NEG	POS	POS	POS	NEG	NEG	NEG	NEG	NEG	NEG	NEG	NEG	NEG	NEG	NEG
2012_274_Z3561 (3861/12)	NEG	NEG	NEG	NEG	NEG	NEG	NEG	NEG	NEG	NEG	NEG	NEG	NEG	NEG	NEG	NEG	NEG	NEG	NEG	NEG	NEG	POS	POS	POS	NEG	NEG	NEG	NEG	NEG	NEG	NEG	NEG	NEG	NEG	NEG
2012_282_Z3569 (4698/12)	NEG	NEG	NEG	NEG	NEG	NEG	NEG	NEG	NEG	NEG	NEG	NEG	NEG	NEG	NEG	NEG	NEG	NEG	NEG	NEG	NEG	POS	POS	POS	NEG	NEG	NEG	NEG	NEG	NEG	NEG	NEG	NEG	NEG	NEG
2012_286_Z3573 (4805/12)	NEG	NEG	NEG	NEG	NEG	NEG	NEG	NEG	NEG	NEG	NEG	NEG	NEG	NEG	NEG	NEG	NEG	NEG	NEG	NEG	NEG	POS	POS	POS	NEG	NEG	NEG	NEG	NEG	NEG	NEG	NEG	NEG	NEG	NEG
2012_293_Z3580 (5007/12)	NEG	NEG	NEG	NEG	NEG	NEG	NEG	NEG	NEG	NEG	NEG	NEG	NEG	NEG	NEG	NEG	NEG	NEG	NEG	NEG	NEG	NEG	NEG	NEG	NEG	NEG	NEG	NEG	NEG	NEG	NEG	NEG	NEG	NEG	NEG
2012_294_Z3581	NEG	NEG	NEG	NEG	NEG	NEG	NEG	NEG	NEG	NEG	NEG	NEG	NEG	NEG	NEG	NEG	NEG	NEG	NEG	NEG	NEG	NEG	NEG	NEG	NEG	NEG	NEG	NEG	NEG	NEG	NEG	NEG	NEG	NEG	NEG
2012_307_Z3594 (5918/12)	NEG	NEG	NEG	NEG	NEG	NEG	NEG	NEG	NEG	NEG	NEG	NEG	NEG	NEG	NEG	NEG	NEG	NEG	NEG	NEG	NEG	POS	POS	POS	NEG	NEG	NEG	NEG	NEG	NEG	NEG	NEG	NEG	NEG	NEG
2012_333_Z3620 (8101/12)	NEG	NEG	NEG	NEG	NEG	NEG	NEG	NEG	NEG	NEG	NEG	NEG	NEG	NEG	NEG	NEG	NEG	NEG	NEG	NEG	NEG	POS	POS	POS	NEG	NEG	NEG	NEG	NEG	NEG	NEG	NEG	NEG	NEG	NEG
2012_343_Z3630 (8195/12)	NEG	NEG	NEG	NEG	NEG	NEG	NEG	NEG	NEG	NEG	NEG	NEG	NEG	NEG	NEG	NEG	NEG	NEG	NEG	NEG	NEG	NEG	NEG	NEG	NEG	NEG	NEG	NEG	NEG	NEG	NEG	NEG	NEG	NEG	NEG
2012_349_Z3636 (7999A/12)	NEG	NEG	NEG	NEG	NEG	NEG	NEG	NEG	NEG	NEG	NEG	AMB	AMB	NEG	NEG	AMB	NEG	NEG	AMB	NEG	NEG	POS	POS	POS	NEG	NEG	NEG	NEG	NEG	NEG	NEG	NEG	NEG	NEG	NEG
2012_353_Z3640 (8575/12)	NEG	NEG	NEG	NEG	NEG	NEG	NEG	NEG	NEG	NEG	NEG	NEG	NEG	NEG	NEG	NEG	NEG	NEG	NEG	NEG	NEG	POS	POS	POS	NEG	NEG	NEG	NEG	NEG	NEG	NEG	NEG	NEG	NEG	NEG
2012_354_Z3641 (8408/12)	NEG	NEG	NEG	NEG	NEG	NEG	NEG	NEG	NEG	NEG	NEG	NEG	NEG	NEG	NEG	NEG	NEG	NEG	NEG	NEG	NEG	POS	POS	POS	NEG	NEG	NEG	NEG	NEG	NEG	NEG	NEG	NEG	NEG	NEG
2012_355_Z3642 (1112-58348)	NEG	NEG	NEG	NEG	NEG	NEG	NEG	NEG	NEG	NEG	NEG	NEG	NEG	NEG	NEG	NEG	NEG	NEG	NEG	NEG	NEG	POS	POS	POS	NEG	NEG	NEG	NEG	NEG	NEG	NEG	NEG	NEG	NEG	NEG
2012_357_Z3417 (1202-6360)	NEG	NEG	NEG	NEG	NEG	NEG	NEG	NEG	NEG	NEG	NEG	NEG	NEG	NEG	NEG	NEG	NEG	NEG	NEG	NEG	NEG	NEG	NEG	NEG	NEG	NEG	NEG	NEG	NEG	NEG	NEG	NEG	NEG	NEG	NEG
2012_364_Z3424 (1112-60417)	NEG	NEG	NEG	NEG	NEG	NEG	NEG	NEG	NEG	NEG	NEG	NEG	NEG	NEG	NEG	NEG	NEG	NEG	NEG	NEG	NEG	POS	POS	POS	NEG	NEG	NEG	NEG	NEG	NEG	NEG	NEG	NEG	NEG	NEG
2012_376_Z3436 (6256)	NEG	NEG	NEG	NEG	NEG	NEG	NEG	NEG	NEG	NEG	NEG	NEG	NEG	NEG	NEG	NEG	NEG	NEG	NEG	NEG	NEG	POS	POS	POS	NEG	NEG	NEG	NEG	NEG	NEG	NEG	NEG	NEG	NEG	NEG
2012_383_Z3443	NEG	NEG	NEG	NEG	NEG	NEG	NEG	NEG	NEG	NEG	NEG	NEG	NEG	NEG	NEG	NEG	NEG	NEG	NEG	NEG	NEG	POS	POS	POS	NEG	NEG	NEG	NEG	NEG	NEG	NEG	NEG	NEG	NEG	NEG
2013_PA01_Z3984	NEG	NEG	NEG	NEG	NEG	NEG	NEG	NEG	NEG	NEG	NEG	NEG	NEG	NEG	NEG	NEG	NEG	NEG	NEG	NEG	NEG	POS	POS	POS	NEG	NEG	NEG	NEG	NEG	NEG	NEG	NEG	NEG	NEG	NEG
2013_PA08_Z3991	NEG	NEG	NEG	NEG	NEG	NEG	NEG	NEG	NEG	NEG	NEG	NEG	NEG	NEG	NEG	NEG	NEG	NEG	NEG	NEG	NEG	POS	POS	POS	NEG	NEG	NEG	NEG	NEG	NEG	NEG	NEG	NEG	NEG	NEG
2013_PA10_Z3993	NEG	NEG	NEG	NEG	NEG	NEG	NEG	NEG	NEG	NEG	NEG	NEG	NEG	NEG	NEG	NEG	NEG	NEG	NEG	NEG	NEG	POS	POS	POS	NEG	NEG	NEG	NEG	NEG	NEG	NEG	NEG	NEG	NEG	NEG
2013_PA13_Z3996	NEG	NEG	NEG	NEG	NEG	NEG	NEG	NEG	NEG	NEG	NEG	NEG	NEG	NEG	NEG	NEG	NEG	NEG	NEG	NEG	NEG	POS	POS	POS	NEG	NEG	NEG	NEG	NEG	NEG	NEG	NEG	NEG	NEG	NEG
2013_PA14_Z3997	NEG	NEG	NEG	NEG	NEG	NEG	NEG	NEG	NEG	NEG	NEG	NEG	NEG	NEG	NEG	NEG	NEG	NEG	NEG	NEG	NEG	POS	POS	POS	NEG	NEG	NEG	NEG	NEG	NEG	NEG	NEG	NEG	NEG	NEG
2013_PA17_Z4000	NEG	NEG	NEG	NEG	NEG	NEG	NEG	NEG	NEG	NEG	NEG	NEG	NEG	NEG	NEG	NEG	NEG	NEG	NEG	NEG	NEG	POS	POS	POS	NEG	NEG	NEG	NEG	NEG	NEG	NEG	NEG	NEG	NEG	NEG
2013_PA26_Z4009	NEG	NEG	NEG	NEG	NEG	NEG	NEG	NEG	NEG	NEG	NEG	NEG	NEG	NEG	NEG	NEG	NEG	NEG	NEG	NEG	NEG	POS	POS	POS	NEG	NEG	NEG	NEG	NEG	NEG	NEG	NEG	NEG	NEG	NEG
2013_PA27_Z4010	NEG	NEG	NEG	NEG	NEG	NEG	NEG	NEG	NEG	NEG	NEG	NEG	NEG	NEG	NEG	NEG	NEG	NEG	NEG	NEG	NEG	POS	POS	POS	NEG	NEG	NEG	NEG	NEG	NEG	NEG	NEG	NEG	NEG	NEG
2013_PA42_Z4025	NEG	NEG	NEG	NEG	NEG	NEG	NEG	NEG	NEG	NEG	NEG	NEG	NEG	NEG	NEG	NEG	NEG	NEG	NEG	NEG	NEG	POS	POS	POS	NEG	NEG	NEG	NEG	NEG	NEG	NEG	NEG	NEG	NEG	NEG
2013_PA43_Z4026	NEG	NEG	NEG	NEG	NEG	NEG	NEG	NEG	NEG	NEG	NEG	NEG	NEG	NEG	NEG	NEG	NEG	NEG	NEG	NEG	NEG	POS	POS	POS	NEG	NEG	NEG	NEG	NEG	NEG	NEG	NEG	NEG	NEG	NEG
2013_PA45_Z4028	NEG	NEG	NEG	NEG	NEG	NEG	NEG	NEG	NEG	NEG	NEG	NEG	NEG	NEG	NEG	NEG	NEG	NEG	NEG	NEG	NEG	POS	POS	POS	NEG	NEG	NEG	NEG	NEG	NEG	NEG	NEG	NEG	NEG	NEG
2013_PA56_Z4039	NEG	NEG	NEG	NEG	NEG	NEG	NEG	NEG	NEG	NEG	NEG	NEG	NEG	NEG	NEG	NEG	NEG	NEG	NEG	NEG	NEG	POS	POS	POS	NEG	NEG	NEG	NEG	NEG	NEG	NEG	NEG	NEG	NEG	NEG
2013_PA58_Z4041	NEG	NEG	NEG	NEG	NEG	NEG	NEG	NEG	NEG	NEG	NEG	NEG	NEG	NEG	NEG	NEG	NEG	NEG	NEG	NEG	NEG	POS	POS	POS	NEG	NEG	NEG	NEG	NEG	NEG	NEG	NEG	NEG	NEG	NEG
2013_PA64_Z4047	NEG	NEG	NEG	NEG	NEG	NEG	NEG	NEG	NEG	NEG	NEG	NEG	NEG	NEG	NEG	NEG	NEG	NEG	NEG	NEG	NEG	POS	POS	POS	NEG	NEG	NEG	NEG	NEG	NEG	NEG	NEG	NEG	NEG	NEG
2013_PA66_Z4049	NEG	NEG	NEG	NEG	NEG	NEG	NEG	NEG	NEG	NEG	NEG	NEG	NEG	NEG	NEG	NEG	NEG	NEG	NEG	NEG	NEG	POS	POS	POS	NEG	NEG	NEG	NEG	NEG	NEG	NEG	NEG	NEG	NEG	NEG
2013_PA67_Z4050	NEG	NEG	NEG	NEG	NEG	NEG	NEG	NEG	NEG	NEG	NEG	NEG	NEG	NEG	NEG	NEG	NEG	NEG	NEG	NEG	NEG	POS	POS	POS	NEG	NEG	NEG	NEG	NEG	NEG	NEG	NEG	NEG	NEG	NEG
2013_PA69_Z4052	NEG	NEG	NEG	NEG	NEG	NEG	NEG	NEG	NEG	NEG	NEG	NEG	NEG	NEG	NEG	NEG	NEG	NEG	NEG	NEG	NEG	POS	POS	POS	NEG	NEG	NEG	NEG	NEG	NEG	NEG	NEG	NEG	NEG	NEG
2013_PA72_Z4055	NEG	NEG	NEG	NEG	NEG	NEG	NEG	NEG	NEG	NEG	NEG	NEG	NEG	NEG	NEG	NEG	NEG	NEG	NEG	NEG	NEG	POS	POS	POS	NEG	NEG	NEG	NEG	NEG	NEG	NEG	NEG	NEG	NEG	NEG
2013_PA76_Z4059	NEG	NEG	NEG	NEG	NEG	NEG	NEG	NEG	NEG	NEG	NEG	NEG	NEG	NEG	NEG	NEG	NEG	NEG	NEG	NEG	NEG	POS	POS	POS	NEG	NEG	NEG	NEG	NEG	NEG	NEG	NEG	NEG	NEG	NEG
2013_PA80_Z4063	NEG	NEG	NEG	NEG	NEG	NEG	NEG	NEG	NEG	NEG	NEG	NEG	NEG	NEG	NEG	NEG	NEG	NEG	NEG	NEG	NEG	POS	POS	POS	NEG	NEG	NEG	NEG	POS	NEG	NEG	NEG	NEG	NEG	NEG

Strain / Isolates	plasmin-sensitive surface protein	hypothetical protein from SCCmec elements	cassette chromosome recombinase genes A/B-2		potassium-trans-locating ATPase A, chain 2	potassium-transporting ATPase B, chain 1	potassium-trans-locating ATPase C, chain 2	sensor kinase protein	KDP operon transcrip-tional regulatory protein	methicillin-resistance regulatory protein	signal transducer protein MecR1	homolog of xylose repressor, associated with SCCmec-elements	cassette chromosome recombinase genes A/B-3		mercury resistance operon		cassette chromosome recombinase genes "ccrAA" (hypothetical) and ccrC					beta-lactamase	beta lactamase repressor (inhibitor)	beta-lactamase regulatory protein	erythromycin/ clindamycin resistance	erythromycin/ clindamycin resistance	erythromycin/ clindamycin resistance	Lincosamid-Nucleo-tidyltrans-ferase	energy-dependent efflux of erythro-mycin	macrolide efflux protein A	probable lysylphos-phatidyl-glycerol synthetase	virginiamycin A acetyl-transferase	acetyl-transferase inactivating streptogramin A	ATP binding protein, streptogramin-A-resistance	virginiamycin B hydrolase
	plsSCC (COL)	Q9XB68-dcs	ccrA-2	ccrB-2	kdpA-SCC	kdpB-SCC	kdpC-SCC	kdpD-SCC	kdpE-SCC	mecI	mecR	xylR	ccrA-3	ccrB-3	merA	merB	ccrAA (MRSAZH47) probe 1	ccrAA (MRSAZH47) probe 2	ccrC (85-2082)	ccrA-4	ccrB-4	blaZ	blaI	blaR	erm(A)	erm(B)	erm(C)	lnu(A)	msr(A)	mef(A)	mph(C)	vat(A)	vat(B)	vga(A) (BM 3327)	vgB(A)
**ST8-MRSA-IV, USA300**																																			
2012_158_Z3491 (26049A/11)	NEG	POS	POS	POS	NEG	NEG	NEG	NEG	NEG	NEG	NEG	NEG	NEG	NEG	NEG	NEG	NEG	NEG	NEG	NEG	NEG	POS	POS	POS	NEG	NEG	NEG	NEG	POS	NEG	POS	NEG	NEG	NEG	NEG
2012_165_Z3513 (26656/11)	NEG	POS	POS	POS	NEG	NEG	NEG	NEG	NEG	NEG	NEG	NEG	NEG	NEG	NEG	NEG	NEG	NEG	NEG	NEG	NEG	POS	POS	POS	NEG	NEG	NEG	NEG	POS	NEG	POS	NEG	NEG	NEG	NEG
2012_214_Z3473 (1322/12)	NEG	POS	POS	POS	NEG	NEG	NEG	NEG	NEG	NEG	NEG	NEG	NEG	NEG	NEG	NEG	NEG	NEG	NEG	NEG	NEG	POS	POS	POS	NEG	NEG	NEG	NEG	POS	NEG	POS	NEG	NEG	NEG	NEG
2012_227_Z3484 (1688/12)	NEG	POS	POS	POS	NEG	NEG	NEG	NEG	NEG	NEG	NEG	NEG	NEG	NEG	NEG	NEG	NEG	NEG	NEG	NEG	NEG	POS	POS	POS	NEG	NEG	NEG	NEG	POS	NEG	POS	NEG	NEG	NEG	NEG
2012_263_Z3550 (3355/12)	NEG	POS	POS	POS	NEG	NEG	NEG	NEG	NEG	NEG	NEG	NEG	NEG	NEG	NEG	NEG	NEG	NEG	NEG	NEG	NEG	POS	POS	POS	NEG	NEG	NEG	NEG	POS	NEG	POS	NEG	NEG	NEG	NEG
2012_266_Z3553	NEG	POS	POS	POS	NEG	NEG	NEG	NEG	NEG	NEG	NEG	NEG	AMB	NEG	NEG	NEG	NEG	NEG	NEG	NEG	NEG	POS	POS	POS	NEG	NEG	NEG	NEG	POS	NEG	POS	NEG	NEG	NEG	NEG
2012_284_Z3571 (4729/12)	NEG	POS	POS	POS	NEG	NEG	NEG	NEG	NEG	NEG	NEG	NEG	NEG	NEG	NEG	NEG	NEG	NEG	NEG	NEG	NEG	POS	POS	POS	NEG	NEG	NEG	NEG	POS	NEG	POS	NEG	NEG	NEG	NEG
2012_298_Z3585 (5203/12)	NEG	POS	POS	POS	NEG	NEG	NEG	NEG	NEG	NEG	NEG	NEG	NEG	NEG	NEG	NEG	NEG	NEG	NEG	NEG	NEG	POS	POS	POS	NEG	NEG	NEG	NEG	POS	NEG	POS	NEG	NEG	NEG	NEG
2012_304_Z3591 (5818/12)	NEG	POS	POS	POS	NEG	NEG	NEG	NEG	NEG	NEG	NEG	NEG	NEG	NEG	NEG	NEG	NEG	NEG	NEG	NEG	NEG	POS	POS	POS	NEG	NEG	NEG	NEG	POS	NEG	POS	NEG	NEG	NEG	NEG
Z3595_TT308 - (EFC74B77-3925-484F-A4CF-A1F7DC	NEG	POS	POS	POS	NEG	NEG	NEG	NEG	NEG	NEG	NEG	NEG	NEG	NEG	NEG	NEG	NEG	NEG	NEG	NEG	NEG	POS	POS	POS	NEG	NEG	NEG	NEG	POS	NEG	POS	NEG	NEG	NEG	NEG
2012_369_Z3429 (1112-58547)	NEG	POS	POS	POS	NEG	NEG	NEG	NEG	NEG	NEG	NEG	NEG	NEG	NEG	NEG	NEG	NEG	NEG	NEG	NEG	NEG	POS	AMB	POS	NEG	NEG	NEG	NEG	NEG	NEG	NEG	NEG	NEG	NEG	NEG
2012_374_Z3434 (1203-13527)	NEG	POS	POS	POS	NEG	NEG	NEG	NEG	NEG	NEG	NEG	NEG	NEG	NEG	NEG	NEG	NEG	NEG	NEG	NEG	NEG	POS	POS	POS	NEG	NEG	NEG	NEG	POS	NEG	POS	NEG	NEG	NEG	NEG
2013_PA06_Z3989	NEG	POS	POS	POS	NEG	NEG	NEG	NEG	NEG	NEG	NEG	NEG	NEG	NEG	NEG	NEG	NEG	NEG	NEG	NEG	NEG	POS	POS	POS	NEG	NEG	NEG	NEG	POS	NEG	POS	NEG	NEG	NEG	NEG
2013_PA55_Z4038	NEG	POS	POS	POS	NEG	NEG	NEG	NEG	NEG	NEG	NEG	NEG	NEG	NEG	NEG	NEG	NEG	NEG	NEG	NEG	NEG	POS	POS	POS	NEG	NEG	NEG	NEG	POS	NEG	POS	NEG	NEG	NEG	NEG
2013_PA71_Z4054	NEG	POS	POS	POS	NEG	NEG	NEG	NEG	NEG	NEG	NEG	NEG	NEG	NEG	NEG	NEG	NEG	NEG	NEG	NEG	NEG	POS	POS	POS	NEG	NEG	NEG	NEG	NEG	NEG	NEG	NEG	NEG	NEG	NEG
**ST8-MRSA-IV [PVL+/ACME+], US**																																			
2012_180_Z3501 (232/12)	NEG	POS	NEG	NEG	NEG	NEG	NEG	NEG	NEG	NEG	NEG	NEG	NEG	NEG	NEG	NEG	NEG	NEG	NEG	NEG	NEG	POS	POS	POS	NEG	NEG	NEG	NEG	POS	NEG	POS	NEG	NEG	NEG	NEG
2013_PA68_Z4051	NEG	POS	NEG	NEG	NEG	NEG	NEG	NEG	NEG	NEG	NEG	NEG	NEG	NEG	NEG	NEG	NEG	NEG	NEG	NEG	NEG	NEG	NEG	NEG	NEG	NEG	NEG	NEG	POS	NEG	POS	NEG	NEG	NEG	NEG
**ST72-MSSA**																																			
2012_174_Z3451 (102/12)	NEG	NEG	NEG	NEG	NEG	NEG	NEG	NEG	NEG	NEG	NEG	NEG	NEG	NEG	NEG	NEG	NEG	NEG	NEG	NEG	NEG	POS	POS	POS	NEG	NEG	NEG	NEG	NEG	NEG	NEG	NEG	NEG	NEG	NEG
2012_223_Z3480 (1506A/12)	NEG	NEG	NEG	NEG	NEG	NEG	NEG	NEG	NEG	NEG	NEG	NEG	NEG	NEG	NEG	NEG	NEG	NEG	NEG	NEG	NEG	POS	POS	POS	NEG	NEG	NEG	NEG	NEG	NEG	NEG	NEG	NEG	NEG	NEG
2012_236_Z3523 (2086/12)	NEG	NEG	NEG	NEG	NEG	NEG	NEG	NEG	NEG	NEG	NEG	NEG	NEG	NEG	NEG	NEG	NEG	NEG	NEG	NEG	NEG	POS	POS	POS	NEG	NEG	NEG	NEG	NEG	NEG	NEG	NEG	NEG	NEG	NEG
2012_239_Z3525 (2346/12)	NEG	NEG	NEG	NEG	NEG	NEG	NEG	NEG	NEG	NEG	NEG	NEG	NEG	NEG	NEG	NEG	NEG	NEG	NEG	NEG	NEG	POS	POS	POS	NEG	NEG	NEG	NEG	NEG	NEG	NEG	NEG	NEG	NEG	NEG
2012_252_Z3535 (3030/12)	NEG	NEG	NEG	NEG	NEG	NEG	NEG	NEG	NEG	NEG	NEG	NEG	NEG	NEG	NEG	NEG	NEG	NEG	NEG	NEG	NEG	POS	POS	POS	NEG	NEG	NEG	NEG	NEG	NEG	NEG	NEG	NEG	NEG	NEG
2012_271_Z3558 (3799/12)	NEG	NEG	NEG	NEG	NEG	NEG	NEG	NEG	NEG	NEG	NEG	NEG	NEG	NEG	NEG	NEG	NEG	NEG	NEG	NEG	NEG	POS	POS	POS	NEG	NEG	NEG	NEG	NEG	NEG	NEG	NEG	NEG	NEG	NEG
2012_276_Z3563	NEG	NEG	NEG	NEG	NEG	NEG	NEG	NEG	NEG	NEG	NEG	NEG	NEG	NEG	NEG	NEG	NEG	NEG	NEG	NEG	NEG	POS	POS	POS	NEG	NEG	NEG	NEG	NEG	NEG	NEG	NEG	NEG	NEG	NEG
2012_300_Z3587 (5638/12)	NEG	NEG	NEG	NEG	NEG	NEG	NEG	NEG	NEG	NEG	NEG	NEG	NEG	NEG	NEG	NEG	NEG	NEG	NEG	NEG	NEG	POS	POS	POS	NEG	NEG	NEG	NEG	NEG	NEG	NEG	NEG	NEG	NEG	NEG
2012_316_Z3603 (6941/12)	NEG	NEG	NEG	NEG	NEG	NEG	NEG	NEG	NEG	NEG	NEG	NEG	NEG	NEG	NEG	NEG	NEG	NEG	NEG	NEG	NEG	POS	POS	POS	NEG	NEG	NEG	NEG	NEG	NEG	NEG	NEG	NEG	NEG	NEG
2012_319_Z3606 (7019/12)	NEG	NEG	NEG	NEG	NEG	NEG	NEG	NEG	NEG	NEG	NEG	NEG	NEG	NEG	NEG	NEG	NEG	NEG	NEG	NEG	NEG	POS	POS	POS	NEG	NEG	NEG	NEG	NEG	NEG	NEG	NEG	NEG	NEG	NEG
2012_371_Z3431 (1204-17577)	NEG	NEG	NEG	NEG	NEG	NEG	NEG	NEG	NEG	NEG	NEG	NEG	NEG	NEG	NEG	NEG	NEG	NEG	NEG	NEG	NEG	POS	POS	POS	NEG	NEG	NEG	NEG	NEG	NEG	NEG	NEG	NEG	NEG	NEG
2013_PA05_Z3988	NEG	NEG	NEG	NEG	NEG	NEG	NEG	NEG	NEG	NEG	NEG	NEG	NEG	NEG	NEG	NEG	NEG	NEG	NEG	NEG	NEG	POS	POS	POS	NEG	NEG	NEG	NEG	NEG	NEG	NEG	NEG	NEG	NEG	NEG
2013_PA65_Z4048	NEG	NEG	NEG	NEG	NEG	NEG	NEG	NEG	NEG	NEG	NEG	NEG	NEG	NEG	NEG	NEG	NEG	NEG	NEG	NEG	NEG	POS	POS	POS	NEG	NEG	NEG	NEG	NEG	NEG	NEG	NEG	NEG	NEG	NEG
2013_PA73_Z4056	NEG	NEG	NEG	NEG	NEG	NEG	NEG	NEG	NEG	NEG	NEG	NEG	NEG	NEG	NEG	NEG	NEG	NEG	NEG	NEG	NEG	POS	POS	POS	NEG	NEG	NEG	NEG	NEG	NEG	NEG	NEG	NEG	NEG	NEG
**ST72-MSSA-SCCfus**																																			
2013_PA16_Z3999	NEG	NEG	NEG	NEG	NEG	NEG	NEG	NEG	NEG	NEG	NEG	NEG	NEG	NEG	NEG	NEG	NEG	NEG	NEG	NEG	NEG	POS	POS	POS	NEG	NEG	NEG	NEG	NEG	NEG	NEG	NEG	NEG	NEG	NEG
**ST72-MSSA-SCCfus/kdp**																																			
2012_361_Z3421 (1201-1299)	NEG	NEG	NEG	NEG	POS	POS	POS	POS	POS	NEG	NEG	NEG	NEG	NEG	NEG	NEG	NEG	NEG	NEG	NEG	NEG	POS	POS	POS	NEG	NEG	NEG	NEG	NEG	NEG	NEG	NEG	NEG	NEG	NEG
**ST239-MRSA-III&SCCmer**																																			
2012_157_Z3490	NEG	NEG	NEG	NEG	NEG	NEG	NEG	NEG	NEG	POS	POS	POS	POS	POS	POS	POS	NEG	AMB	POS	NEG	NEG	POS	POS	POS	POS	NEG	NEG	NEG	NEG	NEG	NEG	NEG	NEG	NEG	NEG
2012_168_Z3494 (27129/11)	NEG	NEG	NEG	NEG	NEG	NEG	NEG	NEG	NEG	POS	POS	POS	POS	POS	POS	POS	NEG	AMB	POS	NEG	NEG	POS	POS	POS	POS	NEG	NEG	NEG	NEG	NEG	NEG	NEG	NEG	NEG	NEG
2012_195_Z3508 (619/12)	NEG	NEG	NEG	NEG	NEG	NEG	NEG	NEG	NEG	POS	POS	POS	POS	POS	POS	POS	NEG	AMB	POS	NEG	NEG	POS	POS	POS	POS	NEG	NEG	NEG	NEG	NEG	NEG	NEG	NEG	NEG	NEG
2012_222_Z3479 (1489/12)	NEG	NEG	NEG	NEG	NEG	NEG	NEG	NEG	NEG	POS	POS	POS	POS	POS	POS	POS	NEG	NEG	POS	NEG	NEG	POS	POS	POS	POS	NEG	NEG	NEG	NEG	NEG	NEG	NEG	NEG	NEG	NEG
2012_233_Z3540 (1943/12)	NEG	NEG	NEG	NEG	NEG	NEG	NEG	NEG	NEG	POS	POS	POS	POS	POS	POS	POS	NEG	NEG	POS	NEG	NEG	POS	POS	POS	POS	NEG	NEG	NEG	NEG	NEG	NEG	NEG	NEG	NEG	NEG
2012_237_Z3541	NEG	NEG	NEG	NEG	NEG	NEG	NEG	NEG	NEG	POS	POS	POS	POS	POS	POS	POS	NEG	AMB	POS	NEG	NEG	POS	POS	POS	POS	NEG	NEG	NEG	NEG	NEG	NEG	NEG	NEG	NEG	NEG
2012_262_Z3549	NEG	NEG	NEG	NEG	NEG	NEG	NEG	NEG	NEG	POS	POS	POS	POS	POS	POS	POS	NEG	AMB	POS	NEG	NEG	POS	POS	POS	POS	NEG	NEG	NEG	NEG	NEG	NEG	NEG	NEG	NEG	NEG
2012_275_Z3562 (3893/12)	NEG	NEG	NEG	NEG	NEG	NEG	NEG	NEG	NEG	POS	POS	POS	POS	POS	POS	POS	NEG	AMB	POS	NEG	NEG	POS	POS	POS	POS	NEG	NEG	NEG	NEG	NEG	AMB	NEG	NEG	NEG	NEG
2012_291_Z3578	NEG	NEG	NEG	NEG	NEG	NEG	NEG	NEG	NEG	POS	POS	POS	POS	POS	POS	POS	NEG	NEG	POS	NEG	NEG	POS	POS	POS	POS	NEG	NEG	NEG	NEG	NEG	NEG	NEG	NEG	NEG	NEG
2012_314_Z3601 (6939/12)	NEG	NEG	AMB	AMB	NEG	NEG	NEG	NEG	NEG	POS	POS	POS	POS	POS	POS	POS	NEG	NEG	POS	NEG	NEG	POS	POS	POS	POS	NEG	NEG	NEG	POS	NEG	POS	NEG	NEG	NEG	NEG
2012323_Z3610	NEG	NEG	NEG	NEG	NEG	NEG	NEG	NEG	NEG	POS	POS	POS	POS	POS	POS	POS	NEG	AMB	POS	NEG	NEG	POS	POS	POS	POS	NEG	NEG	NEG	NEG	NEG	NEG	NEG	NEG	NEG	NEG
2012330_Z3617 (7605/12)	NEG	NEG	NEG	NEG	NEG	NEG	NEG	NEG	NEG	POS	POS	POS	POS	POS	POS	POS	NEG	AMB	POS	NEG	NEG	POS	POS	POS	POS	NEG	NEG	NEG	NEG	NEG	NEG	NEG	NEG	NEG	NEG
2012332_Z3619 (8076/12)	NEG	NEG	NEG	NEG	NEG	NEG	NEG	NEG	NEG	POS	POS	POS	POS	POS	POS	POS	NEG	NEG	POS	NEG	NEG	POS	POS	POS	POS	NEG	NEG	NEG	NEG	NEG	NEG	NEG	NEG	NEG	NEG
2012339_Z3626 (8558/12)	NEG	NEG	NEG	NEG	NEG	NEG	NEG	NEG	NEG	POS	POS	POS	POS	POS	POS	POS	NEG	NEG	POS	NEG	NEG	POS	POS	POS	POS	NEG	NEG	NEG	NEG	NEG	NEG	NEG	NEG	NEG	NEG
2012340_Z3627 (8523/12)	NEG	NEG	NEG	NEG	NEG	NEG	NEG	NEG	NEG	POS	POS	POS	POS	POS	POS	POS	NEG	NEG	POS	NEG	NEG	POS	POS	POS	POS	NEG	NEG	NEG	NEG	NEG	NEG	NEG	NEG	NEG	NEG
2012348_Z3635	NEG	NEG	NEG	NEG	NEG	NEG	NEG	NEG	NEG	POS	POS	POS	POS	POS	POS	POS	NEG	NEG	POS	NEG	NEG	POS	POS	POS	POS	NEG	NEG	NEG	NEG	NEG	NEG	NEG	NEG	NEG	NEG
2012350_Z3637	NEG	NEG	NEG	NEG	NEG	NEG	NEG	NEG	NEG	POS	POS	POS	POS	POS	POS	POS	NEG	NEG	POS	NEG	NEG	POS	POS	POS	POS	NEG	NEG	NEG	NEG	NEG	NEG	NEG	NEG	NEG	NEG
2012_370_Z3430 (1112-52177)	NEG	NEG	NEG	NEG	NEG	NEG	NEG	NEG	NEG	POS	POS	POS	POS	POS	POS	POS	NEG	AMB	POS	NEG	NEG	POS	POS	POS	POS	NEG	NEG	NEG	NEG	NEG	NEG	NEG	NEG	NEG	NEG
2012_378_Z3438 (4139)	NEG	NEG	NEG	NEG	NEG	NEG	NEG	NEG	NEG	POS	POS	POS	POS	POS	POS	POS	NEG	AMB	POS	NEG	NEG	POS	POS	POS	POS	NEG	NEG	AMB	NEG	NEG	NEG	NEG	NEG	NEG	NEG
2013_PA21_Z4004	NEG	NEG	NEG	NEG	NEG	NEG	NEG	NEG	NEG	POS	POS	POS	POS	POS	POS	POS	NEG	NEG	POS	NEG	NEG	POS	POS	POS	POS	NEG	NEG	NEG	NEG	NEG	NEG	NEG	NEG	NEG	NEG
2013_PA22_Z4005	NEG	NEG	NEG	NEG	NEG	NEG	NEG	NEG	NEG	POS	POS	POS	POS	POS	POS	POS	NEG	AMB	POS	NEG	NEG	POS	POS	POS	POS	NEG	NEG	NEG	NEG	NEG	NEG	NEG	NEG	NEG	NEG
2013_PA24_Z4007	NEG	NEG	NEG	NEG	NEG	NEG	NEG	NEG	NEG	POS	POS	POS	POS	POS	POS	POS	NEG	NEG	POS	NEG	NEG	POS	POS	POS	POS	NEG	NEG	NEG	NEG	NEG	NEG	NEG	NEG	NEG	NEG
2013_PA31_Z4014	NEG	NEG	NEG	NEG	NEG	NEG	NEG	NEG	NEG	POS	POS	POS	POS	POS	POS	POS	NEG	NEG	POS	NEG	NEG	POS	POS	POS	POS	NEG	NEG	NEG	NEG	NEG	NEG	NEG	NEG	NEG	NEG
2013_PA35_Z4018	NEG	NEG	NEG	NEG	NEG	NEG	NEG	NEG	NEG	POS	POS	POS	POS	POS	POS	POS	NEG	NEG	POS	NEG	NEG	POS	POS	POS	POS	NEG	NEG	NEG	NEG	NEG	NEG	NEG	NEG	NEG	NEG
2013_PA81_Z4064	NEG	NEG	NEG	NEG	NEG	NEG	NEG	NEG	NEG	POS	POS	POS	POS	POS	POS	POS	NEG	NEG	POS	NEG	NEG	POS	POS	POS	POS	NEG	NEG	NEG	NEG	NEG	NEG	NEG	NEG	NEG	NEG
**ST239-MRSA-III+ccrA/B4**																																			
2012_254_Z3537 (3124A/12)	NEG	NEG	NEG	NEG	NEG	NEG	NEG	NEG	NEG	POS	POS	POS	POS	POS	POS	POS	NEG	NEG	POS	AMB	POS	POS	POS	POS	POS	NEG	NEG	NEG	NEG	NEG	NEG	NEG	NEG	NEG	NEG
2013_PA25_Z4008	NEG	NEG	NEG	NEG	NEG	NEG	NEG	NEG	NEG	POS	POS	POS	POS	POS	POS	POS	NEG	NEG	POS	POS	POS	POS	POS	POS	POS	NEG	NEG	NEG	NEG	NEG	NEG	NEG	NEG	NEG	NEG
**ST834-MSSA**																																			
2012_268_Z3555 (3693/12)	NEG	NEG	NEG	NEG	NEG	NEG	NEG	NEG	NEG	NEG	NEG	NEG	NEG	NEG	NEG	NEG	NEG	NEG	NEG	NEG	NEG	POS	POS	POS	NEG	NEG	NEG	NEG	NEG	NEG	NEG	NEG	NEG	NEG	NEG
**CC12-MSSA**																																			
2012_210_Z3470 (1177/12)	NEG	NEG	NEG	NEG	NEG	NEG	NEG	NEG	NEG	NEG	NEG	NEG	NEG	NEG	NEG	NEG	NEG	NEG	NEG	NEG	NEG	POS	POS	POS	NEG	NEG	NEG	NEG	NEG	NEG	NEG	NEG	NEG	NEG	NEG
2012_230_Z3487 (1797/12)	NEG	NEG	NEG	NEG	NEG	NEG	NEG	NEG	NEG	NEG	NEG	NEG	NEG	NEG	NEG	NEG	NEG	NEG	NEG	NEG	NEG	NEG	NEG	NEG	NEG	NEG	NEG	NEG	NEG	NEG	NEG	NEG	NEG	NEG	NEG
2013_PA18_Z4001	NEG	NEG	NEG	NEG	NEG	NEG	NEG	NEG	NEG	NEG	NEG	NEG	NEG	NEG	NEG	NEG	NEG	NEG	NEG	NEG	NEG	NEG	NEG	NEG	NEG	NEG	NEG	NEG	NEG	NEG	NEG	NEG	NEG	NEG	NEG
**CC15-MSSA**																																			
2012_160_Z3444 (26386/11)	NEG	NEG	NEG	NEG	NEG	NEG	NEG	NEG	NEG	NEG	NEG	NEG	NEG	NEG	NEG	NEG	NEG	NEG	NEG	NEG	NEG	POS	POS	POS	NEG	NEG	NEG	NEG	NEG	NEG	NEG	NEG	NEG	NEG	NEG
2012_167_Z3514 (27010/11)	NEG	NEG	NEG	NEG	NEG	NEG	NEG	NEG	NEG	NEG	NEG	NEG	NEG	NEG	NEG	NEG	NEG	NEG	NEG	NEG	NEG	NEG	NEG	NEG	NEG	NEG	NEG	NEG	NEG	NEG	NEG	NEG	NEG	NEG	NEG
2012_184_Z3455 (376/12)	NEG	NEG	NEG	NEG	NEG	NEG	NEG	NEG	NEG	NEG	NEG	NEG	NEG	NEG	NEG	NEG	NEG	NEG	NEG	NEG	NEG	POS	POS	POS	NEG	NEG	NEG	NEG	NEG	NEG	NEG	NEG	NEG	NEG	NEG
2012_205_Z3465 (939/12)	NEG	NEG	NEG	NEG	NEG	NEG	NEG	NEG	NEG	NEG	NEG	NEG	NEG	NEG	NEG	NEG	NEG	NEG	NEG	NEG	NEG	POS	POS	POS	NEG	NEG	NEG	NEG	NEG	NEG	NEG	NEG	NEG	NEG	NEG
2012_231_Z3519 (1831/12)	NEG	NEG	NEG	NEG	NEG	NEG	NEG	NEG	NEG	NEG	NEG	NEG	NEG	NEG	NEG	NEG	NEG	NEG	NEG	NEG	NEG	POS	POS	POS	NEG	NEG	NEG	NEG	NEG	NEG	NEG	NEG	NEG	NEG	NEG
2012_249_Z3533 (2907/12)	NEG	NEG	NEG	NEG	NEG	NEG	NEG	NEG	NEG	NEG	NEG	NEG	NEG	NEG	NEG	NEG	NEG	NEG	NEG	NEG	NEG	NEG	NEG	NEG	NEG	NEG	NEG	NEG	NEG	NEG	NEG	NEG	NEG	NEG	NEG
2012_305_Z3592 (5839/12)	NEG	NEG	NEG	NEG	NEG	NEG	NEG	NEG	NEG	NEG	NEG	NEG	NEG	NEG	NEG	NEG	NEG	NEG	NEG	NEG	NEG	POS	POS	POS	NEG	NEG	NEG	NEG	NEG	NEG	AMB	NEG	NEG	NEG	NEG
2012_313_Z3600 (6857/12)	NEG	NEG	NEG	NEG	NEG	NEG	NEG	NEG	NEG	NEG	NEG	NEG	NEG	NEG	NEG	NEG	NEG	NEG	NEG	NEG	NEG	POS	POS	POS	NEG	NEG	NEG	NEG	NEG	NEG	NEG	NEG	NEG	NEG	NEG
2012_345_Z3632 (79668/12)	NEG	NEG	NEG	NEG	NEG	NEG	NEG	NEG	NEG	NEG	NEG	NEG	NEG	NEG	NEG	NEG	NEG	NEG	NEG	NEG	NEG	POS	POS	POS	NEG	NEG	NEG	NEG	NEG	NEG	NEG	NEG	NEG	NEG	NEG
2013_PA33_Z4016	NEG	NEG	NEG	NEG	NEG	NEG	NEG	NEG	NEG	NEG	NEG	NEG	NEG	NEG	NEG	NEG	NEG	NEG	NEG	NEG	NEG	POS	POS	POS	NEG	NEG	NEG	NEG	NEG	NEG	NEG	NEG	NEG	NEG	NEG
**CC20-MSSA**																																			
2012_240_Z3526 (2354/12)	NEG	NEG	NEG	NEG	NEG	NEG	NEG	NEG	NEG	NEG	NEG	NEG	NEG	NEG	NEG	NEG	NEG	NEG	NEG	NEG	NEG	POS	POS	POS	NEG	NEG	NEG	NEG	NEG	NEG	NEG	NEG	NEG	NEG	NEG
**CC30-MSSA**																																			
2012_155_Z3488 (25905A/11)	NEG	NEG	NEG	NEG	NEG	NEG	NEG	NEG	NEG	NEG	NEG	NEG	NEG	NEG	NEG	NEG	NEG	NEG	NEG	NEG	NEG	POS	POS	POS	NEG	NEG	NEG	NEG	NEG	NEG	NEG	NEG	NEG	NEG	NEG
**CC30-MSSA [PVL+]**																																			
2012_164_Z3447 (26574/11)	NEG	NEG	NEG	NEG	NEG	NEG	NEG	NEG	NEG	NEG	NEG	NEG	NEG	NEG	NEG	NEG	NEG	NEG	NEG	NEG	NEG	POS	POS	POS	NEG	NEG	NEG	NEG	NEG	NEG	NEG	NEG	NEG	NEG	NEG
2012_166_Z3493 (26988A/11)	NEG	NEG	NEG	NEG	NEG	NEG	NEG	NEG	NEG	NEG	NEG	NEG	NEG	NEG	NEG	NEG	NEG	NEG	NEG	NEG	NEG	POS	POS	POS	NEG	NEG	NEG	NEG	NEG	NEG	NEG	NEG	NEG	NEG	NEG
2012_178_Z3500 (197/12)	NEG	NEG	NEG	NEG	NEG	NEG	NEG	NEG	NEG	NEG	NEG	NEG	NEG	NEG	NEG	NEG	NEG	NEG	NEG	NEG	NEG	POS	POS	POS	NEG	NEG	NEG	NEG	NEG	NEG	NEG	NEG	NEG	NEG	NEG
2012_183_Z3454	NEG	NEG	NEG	NEG	NEG	NEG	NEG	NEG	NEG	NEG	NEG	NEG	NEG	NEG	NEG	NEG	NEG	NEG	NEG	NEG	NEG	POS	POS	POS	NEG	NEG	NEG	NEG	NEG	NEG	NEG	NEG	NEG	NEG	NEG
2012_186_Z3503 (444/12)	NEG	NEG	NEG	NEG	NEG	NEG	NEG	NEG	NEG	NEG	NEG	NEG	NEG	NEG	NEG	NEG	NEG	NEG	NEG	NEG	NEG	POS	POS	POS	NEG	NEG	NEG	NEG	NEG	NEG	NEG	NEG	NEG	NEG	NEG
2012_189_Z3506 (542/12)	NEG	NEG	NEG	NEG	NEG	NEG	NEG	NEG	NEG	NEG	NEG	NEG	NEG	NEG	NEG	NEG	NEG	NEG	NEG	NEG	NEG	POS	POS	POS	NEG	NEG	NEG	NEG	NEG	NEG	NEG	NEG	NEG	NEG	NEG
2012_192_Z3457 (606/12)	NEG	NEG	NEG	NEG	NEG	NEG	NEG	NEG	NEG	NEG	NEG	NEG	NEG	NEG	NEG	NEG	NEG	NEG	NEG	NEG	NEG	POS	POS	POS	NEG	NEG	NEG	NEG	NEG	NEG	NEG	NEG	NEG	NEG	NEG
2012_196_Z3509 (707/12)	NEG	NEG	NEG	NEG	NEG	NEG	NEG	NEG	NEG	NEG	NEG	NEG	NEG	NEG	NEG	NEG	NEG	NEG	NEG	NEG	NEG	POS	POS	POS	NEG	NEG	NEG	NEG	NEG	NEG	NEG	NEG	NEG	NEG	NEG
2012_218_Z3476 (1432/12)	NEG	NEG	NEG	NEG	NEG	NEG	NEG	NEG	NEG	NEG	NEG	NEG	NEG	NEG	NEG	NEG	NEG	NEG	NEG	NEG	NEG	POS	POS	POS	NEG	NEG	NEG	NEG	NEG	NEG	NEG	NEG	NEG	NEG	NEG
2012_232_Z3520 (1892/12)	NEG	NEG	NEG	NEG	NEG	NEG	NEG	NEG	NEG	NEG	NEG	NEG	NEG	NEG	NEG	NEG	NEG	NEG	NEG	NEG	NEG	POS	POS	POS	NEG	NEG	NEG	NEG	NEG	NEG	NEG	NEG	NEG	NEG	NEG
2012_278_Z3565 (4397/12)	NEG	NEG	NEG	NEG	NEG	NEG	NEG	NEG	NEG	NEG	NEG	NEG	NEG	NEG	NEG	NEG	NEG	NEG	NEG	NEG	NEG	POS	POS	POS	NEG	NEG	NEG	NEG	NEG	NEG	NEG	NEG	NEG	NEG	NEG
2012_285_Z3572 (4802/12)	NEG	NEG	NEG	NEG	NEG	NEG	NEG	NEG	NEG	NEG	NEG	NEG	NEG	NEG	NEG	NEG	NEG	NEG	NEG	NEG	NEG	POS	POS	POS	NEG	NEG	NEG	NEG	NEG	NEG	NEG	NEG	NEG	NEG	NEG
2012_287_Z3574 (4906/12)	NEG	NEG	NEG	NEG	NEG	NEG	NEG	NEG	NEG	NEG	NEG	NEG	NEG	NEG	NEG	NEG	NEG	NEG	NEG	NEG	NEG	POS	POS	POS	NEG	NEG	NEG	NEG	NEG	NEG	NEG	NEG	NEG	NEG	NEG
2012_295_Z3582 (5116/12)	NEG	NEG	NEG	NEG	NEG	NEG	NEG	NEG	NEG	NEG	NEG	NEG	NEG	NEG	NEG	NEG	NEG	NEG	NEG	NEG	NEG	NEG	NEG	NEG	NEG	NEG	NEG	NEG	NEG	NEG	NEG	NEG	NEG	NEG	NEG
2012_326_Z3613 (7321/12)	NEG	NEG	NEG	NEG	NEG	NEG	NEG	NEG	NEG	NEG	NEG	NEG	NEG	NEG	NEG	NEG	NEG	NEG	NEG	NEG	NEG	POS	POS	POS	NEG	NEG	NEG	NEG	NEG	NEG	NEG	NEG	NEG	NEG	NEG
2012_356_Z3643 (1204-15897)	NEG	NEG	NEG	NEG	NEG	NEG	NEG	NEG	NEG	NEG	NEG	NEG	NEG	NEG	NEG	NEG	NEG	NEG	NEG	NEG	NEG	POS	POS	POS	NEG	NEG	NEG	NEG	NEG	NEG	NEG	NEG	NEG	NEG	NEG
2012_366_Z3426 (1204-16124)	NEG	NEG	NEG	NEG	NEG	NEG	NEG	NEG	NEG	NEG	NEG	NEG	NEG	NEG	NEG	NEG	NEG	NEG	NEG	NEG	NEG	POS	POS	POS	NEG	NEG	NEG	NEG	NEG	NEG	NEG	NEG	NEG	NEG	NEG
2012_372_Z3432 (1205-19821)	NEG	NEG	NEG	NEG	NEG	NEG	NEG	NEG	NEG	NEG	NEG	NEG	NEG	NEG	NEG	NEG	NEG	NEG	NEG	NEG	NEG	POS	POS	POS	NEG	NEG	NEG	NEG	NEG	NEG	NEG	NEG	NEG	NEG	NEG
2012_380_Z3440 (4141)	NEG	NEG	NEG	NEG	NEG	NEG	NEG	NEG	NEG	NEG	NEG	NEG	NEG	NEG	NEG	NEG	NEG	NEG	NEG	NEG	NEG	POS	POS	POS	NEG	NEG	NEG	NEG	NEG	NEG	NEG	NEG	NEG	NEG	NEG
2013_PA20_Z4003	NEG	NEG	NEG	NEG	NEG	NEG	NEG	NEG	NEG	NEG	NEG	NEG	NEG	NEG	NEG	NEG	NEG	NEG	NEG	NEG	NEG	POS	POS	POS	NEG	NEG	NEG	NEG	NEG	NEG	NEG	NEG	NEG	NEG	NEG
2013_PA36_Z4019	NEG	NEG	NEG	NEG	NEG	NEG	NEG	NEG	NEG	NEG	NEG	NEG	NEG	NEG	NEG	NEG	NEG	NEG	NEG	NEG	NEG	POS	POS	POS	NEG	NEG	NEG	NEG	NEG	NEG	NEG	NEG	NEG	NEG	NEG
2013_PA46_Z4029	NEG	NEG	NEG	NEG	NEG	NEG	NEG	NEG	NEG	NEG	NEG	NEG	NEG	NEG	NEG	NEG	NEG	NEG	NEG	NEG	NEG	POS	POS	POS	NEG	NEG	NEG	NEG	NEG	NEG	NEG	NEG	NEG	NEG	NEG
2013_PA50_Z4033	NEG	NEG	NEG	NEG	NEG	NEG	NEG	NEG	NEG	NEG	NEG	NEG	NEG	NEG	NEG	NEG	NEG	NEG	NEG	NEG	NEG	POS	POS	POS	NEG	NEG	NEG	NEG	NEG	NEG	NEG	NEG	NEG	NEG	NEG
2013_PA63_Z4046	NEG	NEG	NEG	NEG	NEG	NEG	NEG	NEG	NEG	NEG	NEG	NEG	NEG	NEG	NEG	NEG	NEG	NEG	NEG	NEG	NEG	NEG	NEG	NEG	NEG	NEG	NEG	NEG	NEG	NEG	NEG	NEG	NEG	NEG	NEG
2013_PA78_Z4061	NEG	NEG	NEG	NEG	NEG	NEG	NEG	NEG	NEG	NEG	NEG	NEG	NEG	NEG	NEG	NEG	NEG	NEG	NEG	NEG	NEG	POS	POS	POS	NEG	NEG	NEG	NEG	NEG	NEG	NEG	NEG	NEG	NEG	NEG
2013_PA79_Z4062	NEG	NEG	NEG	NEG	NEG	NEG	NEG	NEG	NEG	NEG	NEG	NEG	NEG	NEG	NEG	NEG	NEG	NEG	NEG	NEG	NEG	NEG	NEG	NEG	NEG	NEG	NEG	NEG	NEG	NEG	NEG	NEG	NEG	NEG	NEG
**CC45-MSSA**																																			
2012_172_Z3449 (49/12)	NEG	NEG	NEG	NEG	NEG	NEG	NEG	NEG	NEG	NEG	NEG	NEG	NEG	NEG	NEG	NEG	NEG	NEG	NEG	NEG	NEG	POS	POS	POS	NEG	NEG	NEG	NEG	NEG	NEG	NEG	NEG	NEG	NEG	NEG
2012_208_Z3468 (1011/12)	NEG	NEG	NEG	NEG	NEG	NEG	NEG	NEG	NEG	NEG	NEG	NEG	NEG	NEG	NEG	NEG	NEG	NEG	NEG	NEG	NEG	POS	POS	POS	NEG	NEG	NEG	NEG	NEG	NEG	NEG	NEG	NEG	NEG	NEG
2012_235_Z3522 (2085/12)	NEG	NEG	NEG	NEG	NEG	NEG	NEG	NEG	NEG	NEG	NEG	NEG	NEG	NEG	NEG	NEG	NEG	NEG	NEG	NEG	NEG	POS	POS	POS	NEG	NEG	NEG	NEG	NEG	NEG	NEG	NEG	NEG	NEG	NEG
2012_290_Z3577 (4921/12)	NEG	NEG	NEG	NEG	NEG	NEG	NEG	NEG	NEG	NEG	NEG	NEG	NEG	NEG	NEG	NEG	NEG	NEG	NEG	NEG	NEG	POS	POS	POS	NEG	NEG	NEG	NEG	NEG	NEG	NEG	NEG	NEG	NEG	NEG
2012_311_Z3598 (6182/12)	NEG	NEG	NEG	NEG	NEG	NEG	NEG	NEG	NEG	NEG	NEG	NEG	NEG	NEG	NEG	NEG	NEG	NEG	NEG	NEG	NEG	POS	POS	POS	NEG	NEG	NEG	NEG	NEG	NEG	NEG	NEG	NEG	NEG	NEG
Z3604_TT317 - (DC558C18-2959-4049-B8F3-507365	NEG	NEG	NEG	NEG	NEG	NEG	NEG	NEG	NEG	NEG	NEG	NEG	NEG	NEG	NEG	NEG	NEG	NEG	NEG	NEG	NEG	POS	POS	POS	NEG	NEG	NEG	NEG	NEG	NEG	NEG	NEG	NEG	NEG	NEG
2012_363_Z3423 (1112-61058)	NEG	NEG	NEG	NEG	NEG	NEG	NEG	NEG	NEG	NEG	NEG	NEG	NEG	NEG	NEG	NEG	NEG	NEG	NEG	NEG	NEG	POS	POS	POS	NEG	NEG	NEG	NEG	NEG	NEG	NEG	NEG	NEG	NEG	NEG
2013_PA19_Z4002	NEG	NEG	NEG	NEG	NEG	NEG	NEG	NEG	NEG	NEG	NEG	NEG	NEG	NEG	NEG	NEG	NEG	NEG	NEG	NEG	NEG	POS	POS	POS	NEG	NEG	NEG	NEG	NEG	NEG	NEG	NEG	NEG	NEG	NEG
2013_PA54_Z4037	NEG	NEG	NEG	NEG	NEG	NEG	NEG	NEG	NEG	NEG	NEG	NEG	NEG	NEG	NEG	NEG	NEG	NEG	NEG	NEG	NEG	NEG	NEG	NEG	NEG	NEG	NEG	NEG	NEG	NEG	NEG	NEG	NEG	NEG	NEG
2013_PA82_Z4065	NEG	NEG	NEG	NEG	NEG	NEG	NEG	NEG	NEG	NEG	NEG	NEG	NEG	NEG	NEG	NEG	NEG	NEG	NEG	NEG	NEG	POS	POS	POS	NEG	NEG	NEG	NEG	NEG	NEG	NEG	NEG	NEG	NEG	NEG
**CC97-MSSA**																																			
2012_182_Z3453 (279A/12)	NEG	NEG	NEG	NEG	NEG	NEG	NEG	NEG	NEG	NEG	NEG	NEG	NEG	NEG	NEG	NEG	NEG	NEG	NEG	NEG	NEG	NEG	NEG	NEG	NEG	NEG	NEG	NEG	NEG	NEG	NEG	NEG	NEG	NEG	NEG
2012_187_Z3504	NEG	NEG	NEG	NEG	NEG	NEG	NEG	NEG	NEG	NEG	NEG	NEG	NEG	NEG	NEG	NEG	NEG	NEG	NEG	NEG	NEG	NEG	NEG	NEG	NEG	NEG	NEG	NEG	NEG	NEG	NEG	NEG	NEG	NEG	NEG
2012_197_Z3510 (757/12)	NEG	NEG	NEG	NEG	NEG	NEG	NEG	NEG	NEG	NEG	NEG	NEG	NEG	NEG	NEG	NEG	NEG	NEG	NEG	NEG	NEG	POS	POS	POS	NEG	NEG	NEG	NEG	NEG	NEG	NEG	NEG	NEG	NEG	NEG
2012_199_Z3516 (785/12)	NEG	NEG	NEG	NEG	NEG	NEG	NEG	NEG	NEG	NEG	NEG	NEG	NEG	NEG	NEG	NEG	NEG	NEG	NEG	NEG	NEG	POS	POS	POS	NEG	NEG	NEG	NEG	NEG	NEG	NEG	NEG	NEG	NEG	NEG
2013_PA15_Z3998	NEG	NEG	NEG	NEG	NEG	NEG	NEG	NEG	NEG	NEG	NEG	NEG	NEG	NEG	NEG	NEG	NEG	NEG	NEG	NEG	NEG	POS	POS	POS	NEG	NEG	NEG	NEG	NEG	NEG	NEG	NEG	NEG	NEG	NEG
**CC101-MSSA**																																			
2012_283_Z3570 (4725/12)	NEG	NEG	NEG	NEG	NEG	NEG	NEG	NEG	NEG	NEG	NEG	NEG	NEG	NEG	NEG	NEG	NEG	NEG	NEG	NEG	NEG	NEG	NEG	NEG	NEG	NEG	NEG	NEG	NEG	NEG	NEG	NEG	NEG	NEG	NEG
2012_247_Z3531 (2788/12)	NEG	NEG	NEG	NEG	NEG	NEG	NEG	NEG	NEG	NEG	NEG	NEG	NEG	NEG	NEG	NEG	NEG	NEG	NEG	NEG	NEG	NEG	NEG	NEG	NEG	NEG	NEG	NEG	NEG	NEG	NEG	NEG	NEG	NEG	NEG
2012_279_Z3566 (4506/12)	NEG	NEG	NEG	NEG	NEG	NEG	NEG	NEG	NEG	NEG	NEG	NEG	NEG	NEG	NEG	NEG	NEG	NEG	NEG	NEG	NEG	NEG	NEG	NEG	NEG	NEG	NEG	NEG	NEG	NEG	NEG	NEG	NEG	NEG	NEG

Strain / Isolates	plsSCC (COL)	Q9XB68-dcs	ccrA-2	ccrB-2	kdpA-SCC	kdpB-SCC	kdpC-SCC	kdpD-SCC	kdpE-SCC	mecI	mecR	xylR	ccrA-3	ccrB-3	merA	merB	ccrAA (MRSAZH47) probe 1	ccrAA (MRSAZH47) probe 2	ccrC (85-2082)	ccrA-4	ccrB-4	blaZ	blaI	blaR	erm(A)	erm(B)	erm(C)	lnu(A)	msr(A)	mef(A)	mph(C)	vat(A)	vat(B)	vga(A) (BM 3327)	vgB(A)
	plasmin-sensitive surface protein	hypothetical protein from SCCmec elements	cassette chromosome recombinase genes A/B-2		potassium-trans-locating ATPase A, chain 2	potassium-transporting ATPase B, chain 1	potassium-trans-locating ATPase C, chain 2	sensor kinase protein	KDP operon transcrip-tional regulatory protein	methicillin-resistance regulatory protein	signal transducer protein MecR1	homolog of xylose repressor, associated with SCCmec-elements	cassette chromosome recombinase genes A/B-3		mercury resistance operon		cassette chromosome recombinase genes "ccrAA" (hypothetical) and ccrC					beta-lactamase	beta lactamase repressor (inhibitor)	beta-lactamase regulatory protein	erythromycin/ clindamycin resistance	erythromycin/ clindamycin resistance	erythromycin/ clindamycin resistance	Lincosamid-Nucleo-tidyltrans-ferase	energy-dependent efflux of erythro-mycin	macrolide efflux protein A	probable lysylphos-phatidyl-glycerol synthetase	virginiamycin A acetyl-transferase	acetyl-transferase inactivating streptogramin A	ATP binding protein, streptogramin-A-resistance	virginiamycin B hydrolase
**CC121-MSSA**																																			
2012_163_Z3446 (26495/11)	NEG	NEG	NEG	NEG	NEG	NEG	NEG	NEG	NEG	NEG	NEG	NEG	NEG	NEG	NEG	NEG	NEG	NEG	NEG	NEG	NEG	POS	POS	POS	NEG	NEG	NEG	NEG	NEG	NEG	NEG	NEG	NEG	NEG	NEG
2012_204_Z3464 (938/12)	NEG	NEG	NEG	NEG	NEG	NEG	NEG	NEG	NEG	NEG	NEG	NEG	NEG	NEG	NEG	NEG	NEG	NEG	NEG	NEG	NEG	POS	POS	POS	NEG	NEG	NEG	NEG	NEG	NEG	NEG	NEG	NEG	NEG	NEG
2012_225_Z3482 (1563/12)	NEG	NEG	NEG	NEG	NEG	NEG	NEG	NEG	NEG	NEG	NEG	NEG	NEG	NEG	NEG	NEG	NEG	NEG	NEG	NEG	NEG	POS	POS	POS	NEG	NEG	NEG	NEG	NEG	NEG	NEG	NEG	NEG	NEG	NEG
2012_228_Z3485 (1700B/12)	NEG	NEG	NEG	NEG	NEG	NEG	NEG	NEG	NEG	NEG	NEG	NEG	NEG	NEG	NEG	NEG	NEG	NEG	NEG	NEG	NEG	POS	POS	POS	NEG	NEG	NEG	NEG	NEG	NEG	NEG	NEG	NEG	NEG	NEG
2012_229_Z3486 (1737/12)	NEG	NEG	NEG	NEG	NEG	NEG	NEG	NEG	NEG	NEG	NEG	NEG	NEG	NEG	NEG	NEG	NEG	NEG	NEG	NEG	NEG	POS	POS	POS	NEG	NEG	NEG	NEG	NEG	NEG	NEG	NEG	NEG	NEG	NEG
2012_302_Z3589 (5795/12)	NEG	NEG	NEG	NEG	NEG	NEG	NEG	NEG	NEG	NEG	NEG	NEG	NEG	NEG	NEG	NEG	NEG	NEG	NEG	NEG	NEG	POS	POS	POS	NEG	NEG	NEG	NEG	NEG	NEG	NEG	NEG	NEG	NEG	NEG
2012_359_Z3419	NEG	NEG	NEG	NEG	NEG	NEG	NEG	NEG	NEG	NEG	NEG	NEG	NEG	NEG	NEG	NEG	NEG	NEG	NEG	NEG	NEG	POS	POS	POS	NEG	NEG	NEG	NEG	NEG	NEG	NEG	NEG	NEG	NEG	NEG
2012_362_Z3422 (1201-996)	NEG	NEG	NEG	NEG	NEG	NEG	NEG	NEG	NEG	NEG	NEG	NEG	NEG	NEG	NEG	NEG	NEG	NEG	NEG	NEG	NEG	POS	POS	POS	NEG	NEG	NEG	NEG	NEG	NEG	NEG	NEG	NEG	NEG	NEG
2012_367_Z3427 (1204-16413)	NEG	NEG	NEG	NEG	NEG	NEG	NEG	NEG	NEG	NEG	NEG	NEG	NEG	NEG	NEG	NEG	NEG	NEG	NEG	NEG	NEG	POS	POS	POS	NEG	NEG	NEG	NEG	NEG	NEG	NEG	NEG	NEG	NEG	NEG
2013_PA09_Z3992	NEG	NEG	NEG	NEG	NEG	NEG	NEG	NEG	NEG	NEG	NEG	NEG	NEG	NEG	NEG	NEG	NEG	NEG	NEG	NEG	NEG	POS	POS	POS	NEG	NEG	NEG	NEG	NEG	NEG	NEG	NEG	NEG	NEG	NEG
2013_PA59_Z4042	NEG	NEG	NEG	NEG	NEG	NEG	NEG	NEG	NEG	NEG	NEG	NEG	NEG	NEG	NEG	NEG	NEG	NEG	NEG	NEG	NEG	POS	POS	POS	NEG	NEG	NEG	NEG	NEG	NEG	NEG	NEG	NEG	NEG	NEG
2013_PA60_Z4043	NEG	NEG	NEG	NEG	NEG	NEG	NEG	NEG	NEG	NEG	NEG	NEG	NEG	NEG	NEG	NEG	NEG	NEG	NEG	NEG	NEG	POS	POS	POS	NEG	NEG	NEG	NEG	NEG	NEG	NEG	NEG	NEG	NEG	NEG
**CC152-MSSA [PVL+]**																																			
2012_190_Z3507 (549/12)	NEG	NEG	NEG	NEG	NEG	NEG	NEG	NEG	NEG	NEG	NEG	NEG	NEG	NEG	NEG	NEG	NEG	NEG	NEG	NEG	NEG	POS	POS	POS	NEG	NEG	NEG	NEG	NEG	NEG	NEG	NEG	NEG	NEG	NEG
2012_201_Z3461 (823/12)	NEG	NEG	NEG	NEG	NEG	NEG	NEG	NEG	NEG	NEG	NEG	NEG	NEG	NEG	NEG	NEG	NEG	NEG	NEG	NEG	NEG	POS	POS	POS	NEG	NEG	NEG	NEG	NEG	NEG	NEG	AMB	NEG	NEG	NEG
2012_209_Z3469 (1116/12)	NEG	NEG	NEG	NEG	NEG	NEG	NEG	NEG	NEG	NEG	NEG	NEG	NEG	NEG	NEG	NEG	NEG	NEG	NEG	NEG	NEG	POS	POS	POS	NEG	NEG	NEG	NEG	NEG	NEG	NEG	NEG	NEG	NEG	NEG
2012_211_Z3517 (1238/12)	NEG	NEG	NEG	NEG	NEG	NEG	NEG	NEG	NEG	NEG	NEG	NEG	NEG	NEG	NEG	NEG	NEG	NEG	NEG	NEG	NEG	POS	POS	POS	NEG	NEG	NEG	NEG	NEG	NEG	NEG	NEG	NEG	NEG	NEG
2012_213_Z3472 (1320/12)	NEG	NEG	NEG	NEG	NEG	NEG	NEG	NEG	NEG	NEG	NEG	NEG	NEG	NEG	NEG	NEG	NEG	NEG	NEG	NEG	NEG	POS	POS	POS	NEG	NEG	NEG	NEG	NEG	NEG	NEG	NEG	NEG	NEG	NEG
2012_221_Z3478	NEG	NEG	NEG	NEG	NEG	NEG	NEG	NEG	NEG	NEG	NEG	NEG	NEG	NEG	NEG	NEG	NEG	NEG	NEG	NEG	NEG	POS	POS	POS	NEG	NEG	NEG	NEG	NEG	NEG	NEG	NEG	NEG	NEG	NEG
2012_234_Z3521 (1981/12)	NEG	NEG	NEG	NEG	NEG	NEG	NEG	NEG	NEG	NEG	NEG	NEG	NEG	NEG	NEG	NEG	NEG	NEG	NEG	NEG	NEG	POS	POS	POS	NEG	NEG	NEG	NEG	NEG	NEG	NEG	NEG	NEG	NEG	NEG
2012_238_Z3524 (2199/12)	NEG	NEG	NEG	NEG	NEG	NEG	NEG	NEG	NEG	NEG	NEG	NEG	NEG	NEG	NEG	NEG	NEG	NEG	NEG	NEG	NEG	POS	POS	POS	NEG	NEG	NEG	NEG	NEG	NEG	NEG	NEG	NEG	NEG	NEG
2012_260_Z3547 (3290/12)	NEG	NEG	NEG	NEG	NEG	NEG	NEG	NEG	NEG	NEG	NEG	NEG	NEG	NEG	NEG	NEG	NEG	NEG	NEG	NEG	NEG	POS	POS	POS	NEG	NEG	NEG	NEG	NEG	NEG	NEG	NEG	NEG	NEG	NEG
2012_272_Z3559 (3802/12)	NEG	NEG	NEG	NEG	NEG	NEG	NEG	NEG	NEG	NEG	NEG	NEG	NEG	NEG	NEG	NEG	NEG	NEG	NEG	NEG	NEG	POS	POS	POS	NEG	NEG	NEG	NEG	NEG	NEG	NEG	NEG	NEG	NEG	NEG
2012_289_Z3576 (4912/12)	NEG	NEG	NEG	NEG	NEG	NEG	NEG	NEG	NEG	NEG	NEG	NEG	NEG	NEG	NEG	NEG	NEG	NEG	NEG	NEG	NEG	POS	POS	POS	NEG	NEG	NEG	NEG	NEG	NEG	NEG	NEG	NEG	NEG	NEG
2012_309_Z3596	NEG	NEG	NEG	NEG	NEG	NEG	NEG	NEG	NEG	NEG	NEG	NEG	NEG	NEG	NEG	NEG	NEG	NEG	NEG	NEG	NEG	POS	POS	POS	NEG	NEG	NEG	NEG	NEG	NEG	NEG	NEG	NEG	NEG	NEG
2012_322_Z3609 (7178/12)	NEG	NEG	NEG	NEG	NEG	NEG	NEG	NEG	NEG	NEG	NEG	NEG	NEG	NEG	NEG	NEG	NEG	NEG	NEG	NEG	NEG	POS	POS	POS	NEG	NEG	NEG	NEG	NEG	NEG	NEG	NEG	NEG	NEG	NEG
2012_325_Z3612 (7254/12)	NEG	NEG	NEG	NEG	NEG	NEG	NEG	NEG	NEG	NEG	NEG	NEG	NEG	NEG	NEG	NEG	NEG	NEG	NEG	NEG	NEG	POS	POS	POS	NEG	NEG	NEG	NEG	NEG	NEG	NEG	NEG	NEG	NEG	NEG
2012_328_Z3615 (7456/12)	NEG	NEG	NEG	NEG	NEG	NEG	NEG	NEG	NEG	NEG	NEG	NEG	NEG	NEG	NEG	NEG	NEG	NEG	NEG	NEG	NEG	POS	POS	POS	NEG	NEG	NEG	NEG	NEG	NEG	NEG	NEG	NEG	NEG	NEG
2012_334_Z3621 (8267/12)	NEG	NEG	NEG	NEG	NEG	NEG	NEG	NEG	NEG	NEG	NEG	NEG	NEG	NEG	NEG	NEG	NEG	NEG	NEG	NEG	NEG	NEG	NEG	NEG	NEG	NEG	NEG	NEG	NEG	NEG	NEG	NEG	NEG	NEG	NEG
2012_360_Z3420 (1201-4047)	NEG	NEG	NEG	NEG	NEG	NEG	NEG	NEG	NEG	NEG	NEG	NEG	NEG	NEG	NEG	NEG	NEG	NEG	NEG	NEG	NEG	POS	POS	POS	NEG	NEG	NEG	NEG	NEG	NEG	NEG	NEG	NEG	NEG	NEG
2012_382_Z3442 (4086)	NEG	NEG	NEG	NEG	NEG	NEG	NEG	NEG	NEG	NEG	NEG	NEG	NEG	NEG	NEG	NEG	NEG	NEG	NEG	NEG	NEG	POS	POS	POS	NEG	NEG	NEG	NEG	NEG	NEG	NEG	NEG	NEG	NEG	NEG
2013_PA02_Z3985	NEG	NEG	NEG	NEG	NEG	NEG	NEG	NEG	NEG	NEG	NEG	NEG	NEG	NEG	NEG	NEG	NEG	NEG	NEG	NEG	NEG	POS	POS	POS	NEG	NEG	NEG	NEG	NEG	NEG	NEG	NEG	NEG	NEG	NEG
2013_PA03_Z3986	NEG	NEG	NEG	NEG	NEG	NEG	NEG	NEG	NEG	NEG	NEG	NEG	NEG	NEG	NEG	NEG	NEG	NEG	NEG	NEG	NEG	POS	POS	POS	NEG	NEG	NEG	NEG	NEG	NEG	NEG	NEG	NEG	NEG	NEG
2013_PA07_Z3990	NEG	NEG	NEG	NEG	NEG	NEG	NEG	NEG	NEG	NEG	NEG	NEG	NEG	NEG	NEG	NEG	NEG	NEG	NEG	NEG	NEG	POS	POS	POS	NEG	NEG	NEG	NEG	NEG	NEG	NEG	NEG	NEG	NEG	NEG
2013_PA34_Z4017	NEG	NEG	NEG	NEG	NEG	NEG	NEG	NEG	NEG	NEG	NEG	NEG	NEG	NEG	NEG	NEG	NEG	NEG	NEG	NEG	NEG	POS	POS	POS	NEG	NEG	NEG	NEG	NEG	NEG	NEG	NEG	NEG	NEG	NEG
2013_PA40_Z4023	NEG	NEG	NEG	NEG	NEG	NEG	NEG	NEG	NEG	NEG	NEG	NEG	NEG	NEG	NEG	NEG	NEG	NEG	NEG	NEG	NEG	POS	POS	POS	NEG	NEG	NEG	NEG	NEG	NEG	NEG	NEG	NEG	NEG	NEG
2013_PA48_Z4031	NEG	NEG	NEG	NEG	NEG	NEG	NEG	NEG	NEG	NEG	NEG	NEG	NEG	NEG	NEG	NEG	NEG	NEG	NEG	NEG	NEG	POS	POS	POS	NEG	NEG	NEG	NEG	NEG	NEG	NEG	NEG	NEG	NEG	NEG
2013_PA49_Z4032	NEG	NEG	NEG	NEG	NEG	NEG	NEG	NEG	NEG	NEG	NEG	NEG	NEG	NEG	NEG	NEG	NEG	NEG	NEG	NEG	NEG	POS	POS	POS	NEG	NEG	NEG	NEG	NEG	NEG	NEG	NEG	NEG	NEG	NEG
2013_PA51_Z4034	NEG	NEG	NEG	NEG	NEG	NEG	NEG	NEG	NEG	NEG	NEG	NEG	NEG	NEG	NEG	NEG	NEG	NEG	NEG	NEG	NEG	POS	POS	POS	NEG	NEG	NEG	NEG	NEG	NEG	NEG	NEG	NEG	NEG	NEG
2013_PA70_Z4053	NEG	NEG	NEG	NEG	NEG	NEG	NEG	NEG	NEG	NEG	NEG	NEG	NEG	NEG	NEG	NEG	NEG	NEG	NEG	NEG	NEG	POS	POS	POS	NEG	NEG	NEG	NEG	NEG	NEG	NEG	NEG	NEG	NEG	NEG
2013_PA84_Z4067	NEG	NEG	NEG	NEG	NEG	NEG	NEG	NEG	NEG	NEG	NEG	NEG	NEG	NEG	NEG	NEG	NEG	NEG	NEG	NEG	NEG	POS	POS	POS	NEG	NEG	NEG	NEG	NEG	NEG	NEG	NEG	NEG	NEG	NEG
**CC188-MSSA**																																			
2012_206_Z3466 (968/12)	NEG	NEG	NEG	NEG	NEG	NEG	NEG	NEG	NEG	NEG	NEG	NEG	NEG	NEG	NEG	NEG	NEG	NEG	NEG	NEG	NEG	POS	POS	POS	NEG	NEG	NEG	NEG	NEG	NEG	NEG	NEG	NEG	NEG	NEG
2012_251_Z3534 (3026/12)	NEG	NEG	NEG	NEG	NEG	NEG	NEG	NEG	NEG	NEG	NEG	NEG	NEG	NEG	NEG	NEG	NEG	NEG	NEG	NEG	NEG	POS	POS	POS	NEG	NEG	NEG	NEG	NEG	NEG	NEG	NEG	NEG	NEG	NEG
2012_320_Z3607 (7144/12)	NEG	NEG	NEG	NEG	NEG	NEG	NEG	NEG	NEG	NEG	NEG	NEG	NEG	NEG	NEG	NEG	NEG	NEG	NEG	NEG	NEG	POS	POS	POS	NEG	NEG	NEG	NEG	NEG	NEG	NEG	NEG	NEG	NEG	NEG
2012_321_Z3608 (7147/12)	NEG	NEG	NEG	NEG	NEG	NEG	NEG	NEG	NEG	NEG	NEG	NEG	NEG	NEG	NEG	NEG	NEG	NEG	NEG	NEG	NEG	POS	POS	AMB	NEG	NEG	NEG	NEG	NEG	NEG	NEG	NEG	NEG	NEG	NEG
2012_358_Z3418 (1202-6332)	NEG	NEG	NEG	NEG	NEG	NEG	NEG	NEG	NEG	NEG	NEG	NEG	NEG	NEG	NEG	NEG	NEG	NEG	NEG	NEG	NEG	NEG	NEG	NEG	NEG	NEG	NEG	NEG	NEG	NEG	NEG	NEG	NEG	NEG	NEG
**CC398-MSSA**																																			
2013_PA11_Z3994	NEG	NEG	NEG	NEG	NEG	NEG	NEG	NEG	NEG	NEG	NEG	NEG	NEG	NEG	NEG	NEG	NEG	NEG	NEG	NEG	NEG	NEG	NEG	NEG	NEG	NEG	NEG	NEG	NEG	NEG	NEG	NEG	NEG	NEG	NEG
2013_PA57_Z4040	NEG	NEG	NEG	NEG	NEG	NEG	NEG	NEG	NEG	NEG	NEG	NEG	NEG	NEG	NEG	NEG	NEG	NEG	NEG	NEG	NEG	POS	POS	POS	NEG	NEG	NEG	NEG	NEG	NEG	NEG	NEG	NEG	NEG	NEG
**ST1852-MSSA**																																			
2012_277_Z3564 (4050/12)	NEG	NEG	NEG	NEG	NEG	NEG	NEG	NEG	NEG	NEG	NEG	NEG	NEG	NEG	NEG	NEG	NEG	NEG	NEG	NEG	NEG	NEG	NEG	NEG	NEG	NEG	NEG	NEG	NEG	NEG	NEG	NEG	NEG	NEG	NEG
**CC1223/1594-MSSA**																																			
2012_337_Z3624 (8070B/12)	NEG	NEG	NEG	NEG	NEG	NEG	NEG	NEG	NEG	NEG	NEG	NEG	NEG	NEG	NEG	NEG	NEG	NEG	NEG	NEG	NEG	POS	POS	POS	NEG	NEG	NEG	NEG	NEG	NEG	NEG	NEG	NEG	NEG	NEG
**MSSA related to ST2250/2277**																																			
2012_203_Z3463 (829/12)	NEG	NEG	NEG	NEG	NEG	NEG	NEG	NEG	NEG	NEG	NEG	NEG	NEG	NEG	NEG	NEG	NEG	NEG	NEG	NEG	NEG	POS	POS	POS	NEG	NEG	NEG	NEG	NEG	NEG	NEG	NEG	NEG	NEG	NEG
2012_269_Z3556 (3769A/12)	NEG	NEG	NEG	NEG	NEG	NEG	NEG	NEG	NEG	NEG	NEG	NEG	NEG	NEG	NEG	NEG	NEG	NEG	NEG	NEG	NEG	NEG	NEG	NEG	NEG	NEG	NEG	NEG	NEG	NEG	NEG	NEG	NEG	NEG	NEG
2012_292_Z3579	NEG	NEG	NEG	NEG	NEG	NEG	NEG	NEG	NEG	NEG	NEG	NEG	NEG	NEG	NEG	NEG	NEG	NEG	NEG	NEG	NEG	NEG	NEG	NEG	NEG	NEG	NEG	NEG	NEG	NEG	NEG	NEG	NEG	NEG	NEG
2012_299_Z3586 (5487/12)	NEG	NEG	NEG	NEG	NEG	NEG	NEG	NEG	NEG	NEG	NEG	NEG	NEG	NEG	NEG	NEG	NEG	NEG	NEG	NEG	NEG	NEG	NEG	NEG	NEG	NEG	NEG	NEG	NEG	NEG	NEG	NEG	NEG	NEG	NEG
2012_301_Z3588 (5717/12)	NEG	NEG	NEG	NEG	NEG	NEG	NEG	NEG	NEG	NEG	NEG	NEG	NEG	NEG	NEG	NEG	NEG	NEG	NEG	NEG	NEG	POS	POS	POS	NEG	NEG	NEG	NEG	NEG	NEG	NEG	NEG	NEG	NEG	NEG
**ST2596dlv-MSSA**																																			
2012_310_Z3597 (6034/12)	NEG	NEG	NEG	NEG	NEG	NEG	NEG	NEG	NEG	NEG	NEG	NEG	NEG	NEG	NEG	NEG	NEG	NEG	NEG	NEG	NEG	POS	POS	POS	NEG	NEG	NEG	NEG	NEG	NEG	NEG	NEG	NEG	NEG	NEG

Strain / Isolates	bifunctional enzyme Aac/Aph, gentamicin resistance	amino-glycoside adenyl-transferase, tobramycin resistance	3'5'-aminog-lycoside phospho-transferase, neo-/ kanamycin resistance	streptothricine-acetyl-transferase	dihydrofolate reductase type 1	fusidic acid resistence	hypothetical protein associated with fusidic acid resistance	mupirocin resistence protein	tetrazyklin-resistance	tetracycline-resistance	chloramphenicol acetyltransferase						chloramphenicol/florfenicol exporter	metallothiol transferase		quaternary ammonium compound resistance protein A	quaternary ammonium compound resistance protein C						Transport-/ Effluxprotein	vancomycin resistance gene	vancomycin resistance gene from enterococci and Clostridium	teicoplanin resistance gene from enterococci
	aacA-aphD	aadD	aphA3	sat	dfrS1	far1	fusC (Q6GD50)	mupA	tet(K)	tet(M)	cat (total)	cat (pC221)	cat (pc223)	cat (pMC524/pC194)	cat (pSBK203R)	cfr	fexA	fosB	fosB (plasmid)	qacA	qacC (total)	qacC (consensus)	qacC (equine)	qacC (SA5)	qacC (Ssap)	qacC (ST94)	sdrM	vanA	vanB	vanZ
**CC1-MSSA**																														
2012_257_Z3544 (3216/12)	NEG	NEG	NEG	NEG	NEG	NEG	NEG	NEG	NEG	NEG	NEG	NEG	NEG	NEG	NEG	NEG	NEG	NEG	NEG	NEG	NEG	NEG	NEG	NEG	NEG	NEG	POS	NEG	NEG	NEG
2012_281_Z3568 (4696/12)	NEG	NEG	NEG	NEG	NEG	NEG	NEG	NEG	NEG	POS	NEG	NEG	NEG	NEG	NEG	NEG	NEG	NEG	NEG	NEG	NEG	NEG	NEG	NEG	NEG	NEG	POS	NEG	NEG	NEG
2012_324_Z3611 (7180/12)	NEG	NEG	NEG	NEG	NEG	NEG	NEG	NEG	NEG	NEG	NEG	NEG	NEG	NEG	NEG	NEG	NEG	NEG	NEG	NEG	NEG	NEG	NEG	NEG	NEG	NEG	POS	NEG	NEG	NEG
2012_327_Z3614 (7347/12)	NEG	NEG	NEG	NEG	NEG	NEG	NEG	NEG	NEG	NEG	NEG	NEG	NEG	NEG	NEG	NEG	NEG	NEG	NEG	NEG	NEG	NEG	NEG	NEG	NEG	NEG	POS	NEG	NEG	NEG
2012_351_Z3638 (8273B/12)	NEG	NEG	NEG	NEG	NEG	NEG	NEG	NEG	NEG	NEG	NEG	NEG	NEG	NEG	NEG	NEG	NEG	NEG	NEG	NEG	NEG	NEG	NEG	NEG	NEG	NEG	POS	NEG	NEG	NEG
**CC5-MSSA**																														
2012_188_Z3505 (503/12)	NEG	NEG	NEG	NEG	NEG	NEG	NEG	NEG	NEG	NEG	NEG	NEG	NEG	NEG	NEG	NEG	NEG	POS	NEG	NEG	NEG	NEG	NEG	NEG	NEG	NEG	POS	NEG	NEG	NEG
2012_219_Z3518 (1437/12)	NEG	NEG	NEG	NEG	NEG	NEG	NEG	NEG	NEG	NEG	NEG	NEG	NEG	NEG	NEG	NEG	NEG	POS	AMB	NEG	NEG	NEG	NEG	NEG	NEG	NEG	POS	NEG	NEG	NEG
2012_329_Z3616 (7577/12)	NEG	NEG	POS	POS	NEG	NEG	NEG	NEG	NEG	NEG	NEG	NEG	NEG	NEG	NEG	NEG	NEG	POS	AMB	NEG	NEG	NEG	NEG	NEG	NEG	NEG	POS	NEG	NEG	NEG
2012_377_Z3437 (6242)	NEG	NEG	POS	POS	NEG	NEG	NEG	NEG	NEG	NEG	NEG	NEG	NEG	NEG	NEG	NEG	NEG	POS	NEG	NEG	NEG	NEG	NEG	NEG	NEG	NEG	POS	NEG	NEG	NEG
2013_PA04_Z3987	NEG	NEG	NEG	NEG	NEG	NEG	NEG	NEG	NEG	NEG	NEG	NEG	NEG	NEG	NEG	NEG	NEG	POS	NEG	NEG	NEG	NEG	NEG	NEG	NEG	NEG	POS	NEG	NEG	NEG
2013_PA32_Z4015	NEG	NEG	NEG	NEG	NEG	NEG	NEG	NEG	NEG	NEG	NEG	NEG	NEG	NEG	NEG	NEG	NEG	POS	NEG	NEG	NEG	NEG	NEG	NEG	NEG	NEG	POS	NEG	NEG	NEG
2013_PA41_Z4024	NEG	NEG	NEG	NEG	NEG	NEG	NEG	NEG	POS	NEG	NEG	NEG	NEG	NEG	NEG	NEG	NEG	POS	NEG	NEG	NEG	NEG	NEG	NEG	NEG	NEG	POS	NEG	NEG	NEG
2013_PA44_Z4027	NEG	NEG	NEG	NEG	NEG	NEG	NEG	NEG	POS	NEG	NEG	NEG	NEG	NEG	NEG	NEG	NEG	POS	NEG	NEG	NEG	NEG	NEG	NEG	NEG	NEG	POS	NEG	NEG	NEG
**CC5-MSSA [PVL+]**																														
2012_175_Z3497 (112/12)	NEG	NEG	NEG	NEG	NEG	NEG	NEG	NEG	NEG	NEG	NEG	NEG	NEG	NEG	NEG	NEG	NEG	POS	NEG	NEG	NEG	NEG	NEG	NEG	NEG	NEG	POS	NEG	NEG	NEG
2012_193_Z3458 (617/12)	NEG	NEG	NEG	NEG	NEG	NEG	NEG	NEG	NEG	NEG	NEG	NEG	NEG	NEG	NEG	NEG	NEG	POS	AMB	NEG	NEG	NEG	NEG	NEG	NEG	NEG	POS	NEG	NEG	NEG
2012_215_Z3474 (1341/12)	NEG	NEG	NEG	NEG	NEG	NEG	NEG	NEG	NEG	NEG	NEG	NEG	NEG	NEG	NEG	NEG	NEG	POS	NEG	NEG	NEG	NEG	NEG	NEG	NEG	NEG	POS	NEG	NEG	NEG
2012_216_Z3475 (1370/12)	NEG	NEG	NEG	NEG	NEG	NEG	NEG	NEG	NEG	NEG	NEG	NEG	NEG	NEG	NEG	NEG	NEG	POS	NEG	NEG	NEG	NEG	NEG	NEG	NEG	NEG	POS	NEG	NEG	NEG
2012_297_Z3584 (5140/12)	NEG	NEG	NEG	NEG	NEG	NEG	NEG	NEG	NEG	NEG	NEG	NEG	NEG	NEG	NEG	NEG	NEG	POS	NEG	NEG	NEG	NEG	NEG	NEG	NEG	NEG	POS	NEG	NEG	NEG
2012_331_Z3618 (7644/12)	NEG	NEG	NEG	NEG	NEG	NEG	NEG	NEG	NEG	NEG	NEG	NEG	NEG	NEG	NEG	NEG	NEG	POS	AMB	NEG	NEG	NEG	NEG	NEG	NEG	NEG	POS	NEG	NEG	NEG
2012_379_Z3439 (4126)	NEG	NEG	NEG	NEG	NEG	NEG	NEG	NEG	NEG	NEG	NEG	NEG	NEG	NEG	NEG	NEG	NEG	POS	NEG	NEG	NEG	NEG	NEG	NEG	NEG	NEG	POS	NEG	NEG	NEG
2012_381_Z3441	NEG	NEG	NEG	NEG	NEG	NEG	NEG	NEG	NEG	NEG	NEG	NEG	NEG	NEG	NEG	NEG	NEG	POS	AMB	NEG	NEG	NEG	NEG	NEG	NEG	NEG	POS	NEG	NEG	NEG
2013_PA39_Z4022	NEG	NEG	NEG	NEG	NEG	NEG	NEG	NEG	NEG	NEG	NEG	NEG	NEG	NEG	NEG	NEG	NEG	POS	NEG	NEG	NEG	NEG	NEG	NEG	NEG	NEG	POS	NEG	NEG	NEG
2013_PA47_Z4030	NEG	NEG	NEG	NEG	NEG	NEG	NEG	NEG	NEG	NEG	NEG	NEG	NEG	NEG	NEG	NEG	NEG	POS	NEG	NEG	NEG	NEG	NEG	NEG	NEG	NEG	POS	NEG	NEG	NEG
2013_PA61_I_Z4044	NEG	NEG	NEG	NEG	NEG	NEG	NEG	NEG	NEG	NEG	NEG	NEG	NEG	NEG	NEG	NEG	NEG	POS	NEG	NEG	NEG	NEG	NEG	NEG	NEG	NEG	POS	NEG	NEG	NEG
2013_PA62_I_Z4045	NEG	NEG	NEG	NEG	NEG	NEG	NEG	NEG	NEG	NEG	NEG	NEG	NEG	NEG	NEG	NEG	NEG	POS	NEG	NEG	NEG	NEG	NEG	NEG	NEG	NEG	POS	NEG	NEG	NEG
2013_PA74_Z4057	NEG	NEG	NEG	NEG	NEG	NEG	NEG	NEG	NEG	NEG	NEG	NEG	NEG	NEG	NEG	NEG	NEG	POS	NEG	NEG	NEG	NEG	NEG	NEG	NEG	NEG	POS	NEG	NEG	NEG
2013_PA75_Z4058	NEG	NEG	NEG	NEG	NEG	NEG	NEG	NEG	NEG	NEG	NEG	NEG	NEG	NEG	NEG	NEG	NEG	POS	NEG	NEG	NEG	NEG	NEG	NEG	NEG	NEG	POS	NEG	NEG	NEG
**CC5-MRSA-V [ACME+]**																														
2012_373_Z3433 (1204-17312)	NEG	POS	POS	POS	NEG	NEG	NEG	POS	NEG	NEG	NEG	NEG	NEG	NEG	NEG	NEG	NEG	POS	AMB	NEG	NEG	NEG	NEG	NEG	NEG	NEG	POS	NEG	NEG	NEG
**CC6-MSSA**																														
2012_173_Z3450 (98A/12)	NEG	NEG	NEG	NEG	POS	NEG	NEG	NEG	NEG	NEG	NEG	NEG	NEG	NEG	NEG	NEG	NEG	POS	POS	NEG	NEG	NEG	NEG	NEG	NEG	NEG	POS	NEG	NEG	NEG
2012_176_Z3498	NEG	NEG	NEG	NEG	NEG	NEG	NEG	NEG	NEG	NEG	NEG	NEG	NEG	NEG	NEG	NEG	NEG	POS	AMB	NEG	NEG	NEG	NEG	NEG	NEG	NEG	POS	NEG	NEG	NEG
2012_181_Z3452	NEG	NEG	NEG	NEG	NEG	NEG	NEG	NEG	NEG	NEG	NEG	NEG	NEG	NEG	NEG	NEG	NEG	POS	AMB	NEG	NEG	NEG	NEG	NEG	NEG	NEG	POS	NEG	NEG	NEG
2012_259_Z3546 (3279/12)	NEG	NEG	NEG	NEG	NEG	NEG	NEG	NEG	NEG	NEG	NEG	NEG	NEG	NEG	NEG	NEG	NEG	POS	POS	NEG	NEG	NEG	NEG	NEG	NEG	NEG	POS	NEG	NEG	NEG
2012_264_Z3551 (3407B/12)	NEG	NEG	NEG	NEG	NEG	NEG	NEG	NEG	NEG	NEG	NEG	NEG	NEG	NEG	NEG	NEG	NEG	POS	AMB	NEG	NEG	NEG	NEG	NEG	NEG	NEG	POS	NEG	NEG	NEG
2012_273_Z3560 (3844/12)	NEG	NEG	NEG	NEG	NEG	NEG	NEG	NEG	NEG	NEG	NEG	NEG	NEG	NEG	NEG	NEG	NEG	POS	AMB	NEG	NEG	NEG	NEG	NEG	NEG	NEG	POS	NEG	NEG	NEG
2012_312_Z3599 (6815/12)	NEG	NEG	NEG	NEG	NEG	NEG	NEG	NEG	NEG	NEG	NEG	NEG	NEG	NEG	NEG	NEG	NEG	POS	NEG	NEG	NEG	NEG	NEG	NEG	NEG	NEG	POS	NEG	NEG	NEG
2012_338_Z3625	NEG	NEG	NEG	NEG	NEG	NEG	NEG	NEG	NEG	NEG	NEG	NEG	NEG	NEG	NEG	NEG	NEG	POS	NEG	NEG	NEG	NEG	NEG	NEG	NEG	NEG	POS	NEG	NEG	NEG
2012_352_Z3639	NEG	NEG	NEG	NEG	NEG	NEG	NEG	NEG	NEG	NEG	NEG	NEG	NEG	NEG	NEG	NEG	NEG	POS	NEG	NEG	NEG	NEG	NEG	NEG	NEG	NEG	POS	NEG	NEG	NEG
2012_368_Z3428	NEG	NEG	NEG	NEG	NEG	NEG	NEG	NEG	NEG	NEG	NEG	NEG	NEG	NEG	NEG	NEG	NEG	POS	NEG	NEG	NEG	NEG	NEG	NEG	NEG	NEG	POS	NEG	NEG	NEG
2013_PA12_Z3995	NEG	NEG	NEG	NEG	NEG	NEG	NEG	NEG	NEG	NEG	NEG	NEG	NEG	NEG	NEG	NEG	NEG	POS	NEG	NEG	NEG	NEG	NEG	NEG	NEG	NEG	POS	NEG	NEG	NEG
2013_PA28_Z4011	NEG	NEG	NEG	NEG	NEG	NEG	NEG	NEG	NEG	NEG	NEG	NEG	NEG	NEG	NEG	NEG	NEG	POS	NEG	NEG	NEG	NEG	NEG	NEG	NEG	NEG	POS	NEG	NEG	NEG
**CC6-MSSA [PVL+]**																														
2012_375_Z3435	NEG	NEG	NEG	NEG	NEG	NEG	NEG	NEG	NEG	NEG	NEG	NEG	NEG	NEG	NEG	NEG	NEG	POS	AMB	NEG	NEG	NEG	NEG	NEG	NEG	NEG	POS	NEG	NEG	NEG
2013_PA77_Z4060	NEG	NEG	NEG	NEG	NEG	NEG	NEG	NEG	NEG	NEG	NEG	NEG	NEG	NEG	NEG	NEG	NEG	POS	NEG	NEG	NEG	NEG	NEG	NEG	NEG	NEG	POS	NEG	NEG	NEG
**CC7-MSSA**																														
2012_248_Z3532 (2864/12)	NEG	NEG	NEG	NEG	NEG	NEG	NEG	NEG	NEG	NEG	NEG	NEG	NEG	NEG	NEG	NEG	NEG	AMB	NEG	NEG	NEG	NEG	NEG	NEG	NEG	NEG	POS	NEG	NEG	NEG
2012_265_Z3552 (3518/12)	NEG	POS	NEG	NEG	NEG	NEG	NEG	NEG	NEG	NEG	NEG	NEG	NEG	NEG	NEG	NEG	NEG	POS	NEG	NEG	NEG	NEG	NEG	NEG	NEG	NEG	POS	NEG	NEG	NEG
2013_PA53_Z4036	NEG	NEG	NEG	NEG	NEG	NEG	NEG	NEG	NEG	NEG	NEG	NEG	NEG	NEG	NEG	NEG	NEG	NEG	NEG	POS	POS	POS	NEG	NEG	NEG	NEG	POS	NEG	NEG	NEG
2013_PA83_Z4066	NEG	NEG	NEG	NEG	NEG	NEG	NEG	NEG	NEG	NEG	NEG	NEG	NEG	NEG	NEG	NEG	NEG	NEG	NEG	NEG	NEG	NEG	NEG	NEG	NEG	NEG	POS	NEG	NEG	NEG
**CC8-MSSA**																														
2012_243_Z3528 (2587/12)	NEG	NEG	NEG	NEG	NEG	NEG	NEG	NEG	NEG	NEG	NEG	NEG	NEG	NEG	NEG	NEG	NEG	POS	NEG	NEG	NEG	NEG	NEG	NEG	NEG	NEG	POS	NEG	NEG	NEG
2012_344_Z3631 (8605/12)	NEG	NEG	NEG	NEG	NEG	NEG	NEG	NEG	NEG	NEG	NEG	NEG	NEG	NEG	NEG	NEG	NEG	POS	AMB	NEG	NEG	NEG	NEG	NEG	NEG	NEG	POS	NEG	NEG	NEG
2012_346_Z3633 (8074/12)	NEG	NEG	NEG	NEG	NEG	NEG	NEG	NEG	NEG	NEG	NEG	NEG	NEG	NEG	NEG	NEG	NEG	POS	NEG	NEG	NEG	NEG	NEG	NEG	NEG	NEG	POS	NEG	NEG	NEG
2012_255_Z3538 (3205B/12)	NEG	NEG	NEG	NEG	NEG	NEG	NEG	NEG	NEG	NEG	NEG	NEG	NEG	NEG	NEG	NEG	NEG	POS	NEG	NEG	NEG	NEG	NEG	NEG	NEG	NEG	POS	NEG	NEG	NEG
2012_280_Z3567 (4601/12)	NEG	NEG	NEG	NEG	NEG	NEG	NEG	NEG	NEG	POS	NEG	NEG	NEG	NEG	NEG	NEG	NEG	POS	AMB	NEG	NEG	NEG	NEG	NEG	NEG	NEG	POS	NEG	NEG	NEG
Trinidad&Tobago_2012_267 (3642/12)	NEG	NEG	NEG	NEG	NEG	NEG	NEG	NEG	NEG	NEG	NEG	NEG	NEG	NEG	NEG	NEG	NEG	POS	NEG	NEG	NEG	NEG	NEG	NEG	NEG	NEG	POS	NEG	NEG	NEG
2012_365_Z3425 (1204-15838)	NEG	NEG	NEG	NEG	NEG	NEG	NEG	NEG	NEG	NEG	NEG	NEG	NEG	NEG	NEG	NEG	NEG	POS	AMB	NEG	NEG	NEG	NEG	NEG	NEG	NEG	POS	NEG	NEG	NEG
2012_296_Z3583 (5118/12)	NEG	POS	NEG	NEG	NEG	NEG	NEG	POS	NEG	NEG	NEG	NEG	NEG	NEG	NEG	NEG	NEG	POS	NEG	NEG	NEG	NEG	NEG	NEG	NEG	NEG	POS	NEG	NEG	NEG
2012_318_Z3605 (6960/12)	NEG	NEG	NEG	NEG	NEG	POS	NEG	NEG	NEG	NEG	NEG	NEG	NEG	NEG	NEG	NEG	NEG	POS	NEG	NEG	NEG	NEG	NEG	NEG	NEG	NEG	POS	NEG	NEG	NEG
2012_306_Z3593 (5861/12)	NEG	NEG	NEG	NEG	NEG	NEG	NEG	NEG	NEG	NEG	NEG	NEG	NEG	NEG	NEG	NEG	NEG	POS	AMB	NEG	NEG	NEG	NEG	NEG	NEG	NEG	POS	NEG	NEG	NEG
2013_PA37_Z4020	NEG	POS	NEG	NEG	NEG	NEG	NEG	POS	NEG	NEG	NEG	NEG	NEG	NEG	NEG	NEG	NEG	POS	NEG	NEG	NEG	NEG	NEG	NEG	NEG	NEG	POS	NEG	NEG	NEG
2013_PA38_Z4021	NEG	NEG	NEG	NEG	NEG	NEG	NEG	NEG	NEG	NEG	NEG	NEG	NEG	NEG	NEG	NEG	NEG	POS	NEG	NEG	NEG	NEG	NEG	NEG	NEG	NEG	POS	NEG	NEG	NEG
2013_PA52_Z4035	NEG	NEG	NEG	NEG	NEG	NEG	NEG	AMB	NEG	NEG	NEG	NEG	NEG	NEG	NEG	NEG	NEG	POS	NEG	NEG	NEG	NEG	NEG	NEG	NEG	NEG	POS	NEG	NEG	NEG
**CC8-MSSA [PVL+]**																														
2012_156_Z3489 (25917/11)	NEG	NEG	NEG	NEG	NEG	NEG	NEG	NEG	NEG	NEG	NEG	NEG	NEG	NEG	NEG	NEG	NEG	POS	AMB	NEG	NEG	NEG	NEG	NEG	NEG	NEG	POS	NEG	NEG	NEG
2012_159_Z3492 (26310/11)	NEG	NEG	NEG	NEG	NEG	NEG	NEG	NEG	NEG	NEG	NEG	NEG	NEG	NEG	NEG	NEG	NEG	POS	AMB	NEG	NEG	NEG	NEG	NEG	NEG	NEG	POS	NEG	NEG	NEG
2012_161_Z3445 (26430/11)	NEG	NEG	NEG	NEG	AMB	NEG	NEG	NEG	NEG	NEG	NEG	NEG	NEG	NEG	NEG	NEG	NEG	POS	POS	NEG	NEG	NEG	NEG	NEG	NEG	NEG	POS	NEG	NEG	NEG
2012_162_Z3512 (26433/11)	NEG	NEG	NEG	NEG	NEG	NEG	NEG	NEG	NEG	NEG	NEG	NEG	NEG	NEG	NEG	NEG	NEG	POS	AMB	NEG	POS	NEG	POS	POS	NEG	NEG	POS	NEG	NEG	NEG
2012_169_Z3495 (27194/11)	NEG	NEG	NEG	NEG	NEG	NEG	NEG	NEG	NEG	NEG	NEG	NEG	NEG	NEG	NEG	NEG	NEG	POS	AMB	NEG	NEG	NEG	NEG	NEG	NEG	NEG	POS	NEG	NEG	NEG
2012_170_Z3496 (35/12)	NEG	NEG	NEG	NEG	NEG	NEG	NEG	NEG	NEG	NEG	NEG	NEG	NEG	NEG	NEG	NEG	NEG	POS	AMB	NEG	NEG	NEG	NEG	NEG	NEG	NEG	POS	NEG	NEG	NEG
2012_171_Z3448 (47/12)	NEG	NEG	NEG	NEG	NEG	NEG	NEG	NEG	NEG	NEG	NEG	NEG	NEG	NEG	NEG	NEG	NEG	POS	AMB	NEG	NEG	NEG	NEG	NEG	NEG	NEG	POS	NEG	NEG	NEG
2012_177_Z3499 (184/12)	NEG	NEG	NEG	NEG	NEG	NEG	NEG	NEG	NEG	NEG	NEG	NEG	NEG	NEG	NEG	NEG	NEG	POS	AMB	NEG	NEG	NEG	NEG	NEG	NEG	NEG	POS	NEG	NEG	NEG
2012_179_Z3515 (231/12)	NEG	POS	NEG	NEG	NEG	NEG	NEG	POS	NEG	NEG	NEG	NEG	NEG	NEG	NEG	NEG	NEG	POS	AMB	NEG	NEG	NEG	NEG	NEG	NEG	NEG	POS	NEG	NEG	NEG
2012_185_Z3502 (437/12)	NEG	POS	NEG	NEG	NEG	NEG	NEG	POS	NEG	NEG	NEG	NEG	NEG	NEG	NEG	NEG	NEG	POS	NEG	NEG	NEG	NEG	NEG	NEG	NEG	NEG	POS	NEG	NEG	NEG
2012_191_Z3456 (563/12)	NEG	NEG	NEG	NEG	NEG	NEG	NEG	NEG	NEG	NEG	NEG	NEG	NEG	NEG	NEG	NEG	NEG	POS	NEG	NEG	NEG	NEG	NEG	NEG	NEG	NEG	POS	NEG	NEG	NEG
2012_194_Z3459	NEG	NEG	NEG	NEG	NEG	NEG	NEG	NEG	NEG	NEG	NEG	NEG	NEG	NEG	NEG	NEG	NEG	POS	AMB	NEG	NEG	NEG	NEG	NEG	NEG	NEG	POS	NEG	NEG	NEG
2012_200_Z3460 (820/12)	NEG	NEG	NEG	NEG	AMB	NEG	NEG	NEG	NEG	NEG	NEG	NEG	NEG	NEG	NEG	NEG	NEG	POS	POS	NEG	NEG	NEG	NEG	NEG	NEG	NEG	POS	NEG	NEG	NEG
2012_207_Z3467 (1010/12)	NEG	NEG	NEG	NEG	NEG	NEG	NEG	NEG	NEG	NEG	NEG	NEG	NEG	NEG	NEG	NEG	NEG	POS	NEG	NEG	NEG	NEG	NEG	NEG	NEG	NEG	POS	NEG	NEG	NEG
2012_212_Z3471 (1261/12)	NEG	NEG	NEG	NEG	NEG	NEG	NEG	NEG	NEG	NEG	NEG	NEG	NEG	NEG	NEG	NEG	NEG	POS	NEG	NEG	NEG	NEG	NEG	NEG	NEG	NEG	POS	NEG	NEG	NEG
2012_220_Z3477 (1466/12)	NEG	NEG	NEG	NEG	NEG	NEG	NEG	NEG	NEG	NEG	NEG	NEG	NEG	NEG	NEG	NEG	NEG	POS	NEG	NEG	NEG	NEG	NEG	NEG	NEG	NEG	POS	NEG	NEG	NEG
2012_224_Z3481 (1522/12)	NEG	NEG	NEG	NEG	NEG	NEG	NEG	NEG	NEG	NEG	NEG	NEG	NEG	NEG	NEG	NEG	NEG	POS	NEG	NEG	NEG	NEG	NEG	NEG	NEG	NEG	POS	NEG	NEG	NEG
2012_226_Z3483 (1662/12)	NEG	NEG	NEG	NEG	NEG	NEG	NEG	NEG	NEG	NEG	NEG	NEG	NEG	NEG	NEG	NEG	NEG	POS	AMB	NEG	NEG	NEG	NEG	NEG	NEG	NEG	POS	NEG	NEG	NEG
2012_242_Z3527 (2581/12)	NEG	NEG	NEG	NEG	NEG	NEG	NEG	NEG	NEG	NEG	NEG	NEG	NEG	NEG	NEG	NEG	NEG	POS	AMB	NEG	NEG	NEG	NEG	NEG	NEG	NEG	POS	NEG	NEG	NEG
2012_245_Z3529 (2687/12)	NEG	NEG	NEG	NEG	NEG	NEG	NEG	NEG	NEG	NEG	NEG	NEG	NEG	NEG	NEG	NEG	NEG	POS	NEG	NEG	NEG	NEG	NEG	NEG	NEG	NEG	POS	NEG	NEG	NEG
2012_253_Z3536 (3074/12)	NEG	NEG	NEG	NEG	NEG	NEG	NEG	NEG	NEG	NEG	NEG	NEG	NEG	NEG	NEG	NEG	NEG	POS	AMB	NEG	NEG	NEG	NEG	NEG	NEG	NEG	POS	NEG	NEG	NEG
2012_258_Z3545 (3231/12)	NEG	NEG	NEG	NEG	NEG	NEG	NEG	NEG	NEG	NEG	NEG	NEG	NEG	NEG	NEG	NEG	NEG	POS	AMB	NEG	NEG	NEG	NEG	NEG	NEG	NEG	POS	NEG	NEG	NEG
2012_261_Z3548 (3298/12)	NEG	NEG	NEG	NEG	NEG	NEG	NEG	NEG	NEG	NEG	NEG	NEG	NEG	NEG	NEG	NEG	NEG	POS	AMB	NEG	NEG	NEG	NEG	NEG	NEG	NEG	POS	NEG	NEG	NEG
2012_270_Z3557 (3798/12)	NEG	NEG	NEG	NEG	NEG	NEG	NEG	NEG	NEG	NEG	NEG	NEG	NEG	NEG	NEG	NEG	NEG	POS	AMB	NEG	NEG	NEG	NEG	NEG	NEG	NEG	POS	NEG	NEG	NEG
2012_274_Z3561 (3861/12)	NEG	NEG	NEG	NEG	NEG	NEG	NEG	NEG	NEG	NEG	NEG	NEG	NEG	NEG	NEG	NEG	NEG	POS	NEG	NEG	NEG	NEG	NEG	NEG	NEG	NEG	POS	NEG	NEG	NEG
2012_282_Z3569 (4698/12)	NEG	NEG	NEG	NEG	NEG	NEG	NEG	NEG	NEG	NEG	NEG	NEG	NEG	NEG	NEG	NEG	NEG	POS	NEG	NEG	NEG	NEG	NEG	NEG	NEG	NEG	POS	NEG	NEG	NEG
2012_286_Z3573 (4805/12)	NEG	NEG	NEG	NEG	NEG	NEG	NEG	NEG	NEG	NEG	NEG	NEG	NEG	NEG	NEG	NEG	NEG	POS	NEG	NEG	NEG	NEG	NEG	NEG	NEG	NEG	POS	NEG	NEG	NEG
2012_293_Z3580 (5007/12)	NEG	NEG	NEG	NEG	NEG	NEG	NEG	NEG	NEG	NEG	NEG	NEG	NEG	NEG	NEG	NEG	NEG	POS	NEG	NEG	NEG	NEG	NEG	NEG	NEG	NEG	POS	NEG	NEG	NEG
2012_294_Z3581	NEG	NEG	NEG	NEG	NEG	NEG	NEG	NEG	NEG	NEG	NEG	NEG	NEG	NEG	NEG	NEG	NEG	POS	NEG	NEG	NEG	NEG	NEG	NEG	NEG	NEG	POS	NEG	NEG	NEG
2012_307_Z3594 (5918/12)	NEG	NEG	NEG	NEG	NEG	NEG	NEG	NEG	NEG	NEG	NEG	NEG	NEG	NEG	NEG	NEG	NEG	POS	AMB	NEG	NEG	NEG	NEG	NEG	NEG	NEG	POS	NEG	NEG	NEG
2012_333_Z3620 (8101/12)	NEG	NEG	NEG	NEG	NEG	NEG	NEG	NEG	NEG	NEG	NEG	NEG	NEG	NEG	NEG	NEG	NEG	POS	AMB	NEG	NEG	NEG	NEG	NEG	NEG	NEG	POS	NEG	NEG	NEG
2012_343_Z3630 (8195/12)	NEG	NEG	NEG	NEG	NEG	NEG	NEG	NEG	NEG	NEG	NEG	NEG	NEG	NEG	NEG	NEG	NEG	POS	AMB	NEG	NEG	NEG	NEG	NEG	NEG	NEG	POS	NEG	NEG	NEG
2012_349_Z3636 (7999A/12)	NEG	NEG	NEG	NEG	NEG	NEG	NEG	POS	NEG	AMB	NEG	NEG	NEG	NEG	NEG	NEG	NEG	POS	AMB	POS	NEG	NEG	NEG	NEG	NEG	NEG	POS	NEG	NEG	NEG
2012_353_Z3640 (8575/12)	NEG	NEG	NEG	NEG	NEG	NEG	NEG	NEG	NEG	NEG	NEG	NEG	NEG	NEG	NEG	NEG	NEG	POS	AMB	NEG	NEG	NEG	NEG	NEG	NEG	NEG	POS	NEG	NEG	NEG
2012_354_Z3641 (8408/12)	NEG	NEG	NEG	NEG	NEG	NEG	NEG	NEG	NEG	NEG	NEG	NEG	NEG	NEG	NEG	NEG	NEG	POS	AMB	NEG	NEG	NEG	NEG	NEG	NEG	NEG	POS	NEG	NEG	NEG
2012_355_Z3642 (1112-58348)	NEG	NEG	NEG	NEG	NEG	NEG	NEG	NEG	NEG	NEG	NEG	NEG	NEG	NEG	NEG	NEG	NEG	POS	NEG	NEG	NEG	NEG	NEG	NEG	NEG	NEG	POS	NEG	NEG	NEG
2012_357_Z3417 (1202-6360)	NEG	NEG	NEG	NEG	NEG	NEG	NEG	NEG	NEG	NEG	NEG	NEG	NEG	NEG	NEG	NEG	NEG	POS	AMB	NEG	NEG	NEG	NEG	NEG	NEG	NEG	POS	NEG	NEG	NEG
2012_364_Z3424 (1112-60417)	NEG	NEG	NEG	NEG	NEG	NEG	NEG	NEG	NEG	NEG	NEG	NEG	NEG	NEG	NEG	NEG	NEG	POS	AMB	NEG	NEG	NEG	NEG	NEG	NEG	NEG	POS	NEG	NEG	NEG
2012_376_Z3436 (6256)	NEG	NEG	NEG	NEG	AMB	NEG	NEG	NEG	NEG	NEG	NEG	NEG	NEG	NEG	NEG	NEG	NEG	POS	AMB	NEG	NEG	NEG	NEG	NEG	NEG	NEG	POS	NEG	NEG	NEG
2012_383_Z3443	NEG	NEG	NEG	NEG	NEG	NEG	NEG	NEG	NEG	NEG	NEG	NEG	NEG	NEG	NEG	NEG	NEG	POS	AMB	NEG	NEG	NEG	NEG	NEG	NEG	NEG	POS	NEG	NEG	NEG
2013_PA01_Z3984	NEG	NEG	NEG	NEG	NEG	NEG	NEG	NEG	NEG	NEG	NEG	NEG	NEG	NEG	NEG	NEG	NEG	POS	NEG	NEG	NEG	NEG	NEG	NEG	NEG	NEG	POS	NEG	NEG	NEG
2013_PA08_Z3991	NEG	NEG	NEG	NEG	NEG	NEG	NEG	NEG	NEG	NEG	NEG	NEG	NEG	NEG	NEG	NEG	NEG	POS	NEG	NEG	NEG	NEG	NEG	NEG	NEG	NEG	POS	NEG	NEG	NEG
2013_PA10_Z3993	NEG	NEG	NEG	NEG	NEG	NEG	NEG	NEG	POS	NEG	NEG	NEG	NEG	NEG	NEG	NEG	NEG	POS	NEG	NEG	NEG	NEG	NEG	NEG	NEG	NEG	POS	NEG	NEG	NEG
2013_PA13_Z3996	NEG	NEG	NEG	NEG	NEG	NEG	NEG	NEG	NEG	NEG	NEG	NEG	NEG	NEG	NEG	NEG	NEG	POS	NEG	NEG	NEG	NEG	NEG	NEG	NEG	NEG	POS	NEG	NEG	NEG
2013_PA14_Z3997	NEG	NEG	NEG	NEG	NEG	NEG	NEG	NEG	NEG	NEG	NEG	NEG	NEG	NEG	NEG	NEG	NEG	POS	NEG	NEG	NEG	NEG	NEG	NEG	NEG	NEG	POS	NEG	NEG	NEG
2013_PA17_Z4000	NEG	NEG	NEG	NEG	NEG	NEG	NEG	NEG	NEG	NEG	NEG	NEG	NEG	NEG	NEG	NEG	NEG	POS	NEG	NEG	NEG	NEG	NEG	NEG	NEG	NEG	POS	NEG	NEG	NEG
2013_PA26_Z4009	NEG	NEG	NEG	NEG	NEG	NEG	NEG	NEG	NEG	NEG	NEG	NEG	NEG	NEG	NEG	NEG	NEG	POS	NEG	NEG	NEG	NEG	NEG	NEG	NEG	NEG	POS	NEG	NEG	NEG
2013_PA27_Z4010	NEG	NEG	NEG	NEG	NEG	NEG	NEG	NEG	NEG	NEG	NEG	NEG	NEG	NEG	NEG	NEG	NEG	POS	NEG	NEG	NEG	NEG	NEG	NEG	NEG	NEG	POS	NEG	NEG	NEG
2013_PA42_Z4025	NEG	NEG	NEG	NEG	NEG	NEG	NEG	NEG	NEG	NEG	NEG	NEG	NEG	NEG	NEG	NEG	NEG	POS	NEG	NEG	NEG	NEG	NEG	NEG	NEG	NEG	POS	NEG	NEG	NEG
2013_PA43_Z4026	NEG	NEG	NEG	NEG	NEG	NEG	NEG	NEG	NEG	NEG	NEG	NEG	NEG	NEG	NEG	NEG	NEG	POS	NEG	NEG	NEG	NEG	NEG	NEG	NEG	NEG	POS	NEG	NEG	NEG
2013_PA45_Z4028	NEG	NEG	NEG	NEG	NEG	NEG	NEG	NEG	NEG	NEG	NEG	NEG	NEG	NEG	NEG	NEG	NEG	POS	NEG	NEG	NEG	NEG	NEG	NEG	NEG	NEG	POS	NEG	NEG	NEG
2013_PA56_Z4039	NEG	NEG	NEG	NEG	NEG	NEG	NEG	NEG	NEG	NEG	NEG	NEG	NEG	NEG	NEG	NEG	NEG	POS	NEG	NEG	NEG	NEG	NEG	NEG	NEG	NEG	POS	NEG	NEG	NEG
2013_PA58_Z4041	NEG	NEG	NEG	NEG	NEG	NEG	NEG	NEG	NEG	NEG	NEG	NEG	NEG	NEG	NEG	NEG	NEG	POS	AMB	NEG	NEG	NEG	NEG	NEG	NEG	NEG	POS	NEG	NEG	NEG
2013_PA64_Z4047	NEG	NEG	NEG	NEG	NEG	NEG	NEG	NEG	NEG	NEG	NEG	NEG	NEG	NEG	NEG	NEG	NEG	POS	NEG	NEG	NEG	NEG	NEG	NEG	NEG	NEG	POS	NEG	NEG	NEG
2013_PA66_Z4049	NEG	NEG	NEG	NEG	NEG	NEG	NEG	NEG	NEG	NEG	NEG	NEG	NEG	NEG	NEG	NEG	NEG	POS	NEG	NEG	NEG	NEG	NEG	NEG	NEG	NEG	POS	NEG	NEG	NEG
2013_PA67_Z4050	NEG	NEG	NEG	NEG	NEG	NEG	NEG	NEG	NEG	NEG	NEG	NEG	NEG	NEG	NEG	NEG	NEG	POS	NEG	NEG	POS	NEG	NEG	NEG	NEG	POS	POS	NEG	NEG	NEG
2013_PA69_Z4052	NEG	NEG	NEG	NEG	NEG	NEG	NEG	NEG	NEG	NEG	NEG	NEG	NEG	NEG	NEG	NEG	NEG	POS	NEG	NEG	NEG	NEG	NEG	NEG	NEG	NEG	POS	NEG	NEG	NEG
2013_PA72_Z4055	NEG	NEG	NEG	NEG	NEG	NEG	NEG	NEG	NEG	NEG	NEG	NEG	NEG	NEG	NEG	NEG	NEG	POS	NEG	NEG	NEG	NEG	NEG	NEG	NEG	NEG	POS	NEG	NEG	NEG
2013_PA76_Z4059	NEG	NEG	NEG	NEG	NEG	NEG	NEG	NEG	NEG	NEG	NEG	NEG	NEG	NEG	NEG	NEG	NEG	POS	NEG	NEG	NEG	NEG	NEG	NEG	NEG	NEG	POS	NEG	NEG	NEG
2013_PA80_Z4063	NEG	NEG	NEG	NEG	NEG	NEG	NEG	POS	NEG	NEG	NEG	NEG	NEG	NEG	NEG	NEG	NEG	POS	NEG	NEG	POS	POS	NEG	NEG	NEG	NEG	POS	NEG	NEG	NEG

Strain / Isolates	bifunctional enzyme Aac/Aph, gentamicin resistance	amino-glycoside adenyl-transferase, tobramycin resistance	3'5'-aminoglycoside phospho-transferase, neo-/kanamycin resistance	streptothricine-acetyl-transferase	dihydrofolate reductase type 1	fusidic acid resistence	hypothetical protein associated with fusidic acid resistance	mupirocin resistence protein	tetrazyklin-resistance	tetracycline-resistance	chloramphenicol acetyltransferase						chloramphenicol/florfenicol exporter	metallothiol transferase		quaternary ammonium compound resistance protein A	quaternary ammonium compound resistance protein C						Transport-/Effluxprotein	vancomycin resistance gene	vancomycin resistance gene from enterococci and Clostridium	teicoplanin resistance gene from enterococci
	aacA-aphD	aadD	aphA3	sat	dfrS1	far1	fusC (Q6GD50)	mupA	tet(K)	tet(M)	cat (total)	cat (pC221)	cat (pc223)	cat (pMC524/pC194)	cat (pSBK203R)	cfr	fexA	fosB	fosB (plasmid)	qacA	qacC (total)	qacC (consensus)	qacC (equine)	qacC (SA5)	qacC (Ssap)	qacC (ST94)	sdrM	vanA	vanB	vanZ
**ST8-MRSA-IV, USA300**																														
2012_158_Z3491 (26049A/11)	NEG	NEG	POS	POS	NEG	NEG	NEG	NEG	NEG	NEG	NEG	NEG	NEG	NEG	NEG	NEG	NEG	POS	AMB	NEG	NEG	NEG	NEG	NEG	NEG	NEG	POS	NEG	NEG	NEG
2012_165_Z3513 (26656/11)	NEG	NEG	POS	POS	AMB	NEG	NEG	NEG	NEG	NEG	NEG	NEG	NEG	NEG	NEG	NEG	NEG	POS	AMB	NEG	POS	POS	NEG	NEG	AMB	NEG	POS	NEG	NEG	NEG
2012_214_Z3473 (1322/12)	NEG	NEG	POS	POS	NEG	NEG	NEG	NEG	NEG	NEG	NEG	NEG	NEG	NEG	NEG	NEG	NEG	POS	NEG	NEG	NEG	NEG	NEG	NEG	NEG	NEG	POS	NEG	NEG	NEG
2012_227_Z3484 (1688/12)	NEG	POS	POS	POS	AMB	NEG	NEG	POS	NEG	NEG	NEG	NEG	NEG	NEG	NEG	NEG	NEG	POS	POS	NEG	NEG	NEG	NEG	NEG	NEG	NEG	POS	NEG	NEG	NEG
2012_263_Z3550 (3355/12)	NEG	NEG	POS	POS	NEG	NEG	NEG	NEG	NEG	NEG	NEG	NEG	NEG	NEG	NEG	NEG	NEG	POS	AMB	NEG	NEG	NEG	NEG	NEG	NEG	NEG	POS	NEG	NEG	NEG
2012_266_Z3553	NEG	NEG	POS	POS	NEG	NEG	NEG	NEG	NEG	NEG	NEG	NEG	NEG	NEG	NEG	NEG	NEG	POS	AMB	NEG	NEG	NEG	NEG	NEG	NEG	NEG	POS	NEG	NEG	NEG
2012_284_Z3571 (4729/12)	NEG	NEG	POS	POS	NEG	NEG	NEG	NEG	NEG	NEG	NEG	NEG	NEG	NEG	NEG	NEG	NEG	POS	NEG	NEG	NEG	NEG	NEG	NEG	NEG	NEG	POS	NEG	NEG	NEG
2012_298_Z3585 (5203/12)	NEG	NEG	POS	POS	NEG	NEG	NEG	NEG	NEG	NEG	NEG	NEG	NEG	NEG	NEG	NEG	NEG	POS	NEG	NEG	NEG	NEG	NEG	NEG	NEG	NEG	POS	NEG	NEG	NEG
2012_304_Z3591 (5818/12)	NEG	NEG	POS	POS	NEG	NEG	NEG	NEG	NEG	NEG	NEG	NEG	NEG	NEG	NEG	NEG	NEG	POS	NEG	NEG	NEG	NEG	NEG	NEG	NEG	NEG	POS	NEG	NEG	NEG
Z3595_TT308 - {EFC74B77-3925-484F-A4CF-A1F7DC	NEG	NEG	POS	POS	NEG	NEG	NEG	NEG	NEG	NEG	NEG	NEG	NEG	NEG	NEG	NEG	NEG	POS	NEG	NEG	NEG	NEG	NEG	NEG	NEG	NEG	POS	NEG	NEG	NEG
2012_369_Z3429 (1112-58547)	NEG	NEG	NEG	NEG	NEG	NEG	NEG	NEG	NEG	NEG	NEG	NEG	NEG	NEG	NEG	NEG	NEG	POS	AMB	NEG	NEG	NEG	NEG	NEG	NEG	NEG	POS	NEG	NEG	NEG
2012_374_Z3434 (1203-13527)	NEG	NEG	POS	POS	NEG	NEG	NEG	NEG	NEG	NEG	NEG	NEG	NEG	NEG	NEG	NEG	NEG	POS	AMB	NEG	NEG	NEG	NEG	NEG	NEG	NEG	POS	NEG	NEG	NEG
2013_PA06_Z3989	NEG	NEG	POS	POS	NEG	NEG	NEG	NEG	NEG	NEG	NEG	NEG	NEG	NEG	NEG	NEG	NEG	POS	NEG	NEG	NEG	NEG	NEG	NEG	NEG	NEG	POS	NEG	NEG	NEG
2013_PA55_Z4038	NEG	NEG	POS	POS	NEG	NEG	NEG	NEG	NEG	NEG	NEG	NEG	NEG	NEG	NEG	NEG	NEG	POS	NEG	NEG	NEG	NEG	NEG	NEG	NEG	NEG	POS	NEG	NEG	NEG
2013_PA71_Z4054	POS	POS	POS	POS	NEG	NEG	NEG	NEG	POS	NEG	NEG	NEG	NEG	NEG	NEG	NEG	NEG	POS	NEG	NEG	POS	POS	NEG	NEG	NEG	NEG	POS	NEG	NEG	NEG
**ST8-MRSA-IV [PVL+/ACME+], US**																														
2012_180_Z3501 (232/12)	NEG	NEG	POS	POS	NEG	NEG	NEG	NEG	NEG	NEG	NEG	NEG	NEG	NEG	NEG	NEG	NEG	POS	NEG	NEG	NEG	NEG	NEG	NEG	NEG	NEG	POS	NEG	NEG	NEG
2013_PA68_Z4051	NEG	NEG	NEG	NEG	NEG	NEG	NEG	POS	NEG	NEG	NEG	NEG	NEG	NEG	NEG	NEG	NEG	POS	NEG	NEG	NEG	NEG	NEG	NEG	NEG	NEG	POS	NEG	NEG	NEG
**ST72-MSSA**																														
2012_174_Z3451 (102/12)	NEG	POS	NEG	NEG	NEG	NEG	NEG	NEG	NEG	NEG	NEG	NEG	NEG	NEG	NEG	NEG	NEG	POS	AMB	NEG	NEG	NEG	NEG	NEG	NEG	NEG	POS	NEG	NEG	NEG
2012_223_Z3480 (1506A/12)	NEG	NEG	NEG	NEG	NEG	NEG	NEG	NEG	NEG	NEG	NEG	NEG	NEG	NEG	NEG	NEG	NEG	POS	NEG	NEG	NEG	NEG	NEG	NEG	NEG	NEG	POS	NEG	NEG	NEG
2012_236_Z3523 (2086/12)	NEG	NEG	NEG	NEG	NEG	NEG	NEG	NEG	NEG	NEG	NEG	NEG	NEG	NEG	NEG	NEG	NEG	POS	AMB	NEG	NEG	NEG	NEG	NEG	NEG	NEG	POS	NEG	NEG	NEG
2012_239_Z3525 (2346/12)	NEG	NEG	NEG	NEG	NEG	NEG	NEG	NEG	NEG	NEG	NEG	NEG	NEG	NEG	NEG	NEG	NEG	POS	AMB	NEG	NEG	NEG	NEG	NEG	NEG	NEG	POS	NEG	NEG	NEG
2012_252_Z3535 (3030/12)	NEG	NEG	NEG	NEG	AMB	NEG	NEG	NEG	NEG	NEG	NEG	NEG	NEG	NEG	NEG	NEG	NEG	POS	POS	NEG	NEG	NEG	NEG	NEG	NEG	NEG	POS	NEG	NEG	NEG
2012_271_Z3558 (3799/12)	NEG	NEG	NEG	NEG	NEG	NEG	NEG	NEG	NEG	NEG	NEG	NEG	NEG	NEG	NEG	NEG	NEG	POS	AMB	NEG	NEG	NEG	NEG	NEG	NEG	NEG	POS	NEG	NEG	NEG
2012_276_Z3563	NEG	NEG	NEG	NEG	NEG	NEG	NEG	NEG	NEG	NEG	NEG	NEG	NEG	NEG	NEG	NEG	NEG	POS	AMB	NEG	NEG	NEG	NEG	NEG	NEG	NEG	POS	NEG	NEG	NEG
2012_300_Z3587 (5638/12)	NEG	NEG	NEG	NEG	NEG	NEG	NEG	NEG	NEG	NEG	NEG	NEG	NEG	NEG	NEG	NEG	NEG	POS	NEG	NEG	NEG	NEG	NEG	NEG	NEG	NEG	POS	NEG	NEG	NEG
2012_316_Z3603 (6941/12)	NEG	NEG	NEG	NEG	NEG	NEG	NEG	NEG	NEG	NEG	NEG	NEG	NEG	NEG	NEG	NEG	NEG	POS	AMB	NEG	NEG	NEG	NEG	NEG	NEG	NEG	POS	NEG	NEG	NEG
2012_319_Z3606 (7019/12)	NEG	NEG	NEG	NEG	NEG	NEG	NEG	NEG	NEG	NEG	NEG	NEG	NEG	NEG	NEG	NEG	NEG	POS	NEG	NEG	NEG	NEG	NEG	NEG	NEG	NEG	POS	NEG	NEG	NEG
2012_371_Z3431 (1204-17577)	NEG	NEG	NEG	NEG	NEG	NEG	NEG	NEG	NEG	NEG	NEG	NEG	NEG	NEG	NEG	NEG	NEG	POS	AMB	NEG	NEG	NEG	NEG	NEG	NEG	NEG	POS	NEG	NEG	NEG
2013_PA05_Z3988	NEG	NEG	NEG	NEG	NEG	NEG	NEG	NEG	NEG	NEG	NEG	NEG	NEG	NEG	NEG	NEG	NEG	POS	NEG	NEG	NEG	NEG	NEG	NEG	NEG	NEG	POS	NEG	NEG	NEG
2013_PA65_Z4048	NEG	NEG	NEG	NEG	NEG	NEG	NEG	NEG	NEG	NEG	NEG	NEG	NEG	NEG	NEG	NEG	NEG	POS	NEG	NEG	NEG	NEG	NEG	NEG	NEG	NEG	POS	NEG	NEG	NEG
2013_PA73_Z4056	NEG	NEG	NEG	NEG	NEG	NEG	NEG	NEG	NEG	NEG	NEG	NEG	NEG	NEG	NEG	NEG	NEG	POS	NEG	NEG	NEG	NEG	NEG	NEG	NEG	NEG	POS	NEG	NEG	NEG
**ST72-MSSA-SCCfus**																														
2013_PA16_Z3999	NEG	NEG	NEG	NEG	NEG	NEG	POS	NEG	NEG	NEG	NEG	NEG	NEG	NEG	NEG	NEG	NEG	POS	NEG	NEG	NEG	NEG	NEG	NEG	NEG	NEG	POS	NEG	NEG	NEG
**ST72-MSSA-SCCfus/kdp**																														
2012_361_Z3421 (1201-1299)	NEG	NEG	NEG	NEG	NEG	NEG	POS	NEG	NEG	NEG	NEG	NEG	NEG	NEG	NEG	NEG	NEG	POS	AMB	NEG	NEG	NEG	NEG	NEG	NEG	NEG	POS	NEG	NEG	NEG
**ST239-MRSA-III&SCCmer**																														
2012_157_Z3490	POS	NEG	POS	POS	NEG	NEG	NEG	NEG	POS	POS	NEG	NEG	NEG	NEG	NEG	NEG	NEG	POS	AMB	NEG	NEG	NEG	NEG	NEG	NEG	NEG	POS	NEG	NEG	NEG
2012_168_Z3494 (27129/11)	POS	NEG	POS	POS	NEG	NEG	NEG	NEG	POS	POS	NEG	NEG	NEG	NEG	NEG	NEG	NEG	POS	AMB	POS	NEG	NEG	NEG	NEG	NEG	NEG	POS	NEG	NEG	NEG
2012_195_Z3508 (619/12)	POS	NEG	POS	POS	NEG	NEG	NEG	NEG	NEG	POS	NEG	NEG	NEG	NEG	NEG	NEG	NEG	POS	AMB	NEG	NEG	NEG	NEG	NEG	NEG	NEG	POS	NEG	NEG	NEG
2012_222_Z3479 (1489/12)	POS	NEG	POS	POS	NEG	NEG	NEG	NEG	POS	POS	NEG	NEG	NEG	NEG	NEG	NEG	NEG	POS	NEG	POS	NEG	NEG	NEG	NEG	NEG	NEG	POS	NEG	NEG	NEG
2012_233_Z3540 (1943/12)	POS	NEG	POS	POS	NEG	NEG	NEG	NEG	POS	POS	NEG	NEG	NEG	NEG	NEG	NEG	NEG	POS	NEG	POS	NEG	NEG	NEG	NEG	NEG	NEG	POS	NEG	NEG	NEG
2012_237_Z3541	POS	NEG	POS	POS	NEG	NEG	NEG	NEG	POS	POS	NEG	NEG	NEG	NEG	NEG	NEG	NEG	POS	NEG	POS	NEG	NEG	NEG	NEG	NEG	NEG	POS	NEG	NEG	NEG
2012_262_Z3549	POS	NEG	POS	POS	NEG	NEG	NEG	NEG	POS	POS	NEG	NEG	NEG	NEG	NEG	NEG	NEG	POS	AMB	NEG	NEG	NEG	NEG	NEG	NEG	NEG	POS	NEG	NEG	NEG
2012_275_Z3562 (3893/12)	POS	NEG	POS	POS	NEG	NEG	NEG	NEG	POS	POS	POS	NEG	POS	NEG	POS	NEG	NEG	POS	AMB	NEG	NEG	NEG	NEG	NEG	NEG	NEG	POS	NEG	NEG	NEG
2012_291_Z3578	POS	NEG	POS	POS	NEG	NEG	NEG	NEG	POS	POS	NEG	NEG	NEG	NEG	NEG	NEG	NEG	POS	NEG	NEG	NEG	NEG	NEG	NEG	NEG	NEG	POS	NEG	NEG	NEG
2012_314_Z3601 (6939/12)	POS	NEG	POS	POS	NEG	NEG	NEG	NEG	POS	POS	NEG	NEG	NEG	NEG	NEG	NEG	NEG	POS	NEG	NEG	NEG	NEG	NEG	NEG	NEG	NEG	POS	NEG	NEG	NEG
2012323_Z3610	POS	NEG	POS	POS	NEG	NEG	NEG	NEG	POS	POS	NEG	NEG	NEG	NEG	NEG	NEG	NEG	POS	AMB	POS	NEG	NEG	NEG	NEG	NEG	NEG	POS	NEG	NEG	NEG
2012330_Z3617 (7605/12)	POS	NEG	POS	POS	NEG	NEG	NEG	NEG	NEG	POS	NEG	NEG	NEG	NEG	NEG	NEG	NEG	POS	AMB	NEG	NEG	NEG	NEG	NEG	NEG	NEG	POS	NEG	NEG	NEG
2012332_Z3619 (8076/12)	POS	NEG	POS	POS	NEG	NEG	NEG	NEG	POS	POS	NEG	NEG	NEG	NEG	NEG	NEG	NEG	POS	AMB	POS	NEG	NEG	NEG	NEG	NEG	NEG	POS	NEG	NEG	NEG
2012339_Z3626 (8558/12)	POS	NEG	POS	POS	NEG	NEG	NEG	NEG	POS	POS	NEG	NEG	NEG	NEG	NEG	NEG	NEG	POS	NEG	POS	NEG	NEG	NEG	NEG	NEG	NEG	POS	NEG	NEG	NEG
2012340_Z3627 (8523/12)	POS	NEG	POS	POS	NEG	NEG	NEG	NEG	POS	POS	NEG	NEG	NEG	NEG	NEG	NEG	NEG	POS	NEG	POS	NEG	NEG	NEG	NEG	NEG	NEG	POS	NEG	NEG	NEG
2012348_Z3635	POS	NEG	POS	POS	NEG	NEG	NEG	NEG	POS	POS	NEG	NEG	NEG	NEG	NEG	NEG	NEG	POS	AMB	POS	NEG	NEG	NEG	NEG	NEG	NEG	POS	NEG	NEG	NEG
2012350_Z3637	POS	NEG	POS	POS	NEG	NEG	NEG	NEG	NEG	POS	NEG	NEG	NEG	NEG	NEG	NEG	NEG	POS	NEG	POS	NEG	NEG	NEG	NEG	NEG	NEG	POS	NEG	NEG	NEG
2012_370_Z3430 (1112-52177)	POS	POS	POS	POS	NEG	NEG	NEG	POS	POS	POS	NEG	NEG	NEG	NEG	NEG	NEG	NEG	POS	AMB	NEG	NEG	NEG	NEG	NEG	NEG	NEG	POS	NEG	NEG	NEG
2012_378_Z3438 (4139)	POS	POS	POS	POS	NEG	NEG	NEG	POS	POS	POS	NEG	NEG	NEG	NEG	NEG	NEG	NEG	POS	AMB	POS	POS	NEG	NEG	NEG	NEG	POS	POS	NEG	NEG	NEG
2013_PA21_Z4004	POS	NEG	POS	POS	NEG	NEG	NEG	POS	POS	POS	NEG	NEG	NEG	NEG	NEG	NEG	NEG	POS	NEG	POS	NEG	NEG	NEG	NEG	NEG	NEG	POS	NEG	NEG	NEG
2013_PA22_Z4005	POS	NEG	POS	POS	NEG	NEG	NEG	NEG	POS	POS	NEG	NEG	NEG	NEG	NEG	NEG	NEG	POS	NEG	POS	NEG	NEG	NEG	NEG	NEG	NEG	POS	NEG	NEG	NEG
2013_PA24_Z4007	POS	NEG	POS	POS	NEG	NEG	NEG	NEG	POS	POS	NEG	NEG	NEG	NEG	NEG	NEG	NEG	POS	NEG	POS	NEG	NEG	NEG	NEG	NEG	NEG	POS	NEG	NEG	NEG
2013_PA31_Z4014	POS	NEG	POS	POS	NEG	NEG	NEG	POS	POS	POS	NEG	NEG	NEG	NEG	NEG	NEG	NEG	POS	NEG	POS	NEG	NEG	NEG	NEG	NEG	NEG	POS	NEG	NEG	NEG
2013_PA35_Z4018	POS	NEG	POS	POS	NEG	NEG	NEG	NEG	POS	POS	NEG	NEG	NEG	NEG	NEG	NEG	NEG	POS	NEG	POS	NEG	NEG	NEG	NEG	NEG	NEG	POS	NEG	NEG	NEG
2013_PA81_Z4064	POS	NEG	POS	POS	NEG	NEG	NEG	POS	POS	POS	NEG	NEG	NEG	NEG	NEG	NEG	NEG	POS	NEG	POS	NEG	NEG	NEG	NEG	NEG	NEG	POS	NEG	NEG	NEG
**ST239-MRSA-III+ccrA/B4**																														
2012_254_Z3537 (3124A/12)	POS	NEG	POS	POS	NEG	NEG	NEG	NEG	NEG	POS	NEG	NEG	NEG	NEG	NEG	NEG	NEG	POS	NEG	POS	NEG	NEG	NEG	NEG	NEG	NEG	POS	NEG	NEG	NEG
2013_PA25_Z4008	POS	NEG	POS	POS	NEG	NEG	NEG	NEG	NEG	POS	NEG	NEG	NEG	NEG	NEG	NEG	NEG	POS	NEG	POS	NEG	NEG	NEG	NEG	NEG	NEG	POS	NEG	NEG	NEG
**ST834-MSSA**																														
2012_268_Z3555 (3693/12)	NEG	NEG	NEG	NEG	NEG	NEG	NEG	NEG	NEG	NEG	NEG	NEG	NEG	NEG	NEG	NEG	NEG	POS	AMB	NEG	NEG	NEG	NEG	NEG	NEG	NEG	POS	NEG	NEG	NEG
**CC12-MSSA**																														
2012_210_Z3470 (1177/12)	NEG	NEG	NEG	NEG	NEG	NEG	NEG	NEG	NEG	NEG	NEG	NEG	NEG	NEG	NEG	NEG	NEG	POS	NEG	POS	NEG	NEG	NEG	NEG	NEG	NEG	POS	NEG	NEG	NEG
2012_230_Z3487 (1797/12)	NEG	NEG	NEG	NEG	NEG	NEG	NEG	NEG	NEG	NEG	NEG	NEG	NEG	NEG	NEG	NEG	NEG	POS	AMB	NEG	NEG	NEG	NEG	NEG	NEG	NEG	POS	NEG	NEG	NEG
2013_PA18_Z4001	NEG	NEG	NEG	NEG	NEG	NEG	NEG	NEG	NEG	NEG	NEG	NEG	NEG	NEG	NEG	NEG	NEG	POS	NEG	NEG	NEG	NEG	NEG	NEG	NEG	NEG	POS	NEG	NEG	NEG
**CC15-MSSA**																														
2012_160_Z3444 (26386/11)	NEG	NEG	NEG	NEG	NEG	NEG	NEG	NEG	NEG	NEG	NEG	NEG	NEG	NEG	NEG	NEG	NEG	POS	AMB	NEG	NEG	NEG	NEG	NEG	NEG	NEG	POS	NEG	NEG	NEG
2012_167_Z3514 (27010/11)	NEG	NEG	NEG	NEG	NEG	NEG	NEG	NEG	NEG	NEG	NEG	NEG	NEG	NEG	NEG	NEG	NEG	POS	AMB	NEG	NEG	NEG	NEG	NEG	NEG	NEG	POS	NEG	NEG	NEG
2012_184_Z3455 (376/12)	NEG	NEG	NEG	NEG	NEG	NEG	NEG	NEG	NEG	NEG	NEG	NEG	NEG	NEG	NEG	NEG	NEG	POS	AMB	NEG	NEG	NEG	NEG	NEG	NEG	NEG	POS	NEG	NEG	NEG
2012_205_Z3465 (939/12)	NEG	NEG	NEG	NEG	NEG	NEG	NEG	NEG	NEG	NEG	NEG	NEG	NEG	NEG	NEG	NEG	NEG	POS	AMB	NEG	NEG	NEG	NEG	NEG	NEG	NEG	POS	NEG	NEG	NEG
2012_231_Z3519 (1831/12)	NEG	NEG	NEG	NEG	NEG	NEG	NEG	NEG	NEG	NEG	NEG	NEG	NEG	NEG	NEG	NEG	NEG	POS	AMB	NEG	NEG	NEG	NEG	NEG	NEG	NEG	POS	NEG	NEG	NEG
2012_249_Z3533 (2907/12)	NEG	NEG	NEG	NEG	NEG	NEG	NEG	NEG	NEG	NEG	NEG	NEG	NEG	NEG	NEG	NEG	NEG	POS	AMB	NEG	NEG	NEG	NEG	NEG	NEG	NEG	POS	NEG	NEG	NEG
2012_305_Z3592 (5839/12)	NEG	NEG	NEG	NEG	NEG	NEG	NEG	NEG	NEG	NEG	NEG	NEG	NEG	NEG	NEG	NEG	NEG	POS	NEG	NEG	NEG	NEG	NEG	NEG	NEG	NEG	POS	NEG	NEG	NEG
2012_313_Z3600 (6857/12)	NEG	NEG	NEG	NEG	NEG	NEG	NEG	NEG	NEG	NEG	NEG	NEG	NEG	NEG	NEG	NEG	NEG	POS	AMB	NEG	NEG	NEG	NEG	NEG	NEG	NEG	POS	NEG	NEG	NEG
2012_345_Z3632 (7966B/12)	NEG	NEG	NEG	NEG	NEG	NEG	NEG	NEG	NEG	NEG	NEG	NEG	NEG	NEG	NEG	NEG	NEG	POS	AMB	NEG	NEG	NEG	NEG	NEG	NEG	NEG	POS	NEG	NEG	NEG
2013_PA33_Z4016	NEG	NEG	NEG	NEG	NEG	NEG	NEG	NEG	NEG	NEG	NEG	NEG	NEG	NEG	NEG	NEG	NEG	POS	NEG	NEG	NEG	NEG	NEG	NEG	NEG	NEG	POS	NEG	NEG	NEG
**CC20-MSSA**																														
2012_240_Z3526 (2354/12)	NEG	NEG	NEG	NEG	NEG	NEG	NEG	NEG	NEG	NEG	NEG	NEG	NEG	NEG	NEG	NEG	NEG	POS	AMB	NEG	NEG	NEG	NEG	NEG	NEG	NEG	POS	NEG	NEG	NEG
**CC30-MSSA**																														
2012_155_Z3488 (25905A/11)	NEG	NEG	NEG	NEG	NEG	NEG	NEG	POS	NEG	POS	NEG	NEG	NEG	NEG	NEG	NEG	NEG	POS	AMB	NEG	NEG	NEG	NEG	NEG	NEG	NEG	POS	NEG	NEG	NEG
**CC30-MSSA [PVL+]**																														
2012_164_Z3447 (26574/11)	NEG	NEG	NEG	NEG	AMB	NEG	NEG	NEG	NEG	NEG	NEG	NEG	NEG	NEG	NEG	NEG	NEG	POS	AMB	NEG	NEG	NEG	NEG	NEG	NEG	NEG	POS	NEG	NEG	NEG
2012_166_Z3493 (26988A/11)	NEG	NEG	NEG	NEG	NEG	NEG	NEG	NEG	NEG	NEG	NEG	NEG	NEG	NEG	NEG	NEG	NEG	POS	NEG	NEG	NEG	NEG	NEG	NEG	NEG	NEG	POS	NEG	NEG	NEG
2012_178_Z3500 (197/12)	NEG	NEG	NEG	NEG	NEG	NEG	NEG	NEG	NEG	NEG	NEG	NEG	NEG	NEG	NEG	NEG	NEG	POS	NEG	NEG	NEG	NEG	NEG	NEG	NEG	NEG	POS	NEG	NEG	NEG
2012_183_Z3454	NEG	NEG	NEG	NEG	NEG	NEG	NEG	NEG	NEG	NEG	NEG	NEG	NEG	NEG	NEG	NEG	NEG	POS	NEG	NEG	NEG	NEG	NEG	NEG	NEG	NEG	POS	NEG	NEG	NEG
2012_186_Z3503 (444/12)	NEG	NEG	NEG	NEG	NEG	NEG	NEG	NEG	NEG	NEG	NEG	NEG	NEG	NEG	NEG	NEG	NEG	POS	NEG	NEG	NEG	NEG	NEG	NEG	NEG	NEG	POS	NEG	NEG	NEG
2012_189_Z3506 (542/12)	NEG	NEG	NEG	NEG	NEG	NEG	NEG	NEG	NEG	NEG	NEG	NEG	NEG	NEG	NEG	NEG	NEG	POS	NEG	NEG	NEG	NEG	NEG	NEG	NEG	NEG	POS	NEG	NEG	NEG
2012_192_Z3457 (606/12)	NEG	NEG	NEG	NEG	NEG	NEG	NEG	NEG	NEG	NEG	NEG	NEG	NEG	NEG	NEG	NEG	NEG	POS	NEG	NEG	NEG	NEG	NEG	NEG	NEG	NEG	POS	NEG	NEG	NEG
2012_196_Z3509 (707/12)	NEG	NEG	NEG	NEG	NEG	NEG	NEG	NEG	NEG	NEG	NEG	NEG	NEG	NEG	NEG	NEG	NEG	POS	NEG	NEG	NEG	NEG	NEG	NEG	NEG	NEG	POS	NEG	NEG	NEG
2012_218_Z3476 (1432/12)	NEG	NEG	NEG	NEG	NEG	NEG	NEG	NEG	NEG	NEG	NEG	NEG	NEG	NEG	NEG	NEG	NEG	POS	NEG	NEG	NEG	NEG	NEG	NEG	NEG	NEG	POS	NEG	NEG	NEG
2012_232_Z3520 (1892/12)	NEG	NEG	NEG	NEG	NEG	NEG	NEG	NEG	NEG	NEG	NEG	NEG	NEG	NEG	NEG	NEG	NEG	POS	AMB	NEG	NEG	NEG	NEG	NEG	NEG	NEG	POS	NEG	NEG	NEG
2012_278_Z3565 (4397/12)	NEG	NEG	NEG	NEG	NEG	NEG	NEG	NEG	NEG	NEG	NEG	NEG	NEG	NEG	NEG	NEG	NEG	POS	AMB	NEG	NEG	NEG	NEG	NEG	NEG	NEG	POS	NEG	NEG	NEG
2012_285_Z3572 (4802/12)	NEG	NEG	NEG	NEG	NEG	NEG	NEG	NEG	NEG	NEG	NEG	NEG	NEG	NEG	NEG	NEG	NEG	POS	NEG	NEG	NEG	NEG	NEG	NEG	NEG	NEG	POS	NEG	NEG	NEG
2012_287_Z3574 (4906/12)	NEG	NEG	NEG	NEG	NEG	NEG	NEG	NEG	NEG	NEG	NEG	NEG	NEG	NEG	NEG	NEG	NEG	POS	NEG	NEG	NEG	NEG	NEG	NEG	NEG	NEG	POS	NEG	NEG	NEG
2012_295_Z3582 (5116/12)	NEG	NEG	NEG	NEG	NEG	NEG	NEG	NEG	NEG	NEG	NEG	NEG	NEG	NEG	NEG	NEG	NEG	POS	NEG	NEG	NEG	NEG	NEG	NEG	NEG	NEG	POS	NEG	NEG	NEG
2012_326_Z3613 (7321/12)	NEG	NEG	NEG	NEG	NEG	NEG	NEG	NEG	NEG	NEG	NEG	NEG	NEG	NEG	NEG	NEG	NEG	POS	NEG	NEG	NEG	NEG	NEG	NEG	NEG	NEG	POS	NEG	NEG	NEG
2012_356_Z3643 (1204-15897)	NEG	NEG	NEG	NEG	NEG	NEG	NEG	NEG	NEG	NEG	NEG	NEG	NEG	NEG	NEG	NEG	NEG	POS	NEG	NEG	NEG	NEG	NEG	NEG	NEG	NEG	POS	NEG	NEG	NEG
2012_366_Z3426 (1204-16124)	NEG	NEG	NEG	NEG	NEG	NEG	NEG	NEG	NEG	NEG	NEG	NEG	NEG	NEG	NEG	NEG	NEG	POS	AMB	NEG	NEG	NEG	NEG	NEG	NEG	NEG	POS	NEG	NEG	NEG
2012_372_Z3432 (1205-19821)	NEG	NEG	NEG	NEG	NEG	NEG	NEG	NEG	NEG	NEG	NEG	NEG	NEG	NEG	NEG	NEG	NEG	POS	AMB	NEG	NEG	NEG	NEG	NEG	NEG	NEG	POS	NEG	NEG	NEG
2012_380_Z3440 (4141)	NEG	NEG	NEG	NEG	NEG	NEG	NEG	NEG	NEG	NEG	NEG	NEG	NEG	NEG	NEG	NEG	NEG	POS	AMB	NEG	NEG	NEG	NEG	NEG	NEG	NEG	POS	NEG	NEG	NEG
2013_PA20_Z4003	NEG	NEG	NEG	NEG	NEG	NEG	NEG	NEG	NEG	NEG	NEG	NEG	NEG	NEG	NEG	NEG	NEG	POS	NEG	NEG	NEG	NEG	NEG	NEG	NEG	NEG	POS	NEG	NEG	NEG
2013_PA36_Z4019	NEG	NEG	NEG	NEG	NEG	NEG	NEG	NEG	NEG	NEG	NEG	NEG	NEG	NEG	NEG	NEG	NEG	POS	NEG	NEG	NEG	NEG	NEG	NEG	NEG	NEG	POS	NEG	NEG	NEG
2013_PA46_Z4029	NEG	NEG	NEG	NEG	NEG	NEG	NEG	NEG	NEG	NEG	NEG	NEG	NEG	NEG	NEG	NEG	NEG	POS	NEG	NEG	NEG	NEG	NEG	NEG	NEG	NEG	POS	NEG	NEG	NEG
2013_PA50_Z4033	NEG	POS	NEG	NEG	NEG	NEG	NEG	POS	NEG	NEG	NEG	NEG	NEG	NEG	NEG	NEG	NEG	POS	NEG	NEG	NEG	NEG	NEG	NEG	NEG	NEG	POS	NEG	NEG	NEG
2013_PA63_Z4046	NEG	NEG	NEG	NEG	NEG	NEG	NEG	NEG	NEG	NEG	NEG	NEG	NEG	NEG	NEG	NEG	NEG	POS	NEG	NEG	NEG	NEG	NEG	NEG	NEG	NEG	POS	NEG	NEG	NEG
2013_PA78_Z4061	NEG	NEG	NEG	NEG	NEG	NEG	NEG	NEG	NEG	NEG	NEG	NEG	NEG	NEG	NEG	NEG	NEG	POS	NEG	NEG	NEG	NEG	NEG	NEG	NEG	NEG	POS	NEG	NEG	NEG
2013_PA79_Z4062	NEG	NEG	NEG	NEG	NEG	NEG	NEG	NEG	NEG	NEG	NEG	NEG	NEG	NEG	NEG	NEG	NEG	POS	NEG	NEG	NEG	NEG	NEG	NEG	NEG	NEG	POS	NEG	NEG	NEG
**CC45-MSSA**																														
2012_172_Z3449 (49/12)	NEG	NEG	NEG	NEG	AMB	NEG	NEG	NEG	NEG	NEG	NEG	NEG	NEG	NEG	NEG	NEG	NEG	AMB	NEG	NEG	NEG	NEG	NEG	NEG	NEG	NEG	POS	NEG	NEG	NEG
2012_208_Z3468 (1011/12)	NEG	NEG	NEG	NEG	NEG	NEG	NEG	NEG	NEG	NEG	NEG	NEG	NEG	NEG	NEG	NEG	NEG	NEG	NEG	NEG	NEG	NEG	NEG	NEG	NEG	NEG	POS	NEG	NEG	NEG
2012_235_Z3522 (2085/12)	NEG	NEG	NEG	NEG	NEG	NEG	NEG	NEG	NEG	NEG	NEG	NEG	NEG	NEG	NEG	NEG	NEG	AMB	NEG	NEG	NEG	NEG	NEG	NEG	NEG	NEG	POS	NEG	NEG	NEG
2012_290_Z3577 (4921/12)	NEG	NEG	NEG	NEG	NEG	NEG	NEG	NEG	NEG	NEG	NEG	NEG	NEG	NEG	NEG	NEG	NEG	NEG	NEG	NEG	NEG	NEG	NEG	NEG	NEG	NEG	POS	NEG	NEG	NEG
2012_311_Z3598 (6182/12)	NEG	NEG	NEG	NEG	NEG	NEG	NEG	NEG	NEG	NEG	NEG	NEG	NEG	NEG	NEG	NEG	NEG	NEG	NEG	NEG	NEG	NEG	NEG	NEG	NEG	NEG	POS	NEG	NEG	NEG
Z3604_TT317 - {DC558C18-2959-4049-B8F3-507365	NEG	NEG	NEG	NEG	NEG	NEG	NEG	NEG	NEG	NEG	NEG	NEG	NEG	NEG	NEG	NEG	NEG	NEG	NEG	NEG	NEG	NEG	NEG	NEG	NEG	NEG	POS	NEG	NEG	NEG
2012_363_Z3423 (1112-61058)	NEG	NEG	NEG	NEG	NEG	NEG	NEG	NEG	NEG	NEG	NEG	NEG	NEG	NEG	NEG	NEG	NEG	AMB	NEG	NEG	NEG	NEG	NEG	NEG	NEG	NEG	POS	NEG	NEG	NEG
2013_PA19_Z4002	NEG	NEG	NEG	NEG	NEG	NEG	NEG	NEG	NEG	NEG	NEG	NEG	NEG	NEG	NEG	NEG	NEG	NEG	NEG	NEG	NEG	NEG	NEG	NEG	NEG	NEG	POS	NEG	NEG	NEG
2013_PA54_Z4037	NEG	NEG	NEG	NEG	NEG	NEG	NEG	NEG	NEG	NEG	NEG	NEG	NEG	NEG	NEG	NEG	NEG	NEG	NEG	NEG	POS	POS	NEG	NEG	NEG	NEG	POS	NEG	NEG	NEG
2013_PA82_Z4065	NEG	NEG	NEG	NEG	NEG	NEG	NEG	NEG	NEG	NEG	NEG	NEG	NEG	NEG	NEG	NEG	NEG	NEG	NEG	NEG	NEG	NEG	NEG	NEG	NEG	NEG	POS	NEG	NEG	NEG
**CC97-MSSA**																														
2012_182_Z3453 (279A/12)	NEG	NEG	NEG	NEG	NEG	NEG	NEG	NEG	NEG	NEG	NEG	NEG	NEG	NEG	NEG	NEG	NEG	AMB	NEG	NEG	NEG	NEG	NEG	NEG	NEG	NEG	POS	NEG	NEG	NEG
2012_187_Z3504	NEG	NEG	NEG	NEG	NEG	NEG	NEG	NEG	NEG	NEG	NEG	NEG	NEG	NEG	NEG	NEG	NEG	NEG	NEG	NEG	NEG	NEG	NEG	NEG	NEG	NEG	POS	NEG	NEG	NEG
2012_197_Z3510 (757/12)	NEG	NEG	NEG	NEG	NEG	NEG	NEG	NEG	NEG	NEG	NEG	NEG	NEG	NEG	NEG	NEG	NEG	AMB	NEG	NEG	NEG	NEG	NEG	NEG	NEG	NEG	POS	NEG	NEG	NEG
2012_199_Z3516 (785/12)	NEG	NEG	NEG	NEG	NEG	NEG	NEG	NEG	NEG	NEG	NEG	NEG	NEG	NEG	NEG	NEG	NEG	NEG	NEG	NEG	NEG	NEG	NEG	NEG	NEG	NEG	POS	NEG	NEG	NEG
2013_PA15_Z3998	NEG	NEG	NEG	NEG	NEG	NEG	NEG	NEG	NEG	NEG	NEG	NEG	NEG	NEG	NEG	NEG	NEG	NEG	NEG	NEG	NEG	NEG	NEG	NEG	NEG	NEG	POS	NEG	NEG	NEG
**CC101-MSSA**																														
2012_283_Z3570 (4725/12)	NEG	NEG	NEG	NEG	NEG	NEG	NEG	NEG	NEG	NEG	NEG	NEG	NEG	NEG	NEG	NEG	NEG	POS	NEG	NEG	NEG	NEG	NEG	NEG	NEG	NEG	POS	NEG	NEG	NEG
2012_247_Z3531 (2788/12)	NEG	NEG	NEG	NEG	NEG	NEG	NEG	NEG	NEG	NEG	NEG	NEG	NEG	NEG	NEG	NEG	NEG	POS	NEG	NEG	NEG	NEG	NEG	NEG	NEG	NEG	POS	NEG	NEG	NEG
2012_279_Z3566 (4506/12)	NEG	NEG	NEG	NEG	NEG	NEG	NEG	NEG	NEG	NEG	NEG	NEG	NEG	NEG	NEG	NEG	NEG	POS	AMB	NEG	NEG	NEG	NEG	NEG	NEG	NEG	POS	NEG	NEG	NEG

Strain / Isolates	bifunctional enzyme Aac/Aph, gentamicin resistance	amino-glycoside adenyl-transferase, tobramycin resistance	3'5'-aminog-lycoside phospho-transferase, neo-/ kanamycin resistance	streptothricine-acetyl-transferase	dihydrofolate reductase type 1	fusidic acid resistence	hypothetical protein associated with fusidic acid resistance	mupirocin resistence protein	tetrazyklin-resistance	tetracycline-resistance	chloramphenicol acetyltransferase						chloramphenicol/florfenicol exporter	metallothiol transferase		quaternary ammonium compound resistance protein A	quaternary ammonium compound resistance protein C						Transport-/ Effluxprotein	vancomycin resistance gene	vancomycin resistance gene from enterococci and Clostridium	teicoplanin resistance gene from enterococci
	aacA-aphD	aadD	aphA3	sat	dfrS1	far1	fusC (Q6GD50)	mupA	tet(K)	tet(M)	cat (total)	cat (pC221)	cat (pc223)	cat (pMC524/pC 194)	cat (pSBK203R)	cfr	fexA	fosB	fosB (plasmid)	qacA	qacC (total)	qacC (consensus)	qacC (equine)	qacC (SA5)	qacC (Ssap)	qacC (ST94)	sdrM	vanA	vanB	vanZ
**CC121-MSSA**																														
2012_163_Z3446 (26495/11)	NEG	NEG	NEG	NEG	NEG	NEG	NEG	NEG	NEG	NEG	NEG	NEG	NEG	NEG	NEG	NEG	NEG	POS	AMB	NEG	NEG	NEG	NEG	NEG	NEG	NEG	POS	NEG	NEG	NEG
2012_204_Z3464 (938/12)	NEG	NEG	NEG	NEG	NEG	NEG	NEG	NEG	NEG	NEG	NEG	NEG	NEG	NEG	NEG	NEG	NEG	POS	AMB	NEG	NEG	NEG	NEG	NEG	NEG	NEG	POS	NEG	NEG	NEG
2012_225_Z3482 (1563/12)	NEG	NEG	NEG	NEG	NEG	NEG	NEG	NEG	NEG	NEG	NEG	NEG	NEG	NEG	NEG	NEG	NEG	POS	AMB	NEG	NEG	NEG	NEG	NEG	NEG	NEG	POS	NEG	NEG	NEG
2012_228_Z3485 (1700B/12)	NEG	NEG	NEG	NEG	NEG	NEG	NEG	NEG	NEG	NEG	NEG	NEG	NEG	NEG	NEG	NEG	NEG	POS	AMB	NEG	NEG	NEG	NEG	NEG	NEG	NEG	POS	NEG	NEG	NEG
2012_229_Z3486 (1737/12)	NEG	NEG	NEG	NEG	NEG	NEG	NEG	NEG	NEG	NEG	NEG	NEG	NEG	NEG	NEG	NEG	NEG	POS	AMB	NEG	NEG	NEG	NEG	NEG	NEG	NEG	POS	NEG	NEG	NEG
2012_302_Z3589 (5795/12)	NEG	NEG	NEG	NEG	NEG	NEG	NEG	NEG	NEG	NEG	NEG	NEG	NEG	NEG	NEG	NEG	NEG	POS	NEG	NEG	NEG	NEG	NEG	NEG	NEG	NEG	POS	NEG	NEG	NEG
2012_359_Z3419	NEG	NEG	NEG	NEG	NEG	NEG	NEG	NEG	NEG	NEG	NEG	NEG	NEG	NEG	NEG	NEG	NEG	POS	NEG	NEG	NEG	NEG	NEG	NEG	NEG	NEG	POS	NEG	NEG	NEG
2012_362_Z3422 (1201-996)	NEG	NEG	NEG	NEG	NEG	NEG	NEG	NEG	NEG	NEG	NEG	NEG	NEG	NEG	NEG	NEG	NEG	POS	AMB	NEG	NEG	NEG	NEG	NEG	NEG	NEG	POS	NEG	NEG	NEG
2012_367_Z3427 (1204-16413)	NEG	NEG	NEG	NEG	NEG	NEG	NEG	NEG	NEG	NEG	NEG	NEG	NEG	NEG	NEG	NEG	NEG	POS	AMB	NEG	NEG	NEG	NEG	NEG	NEG	NEG	POS	NEG	NEG	NEG
2013_PA09_Z3992	NEG	NEG	NEG	NEG	NEG	NEG	NEG	POS	NEG	NEG	POS	POS	NEG	NEG	NEG	NEG	NEG	POS	NEG	NEG	NEG	NEG	NEG	NEG	NEG	NEG	POS	NEG	NEG	NEG
2013_PA59_Z4042	NEG	NEG	NEG	NEG	NEG	NEG	NEG	NEG	NEG	NEG	NEG	NEG	NEG	NEG	NEG	NEG	NEG	POS	NEG	NEG	NEG	NEG	NEG	NEG	NEG	NEG	POS	NEG	NEG	NEG
2013_PA60_Z4043	NEG	NEG	NEG	NEG	NEG	NEG	NEG	NEG	NEG	NEG	NEG	NEG	NEG	NEG	NEG	NEG	NEG	POS	NEG	NEG	NEG	NEG	NEG	NEG	NEG	NEG	POS	NEG	NEG	NEG
**CC152-MSSA [PVL+]**																														
2012_190_Z3507 (549/12)	NEG	NEG	NEG	NEG	NEG	NEG	NEG	NEG	NEG	NEG	NEG	NEG	NEG	NEG	NEG	NEG	NEG	NEG	NEG	NEG	NEG	NEG	NEG	NEG	NEG	NEG	POS	NEG	NEG	NEG
2012_201_Z3461 (823/12)	NEG	NEG	NEG	NEG	NEG	NEG	NEG	NEG	NEG	NEG	NEG	NEG	NEG	NEG	NEG	NEG	NEG	NEG	NEG	NEG	NEG	NEG	NEG	NEG	NEG	NEG	POS	NEG	NEG	NEG
2012_209_Z3469 (1116/12)	NEG	NEG	NEG	NEG	NEG	NEG	NEG	NEG	NEG	NEG	NEG	NEG	NEG	NEG	NEG	NEG	NEG	NEG	NEG	NEG	NEG	NEG	NEG	NEG	NEG	NEG	POS	NEG	NEG	NEG
2012_211_Z3517 (1238/12)	NEG	NEG	NEG	NEG	NEG	NEG	NEG	NEG	NEG	NEG	NEG	NEG	NEG	NEG	NEG	NEG	NEG	NEG	NEG	NEG	NEG	NEG	NEG	NEG	NEG	NEG	POS	NEG	NEG	NEG
2012_213_Z3472 (1320/12)	NEG	NEG	NEG	NEG	NEG	NEG	NEG	NEG	NEG	NEG	NEG	NEG	NEG	NEG	NEG	NEG	NEG	NEG	NEG	NEG	NEG	NEG	NEG	NEG	NEG	NEG	POS	NEG	NEG	NEG
2012_221_Z3478	NEG	NEG	NEG	NEG	NEG	NEG	NEG	NEG	NEG	NEG	NEG	NEG	NEG	NEG	NEG	NEG	NEG	NEG	NEG	NEG	NEG	NEG	NEG	NEG	NEG	NEG	POS	NEG	NEG	NEG
2012_234_Z3521 (1981/12)	NEG	NEG	NEG	NEG	NEG	NEG	NEG	NEG	NEG	NEG	NEG	NEG	NEG	NEG	NEG	NEG	NEG	NEG	NEG	NEG	NEG	NEG	NEG	NEG	NEG	NEG	POS	NEG	NEG	NEG
2012_238_Z3524 (2199/12)	NEG	NEG	NEG	NEG	NEG	NEG	NEG	NEG	NEG	NEG	NEG	NEG	NEG	NEG	NEG	NEG	NEG	NEG	NEG	NEG	NEG	NEG	NEG	NEG	NEG	NEG	POS	NEG	NEG	NEG
2012_260_Z3547 (3290/12)	NEG	NEG	AMB	NEG	AMB	NEG	NEG	NEG	NEG	NEG	NEG	NEG	NEG	NEG	NEG	NEG	NEG	NEG	NEG	NEG	NEG	NEG	NEG	NEG	NEG	NEG	POS	NEG	NEG	NEG
2012_272_Z3559 (3802/12)	NEG	NEG	NEG	NEG	NEG	NEG	NEG	NEG	NEG	NEG	NEG	NEG	NEG	NEG	NEG	NEG	NEG	NEG	NEG	NEG	NEG	NEG	NEG	NEG	NEG	NEG	POS	NEG	NEG	NEG
2012_289_Z3576 (4912/12)	NEG	NEG	NEG	NEG	NEG	NEG	NEG	NEG	NEG	NEG	NEG	NEG	NEG	NEG	NEG	NEG	NEG	NEG	NEG	NEG	NEG	NEG	NEG	NEG	NEG	NEG	POS	NEG	NEG	NEG
2012_309_Z3596	NEG	NEG	NEG	NEG	NEG	NEG	NEG	NEG	NEG	NEG	NEG	NEG	NEG	NEG	NEG	NEG	NEG	NEG	NEG	NEG	NEG	NEG	NEG	NEG	NEG	NEG	POS	NEG	NEG	NEG
2012_322_Z3609 (7178/12)	NEG	NEG	NEG	NEG	NEG	NEG	NEG	NEG	NEG	NEG	NEG	NEG	NEG	NEG	NEG	NEG	NEG	NEG	NEG	NEG	NEG	NEG	NEG	NEG	NEG	NEG	POS	NEG	NEG	NEG
2012_325_Z3612 (7254/12)	NEG	NEG	NEG	NEG	NEG	NEG	NEG	NEG	NEG	NEG	NEG	NEG	NEG	NEG	NEG	NEG	NEG	NEG	NEG	NEG	NEG	NEG	NEG	NEG	NEG	NEG	POS	NEG	NEG	NEG
2012_328_Z3615 (7456/12)	NEG	NEG	NEG	NEG	NEG	NEG	NEG	NEG	NEG	NEG	NEG	NEG	NEG	NEG	NEG	NEG	NEG	NEG	NEG	NEG	NEG	NEG	NEG	NEG	NEG	NEG	POS	NEG	NEG	NEG
2012_334_Z3621 (8267/12)	NEG	NEG	NEG	NEG	NEG	NEG	NEG	NEG	NEG	NEG	NEG	NEG	NEG	NEG	NEG	NEG	NEG	NEG	NEG	NEG	NEG	NEG	NEG	NEG	NEG	NEG	POS	NEG	NEG	NEG
2012_360_Z3420 (1201-4047)	NEG	NEG	NEG	NEG	NEG	NEG	NEG	NEG	NEG	NEG	NEG	NEG	NEG	NEG	NEG	NEG	NEG	AMB	NEG	NEG	NEG	NEG	NEG	NEG	NEG	NEG	POS	NEG	NEG	NEG
2012_382_Z3442 (4086)	NEG	NEG	NEG	NEG	NEG	NEG	NEG	NEG	NEG	NEG	NEG	NEG	NEG	NEG	NEG	NEG	NEG	NEG	NEG	NEG	NEG	NEG	NEG	NEG	NEG	NEG	POS	NEG	NEG	NEG
2013_PA02_Z3985	NEG	NEG	NEG	NEG	NEG	NEG	NEG	NEG	NEG	NEG	NEG	NEG	NEG	NEG	NEG	NEG	NEG	NEG	NEG	NEG	NEG	NEG	NEG	NEG	NEG	NEG	POS	NEG	NEG	NEG
2013_PA03_Z3986	NEG	NEG	NEG	NEG	NEG	NEG	NEG	NEG	NEG	NEG	NEG	NEG	NEG	NEG	NEG	NEG	NEG	NEG	NEG	NEG	NEG	NEG	NEG	NEG	NEG	NEG	POS	NEG	NEG	NEG
2013_PA07_Z3990	NEG	NEG	NEG	NEG	NEG	NEG	NEG	NEG	NEG	NEG	NEG	NEG	NEG	NEG	NEG	NEG	NEG	NEG	NEG	NEG	NEG	NEG	NEG	NEG	NEG	NEG	POS	NEG	NEG	NEG
2013_PA34_Z4017	NEG	NEG	NEG	NEG	NEG	NEG	NEG	NEG	NEG	NEG	NEG	NEG	NEG	NEG	NEG	NEG	NEG	NEG	NEG	NEG	NEG	NEG	NEG	NEG	NEG	NEG	POS	NEG	NEG	NEG
2013_PA40_Z4023	NEG	NEG	NEG	NEG	NEG	NEG	NEG	NEG	NEG	NEG	NEG	NEG	NEG	NEG	NEG	NEG	NEG	NEG	NEG	NEG	NEG	NEG	NEG	NEG	NEG	NEG	POS	NEG	NEG	NEG
2013_PA48_Z4031	NEG	NEG	NEG	NEG	NEG	NEG	NEG	NEG	NEG	NEG	NEG	NEG	NEG	NEG	NEG	NEG	NEG	NEG	NEG	NEG	NEG	NEG	NEG	NEG	NEG	NEG	POS	NEG	NEG	NEG
2013_PA49_Z4032	NEG	NEG	NEG	NEG	NEG	NEG	NEG	NEG	NEG	NEG	NEG	NEG	NEG	NEG	NEG	NEG	NEG	NEG	NEG	NEG	NEG	NEG	NEG	NEG	NEG	NEG	POS	NEG	NEG	NEG
2013_PA51_Z4034	NEG	NEG	NEG	NEG	NEG	NEG	NEG	NEG	NEG	NEG	NEG	NEG	NEG	NEG	NEG	NEG	NEG	NEG	NEG	NEG	NEG	NEG	NEG	NEG	NEG	NEG	POS	NEG	NEG	NEG
2013_PA70_Z4053	NEG	NEG	NEG	NEG	NEG	NEG	NEG	NEG	NEG	NEG	NEG	NEG	NEG	NEG	NEG	NEG	NEG	NEG	NEG	NEG	NEG	NEG	NEG	NEG	NEG	NEG	POS	NEG	NEG	NEG
2013_PA84_Z4067	NEG	NEG	NEG	NEG	NEG	NEG	NEG	NEG	NEG	NEG	NEG	NEG	NEG	NEG	NEG	NEG	NEG	NEG	NEG	NEG	NEG	NEG	NEG	NEG	NEG	NEG	POS	NEG	NEG	NEG
**CC188-MSSA**																														
2012_206_Z3466 (968/12)	NEG	NEG	NEG	NEG	NEG	NEG	NEG	NEG	NEG	NEG	NEG	NEG	NEG	NEG	NEG	NEG	NEG	NEG	NEG	NEG	NEG	NEG	NEG	NEG	NEG	NEG	POS	NEG	NEG	NEG
2012_251_Z3534 (3026/12)	NEG	NEG	NEG	NEG	NEG	NEG	NEG	NEG	NEG	NEG	NEG	NEG	NEG	NEG	NEG	NEG	NEG	NEG	NEG	NEG	NEG	NEG	NEG	NEG	NEG	NEG	POS	NEG	NEG	NEG
2012_320_Z3607 (7144/12)	NEG	NEG	NEG	NEG	NEG	NEG	NEG	NEG	NEG	NEG	NEG	NEG	NEG	NEG	NEG	NEG	NEG	NEG	NEG	NEG	NEG	NEG	NEG	NEG	NEG	NEG	POS	NEG	NEG	NEG
2012_321_Z3608 (7147/12)	NEG	NEG	NEG	NEG	NEG	NEG	NEG	NEG	POS	NEG	NEG	NEG	NEG	NEG	NEG	NEG	NEG	NEG	NEG	NEG	NEG	NEG	NEG	NEG	NEG	NEG	POS	NEG	NEG	NEG
2012_358_Z3418 (1202-6332)	NEG	NEG	NEG	NEG	NEG	NEG	NEG	NEG	NEG	NEG	NEG	NEG	NEG	NEG	NEG	NEG	NEG	NEG	NEG	NEG	POS	POS	NEG	NEG	AMB	NEG	POS	NEG	NEG	NEG
**CC398-MSSA**																														
2013_PA11_Z3994	NEG	NEG	NEG	NEG	NEG	NEG	NEG	NEG	NEG	NEG	NEG	NEG	NEG	NEG	NEG	NEG	NEG	NEG	NEG	NEG	NEG	NEG	NEG	NEG	NEG	NEG	POS	NEG	NEG	NEG
2013_PA57_Z4040	NEG	NEG	NEG	NEG	NEG	NEG	NEG	NEG	NEG	NEG	NEG	NEG	NEG	NEG	NEG	NEG	NEG	NEG	NEG	NEG	NEG	NEG	NEG	NEG	NEG	NEG	POS	NEG	NEG	NEG
**ST1852-MSSA**																														
2012_277_Z3564 (4050/12)	NEG	NEG	NEG	NEG	NEG	NEG	NEG	NEG	NEG	NEG	NEG	NEG	NEG	NEG	NEG	NEG	NEG	NEG	NEG	NEG	NEG	NEG	NEG	NEG	NEG	NEG	POS	NEG	NEG	NEG
**CC1223/1594-MSSA**																														
2012_337_Z3624 (8070B/12)	NEG	NEG	NEG	NEG	NEG	NEG	NEG	NEG	NEG	NEG	NEG	NEG	NEG	NEG	NEG	NEG	NEG	POS	NEG	NEG	NEG	NEG	NEG	NEG	NEG	NEG	AMB	NEG	NEG	NEG
**MSSA related to ST2250/2277**																														
2012_203_Z3463 (829/12)	NEG	NEG	AMB	NEG	NEG	NEG	NEG	NEG	POS	NEG	NEG	NEG	NEG	NEG	NEG	NEG	NEG	POS	POS	NEG	NEG	NEG	NEG	NEG	NEG	NEG	POS	NEG	NEG	NEG
2012_269_Z3556 (3769A/12)	NEG	NEG	NEG	NEG	NEG	NEG	NEG	NEG	NEG	NEG	NEG	NEG	NEG	NEG	NEG	NEG	NEG	POS	NEG	NEG	NEG	NEG	NEG	NEG	NEG	NEG	NEG	NEG	NEG	NEG
2012_292_Z3579	NEG	NEG	NEG	NEG	NEG	NEG	NEG	NEG	NEG	NEG	NEG	NEG	NEG	NEG	NEG	NEG	NEG	POS	NEG	NEG	NEG	NEG	NEG	NEG	NEG	NEG	NEG	NEG	NEG	NEG
2012_299_Z3586 (5487/12)	NEG	NEG	NEG	NEG	NEG	NEG	NEG	NEG	NEG	NEG	NEG	NEG	NEG	NEG	NEG	NEG	NEG	POS	NEG	NEG	NEG	NEG	NEG	NEG	NEG	NEG	NEG	NEG	NEG	NEG
2012_301_Z3588 (5717/12)	NEG	NEG	NEG	NEG	NEG	NEG	NEG	NEG	POS	NEG	NEG	NEG	NEG	NEG	NEG	NEG	NEG	POS	NEG	NEG	NEG	NEG	NEG	NEG	NEG	NEG	NEG	NEG	NEG	NEG
**ST2596dlv-MSSA**																														
2012_310_Z3597 (6034/12)	NEG	NEG	NEG	NEG	NEG	NEG	NEG	NEG	NEG	NEG	NEG	NEG	NEG	NEG	NEG	NEG	NEG	NEG	NEG	NEG	NEG	NEG	NEG	NEG	NEG	NEG	NEG	NEG	NEG	NEG

	toxic shock syndrome toxin 1	enterotoxin A	enterotoxin A, allele from strain N315 = enterotoxin P	enterotoxin B	enterotoxin C	enterotoxin D	enterotoxin E	enterotoxin G	enterotoxin H	enterotoxin I	enterotoxin J	enterotoxin K	enterotoxin L	enterotoxin M	enterotoxin N		enterotoxin O	egc cluster	enterotoxin Q	enterotoxin R	Enterotoxin U and/or Y	enterotoxin-like protein ORF CM14	haemolysin gamma / leukocidin, component B	haemolysin gamma / leukocidin, component C		ysin gamma, comp	Panton Valentine leukocidin F component	Panton Valentine leukocidin S component	hypothetical leukocidin from ruminants	leukocidin D component	leukocidin E component
Strain / Isolates	tst1	sea	sea (N315) / sep	seb	sec	sed	see	seg	seh	sei	sej	sek	sel	selm	seln (consensus)	seln (other than RF122)	selo	egc (total)	seq	ser	selu	ORF CM14	lukF	lukS	lukS (ST22+ST45)	hlgA	lukF-PV	lukS-PV	lukF-PV (P83)/ lukM	lukD	lukE
**CC1-MSSA**																															
2012_257_Z3544 (3216/12)	NEG	NEG	NEG	POS	NEG	NEG	NEG	NEG	POS	NEG	NEG	NEG	NEG	NEG	NEG	NEG	NEG	NEG	NEG	NEG	NEG	NEG	POS	POS	POS	POS	NEG	NEG	NEG	POS	POS
2012_281_Z3568 (4696/12)	NEG	NEG	NEG	NEG	NEG	NEG	NEG	NEG	POS	NEG	NEG	NEG	NEG	NEG	NEG	NEG	NEG	NEG	NEG	NEG	NEG	NEG	POS	POS	POS	POS	NEG	NEG	NEG	POS	POS
2012_324_Z3611 (7180/12)	NEG	POS	NEG	POS	NEG	NEG	NEG	NEG	POS	NEG	NEG	POS	NEG	NEG	NEG	NEG	NEG	NEG	POS	NEG	NEG	NEG	POS	POS	POS	POS	NEG	NEG	NEG	POS	POS
2012_327_Z3614 (7347/12)	NEG	NEG	NEG	POS	NEG	NEG	NEG	NEG	POS	NEG	NEG	NEG	NEG	NEG	NEG	NEG	NEG	NEG	NEG	NEG	NEG	NEG	POS	POS	POS	POS	NEG	NEG	NEG	POS	POS
2012_351_Z3638 (8273B/12)	NEG	NEG	NEG	POS	NEG	NEG	NEG	NEG	POS	NEG	NEG	NEG	NEG	NEG	NEG	NEG	NEG	NEG	NEG	NEG	NEG	NEG	POS	POS	POS	POS	NEG	NEG	NEG	POS	POS
**CC5-MSSA**																															
2012_188_Z3505 (503/12)	NEG	NEG	NEG	NEG	NEG	NEG	NEG	POS	NEG	POS	NEG	NEG	NEG	POS	POS	POS	POS	POS	NEG	NEG	POS	NEG	POS	POS	POS	POS	NEG	NEG	NEG	POS	POS
2012_219_Z3518 (1437/12)	NEG	NEG	POS	NEG	NEG	NEG	NEG	POS	NEG	POS	NEG	NEG	NEG	POS	POS	POS	POS	POS	NEG	NEG	POS	NEG	POS	POS	POS	POS	NEG	NEG	NEG	POS	POS
2012_329_Z3616 (7577/12)	NEG	NEG	POS	NEG	NEG	NEG	NEG	POS	NEG	POS	NEG	NEG	NEG	POS	POS	POS	POS	POS	NEG	NEG	POS	NEG	POS	POS	POS	POS	NEG	NEG	NEG	POS	POS
2012_377_Z3437 (6242)	NEG	NEG	POS	NEG	NEG	NEG	NEG	POS	NEG	POS	POS	NEG	NEG	POS	POS	POS	POS	POS	NEG	POS	POS	NEG	POS	POS	POS	POS	NEG	NEG	NEG	POS	POS
2013_PA04_Z3987	NEG	NEG	NEG	NEG	NEG	NEG	NEG	POS	NEG	POS	NEG	NEG	NEG	POS	POS	POS	POS	POS	NEG	NEG	POS	NEG	POS	POS	POS	POS	NEG	NEG	NEG	POS	POS
2013_PA32_Z4015	POS	NEG	NEG	NEG	POS	POS	NEG	POS	NEG	POS	POS	NEG	POS	POS	POS	POS	POS	POS	NEG	POS	POS	NEG	POS	POS	POS	POS	NEG	NEG	NEG	POS	POS
2013_PA41_Z4024	POS	NEG	POS	NEG	NEG	POS	NEG	POS	NEG	POS	POS	NEG	NEG	POS	POS	POS	POS	POS	NEG	POS	POS	NEG	POS	POS	POS	POS	NEG	NEG	NEG	POS	POS
2013_PA44_Z4027	POS	NEG	POS	NEG	NEG	POS	NEG	POS	NEG	POS	POS	NEG	NEG	POS	POS	POS	POS	POS	NEG	POS	POS	NEG	POS	POS	POS	POS	NEG	NEG	NEG	POS	POS
**CC5-MSSA [PVL+]**																															
2012_175_Z3497 (112/12)	NEG	NEG	NEG	NEG	NEG	NEG	NEG	POS	NEG	POS	NEG	NEG	NEG	POS	POS	POS	POS	POS	NEG	NEG	POS	NEG	POS	POS	POS	POS	POS	POS	NEG	POS	POS
2012_193_Z3458 (617/12)	NEG	NEG	NEG	NEG	NEG	NEG	NEG	POS	NEG	POS	NEG	NEG	NEG	POS	POS	POS	POS	POS	NEG	NEG	POS	NEG	POS	POS	POS	POS	POS	POS	NEG	POS	POS
2012_215_Z3474 (1341/12)	NEG	NEG	NEG	NEG	NEG	NEG	NEG	POS	NEG	POS	NEG	NEG	NEG	POS	POS	POS	POS	POS	NEG	NEG	POS	NEG	POS	POS	POS	POS	POS	POS	NEG	POS	POS
2012_216_Z3475 (1370/12)	NEG	NEG	NEG	NEG	NEG	NEG	NEG	POS	NEG	POS	NEG	NEG	NEG	POS	POS	POS	POS	POS	NEG	NEG	POS	NEG	POS	POS	POS	POS	POS	POS	NEG	POS	POS
2012_297_Z3584 (5140/12)	NEG	NEG	NEG	NEG	NEG	NEG	NEG	POS	NEG	POS	NEG	NEG	NEG	POS	POS	POS	POS	POS	NEG	NEG	POS	NEG	POS	POS	POS	POS	POS	POS	NEG	POS	POS
2012_331_Z3618 (7644/12)	NEG	NEG	NEG	NEG	NEG	NEG	NEG	POS	NEG	POS	NEG	NEG	NEG	POS	POS	POS	POS	POS	NEG	NEG	POS	NEG	POS	POS	POS	POS	POS	POS	NEG	POS	POS
2012_379_Z3439 (4126)	NEG	NEG	NEG	NEG	NEG	NEG	NEG	POS	NEG	POS	NEG	NEG	NEG	POS	POS	POS	POS	POS	NEG	NEG	POS	NEG	POS	POS	POS	POS	POS	POS	NEG	POS	POS
2012_381_Z3441	NEG	NEG	NEG	NEG	NEG	NEG	NEG	POS	NEG	POS	NEG	NEG	NEG	POS	POS	POS	POS	POS	NEG	NEG	POS	NEG	POS	POS	POS	POS	POS	POS	NEG	POS	POS
2013_PA39_Z4022	NEG	NEG	NEG	NEG	NEG	NEG	NEG	POS	NEG	POS	NEG	NEG	NEG	POS	POS	POS	POS	POS	NEG	NEG	POS	NEG	POS	POS	POS	POS	POS	POS	NEG	POS	POS
2013_PA47_Z4030	NEG	NEG	NEG	NEG	NEG	NEG	NEG	POS	NEG	POS	NEG	NEG	NEG	POS	POS	POS	POS	POS	NEG	NEG	POS	NEG	POS	POS	AMB	POS	POS	POS	NEG	POS	POS
2013_PA61_I_Z4044	NEG	NEG	NEG	NEG	NEG	NEG	NEG	POS	NEG	POS	NEG	NEG	NEG	POS	POS	POS	POS	POS	NEG	NEG	POS	NEG	POS	POS	POS	POS	POS	POS	NEG	POS	POS
2013_PA62_I_Z4045	NEG	NEG	NEG	NEG	NEG	NEG	NEG	POS	NEG	POS	NEG	NEG	NEG	POS	POS	POS	POS	POS	NEG	NEG	POS	NEG	POS	POS	POS	POS	POS	POS	NEG	POS	POS
2013_PA74_Z4057	NEG	NEG	NEG	NEG	NEG	NEG	NEG	POS	NEG	POS	NEG	NEG	NEG	POS	POS	POS	POS	POS	NEG	NEG	POS	NEG	POS	POS	AMB	POS	POS	POS	NEG	POS	POS
2013_PA75_Z4058	NEG	NEG	NEG	NEG	NEG	NEG	NEG	POS	NEG	POS	NEG	NEG	NEG	POS	POS	POS	POS	POS	NEG	NEG	POS	NEG	POS	POS	AMB	POS	POS	POS	NEG	POS	POS
**CC5-MRSA-V [ACME+]**																															
2012_373_Z3433 (1204-17312)	NEG	NEG	NEG	NEG	NEG	NEG	NEG	POS	NEG	POS	NEG	NEG	NEG	POS	POS	POS	POS	POS	NEG	NEG	POS	NEG	POS	POS	POS	POS	NEG	NEG	NEG	POS	POS
**CC6-MSSA**																															
2012_173_Z3450 (98A/12)	NEG	POS	NEG	NEG	NEG	NEG	NEG	NEG	NEG	NEG	NEG	NEG	NEG	NEG	NEG	NEG	NEG	NEG	NEG	NEG	NEG	NEG	POS	POS	POS	POS	NEG	NEG	NEG	POS	POS
2012_176_Z3498	NEG	POS	NEG	NEG	NEG	NEG	NEG	NEG	NEG	NEG	NEG	NEG	NEG	NEG	NEG	NEG	NEG	NEG	NEG	NEG	NEG	NEG	POS	POS	POS	POS	NEG	NEG	NEG	POS	POS
2012_181_Z3452	NEG	POS	NEG	NEG	NEG	NEG	NEG	NEG	NEG	NEG	NEG	NEG	NEG	NEG	NEG	NEG	NEG	NEG	NEG	NEG	NEG	NEG	POS	POS	POS	POS	NEG	NEG	NEG	POS	POS
2012_259_Z3546 (3279/12)	NEG	POS	NEG	NEG	NEG	NEG	NEG	NEG	NEG	NEG	NEG	NEG	POS	NEG	NEG	NEG	NEG	NEG	NEG	NEG	NEG	NEG	POS	POS	POS	POS	NEG	NEG	NEG	POS	POS
2012_264_Z3551 (3407B/12)	NEG	POS	NEG	NEG	NEG	NEG	NEG	NEG	NEG	NEG	NEG	NEG	NEG	NEG	NEG	NEG	NEG	NEG	NEG	NEG	NEG	NEG	POS	POS	POS	POS	NEG	NEG	NEG	POS	POS
2012_273_Z3560 (3844/12)	NEG	POS	NEG	NEG	NEG	NEG	NEG	NEG	NEG	NEG	NEG	NEG	NEG	NEG	NEG	NEG	NEG	NEG	NEG	NEG	NEG	NEG	POS	POS	POS	POS	NEG	NEG	NEG	POS	POS
2012_312_Z3599 (6815/12)	NEG	POS	NEG	NEG	NEG	NEG	NEG	NEG	NEG	NEG	NEG	NEG	NEG	NEG	NEG	NEG	NEG	NEG	NEG	NEG	NEG	NEG	POS	POS	POS	POS	NEG	NEG	NEG	POS	POS
2012_338_Z3625	NEG	POS	NEG	NEG	NEG	NEG	NEG	NEG	NEG	NEG	NEG	NEG	NEG	NEG	NEG	NEG	NEG	NEG	NEG	NEG	NEG	NEG	POS	POS	POS	POS	NEG	NEG	NEG	POS	POS
2012_352_Z3639	NEG	POS	NEG	NEG	NEG	NEG	NEG	NEG	NEG	NEG	NEG	NEG	NEG	NEG	NEG	NEG	NEG	NEG	NEG	NEG	NEG	NEG	POS	POS	POS	POS	NEG	NEG	NEG	POS	POS
2012_368_Z3428	NEG	POS	NEG	NEG	NEG	NEG	NEG	NEG	NEG	NEG	NEG	NEG	NEG	NEG	NEG	NEG	NEG	NEG	NEG	NEG	NEG	NEG	POS	POS	POS	POS	NEG	NEG	NEG	POS	POS
2013_PA12_Z3995	NEG	POS	NEG	NEG	NEG	NEG	NEG	NEG	NEG	NEG	NEG	NEG	NEG	NEG	NEG	NEG	NEG	NEG	NEG	NEG	NEG	NEG	POS	POS	POS	POS	NEG	NEG	NEG	POS	POS
2013_PA28_Z4011	NEG	POS	NEG	NEG	NEG	NEG	NEG	NEG	NEG	NEG	NEG	NEG	NEG	NEG	NEG	NEG	NEG	NEG	NEG	NEG	NEG	NEG	POS	POS	POS	POS	NEG	NEG	NEG	POS	POS
**CC6-MSSA [PVL+]**																															
2012_375_Z3435	NEG	POS	NEG	NEG	NEG	NEG	NEG	NEG	NEG	NEG	NEG	NEG	NEG	NEG	NEG	NEG	NEG	NEG	NEG	NEG	NEG	NEG	POS	POS	POS	POS	POS	POS	NEG	POS	POS
2013_PA77_Z4060	NEG	POS	NEG	NEG	NEG	NEG	NEG	NEG	NEG	NEG	NEG	NEG	NEG	NEG	NEG	NEG	NEG	NEG	NEG	NEG	NEG	NEG	POS	POS	POS	POS	POS	POS	NEG	POS	POS
**CC7-MSSA**																															
2012_248_Z3532 (2864/12)	NEG	NEG	POS	NEG	NEG	NEG	NEG	NEG	NEG	NEG	NEG	NEG	NEG	NEG	NEG	NEG	NEG	NEG	NEG	NEG	NEG	NEG	POS	POS	POS	POS	NEG	NEG	NEG	POS	POS
2012_265_Z3552 (3518/12)	NEG	AMB	POS	NEG	NEG	NEG	NEG	NEG	NEG	NEG	NEG	NEG	NEG	NEG	NEG	NEG	NEG	NEG	NEG	NEG	NEG	NEG	POS	POS	POS	POS	NEG	NEG	NEG	POS	POS
2013_PA53_Z4036	NEG	NEG	POS	NEG	NEG	NEG	NEG	NEG	NEG	NEG	NEG	NEG	NEG	NEG	NEG	NEG	NEG	NEG	NEG	NEG	NEG	NEG	POS	POS	POS	POS	NEG	NEG	NEG	POS	POS
2013_PA83_Z4066	NEG	NEG	POS	NEG	NEG	NEG	NEG	NEG	NEG	NEG	NEG	NEG	NEG	NEG	NEG	NEG	NEG	NEG	NEG	NEG	NEG	NEG	POS	POS	POS	POS	NEG	NEG	NEG	POS	POS
**CC8-MSSA**																															
2012_243_Z3528 (2587/12)	NEG	NEG	POS	NEG	NEG	POS	NEG	NEG	NEG	NEG	POS	POS	NEG	NEG	NEG	NEG	NEG	NEG	POS	POS	NEG	NEG	POS	POS	POS	POS	NEG	NEG	NEG	POS	POS
2012_344_Z3631 (8605/12)	NEG	NEG	NEG	NEG	NEG	NEG	NEG	NEG	NEG	NEG	NEG	NEG	NEG	NEG	NEG	NEG	NEG	NEG	NEG	NEG	NEG	NEG	POS	POS	POS	POS	NEG	NEG	NEG	POS	POS
2012_346_Z3633 (8074/12)	NEG	NEG	POS	NEG	NEG	NEG	NEG	NEG	NEG	NEG	NEG	NEG	NEG	NEG	NEG	NEG	NEG	NEG	NEG	NEG	NEG	NEG	POS	POS	POS	POS	NEG	NEG	NEG	POS	POS
2012_255_Z3538 (3205B/12)	NEG	NEG	NEG	NEG	NEG	NEG	NEG	NEG	NEG	NEG	NEG	NEG	NEG	NEG	NEG	NEG	NEG	NEG	NEG	NEG	NEG	NEG	POS	POS	POS	POS	NEG	NEG	NEG	POS	POS
2012_280_Z3567 (4601/12)	NEG	NEG	NEG	NEG	NEG	NEG	NEG	NEG	NEG	NEG	NEG	NEG	NEG	NEG	NEG	NEG	NEG	NEG	NEG	NEG	NEG	NEG	POS	POS	POS	POS	NEG	NEG	NEG	POS	POS
Trinidad&Tobago_2012_267 (3642/12)	NEG	NEG	NEG	NEG	NEG	NEG	NEG	NEG	NEG	NEG	NEG	NEG	NEG	NEG	NEG	NEG	NEG	NEG	NEG	NEG	NEG	NEG	POS	POS	POS	POS	NEG	NEG	NEG	POS	POS
2012_365_Z3425 (1204-15838)	POS	NEG	NEG	NEG	NEG	NEG	NEG	NEG	NEG	NEG	NEG	NEG	NEG	NEG	NEG	NEG	NEG	NEG	NEG	NEG	NEG	NEG	POS	POS	POS	POS	NEG	NEG	NEG	POS	POS
2012_296_Z3583 (5118/12)	NEG	NEG	NEG	NEG	NEG	NEG	NEG	NEG	NEG	NEG	NEG	NEG	NEG	NEG	NEG	NEG	NEG	NEG	NEG	NEG	NEG	NEG	POS	POS	POS	POS	NEG	NEG	NEG	POS	POS
2012_318_Z3605 (6960/12)	NEG	NEG	NEG	NEG	NEG	NEG	NEG	NEG	NEG	NEG	NEG	NEG	NEG	NEG	NEG	NEG	NEG	NEG	NEG	NEG	NEG	NEG	POS	POS	POS	POS	NEG	NEG	NEG	POS	POS
2012_306_Z3593 (5861/12)	NEG	NEG	NEG	NEG	NEG	NEG	NEG	NEG	NEG	NEG	NEG	NEG	NEG	NEG	NEG	NEG	NEG	NEG	NEG	NEG	NEG	NEG	POS	POS	POS	POS	NEG	NEG	NEG	POS	POS
2013_PA37_Z4020	NEG	NEG	NEG	NEG	NEG	NEG	NEG	NEG	NEG	NEG	NEG	NEG	NEG	NEG	NEG	NEG	NEG	NEG	NEG	NEG	NEG	NEG	POS	POS	POS	POS	NEG	NEG	NEG	POS	POS
2013_PA38_Z4021	NEG	NEG	NEG	NEG	NEG	NEG	NEG	NEG	NEG	NEG	NEG	NEG	NEG	NEG	NEG	NEG	NEG	NEG	NEG	NEG	NEG	NEG	POS	POS	NEG	POS	NEG	NEG	NEG	POS	POS
2013_PA52_Z4035	NEG	NEG	NEG	NEG	NEG	NEG	NEG	NEG	NEG	NEG	NEG	POS	NEG	NEG	NEG	NEG	NEG	NEG	AMB	NEG	NEG	NEG	POS	POS	NEG	POS	NEG	NEG	NEG	POS	POS
**CC8-MSSA [PVL+]**																															
2012_156_Z3489 (25917/11)	NEG	NEG	NEG	NEG	NEG	POS	NEG	NEG	NEG	NEG	POS	POS	NEG	NEG	NEG	NEG	NEG	NEG	POS	POS	NEG	NEG	POS	POS	POS	POS	POS	POS	NEG	POS	POS
2012_159_Z3492 (26310/11)	NEG	NEG	NEG	NEG	NEG	POS	NEG	NEG	NEG	NEG	POS	POS	NEG	NEG	NEG	NEG	NEG	NEG	POS	POS	NEG	NEG	POS	POS	POS	POS	POS	POS	NEG	POS	POS
2012_161_Z3445 (26430/11)	NEG	NEG	NEG	NEG	NEG	POS	NEG	NEG	NEG	NEG	POS	POS	NEG	NEG	NEG	NEG	NEG	NEG	POS	POS	NEG	NEG	POS	POS	POS	POS	POS	POS	NEG	POS	POS
2012_162_Z3512 (26433/11)	NEG	NEG	NEG	NEG	NEG	POS	NEG	NEG	NEG	NEG	POS	POS	NEG	NEG	NEG	NEG	NEG	NEG	POS	POS	NEG	NEG	POS	POS	POS	POS	POS	POS	NEG	POS	POS
2012_169_Z3495 (27194/11)	NEG	NEG	NEG	NEG	NEG	NEG	NEG	NEG	NEG	NEG	NEG	POS	NEG	NEG	NEG	NEG	NEG	NEG	POS	NEG	NEG	NEG	POS	POS	POS	POS	POS	POS	NEG	POS	POS
2012_170_Z3496 (35/12)	NEG	NEG	NEG	NEG	NEG	POS	NEG	NEG	NEG	NEG	POS	POS	NEG	NEG	NEG	NEG	NEG	NEG	POS	POS	NEG	NEG	POS	POS	POS	POS	POS	POS	NEG	POS	POS
2012_171_Z3448 (47/12)	NEG	NEG	NEG	NEG	NEG	POS	NEG	NEG	NEG	NEG	POS	POS	NEG	NEG	NEG	NEG	NEG	NEG	POS	POS	NEG	NEG	POS	POS	POS	POS	POS	POS	NEG	POS	POS
2012_177_Z3499 (184/12)	NEG	NEG	NEG	NEG	NEG	POS	NEG	NEG	NEG	NEG	POS	POS	NEG	NEG	NEG	NEG	NEG	NEG	POS	POS	NEG	NEG	POS	POS	POS	POS	POS	POS	NEG	POS	POS
2012_179_Z3515 (231/12)	NEG	NEG	NEG	NEG	NEG	POS	NEG	NEG	NEG	NEG	POS	POS	NEG	NEG	NEG	NEG	NEG	NEG	POS	POS	NEG	NEG	POS	POS	POS	POS	POS	POS	NEG	POS	POS
2012_185_Z3502 (437/12)	NEG	NEG	NEG	NEG	NEG	POS	NEG	NEG	NEG	NEG	POS	POS	NEG	NEG	NEG	NEG	NEG	NEG	POS	POS	NEG	NEG	POS	POS	POS	POS	POS	POS	NEG	POS	POS
2012_191_Z3456 (563/12)	NEG	NEG	NEG	NEG	NEG	POS	NEG	NEG	NEG	NEG	POS	POS	NEG	NEG	NEG	NEG	NEG	NEG	POS	POS	NEG	NEG	POS	POS	POS	POS	POS	POS	NEG	POS	POS
2012_194_Z3459	NEG	NEG	NEG	NEG	NEG	POS	NEG	NEG	NEG	NEG	POS	POS	NEG	NEG	NEG	NEG	NEG	NEG	POS	POS	NEG	NEG	POS	POS	POS	POS	POS	POS	NEG	POS	POS
2012_200_Z3460 (820/12)	NEG	NEG	NEG	NEG	NEG	POS	NEG	NEG	NEG	NEG	POS	POS	NEG	NEG	NEG	NEG	NEG	NEG	POS	POS	NEG	NEG	POS	POS	POS	POS	POS	POS	NEG	POS	POS
2012_207_Z3467 (1010/12)	NEG	NEG	NEG	NEG	NEG	POS	NEG	NEG	NEG	NEG	POS	POS	NEG	NEG	NEG	NEG	NEG	NEG	POS	POS	NEG	NEG	POS	POS	POS	POS	POS	POS	NEG	POS	POS
2012_212_Z3471 (1261/12)	NEG	NEG	NEG	NEG	NEG	POS	NEG	NEG	NEG	NEG	POS	POS	NEG	NEG	NEG	NEG	NEG	NEG	POS	POS	NEG	NEG	POS	POS	POS	POS	POS	POS	NEG	POS	POS
2012_220_Z3477 (1466/12)	NEG	NEG	NEG	NEG	NEG	POS	NEG	NEG	NEG	NEG	POS	POS	NEG	NEG	NEG	NEG	NEG	NEG	POS	POS	NEG	NEG	POS	POS	POS	POS	POS	POS	NEG	POS	POS
2012_224_Z3481 (1522/12)	NEG	NEG	NEG	NEG	NEG	POS	NEG	NEG	NEG	NEG	POS	POS	NEG	NEG	NEG	NEG	NEG	NEG	POS	POS	NEG	NEG	POS	POS	POS	POS	POS	POS	NEG	POS	POS
2012_226_Z3483 (1662/12)	NEG	NEG	NEG	NEG	NEG	POS	NEG	NEG	NEG	NEG	POS	POS	NEG	NEG	NEG	NEG	NEG	NEG	POS	POS	NEG	NEG	POS	POS	POS	POS	POS	POS	NEG	POS	POS
2012_242_Z3527 (2581/12)	NEG	NEG	NEG	NEG	NEG	POS	NEG	NEG	NEG	NEG	POS	POS	NEG	NEG	NEG	NEG	NEG	NEG	POS	POS	NEG	NEG	POS	POS	POS	POS	POS	POS	NEG	POS	POS
2012_245_Z3529 (2687/12)	NEG	NEG	NEG	NEG	NEG	POS	NEG	NEG	NEG	NEG	POS	POS	NEG	NEG	NEG	NEG	NEG	NEG	POS	POS	NEG	NEG	POS	POS	POS	POS	POS	POS	NEG	POS	POS
2012_253_Z3536 (3074/12)	NEG	NEG	NEG	NEG	NEG	POS	NEG	NEG	NEG	NEG	POS	POS	NEG	NEG	NEG	NEG	NEG	NEG	POS	POS	NEG	NEG	POS	POS	POS	POS	POS	POS	NEG	POS	POS
2012_258_Z3545 (3231/12)	NEG	NEG	NEG	NEG	NEG	POS	NEG	NEG	NEG	NEG	POS	POS	NEG	NEG	NEG	NEG	NEG	NEG	POS	POS	NEG	NEG	POS	POS	POS	POS	POS	POS	NEG	POS	POS
2012_261_Z3548 (3298/12)	NEG	NEG	NEG	NEG	NEG	POS	NEG	NEG	NEG	NEG	POS	POS	NEG	NEG	NEG	NEG	NEG	NEG	POS	POS	NEG	NEG	POS	POS	POS	POS	POS	POS	NEG	POS	POS
2012_270_Z3557 (3798/12)	NEG	NEG	NEG	NEG	NEG	POS	NEG	NEG	NEG	NEG	POS	POS	NEG	NEG	NEG	NEG	NEG	NEG	POS	POS	NEG	NEG	POS	POS	POS	POS	POS	POS	NEG	POS	POS
2012_274_Z3561 (3861/12)	NEG	NEG	NEG	NEG	NEG	POS	NEG	NEG	NEG	NEG	POS	POS	NEG	NEG	NEG	NEG	NEG	NEG	POS	POS	NEG	NEG	POS	POS	POS	POS	POS	POS	NEG	POS	POS
2012_282_Z3569 (4698/12)	NEG	NEG	NEG	NEG	NEG	POS	NEG	NEG	NEG	NEG	POS	POS	NEG	NEG	NEG	NEG	NEG	NEG	POS	POS	NEG	NEG	POS	POS	POS	POS	POS	POS	NEG	POS	POS
2012_286_Z3573 (4805/12)	NEG	NEG	NEG	NEG	NEG	POS	NEG	NEG	NEG	NEG	POS	POS	NEG	NEG	NEG	NEG	NEG	NEG	POS	POS	NEG	NEG	POS	POS	POS	POS	POS	POS	NEG	POS	POS
2012_293_Z3580 (5007/12)	NEG	NEG	NEG	NEG	NEG	POS	NEG	NEG	NEG	NEG	POS	POS	NEG	NEG	NEG	NEG	NEG	NEG	POS	POS	NEG	NEG	POS	POS	POS	POS	POS	POS	NEG	POS	POS
2012_294_Z3581	NEG	NEG	NEG	NEG	NEG	POS	NEG	NEG	NEG	NEG	POS	POS	NEG	NEG	NEG	NEG	NEG	NEG	POS	POS	NEG	NEG	POS	POS	POS	POS	POS	POS	NEG	POS	POS
2012_307_Z3594 (5918/12)	NEG	NEG	NEG	NEG	NEG	POS	NEG	NEG	NEG	NEG	POS	POS	NEG	NEG	NEG	NEG	NEG	NEG	POS	POS	NEG	NEG	POS	POS	POS	POS	POS	POS	NEG	POS	POS
2012_333_Z3620 (8101/12)	NEG	NEG	NEG	NEG	NEG	POS	NEG	NEG	NEG	NEG	POS	POS	NEG	NEG	NEG	NEG	NEG	NEG	POS	POS	NEG	NEG	POS	POS	POS	POS	POS	POS	NEG	POS	POS
2012_343_Z3630 (8195/12)	NEG	NEG	NEG	NEG	NEG	POS	NEG	NEG	NEG	NEG	POS	POS	NEG	NEG	NEG	NEG	NEG	NEG	POS	POS	NEG	NEG	POS	POS	POS	POS	POS	POS	NEG	POS	POS
2012_349_Z3636 (7999A/12)	NEG	NEG	NEG	NEG	NEG	POS	NEG	NEG	NEG	NEG	POS	POS	NEG	NEG	NEG	NEG	NEG	NEG	POS	POS	NEG	NEG	POS	POS	POS	POS	POS	POS	NEG	POS	POS
2012_353_Z3640 (8575/12)	NEG	NEG	NEG	NEG	NEG	POS	NEG	NEG	NEG	NEG	POS	POS	NEG	NEG	NEG	NEG	NEG	NEG	POS	POS	NEG	NEG	POS	POS	POS	POS	POS	POS	NEG	POS	POS
2012_354_Z3641 (8408/12)	NEG	NEG	NEG	NEG	NEG	POS	NEG	NEG	NEG	NEG	POS	POS	NEG	NEG	NEG	NEG	NEG	NEG	POS	POS	NEG	NEG	POS	POS	POS	POS	POS	POS	NEG	POS	POS
2012_355_Z3642 (1112-58348)	NEG	NEG	NEG	NEG	NEG	POS	NEG	NEG	NEG	NEG	POS	POS	NEG	NEG	NEG	NEG	NEG	NEG	POS	POS	NEG	NEG	POS	POS	POS	POS	POS	POS	NEG	POS	POS
2012_357_Z3417 (1202-6360)	NEG	NEG	NEG	NEG	NEG	NEG	NEG	NEG	NEG	NEG	NEG	POS	NEG	NEG	NEG	NEG	NEG	NEG	POS	NEG	NEG	NEG	POS	POS	POS	POS	POS	POS	NEG	POS	POS
2012_364_Z3424 (1112-60417)	NEG	NEG	NEG	NEG	NEG	POS	NEG	NEG	NEG	NEG	POS	POS	NEG	NEG	NEG	NEG	NEG	NEG	POS	POS	NEG	NEG	POS	POS	POS	POS	POS	POS	NEG	POS	POS
2012_376_Z3436 (6256)	NEG	NEG	NEG	NEG	NEG	POS	NEG	NEG	NEG	NEG	POS	POS	NEG	NEG	NEG	NEG	NEG	NEG	POS	POS	NEG	NEG	POS	POS	POS	POS	POS	POS	NEG	POS	POS
2012_383_Z3443	NEG	NEG	NEG	NEG	NEG	POS	NEG	NEG	NEG	NEG	POS	POS	NEG	NEG	NEG	NEG	NEG	NEG	POS	POS	NEG	NEG	POS	POS	POS	POS	POS	POS	NEG	POS	POS
2013_PA01_Z3984	NEG	NEG	NEG	NEG	NEG	POS	NEG	NEG	NEG	NEG	POS	POS	NEG	NEG	NEG	NEG	NEG	NEG	POS	POS	NEG	NEG	POS	POS	POS	POS	POS	POS	NEG	POS	POS
2013_PA08_Z3991	NEG	NEG	NEG	NEG	NEG	POS	NEG	NEG	NEG	NEG	POS	POS	NEG	NEG	NEG	NEG	NEG	NEG	POS	POS	NEG	NEG	POS	POS	POS	POS	POS	POS	NEG	POS	POS
2013_PA10_Z3993	NEG	NEG	NEG	NEG	NEG	POS	NEG	NEG	NEG	NEG	POS	POS	NEG	NEG	NEG	NEG	NEG	NEG	POS	POS	NEG	NEG	POS	POS	POS	POS	POS	POS	NEG	POS	POS
2013_PA13_Z3996	NEG	NEG	NEG	NEG	NEG	POS	NEG	NEG	NEG	NEG	POS	POS	NEG	NEG	NEG	NEG	NEG	NEG	POS	POS	NEG	NEG	POS	POS	POS	POS	POS	POS	NEG	POS	POS
2013_PA14_Z3997	NEG	NEG	NEG	NEG	NEG	POS	NEG	NEG	NEG	NEG	POS	POS	NEG	NEG	NEG	NEG	NEG	NEG	POS	POS	NEG	NEG	POS	POS	POS	POS	POS	POS	NEG	POS	POS
2013_PA17_Z4000	NEG	NEG	NEG	NEG	NEG	POS	NEG	NEG	NEG	NEG	POS	POS	NEG	NEG	NEG	NEG	NEG	NEG	POS	POS	NEG	NEG	POS	POS	POS	POS	POS	POS	NEG	POS	POS
2013_PA26_Z4009	NEG	NEG	NEG	NEG	NEG	POS	NEG	NEG	NEG	NEG	POS	POS	NEG	NEG	NEG	NEG	NEG	NEG	POS	POS	NEG	NEG	POS	POS	POS	POS	POS	POS	NEG	POS	POS
2013_PA27_Z4010	NEG	NEG	NEG	NEG	NEG	POS	NEG	NEG	NEG	NEG	POS	POS	NEG	NEG	NEG	NEG	NEG	NEG	POS	POS	NEG	NEG	POS	POS	POS	POS	POS	POS	NEG	POS	POS
2013_PA42_Z4025	NEG	NEG	NEG	NEG	NEG	POS	NEG	NEG	NEG	NEG	POS	POS	NEG	NEG	NEG	NEG	NEG	NEG	POS	POS	NEG	NEG	POS	POS	POS	POS	POS	POS	NEG	POS	POS
2013_PA43_Z4026	NEG	NEG	NEG	NEG	NEG	POS	NEG	NEG	NEG	NEG	POS	POS	NEG	NEG	NEG	NEG	NEG	NEG	POS	POS	NEG	NEG	POS	POS	AMB	POS	POS	POS	NEG	POS	POS
2013_PA45_Z4028	NEG	NEG	NEG	NEG	NEG	POS	NEG	NEG	NEG	NEG	POS	POS	NEG	NEG	NEG	NEG	NEG	NEG	POS	POS	NEG	NEG	POS	POS	POS	POS	POS	POS	NEG	POS	POS
2013_PA56_Z4039	NEG	NEG	NEG	NEG	NEG	POS	NEG	NEG	NEG	NEG	POS	POS	NEG	NEG	NEG	NEG	NEG	NEG	POS	POS	NEG	NEG	POS	POS	POS	POS	POS	POS	NEG	POS	POS
2013_PA58_Z4041	NEG	NEG	NEG	NEG	NEG	POS	NEG	NEG	NEG	NEG	POS	POS	NEG	NEG	NEG	NEG	NEG	NEG	POS	POS	NEG	NEG	POS	POS	POS	POS	POS	POS	NEG	POS	POS
2013_PA64_Z4047	NEG	NEG	NEG	NEG	NEG	POS	NEG	NEG	NEG	NEG	POS	POS	NEG	NEG	NEG	NEG	NEG	NEG	POS	POS	NEG	NEG	POS	POS	POS	POS	POS	POS	NEG	POS	POS
2013_PA66_Z4049	NEG	NEG	NEG	NEG	NEG	POS	NEG	NEG	NEG	NEG	POS	POS	NEG	NEG	NEG	NEG	NEG	NEG	POS	POS	NEG	NEG	POS	POS	POS	POS	POS	POS	NEG	POS	POS
2013_PA67_Z4050	NEG	NEG	NEG	NEG	NEG	POS	NEG	NEG	NEG	NEG	POS	POS	NEG	NEG	NEG	NEG	NEG	NEG	POS	POS	NEG	NEG	POS	POS	POS	POS	POS	POS	NEG	POS	POS
2013_PA69_Z4052	NEG	NEG	NEG	NEG	NEG	POS	NEG	NEG	NEG	NEG	POS	POS	NEG	NEG	NEG	NEG	NEG	NEG	POS	POS	NEG	NEG	POS	POS	AMB	POS	POS	POS	NEG	POS	POS
2013_PA72_Z4055	NEG	NEG	NEG	NEG	NEG	POS	NEG	NEG	NEG	NEG	POS	POS	NEG	NEG	NEG	NEG	NEG	NEG	POS	POS	NEG	NEG	POS	POS	POS	POS	POS	POS	NEG	POS	POS
2013_PA76_Z4059	NEG	NEG	NEG	NEG	NEG	POS	NEG	NEG	NEG	NEG	POS	POS	NEG	NEG	NEG	NEG	NEG	NEG	POS	POS	NEG	NEG	POS	POS	POS	POS	POS	POS	NEG	POS	POS
2013_PA80_Z4063	NEG	NEG	NEG	NEG	NEG	POS	NEG	NEG	NEG	NEG	POS	POS	NEG	NEG	NEG	NEG	NEG	NEG	POS	POS	NEG	NEG	POS	POS	POS	POS	POS	POS	NEG	POS	POS

Strain / Isolates	toxic shock syndrome toxin 1	enterotoxin A	enterotoxin A, allele from strain N315 = entero-toxin P	enterotoxin B	enterotoxin C	enterotoxin D	enterotoxin E	enterotoxin G	enterotoxin H	enterotoxin I	enterotoxin J	enterotoxin K	enterotoxin L	enterotoxin M	enterotoxin N		enterotoxin O	egc cluster	enterotoxin Q	enterotoxin R	Enterotoxin U and/or Y	enterotoxin-like protein ORF CM14	haemolysin gamma / leukocidin, component B	haemolysin gamma / leukocidin, component C		ysin gamma, comp	Panton Valentine leukocidin F component	Panton Valentine leukocidin S component	hypothetical leukocidin from ruminants	leukocidin D component	leukocidin E component
	tst1	sea	sea (N315) / sep	seb	sec	sed	see	seg	seh	sei	sej	sek	sel	selm	seln (consensus)	seln (other than RF122)	selo	egc (total)	seq	ser	selu	ORF CM14	lukF	lukS	lukS (ST22+ST45)	hlgA	lukF-PV	lukS-PV	lukF-PV (P83)/ lukM	lukD	lukE
**ST8-MRSA-IV, USA300**																															
2012_158_Z3491 (26049A/11)	NEG	NEG	NEG	NEG	NEG	NEG	NEG	NEG	NEG	NEG	NEG	POS	NEG	NEG	NEG	NEG	NEG	NEG	POS	NEG	NEG	NEG	POS	POS	POS	POS	POS	POS	NEG	POS	POS
2012_165_Z3513 (26656/11)	NEG	NEG	NEG	NEG	NEG	NEG	NEG	NEG	NEG	NEG	NEG	POS	NEG	NEG	NEG	NEG	NEG	NEG	POS	NEG	NEG	NEG	POS	POS	POS	POS	POS	POS	NEG	POS	POS
2012_214_Z3473 (1322/12)	NEG	NEG	NEG	NEG	NEG	NEG	NEG	NEG	NEG	NEG	NEG	POS	NEG	NEG	NEG	NEG	NEG	NEG	POS	NEG	NEG	NEG	POS	POS	POS	POS	POS	POS	NEG	POS	POS
2012_227_Z3484 (1688/12)	NEG	NEG	NEG	NEG	NEG	NEG	NEG	NEG	NEG	NEG	NEG	POS	NEG	NEG	NEG	NEG	NEG	NEG	POS	NEG	NEG	NEG	POS	POS	POS	POS	POS	POS	NEG	POS	POS
2012_263_Z3550 (3355/12)	NEG	NEG	NEG	NEG	NEG	NEG	NEG	NEG	NEG	NEG	NEG	POS	NEG	NEG	NEG	NEG	NEG	NEG	POS	NEG	NEG	NEG	POS	POS	POS	POS	POS	POS	NEG	POS	POS
2012_266_Z3553	NEG	NEG	NEG	NEG	NEG	NEG	NEG	NEG	NEG	NEG	NEG	POS	NEG	NEG	NEG	NEG	NEG	NEG	POS	NEG	NEG	NEG	POS	POS	POS	POS	POS	POS	NEG	POS	POS
2012_284_Z3571 (4729/12)	NEG	NEG	NEG	NEG	NEG	NEG	NEG	NEG	NEG	NEG	NEG	POS	NEG	NEG	NEG	NEG	NEG	NEG	POS	NEG	NEG	NEG	POS	POS	POS	POS	POS	POS	NEG	POS	POS
2012_298_Z3585 (5203/12)	NEG	NEG	NEG	NEG	NEG	NEG	NEG	NEG	NEG	NEG	NEG	POS	NEG	NEG	NEG	NEG	NEG	NEG	POS	NEG	NEG	NEG	POS	POS	POS	POS	POS	POS	NEG	POS	POS
2012_304_Z3591 (5818/12)	NEG	NEG	NEG	NEG	NEG	NEG	NEG	NEG	NEG	NEG	NEG	POS	NEG	NEG	NEG	NEG	NEG	NEG	POS	NEG	NEG	NEG	POS	POS	POS	POS	POS	POS	NEG	POS	POS
Z3595_TT308 - {EFC74B77-3925-484F-A4CF-A1F7DC	NEG	NEG	NEG	NEG	NEG	NEG	NEG	NEG	NEG	NEG	NEG	POS	NEG	NEG	NEG	NEG	NEG	NEG	POS	NEG	NEG	NEG	POS	POS	POS	POS	POS	POS	NEG	POS	POS
2012_369_Z3429 (1112-58547)	NEG	NEG	NEG	NEG	NEG	NEG	NEG	NEG	NEG	NEG	NEG	POS	NEG	NEG	NEG	NEG	NEG	NEG	POS	NEG	NEG	NEG	POS	POS	POS	POS	POS	POS	NEG	POS	POS
2012_374_Z3434 (1203-13527)	NEG	NEG	NEG	NEG	NEG	NEG	NEG	NEG	NEG	NEG	NEG	POS	NEG	NEG	NEG	NEG	NEG	NEG	POS	NEG	NEG	NEG	POS	POS	POS	POS	POS	POS	NEG	POS	POS
2013_PA06_Z3989	NEG	NEG	NEG	NEG	NEG	NEG	NEG	NEG	NEG	NEG	NEG	POS	NEG	NEG	NEG	NEG	NEG	NEG	POS	NEG	NEG	NEG	POS	POS	POS	POS	POS	POS	NEG	POS	POS
2013_PA55_Z4038	NEG	NEG	NEG	NEG	NEG	NEG	NEG	NEG	NEG	NEG	NEG	POS	NEG	NEG	NEG	NEG	NEG	NEG	POS	NEG	NEG	NEG	POS	POS	POS	POS	POS	POS	NEG	POS	POS
2013_PA71_Z4054	NEG	NEG	NEG	NEG	NEG	NEG	NEG	NEG	NEG	NEG	NEG	NEG	NEG	NEG	NEG	NEG	NEG	NEG	NEG	NEG	NEG	NEG	POS	POS	AMB	POS	POS	POS	NEG	POS	POS
**ST8-MRSA-IV [PVL+/ACME+], US**																															
2012_180_Z3501 (232/12)	NEG	NEG	NEG	NEG	NEG	NEG	NEG	NEG	NEG	NEG	NEG	POS	NEG	NEG	NEG	NEG	NEG	NEG	POS	NEG	NEG	NEG	POS	POS	POS	POS	POS	POS	NEG	POS	POS
2013_PA68_Z4051	NEG	NEG	NEG	NEG	NEG	NEG	NEG	NEG	NEG	NEG	NEG	POS	NEG	NEG	NEG	NEG	NEG	NEG	POS	NEG	NEG	NEG	POS	POS	POS	POS	POS	POS	NEG	POS	POS
**ST72-MSSA**																															
2012_174_Z3451 (102/12)	NEG	NEG	NEG	AMB	POS	NEG	NEG	POS	NEG	POS	NEG	NEG	POS	POS	POS	POS	POS	POS	NEG	NEG	POS	NEG	POS	POS	POS	POS	NEG	NEG	NEG	POS	POS
2012_223_Z3480 (1506A/12)	NEG	NEG	NEG	NEG	POS	NEG	NEG	POS	NEG	POS	NEG	NEG	POS	POS	POS	POS	POS	POS	NEG	NEG	POS	NEG	POS	POS	POS	POS	NEG	NEG	NEG	POS	POS
2012_236_Z3523 (2086/12)	NEG	NEG	NEG	AMB	POS	NEG	NEG	POS	NEG	POS	NEG	NEG	POS	POS	POS	POS	POS	POS	NEG	NEG	POS	NEG	POS	POS	POS	POS	NEG	NEG	NEG	POS	POS
2012_239_Z3525 (2346/12)	NEG	NEG	NEG	AMB	POS	NEG	NEG	POS	NEG	POS	NEG	NEG	POS	POS	POS	POS	POS	POS	NEG	NEG	POS	NEG	POS	POS	POS	POS	NEG	NEG	NEG	POS	POS
2012_252_Z3535 (3030/12)	NEG	NEG	NEG	AMB	POS	NEG	NEG	POS	NEG	POS	NEG	NEG	POS	POS	POS	POS	POS	POS	NEG	NEG	POS	NEG	POS	POS	POS	POS	NEG	NEG	NEG	POS	POS
2012_271_Z3558 (3799/12)	NEG	NEG	NEG	AMB	POS	NEG	NEG	POS	NEG	POS	NEG	NEG	POS	POS	POS	POS	POS	POS	NEG	NEG	POS	NEG	POS	POS	POS	POS	NEG	NEG	NEG	POS	POS
2012_276_Z3563	NEG	NEG	NEG	POS	POS	NEG	NEG	POS	NEG	POS	NEG	NEG	POS	POS	POS	POS	POS	POS	NEG	NEG	POS	NEG	POS	POS	POS	POS	NEG	NEG	NEG	POS	POS
2012_300_Z3587 (5638/12)	NEG	NEG	NEG	AMB	POS	NEG	NEG	POS	NEG	POS	NEG	NEG	POS	POS	POS	POS	POS	POS	NEG	NEG	POS	NEG	POS	POS	POS	POS	NEG	NEG	NEG	POS	POS
2012_316_Z3603 (6941/12)	NEG	NEG	NEG	NEG	NEG	NEG	NEG	POS	NEG	POS	NEG	NEG	NEG	POS	POS	POS	POS	POS	NEG	NEG	POS	NEG	POS	POS	POS	POS	NEG	NEG	NEG	POS	POS
2012_319_Z3606 (7019/12)	NEG	NEG	NEG	AMB	POS	NEG	NEG	POS	NEG	POS	NEG	NEG	POS	POS	POS	POS	POS	POS	NEG	NEG	POS	NEG	POS	POS	POS	POS	NEG	NEG	NEG	POS	POS
2012_371_Z3431 (1204-17577)	POS	NEG	NEG	AMB	POS	NEG	NEG	POS	NEG	POS	NEG	NEG	POS	POS	POS	POS	POS	POS	NEG	NEG	POS	NEG	POS	POS	POS	POS	NEG	NEG	NEG	POS	POS
2013_PA05_Z3988	NEG	NEG	NEG	NEG	POS	NEG	NEG	POS	NEG	POS	NEG	NEG	POS	POS	POS	POS	POS	POS	NEG	NEG	POS	NEG	POS	POS	POS	POS	NEG	NEG	NEG	POS	POS
2013_PA65_Z4048	NEG	NEG	NEG	NEG	AMB	NEG	NEG	POS	NEG	POS	NEG	NEG	AMB	POS	POS	POS	POS	POS	NEG	NEG	POS	NEG	POS	POS	AMB	POS	NEG	NEG	NEG	POS	POS
2013_PA73_Z4056	NEG	NEG	NEG	AMB	POS	NEG	NEG	POS	NEG	POS	NEG	NEG	POS	POS	POS	POS	POS	POS	NEG	NEG	POS	NEG	POS	POS	POS	POS	NEG	NEG	NEG	POS	POS
**ST72-MSSA-SCCfus**																															
2013_PA16_Z3999	NEG	NEG	NEG	NEG	POS	NEG	NEG	POS	NEG	POS	NEG	NEG	POS	POS	POS	POS	POS	POS	NEG	NEG	POS	NEG	POS	POS	POS	POS	NEG	NEG	NEG	POS	POS
**ST72-MSSA-SCCfus/kdp**																															
2012_361_Z3421 (1201-1299)	NEG	NEG	NEG	NEG	POS	NEG	NEG	POS	NEG	POS	NEG	NEG	POS	POS	POS	POS	POS	POS	NEG	NEG	POS	NEG	POS	POS	POS	POS	NEG	NEG	NEG	POS	POS
**ST239-MRSA-III&SCCmer**																															
2012_157_Z3490	NEG	POS	NEG	NEG	NEG	NEG	NEG	NEG	NEG	NEG	NEG	POS	NEG	NEG	NEG	NEG	NEG	NEG	POS	NEG	NEG	NEG	POS	POS	POS	POS	NEG	NEG	NEG	POS	POS
2012_168_Z3494 (27129/11)	NEG	POS	NEG	NEG	NEG	NEG	NEG	NEG	NEG	NEG	NEG	POS	NEG	NEG	NEG	NEG	NEG	NEG	POS	NEG	NEG	NEG	POS	POS	POS	POS	NEG	NEG	NEG	POS	POS
2012_195_Z3508 (619/12)	NEG	NEG	NEG	NEG	NEG	NEG	NEG	NEG	NEG	NEG	NEG	POS	NEG	NEG	NEG	NEG	NEG	NEG	POS	NEG	NEG	NEG	POS	POS	POS	POS	NEG	NEG	NEG	POS	POS
2012_222_Z3479 (1489/12)	NEG	POS	NEG	NEG	NEG	NEG	NEG	NEG	NEG	NEG	NEG	POS	NEG	NEG	NEG	NEG	NEG	NEG	POS	NEG	NEG	NEG	POS	POS	POS	POS	NEG	NEG	NEG	POS	POS
2012_233_Z3540 (1943/12)	NEG	POS	NEG	NEG	NEG	NEG	NEG	NEG	NEG	NEG	NEG	POS	NEG	NEG	NEG	NEG	NEG	NEG	POS	NEG	NEG	NEG	POS	POS	POS	POS	NEG	NEG	NEG	POS	POS
2012_237_Z3541	NEG	POS	NEG	NEG	NEG	NEG	NEG	NEG	NEG	NEG	NEG	POS	NEG	NEG	NEG	NEG	NEG	NEG	POS	NEG	NEG	NEG	POS	POS	POS	POS	NEG	NEG	NEG	POS	POS
2012_262_Z3549	NEG	POS	NEG	NEG	NEG	NEG	NEG	NEG	NEG	NEG	NEG	POS	NEG	NEG	NEG	NEG	NEG	NEG	POS	NEG	NEG	NEG	POS	POS	POS	POS	NEG	NEG	NEG	POS	POS
2012_275_Z3562 (3893/12)	NEG	POS	NEG	NEG	NEG	NEG	NEG	NEG	NEG	NEG	NEG	POS	NEG	NEG	NEG	NEG	NEG	NEG	POS	NEG	NEG	NEG	POS	POS	POS	POS	NEG	NEG	NEG	POS	POS
2012_291_Z3578	NEG	POS	NEG	NEG	NEG	NEG	NEG	NEG	NEG	NEG	NEG	POS	NEG	NEG	NEG	NEG	NEG	NEG	POS	NEG	NEG	NEG	POS	POS	POS	POS	NEG	NEG	NEG	POS	POS
2012_314_Z3601 (6939/12)	NEG	POS	NEG	NEG	NEG	NEG	NEG	NEG	NEG	NEG	NEG	POS	NEG	NEG	NEG	NEG	NEG	NEG	POS	NEG	NEG	NEG	POS	POS	POS	POS	NEG	AMB	NEG	POS	POS
2012323_Z3610	NEG	POS	NEG	NEG	NEG	NEG	NEG	NEG	NEG	NEG	NEG	POS	NEG	NEG	NEG	NEG	NEG	NEG	POS	NEG	NEG	NEG	POS	POS	POS	POS	NEG	NEG	NEG	POS	POS
2012330_Z3617 (7605/12)	NEG	POS	NEG	NEG	NEG	NEG	NEG	NEG	NEG	NEG	NEG	POS	NEG	NEG	NEG	NEG	NEG	NEG	POS	NEG	NEG	NEG	POS	POS	POS	POS	NEG	NEG	NEG	POS	POS
2012332_Z3619 (8076/12)	NEG	POS	NEG	NEG	NEG	NEG	NEG	NEG	NEG	NEG	NEG	POS	NEG	NEG	NEG	NEG	NEG	NEG	POS	NEG	NEG	NEG	POS	POS	POS	POS	NEG	NEG	NEG	POS	POS
2012339_Z3626 (8558/12)	NEG	POS	NEG	NEG	NEG	NEG	NEG	NEG	NEG	NEG	NEG	POS	NEG	NEG	NEG	NEG	NEG	NEG	POS	NEG	NEG	NEG	POS	POS	POS	POS	NEG	NEG	NEG	POS	POS
2012340_Z3627 (8523/12)	NEG	POS	NEG	NEG	NEG	NEG	NEG	NEG	NEG	NEG	NEG	POS	NEG	NEG	NEG	NEG	NEG	NEG	POS	NEG	NEG	NEG	POS	POS	POS	POS	NEG	NEG	NEG	POS	POS
2012348_Z3635	NEG	POS	NEG	NEG	NEG	NEG	NEG	NEG	NEG	NEG	NEG	POS	NEG	NEG	NEG	NEG	NEG	NEG	POS	NEG	NEG	NEG	POS	POS	POS	POS	NEG	NEG	NEG	POS	POS
2012350_Z3637	NEG	NEG	NEG	NEG	NEG	NEG	NEG	NEG	NEG	NEG	NEG	POS	NEG	NEG	NEG	NEG	NEG	NEG	POS	NEG	NEG	NEG	POS	POS	POS	POS	NEG	NEG	NEG	POS	POS
2012_370_Z3430 (1112-52177)	NEG	NEG	NEG	NEG	NEG	NEG	NEG	NEG	NEG	NEG	NEG	POS	NEG	NEG	NEG	NEG	NEG	NEG	POS	NEG	NEG	NEG	POS	POS	POS	POS	NEG	NEG	NEG	POS	POS
2012_378_Z3438 (4139)	NEG	POS	NEG	NEG	NEG	NEG	NEG	NEG	NEG	NEG	NEG	POS	NEG	NEG	NEG	NEG	NEG	NEG	POS	NEG	NEG	NEG	POS	POS	POS	POS	NEG	NEG	NEG	POS	POS
2013_PA21_Z4004	NEG	POS	NEG	NEG	NEG	NEG	NEG	NEG	NEG	NEG	NEG	POS	NEG	NEG	NEG	NEG	NEG	NEG	POS	NEG	NEG	NEG	POS	POS	AMB	POS	NEG	NEG	NEG	POS	POS
2013_PA22_Z4005	NEG	POS	NEG	NEG	NEG	NEG	NEG	NEG	NEG	NEG	NEG	POS	NEG	NEG	NEG	NEG	NEG	NEG	POS	NEG	NEG	NEG	POS	POS	POS	POS	NEG	NEG	NEG	POS	POS
2013_PA24_Z4007	NEG	POS	NEG	NEG	NEG	NEG	NEG	NEG	NEG	NEG	NEG	POS	NEG	NEG	NEG	NEG	NEG	NEG	POS	NEG	NEG	NEG	POS	POS	AMB	POS	NEG	NEG	NEG	POS	POS
2013_PA31_Z4014	NEG	POS	NEG	NEG	NEG	NEG	NEG	NEG	NEG	NEG	NEG	POS	NEG	NEG	NEG	NEG	NEG	NEG	POS	NEG	NEG	NEG	POS	POS	POS	POS	NEG	NEG	NEG	POS	POS
2013_PA35_Z4018	NEG	POS	NEG	NEG	NEG	NEG	NEG	NEG	NEG	NEG	NEG	POS	NEG	NEG	NEG	NEG	NEG	NEG	POS	NEG	NEG	NEG	POS	POS	NEG	POS	NEG	NEG	NEG	POS	POS
2013_PA81_Z4064	NEG	NEG	NEG	NEG	NEG	NEG	NEG	NEG	NEG	NEG	NEG	POS	NEG	NEG	NEG	NEG	NEG	NEG	POS	NEG	NEG	NEG	POS	POS	POS	POS	NEG	NEG	NEG	POS	POS
**ST239-MRSA-III+ccrA/B4**																															
2012_254_Z3537 (3124A/12)	NEG	NEG	NEG	NEG	NEG	NEG	NEG	NEG	NEG	NEG	NEG	POS	NEG	NEG	NEG	NEG	NEG	NEG	POS	NEG	NEG	NEG	POS	POS	AMB	POS	NEG	NEG	NEG	POS	POS
2013_PA25_Z4008	NEG	NEG	NEG	NEG	NEG	NEG	NEG	NEG	NEG	NEG	NEG	POS	NEG	NEG	NEG	NEG	NEG	NEG	POS	NEG	NEG	NEG	POS	POS	POS	POS	NEG	NEG	NEG	POS	POS
**ST834-MSSA**																															
2012_268_Z3555 (3693/12)	NEG	NEG	NEG	NEG	NEG	NEG	NEG	NEG	NEG	NEG	NEG	NEG	NEG	NEG	NEG	NEG	NEG	NEG	NEG	NEG	NEG	NEG	POS	POS	POS	POS	NEG	NEG	NEG	POS	POS
**CC12-MSSA**																															
2012_210_Z3470 (1177/12)	NEG	NEG	POS	POS	NEG	NEG	NEG	NEG	NEG	NEG	NEG	NEG	NEG	NEG	NEG	NEG	NEG	NEG	NEG	NEG	NEG	POS	POS	POS	POS	POS	NEG	NEG	NEG	POS	POS
2012_230_Z3487 (1797/12)	NEG	NEG	POS	POS	NEG	NEG	NEG	NEG	NEG	NEG	NEG	NEG	NEG	NEG	NEG	NEG	NEG	NEG	NEG	NEG	NEG	POS	POS	POS	POS	POS	NEG	NEG	NEG	POS	POS
2013_PA18_Z4001	NEG	NEG	POS	POS	NEG	NEG	NEG	NEG	NEG	NEG	NEG	NEG	NEG	NEG	NEG	NEG	NEG	NEG	NEG	NEG	NEG	POS	POS	POS	POS	POS	NEG	NEG	NEG	POS	POS
**CC15-MSSA**																															
2012_160_Z3444 (26386/11)	NEG	NEG	NEG	NEG	NEG	NEG	NEG	NEG	NEG	NEG	NEG	NEG	NEG	NEG	NEG	NEG	NEG	NEG	NEG	NEG	NEG	NEG	POS	POS	POS	POS	NEG	NEG	NEG	POS	POS
2012_167_Z3514 (27010/11)	NEG	NEG	NEG	NEG	NEG	NEG	NEG	NEG	NEG	NEG	NEG	NEG	NEG	NEG	NEG	NEG	NEG	NEG	NEG	NEG	NEG	NEG	POS	POS	POS	POS	NEG	NEG	NEG	POS	POS
2012_184_Z3455 (376/12)	NEG	NEG	NEG	NEG	NEG	NEG	NEG	NEG	NEG	NEG	NEG	NEG	NEG	NEG	NEG	NEG	NEG	NEG	NEG	NEG	NEG	NEG	POS	POS	POS	POS	NEG	NEG	NEG	POS	POS
2012_205_Z3465 (939/12)	NEG	NEG	NEG	NEG	NEG	NEG	NEG	NEG	NEG	NEG	NEG	NEG	NEG	NEG	NEG	NEG	NEG	NEG	NEG	NEG	NEG	NEG	POS	POS	POS	POS	NEG	NEG	NEG	POS	POS
2012_231_Z3519 (1831/12)	NEG	NEG	NEG	NEG	NEG	NEG	NEG	NEG	NEG	NEG	NEG	NEG	NEG	NEG	NEG	NEG	NEG	NEG	NEG	NEG	NEG	NEG	POS	POS	POS	POS	NEG	NEG	NEG	POS	POS
2012_249_Z3533 (2907/12)	NEG	NEG	NEG	NEG	NEG	NEG	NEG	NEG	NEG	NEG	NEG	NEG	NEG	NEG	NEG	NEG	NEG	NEG	NEG	NEG	NEG	NEG	POS	POS	POS	POS	NEG	NEG	NEG	POS	POS
2012_305_Z3592 (5839/12)	NEG	NEG	NEG	NEG	NEG	NEG	NEG	NEG	NEG	NEG	NEG	NEG	NEG	NEG	NEG	NEG	NEG	NEG	NEG	NEG	NEG	NEG	POS	POS	POS	POS	NEG	NEG	NEG	POS	POS
2012_313_Z3600 (6857/12)	NEG	NEG	NEG	NEG	NEG	NEG	NEG	NEG	NEG	NEG	NEG	NEG	NEG	NEG	NEG	NEG	NEG	NEG	NEG	NEG	NEG	NEG	POS	POS	POS	POS	NEG	NEG	NEG	POS	POS
2012_345_Z3632 (7966B/12)	NEG	NEG	NEG	NEG	NEG	NEG	NEG	NEG	NEG	NEG	NEG	NEG	NEG	NEG	NEG	NEG	NEG	NEG	NEG	NEG	NEG	NEG	POS	POS	POS	POS	NEG	NEG	NEG	POS	POS
2013_PA33_Z4016	NEG	NEG	NEG	NEG	NEG	NEG	NEG	NEG	NEG	NEG	NEG	NEG	NEG	NEG	NEG	NEG	NEG	NEG	NEG	NEG	NEG	NEG	POS	POS	AMB	POS	NEG	NEG	NEG	POS	AMB
**CC20-MSSA**																															
2012_240_Z3526 (2354/12)	NEG	NEG	NEG	NEG	NEG	NEG	NEG	POS	NEG	POS	NEG	NEG	NEG	POS	POS	POS	POS	POS	NEG	NEG	POS	NEG	POS	POS	POS	POS	NEG	NEG	NEG	NEG	POS
**CC30-MSSA**																															
2012_155_Z3488 (25905A/11)	NEG	NEG	NEG	NEG	POS	NEG	NEG	POS	NEG	POS	NEG	NEG	POS	POS	POS	POS	POS	POS	NEG	NEG	POS	NEG	POS	POS	POS	POS	NEG	NEG	NEG	NEG	NEG
**CC30-MSSA [PVL+]**																															
2012_164_Z3447 (26574/11)	NEG	NEG	NEG	NEG	NEG	NEG	NEG	POS	NEG	POS	NEG	NEG	NEG	POS	POS	POS	POS	POS	NEG	NEG	POS	NEG	POS	POS	POS	POS	POS	POS	NEG	NEG	NEG
2012_166_Z3493 (26988A/11)	NEG	NEG	NEG	NEG	NEG	NEG	NEG	POS	NEG	POS	NEG	NEG	NEG	POS	POS	POS	POS	POS	NEG	NEG	POS	NEG	POS	POS	POS	POS	POS	POS	NEG	NEG	NEG
2012_178_Z3500 (197/12)	NEG	NEG	NEG	NEG	NEG	NEG	NEG	POS	NEG	POS	NEG	NEG	NEG	POS	POS	POS	POS	POS	NEG	NEG	POS	NEG	POS	POS	POS	POS	POS	POS	NEG	NEG	NEG
2012_183_Z3454	NEG	NEG	NEG	NEG	NEG	NEG	NEG	POS	NEG	POS	NEG	NEG	NEG	POS	POS	POS	POS	POS	NEG	NEG	POS	NEG	POS	POS	POS	POS	POS	POS	NEG	NEG	NEG
2012_186_Z3503 (444/12)	NEG	NEG	NEG	NEG	NEG	NEG	NEG	POS	NEG	POS	NEG	NEG	NEG	POS	POS	POS	POS	POS	NEG	NEG	POS	NEG	POS	POS	POS	POS	POS	POS	NEG	NEG	NEG
2012_189_Z3506 (542/12)	NEG	NEG	NEG	NEG	NEG	NEG	NEG	POS	NEG	POS	NEG	NEG	NEG	POS	POS	POS	POS	POS	NEG	NEG	POS	NEG	POS	POS	POS	POS	POS	POS	NEG	NEG	NEG
2012_192_Z3457 (606/12)	NEG	NEG	NEG	NEG	NEG	NEG	NEG	POS	NEG	POS	NEG	NEG	NEG	POS	POS	POS	POS	POS	NEG	NEG	POS	NEG	POS	POS	POS	POS	POS	POS	NEG	NEG	NEG
2012_196_Z3509 (707/12)	NEG	NEG	NEG	NEG	NEG	NEG	NEG	POS	NEG	POS	NEG	NEG	NEG	POS	POS	POS	POS	POS	NEG	NEG	POS	NEG	POS	POS	POS	POS	POS	POS	NEG	NEG	NEG
2012_218_Z3476 (1432/12)	NEG	NEG	NEG	NEG	NEG	NEG	NEG	POS	NEG	POS	NEG	NEG	NEG	POS	POS	POS	POS	POS	NEG	NEG	POS	NEG	POS	POS	POS	POS	POS	POS	NEG	NEG	NEG
2012_232_Z3520 (1892/12)	NEG	NEG	NEG	NEG	NEG	NEG	NEG	POS	NEG	POS	NEG	NEG	NEG	POS	POS	POS	POS	POS	NEG	NEG	POS	NEG	POS	POS	POS	POS	POS	POS	NEG	NEG	NEG
2012_278_Z3565 (4397/12)	NEG	NEG	NEG	NEG	NEG	NEG	NEG	POS	NEG	POS	NEG	NEG	NEG	POS	POS	POS	POS	POS	NEG	NEG	POS	NEG	POS	POS	POS	POS	POS	POS	NEG	NEG	NEG
2012_285_Z3572 (4802/12)	NEG	NEG	NEG	NEG	NEG	NEG	NEG	POS	NEG	POS	NEG	NEG	NEG	POS	POS	POS	POS	POS	NEG	NEG	POS	NEG	POS	POS	POS	POS	POS	POS	NEG	NEG	NEG
2012_287_Z3574 (4906/12)	NEG	NEG	NEG	NEG	NEG	NEG	NEG	POS	NEG	POS	NEG	NEG	NEG	POS	POS	POS	POS	POS	NEG	NEG	POS	NEG	POS	POS	POS	POS	POS	POS	NEG	NEG	NEG
2012_295_Z3582 (5116/12)	NEG	NEG	NEG	NEG	NEG	NEG	NEG	POS	NEG	POS	NEG	NEG	NEG	POS	POS	POS	POS	POS	NEG	NEG	POS	NEG	POS	POS	POS	POS	POS	POS	NEG	NEG	NEG
2012_326_Z3613 (7321/12)	NEG	NEG	NEG	NEG	NEG	NEG	NEG	POS	NEG	POS	NEG	NEG	NEG	POS	POS	POS	POS	POS	NEG	NEG	POS	NEG	POS	POS	POS	POS	POS	POS	NEG	NEG	NEG
2012_356_Z3643 (1204-15897)	NEG	NEG	NEG	NEG	NEG	NEG	NEG	POS	NEG	POS	NEG	NEG	NEG	POS	POS	POS	POS	POS	NEG	NEG	POS	NEG	POS	POS	POS	POS	POS	POS	NEG	NEG	NEG
2012_366_Z3426 (1204-16124)	NEG	NEG	NEG	NEG	NEG	NEG	NEG	POS	NEG	POS	NEG	NEG	NEG	POS	POS	POS	POS	POS	NEG	NEG	POS	NEG	POS	POS	POS	POS	POS	POS	NEG	NEG	NEG
2012_372_Z3432 (1205-19821)	NEG	NEG	NEG	NEG	NEG	NEG	NEG	POS	NEG	POS	NEG	NEG	NEG	POS	POS	POS	POS	POS	NEG	NEG	POS	NEG	POS	POS	POS	POS	POS	POS	NEG	NEG	NEG
2012_380_Z3440 (4141)	NEG	NEG	NEG	NEG	NEG	NEG	NEG	POS	NEG	POS	NEG	NEG	NEG	POS	POS	POS	POS	POS	NEG	NEG	POS	NEG	POS	POS	POS	POS	POS	POS	NEG	NEG	NEG
2013_PA20_Z4003	NEG	NEG	NEG	NEG	NEG	NEG	NEG	POS	NEG	POS	NEG	NEG	NEG	POS	POS	POS	POS	POS	NEG	NEG	POS	NEG	POS	POS	POS	POS	POS	POS	NEG	NEG	NEG
2013_PA36_Z4019	NEG	NEG	NEG	NEG	NEG	NEG	NEG	POS	NEG	POS	NEG	NEG	NEG	POS	POS	POS	AMB	POS	NEG	NEG	POS	NEG	POS	POS	NEG	AMB	POS	POS	NEG	NEG	NEG
2013_PA46_Z4029	NEG	NEG	NEG	NEG	NEG	NEG	NEG	POS	NEG	POS	NEG	NEG	NEG	POS	POS	POS	POS	POS	NEG	NEG	POS	NEG	POS	POS	NEG	POS	POS	POS	NEG	NEG	NEG
2013_PA50_Z4033	NEG	NEG	NEG	NEG	NEG	NEG	NEG	POS	NEG	POS	NEG	NEG	NEG	POS	POS	POS	POS	POS	NEG	NEG	POS	NEG	POS	POS	NEG	POS	POS	POS	NEG	NEG	NEG
2013_PA63_Z4046	NEG	NEG	NEG	NEG	NEG	NEG	NEG	POS	NEG	POS	NEG	NEG	NEG	POS	POS	POS	POS	POS	NEG	NEG	POS	NEG	POS	POS	AMB	POS	POS	POS	NEG	NEG	NEG
2013_PA78_Z4061	NEG	NEG	NEG	NEG	NEG	NEG	NEG	POS	NEG	POS	NEG	NEG	NEG	POS	POS	POS	POS	POS	NEG	NEG	POS	NEG	POS	POS	AMB	POS	POS	POS	NEG	NEG	NEG
2013_PA79_Z4062	NEG	NEG	NEG	NEG	NEG	NEG	NEG	POS	NEG	POS	NEG	NEG	NEG	POS	POS	POS	POS	POS	NEG	NEG	POS	NEG	POS	POS	POS	POS	POS	POS	NEG	NEG	NEG
**CC45-MSSA**																															
2012_172_Z3449 (49/12)	NEG	NEG	NEG	AMB	POS	NEG	NEG	POS	NEG	POS	NEG	NEG	POS	POS	POS	POS	POS	POS	NEG	NEG	POS	NEG	POS	POS	POS	POS	NEG	NEG	NEG	NEG	NEG
2012_208_Z3468 (1011/12)	NEG	NEG	NEG	NEG	NEG	NEG	NEG	POS	NEG	POS	NEG	NEG	NEG	POS	POS	POS	POS	POS	NEG	NEG	POS	NEG	POS	POS	POS	POS	NEG	NEG	NEG	NEG	NEG
2012_235_Z3522 (2085/12)	NEG	NEG	NEG	NEG	NEG	NEG	NEG	POS	NEG	POS	NEG	NEG	NEG	POS	POS	POS	POS	POS	NEG	NEG	POS	NEG	POS	POS	POS	POS	NEG	NEG	NEG	NEG	NEG
2012_290_Z3577 (4921/12)	NEG	NEG	NEG	NEG	NEG	NEG	NEG	POS	NEG	POS	NEG	POS	NEG	POS	POS	POS	POS	POS	POS	NEG	POS	NEG	POS	POS	POS	POS	NEG	NEG	NEG	NEG	NEG
2012_311_Z3598 (6182/12)	NEG	NEG	NEG	NEG	POS	NEG	NEG	POS	NEG	POS	NEG	NEG	POS	POS	POS	POS	POS	POS	NEG	NEG	POS	NEG	POS	POS	POS	POS	NEG	NEG	NEG	NEG	NEG
Z3604_TT317 - {DC558C18-2959-4049-B8F3-507365	NEG	NEG	NEG	NEG	NEG	NEG	NEG	POS	NEG	POS	NEG	NEG	NEG	POS	POS	POS	POS	POS	NEG	NEG	POS	NEG	POS	POS	POS	POS	NEG	NEG	NEG	NEG	NEG
2012_363_Z3423 (1112-61058)	NEG	NEG	NEG	NEG	NEG	NEG	NEG	POS	NEG	POS	NEG	NEG	NEG	POS	POS	POS	POS	POS	NEG	NEG	POS	NEG	POS	POS	POS	POS	NEG	NEG	NEG	NEG	NEG
2013_PA19_Z4002	NEG	NEG	NEG	NEG	NEG	NEG	NEG	POS	NEG	POS	NEG	NEG	NEG	POS	POS	POS	POS	POS	NEG	NEG	POS	NEG	POS	POS	POS	POS	NEG	NEG	NEG	NEG	NEG
2013_PA54_Z4037	NEG	NEG	NEG	NEG	NEG	NEG	NEG	POS	NEG	POS	NEG	NEG	NEG	POS	POS	POS	POS	POS	NEG	NEG	POS	NEG	POS	POS	POS	POS	NEG	NEG	NEG	NEG	NEG
2013_PA82_Z4065	NEG	NEG	NEG	NEG	POS	NEG	NEG	POS	NEG	POS	NEG	NEG	POS	POS	POS	POS	POS	POS	NEG	NEG	POS	NEG	POS	AMB	POS	POS	NEG	NEG	NEG	NEG	NEG
**CC97-MSSA**																															
2012_182_Z3453 (279A/12)	NEG	NEG	NEG	NEG	NEG	NEG	NEG	NEG	NEG	NEG	NEG	NEG	NEG	NEG	NEG	NEG	NEG	NEG	NEG	NEG	NEG	NEG	POS	POS	POS	POS	NEG	NEG	NEG	POS	POS
2012_187_Z3504	NEG	NEG	NEG	NEG	NEG	NEG	NEG	NEG	NEG	NEG	NEG	NEG	NEG	NEG	NEG	NEG	NEG	NEG	NEG	NEG	NEG	NEG	POS	POS	POS	POS	NEG	NEG	NEG	POS	POS
2012_197_Z3510 (757/12)	NEG	NEG	NEG	NEG	NEG	NEG	NEG	NEG	NEG	NEG	NEG	NEG	NEG	NEG	NEG	NEG	NEG	NEG	NEG	NEG	NEG	NEG	POS	POS	POS	POS	NEG	NEG	NEG	POS	POS
2012_199_Z3516 (785/12)	NEG	NEG	NEG	NEG	NEG	NEG	NEG	NEG	NEG	NEG	NEG	NEG	NEG	NEG	NEG	NEG	NEG	NEG	NEG	NEG	NEG	NEG	POS	POS	POS	POS	NEG	NEG	NEG	POS	POS
2013_PA15_Z3998	NEG	NEG	NEG	NEG	NEG	NEG	NEG	NEG	NEG	NEG	NEG	NEG	NEG	NEG	NEG	NEG	NEG	NEG	NEG	NEG	NEG	NEG	POS	POS	POS	POS	NEG	NEG	NEG	POS	POS
**CC101-MSSA**																															
2012_283_Z3570 (4725/12)	NEG	NEG	NEG	NEG	NEG	NEG	NEG	NEG	NEG	NEG	NEG	NEG	NEG	NEG	NEG	NEG	NEG	NEG	NEG	NEG	NEG	NEG	POS	POS	POS	POS	NEG	NEG	NEG	POS	POS
2012_247_Z3531 (2788/12)	NEG	NEG	NEG	NEG	NEG	NEG	NEG	NEG	NEG	NEG	NEG	NEG	NEG	NEG	NEG	NEG	NEG	NEG	NEG	NEG	NEG	NEG	POS	POS	POS	POS	NEG	NEG	NEG	POS	POS
2012_279_Z3566 (4506/12)	NEG	NEG	NEG	NEG	NEG	NEG	NEG	NEG	NEG	NEG	NEG	NEG	NEG	NEG	NEG	NEG	NEG	NEG	NEG	NEG	NEG	NEG	POS	POS	POS	POS	NEG	NEG	NEG	POS	POS

Strain / Isolates	toxic shock syndrome toxin 1	enterotoxin A	enterotoxin A, allele from strain N315 = entero-toxin P	enterotoxin B	enterotoxin C	enterotoxin D	enterotoxin E	enterotoxin G	enterotoxin H	enterotoxin I	enterotoxin J	enterotoxin K	enterotoxin L	enterotoxin M	enterotoxin N		enterotoxin O	egc cluster	enterotoxin Q	enterotoxin R	Enterotoxin U and/or Y	enterotoxin-like protein ORF CM14	haemolysin gamma / leukocidin, component B	haemolysin gamma / leukocidin, component C		ysin gamma, comp	Panton Valentine leukocidin F component	Panton Valentine leukocidin S component	hypothetical leukocidin from ruminants	leukocidin D component	leukocidin E component
	tst1	sea	sea (N315) / sep	seb	sec	sed	see	seg	seh	sei	sej	sek	sel	selm	seln (consensus)	seln (other than RF122)	selo	egc (total)	seq	ser	selu	ORF CM14	lukF	lukS	lukS (ST22+ST45)	hlgA	lukF-PV	lukS-PV	lukF-PV (P83)/ lukM	lukD	lukE
**CC121-MSSA**																															
2012_163_Z3446 (26495/11)	NEG	NEG	NEG	POS	NEG	NEG	NEG	POS	NEG	POS	NEG	NEG	NEG	POS	POS	POS	POS	POS	NEG	NEG	POS	POS	POS	POS	POS	POS	NEG	NEG	NEG	POS	POS
2012_204_Z3464 (938/12)	NEG	NEG	NEG	POS	NEG	NEG	NEG	POS	NEG	POS	NEG	NEG	NEG	POS	POS	POS	POS	POS	NEG	NEG	POS	POS	POS	POS	POS	POS	NEG	NEG	NEG	POS	POS
2012_225_Z3482 (1563/12)	NEG	NEG	NEG	POS	NEG	NEG	NEG	POS	NEG	POS	NEG	NEG	NEG	POS	POS	POS	POS	POS	NEG	NEG	POS	POS	POS	POS	POS	POS	NEG	NEG	NEG	POS	POS
2012_228_Z3485 (1700B/12)	NEG	NEG	NEG	POS	NEG	NEG	NEG	POS	NEG	POS	NEG	NEG	NEG	POS	POS	POS	POS	POS	NEG	NEG	POS	POS	POS	POS	POS	POS	NEG	NEG	NEG	POS	POS
2012_229_Z3486 (1737/12)	NEG	NEG	NEG	POS	NEG	NEG	NEG	POS	NEG	POS	NEG	NEG	NEG	POS	POS	POS	POS	POS	NEG	NEG	POS	POS	POS	POS	POS	POS	NEG	NEG	NEG	POS	POS
2012_302_Z3589 (5795/12)	NEG	NEG	NEG	POS	NEG	NEG	NEG	POS	NEG	POS	NEG	NEG	NEG	POS	POS	POS	POS	POS	NEG	NEG	POS	POS	POS	POS	POS	POS	NEG	NEG	NEG	POS	POS
2012_359_Z3419	NEG	NEG	NEG	POS	NEG	NEG	NEG	POS	NEG	POS	NEG	NEG	NEG	POS	POS	POS	POS	POS	NEG	NEG	POS	POS	POS	POS	POS	POS	NEG	NEG	NEG	POS	POS
2012_362_Z3422 (1201-996)	NEG	NEG	NEG	POS	NEG	NEG	NEG	POS	NEG	POS	NEG	NEG	NEG	POS	POS	POS	POS	POS	NEG	NEG	POS	POS	POS	POS	POS	POS	NEG	NEG	NEG	POS	POS
2012_367_Z3427 (1204-16413)	NEG	NEG	NEG	POS	NEG	NEG	NEG	POS	NEG	POS	NEG	NEG	NEG	POS	POS	POS	POS	POS	NEG	NEG	POS	POS	POS	POS	POS	POS	NEG	NEG	NEG	POS	POS
2013_PA09_Z3992	NEG	NEG	NEG	POS	NEG	NEG	NEG	POS	NEG	POS	NEG	NEG	NEG	POS	POS	POS	POS	POS	NEG	NEG	POS	POS	POS	POS	POS	POS	NEG	NEG	NEG	POS	POS
2013_PA59_Z4042	NEG	NEG	NEG	POS	NEG	NEG	NEG	POS	NEG	POS	NEG	NEG	NEG	POS	POS	POS	POS	POS	NEG	NEG	POS	POS	POS	POS	POS	POS	NEG	NEG	NEG	POS	POS
2013_PA60_Z4043	NEG	NEG	NEG	POS	NEG	NEG	NEG	POS	NEG	POS	NEG	NEG	NEG	POS	POS	POS	POS	POS	NEG	NEG	POS	POS	POS	POS	AMB	POS	NEG	NEG	NEG	AMB	POS
**CC152-MSSA [PVL+]**																															
2012_190_Z3507 (549/12)	NEG	NEG	NEG	NEG	NEG	NEG	NEG	NEG	NEG	NEG	NEG	NEG	NEG	NEG	NEG	NEG	NEG	NEG	NEG	NEG	NEG	NEG	POS	NEG	NEG	POS	POS	POS	NEG	POS	NEG
2012_201_Z3461 (823/12)	NEG	NEG	NEG	NEG	NEG	NEG	NEG	NEG	NEG	NEG	NEG	NEG	NEG	NEG	NEG	NEG	NEG	NEG	NEG	NEG	NEG	NEG	POS	NEG	NEG	POS	POS	POS	NEG	POS	NEG
2012_209_Z3469 (1116/12)	NEG	NEG	NEG	NEG	NEG	NEG	NEG	NEG	NEG	NEG	NEG	POS	NEG	NEG	NEG	NEG	NEG	NEG	POS	NEG	NEG	NEG	POS	NEG	NEG	POS	POS	POS	NEG	POS	NEG
2012_211_Z3517 (1238/12)	NEG	NEG	NEG	NEG	NEG	NEG	NEG	NEG	NEG	NEG	NEG	NEG	NEG	NEG	NEG	NEG	NEG	NEG	NEG	NEG	NEG	NEG	POS	NEG	NEG	POS	POS	POS	NEG	POS	NEG
2012_213_Z3472 (1320/12)	NEG	NEG	NEG	NEG	NEG	NEG	NEG	NEG	NEG	NEG	NEG	NEG	NEG	NEG	NEG	NEG	NEG	NEG	NEG	NEG	NEG	NEG	POS	NEG	NEG	POS	POS	POS	NEG	AMB	NEG
2012_221_Z3478	NEG	NEG	NEG	NEG	NEG	NEG	NEG	NEG	NEG	NEG	NEG	POS	NEG	NEG	NEG	NEG	NEG	NEG	POS	NEG	NEG	NEG	POS	NEG	NEG	POS	POS	POS	NEG	POS	NEG
2012_234_Z3521 (1981/12)	NEG	NEG	NEG	NEG	NEG	NEG	NEG	NEG	NEG	NEG	NEG	NEG	NEG	NEG	NEG	NEG	NEG	NEG	NEG	NEG	NEG	NEG	POS	NEG	NEG	POS	POS	POS	NEG	POS	NEG
2012_238_Z3524 (2199/12)	NEG	NEG	NEG	NEG	NEG	NEG	NEG	NEG	NEG	NEG	NEG	NEG	NEG	NEG	NEG	NEG	NEG	NEG	NEG	NEG	NEG	NEG	POS	NEG	NEG	POS	POS	POS	NEG	POS	NEG
2012_260_Z3547 (3290/12)	NEG	NEG	NEG	NEG	NEG	NEG	NEG	NEG	NEG	NEG	NEG	NEG	NEG	NEG	NEG	NEG	NEG	NEG	NEG	NEG	NEG	NEG	POS	NEG	NEG	POS	POS	POS	NEG	POS	NEG
2012_272_Z3559 (3802/12)	NEG	NEG	NEG	NEG	NEG	NEG	NEG	NEG	NEG	NEG	NEG	NEG	NEG	NEG	NEG	NEG	NEG	NEG	NEG	NEG	NEG	NEG	POS	NEG	NEG	POS	POS	POS	NEG	POS	NEG
2012_289_Z3576 (4912/12)	NEG	NEG	NEG	NEG	NEG	NEG	NEG	NEG	NEG	NEG	NEG	NEG	NEG	NEG	NEG	NEG	NEG	NEG	NEG	NEG	NEG	NEG	POS	NEG	NEG	POS	POS	POS	NEG	POS	NEG
2012_309_Z3596	NEG	NEG	NEG	NEG	NEG	NEG	NEG	NEG	NEG	NEG	NEG	POS	NEG	NEG	NEG	NEG	NEG	NEG	POS	NEG	NEG	NEG	POS	NEG	NEG	POS	POS	POS	NEG	POS	NEG
2012_322_Z3609 (7178/12)	NEG	NEG	NEG	NEG	NEG	NEG	NEG	NEG	NEG	NEG	NEG	NEG	NEG	NEG	NEG	NEG	NEG	NEG	NEG	NEG	NEG	NEG	AMB	NEG	NEG	POS	POS	POS	NEG	AMB	NEG
2012_325_Z3612 (7254/12)	NEG	NEG	NEG	NEG	NEG	NEG	NEG	NEG	NEG	NEG	NEG	NEG	NEG	NEG	NEG	NEG	NEG	NEG	NEG	NEG	NEG	NEG	POS	NEG	NEG	POS	POS	POS	NEG	POS	NEG
2012_328_Z3615 (7456/12)	NEG	NEG	NEG	NEG	NEG	NEG	NEG	NEG	NEG	NEG	NEG	NEG	NEG	NEG	NEG	NEG	NEG	NEG	NEG	NEG	NEG	NEG	POS	NEG	NEG	POS	POS	POS	NEG	POS	NEG
2012_334_Z3621 (8267/12)	NEG	NEG	NEG	NEG	NEG	NEG	NEG	NEG	NEG	NEG	NEG	NEG	NEG	NEG	NEG	NEG	NEG	NEG	NEG	NEG	NEG	NEG	POS	NEG	NEG	POS	POS	POS	NEG	POS	NEG
2012_360_Z3420 (1201-4047)	NEG	NEG	NEG	NEG	NEG	NEG	NEG	NEG	NEG	NEG	NEG	NEG	NEG	NEG	NEG	NEG	NEG	NEG	NEG	NEG	NEG	NEG	POS	NEG	NEG	POS	POS	POS	NEG	POS	NEG
2012_382_Z3442 (4086)	NEG	NEG	NEG	NEG	NEG	NEG	NEG	NEG	NEG	NEG	NEG	NEG	NEG	NEG	NEG	NEG	NEG	NEG	NEG	NEG	NEG	NEG	POS	NEG	NEG	POS	POS	POS	NEG	POS	NEG
2013_PA02_Z3985	NEG	NEG	NEG	NEG	NEG	NEG	NEG	NEG	NEG	NEG	NEG	NEG	NEG	NEG	NEG	NEG	NEG	NEG	NEG	NEG	NEG	NEG	POS	NEG	NEG	POS	POS	POS	NEG	POS	NEG
2013_PA03_Z3986	NEG	NEG	NEG	NEG	NEG	NEG	NEG	NEG	NEG	NEG	NEG	NEG	NEG	NEG	NEG	NEG	NEG	NEG	NEG	NEG	NEG	NEG	POS	NEG	NEG	POS	POS	POS	NEG	AMB	NEG
2013_PA07_Z3990	NEG	NEG	NEG	NEG	NEG	NEG	NEG	NEG	NEG	NEG	NEG	NEG	NEG	NEG	NEG	NEG	NEG	NEG	NEG	NEG	NEG	NEG	POS	NEG	NEG	POS	POS	POS	NEG	POS	NEG
2013_PA34_Z4017	NEG	NEG	NEG	NEG	NEG	NEG	NEG	NEG	NEG	NEG	NEG	NEG	NEG	NEG	NEG	NEG	NEG	NEG	NEG	NEG	NEG	NEG	AMB	NEG	NEG	POS	POS	POS	NEG	NEG	NEG
2013_PA40_Z4023	NEG	NEG	NEG	NEG	NEG	NEG	NEG	NEG	NEG	NEG	NEG	NEG	NEG	NEG	NEG	NEG	NEG	NEG	NEG	NEG	NEG	NEG	POS	NEG	NEG	POS	POS	POS	NEG	NEG	NEG
2013_PA48_Z4031	NEG	NEG	NEG	NEG	NEG	NEG	NEG	NEG	NEG	NEG	NEG	NEG	NEG	NEG	NEG	NEG	NEG	NEG	NEG	NEG	NEG	NEG	POS	NEG	NEG	POS	POS	POS	NEG	AMB	NEG
2013_PA49_Z4032	NEG	NEG	NEG	NEG	NEG	NEG	NEG	NEG	NEG	NEG	NEG	NEG	NEG	NEG	NEG	NEG	NEG	NEG	NEG	NEG	NEG	NEG	AMB	NEG	NEG	POS	POS	POS	NEG	NEG	NEG
2013_PA51_Z4034	NEG	NEG	NEG	NEG	NEG	NEG	NEG	NEG	NEG	NEG	NEG	NEG	NEG	NEG	NEG	NEG	NEG	NEG	NEG	NEG	NEG	NEG	POS	NEG	NEG	POS	POS	POS	NEG	POS	NEG
2013_PA70_Z4053	NEG	NEG	NEG	NEG	NEG	NEG	NEG	NEG	NEG	NEG	NEG	NEG	NEG	NEG	NEG	NEG	NEG	NEG	NEG	NEG	NEG	NEG	POS	NEG	NEG	POS	POS	POS	NEG	NEG	NEG
2013_PA84_Z4067	NEG	NEG	NEG	NEG	NEG	NEG	NEG	NEG	NEG	NEG	NEG	NEG	NEG	NEG	NEG	NEG	NEG	NEG	NEG	NEG	NEG	NEG	POS	NEG	NEG	POS	POS	POS	NEG	AMB	NEG
**CC188-MSSA**																															
2012_206_Z3466 (968/12)	NEG	NEG	NEG	NEG	NEG	NEG	NEG	NEG	NEG	NEG	NEG	NEG	NEG	NEG	NEG	NEG	NEG	NEG	NEG	NEG	NEG	NEG	POS	POS	POS	POS	NEG	NEG	NEG	POS	POS
2012_251_Z3534 (3026/12)	NEG	NEG	NEG	POS	NEG	NEG	NEG	NEG	NEG	NEG	NEG	NEG	NEG	NEG	NEG	NEG	NEG	NEG	NEG	NEG	NEG	NEG	POS	POS	POS	POS	NEG	NEG	NEG	POS	POS
2012_320_Z3607 (7144/12)	NEG	NEG	NEG	POS	NEG	NEG	NEG	NEG	NEG	NEG	NEG	NEG	NEG	NEG	NEG	NEG	NEG	NEG	NEG	NEG	NEG	NEG	POS	POS	POS	POS	NEG	NEG	NEG	POS	POS
2012_321_Z3608 (7147/12)	NEG	NEG	NEG	NEG	NEG	NEG	NEG	NEG	NEG	NEG	NEG	NEG	NEG	NEG	NEG	NEG	NEG	NEG	NEG	NEG	NEG	NEG	POS	POS	POS	POS	NEG	NEG	NEG	POS	POS
2012_358_Z3418 (1202-6332)	NEG	NEG	NEG	POS	NEG	NEG	NEG	NEG	NEG	NEG	NEG	NEG	NEG	NEG	NEG	NEG	NEG	NEG	NEG	NEG	NEG	NEG	POS	POS	POS	POS	NEG	NEG	NEG	POS	POS
**CC398-MSSA**																															
2013_PA11_Z3994	NEG	NEG	NEG	NEG	NEG	NEG	NEG	NEG	NEG	NEG	NEG	NEG	NEG	NEG	NEG	NEG	NEG	NEG	NEG	NEG	NEG	NEG	POS	POS	POS	POS	NEG	NEG	NEG	NEG	NEG
2013_PA57_Z4040	NEG	NEG	NEG	NEG	NEG	NEG	NEG	NEG	NEG	NEG	NEG	NEG	NEG	NEG	NEG	NEG	NEG	NEG	NEG	NEG	NEG	NEG	POS	POS	POS	POS	NEG	NEG	NEG	NEG	NEG
**ST1852-MSSA**																															
2012_277_Z3564 (4050/12)	NEG	NEG	NEG	NEG	NEG	NEG	NEG	NEG	NEG	NEG	NEG	NEG	NEG	NEG	NEG	NEG	NEG	NEG	NEG	NEG	NEG	NEG	POS	POS	POS	POS	NEG	NEG	NEG	NEG	POS
**CC1223/1594-MSSA**																															
2012_337_Z3624 (8070B/12)	NEG	NEG	NEG	POS	NEG	NEG	NEG	POS	NEG	POS	NEG	NEG	NEG	POS	NEG	POS	POS	POS	NEG	NEG	POS	NEG	NEG	NEG	NEG	NEG	NEG	NEG	NEG	NEG	NEG
**MSSA related to ST2250/2277**																															
2012_203_Z3463 (829/12)	NEG	NEG	NEG	NEG	NEG	POS	NEG	NEG	NEG	NEG	POS	AMB	NEG	NEG	NEG	NEG	NEG	NEG	NEG	NEG	NEG	NEG	NEG	NEG	NEG	NEG	NEG	NEG	NEG	POS	POS
2012_269_Z3556 (3769A/12)	NEG	NEG	NEG	NEG	NEG	NEG	NEG	NEG	NEG	NEG	NEG	NEG	NEG	NEG	NEG	NEG	NEG	NEG	NEG	NEG	NEG	NEG	NEG	NEG	NEG	NEG	NEG	NEG	NEG	POS	NEG
2012_292_Z3579	NEG	NEG	NEG	NEG	NEG	NEG	NEG	NEG	NEG	NEG	NEG	NEG	NEG	NEG	NEG	NEG	NEG	NEG	NEG	NEG	NEG	NEG	NEG	NEG	NEG	NEG	NEG	NEG	NEG	AMB	NEG
2012_299_Z3586 (5487/12)	NEG	NEG	NEG	NEG	NEG	NEG	NEG	NEG	NEG	NEG	NEG	NEG	NEG	NEG	NEG	NEG	NEG	NEG	NEG	NEG	NEG	NEG	NEG	NEG	NEG	NEG	NEG	NEG	NEG	AMB	NEG
2012_301_Z3588 (5717/12)	NEG	NEG	NEG	NEG	NEG	NEG	NEG	NEG	NEG	NEG	NEG	NEG	NEG	NEG	NEG	NEG	NEG	NEG	NEG	NEG	NEG	NEG	NEG	NEG	NEG	NEG	NEG	NEG	NEG	AMB	NEG
**ST2596dlv-MSSA**																															
2012_310_Z3597 (6034/12)	NEG	NEG	NEG	NEG	NEG	NEG	NEG	NEG	NEG	NEG	NEG	NEG	NEG	NEG	NEG	NEG	NEG	NEG	NEG	NEG	NEG	NEG	NEG	NEG	NEG	NEG	NEG	NEG	NEG	AMB	NEG

Strain / Isolates	leukocidin/ haemolysin toxin family protein	leukocidin/haemolysin toxin family protein		putative membrane protein	haemolysin alpha	putative membrane protein		haemolysin beta				staphylo-kinase	chemotaxis-inhibiting protein (CHIPS)	Staphyl. Comple-ment inhibitor	exfoliative toxin serotype A	exfoliative toxin serotype B	exfoliative toxin D	epidermal cell differen-tiation inhibitor	epidermal cell differen-tiation inhibitor B	epidermal cell differen-tiation inhibitor C	ACME-locus	ACME-locus	ACME-locus: ornithincarb-amoyltrans-ferase	ACME-locus: carbamat-kinase	ACME-locus: arginine/orni-thine-antiporter	aureolysin			serin- protease A	serin- protease B	serin- protease E
	lukX	lukY	lukY (ST30+ST45)	hl	hla	hlIII (consensus)	hlIII (other than RF122)	hlb_probe 1	hlb_probe 2	hlb_probe 3	un-disrupted hlb	sak	chp	scn	etA	etB	etD	edinA	edinB	edinC	ACME (total)	arcA-SCC	arcB-SCC	arcC-SCC	arcD-SCC	aur (consensus)	aur (other than MRSA252)	aur (MRSA252)	splA	splB	splE
**CC1-MSSA**																															
2012_257_Z3544 (3216/12)	POS	POS	NEG	POS	POS	POS	POS	POS	POS	POS	POS	NEG	NEG	NEG	NEG	NEG	NEG	NEG	NEG	NEG	NEG	NEG	NEG	NEG	NEG	POS	POS	NEG	POS	POS	POS
2012_281_Z3568 (4696/12)	POS	POS	NEG	POS	POS	POS	POS	POS	POS	POS	POS	NEG	NEG	NEG	NEG	NEG	NEG	NEG	NEG	NEG	NEG	NEG	NEG	NEG	NEG	POS	POS	NEG	POS	POS	POS
2012_324_Z3611 (7180/12)	POS	POS	NEG	POS	POS	POS	POS	POS	POS	POS	POS	POS	NEG	POS	NEG	NEG	NEG	NEG	NEG	NEG	NEG	NEG	NEG	NEG	NEG	POS	POS	NEG	POS	POS	NEG
2012_327_Z3614 (7347/12)	POS	POS	NEG	POS	POS	POS	POS	POS	POS	POS	POS	POS	NEG	POS	NEG	NEG	NEG	NEG	NEG	NEG	NEG	NEG	NEG	NEG	NEG	POS	POS	NEG	POS	POS	POS
2012_351_Z3638 (8273B/12)	POS	POS	NEG	POS	POS	POS	POS	POS	POS	POS	POS	POS	NEG	POS	NEG	NEG	NEG	NEG	NEG	NEG	NEG	NEG	NEG	NEG	NEG	POS	POS	NEG	POS	POS	POS
**CC5-MSSA**																															
2012_188_Z3505 (503/12)	POS	POS	NEG	POS	POS	POS	POS	POS	POS	POS	POS	NEG	NEG	NEG	NEG	NEG	NEG	NEG	NEG	NEG	NEG	NEG	NEG	NEG	NEG	POS	POS	NEG	POS	POS	NEG
2012_219_Z3518 (1437/12)	POS	POS	NEG	POS	POS	POS	POS	POS	POS	POS	AMB	POS	POS	POS	NEG	NEG	NEG	NEG	NEG	NEG	NEG	NEG	NEG	NEG	NEG	POS	POS	NEG	POS	NEG	NEG
2012_329_Z3616 (7577/12)	POS	POS	NEG	POS	POS	POS	POS	POS	POS	POS	POS	POS	NEG	POS	NEG	NEG	NEG	NEG	NEG	NEG	NEG	NEG	NEG	NEG	NEG	POS	POS	NEG	POS	POS	NEG
2012_377_Z3437 (6242)	POS	POS	NEG	POS	POS	POS	POS	POS	POS	POS	POS	POS	NEG	POS	NEG	NEG	NEG	NEG	NEG	NEG	NEG	NEG	NEG	NEG	NEG	POS	POS	NEG	POS	POS	NEG
2013_PA04_Z3987	POS	POS	NEG	POS	POS	POS	POS	POS	POS	POS	NEG	NEG	NEG	NEG	NEG	NEG	NEG	NEG	NEG	NEG	NEG	NEG	NEG	NEG	NEG	POS	POS	NEG	POS	POS	NEG
2013_PA32_Z4015	POS	POS	NEG	POS	POS	POS	POS	POS	POS	POS	AMB	POS	NEG	POS	NEG	NEG	NEG	NEG	NEG	NEG	NEG	NEG	NEG	NEG	NEG	POS	POS	NEG	POS	POS	NEG
2013_PA41_Z4024	POS	POS	NEG	POS	POS	POS	POS	POS	POS	POS	NEG	POS	POS	POS	NEG	NEG	NEG	NEG	NEG	NEG	NEG	NEG	NEG	NEG	NEG	POS	POS	NEG	POS	POS	NEG
2013_PA44_Z4027	POS	POS	NEG	POS	POS	POS	POS	POS	POS	POS	NEG	POS	POS	POS	NEG	NEG	NEG	NEG	NEG	NEG	NEG	NEG	NEG	NEG	NEG	POS	POS	NEG	POS	POS	NEG
**CC5-MSSA [PVL+]**																															
2012_175_Z3497 (112/12)	POS	POS	NEG	POS	POS	POS	POS	POS	POS	POS	AMB	POS	POS	POS	NEG	NEG	NEG	POS	NEG	NEG	NEG	NEG	NEG	NEG	NEG	POS	POS	NEG	POS	POS	NEG
2012_193_Z3458 (617/12)	POS	POS	NEG	POS	POS	POS	POS	POS	POS	POS	POS	POS	POS	POS	NEG	NEG	NEG	POS	NEG	NEG	NEG	NEG	NEG	NEG	NEG	POS	POS	NEG	POS	POS	NEG
2012_215_Z3474 (1341/12)	POS	POS	NEG	POS	POS	POS	POS	POS	POS	POS	NEG	POS	POS	POS	NEG	NEG	NEG	POS	NEG	NEG	NEG	NEG	NEG	NEG	NEG	POS	POS	NEG	POS	POS	NEG
2012_216_Z3475 (1370/12)	POS	POS	NEG	POS	POS	POS	POS	POS	POS	AMB	POS	POS	POS	POS	NEG	NEG	NEG	POS	NEG	NEG	NEG	NEG	NEG	NEG	NEG	POS	POS	NEG	POS	POS	NEG
2012_297_Z3584 (5140/12)	POS	POS	NEG	POS	POS	POS	POS	POS	POS	NEG	AMB	POS	POS	POS	NEG	NEG	NEG	POS	NEG	NEG	NEG	NEG	NEG	NEG	NEG	POS	POS	NEG	POS	POS	NEG
2012_331_Z3618 (7644/12)	POS	POS	NEG	POS	POS	POS	POS	POS	POS	POS	POS	POS	POS	POS	NEG	NEG	NEG	POS	NEG	NEG	NEG	NEG	NEG	NEG	NEG	POS	POS	NEG	POS	POS	NEG
2012_379_Z3439 (4126)	POS	POS	NEG	POS	POS	POS	POS	POS	POS	AMB	AMB	POS	POS	POS	NEG	NEG	NEG	POS	NEG	NEG	NEG	NEG	NEG	NEG	NEG	POS	POS	NEG	POS	POS	NEG
2012_381_Z3441	POS	POS	NEG	POS	POS	POS	POS	POS	POS	POS	POS	POS	POS	POS	NEG	NEG	NEG	POS	NEG	NEG	NEG	NEG	NEG	NEG	NEG	POS	POS	NEG	POS	POS	NEG
2013_PA39_Z4022	POS	POS	NEG	POS	POS	POS	POS	POS	POS	NEG	AMB	POS	POS	POS	NEG	NEG	NEG	POS	NEG	NEG	NEG	NEG	NEG	NEG	NEG	POS	POS	NEG	POS	POS	NEG
2013_PA47_Z4030	POS	POS	NEG	POS	POS	POS	POS	AMB	POS	NEG	NEG	POS	POS	POS	NEG	NEG	NEG	POS	NEG	NEG	NEG	NEG	NEG	NEG	NEG	POS	POS	NEG	POS	POS	NEG
2013_PA61_I_Z4044	POS	POS	NEG	POS	POS	POS	POS	POS	POS	AMB	AMB	POS	POS	POS	NEG	NEG	NEG	POS	NEG	NEG	NEG	NEG	NEG	NEG	NEG	POS	POS	NEG	POS	POS	NEG
2013_PA62_I_Z4045	POS	POS	NEG	POS	POS	POS	POS	POS	POS	AMB	AMB	POS	POS	POS	NEG	NEG	NEG	POS	NEG	NEG	NEG	NEG	NEG	NEG	NEG	POS	POS	NEG	POS	POS	NEG
2013_PA74_Z4057	POS	POS	NEG	POS	POS	POS	POS	AMB	POS	NEG	NEG	POS	POS	POS	NEG	NEG	NEG	NEG	NEG	NEG	NEG	NEG	NEG	NEG	NEG	POS	POS	NEG	POS	POS	NEG
2013_PA75_Z4058	POS	POS	NEG	POS	POS	POS	POS	NEG	POS	NEG	AMB	POS	POS	POS	NEG	NEG	NEG	POS	NEG	NEG	NEG	NEG	NEG	NEG	NEG	POS	POS	NEG	POS	POS	NEG
**CC5-MRSA-V [ACME+]**																															
2012_373_Z3433 (1204-17312)	POS	POS	NEG	POS	POS	POS	POS	POS	POS	POS	POS	NEG	NEG	NEG	NEG	NEG	NEG	NEG	NEG	NEG	POS	POS	POS	POS	POS	POS	POS	NEG	POS	POS	NEG
**CC6-MSSA**																															
2012_173_Z3450 (98A/12)	POS	POS	NEG	POS	POS	POS	POS	POS	POS	POS	POS	POS	NEG	POS	POS	NEG	NEG	NEG	NEG	NEG	NEG	NEG	NEG	NEG	NEG	POS	POS	NEG	POS	POS	POS
2012_176_Z3498	POS	POS	NEG	POS	POS	POS	POS	POS	POS	POS	AMB	POS	NEG	POS	POS	NEG	NEG	NEG	NEG	NEG	NEG	NEG	NEG	NEG	NEG	POS	POS	NEG	POS	POS	POS
2012_181_Z3452	POS	POS	NEG	POS	POS	POS	POS	POS	POS	POS	AMB	POS	NEG	POS	POS	NEG	NEG	NEG	NEG	NEG	NEG	NEG	NEG	NEG	NEG	POS	POS	NEG	POS	POS	POS
2012_259_Z3546 (3279/12)	POS	POS	NEG	POS	POS	POS	POS	POS	POS	POS	POS	POS	NEG	POS	NEG	NEG	NEG	NEG	NEG	NEG	NEG	NEG	NEG	NEG	NEG	POS	POS	NEG	POS	POS	POS
2012_264_Z3551 (3407B/12)	POS	POS	NEG	POS	POS	POS	POS	POS	POS	POS	POS	POS	NEG	POS	NEG	NEG	NEG	NEG	NEG	NEG	NEG	NEG	NEG	NEG	NEG	POS	POS	NEG	POS	POS	POS
2012_273_Z3560 (3844/12)	POS	POS	NEG	POS	POS	POS	POS	POS	POS	POS	POS	POS	NEG	POS	NEG	NEG	NEG	NEG	NEG	NEG	NEG	NEG	NEG	NEG	NEG	POS	POS	NEG	POS	POS	POS
2012_312_Z3599 (6815/12)	POS	POS	NEG	POS	POS	POS	POS	POS	POS	POS	AMB	POS	NEG	POS	POS	NEG	NEG	NEG	NEG	NEG	NEG	NEG	NEG	NEG	NEG	POS	POS	NEG	POS	POS	POS
2012_338_Z3625	POS	POS	NEG	POS	POS	POS	POS	POS	POS	POS	POS	POS	NEG	POS	NEG	NEG	NEG	NEG	NEG	NEG	NEG	NEG	NEG	NEG	NEG	POS	POS	NEG	POS	POS	POS
2012_352_Z3639	POS	POS	NEG	POS	POS	POS	POS	POS	POS	POS	POS	POS	NEG	POS	NEG	NEG	NEG	NEG	NEG	NEG	NEG	NEG	NEG	NEG	NEG	POS	POS	NEG	POS	POS	POS
2012_368_Z3428	POS	POS	NEG	POS	POS	POS	POS	POS	POS	POS	AMB	POS	NEG	POS	NEG	NEG	NEG	NEG	NEG	NEG	NEG	NEG	NEG	NEG	NEG	POS	POS	NEG	POS	POS	POS
2013_PA12_Z3995	POS	POS	NEG	POS	POS	POS	POS	POS	POS	POS	NEG	POS	NEG	POS	NEG	NEG	NEG	NEG	NEG	NEG	NEG	NEG	NEG	NEG	NEG	POS	POS	NEG	POS	POS	NEG
2013_PA28_Z4011	POS	POS	NEG	POS	POS	POS	POS	POS	POS	POS	NEG	POS	NEG	POS	POS	NEG	NEG	NEG	NEG	NEG	NEG	NEG	NEG	NEG	NEG	POS	POS	NEG	POS	POS	POS
**CC6-MSSA [PVL+]**																															
2012_375_Z3435	POS	POS	NEG	POS	POS	POS	POS	POS	POS	POS	AMB	POS	NEG	POS	NEG	NEG	NEG	NEG	NEG	NEG	NEG	NEG	NEG	NEG	NEG	POS	POS	NEG	POS	POS	POS
2013_PA77_Z4060	POS	POS	NEG	POS	POS	POS	POS	POS	POS	POS	AMB	POS	NEG	POS	NEG	NEG	NEG	NEG	NEG	NEG	NEG	NEG	NEG	NEG	NEG	POS	POS	NEG	POS	POS	POS
**CC7-MSSA**																															
2012_248_Z3532 (2864/12)	POS	POS	NEG	POS	POS	POS	POS	POS	POS	POS	AMB	POS	NEG	POS	NEG	NEG	NEG	NEG	NEG	NEG	NEG	NEG	NEG	NEG	NEG	POS	POS	NEG	POS	POS	POS
2012_265_Z3552 (3518/12)	POS	POS	NEG	POS	POS	POS	POS	POS	POS	POS	AMB	POS	NEG	POS	NEG	NEG	NEG	NEG	NEG	NEG	NEG	NEG	NEG	NEG	NEG	POS	POS	NEG	POS	POS	POS
2013_PA53_Z4036	POS	POS	NEG	POS	POS	POS	POS	POS	POS	POS	POS	POS	NEG	POS	NEG	NEG	NEG	NEG	NEG	NEG	NEG	NEG	NEG	NEG	NEG	POS	POS	NEG	POS	POS	POS
2013_PA83_Z4066	POS	POS	NEG	POS	POS	POS	POS	POS	POS	POS	AMB	POS	NEG	POS	NEG	NEG	NEG	NEG	NEG	NEG	NEG	NEG	NEG	NEG	NEG	POS	POS	NEG	POS	POS	POS
**CC8-MSSA**																															
2012_243_Z3528 (2587/12)	POS	POS	NEG	POS	POS	POS	POS	POS	POS	POS	NEG	POS	POS	POS	NEG	NEG	NEG	NEG	NEG	NEG	NEG	NEG	NEG	NEG	NEG	POS	POS	NEG	NEG	NEG	NEG
2012_344_Z3631 (8605/12)	POS	POS	NEG	POS	POS	POS	POS	POS	POS	POS	POS	POS	POS	POS	NEG	NEG	NEG	NEG	NEG	NEG	NEG	NEG	NEG	NEG	NEG	POS	POS	NEG	POS	POS	POS
2012_346_Z3633 (8074/12)	POS	POS	NEG	POS	POS	POS	POS	POS	POS	POS	AMB	POS	NEG	POS	NEG	NEG	NEG	NEG	NEG	NEG	NEG	NEG	NEG	NEG	NEG	POS	POS	NEG	NEG	POS	POS
2012_255_Z3538 (3205B/12)	POS	POS	NEG	POS	POS	POS	POS	POS	POS	POS	POS	NEG	NEG	NEG	NEG	NEG	NEG	NEG	NEG	NEG	NEG	NEG	NEG	NEG	NEG	POS	POS	NEG	NEG	POS	POS
2012_280_Z3567 (4601/12)	POS	POS	NEG	POS	POS	POS	POS	POS	POS	POS	POS	NEG	NEG	NEG	NEG	NEG	NEG	NEG	NEG	NEG	NEG	NEG	NEG	NEG	NEG	POS	POS	NEG	POS	POS	POS
Trinidad&Tobago_2012_267 (3642/12)	POS	POS	NEG	POS	POS	POS	POS	POS	POS	POS	POS	NEG	NEG	NEG	NEG	NEG	NEG	NEG	NEG	NEG	NEG	NEG	NEG	NEG	NEG	POS	POS	NEG	NEG	POS	POS
2012_365_Z3425 (1204-15838)	POS	POS	NEG	POS	POS	POS	POS	POS	POS	POS	AMB	POS	POS	POS	NEG	NEG	NEG	NEG	NEG	NEG	NEG	NEG	NEG	NEG	NEG	POS	POS	NEG	POS	POS	POS
2012_296_Z3583 (5118/12)	POS	POS	NEG	POS	POS	POS	POS	POS	POS	POS	POS	NEG	NEG	NEG	NEG	NEG	NEG	NEG	NEG	NEG	NEG	NEG	NEG	NEG	NEG	POS	POS	NEG	POS	POS	POS
2012_318_Z3605 (6960/12)	POS	POS	NEG	POS	POS	POS	POS	POS	POS	POS	AMB	POS	POS	POS	NEG	NEG	NEG	NEG	NEG	NEG	NEG	NEG	NEG	NEG	NEG	POS	POS	NEG	POS	POS	POS
2012_306_Z3593 (5861/12)	POS	POS	NEG	POS	POS	POS	POS	POS	POS	POS	POS	NEG	NEG	NEG	NEG	NEG	NEG	NEG	NEG	NEG	NEG	NEG	NEG	NEG	NEG	POS	POS	NEG	POS	POS	NEG
2013_PA37_Z4020	POS	POS	NEG	POS	POS	POS	POS	POS	POS	POS	AMB	POS	POS	POS	NEG	NEG	NEG	NEG	NEG	NEG	NEG	NEG	NEG	NEG	NEG	POS	POS	NEG	NEG	POS	POS
2013_PA38_Z4021	POS	AMB	NEG	POS	AMB	POS	POS	POS	POS	AMB	NEG	POS	POS	POS	NEG	NEG	NEG	NEG	NEG	NEG	NEG	NEG	NEG	NEG	NEG	POS	POS	NEG	POS	POS	NEG
2013_PA52_Z4035	POS	POS	NEG	POS	POS	POS	POS	POS	POS	POS	NEG	POS	POS	POS	NEG	NEG	NEG	NEG	NEG	NEG	NEG	NEG	NEG	NEG	NEG	POS	POS	NEG	POS	POS	POS
**CC8-MSSA [PVL+]**																															
2012_156_Z3489 (25917/11)	POS	POS	NEG	POS	POS	POS	POS	POS	POS	POS	POS	POS	POS	POS	NEG	NEG	NEG	NEG	NEG	NEG	NEG	NEG	NEG	NEG	NEG	POS	POS	NEG	NEG	NEG	NEG
2012_159_Z3492 (26310/11)	POS	POS	NEG	POS	POS	POS	POS	POS	POS	POS	AMB	POS	POS	POS	NEG	NEG	NEG	NEG	NEG	NEG	NEG	NEG	NEG	NEG	NEG	POS	POS	NEG	NEG	NEG	NEG
2012_161_Z3445 (26430/11)	POS	POS	NEG	POS	POS	POS	POS	POS	POS	POS	POS	POS	POS	POS	NEG	NEG	NEG	NEG	NEG	NEG	NEG	NEG	NEG	NEG	NEG	POS	POS	NEG	NEG	NEG	NEG
2012_162_Z3512 (26433/11)	POS	POS	NEG	POS	POS	POS	POS	POS	POS	POS	AMB	POS	POS	POS	NEG	NEG	NEG	NEG	NEG	NEG	NEG	NEG	NEG	NEG	NEG	POS	POS	NEG	NEG	NEG	NEG
2012_169_Z3495 (27194/11)	POS	POS	NEG	POS	POS	POS	POS	POS	POS	POS	AMB	POS	POS	POS	NEG	NEG	NEG	NEG	NEG	NEG	NEG	NEG	NEG	NEG	NEG	POS	POS	NEG	NEG	NEG	NEG
2012_170_Z3496 (35/12)	POS	POS	NEG	POS	POS	POS	POS	POS	POS	POS	AMB	POS	POS	POS	NEG	NEG	NEG	NEG	NEG	NEG	NEG	NEG	NEG	NEG	NEG	POS	POS	NEG	NEG	NEG	NEG
2012_171_Z3448 (47/12)	POS	POS	NEG	POS	POS	POS	POS	POS	POS	POS	POS	POS	POS	POS	NEG	NEG	NEG	NEG	NEG	NEG	NEG	NEG	NEG	NEG	NEG	POS	POS	NEG	NEG	NEG	NEG
2012_177_Z3499 (184/12)	POS	POS	NEG	POS	POS	POS	POS	POS	POS	POS	AMB	POS	POS	POS	NEG	NEG	NEG	NEG	NEG	NEG	NEG	NEG	NEG	NEG	NEG	POS	POS	NEG	NEG	NEG	NEG
2012_179_Z3515 (231/12)	POS	POS	NEG	POS	POS	POS	POS	POS	POS	POS	AMB	POS	POS	POS	NEG	NEG	NEG	NEG	NEG	NEG	NEG	NEG	NEG	NEG	NEG	POS	POS	NEG	NEG	NEG	NEG
2012_185_Z3502 (437/12)	POS	POS	NEG	POS	POS	POS	POS	POS	POS	POS	NEG	POS	POS	POS	NEG	NEG	NEG	NEG	NEG	NEG	NEG	NEG	NEG	NEG	NEG	POS	POS	NEG	NEG	NEG	NEG
2012_191_Z3456 (563/12)	POS	POS	NEG	POS	POS	POS	POS	POS	POS	POS	AMB	POS	POS	POS	NEG	NEG	NEG	NEG	NEG	NEG	NEG	NEG	NEG	NEG	NEG	POS	POS	NEG	NEG	NEG	NEG
2012_194_Z3459	POS	POS	NEG	POS	POS	POS	POS	POS	POS	POS	AMB	POS	POS	POS	NEG	NEG	NEG	NEG	NEG	NEG	NEG	NEG	NEG	NEG	NEG	POS	POS	NEG	NEG	NEG	NEG
2012_200_Z3460 (820/12)	POS	POS	NEG	POS	POS	POS	POS	POS	POS	POS	POS	POS	POS	POS	NEG	NEG	NEG	NEG	NEG	NEG	NEG	NEG	NEG	NEG	NEG	POS	POS	NEG	NEG	NEG	NEG
2012_207_Z3467 (1010/12)	POS	POS	NEG	POS	POS	POS	POS	POS	POS	POS	POS	POS	POS	POS	NEG	NEG	NEG	NEG	NEG	NEG	NEG	NEG	NEG	NEG	NEG	POS	POS	NEG	NEG	NEG	NEG
2012_212_Z3471 (1261/12)	POS	POS	NEG	POS	POS	POS	POS	POS	POS	POS	NEG	POS	POS	POS	NEG	NEG	NEG	NEG	NEG	NEG	NEG	NEG	NEG	NEG	NEG	POS	POS	NEG	NEG	NEG	NEG
2012_220_Z3477 (1466/12)	POS	POS	NEG	POS	POS	POS	POS	POS	POS	POS	POS	NEG	NEG	NEG	NEG	NEG	NEG	NEG	NEG	NEG	NEG	NEG	NEG	NEG	NEG	POS	POS	NEG	NEG	NEG	NEG
2012_224_Z3481 (1522/12)	POS	POS	NEG	POS	POS	POS	POS	POS	POS	POS	NEG	POS	POS	POS	NEG	NEG	NEG	NEG	NEG	NEG	NEG	NEG	NEG	NEG	NEG	POS	POS	NEG	NEG	NEG	NEG
2012_226_Z3483 (1662/12)	POS	POS	NEG	POS	POS	POS	POS	POS	POS	POS	POS	POS	POS	POS	NEG	NEG	NEG	NEG	NEG	NEG	NEG	NEG	NEG	NEG	NEG	POS	POS	NEG	NEG	NEG	NEG
2012_242_Z3527 (2581/12)	POS	POS	NEG	POS	POS	POS	POS	POS	POS	POS	POS	POS	POS	POS	NEG	NEG	NEG	NEG	NEG	NEG	NEG	NEG	NEG	NEG	NEG	POS	POS	NEG	NEG	NEG	NEG
2012_245_Z3529 (2687/12)	POS	POS	NEG	POS	POS	POS	POS	POS	POS	POS	AMB	POS	POS	POS	NEG	NEG	NEG	NEG	NEG	NEG	NEG	NEG	NEG	NEG	NEG	POS	POS	NEG	NEG	NEG	NEG
2012_253_Z3536 (3074/12)	POS	POS	NEG	POS	POS	POS	POS	POS	POS	POS	AMB	POS	POS	POS	NEG	NEG	NEG	NEG	NEG	NEG	NEG	NEG	NEG	NEG	NEG	POS	POS	NEG	NEG	NEG	NEG
2012_258_Z3545 (3231/12)	POS	POS	NEG	POS	POS	POS	POS	POS	POS	POS	POS	NEG	NEG	NEG	NEG	NEG	NEG	NEG	NEG	NEG	NEG	NEG	NEG	NEG	NEG	POS	POS	NEG	NEG	NEG	NEG
2012_261_Z3548 (3298/12)	POS	POS	NEG	POS	POS	POS	POS	POS	POS	POS	POS	POS	POS	POS	NEG	NEG	NEG	NEG	POS	NEG	NEG	NEG	NEG	NEG	NEG	POS	POS	NEG	NEG	NEG	NEG
2012_270_Z3557 (3798/12)	POS	POS	NEG	POS	POS	POS	POS	POS	POS	POS	POS	POS	POS	POS	NEG	NEG	NEG	NEG	NEG	NEG	NEG	NEG	NEG	NEG	NEG	POS	POS	NEG	NEG	NEG	NEG
2012_274_Z3561 (3861/12)	POS	POS	NEG	POS	POS	POS	POS	POS	POS	POS	POS	POS	POS	POS	NEG	NEG	NEG	NEG	NEG	NEG	NEG	NEG	NEG	NEG	NEG	POS	POS	NEG	NEG	NEG	NEG
2012_282_Z3569 (4698/12)	POS	POS	NEG	POS	POS	POS	POS	POS	POS	POS	AMB	POS	POS	POS	NEG	NEG	NEG	NEG	NEG	NEG	NEG	NEG	NEG	NEG	NEG	POS	POS	NEG	NEG	NEG	NEG
2012_286_Z3573 (4805/12)	POS	POS	NEG	POS	POS	POS	POS	POS	POS	POS	AMB	POS	POS	POS	NEG	NEG	NEG	NEG	NEG	NEG	NEG	NEG	NEG	NEG	NEG	POS	POS	NEG	NEG	NEG	NEG
2012_293_Z3580 (5007/12)	POS	POS	NEG	POS	POS	POS	POS	POS	POS	POS	AMB	POS	POS	POS	NEG	NEG	NEG	NEG	NEG	NEG	NEG	NEG	NEG	NEG	NEG	POS	POS	NEG	NEG	NEG	NEG
2012_294_Z3581	POS	POS	NEG	POS	POS	POS	POS	POS	POS	POS	AMB	POS	POS	POS	NEG	NEG	NEG	NEG	NEG	NEG	NEG	NEG	NEG	NEG	NEG	POS	POS	NEG	NEG	NEG	NEG
2012_307_Z3594 (5918/12)	POS	POS	NEG	POS	POS	POS	POS	POS	POS	POS	AMB	POS	POS	POS	NEG	NEG	NEG	NEG	NEG	NEG	NEG	NEG	NEG	NEG	NEG	POS	POS	NEG	NEG	NEG	NEG
2012_333_Z3620 (8101/12)	POS	POS	NEG	POS	POS	POS	POS	POS	POS	POS	AMB	POS	POS	POS	NEG	NEG	NEG	NEG	NEG	NEG	NEG	NEG	NEG	NEG	NEG	POS	POS	NEG	NEG	NEG	NEG
2012_343_Z3630 (8195/12)	POS	POS	NEG	POS	POS	POS	POS	POS	POS	POS	POS	POS	POS	POS	NEG	NEG	NEG	NEG	NEG	NEG	NEG	NEG	NEG	NEG	NEG	POS	POS	NEG	NEG	NEG	NEG
2012_349_Z3636 (7999A/12)	POS	POS	NEG	POS	POS	POS	POS	POS	POS	POS	POS	POS	POS	POS	NEG	NEG	NEG	NEG	NEG	NEG	NEG	NEG	NEG	NEG	NEG	POS	POS	NEG	NEG	AMB	NEG
2012_353_Z3640 (8575/12)	POS	POS	NEG	POS	POS	POS	POS	POS	POS	POS	POS	POS	POS	POS	NEG	NEG	NEG	NEG	NEG	NEG	NEG	NEG	NEG	NEG	NEG	POS	POS	NEG	POS	POS	POS
2012_354_Z3641 (8408/12)	POS	POS	NEG	POS	POS	POS	POS	POS	POS	POS	POS	POS	POS	POS	NEG	NEG	NEG	NEG	NEG	NEG	NEG	NEG	NEG	NEG	NEG	POS	POS	NEG	NEG	NEG	NEG
2012_355_Z3642 (1112-58348)	POS	POS	NEG	POS	POS	POS	POS	POS	POS	POS	AMB	POS	POS	POS	NEG	NEG	NEG	NEG	NEG	NEG	NEG	NEG	NEG	NEG	NEG	POS	POS	NEG	NEG	NEG	NEG
2012_357_Z3417 (1202-6360)	POS	POS	NEG	POS	POS	POS	POS	POS	POS	POS	POS	POS	POS	POS	NEG	NEG	NEG	NEG	NEG	NEG	NEG	NEG	NEG	NEG	NEG	POS	POS	NEG	NEG	NEG	NEG
2012_364_Z3424 (1112-60417)	POS	POS	NEG	POS	POS	POS	POS	POS	POS	POS	POS	POS	POS	POS	NEG	NEG	NEG	NEG	NEG	NEG	NEG	NEG	NEG	NEG	NEG	POS	POS	NEG	NEG	NEG	NEG
2012_376_Z3436 (6256)	POS	POS	NEG	POS	POS	POS	POS	POS	POS	POS	POS	POS	POS	POS	NEG	NEG	NEG	NEG	NEG	NEG	NEG	NEG	NEG	NEG	NEG	POS	POS	NEG	NEG	NEG	NEG
2012_383_Z3443	POS	POS	NEG	POS	POS	POS	POS	POS	POS	POS	POS	POS	POS	POS	NEG	NEG	NEG	NEG	POS	NEG	NEG	NEG	NEG	NEG	NEG	POS	POS	NEG	NEG	NEG	NEG
2013_PA01_Z3984	POS	POS	NEG	POS	POS	POS	POS	POS	POS	POS	NEG	POS	POS	POS	NEG	NEG	NEG	NEG	NEG	NEG	NEG	NEG	NEG	NEG	NEG	POS	POS	NEG	NEG	NEG	NEG
2013_PA08_Z3991	POS	POS	NEG	POS	POS	POS	POS	POS	POS	POS	NEG	POS	POS	POS	NEG	NEG	NEG	NEG	NEG	NEG	NEG	NEG	NEG	NEG	NEG	POS	POS	NEG	NEG	NEG	NEG
2013_PA10_Z3993	POS	POS	NEG	POS	POS	POS	POS	POS	POS	POS	NEG	POS	POS	POS	NEG	NEG	NEG	NEG	NEG	NEG	NEG	NEG	NEG	NEG	NEG	POS	POS	NEG	NEG	NEG	NEG
2013_PA13_Z3996	POS	POS	NEG	POS	POS	POS	POS	POS	POS	POS	NEG	POS	POS	POS	NEG	NEG	NEG	NEG	NEG	NEG	NEG	NEG	NEG	NEG	NEG	POS	POS	NEG	NEG	NEG	NEG
2013_PA14_Z3997	POS	POS	NEG	POS	POS	POS	POS	POS	POS	POS	NEG	POS	POS	POS	NEG	NEG	NEG	NEG	NEG	NEG	NEG	NEG	NEG	NEG	NEG	POS	POS	NEG	NEG	NEG	NEG
2013_PA17_Z4000	POS	POS	NEG	POS	POS	POS	POS	POS	POS	POS	NEG	POS	POS	POS	NEG	NEG	NEG	NEG	NEG	NEG	NEG	NEG	NEG	NEG	NEG	POS	POS	NEG	NEG	NEG	NEG
2013_PA26_Z4009	POS	POS	NEG	POS	POS	POS	POS	POS	POS	POS	POS	POS	POS	POS	NEG	NEG	NEG	NEG	NEG	NEG	NEG	NEG	NEG	NEG	NEG	POS	POS	NEG	NEG	NEG	NEG
2013_PA27_Z4010	POS	POS	NEG	POS	POS	POS	POS	POS	POS	POS	AMB	POS	POS	POS	NEG	NEG	NEG	NEG	NEG	NEG	NEG	NEG	NEG	NEG	NEG	POS	POS	NEG	NEG	NEG	NEG
2013_PA42_Z4025	POS	POS	NEG	POS	POS	POS	POS	POS	POS	POS	NEG	POS	POS	POS	NEG	NEG	NEG	NEG	NEG	NEG	NEG	NEG	NEG	NEG	NEG	POS	POS	NEG	NEG	NEG	NEG
2013_PA43_Z4026	POS	POS	NEG	POS	POS	POS	POS	POS	POS	POS	NEG	POS	POS	POS	NEG	NEG	NEG	NEG	NEG	NEG	NEG	NEG	NEG	NEG	NEG	POS	POS	NEG	NEG	NEG	NEG
2013_PA45_Z4028	POS	POS	NEG	POS	POS	POS	POS	POS	POS	POS	NEG	POS	POS	POS	NEG	NEG	NEG	NEG	NEG	NEG	NEG	NEG	NEG	NEG	NEG	POS	POS	NEG	NEG	NEG	NEG
2013_PA56_Z4039	POS	POS	NEG	POS	POS	POS	POS	POS	POS	POS	NEG	POS	POS	POS	NEG	NEG	NEG	NEG	NEG	NEG	NEG	NEG	NEG	NEG	NEG	POS	POS	NEG	NEG	NEG	NEG
2013_PA58_Z4041	POS	POS	NEG	POS	POS	POS	POS	POS	POS	POS	POS	POS	POS	POS	NEG	NEG	NEG	NEG	NEG	NEG	NEG	NEG	NEG	NEG	NEG	POS	POS	NEG	NEG	NEG	NEG
2013_PA64_Z4047	POS	POS	NEG	POS	POS	POS	POS	POS	POS	POS	AMB	POS	POS	POS	NEG	NEG	NEG	NEG	NEG	NEG	NEG	NEG	NEG	NEG	NEG	POS	POS	NEG	NEG	NEG	NEG
2013_PA66_Z4049	POS	POS	NEG	POS	POS	POS	POS	POS	POS	POS	NEG	POS	POS	POS	NEG	NEG	NEG	NEG	NEG	NEG	NEG	NEG	NEG	NEG	NEG	POS	POS	NEG	NEG	NEG	NEG
2013_PA67_Z4050	POS	POS	NEG	POS	POS	POS	POS	POS	POS	POS	AMB	POS	POS	POS	NEG	NEG	NEG	NEG	NEG	NEG	NEG	NEG	NEG	NEG	NEG	POS	POS	NEG	NEG	NEG	NEG
2013_PA69_Z4052	POS	POS	NEG	POS	POS	POS	POS	POS	POS	POS	NEG	POS	POS	POS	NEG	NEG	NEG	NEG	NEG	NEG	NEG	NEG	NEG	NEG	NEG	POS	POS	NEG	NEG	NEG	NEG
2013_PA72_Z4055	POS	POS	NEG	POS	POS	POS	POS	POS	POS	POS	AMB	POS	POS	POS	NEG	NEG	NEG	NEG	NEG	NEG	NEG	NEG	NEG	NEG	NEG	POS	POS	NEG	NEG	NEG	NEG
2013_PA76_Z4059	POS	POS	NEG	POS	POS	POS	POS	POS	POS	POS	AMB	POS	POS	POS	NEG	NEG	NEG	NEG	NEG	NEG	NEG	NEG	NEG	NEG	NEG	POS	POS	NEG	NEG	NEG	NEG
2013_PA80_Z4063	POS	POS	NEG	POS	POS	POS	POS	POS	POS	POS	POS	NEG	NEG	NEG	NEG	NEG	NEG	NEG	NEG	NEG	NEG	NEG	NEG	NEG	NEG	POS	POS	NEG	NEG	NEG	NEG

Strain / Isolates	leukocidin/ haemolysin toxin family protein	leukocidin/haemolysin toxin family protein		putative membrane protein	haemolysin alpha	putative membrane protein		haemolysin beta				staphylo-kinase	chemotaxis-inhibiting protein (CHIPS)	Staphyl. Comple-ment inhibitor	exfoliative toxin serotype A	exfoliative toxin serotype B	exfoliative toxin D	epidermal cell differen-tiation inhibitor	epidermal cell differen-tiation inhibitor B	epidermal cell differen-tiation inhibitor C	ACME-locus	ACME-locus	ACME-locus: ornithincarb-amoyltrans-ferase	ACME-locus: carbamat-kinase	ACME-locus: arginine/orni-thine-antiporter	aureolysin			serin- protease A	serin- protease B	serin- protease E
	lukX	lukY	lukY (ST30+ST45)	hl	hla	hlIII (consensus)	hlIII (other than RF122)	hlb_probe 1	hlb_probe 2	hlb_probe 3	un-disrupted hlb	sak	chp	scn	etA	etB	etD	edinA	edinB	edinC	ACME (total)	arcA-SCC	arcB-SCC	arcC-SCC	arcD-SCC	aur (consensus)	aur (other than MRSA252)	aur (MRSA252)	splA	splB	splE
**ST8-MRSA-IV, USA300**																															
2012_158_Z3491 (26049A/11)	POS	POS	NEG	POS	POS	POS	POS	POS	POS	POS	AMB	POS	POS	POS	NEG	NEG	NEG	NEG	NEG	NEG	POS	POS	POS	POS	POS	POS	POS	NEG	POS	POS	POS
2012_165_Z3513 (26656/11)	POS	POS	NEG	POS	POS	POS	POS	POS	POS	POS	AMB	POS	POS	POS	NEG	NEG	NEG	NEG	NEG	NEG	POS	POS	POS	POS	POS	POS	POS	NEG	POS	POS	NEG
2012_214_Z3473 (1322/12)	POS	POS	NEG	POS	POS	POS	POS	POS	POS	POS	NEG	POS	POS	POS	NEG	NEG	NEG	NEG	NEG	NEG	POS	POS	POS	POS	POS	POS	POS	NEG	POS	POS	POS
2012_227_Z3484 (1688/12)	POS	POS	NEG	POS	POS	POS	POS	POS	POS	POS	POS	POS	POS	POS	NEG	NEG	NEG	NEG	NEG	NEG	POS	POS	POS	POS	POS	POS	POS	NEG	POS	POS	POS
2012_263_Z3550 (3355/12)	POS	POS	NEG	POS	POS	POS	POS	POS	POS	POS	POS	POS	POS	POS	NEG	NEG	NEG	NEG	NEG	NEG	POS	POS	POS	POS	POS	POS	POS	NEG	POS	POS	POS
2012_266_Z3553	POS	POS	NEG	POS	POS	POS	POS	POS	POS	POS	POS	POS	POS	POS	NEG	NEG	NEG	NEG	NEG	NEG	POS	POS	POS	POS	POS	POS	POS	NEG	POS	POS	POS
2012_284_Z3571 (4729/12)	POS	POS	NEG	POS	POS	POS	POS	POS	POS	POS	AMB	POS	POS	POS	NEG	NEG	NEG	NEG	NEG	NEG	POS	POS	POS	POS	POS	POS	POS	NEG	POS	POS	POS
2012_298_Z3585 (5203/12)	POS	POS	NEG	POS	POS	POS	POS	POS	POS	POS	AMB	POS	POS	POS	NEG	NEG	NEG	AMB	NEG	NEG	POS	POS	POS	POS	POS	POS	POS	NEG	POS	POS	POS
2012_304_Z3591 (5818/12)	POS	POS	NEG	POS	POS	POS	POS	POS	POS	POS	AMB	POS	POS	POS	NEG	NEG	NEG	NEG	NEG	NEG	POS	POS	POS	POS	POS	POS	POS	NEG	POS	POS	POS
Z3595_TT308 - (EFC74877-3925-484F-A4CF-A1F7DC	POS	POS	NEG	POS	POS	POS	POS	POS	POS	POS	NEG	POS	POS	POS	NEG	NEG	NEG	NEG	NEG	NEG	POS	POS	POS	POS	POS	POS	POS	NEG	POS	POS	POS
2012_369_Z3429 (1112-58547)	POS	POS	NEG	POS	POS	POS	POS	POS	POS	POS	POS	POS	POS	POS	NEG	NEG	NEG	NEG	NEG	NEG	POS	POS	POS	POS	POS	POS	POS	NEG	POS	POS	POS
2012_374_Z3434 (1203-13527)	POS	POS	NEG	POS	POS	POS	POS	POS	POS	POS	AMB	POS	POS	POS	NEG	NEG	NEG	NEG	NEG	NEG	POS	POS	POS	POS	POS	POS	POS	NEG	POS	POS	POS
2013_PA06_Z3989	POS	POS	NEG	POS	POS	POS	POS	POS	POS	POS	NEG	POS	POS	POS	NEG	NEG	NEG	NEG	NEG	NEG	POS	POS	POS	POS	POS	POS	POS	NEG	POS	POS	POS
2013_PA55_Z4038	POS	POS	NEG	POS	POS	POS	POS	POS	POS	POS	AMB	POS	POS	POS	NEG	NEG	NEG	NEG	NEG	NEG	POS	POS	POS	POS	POS	POS	POS	NEG	POS	POS	POS
2013_PA71_Z4054	POS	POS	NEG	POS	POS	POS	POS	POS	POS	POS	POS	NEG	NEG	NEG	NEG	NEG	NEG	NEG	NEG	NEG	POS	POS	POS	POS	POS	POS	POS	NEG	POS	POS	POS
**ST8-MRSA-IV [PVL+/ACME+], US**																															
2012_180_Z3501 (232/12)	POS	POS	NEG	POS	POS	POS	POS	POS	POS	POS	NEG	POS	POS	POS	NEG	NEG	NEG	NEG	NEG	NEG	POS	POS	POS	POS	POS	POS	POS	NEG	POS	POS	POS
2013_PA68_Z4051	POS	POS	NEG	POS	POS	POS	POS	POS	POS	POS	AMB	POS	POS	POS	NEG	NEG	NEG	NEG	NEG	NEG	POS	POS	POS	POS	POS	POS	POS	NEG	POS	POS	POS
**ST72-MSSA**																															
2012_174_Z3451 (102/12)	POS	POS	NEG	POS	POS	POS	POS	POS	POS	POS	POS	NEG	NEG	NEG	NEG	NEG	NEG	NEG	NEG	NEG	NEG	NEG	NEG	NEG	NEG	POS	POS	NEG	POS	POS	POS
2012_223_Z3480 (1506A/12)	POS	POS	NEG	POS	POS	POS	POS	POS	POS	POS	AMB	POS	POS	POS	NEG	NEG	NEG	NEG	NEG	NEG	NEG	NEG	NEG	NEG	NEG	POS	POS	NEG	POS	POS	POS
2012_236_Z3523 (2086/12)	POS	POS	NEG	POS	POS	POS	POS	POS	POS	POS	AMB	POS	POS	POS	NEG	NEG	NEG	NEG	NEG	NEG	NEG	NEG	NEG	NEG	NEG	POS	POS	NEG	POS	POS	POS
2012_239_Z3525 (2346/12)	POS	POS	NEG	POS	POS	POS	POS	POS	POS	POS	AMB	POS	POS	POS	NEG	NEG	NEG	NEG	NEG	NEG	NEG	NEG	NEG	NEG	NEG	POS	POS	NEG	POS	POS	NEG
2012_252_Z3535 (3030/12)	POS	POS	NEG	POS	POS	POS	POS	POS	POS	POS	POS	POS	POS	POS	NEG	NEG	NEG	NEG	NEG	NEG	NEG	NEG	NEG	NEG	NEG	POS	POS	NEG	POS	POS	NEG
2012_271_Z3558 (3799/12)	POS	POS	NEG	POS	POS	POS	POS	POS	POS	POS	POS	POS	POS	POS	NEG	NEG	NEG	NEG	NEG	NEG	NEG	NEG	NEG	NEG	NEG	POS	POS	NEG	POS	POS	POS
2012_276_Z3563	POS	POS	NEG	POS	POS	POS	POS	POS	POS	POS	POS	POS	POS	POS	NEG	NEG	NEG	NEG	NEG	NEG	NEG	NEG	NEG	NEG	NEG	POS	POS	NEG	POS	POS	POS
2012_300_Z3587 (5638/12)	POS	POS	NEG	POS	POS	POS	POS	POS	POS	POS	AMB	POS	POS	POS	NEG	NEG	NEG	NEG	NEG	NEG	NEG	NEG	NEG	NEG	NEG	POS	POS	NEG	POS	POS	POS
2012_316_Z3603 (6941/12)	POS	POS	NEG	POS	POS	POS	POS	POS	POS	POS	AMB	POS	POS	POS	NEG	NEG	NEG	NEG	NEG	NEG	NEG	NEG	NEG	NEG	NEG	POS	POS	NEG	POS	POS	POS
2012_319_Z3606 (7019/12)	POS	POS	NEG	POS	POS	POS	POS	POS	POS	POS	AMB	POS	POS	POS	NEG	NEG	NEG	NEG	NEG	NEG	NEG	NEG	NEG	NEG	NEG	POS	POS	NEG	POS	POS	POS
2012_371_Z3431 (1204-17577)	POS	POS	NEG	POS	POS	POS	POS	POS	POS	POS	POS	POS	POS	POS	NEG	NEG	NEG	NEG	NEG	NEG	NEG	NEG	NEG	NEG	NEG	POS	POS	NEG	POS	POS	POS
2013_PA05_Z3988	POS	POS	NEG	POS	POS	POS	POS	POS	POS	POS	POS	NEG	NEG	NEG	NEG	NEG	NEG	NEG	NEG	NEG	NEG	NEG	NEG	NEG	NEG	POS	POS	NEG	POS	POS	POS
2013_PA65_Z4048	POS	POS	NEG	POS	POS	POS	POS	POS	POS	POS	NEG	POS	POS	POS	NEG	NEG	NEG	NEG	NEG	NEG	NEG	NEG	NEG	NEG	NEG	POS	POS	NEG	POS	POS	POS
2013_PA73_Z4056	POS	POS	NEG	POS	POS	POS	POS	POS	POS	POS	POS	POS	POS	POS	NEG	NEG	NEG	NEG	NEG	NEG	NEG	NEG	NEG	NEG	NEG	POS	POS	NEG	NEG	NEG	NEG
**ST72-MSSA-SCCfus**																															
2013_PA16_Z3999	POS	POS	NEG	POS	POS	POS	POS	POS	POS	POS	POS	POS	POS	POS	NEG	NEG	NEG	NEG	NEG	NEG	NEG	NEG	NEG	NEG	NEG	POS	POS	NEG	POS	POS	POS
**ST72-MSSA-SCCfus/kdp**																															
2012_361_Z3421 (1201-1299)	POS	POS	NEG	POS	POS	POS	POS	POS	POS	POS	NEG	POS	POS	POS	NEG	NEG	NEG	NEG	NEG	NEG	NEG	NEG	NEG	NEG	NEG	POS	POS	NEG	POS	POS	POS
**ST239-MRSA-III&SCCmer**																															
2012_157_Z3490	POS	POS	NEG	POS	POS	POS	POS	POS	POS	POS	AMB	POS	NEG	POS	NEG	NEG	NEG	NEG	NEG	NEG	NEG	NEG	NEG	NEG	NEG	POS	NEG	POS	POS	POS	POS
2012_168_Z3494 (27129/11)	POS	POS	NEG	POS	POS	POS	POS	POS	POS	POS	AMB	POS	NEG	POS	NEG	NEG	NEG	NEG	NEG	NEG	NEG	NEG	NEG	NEG	NEG	POS	NEG	POS	POS	POS	POS
2012_195_Z3508 (619/12)	POS	POS	NEG	POS	POS	POS	POS	POS	POS	POS	POS	NEG	NEG	NEG	NEG	NEG	NEG	NEG	NEG	NEG	NEG	NEG	NEG	NEG	NEG	POS	NEG	POS	POS	POS	POS
2012_222_Z3479 (1489/12)	POS	POS	NEG	POS	POS	POS	POS	POS	POS	POS	NEG	POS	NEG	POS	NEG	NEG	NEG	NEG	NEG	NEG	NEG	NEG	NEG	NEG	NEG	POS	NEG	POS	POS	POS	POS
2012_233_Z3540 (1943/12)	POS	POS	NEG	POS	POS	POS	POS	POS	POS	POS	AMB	POS	NEG	POS	NEG	NEG	NEG	NEG	NEG	NEG	NEG	NEG	NEG	NEG	NEG	POS	NEG	POS	POS	POS	POS
2012_237_Z3541	POS	POS	NEG	POS	POS	POS	POS	POS	POS	POS	POS	POS	NEG	POS	NEG	NEG	NEG	NEG	NEG	NEG	NEG	NEG	NEG	NEG	NEG	POS	NEG	POS	POS	POS	POS
2012_262_Z3549	POS	POS	NEG	POS	POS	POS	POS	POS	POS	POS	POS	POS	NEG	POS	NEG	NEG	NEG	NEG	NEG	NEG	NEG	NEG	NEG	NEG	NEG	POS	NEG	POS	POS	POS	POS
2012_275_Z3562 (3893/12)	POS	POS	NEG	POS	POS	POS	POS	POS	POS	POS	AMB	POS	NEG	POS	NEG	NEG	NEG	NEG	NEG	NEG	NEG	NEG	NEG	NEG	NEG	POS	NEG	POS	POS	POS	POS
2012_291_Z3578	POS	POS	NEG	POS	POS	POS	POS	POS	POS	POS	AMB	POS	NEG	POS	NEG	NEG	NEG	NEG	NEG	NEG	NEG	NEG	NEG	NEG	NEG	POS	NEG	POS	POS	POS	POS
2012_314_Z3601 (6939/12)	POS	POS	NEG	POS	POS	POS	POS	POS	POS	POS	POS	POS	AMB	POS	NEG	NEG	NEG	NEG	NEG	NEG	NEG	NEG	AMB	NEG	NEG	POS	NEG	POS	POS	POS	POS
2012323_Z3610	POS	POS	NEG	POS	POS	POS	POS	POS	POS	POS	POS	POS	NEG	POS	NEG	NEG	NEG	NEG	NEG	NEG	NEG	NEG	NEG	NEG	NEG	POS	NEG	POS	POS	POS	POS
2012330_Z3617 (7605/12)	POS	POS	NEG	POS	POS	POS	POS	POS	POS	POS	POS	POS	NEG	POS	NEG	NEG	NEG	NEG	NEG	NEG	NEG	NEG	NEG	NEG	NEG	POS	NEG	POS	POS	POS	POS
2012332_Z3619 (8076/12)	POS	POS	NEG	POS	POS	POS	POS	POS	POS	POS	AMB	POS	NEG	POS	NEG	NEG	NEG	NEG	NEG	NEG	NEG	NEG	NEG	NEG	NEG	POS	NEG	POS	POS	POS	POS
2012339_Z3626 (8558/12)	POS	POS	NEG	POS	POS	POS	POS	POS	POS	POS	AMB	POS	NEG	POS	NEG	NEG	NEG	NEG	NEG	NEG	NEG	NEG	NEG	NEG	NEG	POS	NEG	POS	POS	POS	POS
2012340_Z3627 (8523/12)	POS	POS	NEG	POS	POS	POS	POS	POS	POS	POS	AMB	POS	NEG	POS	NEG	NEG	NEG	NEG	NEG	NEG	NEG	NEG	NEG	NEG	NEG	POS	NEG	POS	POS	POS	POS
2012348_Z3635	POS	POS	NEG	POS	POS	POS	POS	POS	POS	POS	AMB	POS	NEG	POS	NEG	NEG	NEG	NEG	NEG	NEG	NEG	NEG	NEG	NEG	NEG	POS	NEG	POS	POS	POS	POS
2012350_Z3637	POS	POS	NEG	POS	POS	POS	POS	POS	POS	POS	POS	NEG	NEG	NEG	NEG	NEG	NEG	NEG	NEG	NEG	NEG	NEG	NEG	NEG	NEG	POS	NEG	POS	POS	POS	POS
2012_370_Z3430 (1112-52177)	POS	POS	NEG	POS	POS	POS	POS	POS	POS	POS	POS	NEG	NEG	NEG	NEG	NEG	NEG	NEG	NEG	NEG	NEG	NEG	NEG	NEG	NEG	POS	NEG	POS	POS	POS	POS
2012_378_Z3438 (4139)	POS	POS	NEG	POS	POS	POS	POS	POS	POS	POS	NEG	POS	NEG	POS	NEG	NEG	NEG	NEG	NEG	NEG	NEG	NEG	NEG	NEG	NEG	POS	NEG	POS	POS	POS	POS
2013_PA21_Z4004	POS	POS	NEG	POS	POS	POS	POS	POS	POS	POS	NEG	POS	NEG	POS	NEG	NEG	NEG	NEG	NEG	NEG	NEG	NEG	NEG	NEG	NEG	POS	NEG	POS	POS	POS	POS
2013_PA22_Z4005	POS	POS	NEG	POS	POS	POS	POS	POS	POS	POS	AMB	POS	NEG	POS	NEG	NEG	NEG	NEG	NEG	NEG	NEG	NEG	NEG	NEG	NEG	POS	NEG	POS	POS	POS	POS
2013_PA24_Z4007	POS	POS	NEG	POS	POS	POS	POS	POS	POS	POS	NEG	POS	NEG	POS	NEG	NEG	NEG	NEG	NEG	NEG	NEG	NEG	NEG	NEG	NEG	POS	NEG	POS	POS	POS	POS
2013_PA31_Z4014	POS	POS	NEG	POS	POS	POS	POS	POS	POS	POS	AMB	POS	NEG	POS	NEG	NEG	NEG	NEG	NEG	NEG	NEG	NEG	NEG	NEG	NEG	POS	NEG	POS	POS	POS	POS
2013_PA35_Z4018	POS	POS	NEG	POS	POS	POS	POS	POS	POS	POS	NEG	POS	NEG	POS	NEG	NEG	NEG	NEG	NEG	NEG	NEG	NEG	NEG	NEG	NEG	POS	NEG	POS	POS	POS	POS
2013_PA81_Z4064	POS	POS	NEG	POS	POS	POS	POS	POS	POS	POS	POS	NEG	NEG	NEG	NEG	NEG	NEG	NEG	NEG	NEG	NEG	NEG	NEG	NEG	NEG	POS	NEG	POS	POS	POS	POS
**ST239-MRSA-III+ccrA/B4**																															
2012_254_Z3537 (3124A/12)	POS	POS	NEG	POS	POS	POS	POS	POS	POS	POS	POS	NEG	NEG	NEG	NEG	NEG	NEG	NEG	NEG	NEG	NEG	NEG	NEG	NEG	NEG	POS	NEG	POS	POS	POS	POS
2013_PA25_Z4008	POS	POS	NEG	POS	POS	POS	POS	POS	POS	POS	POS	NEG	NEG	NEG	NEG	NEG	NEG	NEG	NEG	NEG	NEG	NEG	NEG	NEG	NEG	POS	NEG	POS	POS	POS	POS
**ST834-MSSA**																															
2012_268_Z3555 (3693/12)	POS	POS	NEG	POS	POS	POS	POS	POS	POS	POS	POS	NEG	NEG	POS	NEG	NEG	NEG	NEG	NEG	NEG	NEG	NEG	NEG	NEG	NEG	POS	POS	NEG	POS	POS	POS
**CC12-MSSA**																															
2012_210_Z3470 (1177/12)	POS	POS	NEG	POS	POS	POS	POS	POS	POS	POS	AMB	POS	NEG	POS	NEG	NEG	NEG	NEG	NEG	NEG	NEG	NEG	NEG	NEG	NEG	POS	POS	NEG	POS	POS	POS
2012_230_Z3487 (1797/12)	POS	POS	NEG	POS	POS	POS	POS	POS	POS	POS	POS	POS	POS	POS	NEG	NEG	NEG	NEG	NEG	NEG	NEG	NEG	NEG	NEG	NEG	POS	POS	NEG	POS	POS	POS
2013_PA18_Z4001	POS	POS	NEG	POS	POS	POS	POS	POS	POS	POS	NEG	POS	POS	POS	NEG	NEG	NEG	NEG	NEG	NEG	NEG	NEG	NEG	NEG	NEG	POS	POS	NEG	POS	POS	POS
**CC15-MSSA**																															
2012_160_Z3444 (26386/11)	POS	POS	NEG	POS	POS	POS	POS	NEG	NEG	POS	AMB	NEG	POS	POS	NEG	NEG	NEG	NEG	NEG	NEG	NEG	NEG	NEG	NEG	NEG	POS	POS	NEG	POS	POS	POS
2012_167_Z3514 (27010/11)	POS	POS	NEG	POS	POS	POS	POS	NEG	NEG	POS	AMB	POS	POS	POS	NEG	NEG	NEG	NEG	NEG	NEG	NEG	NEG	NEG	NEG	NEG	POS	POS	NEG	POS	POS	POS
2012_184_Z3455 (376/12)	POS	POS	NEG	POS	POS	POS	POS	NEG	NEG	POS	AMB	NEG	POS	POS	NEG	NEG	NEG	NEG	NEG	NEG	NEG	NEG	NEG	NEG	NEG	POS	POS	NEG	POS	POS	POS
2012_205_Z3465 (939/12)	POS	POS	NEG	POS	POS	POS	POS	NEG	NEG	POS	POS	NEG	POS	POS	NEG	NEG	NEG	NEG	NEG	NEG	NEG	NEG	NEG	NEG	NEG	POS	POS	NEG	POS	POS	POS
2012_231_Z3519 (1831/12)	POS	POS	NEG	POS	POS	POS	POS	NEG	NEG	POS	AMB	NEG	POS	POS	NEG	NEG	NEG	NEG	NEG	NEG	NEG	NEG	NEG	NEG	NEG	POS	POS	NEG	POS	POS	POS
2012_249_Z3533 (2907/12)	POS	POS	NEG	POS	POS	POS	POS	NEG	NEG	POS	AMB	NEG	POS	POS	NEG	NEG	NEG	NEG	NEG	NEG	NEG	NEG	NEG	NEG	NEG	POS	POS	NEG	POS	POS	POS
2012_305_Z3592 (5839/12)	POS	POS	NEG	POS	POS	POS	POS	NEG	NEG	POS	POS	NEG	POS	POS	NEG	NEG	NEG	NEG	NEG	NEG	NEG	NEG	NEG	NEG	NEG	POS	POS	NEG	POS	POS	POS
2012_313_Z3600 (6857/12)	POS	POS	NEG	POS	POS	POS	POS	NEG	NEG	POS	AMB	NEG	POS	POS	NEG	NEG	NEG	NEG	NEG	NEG	NEG	NEG	NEG	NEG	NEG	POS	POS	NEG	POS	POS	POS
2012_345_Z3632 (79668/12)	POS	POS	NEG	POS	POS	POS	POS	NEG	NEG	POS	AMB	POS	POS	POS	NEG	NEG	NEG	NEG	NEG	NEG	NEG	NEG	NEG	NEG	NEG	POS	POS	NEG	POS	POS	POS
2013_PA33_Z4016	POS	POS	NEG	POS	POS	POS	POS	NEG	NEG	AMB	NEG	NEG	POS	POS	NEG	NEG	NEG	NEG	NEG	NEG	NEG	NEG	NEG	NEG	NEG	POS	POS	NEG	POS	POS	POS
**CC20-MSSA**																															
2012_240_Z3526 (2354/12)	POS	POS	NEG	POS	POS	POS	POS	POS	POS	POS	AMB	POS	POS	POS	NEG	NEG	NEG	NEG	NEG	NEG	NEG	NEG	NEG	NEG	NEG	POS	POS	NEG	POS	POS	AMB
**CC30-MSSA**																															
2012_155_Z3488 (25905A/11)	POS	NEG	POS	POS	POS	POS	POS	NEG	NEG	NEG	AMB	NEG	NEG	NEG	NEG	NEG	NEG	NEG	NEG	NEG	NEG	NEG	NEG	NEG	NEG	POS	NEG	POS	NEG	NEG	POS
**CC30-MSSA [PVL+]**																															
2012_164_Z3447 (26574/11)	POS	NEG	POS	POS	POS	POS	POS	POS	POS	POS	POS	POS	POS	POS	NEG	NEG	NEG	NEG	NEG	NEG	NEG	NEG	NEG	NEG	NEG	POS	NEG	POS	NEG	NEG	POS
2012_166_Z3493 (26988A/11)	POS	NEG	POS	POS	POS	POS	POS	POS	POS	POS	AMB	POS	POS	POS	NEG	NEG	NEG	NEG	NEG	NEG	NEG	NEG	NEG	NEG	NEG	POS	NEG	POS	NEG	NEG	POS
2012_178_Z3500 (197/12)	POS	NEG	POS	POS	POS	POS	POS	POS	POS	POS	NEG	POS	POS	POS	NEG	NEG	NEG	NEG	NEG	NEG	NEG	NEG	NEG	NEG	NEG	POS	NEG	POS	NEG	NEG	POS
2012_183_Z3454	POS	NEG	POS	POS	POS	POS	POS	POS	POS	POS	AMB	POS	POS	POS	NEG	NEG	NEG	NEG	NEG	NEG	NEG	NEG	NEG	NEG	NEG	POS	NEG	POS	NEG	NEG	POS
2012_186_Z3503 (444/12)	POS	NEG	POS	POS	POS	POS	POS	POS	POS	POS	NEG	POS	POS	POS	NEG	NEG	NEG	NEG	NEG	NEG	NEG	NEG	NEG	NEG	NEG	POS	NEG	POS	NEG	NEG	POS
2012_189_Z3506 (542/12)	POS	NEG	POS	POS	POS	POS	POS	POS	POS	POS	AMB	POS	POS	POS	NEG	NEG	NEG	NEG	NEG	NEG	NEG	NEG	NEG	NEG	NEG	POS	NEG	POS	NEG	NEG	POS
2012_192_Z3457 (606/12)	POS	NEG	POS	POS	POS	POS	POS	POS	POS	POS	AMB	POS	POS	POS	NEG	NEG	NEG	NEG	NEG	NEG	NEG	NEG	NEG	NEG	NEG	POS	NEG	POS	NEG	NEG	POS
2012_196_Z3509 (707/12)	POS	NEG	POS	POS	POS	POS	POS	POS	POS	POS	POS	POS	NEG	POS	NEG	NEG	NEG	NEG	NEG	NEG	NEG	NEG	NEG	NEG	AMB	POS	NEG	POS	NEG	NEG	POS
2012_218_Z3476 (1432/12)	POS	NEG	POS	POS	POS	POS	POS	POS	POS	POS	AMB	POS	POS	POS	NEG	NEG	NEG	NEG	NEG	NEG	NEG	NEG	NEG	NEG	NEG	POS	NEG	POS	NEG	NEG	POS
2012_232_Z3520 (1892/12)	POS	NEG	POS	POS	POS	POS	POS	POS	POS	POS	AMB	POS	POS	POS	NEG	NEG	NEG	NEG	NEG	NEG	NEG	NEG	NEG	NEG	NEG	POS	NEG	POS	NEG	NEG	POS
2012_278_Z3565 (4397/12)	POS	NEG	POS	POS	POS	POS	POS	POS	POS	POS	POS	POS	POS	POS	NEG	NEG	NEG	NEG	NEG	NEG	NEG	NEG	NEG	NEG	NEG	POS	NEG	POS	NEG	NEG	POS
2012_285_Z3572 (4802/12)	POS	NEG	POS	POS	POS	POS	POS	POS	POS	POS	POS	NEG	NEG	NEG	NEG	NEG	NEG	NEG	NEG	NEG	NEG	NEG	NEG	NEG	NEG	POS	NEG	POS	NEG	NEG	POS
2012_287_Z3574 (4906/12)	POS	NEG	POS	POS	POS	POS	POS	POS	POS	POS	AMB	POS	NEG	POS	NEG	NEG	NEG	NEG	NEG	NEG	NEG	NEG	NEG	NEG	NEG	POS	NEG	POS	NEG	NEG	POS
2012_295_Z3582 (5116/12)	POS	NEG	POS	POS	POS	POS	POS	POS	POS	POS	AMB	POS	POS	POS	NEG	NEG	NEG	NEG	NEG	NEG	NEG	NEG	NEG	NEG	NEG	POS	NEG	POS	NEG	NEG	POS
2012_326_Z3613 (7321/12)	POS	NEG	POS	POS	POS	POS	POS	POS	POS	POS	POS	POS	POS	POS	NEG	NEG	NEG	NEG	NEG	NEG	NEG	NEG	NEG	NEG	AMB	POS	NEG	POS	NEG	NEG	POS
2012_356_Z3643 (1204-15897)	POS	NEG	POS	POS	POS	POS	POS	POS	POS	POS	AMB	POS	NEG	POS	NEG	NEG	NEG	NEG	NEG	NEG	NEG	NEG	NEG	NEG	NEG	POS	NEG	POS	NEG	NEG	POS
2012_366_Z3426 (1204-16124)	POS	NEG	POS	POS	POS	POS	POS	POS	POS	POS	AMB	POS	NEG	POS	NEG	NEG	NEG	NEG	NEG	NEG	NEG	NEG	NEG	NEG	NEG	POS	NEG	POS	NEG	NEG	POS
2012_372_Z3432 (1205-19821)	POS	NEG	POS	POS	POS	POS	POS	POS	POS	POS	POS	NEG	NEG	NEG	NEG	NEG	NEG	NEG	NEG	NEG	NEG	NEG	NEG	NEG	NEG	POS	NEG	POS	NEG	NEG	POS
2012_380_Z3440 (4141)	POS	NEG	POS	POS	POS	POS	POS	POS	POS	POS	AMB	POS	POS	POS	NEG	NEG	NEG	NEG	NEG	NEG	NEG	NEG	NEG	NEG	NEG	POS	NEG	POS	NEG	NEG	POS
2013_PA20_Z4003	POS	NEG	POS	POS	POS	POS	POS	POS	POS	POS	NEG	POS	POS	POS	NEG	NEG	NEG	NEG	NEG	NEG	NEG	NEG	NEG	NEG	NEG	POS	NEG	POS	NEG	NEG	POS
2013_PA36_Z4019	POS	NEG	POS	POS	AMB	POS	POS	POS	POS	AMB	NEG	POS	POS	AMB	NEG	NEG	NEG	NEG	NEG	NEG	NEG	NEG	NEG	NEG	NEG	POS	NEG	POS	NEG	NEG	POS
2013_PA46_Z4029	AMB	NEG	POS	POS	AMB	POS	POS	POS	POS	AMB	NEG	POS	POS	AMB	NEG	NEG	NEG	NEG	NEG	NEG	NEG	NEG	NEG	NEG	NEG	POS	NEG	POS	NEG	NEG	AMB
2013_PA50_Z4033	POS	NEG	POS	POS	POS	POS	POS	POS	POS	AMB	NEG	POS	POS	POS	NEG	NEG	NEG	NEG	NEG	NEG	NEG	NEG	NEG	NEG	NEG	POS	NEG	POS	NEG	NEG	POS
2013_PA63_Z4046	POS	NEG	POS	POS	POS	POS	POS	POS	POS	POS	NEG	POS	POS	POS	NEG	NEG	NEG	NEG	NEG	NEG	NEG	NEG	NEG	NEG	NEG	POS	NEG	POS	NEG	NEG	POS
2013_PA78_Z4061	POS	NEG	POS	POS	POS	POS	POS	POS	POS	POS	NEG	POS	POS	POS	NEG	NEG	NEG	NEG	NEG	NEG	NEG	NEG	NEG	NEG	NEG	POS	NEG	POS	NEG	NEG	POS
2013_PA79_Z4062	POS	NEG	POS	POS	POS	POS	POS	POS	POS	POS	AMB	POS	POS	POS	NEG	NEG	NEG	NEG	NEG	NEG	NEG	NEG	NEG	NEG	NEG	POS	NEG	POS	NEG	NEG	POS
**CC45-MSSA**																															
2012_172_Z3449 (49/12)	POS	NEG	POS	POS	POS	POS	NEG	NEG	NEG	NEG	AMB	POS	POS	POS	NEG	NEG	NEG	NEG	NEG	NEG	NEG	NEG	NEG	NEG	NEG	POS	NEG	POS	NEG	NEG	NEG
2012_208_Z3468 (1011/12)	POS	NEG	POS	POS	POS	POS	NEG	NEG	NEG	NEG	NEG	POS	POS	POS	NEG	NEG	NEG	NEG	NEG	NEG	NEG	NEG	NEG	NEG	NEG	POS	NEG	POS	NEG	NEG	NEG
2012_235_Z3522 (2085/12)	POS	NEG	POS	POS	POS	POS	NEG	NEG	NEG	NEG	AMB	POS	POS	POS	NEG	NEG	NEG	NEG	NEG	NEG	NEG	NEG	NEG	NEG	NEG	POS	NEG	POS	NEG	NEG	NEG
2012_290_Z3577 (4921/12)	POS	NEG	POS	POS	POS	POS	NEG	NEG	NEG	NEG	NEG	POS	POS	POS	NEG	NEG	NEG	NEG	NEG	NEG	NEG	NEG	NEG	NEG	NEG	POS	NEG	POS	NEG	NEG	NEG
2012_311_Z3598 (6182/12)	POS	NEG	POS	POS	POS	POS	NEG	NEG	NEG	NEG	NEG	POS	POS	POS	NEG	NEG	NEG	NEG	NEG	NEG	NEG	NEG	NEG	NEG	NEG	POS	NEG	POS	NEG	NEG	NEG
Z3604_TT317 - (DC558C18-2959-4049-B8F3-507365	POS	NEG	POS	POS	POS	POS	NEG	NEG	NEG	NEG	NEG	POS	POS	POS	NEG	NEG	NEG	NEG	NEG	NEG	NEG	NEG	NEG	NEG	NEG	POS	NEG	POS	NEG	NEG	NEG
2012_363_Z3423 (1112-61058)	POS	NEG	POS	POS	POS	POS	NEG	NEG	NEG	NEG	NEG	POS	POS	POS	NEG	NEG	NEG	NEG	NEG	NEG	NEG	NEG	NEG	NEG	NEG	POS	NEG	POS	NEG	NEG	NEG
2013_PA19_Z4002	POS	NEG	POS	POS	POS	POS	NEG	NEG	NEG	NEG	NEG	POS	POS	POS	NEG	NEG	NEG	NEG	NEG	NEG	NEG	NEG	NEG	NEG	NEG	POS	NEG	POS	NEG	NEG	NEG
2013_PA54_Z4037	POS	NEG	POS	POS	POS	POS	NEG	NEG	NEG	NEG	AMB	POS	POS	POS	NEG	NEG	NEG	NEG	NEG	NEG	NEG	NEG	NEG	NEG	NEG	POS	NEG	POS	NEG	NEG	NEG
2013_PA82_Z4065	POS	NEG	POS	POS	POS	POS	NEG	NEG	NEG	NEG	NEG	POS	POS	POS	NEG	NEG	NEG	NEG	NEG	NEG	NEG	NEG	NEG	NEG	NEG	POS	NEG	POS	NEG	NEG	NEG
**CC97-MSSA**																															
2012_182_Z3453 (279A/12)	POS	POS	NEG	POS	POS	POS	POS	POS	POS	POS	AMB	POS	NEG	POS	NEG	NEG	NEG	NEG	NEG	NEG	NEG	NEG	NEG	NEG	NEG	POS	POS	NEG	POS	POS	NEG
2012_187_Z3504	POS	POS	NEG	POS	POS	POS	POS	POS	POS	POS	NEG	POS	NEG	POS	NEG	NEG	NEG	NEG	NEG	NEG	NEG	NEG	NEG	NEG	NEG	POS	POS	NEG	POS	POS	NEG
2012_197_Z3510 (757/12)	POS	POS	NEG	POS	POS	POS	POS	POS	POS	POS	NEG	POS	NEG	POS	NEG	NEG	NEG	NEG	NEG	NEG	NEG	NEG	NEG	NEG	NEG	POS	POS	NEG	POS	POS	POS
2012_199_Z3516 (785/12)	POS	POS	NEG	POS	POS	POS	POS	POS	POS	POS	AMB	POS	NEG	POS	NEG	NEG	NEG	NEG	NEG	NEG	NEG	NEG	NEG	NEG	NEG	POS	POS	NEG	POS	POS	POS
2013_PA15_Z3998	POS	POS	NEG	POS	POS	POS	POS	POS	POS	POS	AMB	POS	NEG	POS	NEG	NEG	NEG	NEG	NEG	NEG	NEG	NEG	NEG	NEG	NEG	POS	POS	NEG	POS	POS	POS
**CC101-MSSA**																															
2012_283_Z3570 (4725/12)	POS	POS	NEG	POS	POS	POS	POS	POS	POS	POS	POS	NEG	NEG	NEG	NEG	NEG	NEG	NEG	NEG	NEG	NEG	NEG	NEG	NEG	NEG	POS	POS	NEG	POS	POS	POS
2012_247_Z3531 (2788/12)	POS	POS	NEG	POS	POS	POS	POS	POS	POS	POS	NEG	POS	NEG	POS	NEG	NEG	NEG	NEG	NEG	NEG	NEG	NEG	NEG	NEG	NEG	POS	POS	NEG	POS	POS	POS
2012_279_Z3566 (4506/12)	POS	POS	NEG	POS	POS	POS	POS	POS	POS	POS	POS	POS	NEG	POS	NEG	NEG	NEG	NEG	NEG	NEG	NEG	NEG	NEG	NEG	NEG	POS	POS	NEG	POS	POS	POS

Strain / Isolates	leukocidin/ haemolysin toxin family protein	leukocidin/haemolysin toxin family protein		putative membrane protein	haemolysin alpha	putative membrane protein		haemolysin beta				staphylo-kinase	chemotaxis-inhibiting protein (CHIPS)	Staphyl. Comple-ment inhibitor	exfoliative toxin serotype A	exfoliative toxin serotype B	exfoliative toxin D	epidermal cell differen-tiation inhibitor	epidermal cell differen-tiation inhibitor B	epidermal cell differen-tiation inhibitor C	ACME-locus	ACME-locus	ACME-locus: ornithincarb-amoyltrans-ferase	ACME-locus: carbamat-kinase	ACME-locus: arginine/orni-thine-antiporter	aureolysin			serin- protease A	serin- protease B	serin- protease E
	lukX	lukY	lukY (ST30+ST45)	hl	hla	hlIII (consensus)	hlIII (other than RF122)	hlb_probe 1	hlb_probe 2	hlb_probe 3	un-disrupted hlb	sak	chp	scn	etA	etB	etD	edinA	edinB	edinC	ACME (total)	arcA-SCC	arcB-SCC	arcC-SCC	arcD-SCC	aur (consensus)	aur (other than MRSA252)	aur (MRSA252)	splA	splB	splE
**CC121-MSSA**																															
2012_163_Z3446 (26495/11)	POS	POS	NEG	POS	POS	POS	NEG	POS	POS	POS	AMB	POS	NEG	POS	POS	NEG	NEG	NEG	NEG	NEG	NEG	NEG	NEG	NEG	NEG	POS	POS	NEG	POS	POS	NEG
2012_204_Z3464 (938/12)	POS	POS	NEG	POS	POS	POS	NEG	POS	POS	POS	POS	POS	NEG	POS	NEG	POS	NEG	NEG	NEG	POS	NEG	NEG	NEG	NEG	NEG	POS	POS	NEG	POS	POS	NEG
2012_225_Z3482 (1563/12)	POS	POS	NEG	POS	POS	POS	NEG	POS	POS	POS	POS	POS	NEG	POS	POS	POS	NEG	NEG	NEG	POS	NEG	NEG	NEG	NEG	NEG	POS	POS	NEG	POS	POS	NEG
2012_228_Z3485 (1700B/12)	POS	POS	NEG	POS	POS	POS	NEG	POS	POS	POS	POS	POS	NEG	POS	POS	NEG	NEG	NEG	NEG	NEG	NEG	NEG	NEG	NEG	NEG	POS	POS	NEG	POS	POS	NEG
2012_229_Z3486 (1737/12)	POS	POS	NEG	POS	POS	POS	NEG	POS	POS	POS	POS	POS	NEG	POS	POS	NEG	NEG	NEG	NEG	NEG	NEG	NEG	NEG	NEG	NEG	POS	POS	NEG	POS	POS	NEG
2012_302_Z3589 (5795/12)	POS	POS	NEG	POS	POS	POS	NEG	POS	POS	POS	NEG	POS	NEG	POS	NEG	NEG	NEG	NEG	NEG	NEG	NEG	NEG	NEG	NEG	NEG	POS	POS	NEG	POS	POS	NEG
2012_359_Z3419	POS	POS	NEG	POS	POS	POS	NEG	POS	POS	POS	AMB	POS	NEG	POS	POS	NEG	NEG	NEG	NEG	NEG	NEG	NEG	NEG	NEG	NEG	POS	POS	NEG	POS	POS	NEG
2012_362_Z3422 (1201-996)	POS	POS	NEG	POS	POS	POS	NEG	POS	POS	POS	AMB	POS	NEG	POS	NEG	POS	NEG	NEG	NEG	POS	NEG	NEG	NEG	NEG	NEG	POS	POS	NEG	POS	POS	NEG
2012_367_Z3427 (1204-16413)	POS	POS	NEG	POS	POS	POS	NEG	POS	POS	POS	AMB	POS	NEG	POS	NEG	NEG	NEG	NEG	NEG	NEG	NEG	NEG	NEG	NEG	NEG	POS	POS	NEG	POS	POS	NEG
2013_PA09_Z3992	POS	POS	NEG	POS	POS	POS	NEG	POS	POS	POS	NEG	POS	NEG	POS	POS	POS	NEG	NEG	NEG	POS	NEG	NEG	NEG	NEG	NEG	POS	POS	NEG	POS	POS	NEG
2013_PA59_Z4042	POS	POS	NEG	POS	POS	POS	NEG	POS	POS	POS	NEG	POS	NEG	POS	NEG	POS	NEG	NEG	NEG	AMB	NEG	NEG	NEG	NEG	NEG	POS	POS	NEG	POS	POS	NEG
2013_PA60_Z4043	POS	POS	NEG	POS	POS	POS	NEG	POS	POS	POS	NEG	POS	NEG	POS	NEG	NEG	NEG	NEG	NEG	NEG	NEG	NEG	NEG	NEG	NEG	POS	POS	NEG	POS	POS	NEG
**CC152-MSSA [PVL+]**																															
2012_190_Z3507 (549/12)	POS	POS	NEG	NEG	POS	POS	NEG	AMB	AMB	POS	POS	POS	NEG	POS	NEG	NEG	NEG	NEG	POS	NEG	NEG	NEG	NEG	NEG	NEG	POS	POS	NEG	NEG	NEG	NEG
2012_201_Z3461 (823/12)	POS	POS	NEG	NEG	POS	POS	NEG	POS	AMB	POS	POS	POS	NEG	POS	NEG	NEG	NEG	NEG	POS	NEG	NEG	NEG	NEG	NEG	NEG	POS	POS	NEG	NEG	NEG	NEG
2012_209_Z3469 (1116/12)	POS	POS	NEG	NEG	POS	POS	NEG	NEG	NEG	POS	POS	POS	NEG	POS	NEG	NEG	NEG	NEG	POS	NEG	NEG	NEG	NEG	NEG	NEG	POS	POS	NEG	NEG	NEG	NEG
2012_211_Z3517 (1238/12)	NEG	POS	NEG	NEG	POS	POS	NEG	AMB	NEG	POS	POS	POS	NEG	POS	NEG	NEG	NEG	NEG	POS	NEG	NEG	NEG	NEG	NEG	NEG	POS	POS	NEG	NEG	NEG	NEG
2012_213_Z3472 (1320/12)	AMB	POS	NEG	NEG	POS	POS	NEG	AMB	NEG	POS	POS	POS	NEG	POS	NEG	NEG	NEG	NEG	POS	NEG	NEG	NEG	NEG	NEG	NEG	POS	POS	NEG	NEG	NEG	NEG
2012_221_Z3478	POS	POS	NEG	NEG	POS	POS	NEG	AMB	NEG	POS	POS	POS	NEG	POS	NEG	NEG	NEG	NEG	POS	NEG	NEG	NEG	NEG	NEG	NEG	POS	POS	NEG	NEG	NEG	NEG
2012_234_Z3521 (1981/12)	AMB	POS	NEG	NEG	POS	POS	NEG	POS	POS	POS	POS	POS	NEG	POS	NEG	NEG	NEG	NEG	POS	NEG	NEG	NEG	NEG	NEG	NEG	POS	POS	NEG	NEG	NEG	NEG
2012_238_Z3524 (2199/12)	AMB	POS	NEG	NEG	POS	POS	NEG	AMB	AMB	POS	POS	POS	NEG	POS	NEG	NEG	NEG	NEG	POS	NEG	NEG	NEG	NEG	NEG	NEG	POS	POS	NEG	NEG	NEG	NEG
2012_260_Z3547 (3290/12)	POS	POS	NEG	NEG	POS	POS	NEG	POS	POS	POS	POS	POS	NEG	POS	NEG	NEG	NEG	NEG	NEG	NEG	NEG	NEG	NEG	NEG	NEG	POS	POS	NEG	NEG	NEG	NEG
2012_272_Z3559 (3802/12)	POS	POS	NEG	NEG	POS	POS	NEG	POS	POS	POS	POS	POS	NEG	POS	NEG	NEG	NEG	NEG	NEG	NEG	NEG	NEG	NEG	NEG	NEG	POS	POS	NEG	NEG	NEG	NEG
2012_289_Z3576 (4912/12)	AMB	POS	NEG	NEG	POS	POS	NEG	AMB	NEG	POS	POS	POS	NEG	POS	NEG	NEG	NEG	NEG	POS	NEG	NEG	NEG	NEG	NEG	NEG	POS	POS	NEG	NEG	NEG	NEG
2012_309_Z3596	AMB	POS	NEG	NEG	POS	POS	NEG	AMB	AMB	POS	POS	POS	NEG	POS	NEG	NEG	NEG	NEG	POS	NEG	NEG	NEG	NEG	NEG	NEG	POS	POS	NEG	NEG	NEG	NEG
2012_322_Z3609 (7178/12)	NEG	POS	NEG	NEG	POS	POS	NEG	NEG	NEG	POS	POS	POS	NEG	POS	NEG	NEG	NEG	NEG	POS	NEG	NEG	NEG	NEG	NEG	NEG	POS	POS	NEG	NEG	NEG	NEG
2012_325_Z3612 (7254/12)	POS	POS	NEG	NEG	POS	POS	NEG	POS	AMB	POS	POS	POS	NEG	POS	NEG	NEG	NEG	NEG	NEG	NEG	NEG	NEG	NEG	NEG	NEG	POS	POS	NEG	NEG	NEG	NEG
2012_328_Z3615 (7456/12)	POS	POS	NEG	NEG	POS	POS	NEG	POS	POS	POS	POS	POS	NEG	POS	NEG	NEG	NEG	NEG	POS	NEG	NEG	NEG	NEG	NEG	NEG	POS	POS	NEG	NEG	NEG	NEG
2012_334_Z3621 (8267/12)	POS	POS	NEG	NEG	POS	POS	NEG	POS	AMB	POS	POS	POS	NEG	POS	NEG	NEG	NEG	NEG	POS	NEG	NEG	NEG	NEG	NEG	NEG	POS	POS	NEG	NEG	NEG	NEG
2012_360_Z3420 (1201-4047)	AMB	POS	NEG	NEG	POS	POS	NEG	POS	POS	POS	POS	POS	POS	POS	NEG	NEG	NEG	NEG	POS	NEG	NEG	NEG	NEG	NEG	NEG	POS	POS	NEG	NEG	NEG	NEG
2012_382_Z3442 (4086)	AMB	POS	NEG	NEG	POS	POS	NEG	NEG	AMB	POS	POS	POS	NEG	POS	NEG	NEG	NEG	NEG	POS	NEG	NEG	NEG	NEG	NEG	NEG	POS	POS	NEG	NEG	NEG	NEG
2013_PA02_Z3985	NEG	POS	NEG	NEG	POS	POS	NEG	AMB	AMB	POS	POS	POS	NEG	POS	NEG	NEG	NEG	NEG	POS	NEG	NEG	NEG	NEG	NEG	NEG	POS	POS	NEG	NEG	NEG	NEG
2013_PA03_Z3986	NEG	POS	NEG	NEG	POS	POS	NEG	AMB	AMB	POS	POS	POS	NEG	POS	NEG	NEG	NEG	NEG	POS	NEG	NEG	NEG	NEG	NEG	NEG	POS	POS	NEG	NEG	NEG	NEG
2013_PA07_Z3990	AMB	POS	NEG	NEG	POS	POS	NEG	POS	POS	POS	POS	POS	NEG	POS	NEG	NEG	NEG	NEG	POS	NEG	NEG	NEG	NEG	NEG	NEG	POS	POS	NEG	NEG	NEG	NEG
2013_PA34_Z4017	NEG	POS	NEG	NEG	POS	POS	NEG	NEG	NEG	POS	POS	POS	NEG	POS	NEG	NEG	NEG	NEG	POS	NEG	NEG	NEG	NEG	NEG	NEG	POS	POS	NEG	NEG	NEG	NEG
2013_PA40_Z4023	NEG	POS	NEG	NEG	POS	POS	NEG	NEG	NEG	POS	POS	POS	NEG	POS	NEG	NEG	NEG	NEG	POS	NEG	NEG	NEG	NEG	NEG	NEG	POS	POS	NEG	NEG	NEG	NEG
2013_PA48_Z4031	AMB	POS	NEG	NEG	POS	POS	NEG	AMB	AMB	POS	POS	POS	NEG	POS	NEG	NEG	NEG	NEG	POS	NEG	NEG	NEG	NEG	NEG	NEG	POS	POS	NEG	NEG	NEG	NEG
2013_PA49_Z4032	NEG	POS	NEG	NEG	POS	POS	NEG	NEG	NEG	POS	POS	POS	NEG	POS	NEG	NEG	NEG	NEG	POS	NEG	NEG	NEG	NEG	NEG	NEG	POS	POS	NEG	NEG	NEG	NEG
2013_PA51_Z4034	AMB	POS	NEG	NEG	POS	POS	NEG	AMB	AMB	POS	POS	POS	NEG	POS	NEG	NEG	NEG	NEG	POS	NEG	NEG	NEG	NEG	NEG	NEG	POS	POS	NEG	NEG	NEG	NEG
2013_PA70_Z4053	NEG	POS	NEG	NEG	POS	POS	NEG	NEG	NEG	POS	POS	POS	NEG	POS	NEG	NEG	NEG	NEG	POS	NEG	NEG	NEG	NEG	NEG	NEG	POS	POS	NEG	NEG	NEG	NEG
2013_PA84_Z4067	NEG	POS	NEG	NEG	POS	POS	NEG	AMB	AMB	POS	POS	POS	NEG	POS	NEG	NEG	NEG	NEG	POS	NEG	NEG	NEG	NEG	NEG	NEG	POS	POS	NEG	NEG	NEG	NEG
**CC188-MSSA**																															
2012_206_Z3466 (968/12)	POS	POS	NEG	POS	POS	POS	POS	POS	POS	POS	POS	POS	NEG	POS	NEG	NEG	NEG	NEG	NEG	NEG	NEG	NEG	NEG	NEG	NEG	POS	POS	NEG	POS	POS	POS
2012_251_Z3534 (3026/12)	POS	POS	NEG	POS	POS	POS	POS	POS	POS	POS	AMB	POS	POS	POS	NEG	NEG	NEG	NEG	NEG	NEG	NEG	NEG	NEG	NEG	NEG	POS	POS	NEG	POS	POS	POS
2012_320_Z3607 (7144/12)	POS	POS	NEG	POS	POS	POS	POS	POS	POS	POS	AMB	POS	NEG	POS	NEG	NEG	NEG	NEG	NEG	NEG	NEG	NEG	NEG	NEG	NEG	POS	POS	NEG	POS	POS	POS
2012_321_Z3608 (7147/12)	POS	POS	NEG	POS	POS	POS	POS	POS	POS	POS	AMB	POS	NEG	POS	NEG	NEG	NEG	NEG	NEG	NEG	NEG	NEG	NEG	NEG	NEG	POS	POS	NEG	POS	POS	POS
2012_358_Z3418 (1202-6332)	POS	POS	NEG	POS	POS	POS	POS	POS	POS	POS	POS	POS	NEG	POS	NEG	NEG	NEG	NEG	NEG	NEG	NEG	NEG	NEG	NEG	NEG	POS	POS	NEG	POS	POS	POS
**CC398-MSSA**																															
2013_PA11_Z3994	POS	POS	NEG	POS	POS	POS	POS	POS	POS	NEG	NEG	NEG	POS	POS	NEG	NEG	NEG	NEG	NEG	NEG	NEG	NEG	NEG	NEG	NEG	POS	NEG	POS	NEG	NEG	NEG
2013_PA57_Z4040	POS	POS	NEG	POS	POS	POS	POS	POS	POS	NEG	NEG	NEG	POS	POS	NEG	NEG	NEG	NEG	NEG	NEG	NEG	NEG	NEG	NEG	NEG	POS	NEG	POS	NEG	NEG	NEG
**ST1852-MSSA**																															
2012_277_Z3564 (4050/12)	NEG	NEG	POS	POS	POS	POS	POS	POS	POS	NEG	NEG	POS	NEG	POS	NEG	NEG	NEG	NEG	NEG	NEG	NEG	NEG	NEG	NEG	NEG	POS	POS	NEG	POS	POS	POS
**CC1223/1594-MSSA**																															
2012_337_Z3624 (8070B/12)	NEG	NEG	NEG	NEG	AMB	NEG	AMB	NEG	AMB	NEG	NEG	NEG	NEG	POS	NEG	NEG	NEG	NEG	NEG	NEG	NEG	NEG	NEG	NEG	NEG	POS	NEG	NEG	NEG	NEG	NEG
**MSSA related to ST2250/2277**																															
2012_203_Z3463 (829/12)	NEG	NEG	NEG	POS	POS	POS	POS	AMB	POS	NEG	NEG	POS	NEG	POS	NEG	NEG	NEG	NEG	NEG	NEG	NEG	NEG	NEG	NEG	NEG	POS	POS	POS	NEG	NEG	NEG
2012_269_Z3556 (3769A/12)	NEG	NEG	NEG	NEG	NEG	AMB	NEG	NEG	AMB	NEG	NEG	POS	NEG	POS	NEG	NEG	NEG	NEG	NEG	NEG	NEG	NEG	NEG	NEG	NEG	NEG	POS	POS	NEG	NEG	NEG
2012_292_Z3579	NEG	NEG	NEG	NEG	AMB	NEG	NEG	NEG	AMB	NEG	NEG	POS	NEG	POS	NEG	NEG	NEG	NEG	NEG	NEG	NEG	NEG	NEG	NEG	NEG	AMB	POS	POS	NEG	NEG	NEG
2012_299_Z3586 (5487/12)	NEG	NEG	NEG	NEG	NEG	NEG	NEG	NEG	AMB	NEG	NEG	POS	NEG	POS	NEG	NEG	NEG	NEG	NEG	NEG	NEG	NEG	NEG	NEG	NEG	AMB	POS	POS	NEG	NEG	NEG
2012_301_Z3588 (5717/12)	NEG	NEG	NEG	NEG	NEG	NEG	NEG	NEG	NEG	NEG	NEG	POS	NEG	POS	NEG	NEG	NEG	NEG	NEG	NEG	NEG	NEG	NEG	NEG	NEG	AMB	POS	POS	NEG	NEG	NEG
**ST2596dlv-MSSA**																															
2012_310_Z3597 (6034/12)	NEG	NEG	NEG	NEG	NEG	AMB	NEG	NEG	AMB	NEG	NEG	NEG	NEG	POS	NEG	NEG	NEG	NEG	NEG	NEG	NEG	NEG	NEG	NEG	NEG	AMB	AMB	AMB	NEG	NEG	NEG

Strain / Isolates	glutamylendopeptidase	staphopain B, protease	staphopain A (staphylopain A), protease		staphyl. exotoxin-like protein	staphylococcal superantigen-like protein 1												staphylococcal superantigen-like protein 2		staphylococcal superantigen-like protein 3			staphylococcal superantigen-like protein 4		staphylococcal superantigen-like protein 5				staphylococcal superantigen-like protein 6		staphylococcal superantigen-like protein 7			staphylococcal superantigen-like protein 8	
	sspA	sspB	sspP (consensus)	sspP (other than ST93)	setC	ssl01/set6_probe1_11	ssl01/set6_probe2_11	ssl01/set6_probe1_12	ssl01/set6_probe2_12	ssl01/set6_probe4_11	ssl01/set6_probeRF122	ssl01/set6 (COL)	ssl01/set6 (Mu50+N315)	ssl01/set6 (MW2+MSSA476)	ssl01/set6 (MRSA252)	ssl01/set6 (RF122)	ssl01/set6 (other alleles)	ssl02/set7	ssl02/set7 (MRSA252)	ssl03/set8_probe 1	ssl03/set8_probe 2	ssl03/set8 (MRSA252, SAR0424)	ssl04/set9	ssl04/set9 (MRSA252, SAR0425)	ssl05/set3_probe 1	ssl05/set3 (RF122, probe-611)	ssl05/set3_probe 2 (612)	ssl05/set3 (MRSA252)	ssl06/set21	ssl06 (NCTC8325+MW2)	ssl07/set1	ssl07/set1 (MRSA252)	ssl07/set1 (AF188836)	ssl08/set12_probe 1	ssl08/set12_probe 2
**CC1-MSSA**																																			
2012_257_Z3544 (3216/12)	POS	POS	POS	POS	POS	NEG	POS	AMB	POS	NEG	NEG	NEG	NEG	POS	NEG	NEG	NEG	POS	AMB	POS	POS	NEG	POS	AMB	POS	AMB	POS	NEG	POS	POS	AMB	POS	AMB	POS	POS
2012_281_Z3568 (4696/12)	POS	POS	POS	POS	POS	NEG	POS	NEG	POS	NEG	NEG	NEG	NEG	POS	NEG	NEG	NEG	POS	AMB	POS	POS	NEG	POS	NEG	POS	POS	AMB	NEG	POS	POS	AMB	POS	AMB	POS	POS
2012_324_Z3611 (7180/12)	POS	POS	POS	POS	POS	NEG	POS	NEG	POS	NEG	NEG	NEG	NEG	POS	NEG	NEG	NEG	POS	AMB	POS	POS	NEG	POS	AMB	POS	AMB	POS	NEG	POS	POS	AMB	POS	AMB	POS	POS
2012_327_Z3614 (7347/12)	POS	POS	POS	POS	POS	NEG	POS	AMB	POS	NEG	NEG	NEG	NEG	POS	NEG	NEG	NEG	POS	AMB	POS	POS	NEG	POS	AMB	POS	AMB	POS	NEG	NEG	NEG	AMB	POS	AMB	POS	POS
2012_351_Z3638 (8273B/12)	POS	POS	POS	POS	POS	NEG	POS	NEG	POS	NEG	NEG	NEG	NEG	POS	NEG	NEG	NEG	POS	AMB	POS	POS	NEG	POS	NEG	POS	AMB	POS	NEG	NEG	NEG	AMB	POS	AMB	POS	POS
**CC5-MSSA**																																			
2012_188_Z3505 (503/12)	POS	POS	POS	POS	POS	POS	NEG	POS	NEG	POS	NEG	POS	AMB	NEG	NEG	NEG	NEG	AMB	POS	POS	POS	NEG	POS	NEG	POS	AMB	POS	NEG	NEG	NEG	AMB	POS	AMB	POS	POS
2012_219_Z3518 (1437/12)	POS	POS	POS	POS	POS	POS	AMB	POS	AMB	POS	NEG	NEG	POS	NEG	NEG	NEG	NEG	AMB	POS	POS	POS	NEG	AMB	NEG	POS	POS	AMB	NEG	NEG	AMB	AMB	POS	AMB	POS	POS
2012_329_Z3616 (7577/12)	POS	POS	POS	POS	POS	POS	NEG	POS	AMB	POS	NEG	NEG	POS	NEG	NEG	NEG	NEG	AMB	POS	POS	POS	NEG	POS	AMB	POS	AMB	POS	NEG	NEG	AMB	AMB	POS	AMB	POS	POS
2012_377_Z3437 (6242)	POS	POS	POS	POS	POS	POS	NEG	POS	NEG	POS	NEG	POS	AMB	NEG	NEG	NEG	NEG	AMB	POS	POS	POS	NEG	POS	AMB	POS	AMB	POS	NEG	NEG	AMB	AMB	POS	AMB	POS	POS
2013_PA04_Z3987	POS	POS	POS	POS	POS	POS	NEG	POS	NEG	POS	NEG	POS	AMB	NEG	NEG	NEG	NEG	POS	AMB	POS	POS	NEG	POS	AMB	POS	AMB	POS	NEG	NEG	NEG	POS	AMB	AMB	POS	POS
2013_PA32_Z4015	POS	POS	POS	POS	POS	POS	NEG	POS	NEG	POS	NEG	POS	AMB	NEG	NEG	NEG	NEG	POS	AMB	POS	POS	NEG	POS	AMB	POS	AMB	POS	NEG	NEG	AMB	AMB	POS	AMB	POS	POS
2013_PA41_Z4024	POS	POS	POS	POS	POS	POS	NEG	POS	NEG	POS	NEG	NEG	POS	NEG	NEG	NEG	NEG	POS	AMB	POS	POS	NEG	POS	NEG	POS	AMB	POS	NEG	NEG	NEG	POS	AMB	AMB	POS	POS
2013_PA44_Z4027	POS	POS	POS	POS	POS	POS	NEG	POS	NEG	POS	NEG	NEG	POS	NEG	NEG	NEG	NEG	POS	AMB	POS	POS	NEG	POS	NEG	POS	AMB	POS	NEG	NEG	NEG	POS	AMB	AMB	POS	POS
**CC5-MSSA [PVL+]**																																			
2012_175_Z3497 (112/12)	POS	POS	POS	POS	POS	POS	NEG	POS	NEG	POS	NEG	NEG	POS	NEG	NEG	NEG	NEG	AMB	POS	POS	POS	NEG	POS	NEG	POS	POS	AMB	NEG	NEG	NEG	AMB	POS	AMB	POS	POS
2012_193_Z3458 (617/12)	POS	POS	POS	POS	POS	POS	NEG	POS	AMB	POS	NEG	NEG	POS	NEG	NEG	NEG	NEG	AMB	POS	POS	POS	NEG	POS	AMB	POS	AMB	POS	NEG	NEG	NEG	AMB	POS	AMB	POS	POS
2012_215_Z3474 (1341/12)	POS	POS	POS	POS	POS	POS	NEG	POS	NEG	POS	NEG	POS	AMB	NEG	NEG	NEG	NEG	AMB	POS	POS	POS	NEG	POS	NEG	POS	AMB	POS	NEG	NEG	NEG	AMB	POS	AMB	POS	POS
2012_216_Z3475 (1370/12)	POS	POS	POS	POS	POS	POS	NEG	POS	NEG	POS	NEG	POS	AMB	NEG	NEG	NEG	NEG	POS	AMB	POS	POS	NEG	POS	NEG	POS	AMB	POS	NEG	NEG	NEG	POS	AMB	AMB	POS	POS
2012_297_Z3584 (5140/12)	POS	POS	POS	POS	POS	POS	NEG	POS	NEG	POS	NEG	POS	AMB	NEG	NEG	NEG	NEG	POS	AMB	POS	POS	NEG	POS	NEG	POS	AMB	POS	NEG	NEG	NEG	AMB	POS	AMB	POS	POS
2012_331_Z3618 (7644/12)	POS	POS	POS	POS	POS	POS	AMB	POS	AMB	POS	NEG	NEG	POS	NEG	NEG	NEG	NEG	AMB	POS	POS	POS	NEG	POS	AMB	POS	POS	AMB	NEG	NEG	NEG	AMB	POS	AMB	POS	POS
2012_379_Z3439 (4126)	POS	POS	POS	POS	POS	POS	NEG	POS	NEG	POS	NEG	POS	AMB	NEG	NEG	NEG	NEG	AMB	POS	POS	POS	NEG	POS	NEG	POS	AMB	POS	NEG	NEG	NEG	AMB	POS	AMB	POS	POS
2012_381_Z3441	POS	POS	POS	POS	POS	POS	AMB	POS	POS	POS	NEG	POS	AMB	NEG	NEG	NEG	NEG	POS	AMB	POS	POS	NEG	POS	AMB	POS	POS	AMB	NEG	NEG	POS	AMB	POS	AMB	POS	POS
2013_PA39_Z4022	POS	POS	POS	POS	POS	POS	NEG	POS	NEG	POS	NEG	NEG	POS	NEG	NEG	NEG	NEG	POS	AMB	POS	POS	NEG	POS	NEG	POS	AMB	POS	NEG	NEG	NEG	POS	AMB	AMB	POS	POS
2013_PA47_Z4030	POS	POS	POS	POS	POS	POS	NEG	POS	NEG	POS	NEG	NEG	POS	NEG	NEG	NEG	NEG	POS	AMB	POS	POS	NEG	POS	NEG	POS	AMB	POS	NEG	NEG	NEG	POS	AMB	NEG	POS	POS
2013_PA61_I_Z4044	POS	POS	POS	POS	POS	POS	NEG	POS	NEG	POS	NEG	POS	AMB	NEG	NEG	NEG	NEG	POS	AMB	POS	POS	NEG	POS	AMB	POS	AMB	POS	NEG	NEG	AMB	AMB	POS	AMB	POS	POS
2013_PA62_I_Z4045	POS	POS	POS	POS	POS	POS	NEG	POS	NEG	POS	NEG	NEG	POS	NEG	NEG	NEG	NEG	POS	AMB	POS	POS	NEG	POS	NEG	POS	AMB	POS	NEG	NEG	NEG	POS	AMB	AMB	POS	POS
2013_PA74_Z4057	POS	POS	POS	POS	POS	POS	NEG	POS	NEG	POS	NEG	NEG	POS	NEG	NEG	NEG	NEG	POS	AMB	POS	POS	NEG	POS	NEG	POS	AMB	POS	NEG	NEG	NEG	POS	AMB	AMB	POS	POS
2013_PA75_Z4058	POS	POS	POS	POS	POS	POS	NEG	POS	NEG	POS	NEG	NEG	POS	NEG	NEG	NEG	NEG	POS	AMB	POS	POS	NEG	POS	NEG	POS	AMB	POS	NEG	NEG	NEG	POS	AMB	AMB	POS	POS
**CC5-MRSA-V [ACME+]**																																			
2012_373_Z3433 (1204-17312)	POS	POS	POS	POS	POS	POS	NEG	POS	AMB	POS	NEG	NEG	POS	NEG	NEG	NEG	NEG	AMB	POS	POS	POS	NEG	POS	AMB	POS	AMB	POS	NEG	NEG	AMB	AMB	POS	AMB	POS	POS
**CC6-MSSA**																																			
2012_173_Z3450 (98A/12)	POS	POS	POS	POS	POS	NEG	NEG	POS	NEG	POS	POS	NEG	NEG	NEG	NEG	NEG	POS	AMB	POS	POS	POS	NEG	POS	AMB	POS	AMB	POS	POS	NEG	AMB	AMB	AMB	POS	POS	POS
2012_176_Z3498	POS	POS	POS	POS	POS	NEG	NEG	POS	NEG	POS	POS	NEG	NEG	NEG	NEG	NEG	POS	AMB	POS	POS	POS	NEG	POS	NEG	POS	AMB	POS	POS	NEG	NEG	POS	NEG	AMB	POS	POS
2012_181_Z3452	POS	POS	POS	POS	POS	NEG	NEG	POS	NEG	POS	POS	NEG	NEG	NEG	NEG	NEG	POS	AMB	POS	POS	POS	NEG	POS	NEG	POS	AMB	POS	POS	NEG	NEG	POS	NEG	AMB	POS	POS
2012_259_Z3546 (3279/12)	POS	POS	POS	POS	POS	NEG	NEG	POS	NEG	POS	POS	NEG	NEG	NEG	NEG	NEG	POS	POS	AMB	POS	POS	NEG	POS	AMB	POS	AMB	POS	POS	NEG	NEG	POS	NEG	NEG	POS	POS
2012_264_Z3551 (3407B/12)	POS	POS	POS	POS	POS	NEG	NEG	POS	NEG	POS	POS	NEG	NEG	NEG	NEG	NEG	POS	POS	AMB	POS	POS	NEG	POS	AMB	POS	AMB	POS	AMB	NEG	NEG	POS	NEG	AMB	POS	POS
2012_273_Z3560 (3844/12)	POS	POS	POS	POS	POS	NEG	NEG	POS	NEG	POS	POS	NEG	NEG	NEG	NEG	NEG	POS	POS	AMB	POS	POS	NEG	POS	AMB	POS	AMB	POS	POS	NEG	NEG	POS	NEG	AMB	POS	POS
2012_312_Z3599 (6815/12)	POS	POS	POS	POS	POS	NEG	NEG	POS	NEG	POS	POS	NEG	NEG	NEG	NEG	NEG	POS	POS	AMB	POS	POS	NEG	POS	NEG	POS	AMB	POS	NEG	NEG	NEG	POS	NEG	NEG	POS	POS
2012_338_Z3625	POS	POS	POS	POS	POS	NEG	NEG	POS	NEG	POS	POS	NEG	NEG	NEG	NEG	NEG	POS	POS	AMB	POS	POS	NEG	POS	NEG	POS	AMB	POS	AMB	NEG	NEG	POS	NEG	AMB	POS	POS
2012_352_Z3639	POS	POS	POS	POS	POS	NEG	NEG	POS	NEG	POS	POS	NEG	NEG	NEG	NEG	NEG	POS	POS	AMB	POS	POS	NEG	POS	NEG	POS	AMB	POS	AMB	NEG	NEG	POS	NEG	AMB	POS	POS
2012_368_Z3428	POS	POS	POS	POS	POS	NEG	NEG	AMB	NEG	POS	POS	NEG	NEG	NEG	NEG	NEG	POS	POS	AMB	POS	POS	NEG	POS	NEG	POS	AMB	POS	NEG	NEG	AMB	POS	NEG	NEG	POS	POS
2013_PA12_Z3995	POS	POS	POS	POS	POS	NEG	NEG	POS	NEG	POS	POS	NEG	NEG	NEG	NEG	NEG	POS	POS	AMB	POS	POS	NEG	POS	NEG	POS	AMB	POS	AMB	NEG	NEG	POS	NEG	AMB	POS	POS
2013_PA28_Z4011	POS	POS	POS	POS	POS	NEG	NEG	NEG	NEG	POS	POS	NEG	NEG	NEG	NEG	NEG	POS	POS	AMB	POS	POS	NEG	POS	NEG	POS	AMB	POS	NEG	NEG	NEG	POS	NEG	NEG	POS	POS
**CC6-MSSA [PVL+]**																																			
2012_375_Z3435	POS	POS	POS	POS	POS	NEG	NEG	POS	NEG	POS	POS	NEG	NEG	NEG	NEG	NEG	POS	AMB	POS	POS	POS	NEG	POS	NEG	POS	AMB	POS	POS	NEG	NEG	POS	NEG	NEG	POS	POS
2013_PA77_Z4060	POS	POS	POS	POS	POS	NEG	NEG	POS	NEG	POS	POS	NEG	NEG	NEG	NEG	NEG	POS	POS	AMB	POS	POS	NEG	POS	NEG	POS	AMB	POS	NEG	NEG	AMB	POS	NEG	AMB	POS	POS
**CC7-MSSA**																																			
2012_248_Z3532 (2864/12)	POS	POS	POS	POS	POS	POS	NEG	AMB	POS	NEG	NEG	NEG	NEG	NEG	NEG	NEG	POS	POS	AMB	POS	POS	NEG	POS	NEG	POS	NEG	POS	NEG	POS	POS	AMB	POS	AMB	POS	POS
2012_265_Z3552 (3518/12)	POS	POS	POS	POS	POS	POS	NEG	AMB	POS	POS	AMB	POS	AMB	NEG	NEG	NEG	NEG	POS	AMB	POS	POS	NEG	POS	AMB	POS	AMB	POS	NEG	POS	POS	AMB	POS	AMB	POS	POS
2013_PA53_Z4036	POS	POS	POS	POS	POS	POS	NEG	NEG	POS	NEG	NEG	NEG	NEG	NEG	NEG	NEG	POS	POS	AMB	POS	POS	NEG	POS	AMB	POS	NEG	POS	NEG	POS	POS	AMB	POS	AMB	POS	POS
2013_PA83_Z4066	POS	POS	POS	POS	POS	POS	NEG	NEG	POS	NEG	NEG	NEG	NEG	NEG	NEG	NEG	POS	POS	AMB	POS	POS	NEG	POS	AMB	POS	NEG	POS	NEG	POS	POS	AMB	POS	AMB	POS	POS
**CC8-MSSA**																																			
2012_243_Z3528 (2587/12)	POS	POS	POS	POS	POS	POS	NEG	POS	NEG	POS	NEG	POS	AMB	NEG	NEG	NEG	NEG	POS	AMB	POS	POS	NEG	POS	NEG	POS	POS	AMB	NEG	POS	POS	AMB	POS	AMB	POS	POS
2012_344_Z3631 (8605/12)	POS	POS	POS	POS	POS	POS	NEG	POS	NEG	POS	NEG	POS	AMB	NEG	NEG	NEG	NEG	POS	AMB	POS	POS	NEG	POS	NEG	POS	POS	AMB	NEG	POS	POS	AMB	POS	AMB	POS	POS
2012_346_Z3633 (8074/12)	POS	POS	POS	POS	POS	POS	NEG	POS	NEG	POS	NEG	POS	AMB	NEG	NEG	NEG	NEG	POS	AMB	POS	POS	NEG	POS	NEG	POS	POS	AMB	NEG	POS	POS	AMB	POS	AMB	POS	POS
2012_255_Z3538 (3205B/12)	POS	POS	POS	POS	POS	POS	NEG	POS	NEG	POS	NEG	POS	AMB	NEG	NEG	NEG	NEG	POS	AMB	POS	POS	NEG	POS	NEG	POS	POS	AMB	NEG	NEG	POS	AMB	POS	AMB	POS	POS
2012_280_Z3567 (4601/12)	POS	POS	POS	POS	POS	POS	NEG	POS	NEG	POS	NEG	POS	AMB	NEG	NEG	NEG	NEG	POS	AMB	POS	POS	NEG	POS	NEG	POS	POS	AMB	NEG	POS	POS	AMB	POS	AMB	POS	POS
Trinidad&Tobago_2012_267 (3642/12)	POS	POS	POS	POS	POS	POS	NEG	POS	NEG	POS	NEG	POS	AMB	NEG	NEG	NEG	NEG	POS	NEG	POS	POS	NEG	POS	NEG	POS	AMB	POS	NEG	NEG	NEG	POS	AMB	NEG	POS	POS
2012_365_Z3425 (1204-15838)	POS	POS	POS	POS	POS	POS	NEG	POS	NEG	POS	NEG	NEG	POS	NEG	NEG	NEG	NEG	AMB	POS	POS	POS	NEG	POS	NEG	POS	POS	AMB	NEG	POS	POS	AMB	POS	AMB	POS	POS
2012_296_Z3583 (5118/12)	POS	POS	POS	POS	POS	POS	NEG	POS	NEG	POS	NEG	POS	AMB	NEG	NEG	NEG	NEG	POS	AMB	POS	POS	NEG	POS	NEG	AMB	AMB	POS	NEG	POS	POS	AMB	POS	AMB	POS	POS
2012_318_Z3605 (6960/12)	POS	POS	POS	POS	POS	POS	NEG	POS	NEG	POS	NEG	POS	AMB	NEG	NEG	NEG	NEG	POS	AMB	POS	AMB	NEG	POS	NEG	POS	POS	AMB	NEG	POS	POS	AMB	POS	AMB	POS	POS
2012_306_Z3593 (5861/12)	POS	POS	POS	POS	POS	POS	NEG	POS	NEG	POS	NEG	POS	AMB	NEG	NEG	NEG	NEG	POS	AMB	POS	POS	NEG	POS	AMB	POS	POS	AMB	NEG	POS	POS	AMB	POS	AMB	POS	POS
2013_PA37_Z4020	POS	POS	POS	POS	POS	POS	NEG	POS	NEG	POS	NEG	POS	AMB	NEG	NEG	NEG	NEG	POS	AMB	POS	POS	NEG	POS	NEG	POS	AMB	POS	NEG	NEG	POS	AMB	POS	AMB	POS	POS
2013_PA38_Z4021	POS	POS	POS	POS	POS	POS	NEG	NEG	NEG	POS	NEG	POS	AMB	NEG	NEG	NEG	NEG	POS	NEG	POS	POS	NEG	POS	NEG	POS	NEG	POS	NEG	POS	POS	POS	AMB	NEG	POS	POS
2013_PA52_Z4035	POS	POS	POS	POS	POS	POS	NEG	NEG	NEG	POS	NEG	POS	AMB	NEG	NEG	NEG	NEG	POS	NEG	POS	POS	NEG	POS	NEG	POS	NEG	POS	NEG	POS	POS	POS	AMB	NEG	POS	POS
**CC8-MSSA [PVL+]**																																			
2012_156_Z3489 (25917/11)	POS	POS	POS	POS	POS	POS	NEG	POS	NEG	POS	NEG	NEG	POS	NEG	NEG	NEG	NEG	AMB	POS	POS	POS	NEG	POS	NEG	POS	AMB	POS	NEG	POS	POS	AMB	POS	AMB	POS	POS
2012_159_Z3492 (26310/11)	POS	POS	POS	POS	POS	POS	NEG	POS	NEG	POS	NEG	NEG	POS	NEG	NEG	NEG	NEG	AMB	POS	POS	POS	NEG	POS	NEG	POS	POS	AMB	NEG	POS	POS	AMB	POS	AMB	POS	POS
2012_161_Z3445 (26430/11)	POS	POS	POS	POS	POS	POS	NEG	POS	AMB	POS	NEG	NEG	POS	NEG	NEG	NEG	NEG	AMB	POS	POS	POS	NEG	POS	AMB	POS	AMB	POS	NEG	POS	POS	AMB	AMB	POS	POS	POS
2012_162_Z3512 (26433/11)	POS	POS	POS	POS	POS	POS	NEG	POS	NEG	POS	NEG	NEG	POS	NEG	NEG	NEG	NEG	AMB	POS	POS	POS	NEG	POS	AMB	POS	POS	AMB	NEG	POS	POS	AMB	POS	AMB	POS	POS
2012_169_Z3495 (27194/11)	POS	POS	POS	POS	POS	POS	NEG	POS	NEG	POS	NEG	NEG	POS	NEG	NEG	NEG	NEG	AMB	POS	POS	POS	NEG	POS	AMB	POS	POS	AMB	NEG	POS	POS	AMB	POS	AMB	POS	POS
2012_170_Z3496 (35/12)	POS	POS	POS	POS	POS	POS	NEG	POS	NEG	POS	NEG	NEG	POS	NEG	NEG	NEG	NEG	AMB	POS	POS	POS	NEG	POS	NEG	POS	POS	AMB	NEG	POS	POS	AMB	POS	AMB	POS	POS
2012_171_Z3448 (47/12)	POS	POS	POS	POS	POS	POS	NEG	POS	NEG	POS	NEG	NEG	POS	NEG	NEG	NEG	NEG	AMB	POS	POS	POS	NEG	POS	AMB	POS	POS	AMB	NEG	POS	POS	AMB	POS	AMB	POS	POS
2012_177_Z3499 (184/12)	POS	POS	POS	POS	POS	POS	NEG	POS	NEG	POS	NEG	NEG	POS	NEG	NEG	NEG	NEG	AMB	POS	POS	POS	NEG	POS	NEG	POS	POS	AMB	NEG	POS	POS	AMB	POS	AMB	POS	POS
2012_179_Z3515 (231/12)	POS	POS	POS	POS	POS	POS	NEG	POS	NEG	POS	NEG	NEG	POS	NEG	NEG	NEG	NEG	AMB	POS	POS	POS	NEG	POS	NEG	POS	POS	AMB	NEG	POS	POS	AMB	POS	AMB	POS	POS
2012_185_Z3502 (437/12)	POS	POS	POS	POS	POS	POS	NEG	POS	NEG	POS	NEG	POS	AMB	NEG	NEG	NEG	NEG	POS	AMB	POS	POS	NEG	POS	NEG	POS	POS	AMB	NEG	POS	POS	AMB	POS	AMB	POS	POS
2012_191_Z3456 (563/12)	POS	POS	POS	POS	POS	POS	NEG	POS	NEG	POS	NEG	POS	AMB	NEG	NEG	NEG	NEG	AMB	POS	POS	POS	NEG	POS	NEG	POS	POS	AMB	NEG	POS	POS	AMB	POS	AMB	POS	POS
2012_194_Z3459	POS	POS	POS	POS	POS	POS	NEG	POS	NEG	POS	NEG	POS	AMB	NEG	NEG	NEG	NEG	AMB	POS	POS	POS	NEG	POS	NEG	POS	POS	AMB	NEG	POS	POS	AMB	POS	AMB	POS	POS
2012_200_Z3460 (820/12)	POS	POS	POS	POS	POS	POS	NEG	POS	AMB	POS	NEG	NEG	POS	NEG	NEG	NEG	NEG	AMB	POS	POS	POS	NEG	POS	AMB	POS	POS	AMB	NEG	POS	POS	AMB	POS	AMB	POS	POS
2012_207_Z3467 (1010/12)	POS	POS	POS	POS	POS	POS	NEG	POS	NEG	POS	NEG	POS	AMB	NEG	NEG	NEG	NEG	POS	AMB	POS	POS	NEG	POS	NEG	POS	POS	AMB	NEG	POS	POS	AMB	POS	AMB	POS	POS
2012_212_Z3471 (1261/12)	POS	POS	POS	POS	POS	POS	NEG	AMB	NEG	POS	NEG	POS	AMB	NEG	NEG	NEG	NEG	POS	AMB	POS	POS	NEG	POS	NEG	POS	AMB	POS	NEG	POS	POS	POS	AMB	AMB	POS	POS
2012_220_Z3477 (1466/12)	POS	POS	POS	POS	POS	POS	NEG	POS	NEG	POS	NEG	POS	AMB	NEG	NEG	NEG	NEG	POS	AMB	POS	POS	NEG	POS	NEG	POS	POS	AMB	NEG	POS	POS	AMB	POS	AMB	POS	POS
2012_224_Z3481 (1522/12)	POS	POS	POS	POS	POS	POS	NEG	POS	NEG	POS	NEG	POS	AMB	NEG	NEG	NEG	NEG	POS	AMB	POS	POS	NEG	POS	NEG	POS	POS	AMB	NEG	POS	POS	AMB	POS	AMB	POS	POS
2012_226_Z3483 (1662/12)	POS	POS	POS	POS	POS	POS	NEG	POS	NEG	POS	NEG	NEG	POS	NEG	NEG	NEG	NEG	AMB	POS	POS	POS	NEG	POS	AMB	POS	POS	AMB	NEG	POS	POS	AMB	POS	AMB	POS	POS
2012_242_Z3527 (2581/12)	POS	POS	POS	POS	POS	POS	NEG	POS	NEG	POS	NEG	NEG	POS	NEG	NEG	NEG	NEG	POS	AMB	POS	POS	NEG	POS	AMB	POS	POS	AMB	NEG	POS	POS	AMB	POS	AMB	POS	POS
2012_245_Z3529 (2687/12)	POS	POS	POS	POS	POS	POS	NEG	POS	NEG	POS	NEG	POS	AMB	NEG	NEG	NEG	NEG	POS	AMB	POS	POS	NEG	POS	NEG	POS	POS	AMB	NEG	POS	POS	AMB	POS	AMB	POS	POS
2012_253_Z3536 (3074/12)	POS	POS	POS	POS	POS	POS	NEG	POS	NEG	POS	NEG	POS	AMB	NEG	NEG	NEG	NEG	POS	AMB	POS	POS	NEG	POS	NEG	POS	POS	AMB	NEG	POS	POS	AMB	POS	AMB	POS	POS
2012_258_Z3545 (3231/12)	POS	POS	POS	POS	POS	POS	NEG	POS	NEG	POS	NEG	NEG	POS	NEG	NEG	NEG	NEG	AMB	POS	POS	POS	NEG	POS	AMB	POS	POS	AMB	NEG	POS	POS	AMB	POS	AMB	POS	POS
2012_261_Z3548 (3298/12)	POS	POS	POS	POS	POS	POS	NEG	POS	POS	POS	POS	NEG	POS	NEG	NEG	NEG	NEG	AMB	POS	POS	POS	NEG	NEG	NEG	POS	POS	AMB	NEG	POS	POS	AMB	POS	AMB	POS	POS
2012_270_Z3557 (3798/12)	POS	POS	POS	POS	POS	POS	NEG	POS	NEG	POS	NEG	POS	AMB	NEG	NEG	NEG	NEG	POS	AMB	POS	POS	NEG	POS	AMB	POS	POS	AMB	NEG	POS	POS	AMB	POS	AMB	POS	POS
2012_274_Z3561 (3861/12)	POS	POS	POS	POS	POS	POS	NEG	POS	NEG	POS	NEG	POS	AMB	NEG	NEG	NEG	NEG	POS	AMB	POS	POS	NEG	POS	NEG	POS	AMB	POS	NEG	POS	POS	AMB	POS	AMB	POS	POS
2012_282_Z3569 (4698/12)	POS	POS	POS	POS	POS	POS	NEG	POS	NEG	POS	NEG	POS	AMB	NEG	NEG	NEG	NEG	POS	AMB	POS	POS	NEG	POS	NEG	POS	POS	AMB	NEG	POS	POS	AMB	POS	AMB	POS	POS
2012_286_Z3573 (4805/12)	POS	POS	POS	POS	POS	POS	NEG	POS	NEG	POS	NEG	POS	AMB	NEG	NEG	NEG	NEG	POS	AMB	POS	POS	NEG	POS	NEG	POS	POS	AMB	NEG	POS	POS	AMB	POS	AMB	POS	POS
2012_293_Z3580 (5007/12)	POS	POS	POS	POS	POS	POS	NEG	POS	NEG	POS	NEG	POS	AMB	NEG	NEG	NEG	NEG	POS	AMB	POS	POS	NEG	POS	NEG	POS	AMB	POS	NEG	POS	POS	AMB	POS	AMB	POS	POS
2012_294_Z3581	POS	POS	POS	POS	POS	POS	NEG	POS	NEG	POS	NEG	POS	AMB	NEG	NEG	NEG	NEG	POS	AMB	POS	POS	NEG	POS	NEG	POS	POS	AMB	NEG	POS	POS	AMB	POS	AMB	POS	POS
2012_307_Z3594 (5918/12)	POS	POS	POS	POS	POS	POS	NEG	POS	NEG	POS	NEG	POS	AMB	NEG	NEG	NEG	NEG	POS	AMB	POS	POS	NEG	POS	AMB	POS	POS	AMB	NEG	POS	POS	AMB	POS	AMB	POS	POS
2012_333_Z3620 (8101/12)	POS	POS	POS	POS	POS	POS	NEG	POS	NEG	POS	NEG	NEG	POS	NEG	NEG	NEG	NEG	POS	AMB	POS	POS	NEG	POS	NEG	POS	POS	AMB	NEG	POS	POS	AMB	POS	AMB	POS	POS
2012_343_Z3630 (8195/12)	POS	POS	POS	POS	POS	POS	NEG	POS	NEG	POS	NEG	POS	AMB	NEG	NEG	NEG	NEG	POS	AMB	POS	POS	NEG	POS	NEG	POS	POS	AMB	NEG	POS	POS	AMB	POS	AMB	POS	POS
2012_349_Z3636 (7999A/12)	POS	POS	POS	POS	POS	POS	NEG	POS	NEG	POS	NEG	POS	AMB	NEG	NEG	NEG	NEG	POS	AMB	POS	POS	NEG	POS	NEG	POS	POS	AMB	NEG	POS	POS	AMB	POS	AMB	POS	POS
2012_353_Z3640 (8575/12)	POS	POS	POS	POS	POS	POS	NEG	POS	NEG	POS	NEG	POS	AMB	NEG	NEG	NEG	NEG	POS	AMB	POS	POS	NEG	POS	AMB	POS	POS	AMB	NEG	POS	POS	AMB	POS	AMB	POS	POS
2012_354_Z3641 (8408/12)	POS	POS	POS	POS	POS	POS	NEG	POS	NEG	POS	NEG	POS	AMB	NEG	NEG	NEG	NEG	POS	AMB	POS	POS	NEG	POS	AMB	POS	POS	AMB	NEG	POS	POS	AMB	POS	AMB	POS	POS
2012_355_Z3642 (1112-58348)	POS	POS	POS	POS	POS	POS	NEG	AMB	NEG	POS	NEG	POS	AMB	NEG	NEG	NEG	NEG	POS	AMB	POS	POS	NEG	POS	NEG	POS	POS	AMB	NEG	POS	POS	AMB	POS	AMB	POS	POS
2012_357_Z3417 (1202-6360)	POS	POS	POS	POS	POS	POS	NEG	POS	NEG	POS	NEG	NEG	POS	NEG	NEG	NEG	NEG	AMB	POS	POS	POS	NEG	POS	AMB	POS	POS	AMB	NEG	POS	POS	AMB	POS	AMB	POS	POS
2012_364_Z3424 (1112-60417)	POS	POS	POS	POS	POS	POS	NEG	POS	NEG	POS	NEG	NEG	POS	NEG	NEG	NEG	NEG	AMB	POS	POS	AMB	NEG	POS	AMB	POS	POS	AMB	NEG	POS	POS	AMB	AMB	POS	POS	POS
2012_376_Z3436 (6256)	POS	POS	POS	POS	POS	POS	NEG	POS	NEG	POS	NEG	NEG	POS	NEG	NEG	NEG	NEG	AMB	POS	POS	POS	NEG	POS	AMB	POS	POS	AMB	NEG	POS	POS	AMB	POS	AMB	POS	POS
2012_383_Z3443	POS	POS	POS	POS	POS	POS	NEG	POS	POS	POS	POS	NEG	POS	NEG	NEG	NEG	NEG	AMB	POS	POS	POS	NEG	POS	AMB	POS	POS	AMB	NEG	POS	POS	AMB	POS	AMB	POS	POS
2013_PA01_Z3984	POS	POS	POS	POS	POS	POS	NEG	POS	NEG	POS	NEG	POS	AMB	NEG	NEG	NEG	NEG	POS	AMB	POS	POS	NEG	POS	NEG	POS	AMB	POS	NEG	POS	POS	AMB	POS	AMB	POS	POS
2013_PA08_Z3991	POS	POS	POS	POS	POS	POS	NEG	POS	NEG	POS	NEG	POS	AMB	NEG	NEG	NEG	NEG	POS	AMB	POS	POS	NEG	POS	NEG	POS	AMB	POS	NEG	POS	POS	AMB	POS	AMB	POS	POS
2013_PA10_Z3993	POS	POS	POS	POS	POS	POS	NEG	POS	NEG	POS	NEG	POS	AMB	NEG	NEG	NEG	NEG	POS	AMB	POS	POS	NEG	POS	NEG	POS	AMB	POS	NEG	POS	POS	AMB	POS	AMB	POS	POS
2013_PA13_Z3996	POS	POS	POS	POS	POS	POS	NEG	POS	NEG	POS	NEG	POS	AMB	NEG	NEG	NEG	NEG	POS	AMB	POS	POS	NEG	POS	NEG	POS	AMB	POS	NEG	POS	POS	AMB	POS	AMB	POS	POS
2013_PA14_Z3997	POS	POS	POS	POS	POS	POS	NEG	POS	NEG	POS	NEG	POS	AMB	NEG	NEG	NEG	NEG	POS	AMB	POS	POS	NEG	POS	NEG	POS	AMB	POS	NEG	POS	POS	AMB	POS	AMB	POS	POS
2013_PA17_Z4000	POS	POS	POS	POS	POS	POS	NEG	POS	NEG	POS	NEG	POS	AMB	NEG	NEG	NEG	NEG	POS	AMB	POS	POS	NEG	POS	NEG	POS	AMB	POS	NEG	POS	POS	POS	AMB	AMB	POS	POS
2013_PA26_Z4009	POS	POS	POS	POS	POS	POS	NEG	AMB	NEG	POS	NEG	POS	AMB	NEG	NEG	NEG	NEG	POS	AMB	POS	POS	NEG	POS	NEG	POS	AMB	POS	NEG	POS	POS	POS	AMB	AMB	POS	POS
2013_PA27_Z4010	POS	POS	POS	POS	POS	POS	NEG	POS	NEG	POS	NEG	POS	AMB	NEG	NEG	NEG	NEG	POS	AMB	POS	POS	NEG	POS	NEG	POS	AMB	POS	NEG	POS	POS	AMB	POS	AMB	POS	POS
2013_PA42_Z4025	POS	POS	POS	POS	POS	POS	NEG	POS	NEG	POS	NEG	POS	AMB	NEG	NEG	NEG	NEG	POS	AMB	POS	POS	NEG	POS	NEG	POS	AMB	POS	NEG	POS	POS	POS	AMB	AMB	POS	POS
2013_PA43_Z4026	POS	POS	POS	POS	POS	POS	NEG	AMB	NEG	POS	NEG	POS	AMB	NEG	NEG	NEG	NEG	POS	NEG	POS	POS	NEG	POS	NEG	POS	AMB	POS	NEG	POS	POS	POS	AMB	NEG	POS	POS
2013_PA45_Z4028	POS	POS	POS	POS	POS	POS	NEG	POS	NEG	POS	NEG	POS	AMB	NEG	NEG	NEG	NEG	POS	AMB	POS	POS	NEG	POS	NEG	POS	AMB	POS	NEG	POS	POS	POS	AMB	AMB	POS	POS
2013_PA56_Z4039	POS	POS	POS	POS	POS	POS	NEG	POS	NEG	POS	NEG	POS	AMB	NEG	NEG	NEG	NEG	POS	AMB	POS	POS	NEG	POS	NEG	POS	AMB	POS	NEG	POS	POS	POS	AMB	AMB	POS	POS
2013_PA58_Z4041	POS	POS	POS	POS	POS	POS	NEG	POS	NEG	POS	NEG	POS	AMB	NEG	NEG	NEG	NEG	POS	AMB	POS	POS	NEG	POS	AMB	POS	AMB	POS	NEG	POS	POS	AMB	POS	AMB	POS	POS
2013_PA64_Z4047	POS	POS	POS	POS	POS	POS	NEG	POS	NEG	POS	NEG	POS	AMB	NEG	NEG	NEG	NEG	POS	AMB	POS	POS	NEG	POS	NEG	POS	AMB	POS	NEG	POS	POS	AMB	POS	AMB	POS	POS
2013_PA66_Z4049	POS	POS	POS	POS	POS	POS	NEG	POS	NEG	POS	NEG	POS	AMB	NEG	NEG	NEG	NEG	POS	AMB	POS	POS	NEG	POS	NEG	POS	AMB	POS	NEG	POS	POS	POS	AMB	AMB	POS	POS
2013_PA67_Z4050	POS	POS	POS	POS	POS	POS	NEG	POS	NEG	POS	NEG	POS	AMB	NEG	NEG	NEG	NEG	POS	AMB	POS	POS	NEG	POS	NEG	POS	AMB	POS	NEG	POS	POS	AMB	POS	AMB	POS	POS
2013_PA69_Z4052	POS	POS	POS	POS	POS	POS	NEG	AMB	NEG	POS	NEG	POS	AMB	NEG	NEG	NEG	NEG	POS	AMB	POS	POS	NEG	POS	NEG	POS	AMB	POS	NEG	POS	POS	AMB	POS	AMB	POS	POS
2013_PA72_Z4055	POS	POS	POS	POS	POS	POS	NEG	POS	NEG	POS	NEG	POS	AMB	NEG	NEG	NEG	NEG	POS	AMB	POS	POS	NEG	POS	NEG	POS	AMB	POS	NEG	POS	POS	AMB	POS	AMB	POS	POS
2013_PA76_Z4059	POS	POS	POS	POS	POS	POS	NEG	POS	NEG	POS	NEG	POS	AMB	NEG	NEG	NEG	NEG	POS	AMB	POS	POS	NEG	POS	NEG	POS	AMB	POS	NEG	POS	POS	AMB	POS	AMB	POS	POS
2013_PA80_Z4063	POS	POS	POS	POS	POS	POS	NEG	POS	NEG	POS	NEG	POS	AMB	NEG	NEG	NEG	NEG	POS	AMB	POS	POS	NEG	POS	NEG	POS	AMB	POS	NEG	POS	POS	AMB	POS	AMB	POS	POS

Strain / Isolates	glutamylendopeptidase	staphopain B, protease	staphopain A (staphylopain A), protease		staphyl. exotoxin-like protein	staphylococcal superantigen-like protein 1											
	sspA	sspB	sspP (consensus)	sspP (other than ST93)	setC	ssl01/set6_probe1_11	ssl01/set6_probe2_11	ssl01/set6_probe1_12	ssl01/set6_probe2_12	ssl01/set6_probe4_11	ssl01/set6_probeRF122	ssl01/set6 (COL)	ssl01/set6 (Mu50+N315)	ssl01/set6 (MW2+MSSA476)	ssl01/set6 (MRSA252)	ssl01/set6 (RF122)	ssl01/set6 (other alleles)
**ST8-MRSA-IV, USA300**																	
2012_158_Z3491 (26049A/11)	POS	POS	POS	POS	POS	POS	NEG	POS	NEG	POS	NEG	NEG	POS	NEG	NEG	NEG	NEG
2012_165_Z3513 (26656/11)	POS	POS	POS	POS	POS	POS	NEG	POS	NEG	POS	NEG	NEG	POS	NEG	NEG	NEG	NEG
2012_214_Z3473 (1322/12)	POS	POS	POS	POS	POS	POS	NEG	POS	NEG	POS	NEG	POS	AMB	NEG	NEG	NEG	NEG
2012_227_Z3484 (1688/12)	POS	POS	POS	POS	POS	POS	NEG	POS	NEG	POS	NEG	POS	AMB	NEG	NEG	NEG	NEG
2012_263_Z3550 (3355/12)	POS	POS	POS	POS	POS	POS	NEG	POS	NEG	POS	NEG	POS	AMB	NEG	NEG	NEG	NEG
2012_266_Z3553	POS	POS	POS	POS	POS	POS	NEG	POS	NEG	POS	NEG	NEG	POS	NEG	NEG	NEG	NEG
2012_284_Z3571 (4729/12)	POS	POS	POS	POS	POS	POS	NEG	POS	NEG	POS	NEG	POS	AMB	NEG	NEG	NEG	NEG
2012_298_Z3585 (5203/12)	POS	POS	POS	POS	POS	POS	NEG	POS	NEG	POS	NEG	POS	AMB	NEG	NEG	NEG	NEG
2012_304_Z3591 (5818/12)	POS	POS	POS	POS	POS	POS	NEG	POS	NEG	POS	NEG	POS	AMB	NEG	NEG	NEG	NEG
Z3595_TT308 - {EFC74B77-3925-484F-A4CF-A1F7DC	POS	POS	POS	POS	POS	POS	NEG	POS	NEG	POS	NEG	POS	AMB	NEG	NEG	NEG	NEG
2012_369_Z3429 (1112-58547)	POS	POS	POS	POS	POS	POS	NEG	POS	NEG	POS	NEG	POS	AMB	NEG	NEG	NEG	NEG
2012_374_Z3434 (1203-13527)	POS	POS	POS	POS	POS	POS	NEG	POS	NEG	POS	NEG	POS	AMB	NEG	NEG	NEG	NEG
2013_PA06_Z3989	POS	POS	POS	POS	POS	POS	NEG	POS	NEG	POS	NEG	POS	AMB	NEG	NEG	NEG	NEG
2013_PA55_Z4038	POS	POS	POS	POS	POS	POS	NEG	POS	NEG	POS	NEG	POS	AMB	NEG	NEG	NEG	NEG
2013_PA71_Z4054	POS	POS	POS	POS	POS	POS	NEG	AMB	NEG	POS	NEG	POS	AMB	NEG	NEG	NEG	NEG
**ST8-MRSA-IV [PVL+/ACME+], US**																	
2012_180_Z3501 (232/12)	POS	POS	POS	POS	POS	POS	NEG	POS	NEG	POS	NEG	POS	AMB	NEG	NEG	NEG	NEG
2013_PA68_Z4051	POS	POS	POS	POS	POS	POS	NEG	POS	NEG	POS	NEG	POS	AMB	NEG	NEG	NEG	NEG
**ST72-MSSA**																	
2012_174_Z3451 (102/12)	POS	POS	POS	POS	POS	POS	AMB	POS	NEG	POS	NEG	POS	AMB	NEG	NEG	NEG	NEG
2012_223_Z3480 (1506A/12)	POS	POS	POS	POS	POS	POS	NEG	NEG	NEG	NEG	NEG	NEG	NEG	NEG	NEG	NEG	POS
2012_236_Z3523 (2086/12)	POS	POS	POS	POS	POS	POS	NEG	POS	NEG	NEG	NEG	NEG	POS	NEG	NEG	NEG	NEG
2012_239_Z3525 (2346/12)	POS	POS	POS	POS	POS	POS	NEG	POS	NEG	AMB	NEG	NEG	POS	NEG	NEG	NEG	NEG
2012_252_Z3535 (3030/12)	POS	POS	POS	POS	POS	POS	AMB	POS	NEG	POS	NEG	NEG	POS	NEG	NEG	NEG	NEG
2012_271_Z3558 (3799/12)	POS	POS	POS	POS	POS	POS	NEG	POS	NEG	AMB	NEG	NEG	POS	NEG	NEG	NEG	NEG
2012_276_Z3563	POS	POS	POS	POS	POS	POS	POS	POS	NEG	AMB	NEG	NEG	POS	NEG	POS	NEG	NEG
2012_300_Z3587 (5638/12)	POS	POS	POS	POS	POS	POS	NEG	AMB	NEG	NEG	NEG	NEG	NEG	NEG	NEG	NEG	POS
2012_316_Z3603 (6941/12)	POS	POS	POS	POS	POS	POS	NEG	AMB	NEG	AMB	NEG	NEG	NEG	NEG	NEG	NEG	POS
2012_319_Z3606 (7019/12)	POS	POS	POS	POS	POS	POS	NEG	AMB	NEG	NEG	NEG	NEG	NEG	NEG	NEG	NEG	POS
2012_371_Z3431 (1204-17577)	POS	POS	POS	POS	POS	POS	AMB	POS	NEG	POS	NEG	NEG	POS	NEG	NEG	NEG	NEG
2013_PA05_Z3988	POS	POS	POS	POS	POS	POS	NEG	AMB	NEG	NEG	NEG	NEG	NEG	NEG	NEG	NEG	POS
2013_PA65_Z4048	POS	POS	POS	POS	POS	POS	NEG	NEG	NEG	NEG	NEG	NEG	NEG	NEG	NEG	NEG	POS
2013_PA73_Z4056	POS	POS	POS	POS	POS	POS	NEG	AMB	NEG	NEG	NEG	NEG	NEG	NEG	NEG	NEG	POS
**ST72-MSSA-SCCfus**																	
2013_PA16_Z3999	POS	POS	POS	POS	POS	POS	NEG	NEG	NEG	NEG	NEG	NEG	NEG	NEG	NEG	NEG	POS
**ST72-MSSA-SCCfus/kdp**																	
2012_361_Z3421 (1201-1299)	POS	POS	POS	POS	POS	POS	NEG	AMB	NEG	NEG	NEG	NEG	NEG	NEG	NEG	NEG	POS
**ST239-MRSA-III&SCCmer**																	
2012_157_Z3490	POS	POS	POS	POS	POS	POS	NEG	POS	NEG	POS	NEG	NEG	POS	NEG	NEG	NEG	NEG
2012_168_Z3494 (27129/11)	POS	POS	POS	POS	POS	POS	NEG	POS	NEG	POS	NEG	NEG	POS	NEG	NEG	NEG	NEG
2012_195_Z3508 (619/12)	POS	POS	POS	POS	POS	POS	NEG	POS	NEG	POS	NEG	POS	AMB	NEG	NEG	NEG	NEG
2012_222_Z3479 (1489/12)	POS	POS	POS	POS	POS	POS	NEG	POS	NEG	POS	NEG	POS	AMB	NEG	NEG	NEG	NEG
2012_233_Z3540 (1943/12)	POS	POS	POS	POS	POS	POS	NEG	POS	NEG	POS	NEG	POS	AMB	NEG	NEG	NEG	NEG
2012_237_Z3541	POS	POS	POS	POS	POS	POS	NEG	POS	NEG	POS	NEG	POS	AMB	NEG	NEG	NEG	NEG
2012_262_Z3549	POS	POS	POS	POS	POS	POS	NEG	POS	NEG	POS	NEG	NEG	POS	NEG	NEG	NEG	NEG
2012_275_Z3562 (3893/12)	POS	POS	POS	POS	POS	POS	NEG	POS	NEG	POS	NEG	POS	AMB	NEG	NEG	NEG	NEG
2012_291_Z3578	POS	POS	POS	POS	POS	POS	NEG	POS	NEG	POS	NEG	POS	AMB	NEG	NEG	NEG	NEG
2012_314_Z3601 (6939/12)	POS	POS	POS	POS	POS	POS	NEG	POS	NEG	POS	NEG	POS	AMB	NEG	NEG	NEG	NEG
2012323_Z3610	POS	POS	POS	POS	POS	POS	NEG	POS	NEG	POS	NEG	NEG	POS	NEG	NEG	NEG	NEG
2012330_Z3617 (7605/12)	POS	POS	POS	POS	POS	POS	NEG	POS	NEG	POS	NEG	NEG	POS	NEG	NEG	NEG	NEG
2012332_Z3619 (8076/12)	POS	POS	POS	POS	POS	POS	NEG	POS	NEG	POS	NEG	NEG	POS	NEG	NEG	NEG	NEG
2012339_Z3626 (8558/12)	POS	POS	POS	POS	POS	POS	NEG	POS	NEG	POS	NEG	POS	AMB	NEG	NEG	NEG	NEG
2012340_Z3627 (8523/12)	POS	POS	POS	POS	POS	POS	NEG	POS	NEG	POS	NEG	POS	AMB	NEG	NEG	NEG	NEG
2012348_Z3635	POS	POS	POS	POS	POS	POS	NEG	POS	NEG	POS	NEG	POS	AMB	NEG	NEG	NEG	NEG
2012350_Z3637	POS	POS	POS	POS	POS	POS	NEG	POS	NEG	POS	NEG	POS	AMB	NEG	NEG	NEG	NEG
2012_370_Z3430 (1112-52177)	POS	POS	POS	POS	POS	POS	NEG	POS	NEG	POS	NEG	NEG	POS	NEG	NEG	NEG	NEG
2012_378_Z3438 (4139)	POS	POS	POS	POS	POS	POS	NEG	POS	NEG	POS	NEG	POS	AMB	NEG	NEG	NEG	NEG
2013_PA21_Z4004	POS	POS	POS	POS	POS	POS	NEG	NEG	NEG	POS	NEG	POS	AMB	NEG	NEG	NEG	NEG
2013_PA22_Z4005	POS	POS	POS	POS	POS	POS	NEG	AMB	NEG	POS	NEG	POS	AMB	NEG	NEG	NEG	NEG
2013_PA24_Z4007	POS	POS	POS	POS	POS	POS	NEG	NEG	NEG	POS	NEG	POS	AMB	NEG	NEG	NEG	NEG
2013_PA31_Z4014	POS	POS	POS	POS	POS	POS	NEG	AMB	NEG	POS	NEG	POS	AMB	NEG	NEG	NEG	NEG
2013_PA35_Z4018	POS	POS	POS	POS	POS	POS	NEG	NEG	NEG	POS	NEG	POS	AMB	NEG	NEG	NEG	NEG
2013_PA81_Z4064	POS	POS	POS	POS	POS	POS	NEG	NEG	NEG	POS	NEG	POS	AMB	NEG	NEG	NEG	NEG
**ST239-MRSA-III+ccrA/B4**																	
2012_254_Z3537 (3124A/12)	POS	POS	POS	POS	POS	POS	NEG	AMB	NEG	POS	NEG	POS	AMB	NEG	NEG	NEG	NEG
2013_PA25_Z4008	POS	POS	POS	POS	POS	POS	NEG	POS	NEG	POS	NEG	POS	AMB	NEG	NEG	NEG	NEG
**ST834-MSSA**																	
2012_268_Z3555 (3693/12)	POS	POS	POS	POS	POS	NEG	POS	AMB	NEG	AMB	NEG	NEG	NEG	NEG	NEG	NEG	POS
**CC12-MSSA**																	
2012_210_Z3470 (1177/12)	POS	POS	POS	POS	POS	NEG	NEG	POS	NEG	POS	POS	NEG	NEG	NEG	NEG	NEG	POS
2012_230_Z3487 (1797/12)	POS	POS	POS	POS	POS	NEG	NEG	POS	NEG	POS	POS	NEG	NEG	NEG	NEG	NEG	POS
2013_PA18_Z4001	POS	POS	POS	POS	POS	NEG	NEG	AMB	NEG	POS	POS	NEG	NEG	NEG	NEG	NEG	POS
**CC15-MSSA**																	
2012_160_Z3444 (26386/11)	POS	POS	POS	POS	POS	NEG	POS	NEG	NEG	POS	POS	NEG	NEG	NEG	NEG	NEG	POS
2012_167_Z3514 (27010/11)	POS	POS	POS	POS	POS	NEG	POS	NEG	NEG	POS	POS	NEG	NEG	NEG	NEG	NEG	POS
2012_184_Z3455 (376/12)	POS	POS	POS	POS	POS	NEG	POS	NEG	NEG	POS	AMB	NEG	NEG	NEG	NEG	NEG	POS
2012_205_Z3465 (939/12)	POS	POS	POS	POS	POS	NEG	POS	NEG	NEG	POS	AMB	NEG	NEG	NEG	NEG	NEG	POS
2012_231_Z3519 (1831/12)	POS	POS	POS	POS	POS	NEG	POS	NEG	NEG	POS	POS	NEG	NEG	NEG	NEG	NEG	POS
2012_249_Z3533 (2907/12)	POS	POS	POS	POS	POS	NEG	POS	NEG	NEG	POS	NEG	NEG	NEG	NEG	NEG	NEG	POS
2012_305_Z3592 (5839/12)	POS	POS	POS	POS	POS	NEG	POS	NEG	NEG	POS	NEG	NEG	NEG	NEG	NEG	NEG	POS
2012_313_Z3600 (6857/12)	POS	POS	POS	POS	POS	NEG	POS	NEG	NEG	POS	POS	NEG	NEG	NEG	NEG	NEG	POS
2012_345_Z3632 (7966B/12)	POS	POS	POS	POS	POS	NEG	POS	NEG	NEG	POS	NEG	NEG	NEG	NEG	NEG	NEG	POS
2013_PA33_Z4016	POS	POS	POS	POS	POS	NEG	NEG	NEG	NEG	POS	NEG	NEG	NEG	NEG	NEG	NEG	POS
**CC20-MSSA**																	
2012_240_Z3526 (2354/12)	POS	POS	POS	POS	POS	AMB	POS	AMB	POS	NEG	POS	NEG	NEG	POS	NEG	NEG	NEG
**CC30-MSSA**																	
2012_155_Z3488 (25905A/11)	POS	POS	POS	POS	NEG	NEG	POS	POS	NEG	POS	NEG	NEG	NEG	NEG	POS	NEG	NEG
**CC30-MSSA [PVL+]**																	
2012_164_Z3447 (26574/11)	POS	POS	POS	POS	NEG	NEG	POS	POS	AMB	POS	NEG	NEG	NEG	NEG	POS	NEG	NEG
2012_166_Z3493 (26988A/11)	POS	POS	POS	POS	NEG	NEG	POS	POS	NEG	POS	NEG	NEG	NEG	NEG	POS	NEG	NEG
2012_178_Z3500 (197/12)	POS	POS	POS	POS	NEG	NEG	POS	POS	NEG	POS	NEG	NEG	NEG	NEG	POS	NEG	NEG
2012_183_Z3454	POS	POS	POS	POS	NEG	NEG	POS	POS	NEG	POS	NEG	NEG	NEG	NEG	POS	NEG	NEG
2012_186_Z3503 (444/12)	POS	POS	POS	POS	NEG	NEG	POS	POS	NEG	POS	NEG	NEG	NEG	NEG	POS	NEG	NEG
2012_189_Z3506 (542/12)	POS	POS	POS	POS	NEG	NEG	POS	POS	NEG	POS	NEG	NEG	NEG	NEG	POS	NEG	NEG
2012_192_Z3457 (606/12)	POS	POS	POS	POS	NEG	NEG	POS	POS	NEG	POS	NEG	NEG	NEG	NEG	POS	NEG	NEG
2012_196_Z3509 (707/12)	POS	POS	POS	POS	NEG	NEG	POS	POS	NEG	POS	NEG	NEG	NEG	NEG	POS	NEG	NEG
2012_218_Z3476 (1432/12)	POS	POS	POS	POS	NEG	NEG	POS	POS	NEG	POS	NEG	NEG	NEG	NEG	POS	NEG	NEG
2012_232_Z3520 (1892/12)	POS	POS	POS	POS	NEG	NEG	POS	POS	NEG	POS	NEG	NEG	NEG	NEG	POS	NEG	NEG
2012_278_Z3565 (4397/12)	POS	POS	POS	POS	NEG	NEG	POS	POS	AMB	POS	NEG	NEG	NEG	NEG	POS	NEG	NEG
2012_285_Z3572 (4802/12)	POS	POS	POS	POS	NEG	NEG	POS	POS	NEG	POS	NEG	NEG	NEG	NEG	POS	NEG	NEG
2012_287_Z3574 (4906/12)	POS	POS	POS	POS	NEG	NEG	POS	POS	NEG	POS	NEG	NEG	NEG	NEG	POS	NEG	NEG
2012_295_Z3582 (5116/12)	POS	POS	POS	POS	NEG	NEG	POS	POS	NEG	POS	NEG	NEG	NEG	NEG	POS	NEG	NEG
2012_326_Z3613 (7321/12)	POS	POS	POS	POS	NEG	NEG	POS	POS	AMB	POS	NEG	NEG	NEG	NEG	POS	NEG	NEG
2012_356_Z3643 (1204-15897)	POS	POS	POS	POS	NEG	NEG	POS	POS	NEG	POS	NEG	NEG	NEG	NEG	POS	NEG	NEG
2012_366_Z3426 (1204-16124)	POS	POS	POS	POS	NEG	NEG	POS	POS	NEG	POS	NEG	NEG	NEG	NEG	POS	NEG	NEG
2012_372_Z3432 (1205-19821)	POS	POS	POS	POS	NEG	NEG	POS	POS	NEG	POS	NEG	NEG	NEG	NEG	POS	NEG	NEG
2012_380_Z3440 (4141)	POS	POS	POS	POS	NEG	NEG	POS	POS	NEG	POS	NEG	NEG	NEG	NEG	POS	NEG	NEG
2013_PA20_Z4003	POS	POS	POS	POS	NEG	NEG	POS	POS	NEG	POS	NEG	NEG	NEG	NEG	POS	NEG	NEG
2013_PA36_Z4019	POS	POS	POS	POS	NEG	NEG	POS	POS	NEG	NEG	NEG	NEG	NEG	NEG	POS	NEG	NEG
2013_PA46_Z4029	POS	POS	POS	POS	NEG	NEG	POS	POS	NEG	AMB	NEG	NEG	NEG	NEG	POS	NEG	NEG
2013_PA50_Z4033	POS	POS	POS	POS	NEG	NEG	POS	POS	NEG	AMB	NEG	NEG	NEG	NEG	POS	NEG	NEG
2013_PA63_Z4046	POS	POS	POS	POS	NEG	NEG	POS	POS	NEG	POS	NEG	NEG	NEG	NEG	POS	NEG	NEG
2013_PA78_Z4061	POS	POS	POS	POS	NEG	NEG	POS	POS	NEG	POS	NEG	NEG	NEG	NEG	POS	NEG	NEG
2013_PA79_Z4062	POS	POS	POS	POS	NEG	NEG	POS	POS	NEG	POS	NEG	NEG	NEG	NEG	POS	NEG	NEG
**CC45-MSSA**																	
2012_172_Z3449 (49/12)	POS	POS	POS	POS	POS	NEG	POS	POS	NEG	POS	NEG	NEG	NEG	NEG	POS	NEG	NEG
2012_208_Z3468 (1011/12)	POS	POS	POS	POS	POS	NEG	POS	POS	NEG	POS	NEG	NEG	NEG	NEG	POS	NEG	NEG
2012_235_Z3522 (2085/12)	POS	POS	POS	POS	POS	NEG	POS	POS	NEG	POS	NEG	NEG	NEG	NEG	POS	NEG	NEG
2012_290_Z3577 (4921/12)	POS	POS	POS	POS	POS	NEG	POS	POS	NEG	POS	NEG	NEG	NEG	NEG	POS	NEG	NEG
2012_311_Z3598 (6182/12)	POS	POS	POS	POS	POS	NEG	POS	POS	NEG	POS	NEG	NEG	NEG	NEG	POS	NEG	NEG
Z3604_TT317 - {DC558C18-2959-4049-B8F3-507365	POS	POS	POS	POS	POS	NEG	POS	POS	NEG	POS	NEG	NEG	NEG	NEG	POS	NEG	NEG
2012_363_Z3423 (1112-61058)	POS	POS	POS	POS	POS	NEG	POS	POS	NEG	POS	NEG	NEG	NEG	NEG	POS	NEG	NEG
2013_PA19_Z4002	POS	POS	POS	POS	POS	NEG	POS	AMB	NEG	POS	NEG	NEG	NEG	NEG	NEG	NEG	POS
2013_PA54_Z4037	POS	POS	POS	POS	POS	NEG	POS	POS	NEG	POS	NEG	NEG	NEG	NEG	POS	NEG	NEG
2013_PA82_Z4065	POS	POS	POS	POS	AMB	NEG	POS	AMB	NEG	POS	NEG	NEG	NEG	NEG	NEG	NEG	POS
**CC97-MSSA**																	
2012_182_Z3453 (279A/12)	POS	POS	POS	POS	POS	NEG	NEG	AMB	NEG	AMB	POS	NEG	NEG	NEG	NEG	NEG	POS
2012_187_Z3504	POS	POS	POS	POS	POS	NEG	NEG	NEG	NEG	NEG	POS	NEG	NEG	NEG	NEG	POS	NEG
2012_197_Z3510 (757/12)	POS	POS	POS	POS	POS	NEG	NEG	AMB	NEG	NEG	POS	NEG	NEG	NEG	NEG	NEG	POS
2012_199_Z3516 (785/12)	POS	POS	POS	POS	POS	NEG	NEG	AMB	NEG	POS	POS	NEG	NEG	NEG	NEG	NEG	POS
2013_PA15_Z3998	POS	POS	POS	POS	POS	NEG	NEG	NEG	NEG	AMB	POS	NEG	NEG	NEG	NEG	NEG	POS
**CC101-MSSA**																	
2012_283_Z3570 (4725/12)	POS	POS	POS	POS	POS	NEG	POS	NEG	POS	NEG	NEG	NEG	NEG	POS	NEG	NEG	NEG
2012_247_Z3531 (2788/12)	POS	POS	POS	POS	POS	NEG	POS	NEG	POS	NEG	NEG	NEG	NEG	POS	NEG	NEG	NEG
2012_279_Z3566 (4506/12)	POS	POS	POS	POS	POS	NEG	POS	NEG	POS	NEG	NEG	NEG	NEG	POS	NEG	NEG	NEG

Strain / Isolates	staphylococcal superantigen-like protein 2		staphylococcal superantigen-like protein 3			staphylococcal superantigen-like protein 4		staphylococcal superantigen-like protein 5				staphylococcal superantigen-like protein 6		staphylococcal superantigen-like protein 7			staphylococcal superantigen-like protein 8	
	ssl02/set7	ssl02/set7 (MRSA252)	ssl03/set8_probe 1	ssl03/set8_probe 2	ssl03/set8 (MRSA252, SAR0424)	ssl04/set9	ssl04/set9 (MRSA252, SAR0425)	ssl05/set3_probe 1	ssl05/set3 (RF122, probe-611)	ssl05/set3_probe 2 (612)	ssl05/set3 (MRSA252)	ssl06/set21	ssl06 (NCTC8325+MW2)	ssl07/set1	ssl07/set1 (MRSA252)	ssl07/set1 (AF188836)	ssl08/set12_probe 1	ssl08/set12_probe 2
**ST8-MRSA-IV, USA300**																		
2012_158_Z3491 (26049A/11)	AMB	POS	POS	POS	NEG	POS	AMB	POS	POS	AMB	NEG	POS	POS	AMB	POS	AMB	POS	POS
2012_165_Z3513 (26656/11)	AMB	POS	POS	POS	NEG	POS	AMB	POS	POS	AMB	NEG	POS	POS	AMB	POS	AMB	POS	POS
2012_214_Z3473 (1322/12)	POS	AMB	POS	POS	NEG	POS	NEG	POS	AMB	POS	NEG	POS	POS	AMB	POS	AMB	POS	POS
2012_227_Z3484 (1688/12)	AMB	POS	POS	POS	NEG	POS	AMB	POS	POS	AMB	NEG	POS	POS	AMB	POS	AMB	POS	POS
2012_263_Z3550 (3355/12)	POS	AMB	POS	POS	NEG	POS	AMB	POS	POS	AMB	NEG	POS	POS	AMB	POS	AMB	POS	POS
2012_266_Z3553	POS	AMB	POS	POS	NEG	POS	AMB	POS	POS	AMB	NEG	POS	POS	AMB	POS	AMB	POS	POS
2012_284_Z3571 (4729/12)	POS	AMB	POS	POS	NEG	POS	NEG	POS	POS	AMB	NEG	POS	POS	AMB	POS	AMB	POS	POS
2012_298_Z3585 (5203/12)	POS	AMB	POS	POS	NEG	POS	NEG	POS	POS	AMB	NEG	POS	POS	AMB	POS	AMB	POS	POS
2012_304_Z3591 (5818/12)	POS	AMB	POS	POS	NEG	POS	NEG	POS	POS	AMB	NEG	POS	POS	AMB	POS	AMB	POS	POS
Z3595_TT308 - {EFC74B77-3925-484F-A4CF-A1F7DC	POS	AMB	POS	POS	NEG	POS	NEG	POS	AMB	POS	NEG	POS	POS	AMB	POS	AMB	POS	POS
2012_369_Z3429 (1112-58547)	AMB	POS	AMB	NEG	NEG	POS	AMB	POS	POS	AMB	NEG	NEG	POS	AMB	POS	AMB	POS	POS
2012_374_Z3434 (1203-13527)	POS	AMB	POS	POS	NEG	POS	NEG	POS	POS	AMB	NEG	POS	POS	AMB	POS	AMB	POS	POS
2013_PA06_Z3989	POS	AMB	POS	POS	NEG	POS	NEG	POS	AMB	POS	NEG	POS	POS	AMB	POS	AMB	POS	POS
2013_PA55_Z4038	POS	AMB	POS	POS	NEG	POS	NEG	POS	AMB	POS	NEG	POS	POS	AMB	POS	AMB	POS	POS
2013_PA71_Z4054	POS	AMB	POS	POS	NEG	POS	NEG	POS	AMB	POS	NEG	POS	POS	POS	AMB	AMB	POS	POS
**ST8-MRSA-IV [PVL+/ACME+], US**																		
2012_180_Z3501 (232/12)	POS	AMB	POS	POS	NEG	POS	NEG	POS	POS	AMB	NEG	POS	POS	AMB	POS	AMB	POS	POS
2013_PA68_Z4051	POS	AMB	POS	POS	NEG	POS	NEG	POS	AMB	POS	NEG	POS	POS	AMB	POS	AMB	POS	POS
**ST72-MSSA**																		
2012_174_Z3451 (102/12)	AMB	POS	POS	POS	NEG	POS	AMB	POS	AMB	POS	AMB	NEG	AMB	AMB	POS	AMB	POS	POS
2012_223_Z3480 (1506A/12)	POS	AMB	POS	POS	NEG	POS	NEG	POS	AMB	POS	NEG	NEG	NEG	POS	AMB	AMB	POS	POS
2012_236_Z3523 (2086/12)	POS	AMB	POS	POS	NEG	POS	AMB	POS	AMB	POS	NEG	NEG	NEG	AMB	POS	AMB	POS	POS
2012_239_Z3525 (2346/12)	POS	AMB	POS	POS	NEG	POS	AMB	POS	AMB	POS	NEG	NEG	NEG	AMB	POS	AMB	POS	POS
2012_252_Z3535 (3030/12)	POS	AMB	POS	POS	NEG	POS	AMB	POS	AMB	POS	POS	NEG	AMB	AMB	POS	AMB	POS	POS
2012_271_Z3558 (3799/12)	POS	AMB	POS	POS	NEG	POS	NEG	POS	AMB	POS	AMB	NEG	NEG	AMB	POS	AMB	POS	POS
2012_276_Z3563	POS	AMB	POS	POS	NEG	POS	POS	POS	AMB	POS	NEG	NEG	NEG	AMB	POS	AMB	POS	POS
2012_300_Z3587 (5638/12)	POS	AMB	POS	POS	NEG	POS	NEG	POS	AMB	POS	NEG	NEG	NEG	AMB	POS	AMB	POS	POS
2012_316_Z3603 (6941/12)	POS	AMB	POS	POS	NEG	POS	AMB	POS	AMB	POS	NEG	NEG	NEG	AMB	POS	AMB	POS	POS
2012_319_Z3606 (7019/12)	POS	AMB	POS	POS	NEG	POS	AMB	POS	AMB	POS	NEG	NEG	NEG	AMB	POS	AMB	POS	POS
2012_371_Z3431 (1204-17577)	AMB	POS	POS	POS	NEG	POS	AMB	POS	AMB	POS	AMB	NEG	AMB	AMB	POS	AMB	POS	POS
2013_PA05_Z3988	POS	AMB	POS	POS	NEG	POS	NEG	POS	AMB	POS	NEG	NEG	NEG	POS	AMB	AMB	POS	POS
2013_PA65_Z4048	POS	NEG	POS	POS	NEG	POS	NEG	POS	POS	AMB	NEG	NEG	NEG	POS	AMB	NEG	POS	POS
2013_PA73_Z4056	POS	AMB	POS	POS	NEG	POS	AMB	POS	AMB	POS	NEG	NEG	AMB	AMB	POS	AMB	POS	POS
**ST72-MSSA-SCCfus**																		
2013_PA16_Z3999	POS	AMB	POS	POS	NEG	POS	NEG	POS	AMB	POS	NEG	NEG	NEG	POS	AMB	AMB	POS	POS
**ST72-MSSA-SCCfus/kdp**																		
2012_361_Z3421 (1201-1299)	POS	AMB	POS	POS	NEG	POS	NEG	POS	AMB	POS	NEG	NEG	NEG	AMB	POS	AMB	POS	POS
**ST239-MRSA-III&SCCmer**																		
2012_157_Z3490	AMB	POS	POS	POS	NEG	POS	NEG	POS	POS	AMB	NEG	POS	POS	AMB	POS	AMB	POS	POS
2012_168_Z3494 (27129/11)	AMB	POS	POS	POS	NEG	POS	NEG	POS	POS	AMB	NEG	POS	POS	AMB	POS	AMB	POS	POS
2012_195_Z3508 (619/12)	POS	AMB	POS	POS	NEG	POS	NEG	POS	POS	AMB	NEG	POS	POS	AMB	POS	AMB	POS	POS
2012_222_Z3479 (1489/12)	POS	AMB	POS	POS	NEG	POS	NEG	POS	POS	AMB	NEG	POS	POS	AMB	POS	AMB	POS	POS
2012_233_Z3540 (1943/12)	POS	AMB	POS	POS	NEG	POS	NEG	POS	AMB	POS	NEG	POS	POS	AMB	POS	AMB	POS	POS
2012_237_Z3541	POS	AMB	POS	POS	NEG	POS	NEG	POS	POS	AMB	NEG	POS	POS	AMB	POS	AMB	POS	POS
2012_262_Z3549	POS	AMB	POS	POS	NEG	POS	AMB	POS	POS	AMB	NEG	POS	POS	AMB	POS	AMB	POS	POS
2012_275_Z3562 (3893/12)	POS	AMB	POS	POS	NEG	POS	AMB	POS	POS	AMB	NEG	POS	POS	AMB	POS	AMB	POS	POS
2012_291_Z3578	POS	AMB	POS	POS	NEG	POS	NEG	POS	POS	AMB	NEG	POS	POS	AMB	POS	AMB	POS	POS
2012_314_Z3601 (6939/12)	POS	AMB	POS	POS	NEG	POS	NEG	POS	POS	AMB	NEG	POS	POS	AMB	POS	AMB	POS	POS
2012323_Z3610	AMB	POS	POS	POS	NEG	POS	AMB	POS	POS	AMB	NEG	POS	POS	AMB	POS	AMB	POS	POS
2012330_Z3617 (7605/12)	AMB	POS	POS	POS	NEG	POS	AMB	POS	POS	AMB	NEG	POS	POS	AMB	POS	AMB	POS	POS
2012332_Z3619 (8076/12)	POS	AMB	POS	POS	NEG	POS	NEG	POS	POS	AMB	NEG	POS	POS	AMB	POS	AMB	POS	POS
2012339_Z3626 (8558/12)	POS	AMB	POS	POS	NEG	POS	NEG	POS	POS	AMB	NEG	POS	POS	AMB	POS	AMB	POS	POS
2012340_Z3627 (8523/12)	POS	AMB	POS	POS	NEG	POS	NEG	POS	POS	AMB	NEG	POS	POS	AMB	POS	AMB	POS	POS
2012348_Z3635	POS	AMB	POS	POS	NEG	POS	NEG	POS	POS	AMB	NEG	POS	POS	AMB	POS	AMB	POS	POS
2012350_Z3637	POS	AMB	POS	POS	NEG	POS	NEG	POS	POS	AMB	NEG	POS	POS	AMB	POS	AMB	POS	POS
2012_370_Z3430 (1112-52177)	POS	AMB	POS	POS	NEG	POS	AMB	POS	POS	AMB	NEG	POS	POS	AMB	POS	AMB	POS	POS
2012_378_Z3438 (4139)	POS	AMB	POS	POS	NEG	POS	NEG	POS	POS	AMB	NEG	POS	POS	AMB	POS	AMB	POS	POS
2013_PA21_Z4004	POS	NEG	POS	POS	NEG	POS	NEG	POS	AMB	POS	NEG	POS	POS	POS	AMB	NEG	POS	POS
2013_PA22_Z4005	POS	AMB	POS	POS	NEG	POS	AMB	POS	AMB	POS	NEG	POS	POS	AMB	POS	AMB	POS	POS
2013_PA24_Z4007	POS	NEG	POS	POS	NEG	POS	NEG	POS	AMB	POS	NEG	POS	POS	POS	AMB	NEG	POS	POS
2013_PA31_Z4014	POS	AMB	POS	POS	NEG	POS	NEG	POS	AMB	POS	NEG	POS	POS	POS	AMB	AMB	POS	POS
2013_PA35_Z4018	POS	NEG	POS	POS	NEG	POS	NEG	POS	NEG	POS	NEG	POS	POS	POS	AMB	NEG	POS	POS
2013_PA81_Z4064	POS	AMB	POS	POS	NEG	POS	NEG	POS	AMB	POS	NEG	POS	POS	POS	AMB	AMB	POS	POS
**ST239-MRSA-III+ccrA/B4**																		
2012_254_Z3537 (3124A/12)	POS	AMB	POS	POS	NEG	POS	NEG	POS	AMB	POS	NEG	POS	POS	POS	AMB	NEG	POS	POS
2013_PA25_Z4008	POS	AMB	POS	POS	NEG	POS	NEG	POS	AMB	POS	NEG	POS	POS	AMB	POS	AMB	POS	POS
**ST834-MSSA**																		
2012_268_Z3555 (3693/12)	POS	AMB	POS	POS	NEG	POS	POS	POS	POS	AMB	NEG	NEG	AMB	AMB	POS	AMB	POS	POS
**CC12-MSSA**																		
2012_210_Z3470 (1177/12)	POS	AMB	POS	POS	NEG	POS	NEG	POS	AMB	POS	NEG	NEG	NEG	POS	NEG	NEG	POS	POS
2012_230_Z3487 (1797/12)	POS	AMB	POS	POS	NEG	POS	POS	POS	AMB	POS	NEG	NEG	AMB	POS	AMB	NEG	POS	POS
2013_PA18_Z4001	POS	AMB	POS	POS	NEG	POS	AMB	POS	AMB	POS	NEG	NEG	NEG	POS	NEG	NEG	POS	POS
**CC15-MSSA**																		
2012_160_Z3444 (26386/11)	AMB	POS	POS	POS	NEG	POS	AMB	POS	AMB	POS	AMB	POS	POS	AMB	POS	AMB	POS	POS
2012_167_Z3514 (27010/11)	AMB	POS	POS	POS	NEG	POS	AMB	POS	AMB	POS	AMB	POS	POS	AMB	POS	AMB	POS	POS
2012_184_Z3455 (376/12)	AMB	POS	POS	POS	NEG	POS	AMB	POS	AMB	POS	AMB	POS	POS	AMB	POS	AMB	POS	POS
2012_205_Z3465 (939/12)	AMB	POS	POS	POS	NEG	POS	AMB	POS	AMB	POS	AMB	POS	POS	AMB	POS	AMB	POS	POS
2012_231_Z3519 (1831/12)	AMB	POS	POS	POS	NEG	POS	AMB	POS	AMB	POS	AMB	POS	POS	AMB	POS	AMB	POS	POS
2012_249_Z3533 (2907/12)	POS	AMB	POS	POS	NEG	POS	NEG	POS	AMB	POS	NEG	POS	POS	AMB	POS	AMB	POS	POS
2012_305_Z3592 (5839/12)	POS	AMB	POS	POS	NEG	POS	AMB	POS	AMB	POS	NEG	POS	POS	AMB	POS	AMB	POS	POS
2012_313_Z3600 (6857/12)	POS	AMB	POS	POS	NEG	POS	AMB	POS	AMB	POS	NEG	POS	POS	AMB	POS	AMB	POS	POS
2012_345_Z3632 (7966B/12)	POS	AMB	POS	POS	NEG	POS	NEG	POS	AMB	POS	NEG	POS	POS	AMB	POS	AMB	POS	POS
2013_PA33_Z4016	POS	NEG	POS	POS	NEG	POS	NEG	POS	POS	AMB	NEG	POS	POS	POS	AMB	NEG	NEG	POS
**CC20-MSSA**																		
2012_240_Z3526 (2354/12)	POS	NEG	POS	POS	NEG	POS	AMB	NEG	POS	AMB	NEG	POS	POS	AMB	POS	AMB	POS	POS
**CC30-MSSA**																		
2012_155_Z3488 (25905A/11)	AMB	POS	NEG	AMB	POS	AMB	POS	NEG	NEG	NEG	POS	NEG	POS	AMB	POS	AMB	NEG	NEG
**CC30-MSSA [PVL+]**																		
2012_164_Z3447 (26574/11)	AMB	POS	AMB	POS	POS	AMB	POS	NEG	POS	NEG	POS	NEG	POS	AMB	POS	AMB	NEG	NEG
2012_166_Z3493 (26988A/11)	NEG	POS	NEG	AMB	POS	AMB	POS	NEG	NEG	NEG	POS	NEG	POS	AMB	AMB	POS	NEG	NEG
2012_178_Z3500 (197/12)	AMB	POS	NEG	NEG	POS	NEG	POS	NEG	POS	NEG	POS	NEG	POS	NEG	POS	AMB	NEG	NEG
2012_183_Z3454	AMB	POS	NEG	AMB	POS	AMB	POS	NEG	POS	NEG	POS	NEG	POS	AMB	POS	AMB	NEG	NEG
2012_186_Z3503 (444/12)	AMB	POS	NEG	NEG	POS	NEG	POS	NEG	NEG	NEG	POS	NEG	AMB	NEG	POS	AMB	NEG	NEG
2012_189_Z3506 (542/12)	AMB	POS	NEG	AMB	POS	NEG	POS	NEG	POS	NEG	POS	NEG	POS	AMB	POS	AMB	NEG	NEG
2012_192_Z3457 (606/12)	AMB	POS	NEG	NEG	POS	NEG	POS	NEG	POS	NEG	POS	NEG	POS	AMB	POS	AMB	NEG	NEG
2012_196_Z3509 (707/12)	AMB	POS	NEG	AMB	POS	NEG	POS	NEG	POS	NEG	POS	NEG	POS	AMB	POS	AMB	NEG	NEG
2012_218_Z3476 (1432/12)	AMB	POS	NEG	NEG	POS	NEG	POS	NEG	NEG	NEG	POS	NEG	POS	NEG	POS	AMB	NEG	NEG
2012_232_Z3520 (1892/12)	AMB	POS	NEG	POS	POS	AMB	POS	NEG	POS	NEG	POS	NEG	POS	AMB	POS	AMB	NEG	NEG
2012_278_Z3565 (4397/12)	NEG	POS	NEG	AMB	POS	NEG	POS	NEG	POS	NEG	POS	NEG	POS	AMB	POS	AMB	NEG	NEG
2012_285_Z3572 (4802/12)	AMB	POS	NEG	NEG	POS	NEG	POS	NEG	NEG	NEG	POS	NEG	POS	AMB	POS	AMB	NEG	NEG
2012_287_Z3574 (4906/12)	AMB	POS	NEG	NEG	POS	NEG	POS	NEG	POS	NEG	POS	NEG	POS	AMB	POS	AMB	NEG	NEG
2012_295_Z3582 (5116/12)	AMB	POS	NEG	NEG	POS	NEG	POS	NEG	NEG	NEG	POS	NEG	POS	NEG	POS	AMB	NEG	NEG
2012_326_Z3613 (7321/12)	AMB	POS	NEG	POS	POS	AMB	POS	NEG	NEG	NEG	POS	NEG	POS	AMB	POS	AMB	NEG	AMB
2012_356_Z3643 (1204-15897)	AMB	POS	NEG	NEG	POS	NEG	POS	NEG	NEG	NEG	POS	NEG	AMB	AMB	POS	AMB	NEG	NEG
2012_366_Z3426 (1204-16124)	AMB	POS	NEG	POS	POS	AMB	POS	NEG	POS	NEG	POS	NEG	POS	AMB	POS	AMB	NEG	NEG
2012_372_Z3432 (1205-19821)	NEG	POS	NEG	POS	POS	AMB	POS	NEG	POS	NEG	POS	NEG	POS	AMB	POS	AMB	NEG	NEG
2012_380_Z3440 (4141)	AMB	POS	NEG	POS	POS	AMB	POS	NEG	POS	NEG	POS	NEG	POS	AMB	POS	AMB	NEG	NEG
2013_PA20_Z4003	NEG	POS	NEG	NEG	POS	NEG	POS	NEG	NEG	NEG	POS	NEG	NEG	NEG	POS	AMB	NEG	NEG
2013_PA36_Z4019	NEG	POS	NEG	NEG	POS	NEG	POS	NEG	NEG	NEG	POS	NEG	NEG	NEG	POS	NEG	NEG	NEG
2013_PA46_Z4029	NEG	POS	NEG	NEG	AMB	NEG	POS	NEG	NEG	NEG	POS	NEG	NEG	NEG	POS	NEG	NEG	NEG
2013_PA50_Z4033	NEG	POS	NEG	NEG	POS	NEG	POS	NEG	NEG	NEG	POS	NEG	NEG	NEG	POS	NEG	NEG	NEG
2013_PA63_Z4046	NEG	POS	NEG	NEG	POS	NEG	POS	NEG	NEG	NEG	POS	NEG	NEG	NEG	POS	NEG	NEG	NEG
2013_PA78_Z4061	NEG	POS	NEG	NEG	POS	NEG	POS	NEG	NEG	NEG	POS	NEG	AMB	NEG	POS	AMB	NEG	NEG
2013_PA79_Z4062	NEG	POS	NEG	AMB	POS	NEG	POS	NEG	NEG	NEG	POS	NEG	POS	AMB	POS	AMB	NEG	NEG
**CC45-MSSA**																		
2012_172_Z3449 (49/12)	POS	AMB	AMB	NEG	NEG	NEG	POS	NEG	NEG	NEG	POS	NEG	POS	NEG	POS	AMB	NEG	NEG
2012_208_Z3468 (1011/12)	AMB	POS	NEG	NEG	NEG	NEG	POS	NEG	NEG	NEG	POS	NEG	POS	NEG	AMB	POS	NEG	NEG
2012_235_Z3522 (2085/12)	AMB	POS	NEG	NEG	NEG	NEG	POS	NEG	NEG	NEG	POS	NEG	POS	NEG	AMB	POS	NEG	NEG
2012_290_Z3577 (4921/12)	AMB	POS	NEG	NEG	NEG	NEG	AMB	NEG	NEG	NEG	POS	NEG	POS	NEG	NEG	POS	NEG	NEG
2012_311_Z3598 (6182/12)	NEG	POS	NEG	NEG	NEG	NEG	POS	NEG	NEG	NEG	POS	NEG	POS	NEG	AMB	POS	NEG	NEG
Z3604_TT317 - {DC558C18-2959-4049-B8F3-507365	AMB	POS	NEG	NEG	NEG	NEG	POS	NEG	NEG	NEG	POS	NEG	POS	NEG	AMB	POS	NEG	NEG
2012_363_Z3423 (1112-61058)	AMB	POS	NEG	NEG	NEG	NEG	POS	NEG	NEG	NEG	POS	NEG	POS	NEG	NEG	NEG	NEG	NEG
2013_PA19_Z4002	AMB	POS	NEG	NEG	NEG	NEG	POS	NEG	NEG	NEG	POS	NEG	AMB	NEG	AMB	POS	NEG	NEG
2013_PA54_Z4037	AMB	POS	NEG	NEG	NEG	NEG	POS	NEG	NEG	NEG	POS	NEG	POS	NEG	AMB	POS	NEG	NEG
2013_PA82_Z4065	NEG	POS	NEG	NEG	NEG	NEG	POS	NEG	NEG	NEG	POS	NEG	AMB	NEG	AMB	POS	NEG	NEG
**CC97-MSSA**																		
2012_182_Z3453 (279A/12)	AMB	POS	POS	POS	NEG	POS	AMB	POS	AMB	POS	AMB	NEG	POS	AMB	POS	AMB	POS	POS
2012_187_Z3504	POS	AMB	POS	AMB	NEG	POS	NEG	POS	AMB	POS	NEG	NEG	AMB	AMB	POS	AMB	POS	POS
2012_197_Z3510 (757/12)	POS	AMB	POS	POS	NEG	POS	NEG	POS	AMB	POS	NEG	NEG	POS	AMB	POS	AMB	POS	POS
2012_199_Z3516 (785/12)	AMB	POS	POS	POS	NEG	POS	AMB	POS	AMB	POS	AMB	NEG	POS	AMB	POS	AMB	POS	POS
2013_PA15_Z3998	POS	AMB	POS	POS	NEG	POS	AMB	POS	AMB	POS	NEG	NEG	POS	AMB	POS	AMB	POS	POS
**CC101-MSSA**																		
2012_283_Z3570 (4725/12)	POS	NEG	POS	POS	NEG	POS	NEG	NEG	AMB	POS	NEG	NEG	NEG	AMB	POS	AMB	POS	POS
2012_247_Z3531 (2788/12)	POS	NEG	POS	POS	NEG	POS	NEG	NEG	AMB	POS	NEG	NEG	NEG	AMB	POS	AMB	POS	POS
2012_279_Z3566 (4506/12)	POS	NEG	POS	POS	NEG	POS	AMB	NEG	AMB	POS	NEG	NEG	NEG	AMB	POS	AMB	POS	POS

Strain / Isolates	glutamylendopeptidase	staphopain B, protease	staphopain A (staphylopain A), protease		staphyl. exotoxin-like protein	staphylococcal superantigen-like protein 1												staphylococcal superantigen-like protein 2		staphylococcal superantigen-like protein 3			staphylococcal superantigen-like protein 4		staphylococcal superantigen-like protein 5				staphylococcal superantigen-like protein 6		staphylococcal superantigen-like protein 7			staphylococcal superantigen-like protein 8	
	sspA	sspB	sspP (consensus)	sspP (other than ST93)	setC	ssl01/set6_probe1_11	ssl01/set6_probe2_11	ssl01/set6_probe1_12	ssl01/set6_probe2_12	ssl01/set6_probe4_11	ssl01/set6_probeRF122	ssl01/set6 (COL)	ssl01/set6 (Mu50+N315)	ssl01/set6 (MW2+MSSA476)	ssl01/set6 (MRSA252)	ssl01/set6 (RF122)	ssl01/set6 (other alleles)	ssl02/set7	ssl02/set7 (MRSA252)	ssl03/set8_probe 1	ssl03/set8_probe 2	ssl03/set8 (MRSA252, SAR0424)	ssl04/set9	ssl04/set9 (MRSA252, SAR0425)	ssl05/set3_probe 1	ssl05/set3 (RF122, probe-611)	ssl05/set3_probe 2 (612)	ssl05/set3 (MRSA252)	ssl06/set21	ssl06 (NCTC8325+MW2)	ssl07/set1	ssl07/set1 (MRSA252)	ssl07/set1 (AF188836)	ssl08/set12_probe 1	ssl08/set12_probe 2
**CC121-MSSA**																																			
2012_163_Z3446 (26495/11)	POS	POS	POS	POS	POS	POS	NEG	POS	NEG	POS	NEG	POS	AMB	NEG	NEG	NEG	NEG	POS	NEG	POS	POS	NEG	POS	AMB	NEG	AMB	POS	NEG	POS	POS	AMB	POS	AMB	POS	POS
2012_204_Z3464 (938/12)	POS	POS	POS	POS	POS	POS	NEG	POS	NEG	POS	NEG	NEG	POS	NEG	NEG	NEG	NEG	POS	NEG	POS	POS	NEG	POS	POS	NEG	AMB	POS	POS	POS	POS	POS	AMB	NEG	POS	POS
2012_225_Z3482 (1563/12)	POS	POS	POS	POS	POS	POS	NEG	POS	AMB	POS	NEG	POS	AMB	NEG	NEG	NEG	NEG	POS	NEG	POS	POS	NEG	POS	POS	NEG	AMB	POS	AMB	POS	POS	AMB	POS	AMB	POS	POS
2012_228_Z3485 (1700B/12)	POS	POS	POS	POS	POS	POS	NEG	POS	NEG	POS	NEG	POS	AMB	NEG	NEG	NEG	NEG	POS	NEG	POS	POS	NEG	POS	AMB	NEG	AMB	POS	NEG	POS	POS	AMB	POS	AMB	POS	POS
2012_229_Z3486 (1737/12)	POS	POS	POS	POS	POS	POS	NEG	POS	NEG	POS	NEG	POS	AMB	NEG	NEG	NEG	NEG	POS	NEG	POS	POS	NEG	POS	AMB	NEG	AMB	POS	NEG	POS	POS	AMB	POS	AMB	POS	POS
2012_302_Z3589 (5795/12)	POS	POS	POS	POS	POS	POS	NEG	POS	NEG	POS	NEG	POS	AMB	NEG	NEG	NEG	NEG	POS	NEG	POS	POS	NEG	POS	AMB	NEG	AMB	POS	NEG	POS	POS	POS	AMB	NEG	POS	POS
2012_359_Z3419	POS	POS	POS	POS	POS	POS	NEG	POS	NEG	POS	NEG	POS	AMB	NEG	NEG	NEG	NEG	POS	NEG	POS	POS	NEG	POS	NEG	NEG	AMB	POS	NEG	POS	POS	POS	AMB	NEG	POS	POS
2012_362_Z3422 (1201-996)	POS	POS	POS	POS	POS	POS	NEG	POS	NEG	POS	NEG	POS	AMB	NEG	NEG	NEG	NEG	POS	NEG	POS	POS	NEG	POS	AMB	NEG	AMB	POS	AMB	POS	POS	POS	AMB	NEG	POS	POS
2012_367_Z3427 (1204-16413)	POS	POS	POS	POS	POS	POS	NEG	POS	NEG	POS	NEG	NEG	POS	NEG	NEG	NEG	NEG	POS	NEG	POS	POS	NEG	POS	AMB	NEG	AMB	POS	NEG	POS	POS	AMB	POS	NEG	POS	POS
2013_PA09_Z3992	POS	POS	POS	POS	POS	POS	NEG	POS	NEG	POS	NEG	POS	AMB	NEG	NEG	NEG	NEG	POS	NEG	POS	POS	NEG	POS	AMB	NEG	AMB	POS	NEG	POS	POS	POS	AMB	NEG	POS	POS
2013_PA59_Z4042	POS	POS	POS	POS	POS	POS	NEG	POS	NEG	POS	NEG	POS	AMB	NEG	NEG	NEG	NEG	POS	NEG	POS	POS	NEG	POS	AMB	NEG	AMB	POS	NEG	POS	POS	POS	AMB	NEG	POS	POS
2013_PA60_Z4043	POS	POS	POS	POS	POS	POS	NEG	POS	NEG	POS	NEG	NEG	POS	NEG	NEG	NEG	NEG	POS	NEG	POS	POS	NEG	POS	NEG	NEG	POS	AMB	NEG	POS	POS	POS	NEG	NEG	POS	POS
**CC152-MSSA [PVL+]**																																			
2012_190_Z3507 (549/12)	POS	POS	POS	POS	NEG	NEG	NEG	NEG	POS	NEG	POS	NEG	NEG	NEG	NEG	POS	NEG	AMB	POS	NEG	NEG	NEG	NEG	NEG	NEG	NEG	NEG	NEG	NEG	NEG	NEG	POS	AMB	NEG	NEG
2012_201_Z3461 (823/12)	POS	POS	POS	POS	NEG	NEG	NEG	NEG	POS	NEG	POS	NEG	NEG	NEG	NEG	POS	NEG	AMB	POS	NEG	NEG	NEG	NEG	NEG	NEG	NEG	NEG	NEG	NEG	NEG	NEG	POS	AMB	NEG	NEG
2012_209_Z3469 (1116/12)	POS	POS	POS	POS	NEG	NEG	NEG	NEG	POS	NEG	POS	NEG	NEG	NEG	NEG	POS	NEG	AMB	POS	NEG	NEG	NEG	NEG	NEG	NEG	NEG	NEG	NEG	NEG	NEG	NEG	POS	AMB	NEG	NEG
2012_211_Z3517 (1238/12)	POS	POS	POS	POS	NEG	NEG	NEG	NEG	POS	NEG	POS	NEG	NEG	NEG	NEG	POS	NEG	AMB	POS	NEG	NEG	NEG	NEG	NEG	NEG	NEG	NEG	NEG	NEG	NEG	NEG	NEG	NEG	NEG	NEG
2012_213_Z3472 (1320/12)	POS	POS	POS	POS	NEG	NEG	NEG	NEG	POS	NEG	POS	NEG	NEG	NEG	NEG	POS	NEG	AMB	POS	NEG	NEG	NEG	NEG	NEG	NEG	NEG	NEG	NEG	NEG	NEG	NEG	NEG	NEG	NEG	NEG
2012_221_Z3478	POS	POS	POS	POS	NEG	NEG	NEG	NEG	POS	NEG	POS	NEG	NEG	NEG	NEG	POS	NEG	AMB	POS	NEG	NEG	NEG	NEG	NEG	NEG	NEG	NEG	NEG	NEG	NEG	NEG	POS	AMB	NEG	NEG
2012_234_Z3521 (1981/12)	POS	POS	POS	POS	NEG	NEG	NEG	NEG	POS	NEG	POS	NEG	NEG	NEG	NEG	POS	NEG	AMB	POS	NEG	NEG	NEG	NEG	NEG	NEG	NEG	NEG	NEG	NEG	NEG	NEG	NEG	NEG	NEG	NEG
2012_238_Z3524 (2199/12)	POS	POS	POS	POS	NEG	NEG	NEG	NEG	POS	NEG	POS	NEG	NEG	NEG	NEG	POS	NEG	AMB	POS	NEG	NEG	NEG	NEG	NEG	NEG	NEG	NEG	NEG	NEG	NEG	NEG	NEG	NEG	NEG	NEG
2012_260_Z3547 (3290/12)	POS	POS	POS	POS	NEG	NEG	NEG	NEG	POS	NEG	POS	NEG	NEG	NEG	NEG	POS	NEG	AMB	POS	NEG	NEG	NEG	NEG	NEG	NEG	NEG	NEG	NEG	AMB	NEG	NEG	POS	AMB	NEG	NEG
2012_272_Z3559 (3802/12)	POS	POS	POS	POS	NEG	NEG	NEG	NEG	POS	NEG	POS	NEG	NEG	NEG	NEG	POS	NEG	AMB	POS	NEG	NEG	NEG	NEG	NEG	NEG	NEG	NEG	NEG	NEG	NEG	NEG	POS	NEG	NEG	NEG
2012_289_Z3576 (4912/12)	POS	POS	POS	POS	NEG	NEG	NEG	NEG	POS	NEG	POS	NEG	NEG	NEG	NEG	POS	NEG	AMB	POS	NEG	NEG	NEG	NEG	NEG	NEG	NEG	NEG	NEG	NEG	NEG	NEG	POS	NEG	NEG	NEG
2012_309_Z3596	POS	POS	POS	POS	NEG	NEG	NEG	NEG	POS	NEG	POS	NEG	NEG	NEG	NEG	POS	NEG	AMB	POS	NEG	NEG	NEG	NEG	NEG	NEG	NEG	NEG	NEG	NEG	NEG	NEG	POS	NEG	NEG	NEG
2012_322_Z3609 (7178/12)	POS	POS	POS	POS	NEG	NEG	NEG	NEG	POS	NEG	POS	NEG	NEG	NEG	NEG	POS	NEG	AMB	POS	NEG	NEG	NEG	NEG	NEG	NEG	NEG	NEG	NEG	NEG	NEG	NEG	NEG	NEG	NEG	NEG
2012_325_Z3612 (7254/12)	POS	POS	POS	POS	NEG	NEG	NEG	NEG	POS	NEG	POS	NEG	NEG	NEG	NEG	POS	NEG	AMB	POS	NEG	NEG	NEG	NEG	NEG	NEG	NEG	NEG	NEG	NEG	NEG	NEG	POS	AMB	NEG	NEG
2012_328_Z3615 (7456/12)	POS	POS	POS	POS	NEG	NEG	NEG	NEG	POS	NEG	POS	NEG	NEG	NEG	NEG	POS	NEG	AMB	POS	NEG	NEG	NEG	NEG	NEG	NEG	NEG	NEG	NEG	NEG	NEG	NEG	POS	AMB	NEG	NEG
2012_334_Z3621 (8267/12)	POS	POS	POS	POS	NEG	NEG	NEG	NEG	POS	NEG	POS	NEG	NEG	NEG	NEG	POS	NEG	AMB	POS	NEG	NEG	NEG	NEG	NEG	NEG	NEG	NEG	NEG	NEG	NEG	NEG	POS	AMB	NEG	NEG
2012_360_Z3420 (1201-4047)	POS	POS	POS	POS	NEG	NEG	NEG	NEG	POS	NEG	POS	NEG	NEG	NEG	NEG	POS	NEG	AMB	POS	NEG	NEG	NEG	NEG	NEG	NEG	NEG	NEG	NEG	NEG	NEG	NEG	POS	NEG	NEG	NEG
2012_382_Z3442 (4086)	POS	POS	POS	POS	NEG	NEG	NEG	NEG	POS	NEG	POS	NEG	NEG	NEG	NEG	POS	NEG	AMB	POS	NEG	NEG	NEG	NEG	NEG	NEG	NEG	NEG	NEG	NEG	NEG	NEG	NEG	NEG	NEG	NEG
2013_PA02_Z3985	POS	POS	POS	POS	NEG	NEG	NEG	NEG	POS	NEG	POS	NEG	NEG	NEG	NEG	POS	NEG	AMB	POS	NEG	NEG	NEG	NEG	NEG	NEG	NEG	NEG	NEG	NEG	NEG	NEG	NEG	NEG	NEG	NEG
2013_PA03_Z3986	POS	POS	POS	POS	NEG	NEG	NEG	NEG	POS	NEG	POS	NEG	NEG	NEG	NEG	POS	NEG	AMB	POS	NEG	NEG	NEG	NEG	NEG	NEG	NEG	NEG	NEG	NEG	NEG	NEG	NEG	NEG	NEG	NEG
2013_PA07_Z3990	POS	POS	POS	POS	NEG	NEG	NEG	NEG	POS	NEG	POS	NEG	NEG	NEG	NEG	POS	NEG	AMB	POS	NEG	NEG	NEG	NEG	NEG	NEG	NEG	NEG	NEG	NEG	NEG	NEG	NEG	NEG	NEG	NEG
2013_PA34_Z4017	POS	POS	POS	POS	NEG	NEG	NEG	NEG	POS	NEG	POS	NEG	NEG	NEG	NEG	POS	NEG	AMB	POS	NEG	NEG	NEG	NEG	NEG	NEG	NEG	NEG	NEG	NEG	NEG	NEG	NEG	NEG	NEG	NEG
2013_PA40_Z4023	POS	POS	POS	POS	NEG	NEG	NEG	NEG	POS	NEG	POS	NEG	NEG	NEG	NEG	POS	NEG	AMB	POS	NEG	NEG	NEG	NEG	NEG	NEG	NEG	NEG	NEG	NEG	NEG	NEG	NEG	NEG	NEG	NEG
2013_PA48_Z4031	POS	POS	POS	POS	NEG	NEG	NEG	NEG	POS	NEG	POS	NEG	NEG	NEG	NEG	POS	NEG	AMB	POS	NEG	NEG	NEG	NEG	NEG	NEG	NEG	NEG	NEG	NEG	NEG	NEG	NEG	NEG	NEG	NEG
2013_PA49_Z4032	POS	POS	POS	POS	NEG	NEG	NEG	NEG	POS	NEG	POS	NEG	NEG	NEG	NEG	POS	NEG	AMB	POS	NEG	NEG	NEG	NEG	NEG	NEG	NEG	NEG	NEG	NEG	NEG	NEG	NEG	NEG	NEG	NEG
2013_PA51_Z4034	POS	POS	POS	POS	NEG	NEG	NEG	NEG	POS	NEG	POS	NEG	NEG	NEG	NEG	POS	NEG	AMB	POS	NEG	NEG	NEG	NEG	NEG	NEG	NEG	NEG	NEG	NEG	NEG	NEG	NEG	NEG	NEG	NEG
2013_PA70_Z4053	POS	POS	POS	POS	NEG	NEG	NEG	NEG	POS	NEG	POS	NEG	NEG	NEG	NEG	POS	NEG	AMB	POS	NEG	NEG	NEG	NEG	NEG	NEG	NEG	NEG	NEG	NEG	NEG	NEG	NEG	NEG	NEG	NEG
2013_PA84_Z4067	POS	POS	POS	POS	NEG	NEG	NEG	NEG	POS	NEG	POS	NEG	NEG	NEG	NEG	POS	NEG	AMB	POS	NEG	NEG	NEG	NEG	NEG	NEG	NEG	NEG	NEG	NEG	NEG	NEG	NEG	NEG	NEG	NEG
**CC188-MSSA**																																			
2012_206_Z3466 (968/12)	POS	POS	POS	POS	POS	NEG	POS	NEG	POS	NEG	NEG	NEG	NEG	POS	NEG	NEG	NEG	AMB	POS	POS	POS	NEG	POS	AMB	POS	POS	AMB	NEG	POS	POS	AMB	POS	AMB	POS	POS
2012_251_Z3534 (3026/12)	POS	POS	POS	POS	POS	NEG	POS	NEG	POS	NEG	NEG	NEG	NEG	POS	NEG	NEG	NEG	POS	AMB	POS	POS	NEG	POS	NEG	POS	POS	AMB	NEG	POS	POS	AMB	POS	AMB	POS	POS
2012_320_Z3607 (7144/12)	POS	POS	POS	POS	POS	NEG	POS	NEG	POS	NEG	NEG	NEG	NEG	POS	NEG	NEG	NEG	POS	AMB	POS	POS	NEG	POS	NEG	POS	AMB	POS	NEG	POS	POS	AMB	POS	AMB	POS	POS
2012_321_Z3608 (7147/12)	POS	AMB	POS	POS	POS	NEG	POS	NEG	POS	NEG	NEG	NEG	NEG	POS	NEG	NEG	NEG	POS	AMB	POS	POS	NEG	POS	NEG	POS	POS	AMB	NEG	POS	POS	AMB	POS	AMB	POS	POS
2012_358_Z3418 (1202-6332)	POS	POS	POS	POS	POS	NEG	POS	AMB	POS	NEG	NEG	NEG	NEG	POS	NEG	NEG	NEG	POS	AMB	POS	POS	NEG	POS	AMB	POS	POS	AMB	NEG	POS	POS	AMB	POS	AMB	POS	POS
**CC398-MSSA**																																			
2013_PA11_Z3994	POS	POS	POS	POS	POS	POS	NEG	POS	NEG	POS	NEG	NEG	POS	NEG	NEG	NEG	NEG	AMB	POS	NEG	NEG	NEG	NEG	POS	NEG	NEG	NEG	POS	NEG	AMB	AMB	POS	AMB	NEG	NEG
2013_PA57_Z4040	POS	POS	POS	POS	POS	POS	NEG	POS	NEG	POS	NEG	NEG	POS	NEG	NEG	NEG	NEG	AMB	POS	NEG	NEG	NEG	NEG	POS	NEG	NEG	NEG	POS	NEG	NEG	AMB	POS	AMB	NEG	NEG
**ST1852-MSSA**																																			
2012_277_Z3564 (4050/12)	POS	POS	POS	POS	POS	NEG	NEG	NEG	POS	NEG	POS	NEG	NEG	NEG	NEG	POS	NEG	AMB	POS	NEG	POS	POS	NEG	POS	NEG	POS	NEG	POS	NEG	POS	AMB	POS	AMB	NEG	NEG
**CC1223/1594-MSSA**																																			
2012_337_Z3624 (8070B/12)	NEG	NEG	NEG	NEG	POS	NEG	AMB	POS	NEG	POS	POS	NEG	NEG	NEG	NEG	NEG	POS	NEG	NEG	NEG	NEG	NEG	NEG	AMB	NEG	NEG	NEG	NEG	NEG	POS	POS	NEG	NEG	POS	POS
**MSSA related to ST2250/2277**																																			
2012_203_Z3463 (829/12)	AMB	POS	NEG	AMB	AMB	NEG	NEG	AMB	NEG	POS	AMB	NEG	NEG	NEG	NEG	NEG	POS	POS	NEG	NEG	NEG	NEG	POS	POS	NEG	POS	NEG	NEG	NEG	NEG	AMB	POS	AMB	AMB	AMB
2012_269_Z3556 (3769A/12)	NEG	NEG	NEG	NEG	NEG	NEG	NEG	NEG	NEG	POS	NEG	NEG	NEG	NEG	NEG	NEG	POS	NEG	NEG	NEG	NEG	NEG	AMB	POS	NEG	NEG	NEG	NEG	NEG	NEG	AMB	POS	AMB	NEG	NEG
2012_292_Z3579	NEG	NEG	NEG	NEG	NEG	NEG	NEG	NEG	NEG	NEG	NEG	NEG	NEG	NEG	NEG	NEG	NEG	NEG	NEG	NEG	NEG	NEG	NEG	AMB	NEG	NEG	NEG	NEG	NEG	NEG	AMB	POS	AMB	NEG	NEG
2012_299_Z3586 (5487/12)	NEG	NEG	NEG	NEG	NEG	NEG	NEG	NEG	NEG	AMB	NEG	NEG	NEG	NEG	NEG	NEG	NEG	NEG	NEG	NEG	NEG	NEG	AMB	AMB	NEG	NEG	NEG	NEG	NEG	NEG	AMB	POS	AMB	NEG	NEG
2012_301_Z3588 (5717/12)	NEG	NEG	NEG	NEG	NEG	NEG	NEG	NEG	NEG	AMB	NEG	NEG	NEG	NEG	NEG	NEG	NEG	NEG	NEG	NEG	NEG	NEG	AMB	AMB	NEG	NEG	NEG	NEG	NEG	NEG	AMB	POS	AMB	NEG	NEG
**ST2596dlv-MSSA**																																			
2012_310_Z3597 (6034/12)	NEG	NEG	NEG	NEG	POS	NEG	POS	NEG	NEG	AMB	POS	NEG	NEG	NEG	NEG	NEG	POS	NEG	NEG	NEG	NEG	NEG	AMB	AMB	NEG	NEG	NEG	NEG	NEG	POS	POS	NEG	NEG	AMB	POS

Strain / Isolates	staphylococcal superantigen-like protein 9			staphylococcal superantigen-like protein 10			staphylococcal superantigene-like protein 11				staphylococcal exotoxin-like protein, second locus					Capsule type 1	capsular poly-saccharide synthesis enzyme	O-antigen poly-merase	capsular poly-saccharide biosyn-thesis protein	Capsule type 5	capsular poly-saccharide synthesis enzyme	O-antigen poly-merase	capsular poly-saccharide biosyn-thesis protein	Capsule type 8	capsular poly-saccharide synthesis enzyme	capsular poly-saccharide biosyn-thesis protein	O-antigen poly-merase	capsular poly-saccharide biosyn-thesis protein	intercellular adhesion protein A	intercellular adhesion protein C	biofilm PIA synthesis protein D	surface protein involved in biofilm formation
	ssl09/set5_probe 1	ssl09/set5_probe 2	ssl09/set5 (MRSA252)	ssl10/set4	ssl10 (RF122)	ssl10/set4 (MRSA252)	ssl11/set2 (COL)	ssl11+set2(Mu50+N315)	ssl11+set2(MW2+MSSA476)	ssl11/set2 (MRSA252)	setB3	setB3 (MRSA252)	setB2	setB2 (MRSA252)	setB1	cap 1 (total)	capH1	capJ1	capK1	cap 5 (total)	capH5	capJ5	capK5	cap 8 (total)	capH8	capI8	capJ8	capK8	icaA	icaC	icaD	bap
**CC1-MSSA**																																
2012_257_Z3544 (3216/12)	POS	POS	AMB	POS	AMB	AMB	NEG	NEG	POS	NEG	POS	NEG	POS	NEG	POS	NEG	NEG	NEG	NEG	NEG	NEG	NEG	NEG	POS	POS	POS	POS	POS	POS	POS	POS	NEG
2012_281_Z3568 (4696/12)	POS	POS	NEG	POS	AMB	AMB	NEG	NEG	POS	NEG	POS	NEG	POS	NEG	POS	NEG	NEG	NEG	NEG	NEG	NEG	NEG	NEG	POS	POS	POS	POS	POS	POS	POS	POS	NEG
2012_324_Z3611 (7180/12)	POS	POS	NEG	AMB	AMB	POS	NEG	NEG	POS	NEG	POS	NEG	POS	NEG	POS	NEG	NEG	NEG	NEG	NEG	NEG	NEG	NEG	POS	POS	POS	POS	POS	POS	POS	POS	NEG
2012_327_Z3614 (7347/12)	POS	POS	AMB	AMB	AMB	POS	NEG	NEG	POS	NEG	POS	NEG	POS	NEG	POS	NEG	NEG	NEG	NEG	NEG	NEG	NEG	NEG	POS	POS	POS	POS	POS	POS	POS	POS	NEG
2012_351_Z3638 (8273B/12)	POS	POS	NEG	POS	AMB	AMB	NEG	NEG	POS	NEG	POS	NEG	POS	NEG	POS	NEG	AMB	NEG	NEG	NEG	NEG	NEG	NEG	POS	POS	POS	POS	POS	POS	POS	POS	NEG
**CC5-MSSA**																																
2012_188_Z3505 (503/12)	POS	POS	NEG	POS	NEG	AMB	NEG	POS	NEG	NEG	POS	NEG	POS	NEG	POS	NEG	NEG	NEG	NEG	POS	POS	POS	POS	NEG	NEG	NEG	NEG	NEG	POS	POS	POS	NEG
2012_219_Z3518 (1437/12)	POS	POS	NEG	POS	AMB	AMB	NEG	POS	NEG	NEG	POS	NEG	POS	NEG	POS	NEG	NEG	NEG	NEG	POS	POS	POS	POS	NEG	NEG	NEG	NEG	NEG	POS	POS	POS	NEG
2012_329_Z3616 (7577/12)	POS	POS	AMB	POS	AMB	AMB	NEG	POS	NEG	NEG	POS	NEG	POS	NEG	POS	NEG	NEG	NEG	NEG	POS	POS	POS	POS	NEG	NEG	NEG	NEG	NEG	POS	POS	POS	NEG
2012_377_Z3437 (6242)	POS	POS	AMB	POS	AMB	AMB	NEG	POS	NEG	NEG	POS	NEG	POS	NEG	POS	NEG	NEG	AMB	NEG	POS	POS	POS	POS	NEG	NEG	NEG	NEG	NEG	POS	POS	POS	NEG
2013_PA04_Z3987	POS	POS	NEG	POS	AMB	AMB	NEG	POS	NEG	NEG	POS	NEG	POS	NEG	POS	NEG	NEG	NEG	NEG	POS	POS	POS	POS	NEG	NEG	NEG	NEG	NEG	POS	POS	POS	NEG
2013_PA32_Z4015	POS	POS	NEG	POS	AMB	AMB	NEG	POS	NEG	NEG	POS	NEG	POS	NEG	POS	NEG	NEG	NEG	NEG	POS	POS	POS	POS	NEG	NEG	NEG	NEG	NEG	POS	POS	POS	NEG
2013_PA41_Z4024	POS	POS	NEG	POS	NEG	AMB	NEG	POS	NEG	NEG	POS	NEG	POS	NEG	POS	NEG	NEG	NEG	NEG	POS	POS	POS	POS	NEG	NEG	NEG	NEG	NEG	POS	POS	POS	NEG
2013_PA44_Z4027	POS	POS	NEG	POS	NEG	AMB	NEG	POS	NEG	NEG	POS	NEG	POS	NEG	POS	NEG	NEG	NEG	NEG	POS	POS	POS	POS	NEG	NEG	NEG	NEG	NEG	POS	POS	POS	NEG
**CC5-MSSA [PVL+]**																																
2012_175_Z3497 (112/12)	POS	POS	NEG	POS	NEG	AMB	NEG	POS	NEG	NEG	POS	NEG	POS	NEG	POS	NEG	NEG	NEG	NEG	POS	POS	POS	POS	NEG	NEG	NEG	NEG	NEG	POS	POS	POS	NEG
2012_193_Z3458 (617/12)	POS	POS	NEG	POS	AMB	AMB	NEG	POS	NEG	NEG	POS	NEG	POS	NEG	POS	NEG	NEG	AMB	NEG	POS	POS	POS	POS	NEG	NEG	NEG	NEG	NEG	POS	POS	POS	NEG
2012_215_Z3474 (1341/12)	POS	POS	NEG	POS	NEG	AMB	NEG	POS	NEG	NEG	POS	NEG	POS	NEG	POS	NEG	NEG	NEG	NEG	POS	POS	POS	POS	NEG	NEG	NEG	NEG	NEG	POS	POS	POS	NEG
2012_216_Z3475 (1370/12)	POS	POS	NEG	POS	NEG	AMB	NEG	POS	NEG	NEG	POS	NEG	POS	NEG	POS	NEG	NEG	NEG	NEG	POS	POS	POS	POS	NEG	NEG	NEG	NEG	NEG	POS	POS	POS	NEG
2012_297_Z3584 (5140/12)	POS	POS	NEG	POS	NEG	AMB	NEG	POS	NEG	NEG	POS	NEG	POS	NEG	POS	NEG	NEG	NEG	NEG	POS	POS	POS	POS	NEG	NEG	NEG	NEG	NEG	POS	POS	POS	NEG
2012_331_Z3618 (7644/12)	POS	POS	NEG	POS	AMB	AMB	NEG	POS	NEG	NEG	POS	NEG	POS	NEG	POS	NEG	NEG	AMB	NEG	POS	POS	POS	POS	NEG	NEG	NEG	NEG	NEG	POS	POS	POS	NEG
2012_379_Z3439 (4126)	POS	POS	NEG	POS	NEG	AMB	NEG	POS	NEG	NEG	POS	NEG	POS	NEG	POS	NEG	NEG	NEG	NEG	POS	POS	POS	POS	NEG	NEG	NEG	NEG	NEG	POS	POS	POS	NEG
2012_381_Z3441	POS	POS	AMB	POS	AMB	AMB	NEG	POS	NEG	NEG	POS	NEG	POS	NEG	POS	NEG	NEG	AMB	NEG	POS	POS	POS	POS	NEG	NEG	NEG	NEG	NEG	POS	POS	POS	NEG
2013_PA39_Z4022	POS	POS	NEG	POS	NEG	AMB	NEG	POS	NEG	NEG	POS	NEG	POS	NEG	POS	NEG	NEG	NEG	NEG	POS	POS	POS	POS	NEG	NEG	NEG	NEG	NEG	POS	POS	POS	NEG
2013_PA47_Z4030	POS	POS	NEG	POS	NEG	AMB	NEG	POS	NEG	NEG	POS	NEG	POS	NEG	POS	NEG	NEG	NEG	NEG	POS	POS	POS	POS	NEG	NEG	NEG	NEG	NEG	POS	POS	POS	NEG
2013_PA61_I_Z4044	POS	POS	NEG	POS	AMB	AMB	NEG	POS	NEG	NEG	POS	NEG	POS	NEG	POS	NEG	NEG	NEG	NEG	POS	POS	POS	POS	NEG	NEG	NEG	NEG	NEG	POS	POS	POS	NEG
2013_PA62_I_Z4045	POS	POS	NEG	POS	NEG	AMB	NEG	POS	NEG	NEG	POS	NEG	POS	NEG	POS	NEG	NEG	NEG	NEG	POS	POS	POS	POS	NEG	NEG	NEG	NEG	NEG	POS	POS	POS	NEG
2013_PA74_Z4057	POS	POS	NEG	POS	NEG	AMB	NEG	POS	NEG	NEG	POS	NEG	POS	NEG	POS	NEG	NEG	NEG	NEG	POS	POS	POS	POS	NEG	NEG	NEG	NEG	NEG	POS	POS	POS	NEG
2013_PA75_Z4058	POS	POS	NEG	POS	NEG	AMB	NEG	POS	NEG	NEG	POS	NEG	POS	NEG	POS	NEG	NEG	NEG	NEG	POS	POS	POS	POS	NEG	NEG	NEG	NEG	NEG	POS	POS	POS	NEG
**CC5-MRSA-V [ACME+]**																																
2012_373_Z3433 (1204-17312)	POS	POS	AMB	POS	AMB	AMB	NEG	POS	NEG	NEG	POS	NEG	POS	NEG	POS	NEG	NEG	AMB	NEG	POS	POS	POS	POS	NEG	NEG	NEG	NEG	NEG	POS	POS	POS	NEG
**CC6-MSSA**																																
2012_173_Z3450 (98A/12)	POS	POS	POS	AMB	POS	POS	NEG	POS	AMB	NEG	POS	NEG	POS	NEG	POS	NEG	NEG	NEG	AMB	NEG	NEG	NEG	NEG	POS	POS	POS	POS	POS	POS	POS	POS	NEG
2012_176_Z3498	POS	POS	NEG	POS	AMB	AMB	NEG	POS	NEG	NEG	POS	NEG	POS	NEG	POS	NEG	NEG	NEG	NEG	NEG	NEG	NEG	NEG	POS	POS	POS	POS	POS	POS	POS	POS	NEG
2012_181_Z3452	POS	POS	AMB	POS	AMB	AMB	NEG	POS	NEG	NEG	POS	NEG	POS	NEG	POS	NEG	NEG	NEG	NEG	NEG	NEG	NEG	NEG	POS	POS	POS	POS	POS	POS	POS	POS	NEG
2012_259_Z3546 (3279/12)	POS	POS	AMB	POS	AMB	AMB	NEG	POS	NEG	NEG	POS	NEG	POS	NEG	POS	NEG	NEG	NEG	AMB	NEG	NEG	NEG	NEG	POS	POS	POS	POS	POS	POS	POS	POS	NEG
2012_264_Z3551 (3407B/12)	POS	POS	AMB	POS	AMB	AMB	NEG	POS	NEG	NEG	POS	NEG	POS	NEG	POS	NEG	NEG	NEG	NEG	NEG	NEG	NEG	NEG	POS	POS	POS	POS	POS	POS	POS	POS	NEG
2012_273_Z3560 (3844/12)	POS	POS	NEG	POS	AMB	AMB	NEG	POS	NEG	NEG	POS	NEG	POS	NEG	POS	AMB	AMB	NEG	AMB	NEG	NEG	NEG	NEG	POS	POS	POS	POS	POS	POS	POS	POS	NEG
2012_312_Z3599 (6815/12)	POS	POS	NEG	POS	AMB	AMB	NEG	POS	NEG	NEG	POS	NEG	POS	NEG	POS	NEG	NEG	NEG	NEG	NEG	NEG	NEG	NEG	POS	POS	POS	POS	POS	POS	POS	POS	NEG
2012_338_Z3625	POS	POS	NEG	POS	AMB	AMB	NEG	POS	NEG	NEG	POS	NEG	POS	NEG	POS	NEG	NEG	NEG	NEG	NEG	NEG	NEG	NEG	POS	POS	POS	POS	POS	POS	POS	POS	NEG
2012_352_Z3639	POS	POS	NEG	POS	AMB	AMB	NEG	POS	NEG	NEG	POS	NEG	POS	NEG	POS	NEG	NEG	NEG	NEG	NEG	NEG	NEG	NEG	POS	POS	POS	POS	POS	POS	POS	POS	NEG
2012_368_Z3428	POS	POS	NEG	POS	AMB	AMB	NEG	POS	NEG	NEG	POS	NEG	POS	NEG	POS	NEG	NEG	NEG	NEG	NEG	NEG	NEG	NEG	POS	POS	POS	POS	POS	POS	POS	POS	NEG
2013_PA12_Z3995	POS	POS	NEG	POS	AMB	AMB	NEG	POS	NEG	NEG	POS	NEG	POS	NEG	POS	NEG	NEG	NEG	NEG	NEG	NEG	NEG	NEG	POS	POS	POS	POS	POS	POS	POS	POS	NEG
2013_PA28_Z4011	POS	POS	NEG	POS	AMB	AMB	NEG	POS	NEG	NEG	POS	NEG	POS	NEG	POS	NEG	NEG	NEG	NEG	NEG	NEG	NEG	NEG	POS	POS	POS	POS	POS	POS	POS	POS	NEG
**CC6-MSSA [PVL+]**																																
2012_375_Z3435	POS	POS	AMB	POS	AMB	AMB	NEG	POS	NEG	NEG	POS	NEG	POS	NEG	POS	NEG	AMB	NEG	NEG	NEG	NEG	NEG	NEG	POS	POS	POS	POS	POS	POS	POS	POS	NEG
2013_PA77_Z4060	POS	POS	NEG	POS	AMB	AMB	NEG	POS	NEG	NEG	POS	NEG	POS	NEG	POS	NEG	NEG	NEG	NEG	NEG	NEG	NEG	NEG	POS	POS	POS	POS	POS	POS	POS	POS	NEG
**CC7-MSSA**																																
2012_248_Z3532 (2864/12)	POS	POS	NEG	POS	AMB	AMB	NEG	NEG	NEG	NEG	POS	NEG	POS	NEG	POS	AMB	AMB	NEG	AMB	NEG	NEG	NEG	NEG	POS	POS	POS	POS	POS	POS	POS	POS	NEG
2012_265_Z3552 (3518/12)	POS	POS	NEG	POS	AMB	AMB	NEG	POS	NEG	NEG	POS	NEG	POS	NEG	POS	NEG	AMB	NEG	NEG	NEG	NEG	NEG	NEG	POS	POS	POS	POS	POS	POS	POS	POS	NEG
2013_PA53_Z4036	POS	POS	NEG	POS	AMB	AMB	NEG	NEG	NEG	NEG	POS	NEG	POS	NEG	POS	NEG	NEG	NEG	NEG	NEG	NEG	NEG	NEG	POS	POS	POS	POS	POS	POS	POS	POS	NEG
2013_PA83_Z4066	POS	POS	NEG	POS	AMB	AMB	NEG	NEG	NEG	NEG	POS	NEG	POS	NEG	POS	NEG	NEG	NEG	NEG	NEG	NEG	NEG	NEG	POS	POS	POS	POS	POS	POS	POS	POS	NEG
**CC8-MSSA**																																
2012_243_Z3528 (2587/12)	POS	POS	NEG	POS	NEG	AMB	POS	NEG	NEG	NEG	POS	NEG	POS	NEG	POS	NEG	NEG	NEG	NEG	POS	POS	POS	POS	NEG	NEG	NEG	NEG	NEG	POS	POS	POS	NEG
2012_344_Z3631 (8605/12)	POS	POS	NEG	POS	AMB	AMB	POS	NEG	NEG	NEG	POS	NEG	POS	NEG	POS	NEG	NEG	NEG	NEG	POS	POS	POS	POS	NEG	NEG	NEG	NEG	NEG	POS	POS	POS	NEG
2012_346_Z3633 (8074/12)	POS	POS	NEG	POS	NEG	AMB	POS	NEG	NEG	NEG	POS	NEG	POS	NEG	POS	NEG	NEG	NEG	NEG	POS	POS	POS	POS	NEG	NEG	NEG	NEG	NEG	POS	POS	POS	NEG
2012_255_Z3538 (3205B/12)	POS	POS	NEG	POS	NEG	AMB	POS	NEG	NEG	NEG	POS	NEG	POS	NEG	POS	NEG	NEG	NEG	NEG	POS	POS	POS	POS	NEG	NEG	NEG	NEG	NEG	POS	POS	POS	NEG
2012_280_Z3567 (4601/12)	POS	POS	NEG	POS	AMB	AMB	POS	NEG	NEG	NEG	POS	NEG	POS	NEG	POS	NEG	NEG	NEG	NEG	POS	POS	POS	POS	NEG	NEG	NEG	NEG	NEG	POS	POS	POS	NEG
Trinidad&Tobago_2012_267 (3642/12)	POS	POS	NEG	POS	NEG	AMB	POS	NEG	NEG	NEG	POS	NEG	POS	NEG	POS	NEG	NEG	NEG	NEG	POS	POS	POS	POS	NEG	NEG	NEG	NEG	NEG	POS	POS	POS	NEG
2012_365_Z3425 (1204-15838)	POS	POS	NEG	AMB	AMB	POS	POS	NEG	NEG	NEG	POS	NEG	POS	NEG	POS	NEG	NEG	AMB	NEG	POS	POS	POS	POS	NEG	NEG	NEG	NEG	NEG	POS	POS	POS	NEG
2012_296_Z3583 (5118/12)	POS	POS	NEG	POS	AMB	AMB	POS	NEG	NEG	NEG	POS	NEG	POS	NEG	POS	NEG	NEG	NEG	NEG	POS	POS	POS	POS	NEG	NEG	NEG	NEG	NEG	POS	POS	POS	NEG
2012_318_Z3605 (6960/12)	POS	AMB	NEG	POS	NEG	AMB	POS	NEG	NEG	NEG	POS	NEG	POS	NEG	POS	NEG	NEG	NEG	NEG	POS	POS	POS	POS	NEG	NEG	NEG	NEG	NEG	POS	POS	POS	NEG
2012_306_Z3593 (5861/12)	POS	POS	NEG	POS	AMB	AMB	POS	NEG	NEG	NEG	POS	NEG	POS	NEG	POS	NEG	NEG	AMB	NEG	POS	POS	POS	POS	NEG	NEG	NEG	NEG	NEG	POS	POS	POS	NEG
2013_PA37_Z4020	POS	POS	NEG	POS	AMB	AMB	POS	NEG	NEG	NEG	POS	NEG	POS	NEG	POS	NEG	NEG	NEG	NEG	POS	POS	POS	POS	NEG	NEG	NEG	NEG	NEG	POS	POS	POS	NEG
2013_PA38_Z4021	POS	POS	NEG	POS	NEG	NEG	POS	NEG	NEG	NEG	POS	NEG	POS	NEG	POS	NEG	NEG	NEG	NEG	POS	POS	POS	POS	NEG	NEG	NEG	NEG	NEG	POS	POS	POS	NEG
2013_PA52_Z4035	POS	POS	NEG	POS	NEG	NEG	POS	NEG	NEG	NEG	POS	NEG	POS	NEG	POS	NEG	NEG	NEG	NEG	POS	POS	POS	POS	NEG	NEG	NEG	NEG	NEG	POS	POS	POS	NEG
**CC8-MSSA [PVL+]**																																
2012_156_Z3489 (25917/11)	POS	POS	NEG	POS	AMB	AMB	POS	NEG	NEG	NEG	POS	NEG	POS	NEG	POS	NEG	NEG	NEG	NEG	POS	POS	POS	POS	NEG	NEG	NEG	NEG	NEG	POS	POS	POS	NEG
2012_159_Z3492 (26310/11)	POS	POS	NEG	POS	AMB	AMB	POS	NEG	NEG	NEG	POS	NEG	POS	NEG	POS	NEG	NEG	NEG	NEG	POS	POS	POS	POS	NEG	NEG	NEG	NEG	NEG	POS	POS	POS	NEG
2012_161_Z3445 (26430/11)	POS	POS	AMB	AMB	AMB	POS	POS	NEG	NEG	NEG	POS	NEG	POS	NEG	POS	AMB	AMB	AMB	NEG	POS	POS	POS	POS	NEG	NEG	NEG	NEG	NEG	POS	POS	POS	NEG
2012_162_Z3512 (26433/11)	POS	POS	NEG	POS	AMB	AMB	POS	NEG	NEG	NEG	POS	NEG	POS	NEG	POS	NEG	NEG	NEG	NEG	POS	POS	POS	POS	NEG	NEG	NEG	NEG	NEG	POS	POS	POS	NEG
2012_169_Z3495 (27194/11)	POS	POS	NEG	POS	AMB	AMB	POS	NEG	NEG	NEG	POS	NEG	POS	NEG	POS	NEG	NEG	NEG	NEG	POS	POS	POS	POS	NEG	NEG	NEG	NEG	NEG	POS	POS	POS	NEG
2012_170_Z3496 (35/12)	POS	POS	NEG	POS	AMB	AMB	POS	NEG	NEG	NEG	POS	NEG	POS	NEG	POS	NEG	NEG	NEG	NEG	POS	POS	POS	POS	NEG	NEG	NEG	NEG	NEG	POS	POS	POS	NEG
2012_171_Z3448 (47/12)	POS	POS	NEG	POS	AMB	AMB	POS	NEG	NEG	NEG	POS	NEG	POS	NEG	POS	NEG	NEG	NEG	NEG	POS	POS	POS	POS	NEG	NEG	NEG	NEG	NEG	POS	POS	POS	NEG
2012_177_Z3499 (184/12)	POS	POS	NEG	POS	AMB	AMB	POS	NEG	NEG	NEG	POS	NEG	POS	NEG	POS	NEG	NEG	NEG	NEG	POS	POS	POS	POS	NEG	NEG	NEG	NEG	NEG	POS	POS	POS	NEG
2012_179_Z3515 (231/12)	POS	POS	NEG	POS	AMB	AMB	POS	NEG	NEG	NEG	POS	NEG	POS	NEG	POS	AMB	AMB	AMB	NEG	POS	POS	POS	POS	NEG	NEG	NEG	NEG	NEG	POS	POS	POS	NEG
2012_185_Z3502 (437/12)	POS	POS	NEG	POS	NEG	AMB	POS	NEG	NEG	NEG	POS	NEG	POS	NEG	POS	NEG	NEG	NEG	NEG	POS	POS	POS	POS	NEG	NEG	NEG	NEG	NEG	POS	POS	POS	NEG
2012_191_Z3456 (563/12)	POS	POS	NEG	POS	NEG	AMB	POS	NEG	NEG	NEG	POS	NEG	POS	NEG	POS	NEG	NEG	AMB	NEG	POS	POS	POS	POS	NEG	NEG	NEG	NEG	NEG	POS	POS	POS	NEG
2012_194_Z3459	POS	POS	NEG	POS	NEG	AMB	POS	NEG	NEG	NEG	POS	NEG	POS	NEG	POS	NEG	NEG	AMB	NEG	POS	POS	POS	POS	NEG	NEG	NEG	NEG	NEG	POS	POS	POS	NEG
2012_200_Z3460 (820/12)	POS	POS	NEG	AMB	POS	AMB	POS	NEG	NEG	NEG	POS	NEG	POS	NEG	POS	NEG	AMB	NEG	NEG	POS	POS	POS	POS	NEG	NEG	NEG	NEG	NEG	POS	POS	POS	NEG
2012_207_Z3467 (1010/12)	POS	POS	NEG	POS	NEG	AMB	POS	NEG	NEG	NEG	POS	NEG	POS	NEG	POS	NEG	AMB	AMB	NEG	POS	POS	POS	POS	NEG	NEG	NEG	NEG	NEG	POS	POS	POS	NEG
2012_212_Z3471 (1261/12)	POS	POS	NEG	POS	NEG	AMB	POS	NEG	NEG	NEG	POS	NEG	POS	NEG	POS	NEG	NEG	NEG	NEG	POS	POS	POS	POS	NEG	NEG	NEG	NEG	NEG	POS	POS	POS	NEG
2012_220_Z3477 (1466/12)	POS	POS	NEG	POS	NEG	AMB	POS	NEG	NEG	NEG	POS	NEG	POS	NEG	POS	NEG	NEG	NEG	NEG	POS	POS	POS	POS	NEG	NEG	NEG	NEG	NEG	POS	POS	POS	NEG
2012_224_Z3481 (1522/12)	POS	POS	NEG	POS	NEG	AMB	POS	NEG	NEG	NEG	POS	NEG	POS	NEG	POS	NEG	NEG	NEG	NEG	POS	POS	POS	POS	NEG	NEG	NEG	NEG	NEG	POS	POS	POS	NEG
2012_226_Z3483 (1662/12)	POS	POS	NEG	POS	AMB	AMB	POS	NEG	NEG	NEG	POS	NEG	POS	NEG	POS	NEG	NEG	NEG	NEG	POS	POS	POS	POS	NEG	NEG	NEG	NEG	NEG	POS	POS	POS	NEG
2012_242_Z3527 (2581/12)	POS	POS	NEG	POS	AMB	AMB	POS	NEG	NEG	NEG	POS	NEG	POS	NEG	POS	NEG	NEG	AMB	NEG	POS	POS	POS	POS	NEG	NEG	NEG	NEG	NEG	POS	POS	POS	NEG
2012_245_Z3529 (2687/12)	POS	POS	NEG	POS	AMB	AMB	POS	NEG	NEG	NEG	POS	NEG	POS	NEG	POS	NEG	NEG	AMB	NEG	POS	POS	POS	POS	NEG	NEG	NEG	NEG	NEG	POS	POS	POS	NEG
2012_253_Z3536 (3074/12)	POS	POS	NEG	POS	AMB	AMB	POS	NEG	NEG	NEG	POS	NEG	POS	NEG	POS	AMB	AMB	AMB	NEG	POS	POS	POS	POS	NEG	NEG	NEG	NEG	NEG	POS	POS	POS	NEG
2012_258_Z3545 (3231/12)	POS	POS	NEG	POS	AMB	AMB	POS	NEG	NEG	NEG	POS	NEG	POS	NEG	POS	NEG	AMB	AMB	NEG	POS	POS	POS	POS	NEG	NEG	NEG	NEG	NEG	POS	POS	POS	NEG
2012_261_Z3548 (3298/12)	POS	POS	NEG	POS	AMB	AMB	POS	NEG	NEG	NEG	POS	NEG	POS	NEG	POS	NEG	NEG	NEG	NEG	POS	POS	POS	POS	NEG	NEG	NEG	NEG	NEG	POS	POS	POS	NEG
2012_270_Z3557 (3798/12)	POS	POS	NEG	POS	AMB	AMB	POS	NEG	NEG	POS	POS	NEG	POS	NEG	POS	AMB	AMB	AMB	NEG	POS	POS	POS	POS	NEG	NEG	NEG	NEG	NEG	POS	POS	POS	NEG
2012_274_Z3561 (3861/12)	POS	POS	NEG	POS	AMB	AMB	POS	NEG	NEG	NEG	POS	NEG	POS	NEG	POS	NEG	NEG	NEG	NEG	POS	POS	POS	POS	NEG	NEG	NEG	NEG	NEG	POS	POS	POS	NEG
2012_282_Z3569 (4698/12)	POS	POS	NEG	POS	NEG	AMB	POS	NEG	NEG	NEG	POS	NEG	POS	NEG	POS	NEG	NEG	NEG	NEG	POS	POS	POS	POS	NEG	NEG	NEG	NEG	NEG	POS	POS	POS	NEG
2012_286_Z3573 (4805/12)	POS	POS	NEG	POS	NEG	AMB	POS	NEG	NEG	NEG	POS	NEG	POS	NEG	POS	NEG	NEG	NEG	NEG	POS	POS	POS	POS	NEG	NEG	NEG	NEG	NEG	POS	POS	POS	NEG
2012_293_Z3580 (5007/12)	POS	POS	NEG	POS	NEG	AMB	POS	NEG	NEG	NEG	POS	NEG	POS	NEG	POS	NEG	NEG	NEG	NEG	POS	POS	POS	POS	NEG	NEG	NEG	NEG	NEG	POS	POS	POS	NEG
2012_294_Z3581	POS	POS	NEG	POS	NEG	AMB	POS	NEG	NEG	NEG	POS	NEG	POS	NEG	POS	NEG	NEG	NEG	NEG	POS	POS	POS	POS	NEG	NEG	NEG	NEG	NEG	POS	POS	POS	NEG
2012_307_Z3594 (5918/12)	POS	POS	NEG	POS	AMB	AMB	POS	NEG	NEG	NEG	POS	NEG	POS	NEG	POS	NEG	AMB	NEG	NEG	POS	POS	POS	POS	NEG	NEG	NEG	NEG	NEG	POS	POS	POS	NEG
2012_333_Z3620 (8101/12)	POS	POS	NEG	POS	AMB	AMB	POS	NEG	NEG	NEG	POS	NEG	POS	NEG	POS	NEG	NEG	NEG	NEG	POS	POS	POS	POS	NEG	NEG	NEG	NEG	NEG	POS	POS	POS	NEG
2012_343_Z3630 (8195/12)	POS	POS	NEG	POS	AMB	AMB	POS	NEG	NEG	NEG	POS	NEG	POS	NEG	POS	NEG	NEG	NEG	NEG	POS	POS	POS	POS	NEG	NEG	NEG	NEG	NEG	POS	POS	POS	NEG
2012_349_Z3636 (7999A/12)	POS	POS	NEG	POS	AMB	AMB	POS	NEG	NEG	NEG	POS	NEG	POS	NEG	POS	AMB	AMB	AMB	NEG	POS	POS	POS	POS	NEG	NEG	NEG	AMB	NEG	POS	POS	POS	NEG
2012_353_Z3640 (8575/12)	POS	POS	NEG	POS	AMB	AMB	POS	NEG	NEG	NEG	POS	NEG	POS	NEG	POS	NEG	NEG	AMB	NEG	POS	POS	POS	POS	NEG	NEG	NEG	NEG	NEG	POS	POS	POS	NEG
2012_354_Z3641 (8408/12)	POS	POS	NEG	POS	AMB	AMB	POS	NEG	AMB	NEG	POS	NEG	POS	NEG	POS	AMB	AMB	AMB	NEG	POS	POS	POS	POS	NEG	NEG	NEG	NEG	NEG	POS	POS	POS	NEG
2012_355_Z3642 (1112-58348)	POS	POS	NEG	POS	NEG	AMB	POS	NEG	NEG	NEG	POS	NEG	POS	NEG	POS	NEG	NEG	NEG	NEG	POS	POS	POS	POS	NEG	NEG	NEG	NEG	NEG	POS	POS	POS	NEG
2012_357_Z3417 (1202-6360)	POS	POS	NEG	AMB	AMB	POS	POS	NEG	NEG	NEG	POS	NEG	POS	NEG	POS	NEG	NEG	AMB	NEG	POS	POS	POS	POS	NEG	NEG	NEG	NEG	NEG	POS	POS	POS	NEG
2012_364_Z3424 (1112-60417)	POS	POS	NEG	POS	AMB	AMB	POS	NEG	NEG	NEG	POS	NEG	POS	NEG	POS	NEG	NEG	AMB	NEG	POS	POS	POS	POS	NEG	NEG	NEG	NEG	NEG	POS	POS	POS	NEG
2012_376_Z3436 (6256)	POS	POS	AMB	AMB	AMB	POS	POS	NEG	NEG	NEG	POS	NEG	POS	NEG	POS	AMB	AMB	POS	NEG	POS	POS	POS	POS	NEG	NEG	NEG	NEG	NEG	POS	POS	POS	NEG
2012_383_Z3443	POS	POS	NEG	AMB	AMB	POS	POS	NEG	NEG	NEG	POS	NEG	POS	NEG	POS	NEG	NEG	NEG	NEG	POS	POS	POS	POS	NEG	NEG	NEG	NEG	NEG	POS	POS	POS	NEG
2013_PA01_Z3984	POS	POS	NEG	POS	AMB	AMB	POS	NEG	NEG	NEG	POS	NEG	POS	NEG	POS	NEG	NEG	NEG	NEG	POS	POS	POS	POS	NEG	NEG	NEG	NEG	NEG	POS	POS	POS	NEG
2013_PA08_Z3991	POS	POS	NEG	POS	AMB	AMB	POS	NEG	NEG	NEG	POS	NEG	POS	NEG	POS	NEG	NEG	NEG	NEG	POS	POS	POS	POS	NEG	NEG	NEG	NEG	NEG	POS	POS	POS	NEG
2013_PA10_Z3993	POS	POS	NEG	POS	AMB	AMB	POS	NEG	NEG	NEG	POS	NEG	POS	NEG	POS	NEG	NEG	NEG	NEG	POS	POS	POS	POS	NEG	NEG	NEG	NEG	NEG	POS	POS	POS	NEG
2013_PA13_Z3996	POS	POS	NEG	POS	AMB	AMB	POS	NEG	NEG	NEG	POS	NEG	POS	NEG	POS	NEG	NEG	NEG	NEG	POS	POS	POS	POS	NEG	NEG	NEG	NEG	NEG	POS	POS	POS	NEG
2013_PA14_Z3997	POS	POS	NEG	POS	AMB	AMB	POS	NEG	NEG	NEG	POS	NEG	POS	NEG	POS	NEG	NEG	NEG	NEG	POS	POS	POS	POS	NEG	NEG	NEG	NEG	NEG	POS	POS	POS	NEG
2013_PA17_Z4000	POS	POS	NEG	POS	AMB	AMB	POS	NEG	NEG	NEG	POS	NEG	POS	NEG	POS	NEG	NEG	NEG	NEG	POS	POS	POS	POS	NEG	NEG	NEG	NEG	NEG	POS	POS	POS	NEG
2013_PA26_Z4009	POS	POS	NEG	POS	NEG	AMB	POS	NEG	NEG	NEG	POS	NEG	POS	NEG	POS	NEG	NEG	NEG	NEG	POS	POS	POS	POS	NEG	NEG	NEG	NEG	NEG	POS	POS	POS	NEG
2013_PA27_Z4010	POS	POS	NEG	POS	AMB	AMB	POS	NEG	NEG	NEG	POS	NEG	POS	NEG	POS	NEG	NEG	NEG	NEG	POS	POS	POS	POS	NEG	NEG	NEG	NEG	NEG	POS	POS	POS	NEG
2013_PA42_Z4025	POS	POS	NEG	POS	AMB	AMB	POS	NEG	NEG	NEG	POS	NEG	POS	NEG	POS	NEG	NEG	NEG	NEG	POS	POS	POS	POS	NEG	NEG	NEG	NEG	NEG	POS	POS	POS	NEG
2013_PA43_Z4026	POS	POS	NEG	POS	NEG	AMB	POS	NEG	NEG	NEG	POS	NEG	POS	NEG	POS	NEG	NEG	NEG	NEG	POS	POS	POS	POS	NEG	NEG	NEG	NEG	NEG	POS	POS	POS	NEG
2013_PA45_Z4028	POS	POS	NEG	POS	AMB	AMB	POS	NEG	NEG	NEG	POS	NEG	POS	NEG	POS	NEG	NEG	NEG	NEG	POS	POS	POS	POS	NEG	NEG	NEG	NEG	NEG	POS	POS	POS	NEG
2013_PA56_Z4039	POS	POS	NEG	POS	AMB	AMB	POS	NEG	NEG	NEG	POS	NEG	POS	NEG	POS	NEG	NEG	NEG	NEG	POS	POS	POS	POS	NEG	NEG	NEG	NEG	NEG	POS	POS	POS	NEG
2013_PA58_Z4041	POS	POS	NEG	POS	AMB	AMB	POS	NEG	NEG	NEG	POS	NEG	POS	NEG	POS	NEG	NEG	NEG	NEG	POS	POS	POS	POS	NEG	NEG	NEG	NEG	NEG	POS	POS	POS	NEG
2013_PA64_Z4047	POS	POS	NEG	POS	AMB	AMB	POS	NEG	NEG	NEG	POS	NEG	POS	NEG	POS	NEG	NEG	NEG	NEG	POS	POS	POS	POS	NEG	NEG	NEG	NEG	NEG	POS	POS	POS	NEG
2013_PA66_Z4049	POS	POS	NEG	POS	AMB	AMB	POS	NEG	NEG	NEG	POS	NEG	POS	NEG	POS	NEG	NEG	NEG	NEG	POS	POS	POS	POS	NEG	NEG	NEG	NEG	NEG	POS	POS	POS	NEG
2013_PA67_Z4050	POS	POS	NEG	POS	AMB	AMB	POS	NEG	NEG	NEG	POS	NEG	POS	NEG	POS	NEG	NEG	NEG	NEG	POS	POS	POS	POS	NEG	NEG	NEG	NEG	NEG	POS	POS	POS	NEG
2013_PA69_Z4052	POS	POS	NEG	POS	AMB	AMB	POS	NEG	NEG	NEG	POS	NEG	POS	NEG	POS	NEG	NEG	NEG	NEG	POS	POS	POS	POS	NEG	NEG	NEG	NEG	NEG	POS	POS	POS	NEG
2013_PA72_Z4055	POS	POS	NEG	POS	AMB	AMB	POS	NEG	NEG	NEG	POS	NEG	POS	NEG	POS	NEG	NEG	NEG	NEG	POS	POS	POS	POS	NEG	NEG	NEG	NEG	NEG	POS	POS	POS	NEG
2013_PA76_Z4059	POS	POS	NEG	POS	AMB	AMB	POS	NEG	NEG	NEG	POS	NEG	POS	NEG	POS	NEG	NEG	NEG	NEG	POS	POS	POS	POS	NEG	NEG	NEG	NEG	NEG	POS	POS	POS	NEG
2013_PA80_Z4063	POS	POS	NEG	POS	AMB	AMB	POS	NEG	NEG	NEG	POS	NEG	POS	NEG	POS	NEG	NEG	NEG	NEG	POS	POS	POS	POS	NEG	NEG	NEG	NEG	NEG	POS	POS	POS	NEG

Strain / Isolates	staphylococcal superantigen-like protein 9			staphylococcal superantigen-like protein 10			staphylococcal superantigene-like protein 11				staphylococcal exotoxin-like protein, second locus					Capsule type 1	capsular poly-saccharide synthesis enzyme	O-antigen poly-merase	capsular poly-saccharide biosyn-thesis protein	Capsule type 5	capsular poly-saccharide synthesis enzyme	O-antigen poly-merase	capsular poly-saccharide biosyn-thesis protein	Capsule type 8	capsular poly-saccharide synthesis enzyme	capsular poly-saccharide biosyn-thesis protein	O-antigen poly-merase	capsular poly-saccharide biosyn-thesis protein	intercellular adhesion protein A	intercellular adhesion protein C	biofilm PIA synthesis protein D	surface protein involved in biofilm formation
	ssl09/set5_probe 1	ssl09/set5_probe 2	ssl09/set5 (MRSA252)	ssl10/set4	ssl10 (RF122)	ssl10/set4 (MRSA252)	ssl11/set2 (COL)	ssl11+set2(Mu50+N315)	ssl11+set2(MW2+MSSA 476)	ssl11/set2 (MRSA252)	setB3	setB3 (MRSA252)	setB2	setB2 (MRSA252)	setB1	cap 1 (total)	capH1	capJ1	capK1	cap 5 (total)	capH5	capJ5	capK5	cap 8 (total)	capH8	capI8	capJ8	capK8	icaA	icaC	icaD	bap
**ST8-MRSA-IV, USA300**																																
2012_158_Z3491 (26049A/11)	POS	POS	NEG	POS	AMB	AMB	POS	NEG	NEG	NEG	POS	NEG	POS	NEG	POS	NEG	NEG	#ZAHL!	NEG	POS	POS	POS	POS	NEG	NEG	NEG	NEG	NEG	POS	POS	POS	NEG
2012_165_Z3513 (26656/11)	POS	POS	NEG	POS	AMB	AMB	POS	NEG	NEG	NEG	POS	NEG	POS	NEG	POS	NEG	NEG	NEG	NEG	POS	POS	POS	POS	NEG	NEG	NEG	NEG	NEG	POS	POS	POS	NEG
2012_214_Z3473 (1322/12)	POS	POS	NEG	POS	NEG	AMB	POS	NEG	NEG	NEG	POS	NEG	POS	NEG	POS	NEG	NEG	NEG	NEG	POS	POS	POS	POS	NEG	NEG	NEG	NEG	NEG	POS	POS	POS	NEG
2012_227_Z3484 (1688/12)	POS	POS	NEG	POS	AMB	AMB	POS	NEG	NEG	NEG	POS	NEG	POS	NEG	POS	NEG	NEG	NEG	NEG	POS	POS	POS	POS	NEG	NEG	NEG	NEG	NEG	POS	POS	POS	NEG
2012_263_Z3550 (3355/12)	POS	POS	NEG	POS	AMB	AMB	POS	NEG	NEG	NEG	POS	NEG	POS	NEG	POS	NEG	NEG	NEG	NEG	POS	POS	POS	POS	NEG	NEG	NEG	NEG	NEG	POS	POS	POS	NEG
2012_266_Z3553	POS	POS	NEG	POS	AMB	AMB	POS	NEG	NEG	NEG	POS	NEG	POS	NEG	POS	NEG	NEG	AMB	NEG	POS	POS	POS	POS	NEG	NEG	NEG	NEG	NEG	POS	POS	POS	NEG
2012_284_Z3571 (4729/12)	POS	POS	NEG	POS	AMB	AMB	POS	NEG	NEG	NEG	POS	NEG	POS	NEG	POS	NEG	NEG	NEG	NEG	POS	POS	POS	POS	NEG	NEG	NEG	NEG	NEG	POS	POS	POS	NEG
2012_298_Z3585 (5203/12)	POS	POS	NEG	POS	NEG	AMB	POS	NEG	NEG	NEG	POS	NEG	POS	NEG	POS	NEG	NEG	NEG	NEG	POS	POS	POS	POS	NEG	NEG	NEG	NEG	NEG	POS	POS	POS	NEG
2012_304_Z3591 (5818/12)	POS	POS	NEG	POS	NEG	AMB	POS	NEG	NEG	NEG	POS	NEG	POS	NEG	POS	NEG	NEG	NEG	NEG	POS	POS	POS	POS	NEG	NEG	NEG	NEG	NEG	POS	POS	POS	NEG
Z3595_TT308 - {EFC74B77-3925-484F-A4CF-A1F7DC	POS	POS	NEG	POS	NEG	AMB	POS	NEG	NEG	NEG	POS	NEG	POS	NEG	POS	NEG	NEG	NEG	NEG	POS	POS	POS	POS	NEG	NEG	NEG	NEG	NEG	POS	POS	POS	NEG
2012_369_Z3429 (1112-58547)	POS	POS	NEG	POS	AMB	AMB	POS	NEG	NEG	NEG	POS	NEG	POS	NEG	POS	NEG	NEG	NEG	NEG	POS	POS	POS	POS	NEG	NEG	AMB	AMB	NEG	POS	POS	POS	NEG
2012_374_Z3434 (1203-13527)	POS	POS	NEG	POS	AMB	AMB	POS	NEG	NEG	NEG	POS	NEG	POS	NEG	POS	NEG	NEG	AMB	NEG	POS	POS	POS	POS	NEG	NEG	NEG	NEG	NEG	POS	POS	POS	NEG
2013_PA06_Z3989	POS	POS	NEG	POS	AMB	AMB	POS	NEG	NEG	NEG	POS	NEG	POS	NEG	POS	NEG	NEG	NEG	NEG	POS	POS	POS	POS	NEG	NEG	NEG	NEG	NEG	POS	POS	POS	NEG
2013_PA55_Z4038	POS	POS	NEG	POS	AMB	AMB	POS	NEG	NEG	NEG	POS	NEG	POS	NEG	POS	NEG	NEG	NEG	NEG	POS	POS	POS	POS	NEG	NEG	NEG	NEG	NEG	POS	POS	POS	NEG
2013_PA71_Z4054	POS	POS	NEG	POS	NEG	AMB	POS	NEG	NEG	NEG	POS	NEG	POS	NEG	POS	NEG	NEG	NEG	NEG	POS	POS	POS	POS	NEG	NEG	NEG	NEG	NEG	POS	POS	POS	NEG
**ST8-MRSA-IV [PVL+/ACME+], US**																																
2012_180_Z3501 (232/12)	POS	POS	NEG	POS	NEG	AMB	POS	NEG	NEG	NEG	POS	NEG	POS	NEG	POS	NEG	NEG	NEG	NEG	POS	POS	POS	POS	NEG	NEG	NEG	NEG	NEG	POS	POS	POS	NEG
2013_PA68_Z4051	POS	POS	NEG	POS	AMB	AMB	POS	NEG	NEG	NEG	POS	NEG	POS	NEG	POS	NEG	NEG	NEG	NEG	POS	POS	POS	POS	NEG	NEG	NEG	NEG	NEG	POS	POS	POS	NEG
**ST72-MSSA**																																
2012_174_Z3451 (102/12)	POS	POS	AMB	POS	AMB	AMB	NEG	NEG	NEG	NEG	POS	NEG	POS	NEG	POS	NEG	NEG	NEG	NEG	POS	POS	POS	POS	NEG	NEG	NEG	NEG	NEG	POS	POS	POS	NEG
2012_223_Z3480 (1506A/12)	POS	POS	NEG	POS	NEG	AMB	NEG	NEG	NEG	NEG	POS	NEG	POS	NEG	POS	NEG	NEG	NEG	NEG	POS	POS	POS	POS	NEG	NEG	NEG	NEG	NEG	POS	POS	POS	NEG
2012_236_Z3523 (2086/12)	POS	POS	NEG	POS	AMB	AMB	NEG	NEG	NEG	NEG	POS	NEG	POS	NEG	POS	NEG	NEG	NEG	NEG	POS	POS	POS	POS	NEG	NEG	NEG	NEG	NEG	POS	POS	POS	NEG
2012_239_Z3525 (2346/12)	POS	POS	NEG	POS	AMB	AMB	NEG	NEG	NEG	NEG	POS	NEG	POS	NEG	POS	NEG	NEG	AMB	NEG	POS	POS	POS	POS	NEG	NEG	NEG	NEG	NEG	POS	POS	POS	NEG
2012_252_Z3535 (3030/12)	POS	POS	AMB	POS	AMB	AMB	NEG	NEG	NEG	NEG	POS	NEG	POS	NEG	POS	NEG	NEG	POS	NEG	POS	POS	POS	POS	NEG	NEG	NEG	NEG	NEG	POS	POS	POS	NEG
2012_271_Z3558 (3799/12)	POS	POS	NEG	POS	AMB	AMB	NEG	NEG	NEG	NEG	POS	NEG	POS	NEG	POS	NEG	NEG	AMB	NEG	POS	POS	POS	POS	NEG	NEG	NEG	NEG	NEG	POS	POS	POS	NEG
2012_276_Z3563	POS	POS	NEG	POS	AMB	AMB	NEG	NEG	NEG	NEG	POS	NEG	POS	NEG	POS	NEG	NEG	NEG	NEG	POS	POS	POS	POS	NEG	NEG	NEG	NEG	NEG	POS	POS	POS	NEG
2012_300_Z3587 (5638/12)	POS	POS	NEG	POS	NEG	AMB	NEG	NEG	NEG	NEG	POS	NEG	POS	NEG	POS	NEG	NEG	NEG	NEG	POS	POS	POS	POS	NEG	NEG	NEG	NEG	NEG	POS	POS	POS	NEG
2012_316_Z3603 (6941/12)	POS	POS	NEG	POS	AMB	AMB	NEG	NEG	NEG	NEG	POS	NEG	POS	NEG	POS	NEG	NEG	NEG	NEG	POS	POS	POS	POS	NEG	NEG	NEG	NEG	NEG	POS	POS	POS	NEG
2012_319_Z3606 (7019/12)	POS	POS	NEG	POS	AMB	AMB	NEG	NEG	NEG	NEG	POS	NEG	POS	NEG	POS	NEG	NEG	NEG	NEG	POS	POS	POS	POS	NEG	NEG	NEG	NEG	NEG	POS	POS	POS	NEG
2012_371_Z3431 (1204-17577)	POS	POS	AMB	POS	AMB	AMB	NEG	NEG	NEG	NEG	POS	NEG	POS	NEG	POS	NEG	NEG	NEG	NEG	POS	POS	POS	POS	NEG	NEG	NEG	NEG	NEG	POS	POS	POS	NEG
2013_PA05_Z3988	POS	POS	NEG	POS	AMB	AMB	NEG	NEG	NEG	NEG	POS	NEG	POS	NEG	POS	NEG	NEG	NEG	NEG	POS	POS	POS	POS	NEG	NEG	NEG	NEG	NEG	POS	POS	POS	NEG
2013_PA65_Z4048	POS	POS	NEG	POS	NEG	NEG	NEG	NEG	NEG	NEG	POS	NEG	POS	NEG	POS	NEG	NEG	NEG	NEG	POS	POS	POS	POS	NEG	NEG	NEG	NEG	NEG	POS	POS	POS	NEG
2013_PA73_Z4056	POS	POS	NEG	POS	AMB	AMB	NEG	NEG	NEG	NEG	POS	NEG	POS	NEG	POS	NEG	NEG	NEG	NEG	POS	POS	POS	POS	NEG	NEG	NEG	NEG	NEG	POS	POS	POS	NEG
**ST72-MSSA-SCCfus**																																
2013_PA16_Z3999	POS	POS	NEG	POS	AMB	AMB	NEG	NEG	NEG	NEG	POS	NEG	POS	NEG	POS	NEG	NEG	NEG	NEG	POS	POS	POS	POS	NEG	NEG	NEG	NEG	NEG	POS	POS	POS	NEG
**ST72-MSSA-SCCfus/kdp**																																
2012_361_Z3421 (1201-1299)	POS	POS	NEG	POS	AMB	AMB	NEG	NEG	NEG	NEG	POS	NEG	POS	NEG	POS	NEG	NEG	NEG	NEG	POS	POS	POS	POS	NEG	NEG	NEG	NEG	NEG	POS	POS	POS	NEG
**ST239-MRSA-III&SCCmer**																																
2012_157_Z3490	POS	POS	NEG	POS	AMB	AMB	POS	NEG	NEG	NEG	POS	NEG	POS	NEG	POS	NEG	NEG	NEG	NEG	NEG	NEG	NEG	NEG	POS	POS	POS	POS	POS	POS	POS	POS	NEG
2012_168_Z3494 (27129/11)	POS	POS	NEG	POS	AMB	AMB	POS	NEG	NEG	NEG	POS	NEG	POS	NEG	POS	NEG	NEG	NEG	NEG	NEG	NEG	NEG	NEG	POS	POS	POS	POS	POS	POS	POS	POS	NEG
2012_195_Z3508 (619/12)	POS	POS	NEG	AMB	AMB	POS	POS	NEG	NEG	NEG	POS	NEG	POS	NEG	POS	NEG	NEG	NEG	NEG	NEG	NEG	NEG	NEG	POS	POS	POS	POS	POS	POS	POS	POS	NEG
2012_222_Z3479 (1489/12)	POS	POS	NEG	POS	NEG	AMB	POS	NEG	NEG	NEG	POS	NEG	POS	NEG	POS	NEG	NEG	NEG	NEG	NEG	NEG	NEG	NEG	POS	POS	POS	POS	POS	POS	POS	POS	NEG
2012_233_Z3540 (1943/12)	POS	POS	NEG	POS	AMB	AMB	POS	NEG	NEG	NEG	POS	NEG	POS	NEG	POS	NEG	NEG	NEG	NEG	NEG	NEG	NEG	NEG	POS	POS	POS	POS	POS	POS	POS	POS	NEG
2012_237_Z3541	POS	POS	NEG	POS	AMB	AMB	POS	NEG	NEG	NEG	POS	NEG	POS	NEG	POS	NEG	NEG	NEG	NEG	NEG	NEG	NEG	NEG	POS	POS	POS	POS	POS	POS	POS	POS	NEG
2012_262_Z3549	POS	POS	NEG	POS	AMB	AMB	POS	NEG	NEG	NEG	POS	NEG	POS	NEG	POS	NEG	NEG	NEG	NEG	NEG	NEG	NEG	NEG	POS	POS	POS	POS	POS	POS	POS	POS	NEG
2012_275_Z3562 (3893/12)	POS	POS	NEG	POS	AMB	AMB	POS	NEG	NEG	NEG	POS	NEG	POS	NEG	POS	NEG	NEG	NEG	NEG	NEG	NEG	NEG	NEG	POS	POS	POS	POS	POS	POS	POS	POS	NEG
2012_291_Z3578	POS	POS	NEG	POS	NEG	AMB	POS	NEG	NEG	NEG	POS	NEG	POS	NEG	POS	NEG	NEG	NEG	NEG	NEG	NEG	NEG	NEG	POS	POS	POS	POS	POS	POS	POS	POS	NEG
2012_314_Z3601 (6939/12)	POS	POS	NEG	POS	AMB	AMB	POS	NEG	AMB	NEG	POS	NEG	POS	NEG	POS	NEG	NEG	NEG	NEG	AMB	POS	AMB	AMB	POS	POS	POS	POS	POS	POS	POS	POS	NEG
2012323_Z3610	POS	POS	NEG	AMB	AMB	POS	POS	NEG	NEG	NEG	POS	NEG	POS	NEG	POS	NEG	NEG	NEG	NEG	NEG	NEG	NEG	NEG	POS	POS	POS	POS	POS	POS	POS	POS	NEG
2012330_Z3617 (7605/12)	POS	POS	NEG	POS	AMB	AMB	POS	NEG	NEG	NEG	POS	NEG	POS	NEG	POS	NEG	NEG	NEG	NEG	NEG	NEG	NEG	NEG	POS	POS	POS	POS	POS	POS	POS	POS	NEG
2012332_Z3619 (8076/12)	POS	POS	NEG	POS	AMB	AMB	POS	NEG	NEG	NEG	POS	NEG	POS	NEG	POS	NEG	NEG	NEG	NEG	NEG	NEG	NEG	NEG	POS	POS	POS	POS	POS	POS	POS	POS	NEG
2012339_Z3626 (8558/12)	POS	POS	NEG	POS	AMB	AMB	POS	NEG	NEG	NEG	POS	NEG	POS	NEG	POS	NEG	NEG	NEG	NEG	NEG	NEG	NEG	NEG	POS	POS	POS	POS	POS	POS	POS	POS	NEG
2012340_Z3627 (8523/12)	POS	POS	NEG	POS	AMB	AMB	POS	NEG	NEG	NEG	POS	NEG	POS	NEG	POS	NEG	NEG	NEG	NEG	NEG	NEG	NEG	NEG	POS	POS	POS	POS	POS	POS	POS	POS	NEG
2012348_Z3635	POS	POS	NEG	POS	AMB	AMB	POS	NEG	NEG	NEG	POS	NEG	POS	NEG	POS	NEG	NEG	NEG	NEG	NEG	NEG	NEG	NEG	POS	POS	POS	POS	POS	POS	POS	POS	NEG
2012350_Z3637	POS	POS	NEG	POS	NEG	AMB	POS	NEG	NEG	NEG	POS	NEG	POS	NEG	POS	NEG	NEG	NEG	NEG	NEG	NEG	NEG	NEG	POS	POS	POS	POS	POS	POS	POS	POS	NEG
2012_370_Z3430 (1112-52177)	POS	POS	NEG	AMB	AMB	POS	POS	NEG	NEG	NEG	POS	NEG	POS	NEG	POS	NEG	NEG	NEG	NEG	NEG	NEG	NEG	NEG	POS	POS	POS	POS	POS	POS	POS	POS	NEG
2012_378_Z3438 (4139)	POS	POS	NEG	POS	AMB	AMB	POS	NEG	NEG	NEG	POS	NEG	POS	NEG	POS	NEG	NEG	NEG	NEG	NEG	NEG	NEG	NEG	POS	POS	POS	POS	POS	POS	POS	POS	NEG
2013_PA21_Z4004	POS	POS	NEG	POS	NEG	AMB	POS	NEG	NEG	NEG	POS	NEG	POS	NEG	POS	NEG	NEG	NEG	NEG	NEG	NEG	NEG	NEG	POS	POS	POS	POS	POS	POS	POS	POS	NEG
2013_PA22_Z4005	POS	POS	NEG	POS	AMB	AMB	POS	NEG	NEG	NEG	POS	NEG	POS	NEG	POS	NEG	NEG	NEG	NEG	NEG	NEG	NEG	NEG	POS	POS	POS	POS	POS	POS	POS	POS	NEG
2013_PA24_Z4007	POS	POS	NEG	POS	NEG	AMB	POS	NEG	NEG	NEG	POS	NEG	POS	NEG	POS	NEG	NEG	NEG	NEG	NEG	NEG	NEG	NEG	POS	POS	POS	POS	POS	POS	POS	POS	NEG
2013_PA31_Z4014	POS	POS	NEG	POS	AMB	AMB	POS	NEG	NEG	NEG	POS	NEG	POS	NEG	POS	NEG	NEG	NEG	NEG	NEG	NEG	NEG	NEG	POS	POS	POS	POS	POS	POS	POS	POS	NEG
2013_PA35_Z4018	POS	POS	NEG	POS	NEG	NEG	POS	NEG	NEG	NEG	POS	NEG	POS	NEG	POS	NEG	NEG	NEG	NEG	NEG	NEG	NEG	NEG	POS	POS	POS	POS	POS	POS	POS	POS	NEG
2013_PA81_Z4064	POS	POS	NEG	POS	AMB	AMB	POS	NEG	NEG	NEG	POS	NEG	POS	NEG	POS	NEG	NEG	NEG	NEG	NEG	NEG	NEG	NEG	POS	POS	POS	POS	POS	POS	POS	POS	NEG
**ST239-MRSA-III+ccrA/B4**																																
2012_254_Z3537 (3124A/12)	POS	POS	NEG	POS	NEG	AMB	POS	NEG	NEG	NEG	POS	NEG	POS	NEG	POS	NEG	NEG	NEG	NEG	NEG	NEG	NEG	NEG	POS	POS	POS	POS	POS	POS	POS	POS	NEG
2013_PA25_Z4008	POS	POS	NEG	POS	AMB	AMB	POS	NEG	NEG	NEG	POS	NEG	POS	NEG	POS	NEG	NEG	NEG	NEG	NEG	NEG	NEG	NEG	POS	POS	POS	POS	POS	POS	POS	POS	NEG
**ST834-MSSA**																																
2012_268_Z3555 (3693/12)	POS	POS	AMB	POS	AMB	AMB	NEG	NEG	NEG	NEG	POS	NEG	POS	NEG	POS	NEG	NEG	NEG	NEG	NEG	NEG	NEG	NEG	POS	POS	POS	POS	POS	POS	POS	POS	NEG
**CC12-MSSA**																																
2012_210_Z3470 (1177/12)	POS	POS	NEG	POS	NEG	AMB	POS	NEG	NEG	NEG	POS	NEG	POS	NEG	POS	NEG	NEG	NEG	NEG	NEG	NEG	NEG	NEG	POS	POS	POS	POS	POS	POS	POS	POS	NEG
2012_230_Z3487 (1797/12)	POS	POS	NEG	AMB	AMB	AMB	POS	NEG	NEG	NEG	POS	NEG	POS	NEG	POS	NEG	NEG	NEG	NEG	NEG	NEG	NEG	NEG	POS	POS	POS	POS	POS	POS	POS	POS	NEG
2013_PA18_Z4001	POS	POS	NEG	POS	AMB	AMB	POS	NEG	NEG	NEG	POS	NEG	POS	NEG	POS	NEG	NEG	NEG	NEG	NEG	NEG	NEG	NEG	POS	POS	POS	POS	POS	POS	POS	POS	NEG
**CC15-MSSA**																																
2012_160_Z3444 (26386/11)	POS	POS	AMB	AMB	AMB	AMB	NEG	NEG	NEG	POS	POS	NEG	POS	NEG	POS	NEG	NEG	NEG	NEG	NEG	NEG	NEG	NEG	POS	POS	POS	POS	POS	POS	POS	POS	NEG
2012_167_Z3514 (27010/11)	POS	POS	AMB	POS	AMB	AMB	NEG	NEG	NEG	POS	POS	NEG	POS	NEG	POS	NEG	NEG	NEG	NEG	NEG	NEG	NEG	NEG	POS	POS	POS	POS	POS	POS	POS	POS	NEG
2012_184_Z3455 (376/12)	POS	POS	AMB	POS	AMB	AMB	NEG	NEG	NEG	POS	POS	NEG	POS	NEG	POS	NEG	AMB	NEG	NEG	NEG	NEG	NEG	NEG	POS	POS	POS	POS	POS	POS	POS	POS	NEG
2012_205_Z3465 (939/12)	POS	POS	NEG	POS	AMB	AMB	NEG	NEG	NEG	POS	POS	NEG	POS	NEG	POS	NEG	AMB	NEG	NEG	NEG	NEG	NEG	NEG	POS	POS	POS	POS	POS	POS	POS	POS	NEG
2012_231_Z3519 (1831/12)	POS	POS	NEG	AMB	AMB	AMB	NEG	NEG	NEG	POS	POS	NEG	POS	NEG	POS	NEG	NEG	NEG	NEG	NEG	NEG	NEG	NEG	POS	POS	POS	POS	POS	POS	POS	POS	NEG
2012_249_Z3533 (2907/12)	POS	POS	NEG	POS	AMB	AMB	NEG	NEG	NEG	POS	POS	NEG	POS	NEG	POS	NEG	NEG	NEG	AMB	NEG	NEG	NEG	NEG	POS	POS	POS	POS	POS	POS	POS	POS	NEG
2012_305_Z3592 (5839/12)	POS	POS	NEG	POS	AMB	NEG	NEG	NEG	NEG	POS	POS	NEG	POS	NEG	POS	NEG	NEG	NEG	NEG	NEG	NEG	NEG	NEG	POS	POS	POS	POS	POS	POS	POS	POS	NEG
2012_313_Z3600 (6857/12)	POS	POS	NEG	POS	AMB	AMB	NEG	NEG	NEG	POS	POS	NEG	POS	NEG	POS	NEG	NEG	NEG	NEG	NEG	NEG	NEG	NEG	POS	POS	POS	POS	POS	POS	POS	POS	NEG
2012_345_Z3632 (7966B/12)	POS	POS	NEG	POS	AMB	AMB	NEG	NEG	NEG	POS	POS	NEG	POS	NEG	POS	NEG	AMB	NEG	NEG	NEG	NEG	NEG	NEG	POS	POS	POS	POS	POS	POS	POS	POS	NEG
2013_PA33_Z4016	POS	POS	NEG	POS	AMB	NEG	NEG	NEG	NEG	POS	POS	NEG	NEG	NEG	POS	NEG	NEG	NEG	NEG	NEG	NEG	NEG	NEG	POS	POS	POS	POS	POS	POS	POS	POS	NEG
**CC20-MSSA**																																
2012_240_Z3526 (2354/12)	POS	POS	AMB	POS	AMB	AMB	NEG	NEG	NEG	NEG	POS	NEG	POS	NEG	POS	NEG	NEG	AMB	NEG	POS	POS	POS	POS	NEG	NEG	NEG	NEG	NEG	POS	POS	POS	NEG
**CC30-MSSA**																																
2012_155_Z3488 (25905A/11)	NEG	NEG	POS	AMB	NEG	POS	NEG	NEG	NEG	POS	NEG	POS	NEG	POS	POS	NEG	NEG	NEG	NEG	NEG	NEG	NEG	NEG	POS	POS	POS	POS	POS	POS	POS	POS	NEG
**CC30-MSSA [PVL+]**																																
2012_164_Z3447 (26574/11)	NEG	NEG	POS	AMB	NEG	POS	NEG	NEG	NEG	POS	NEG	POS	NEG	POS	POS	AMB	AMB	NEG	AMB	NEG	NEG	NEG	NEG	POS	POS	POS	POS	POS	POS	POS	POS	NEG
2012_166_Z3493 (26988A/11)	NEG	NEG	POS	AMB	NEG	POS	NEG	NEG	NEG	POS	NEG	POS	NEG	POS	POS	NEG	NEG	NEG	NEG	NEG	NEG	NEG	NEG	POS	POS	POS	POS	POS	POS	POS	POS	NEG
2012_178_Z3500 (197/12)	NEG	NEG	POS	AMB	NEG	POS	NEG	NEG	NEG	POS	NEG	POS	NEG	POS	POS	NEG	NEG	NEG	NEG	NEG	NEG	NEG	NEG	POS	POS	POS	POS	POS	POS	POS	POS	NEG
2012_183_Z3454	NEG	NEG	POS	AMB	NEG	POS	NEG	NEG	NEG	POS	NEG	POS	NEG	POS	POS	NEG	AMB	NEG	NEG	NEG	NEG	NEG	NEG	POS	POS	POS	POS	POS	POS	POS	POS	NEG
2012_186_Z3503 (444/12)	NEG	NEG	POS	POS	NEG	AMB	NEG	NEG	NEG	POS	NEG	POS	NEG	POS	POS	NEG	NEG	NEG	NEG	NEG	NEG	NEG	NEG	POS	POS	POS	POS	POS	POS	POS	POS	NEG
2012_189_Z3506 (542/12)	NEG	NEG	POS	AMB	NEG	POS	NEG	NEG	NEG	POS	NEG	POS	NEG	POS	POS	NEG	NEG	NEG	NEG	NEG	NEG	NEG	NEG	POS	POS	POS	POS	POS	POS	POS	POS	NEG
2012_192_Z3457 (606/12)	NEG	NEG	POS	AMB	NEG	POS	NEG	NEG	NEG	POS	NEG	POS	NEG	POS	POS	NEG	NEG	NEG	NEG	NEG	NEG	NEG	NEG	POS	POS	POS	POS	POS	POS	POS	POS	NEG
2012_196_Z3509 (707/12)	NEG	NEG	POS	POS	NEG	AMB	NEG	NEG	NEG	POS	NEG	POS	NEG	POS	POS	NEG	AMB	NEG	NEG	NEG	NEG	NEG	NEG	POS	POS	POS	POS	POS	POS	POS	POS	NEG
2012_218_Z3476 (1432/12)	NEG	NEG	POS	AMB	NEG	POS	NEG	NEG	NEG	POS	NEG	POS	NEG	POS	POS	NEG	NEG	NEG	NEG	NEG	NEG	NEG	NEG	POS	POS	POS	POS	POS	POS	POS	POS	NEG
2012_232_Z3520 (1892/12)	NEG	NEG	POS	AMB	NEG	POS	NEG	NEG	NEG	POS	NEG	POS	NEG	POS	POS	NEG	NEG	NEG	NEG	NEG	NEG	NEG	NEG	POS	POS	POS	POS	POS	POS	POS	POS	NEG
2012_278_Z3565 (4397/12)	NEG	NEG	POS	AMB	NEG	POS	NEG	NEG	NEG	POS	NEG	POS	NEG	POS	POS	NEG	NEG	NEG	AMB	NEG	NEG	NEG	NEG	POS	POS	POS	POS	POS	POS	POS	POS	NEG
2012_285_Z3572 (4802/12)	NEG	NEG	POS	AMB	NEG	POS	NEG	NEG	NEG	POS	NEG	POS	NEG	POS	POS	NEG	NEG	NEG	NEG	NEG	NEG	NEG	NEG	POS	POS	POS	POS	POS	POS	POS	POS	NEG
2012_287_Z3574 (4906/12)	NEG	NEG	POS	AMB	NEG	POS	NEG	NEG	NEG	POS	NEG	POS	NEG	POS	POS	NEG	NEG	NEG	NEG	NEG	NEG	NEG	NEG	POS	POS	POS	POS	POS	POS	POS	POS	NEG
2012_295_Z3582 (5116/12)	NEG	NEG	POS	AMB	NEG	POS	NEG	NEG	NEG	POS	NEG	POS	NEG	POS	POS	NEG	NEG	NEG	NEG	NEG	NEG	NEG	NEG	POS	POS	POS	POS	POS	POS	POS	POS	NEG
2012_326_Z3613 (7321/12)	NEG	NEG	POS	AMB	NEG	POS	NEG	NEG	AMB	POS	NEG	POS	NEG	POS	POS	NEG	NEG	NEG	NEG	NEG	NEG	NEG	NEG	POS	POS	POS	POS	POS	POS	POS	POS	NEG
2012_356_Z3643 (1204-15897)	NEG	NEG	POS	AMB	NEG	POS	NEG	NEG	NEG	POS	NEG	POS	NEG	POS	POS	NEG	NEG	NEG	NEG	NEG	NEG	NEG	NEG	POS	POS	POS	POS	POS	POS	POS	POS	NEG
2012_366_Z3426 (1204-16124)	NEG	NEG	POS	AMB	NEG	POS	NEG	NEG	NEG	POS	NEG	POS	NEG	POS	POS	NEG	AMB	NEG	NEG	NEG	NEG	NEG	NEG	POS	POS	POS	POS	POS	POS	POS	POS	NEG
2012_372_Z3432 (1205-19821)	NEG	NEG	POS	AMB	NEG	POS	NEG	NEG	NEG	POS	NEG	POS	NEG	POS	POS	NEG	NEG	NEG	NEG	NEG	NEG	NEG	NEG	POS	POS	POS	POS	POS	POS	POS	POS	NEG
2012_380_Z3440 (4141)	NEG	NEG	POS	AMB	NEG	POS	NEG	NEG	NEG	POS	NEG	POS	NEG	POS	POS	NEG	NEG	NEG	NEG	NEG	NEG	NEG	NEG	POS	POS	POS	POS	POS	POS	POS	POS	NEG
2013_PA20_Z4003	NEG	NEG	POS	POS	NEG	AMB	NEG	NEG	NEG	POS	NEG	POS	NEG	POS	POS	NEG	NEG	NEG	NEG	NEG	NEG	NEG	NEG	POS	POS	POS	POS	POS	POS	POS	POS	NEG
2013_PA36_Z4019	NEG	NEG	POS	AMB	NEG	POS	NEG	NEG	NEG	NEG	NEG	POS	NEG	POS	POS	NEG	NEG	NEG	NEG	NEG	NEG	NEG	NEG	POS	AMB	POS	POS	POS	POS	POS	POS	NEG
2013_PA46_Z4029	NEG	NEG	POS	AMB	NEG	POS	NEG	NEG	NEG	AMB	NEG	POS	NEG	POS	POS	NEG	NEG	NEG	NEG	NEG	NEG	NEG	NEG	POS	POS	POS	POS	POS	POS	POS	POS	NEG
2013_PA50_Z4033	NEG	NEG	POS	AMB	NEG	POS	NEG	NEG	NEG	AMB	NEG	POS	NEG	POS	POS	NEG	NEG	NEG	NEG	NEG	NEG	NEG	NEG	POS	POS	POS	POS	POS	POS	POS	POS	NEG
2013_PA63_Z4046	NEG	NEG	POS	POS	NEG	AMB	NEG	NEG	NEG	POS	NEG	POS	NEG	POS	POS	NEG	NEG	NEG	NEG	NEG	NEG	NEG	NEG	POS	POS	POS	POS	POS	POS	POS	POS	NEG
2013_PA78_Z4061	NEG	NEG	POS	AMB	NEG	POS	NEG	NEG	NEG	POS	NEG	POS	NEG	POS	POS	NEG	NEG	NEG	NEG	NEG	NEG	NEG	NEG	POS	POS	POS	POS	POS	POS	POS	POS	NEG
2013_PA79_Z4062	NEG	NEG	POS	AMB	NEG	POS	NEG	NEG	NEG	POS	NEG	POS	NEG	POS	POS	NEG	NEG	NEG	NEG	NEG	NEG	NEG	NEG	POS	POS	POS	POS	POS	POS	POS	POS	NEG
**CC45-MSSA**																																
2012_172_Z3449 (49/12)	NEG	NEG	POS	POS	NEG	AMB	NEG	NEG	NEG	NEG	NEG	POS	NEG	POS	POS	NEG	NEG	NEG	NEG	NEG	NEG	NEG	NEG	POS	POS	POS	POS	POS	POS	POS	POS	NEG
2012_208_Z3468 (1011/12)	NEG	NEG	POS	POS	NEG	AMB	NEG	NEG	NEG	NEG	NEG	POS	NEG	AMB	NEG	NEG	NEG	NEG	NEG	NEG	NEG	NEG	NEG	POS	POS	POS	POS	POS	POS	POS	POS	NEG
2012_235_Z3522 (2085/12)	NEG	NEG	POS	AMB	NEG	POS	NEG	NEG	NEG	NEG	NEG	POS	NEG	POS	AMB	NEG	NEG	NEG	NEG	NEG	NEG	NEG	NEG	POS	POS	POS	POS	POS	POS	POS	POS	NEG
2012_290_Z3577 (4921/12)	NEG	NEG	POS	AMB	NEG	POS	NEG	NEG	NEG	NEG	NEG	POS	NEG	POS	AMB	NEG	NEG	NEG	NEG	NEG	NEG	NEG	NEG	POS	POS	POS	POS	POS	POS	POS	POS	NEG
2012_311_Z3598 (6182/12)	NEG	NEG	POS	AMB	NEG	POS	NEG	NEG	NEG	NEG	NEG	POS	NEG	AMB	AMB	NEG	NEG	NEG	NEG	NEG	NEG	NEG	NEG	POS	POS	POS	POS	POS	POS	POS	POS	NEG
Z3604_TT317 - {DC558C18-2959-4049-B8F3-507365	NEG	NEG	POS	POS	NEG	AMB	NEG	NEG	NEG	NEG	NEG	POS	NEG	POS	NEG	NEG	NEG	NEG	NEG	NEG	NEG	NEG	NEG	POS	POS	POS	POS	POS	POS	POS	POS	NEG
2012_363_Z3423 (1112-61058)	NEG	NEG	POS	AMB	NEG	POS	NEG	NEG	NEG	NEG	NEG	POS	NEG	POS	NEG	NEG	AMB	NEG	NEG	NEG	NEG	NEG	NEG	POS	POS	POS	POS	POS	POS	POS	POS	NEG
2013_PA19_Z4002	NEG	NEG	POS	POS	NEG	AMB	NEG	NEG	NEG	NEG	NEG	POS	NEG	POS	NEG	NEG	NEG	NEG	NEG	NEG	NEG	NEG	NEG	POS	POS	POS	POS	POS	POS	POS	POS	NEG
2013_PA54_Z4037	NEG	NEG	POS	POS	AMB	AMB	NEG	NEG	NEG	NEG	NEG	POS	NEG	POS	POS	NEG	NEG	NEG	NEG	NEG	NEG	NEG	NEG	POS	POS	POS	POS	POS	POS	POS	POS	NEG
2013_PA82_Z4065	NEG	NEG	POS	AMB	NEG	POS	NEG	NEG	NEG	NEG	NEG	POS	NEG	POS	NEG	NEG	NEG	NEG	NEG	NEG	NEG	NEG	NEG	POS	POS	POS	POS	POS	POS	POS	POS	NEG
**CC97-MSSA**																																
2012_182_Z3453 (279A/12)	POS	POS	NEG	POS	AMB	AMB	NEG	NEG	NEG	NEG	POS	NEG	POS	NEG	POS	NEG	NEG	AMB	NEG	POS	POS	POS	POS	NEG	NEG	NEG	NEG	NEG	POS	POS	POS	NEG
2012_187_Z3504	POS	POS	NEG	POS	NEG	AMB	NEG	NEG	NEG	NEG	POS	NEG	POS	NEG	POS	NEG	NEG	NEG	NEG	POS	POS	POS	POS	NEG	NEG	NEG	NEG	NEG	POS	POS	POS	NEG
2012_197_Z3510 (757/12)	POS	POS	NEG	POS	AMB	AMB	NEG	NEG	NEG	NEG	POS	NEG	POS	NEG	POS	NEG	NEG	NEG	NEG	POS	POS	POS	POS	NEG	NEG	NEG	NEG	NEG	POS	POS	POS	NEG
2012_199_Z3516 (785/12)	POS	POS	NEG	POS	AMB	AMB	NEG	NEG	NEG	NEG	POS	NEG	POS	NEG	POS	NEG	NEG	NEG	NEG	POS	POS	AMB	POS	NEG	NEG	NEG	NEG	NEG	POS	POS	POS	NEG
2013_PA15_Z3998	POS	POS	NEG	POS	AMB	AMB	NEG	NEG	NEG	NEG	POS	NEG	POS	NEG	POS	NEG	NEG	NEG	NEG	POS	POS	POS	POS	NEG	NEG	NEG	NEG	NEG	POS	POS	POS	NEG
**CC101-MSSA**																																
2012_283_Z3570 (4725/12)	POS	POS	NEG	POS	NEG	AMB	NEG	NEG	NEG	NEG	POS	NEG	POS	NEG	POS	NEG	NEG	NEG	NEG	NEG	NEG	NEG	NEG	POS	POS	POS	POS	POS	POS	POS	POS	NEG
2012_247_Z3531 (2788/12)	POS	POS	NEG	POS	AMB	AMB	NEG	NEG	NEG	NEG	POS	NEG	POS	NEG	POS	NEG	NEG	NEG	NEG	NEG	NEG	NEG	NEG	POS	POS	POS	POS	POS	POS	POS	POS	NEG
2012_279_Z3566 (4506/12)	POS	POS	NEG	POS	AMB	AMB	NEG	NEG	NEG	NEG	POS	NEG	POS	NEG	POS	NEG	NEG	NEG	NEG	NEG	NEG	NEG	NEG	POS	POS	POS	POS	POS	POS	POS	POS	NEG

Strain / Isolates	staphylococcal superantigen-like protein 9			staphylococcal superantigen-like protein 10			staphylococcal superantigene-like protein 11				staphylococcal exotoxin-like protein, second locus					Capsule type 1	capsular poly-saccharide synthesis enzyme	O-antigen poly-merase	capsular poly-saccharide biosyn-thesis protein	Capsule type 5	capsular poly-saccharide synthesis enzyme	O-antigen poly-merase	capsular poly-saccharide biosyn-thesis protein	Capsule type 8	capsular poly-saccharide synthesis enzyme	capsular poly-saccharide biosyn-thesis protein	O-antigen poly-merase	capsular poly-saccharide biosyn-thesis protein	intercellular adhesion protein A	intercellular adhesion protein C	biofilm PIA synthesis protein D	surface protein involved in biofilm formation
	ssl09/set5_probe 1	ssl09/set5_probe 2	ssl09/set5 (MRSA252)	ssl10/set4	ssl10 (RF122)	ssl10/set4 (MRSA252)	ssl11/set2 (COL)	ssl11+set2(Mu50+N315)	ssl11+set2(MW2+MSSA 476)	ssl11/set2 (MRSA252)	setB3	setB3 (MRSA252)	setB2	setB2 (MRSA252)	setB1	cap 1 (total)	capH1	capJ1	capK1	cap 5 (total)	capH5	capJ5	capK5	cap 8 (total)	capH8	capI8	capJ8	capK8	icaA	icaC	icaD	bap
**CC121-MSSA**																																
2012_163_Z3446 (26495/11)	POS	POS	NEG	POS	AMB	AMB	NEG	NEG	NEG	NEG	POS	NEG	POS	NEG	POS	NEG	NEG	NEG	NEG	NEG	NEG	NEG	NEG	POS	POS	POS	POS	POS	POS	POS	POS	NEG
2012_204_Z3464 (938/12)	POS	POS	NEG	POS	AMB	AMB	NEG	NEG	NEG	NEG	POS	NEG	POS	NEG	POS	NEG	NEG	NEG	NEG	NEG	NEG	NEG	NEG	POS	POS	POS	POS	POS	POS	POS	POS	NEG
2012_225_Z3482 (1563/12)	POS	POS	NEG	POS	AMB	AMB	NEG	NEG	NEG	NEG	POS	NEG	POS	NEG	POS	NEG	NEG	NEG	NEG	NEG	NEG	NEG	NEG	POS	POS	POS	POS	POS	POS	POS	POS	NEG
2012_228_Z3485 (17008/12)	POS	POS	NEG	POS	AMB	AMB	NEG	NEG	NEG	NEG	POS	NEG	POS	NEG	POS	NEG	NEG	NEG	NEG	NEG	NEG	NEG	NEG	POS	POS	POS	POS	POS	POS	POS	POS	NEG
2012_229_Z3486 (1737/12)	POS	POS	NEG	POS	AMB	AMB	NEG	NEG	NEG	NEG	POS	NEG	POS	NEG	POS	NEG	NEG	NEG	NEG	NEG	NEG	NEG	NEG	POS	POS	POS	POS	POS	POS	POS	POS	NEG
2012_302_Z3589 (5795/12)	POS	POS	NEG	POS	AMB	NEG	NEG	NEG	NEG	NEG	POS	NEG	POS	NEG	POS	NEG	NEG	NEG	NEG	NEG	NEG	NEG	NEG	POS	POS	POS	POS	POS	POS	POS	POS	NEG
2012_359_Z3419	POS	POS	NEG	POS	AMB	NEG	NEG	NEG	NEG	NEG	POS	NEG	POS	NEG	POS	NEG	NEG	NEG	NEG	NEG	NEG	NEG	NEG	POS	POS	POS	POS	POS	POS	POS	POS	NEG
2012_362_Z3422 (1201-996)	POS	POS	NEG	POS	AMB	AMB	NEG	NEG	NEG	NEG	POS	NEG	POS	NEG	POS	NEG	NEG	NEG	NEG	NEG	NEG	NEG	NEG	POS	POS	POS	POS	POS	POS	POS	POS	NEG
2012_367_Z3427 (1204-16413)	POS	POS	NEG	POS	AMB	AMB	NEG	NEG	NEG	NEG	POS	NEG	POS	NEG	POS	NEG	NEG	NEG	NEG	NEG	NEG	NEG	NEG	POS	POS	POS	POS	POS	POS	POS	POS	NEG
2013_PA09_Z3992	POS	POS	NEG	POS	AMB	AMB	NEG	NEG	NEG	NEG	POS	NEG	POS	NEG	POS	NEG	NEG	NEG	NEG	NEG	NEG	NEG	NEG	POS	POS	POS	POS	POS	POS	POS	POS	NEG
2013_PA59_Z4042	POS	POS	NEG	POS	AMB	NEG	NEG	NEG	NEG	NEG	POS	NEG	POS	NEG	POS	NEG	NEG	NEG	NEG	NEG	NEG	NEG	NEG	POS	POS	POS	POS	POS	POS	POS	POS	NEG
2013_PA60_Z4043	POS	POS	NEG	POS	AMB	NEG	NEG	NEG	NEG	NEG	POS	NEG	POS	NEG	POS	NEG	NEG	NEG	NEG	NEG	NEG	NEG	NEG	POS	POS	POS	POS	POS	POS	POS	POS	NEG
**CC152-MSSA [PVL+]**																																
2012_190_Z3507 (549/12)	NEG	NEG	NEG	POS	NEG	AMB	NEG	NEG	AMB	NEG	POS	NEG	POS	NEG	POS	NEG	NEG	NEG	NEG	POS	POS	POS	POS	NEG	NEG	NEG	NEG	NEG	POS	NEG	POS	NEG
2012_201_Z3461 (823/12)	NEG	NEG	NEG	POS	AMB	AMB	NEG	NEG	AMB	NEG	POS	NEG	POS	NEG	POS	NEG	NEG	NEG	NEG	POS	POS	POS	POS	NEG	NEG	NEG	NEG	NEG	POS	NEG	POS	NEG
2012_209_Z3469 (1116/12)	NEG	NEG	NEG	POS	NEG	AMB	NEG	NEG	AMB	NEG	POS	NEG	POS	NEG	POS	NEG	NEG	NEG	NEG	POS	POS	POS	POS	NEG	NEG	NEG	NEG	NEG	POS	NEG	POS	NEG
2012_211_Z3517 (1238/12)	NEG	NEG	NEG	POS	NEG	AMB	NEG	NEG	NEG	NEG	POS	NEG	POS	NEG	POS	NEG	NEG	NEG	NEG	POS	POS	POS	POS	NEG	NEG	NEG	NEG	NEG	POS	NEG	POS	NEG
2012_213_Z3472 (1320/12)	NEG	NEG	NEG	POS	NEG	AMB	NEG	NEG	NEG	NEG	POS	NEG	POS	NEG	POS	NEG	NEG	NEG	NEG	POS	POS	POS	POS	NEG	NEG	NEG	NEG	NEG	POS	NEG	POS	NEG
2012_221_Z3478	NEG	NEG	NEG	POS	NEG	AMB	NEG	NEG	NEG	NEG	POS	NEG	POS	NEG	POS	NEG	NEG	NEG	NEG	POS	POS	POS	POS	NEG	NEG	NEG	NEG	NEG	POS	NEG	POS	NEG
2012_234_Z3521 (1981/12)	NEG	NEG	NEG	POS	AMB	AMB	NEG	NEG	NEG	NEG	POS	NEG	POS	NEG	POS	NEG	NEG	NEG	NEG	POS	POS	POS	POS	NEG	NEG	NEG	NEG	NEG	POS	NEG	POS	NEG
2012_238_Z3524 (2199/12)	NEG	NEG	NEG	POS	NEG	AMB	NEG	NEG	NEG	NEG	POS	NEG	POS	NEG	POS	NEG	NEG	NEG	NEG	POS	POS	POS	POS	NEG	NEG	NEG	NEG	NEG	POS	NEG	POS	NEG
2012_260_Z3547 (3290/12)	NEG	NEG	NEG	POS	AMB	AMB	NEG	NEG	AMB	NEG	POS	NEG	POS	NEG	POS	NEG	NEG	NEG	NEG	POS	POS	POS	POS	NEG	NEG	NEG	NEG	NEG	POS	NEG	POS	NEG
2012_272_Z3559 (3802/12)	NEG	NEG	NEG	POS	AMB	AMB	NEG	NEG	NEG	NEG	POS	NEG	POS	NEG	POS	NEG	AMB	NEG	NEG	POS	POS	POS	POS	NEG	NEG	NEG	NEG	NEG	POS	NEG	POS	NEG
2012_289_Z3576 (4912/12)	NEG	NEG	NEG	POS	NEG	AMB	NEG	NEG	NEG	NEG	POS	NEG	POS	NEG	POS	NEG	NEG	NEG	NEG	POS	POS	POS	POS	NEG	NEG	NEG	NEG	NEG	POS	NEG	POS	NEG
2012_309_Z3596	NEG	NEG	NEG	POS	NEG	AMB	NEG	NEG	NEG	NEG	POS	NEG	POS	NEG	POS	NEG	NEG	NEG	NEG	POS	POS	POS	POS	NEG	NEG	NEG	NEG	NEG	POS	NEG	POS	NEG
2012_322_Z3609 (7178/12)	NEG	NEG	NEG	NEG	NEG	NEG	NEG	NEG	NEG	NEG	POS	NEG	POS	NEG	POS	NEG	NEG	NEG	NEG	POS	POS	POS	POS	NEG	NEG	NEG	NEG	NEG	POS	NEG	POS	NEG
2012_325_Z3612 (7254/12)	NEG	NEG	NEG	POS	AMB	AMB	NEG	NEG	AMB	NEG	POS	NEG	POS	NEG	POS	NEG	NEG	NEG	NEG	POS	POS	POS	POS	NEG	NEG	NEG	NEG	NEG	POS	NEG	POS	NEG
2012_328_Z3615 (7456/12)	NEG	NEG	NEG	POS	AMB	AMB	NEG	NEG	AMB	NEG	POS	NEG	POS	NEG	POS	NEG	NEG	NEG	NEG	POS	POS	POS	POS	NEG	NEG	NEG	NEG	NEG	POS	NEG	POS	NEG
2012_334_Z3621 (8267/12)	NEG	NEG	NEG	POS	AMB	AMB	NEG	NEG	NEG	NEG	POS	NEG	POS	NEG	POS	NEG	NEG	NEG	NEG	POS	POS	POS	POS	NEG	NEG	NEG	NEG	NEG	POS	NEG	POS	NEG
2012_360_Z3420 (1201-4047)	NEG	NEG	NEG	POS	AMB	AMB	NEG	NEG	NEG	NEG	POS	NEG	POS	NEG	POS	NEG	AMB	NEG	NEG	POS	POS	POS	POS	NEG	NEG	NEG	NEG	NEG	POS	NEG	POS	NEG
2012_382_Z3442 (4086)	NEG	NEG	NEG	POS	AMB	AMB	NEG	NEG	NEG	NEG	POS	NEG	POS	NEG	POS	NEG	NEG	NEG	NEG	POS	POS	POS	POS	NEG	NEG	NEG	NEG	NEG	POS	NEG	POS	NEG
2013_PA02_Z3985	NEG	NEG	NEG	POS	AMB	AMB	NEG	NEG	NEG	NEG	POS	NEG	POS	NEG	POS	NEG	NEG	NEG	NEG	POS	POS	POS	POS	NEG	NEG	NEG	NEG	NEG	POS	NEG	POS	NEG
2013_PA03_Z3986	NEG	NEG	NEG	POS	NEG	AMB	NEG	NEG	NEG	NEG	POS	NEG	POS	NEG	POS	NEG	NEG	NEG	NEG	POS	POS	POS	POS	NEG	NEG	NEG	NEG	NEG	POS	NEG	POS	NEG
2013_PA07_Z3990	NEG	NEG	NEG	POS	AMB	AMB	NEG	NEG	NEG	NEG	POS	NEG	POS	NEG	POS	NEG	NEG	NEG	NEG	POS	POS	POS	POS	NEG	NEG	NEG	NEG	NEG	POS	NEG	POS	NEG
2013_PA34_Z4017	NEG	NEG	NEG	POS	NEG	NEG	NEG	NEG	NEG	NEG	POS	NEG	POS	NEG	AMB	NEG	NEG	NEG	NEG	POS	POS	POS	POS	NEG	NEG	NEG	NEG	NEG	POS	NEG	POS	NEG
2013_PA40_Z4023	NEG	NEG	NEG	POS	NEG	NEG	NEG	NEG	NEG	NEG	POS	NEG	POS	NEG	AMB	NEG	NEG	NEG	NEG	POS	POS	POS	POS	NEG	NEG	NEG	NEG	NEG	POS	NEG	POS	NEG
2013_PA48_Z4031	NEG	NEG	NEG	POS	NEG	AMB	NEG	NEG	NEG	NEG	POS	NEG	POS	NEG	POS	NEG	NEG	NEG	NEG	POS	POS	POS	POS	NEG	NEG	NEG	NEG	NEG	POS	NEG	POS	NEG
2013_PA49_Z4032	NEG	NEG	NEG	POS	NEG	NEG	NEG	NEG	NEG	NEG	POS	NEG	POS	NEG	AMB	NEG	NEG	NEG	NEG	POS	POS	POS	POS	NEG	NEG	NEG	NEG	NEG	POS	NEG	POS	NEG
2013_PA51_Z4034	NEG	NEG	NEG	POS	NEG	AMB	NEG	NEG	NEG	NEG	POS	NEG	POS	NEG	POS	NEG	NEG	NEG	NEG	POS	POS	POS	POS	NEG	NEG	NEG	NEG	NEG	POS	NEG	POS	NEG
2013_PA70_Z4053	NEG	NEG	NEG	POS	NEG	NEG	NEG	NEG	NEG	NEG	POS	NEG	POS	NEG	POS	NEG	NEG	NEG	NEG	POS	POS	POS	POS	NEG	NEG	NEG	NEG	NEG	POS	NEG	POS	NEG
2013_PA84_Z4067	NEG	NEG	NEG	POS	NEG	AMB	NEG	NEG	NEG	NEG	POS	NEG	POS	NEG	POS	NEG	NEG	NEG	NEG	POS	POS	POS	POS	NEG	NEG	NEG	NEG	NEG	POS	NEG	POS	NEG
**CC188-MSSA**																																
2012_206_Z3466 (968/12)	POS	POS	AMB	POS	AMB	AMB	NEG	NEG	POS	NEG	POS	NEG	POS	NEG	POS	AMB	AMB	NEG	AMB	NEG	NEG	NEG	NEG	POS	POS	POS	POS	POS	POS	POS	POS	NEG
2012_251_Z3534 (3026/12)	POS	POS	AMB	POS	AMB	AMB	NEG	NEG	POS	NEG	POS	NEG	POS	NEG	POS	NEG	AMB	NEG	NEG	NEG	NEG	NEG	NEG	POS	POS	POS	POS	POS	POS	POS	POS	NEG
2012_320_Z3607 (7144/12)	POS	POS	NEG	AMB	AMB	POS	NEG	NEG	POS	NEG	POS	NEG	POS	NEG	POS	NEG	NEG	NEG	NEG	NEG	NEG	NEG	NEG	POS	POS	POS	POS	POS	POS	POS	POS	NEG
2012_321_Z3608 (7147/12)	POS	POS	NEG	AMB	AMB	POS	NEG	NEG	POS	NEG	POS	NEG	POS	NEG	POS	NEG	NEG	NEG	NEG	NEG	NEG	NEG	NEG	POS	POS	POS	POS	POS	POS	POS	POS	NEG
2012_358_Z3418 (1202-6332)	POS	POS	AMB	POS	AMB	AMB	NEG	NEG	POS	NEG	POS	NEG	POS	NEG	POS	NEG	NEG	NEG	NEG	NEG	NEG	NEG	NEG	POS	POS	POS	POS	POS	POS	POS	POS	NEG
**CC398-MSSA**																																
2013_PA11_Z3994	NEG	NEG	POS	AMB	NEG	POS	NEG	NEG	NEG	POS	NEG	POS	NEG	POS	POS	NEG	NEG	NEG	NEG	POS	POS	POS	POS	NEG	NEG	NEG	NEG	NEG	POS	POS	POS	NEG
2013_PA57_Z4040	NEG	NEG	POS	POS	NEG	AMB	NEG	NEG	NEG	POS	NEG	POS	NEG	POS	POS	NEG	NEG	NEG	NEG	POS	POS	POS	POS	NEG	NEG	NEG	NEG	NEG	POS	POS	POS	NEG
**ST1852-MSSA**																																
2012_277_Z3564 (4050/12)	NEG	NEG	NEG	AMB	AMB	POS	POS	NEG	NEG	NEG	NEG	NEG	NEG	NEG	POS	NEG	NEG	NEG	AMB	NEG	NEG	NEG	NEG	POS	POS	POS	POS	POS	POS	POS	POS	NEG
**CC1223/1594-MSSA**																																
2012_337_Z3624 (8070B/12)	POS	POS	NEG	AMB	NEG	POS	NEG	NEG	NEG	NEG	NEG	NEG	NEG	NEG	AMB	NEG	NEG	NEG	NEG	NEG	NEG	NEG	NEG	NEG	NEG	NEG	NEG	NEG	POS	NEG	NEG	NEG
**MSSA related to ST2250/2277**																																
2012_203_Z3463 (829/12)	NEG	NEG	NEG	AMB	NEG	POS	NEG	NEG	POS	POS	NEG	NEG	NEG	NEG	AMB	NEG	AMB	NEG	NEG	NEG	AMB	NEG	NEG	NEG	NEG	NEG	NEG	NEG	POS	NEG	NEG	NEG
2012_269_Z3556 (3769A/12)	NEG	NEG	NEG	POS	NEG	AMB	NEG	NEG	POS	POS	NEG	NEG	NEG	NEG	NEG	NEG	NEG	NEG	NEG	NEG	NEG	NEG	NEG	NEG	NEG	NEG	NEG	NEG	POS	NEG	NEG	NEG
2012_292_Z3579	NEG	NEG	NEG	AMB	NEG	POS	NEG	NEG	AMB	POS	NEG	NEG	NEG	NEG	NEG	NEG	NEG	NEG	NEG	NEG	NEG	NEG	NEG	NEG	NEG	NEG	NEG	NEG	POS	NEG	NEG	NEG
2012_299_Z3586 (5487/12)	NEG	NEG	NEG	AMB	NEG	POS	NEG	NEG	AMB	POS	NEG	NEG	NEG	NEG	NEG	NEG	NEG	NEG	NEG	NEG	NEG	NEG	NEG	NEG	NEG	NEG	NEG	NEG	POS	NEG	NEG	NEG
2012_301_Z3588 (5717/12)	NEG	NEG	NEG	AMB	NEG	POS	NEG	NEG	AMB	POS	NEG	NEG	NEG	NEG	NEG	NEG	NEG	NEG	NEG	NEG	NEG	NEG	NEG	NEG	NEG	NEG	NEG	NEG	POS	NEG	NEG	NEG
**ST2596dlv-MSSA**																																
2012_310_Z3597 (6034/12)	NEG	NEG	NEG	POS	NEG	AMB	NEG	NEG	NEG	NEG	NEG	NEG	NEG	NEG	NEG	NEG	AMB	NEG	NEG	POS	POS	POS	POS	NEG	NEG	NEG	NEG	NEG	POS	NEG	NEG	NEG

Strain / Isolates	bone sialoprotein-binding protein							clumping factor A					clumping factor B			
	bbp (total)	bbp (consensus)	bbp (COL+MW2)	bbp (MRSA252)	bbp (Mu50)	bbp (RF122)	bbp (ST45)	clfA (total)	clfA (consensus)	clfA (COL+RF122)	clfA (MRSA252)	clfA (Mu50+MW2)	clfB (total)	clfB (consensus)	clfB (COL+Mu50)	clfB (MW2)
**CC1-MSSA**																
2012_257_Z3544 (3216/12)	POS	POS	POS	NEG	AMB	NEG	NEG	POS	POS	NEG	NEG	POS	POS	POS	NEG	POS
2012_281_Z3568 (4696/12)	POS	POS	POS	NEG	NEG	NEG	NEG	POS	POS	NEG	NEG	POS	POS	POS	NEG	POS
2012_324_Z3611 (7180/12)	POS	POS	POS	NEG	AMB	NEG	NEG	POS	POS	NEG	NEG	POS	POS	POS	NEG	AMB
2012_327_Z3614 (7347/12)	POS	POS	POS	NEG	AMB	NEG	NEG	POS	POS	NEG	NEG	POS	POS	POS	NEG	POS
2012_351_Z3638 (8273B/12)	POS	POS	POS	NEG	AMB	NEG	NEG	POS	POS	NEG	NEG	POS	POS	POS	NEG	AMB
**CC5-MSSA**																
2012_188_Z3505 (503/12)	POS	POS	AMB	NEG	POS	NEG	NEG	POS	POS	NEG	NEG	POS	POS	POS	POS	NEG
2012_219_Z3518 (1437/12)	POS	POS	AMB	NEG	POS	NEG	NEG	POS	POS	NEG	NEG	POS	POS	POS	POS	NEG
2012_329_Z3616 (7577/12)	POS	POS	AMB	NEG	POS	NEG	AMB	POS	POS	NEG	NEG	POS	POS	POS	POS	NEG
2012_377_Z3437 (6242)	POS	POS	POS	NEG	AMB	NEG	NEG	POS	POS	NEG	NEG	POS	POS	POS	POS	NEG
2013_PA04_Z3987	POS	POS	NEG	NEG	POS	NEG	NEG	POS	POS	NEG	NEG	POS	POS	POS	POS	NEG
2013_PA32_Z4015	POS	POS	AMB	NEG	POS	NEG	AMB	POS	POS	NEG	NEG	POS	POS	POS	POS	NEG
2013_PA41_Z4024	POS	POS	NEG	NEG	POS	NEG	NEG	POS	POS	NEG	NEG	POS	POS	POS	POS	NEG
2013_PA44_Z4027	POS	POS	NEG	NEG	POS	NEG	NEG	POS	POS	NEG	NEG	POS	POS	POS	POS	NEG
**CC5-MSSA [PVL+]**																
2012_175_Z3497 (112/12)	POS	POS	AMB	NEG	POS	NEG	NEG	POS	POS	NEG	NEG	POS	POS	POS	POS	NEG
2012_193_Z3458 (617/12)	POS	POS	POS	NEG	AMB	NEG	NEG	POS	POS	NEG	NEG	POS	POS	POS	POS	NEG
2012_215_Z3474 (1341/12)	POS	POS	AMB	NEG	POS	NEG	NEG	POS	POS	NEG	NEG	POS	POS	POS	POS	NEG
2012_216_Z3475 (1370/12)	POS	POS	AMB	NEG	POS	NEG	NEG	POS	POS	NEG	NEG	POS	POS	POS	POS	NEG
2012_297_Z3584 (5140/12)	POS	POS	NEG	NEG	POS	NEG	NEG	POS	POS	NEG	NEG	POS	POS	POS	POS	NEG
2012_331_Z3618 (7644/12)	POS	POS	AMB	NEG	POS	NEG	NEG	POS	POS	NEG	NEG	POS	POS	POS	POS	NEG
2012_379_Z3439 (4126)	POS	POS	AMB	NEG	POS	NEG	NEG	POS	POS	NEG	NEG	POS	POS	POS	POS	NEG
2012_381_Z3441	POS	POS	AMB	NEG	POS	NEG	NEG	POS	POS	NEG	NEG	POS	POS	POS	POS	AMB
2013_PA39_Z4022	POS	POS	NEG	NEG	POS	NEG	NEG	POS	POS	NEG	NEG	POS	POS	POS	POS	NEG
2013_PA47_Z4030	POS	POS	NEG	NEG	POS	NEG	NEG	POS	POS	NEG	NEG	POS	POS	POS	POS	NEG
2013_PA61_I_Z4044	POS	POS	AMB	NEG	POS	NEG	NEG	POS	POS	NEG	NEG	POS	POS	POS	POS	NEG
2013_PA62_I_Z4045	POS	POS	NEG	NEG	POS	NEG	NEG	POS	POS	NEG	NEG	POS	POS	POS	POS	NEG
2013_PA74_Z4057	POS	POS	NEG	NEG	POS	NEG	NEG	POS	POS	NEG	NEG	POS	POS	POS	POS	NEG
2013_PA75_Z4058	POS	POS	NEG	NEG	POS	NEG	NEG	POS	POS	NEG	NEG	POS	POS	POS	POS	NEG
**CC5-MRSA-V [ACME+]**																
2012_373_Z3433 (1204-17312)	POS	POS	AMB	NEG	POS	NEG	NEG	POS	POS	NEG	NEG	POS	POS	POS	POS	NEG
**CC6-MSSA**																
2012_173_Z3450 (98A/12)	POS	POS	POS	NEG	AMB	AMB	NEG	POS	POS	NEG	NEG	POS	POS	POS	AMB	NEG
2012_176_Z3498	POS	POS	POS	NEG	NEG	NEG	NEG	POS	POS	NEG	NEG	POS	POS	POS	NEG	NEG
2012_181_Z3452	POS	POS	POS	NEG	AMB	NEG	NEG	POS	POS	NEG	NEG	POS	POS	POS	NEG	NEG
2012_259_Z3546 (3279/12)	POS	POS	POS	NEG	AMB	NEG	AMB	POS	POS	NEG	NEG	POS	POS	POS	NEG	NEG
2012_264_Z3551 (3407B/12)	POS	POS	POS	NEG	AMB	NEG	NEG	POS	POS	NEG	NEG	POS	POS	POS	AMB	NEG
2012_273_Z3560 (3844/12)	POS	POS	POS	NEG	AMB	NEG	NEG	POS	POS	NEG	NEG	POS	POS	POS	AMB	NEG
2012_312_Z3599 (6815/12)	POS	POS	POS	NEG	AMB	NEG	NEG	POS	POS	NEG	NEG	POS	POS	POS	NEG	NEG
2012_338_Z3625	POS	POS	POS	NEG	NEG	NEG	NEG	POS	POS	NEG	NEG	POS	POS	POS	NEG	NEG
2012_352_Z3639	POS	POS	POS	NEG	AMB	NEG	NEG	POS	POS	NEG	NEG	POS	POS	POS	NEG	NEG
2012_368_Z3428	POS	POS	POS	NEG	AMB	NEG	AMB	POS	POS	NEG	NEG	POS	POS	POS	NEG	NEG
2013_PA12_Z3995	POS	POS	POS	NEG	AMB	NEG	NEG	POS	POS	NEG	NEG	POS	POS	POS	NEG	NEG
2013_PA28_Z4011	POS	POS	POS	NEG	AMB	NEG	NEG	POS	POS	NEG	NEG	POS	POS	POS	NEG	NEG
**CC6-MSSA [PVL+]**																
2012_375_Z3435	POS	POS	POS	NEG	AMB	NEG	NEG	POS	POS	NEG	NEG	POS	POS	POS	AMB	NEG
2013_PA77_Z4060	POS	POS	POS	NEG	AMB	NEG	NEG	POS	POS	NEG	NEG	POS	POS	POS	NEG	NEG
**CC7-MSSA**																
2012_248_Z3532 (2864/12)	POS	POS	POS	AMB	NEG	NEG	NEG	POS	POS	POS	AMB	AMB	POS	POS	NEG	NEG
2012_265_Z3552 (3518/12)	POS	POS	POS	AMB	NEG	NEG	NEG	POS	POS	AMB	POS	AMB	POS	POS	NEG	NEG
2013_PA53_Z4036	POS	POS	POS	AMB	NEG	NEG	NEG	POS	POS	POS	AMB	AMB	POS	POS	NEG	NEG
2013_PA83_Z4066	POS	POS	POS	AMB	NEG	NEG	NEG	POS	POS	AMB	AMB	POS	POS	POS	NEG	NEG
**CC8-MSSA**																
2012_243_Z3528 (2587/12)	POS	POS	POS	NEG	NEG	NEG	NEG	POS	POS	AMB	AMB	POS	POS	POS	POS	NEG
2012_344_Z3631 (8605/12)	POS	POS	POS	NEG	AMB	NEG	NEG	POS	POS	AMB	POS	AMB	POS	POS	POS	NEG
2012_346_Z3633 (8074/12)	POS	POS	POS	NEG	AMB	NEG	NEG	POS	POS	AMB	POS	AMB	POS	POS	POS	NEG
2012_255_Z3538 (3205B/12)	POS	POS	POS	NEG	NEG	NEG	NEG	POS	POS	AMB	POS	AMB	POS	POS	POS	NEG
2012_280_Z3567 (4601/12)	POS	POS	POS	NEG	AMB	NEG	NEG	POS	POS	AMB	POS	AMB	POS	POS	POS	NEG
Trinidad&Tobago_2012_267 (3642/12)	POS	POS	POS	NEG	AMB	NEG	NEG	POS	POS	POS	AMB	AMB	POS	POS	POS	NEG
2012_365_Z3425 (1204-15838)	POS	POS	POS	NEG	AMB	NEG	NEG	POS	POS	AMB	POS	AMB	POS	POS	POS	NEG
2012_296_Z3583 (5118/12)	POS	POS	POS	NEG	AMB	NEG	NEG	POS	POS	AMB	AMB	POS	POS	POS	POS	NEG
2012_318_Z3605 (6960/12)	POS	POS	POS	NEG	AMB	NEG	NEG	POS	POS	AMB	POS	AMB	POS	POS	POS	NEG
2012_306_Z3593 (5861/12)	NEG	NEG	NEG	NEG	NEG	NEG	NEG	POS	POS	AMB	POS	AMB	POS	POS	POS	NEG
2013_PA37_Z4020	POS	POS	POS	NEG	AMB	NEG	NEG	POS	POS	AMB	AMB	POS	POS	POS	POS	NEG
2013_PA38_Z4021	POS	POS	POS	NEG	NEG	NEG	NEG	POS	POS	POS	NEG	AMB	POS	POS	POS	NEG
2013_PA52_Z4035	POS	POS	POS	NEG	NEG	NEG	NEG	POS	POS	POS	AMB	AMB	POS	POS	POS	NEG
**CC8-MSSA [PVL+]**																
2012_156_Z3489 (25917/11)	POS	POS	POS	NEG	AMB	NEG	NEG	POS	POS	AMB	AMB	POS	POS	POS	POS	NEG
2012_159_Z3492 (26310/11)	POS	POS	POS	NEG	AMB	NEG	NEG	POS	POS	AMB	POS	AMB	POS	POS	POS	NEG
2012_161_Z3445 (26430/11)	POS	POS	POS	NEG	AMB	NEG	NEG	POS	POS	AMB	POS	AMB	POS	POS	POS	AMB
2012_162_Z3512 (26433/11)	POS	POS	POS	NEG	AMB	NEG	NEG	POS	POS	AMB	POS	AMB	POS	POS	POS	NEG
2012_169_Z3495 (27194/11)	POS	POS	POS	NEG	AMB	NEG	NEG	POS	POS	AMB	POS	AMB	POS	POS	POS	NEG
2012_170_Z3496 (35/12)	POS	POS	POS	NEG	AMB	NEG	NEG	POS	POS	AMB	POS	AMB	POS	POS	POS	NEG
2012_171_Z3448 (47/12)	POS	POS	POS	NEG	AMB	NEG	NEG	POS	POS	AMB	POS	AMB	POS	POS	POS	NEG
2012_177_Z3499 (184/12)	POS	POS	POS	NEG	NEG	NEG	NEG	POS	POS	AMB	POS	AMB	POS	POS	POS	NEG
2012_179_Z3515 (231/12)	POS	POS	POS	NEG	AMB	NEG	NEG	POS	POS	AMB	POS	AMB	POS	POS	POS	NEG
2012_185_Z3502 (437/12)	POS	POS	POS	NEG	NEG	NEG	NEG	POS	POS	AMB	AMB	POS	POS	POS	POS	NEG
2012_191_Z3456 (563/12)	POS	POS	POS	NEG	NEG	NEG	NEG	POS	POS	AMB	POS	AMB	POS	POS	POS	NEG
2012_194_Z3459	POS	POS	POS	NEG	NEG	NEG	NEG	POS	POS	POS	AMB	AMB	POS	POS	POS	NEG
2012_200_Z3460 (820/12)	POS	POS	POS	NEG	AMB	AMB	AMB	POS	POS	AMB	POS	AMB	POS	POS	POS	NEG
2012_207_Z3467 (1010/12)	POS	POS	POS	NEG	NEG	NEG	NEG	POS	POS	AMB	POS	AMB	POS	POS	POS	NEG
2012_212_Z3471 (1261/12)	POS	POS	POS	NEG	NEG	NEG	NEG	POS	POS	AMB	AMB	POS	POS	POS	POS	NEG
2012_220_Z3477 (1466/12)	POS	POS	POS	NEG	NEG	NEG	NEG	POS	POS	AMB	AMB	POS	POS	POS	POS	NEG
2012_224_Z3481 (1522/12)	POS	POS	POS	NEG	NEG	NEG	NEG	POS	POS	AMB	AMB	POS	POS	POS	POS	NEG
2012_226_Z3483 (1662/12)	POS	POS	POS	NEG	AMB	NEG	NEG	POS	POS	AMB	AMB	POS	POS	POS	POS	NEG
2012_242_Z3527 (2581/12)	POS	POS	POS	NEG	AMB	NEG	NEG	POS	POS	AMB	POS	AMB	POS	POS	POS	NEG
2012_245_Z3529 (2687/12)	POS	POS	POS	NEG	NEG	NEG	NEG	POS	POS	AMB	POS	AMB	POS	POS	POS	NEG
2012_253_Z3536 (3074/12)	POS	POS	POS	NEG	NEG	NEG	NEG	POS	POS	AMB	AMB	POS	POS	POS	POS	NEG
2012_258_Z3545 (3231/12)	POS	POS	POS	NEG	AMB	NEG	AMB	POS	POS	AMB	POS	AMB	POS	POS	POS	NEG
2012_261_Z3548 (3298/12)	POS	POS	POS	NEG	AMB	NEG	NEG	POS	POS	AMB	POS	AMB	POS	POS	AMB	AMB
2012_270_Z3557 (3798/12)	POS	POS	POS	NEG	AMB	NEG	NEG	POS	POS	AMB	AMB	POS	POS	POS	POS	NEG
2012_274_Z3561 (3861/12)	POS	POS	POS	NEG	AMB	NEG	NEG	POS	POS	AMB	AMB	POS	POS	POS	POS	NEG
2012_282_Z3569 (4698/12)	POS	POS	POS	NEG	NEG	NEG	NEG	POS	POS	AMB	POS	AMB	POS	POS	POS	NEG
2012_286_Z3573 (4805/12)	POS	POS	POS	NEG	NEG	NEG	NEG	POS	POS	AMB	POS	AMB	POS	POS	POS	NEG
2012_293_Z3580 (5007/12)	POS	POS	POS	NEG	AMB	NEG	NEG	POS	POS	AMB	POS	AMB	POS	POS	POS	NEG
2012_294_Z3581	POS	POS	POS	NEG	NEG	NEG	NEG	POS	POS	AMB	POS	AMB	POS	POS	POS	NEG
2012_307_Z3594 (5918/12)	POS	POS	POS	NEG	AMB	NEG	NEG	POS	POS	AMB	POS	AMB	POS	POS	POS	NEG
2012_333_Z3620 (8101/12)	POS	POS	POS	NEG	AMB	NEG	NEG	POS	POS	AMB	POS	AMB	POS	POS	POS	NEG
2012_343_Z3630 (8195/12)	POS	POS	POS	NEG	AMB	NEG	NEG	POS	POS	AMB	POS	AMB	POS	POS	POS	NEG
2012_349_Z3636 (7999A/12)	POS	POS	POS	NEG	AMB	NEG	NEG	POS	POS	AMB	POS	AMB	POS	POS	POS	NEG
2012_353_Z3640 (8575/12)	POS	POS	POS	NEG	AMB	NEG	NEG	POS	POS	AMB	POS	AMB	POS	POS	POS	NEG
2012_354_Z3641 (8408/12)	POS	POS	POS	NEG	AMB	AMB	NEG	POS	POS	AMB	POS	AMB	POS	POS	POS	NEG
2012_355_Z3642 (1112-58348)	POS	POS	POS	NEG	NEG	NEG	NEG	POS	POS	AMB	AMB	POS	POS	POS	POS	NEG
2012_357_Z3417 (1202-6360)	POS	POS	POS	NEG	AMB	NEG	NEG	POS	POS	AMB	POS	AMB	POS	POS	POS	NEG
2012_364_Z3424 (1112-60417)	POS	POS	POS	NEG	AMB	NEG	NEG	POS	POS	AMB	AMB	POS	POS	POS	POS	NEG
2012_376_Z3436 (6256)	POS	POS	POS	NEG	AMB	NEG	NEG	POS	POS	AMB	POS	AMB	POS	POS	POS	NEG
2012_383_Z3443	POS	POS	POS	NEG	AMB	AMB	NEG	POS	POS	AMB	AMB	POS	POS	POS	POS	AMB
2013_PA01_Z3984	POS	POS	POS	NEG	AMB	NEG	NEG	POS	POS	AMB	AMB	POS	POS	POS	POS	NEG
2013_PA08_Z3991	POS	POS	POS	NEG	AMB	NEG	NEG	POS	POS	POS	AMB	AMB	POS	POS	POS	NEG
2013_PA10_Z3993	POS	POS	POS	NEG	AMB	NEG	NEG	POS	POS	POS	AMB	AMB	POS	POS	POS	NEG
2013_PA13_Z3996	POS	POS	POS	NEG	AMB	NEG	NEG	POS	POS	POS	AMB	AMB	POS	POS	POS	NEG
2013_PA14_Z3997	POS	POS	POS	NEG	AMB	NEG	NEG	POS	POS	POS	AMB	AMB	POS	POS	POS	NEG
2013_PA17_Z4000	POS	POS	POS	NEG	AMB	NEG	NEG	POS	POS	POS	AMB	AMB	POS	POS	POS	NEG
2013_PA26_Z4009	POS	POS	POS	NEG	NEG	NEG	NEG	POS	POS	AMB	AMB	POS	POS	POS	POS	NEG
2013_PA27_Z4010	POS	POS	POS	NEG	AMB	NEG	NEG	POS	POS	AMB	AMB	POS	POS	POS	POS	NEG
2013_PA42_Z4025	POS	POS	POS	NEG	AMB	NEG	NEG	POS	POS	POS	AMB	AMB	POS	POS	POS	NEG
2013_PA43_Z4026	POS	POS	POS	NEG	NEG	NEG	NEG	POS	POS	POS	AMB	AMB	POS	POS	POS	NEG
2013_PA45_Z4028	POS	POS	POS	NEG	AMB	NEG	NEG	POS	POS	POS	AMB	AMB	POS	POS	POS	NEG
2013_PA56_Z4039	POS	POS	POS	NEG	AMB	NEG	NEG	POS	POS	POS	AMB	AMB	POS	POS	POS	NEG
2013_PA58_Z4041	POS	POS	POS	NEG	AMB	NEG	NEG	POS	POS	AMB	AMB	POS	POS	POS	POS	NEG
2013_PA64_Z4047	POS	POS	POS	NEG	AMB	NEG	NEG	POS	POS	AMB	AMB	POS	POS	POS	POS	NEG
2013_PA66_Z4049	POS	POS	POS	NEG	AMB	NEG	NEG	POS	POS	AMB	AMB	POS	POS	POS	POS	NEG
2013_PA67_Z4050	POS	POS	POS	NEG	AMB	NEG	NEG	POS	POS	AMB	AMB	POS	POS	POS	POS	NEG
2013_PA69_Z4052	POS	POS	POS	NEG	AMB	NEG	NEG	POS	POS	AMB	AMB	POS	POS	POS	POS	NEG
2013_PA72_Z4055	POS	POS	POS	NEG	AMB	NEG	NEG	POS	POS	AMB	AMB	POS	POS	POS	POS	NEG
2013_PA76_Z4059	POS	POS	POS	NEG	AMB	NEG	NEG	POS	POS	AMB	AMB	POS	POS	POS	POS	NEG
2013_PA80_Z4063	POS	POS	POS	NEG	AMB	NEG	NEG	POS	POS	POS	AMB	AMB	POS	POS	POS	NEG

Strain / Isolates	clumping factor B	collagen-binding adhesin	cell wall associated fibronectin-binding protein	cell surface elastin binding protein					enolase	fibrinogen binding protein (19 kDa)		fibronectin-binding protein A					
	clfB (RF122)	cna	ebh (consensus)	ebpS (total)	ebpS_probe 612	ebpS_probe 614	ebpS (01-1111)	ebpS (COL)	eno	fib	fib (MRSA252)	fnbA (total)	fnbA (consensus)	fnbA (COL)	fnbA (MRSA252)	fnbA (Mu50+MW2)	fnbA (RF122)
**CC1-MSSA**																	
2012_257_Z3544 (3216/12)	AMB	POS	POS	POS	POS	POS	NEG	NEG	POS	POS	NEG	POS	POS	NEG	NEG	POS	NEG
2012_281_Z3568 (4696/12)	AMB	POS	POS	POS	POS	POS	NEG	NEG	POS	POS	NEG	POS	POS	NEG	NEG	POS	NEG
2012_324_Z3611 (7180/12)	POS	POS	POS	POS	POS	POS	NEG	NEG	POS	POS	NEG	POS	POS	NEG	NEG	POS	NEG
2012_327_Z3614 (7347/12)	AMB	POS	POS	POS	POS	POS	NEG	NEG	POS	POS	NEG	POS	POS	NEG	NEG	POS	NEG
2012_351_Z3638 (8273B/12)	POS	POS	POS	POS	POS	POS	NEG	NEG	POS	POS	NEG	POS	POS	NEG	NEG	POS	NEG
**CC5-MSSA**																	
2012_188_Z3505 (503/12)	NEG	NEG	POS	POS	POS	POS	NEG	NEG	POS	POS	NEG	POS	POS	NEG	NEG	POS	NEG
2012_219_Z3518 (1437/12)	NEG	NEG	POS	POS	POS	POS	NEG	NEG	POS	POS	NEG	POS	POS	NEG	NEG	POS	NEG
2012_329_Z3616 (7577/12)	NEG	NEG	POS	POS	POS	POS	NEG	NEG	POS	POS	NEG	POS	POS	NEG	NEG	POS	NEG
2012_377_Z3437 (6242)	NEG	NEG	POS	POS	POS	POS	NEG	NEG	POS	POS	NEG	POS	POS	NEG	NEG	POS	NEG
2013_PA04_Z3987	NEG	NEG	POS	POS	POS	POS	NEG	NEG	POS	POS	NEG	POS	POS	NEG	NEG	POS	NEG
2013_PA32_Z4015	NEG	NEG	POS	POS	POS	POS	NEG	NEG	POS	POS	NEG	POS	POS	NEG	NEG	POS	NEG
2013_PA41_Z4024	NEG	NEG	POS	POS	POS	POS	NEG	NEG	POS	POS	NEG	POS	POS	NEG	NEG	POS	NEG
2013_PA44_Z4027	NEG	NEG	POS	POS	POS	POS	NEG	NEG	POS	POS	NEG	POS	POS	NEG	NEG	POS	NEG
**CC5-MSSA [PVL+]**																	
2012_175_Z3497 (112/12)	NEG	NEG	POS	POS	POS	POS	NEG	NEG	POS	POS	NEG	POS	POS	NEG	NEG	POS	NEG
2012_193_Z3458 (617/12)	NEG	NEG	POS	POS	POS	POS	NEG	NEG	POS	POS	NEG	POS	POS	NEG	NEG	POS	NEG
2012_215_Z3474 (1341/12)	NEG	NEG	POS	POS	POS	POS	NEG	NEG	POS	POS	NEG	POS	POS	NEG	NEG	POS	NEG
2012_216_Z3475 (1370/12)	NEG	NEG	POS	POS	POS	POS	NEG	NEG	POS	POS	NEG	POS	POS	NEG	NEG	POS	NEG
2012_297_Z3584 (5140/12)	NEG	NEG	POS	POS	POS	POS	NEG	NEG	POS	POS	NEG	POS	POS	NEG	NEG	POS	NEG
2012_331_Z3618 (7644/12)	NEG	NEG	POS	POS	POS	POS	NEG	NEG	POS	POS	AMB	POS	POS	NEG	NEG	POS	NEG
2012_379_Z3439 (4126)	NEG	NEG	POS	POS	POS	POS	NEG	NEG	POS	POS	NEG	POS	POS	NEG	NEG	POS	NEG
2012_381_Z3441	NEG	NEG	POS	POS	POS	POS	NEG	AMB	POS	POS	AMB	POS	POS	AMB	NEG	POS	NEG
2013_PA39_Z4022	NEG	NEG	POS	POS	POS	POS	NEG	NEG	POS	POS	NEG	POS	POS	NEG	NEG	POS	NEG
2013_PA47_Z4030	NEG	NEG	POS	POS	POS	POS	NEG	NEG	POS	POS	NEG	POS	POS	NEG	NEG	POS	NEG
2013_PA61_I_Z4044	NEG	NEG	POS	POS	POS	POS	NEG	NEG	POS	POS	NEG	POS	POS	NEG	NEG	POS	NEG
2013_PA62_I_Z4045	NEG	NEG	POS	POS	POS	POS	NEG	NEG	POS	POS	NEG	POS	POS	NEG	NEG	POS	NEG
2013_PA74_Z4057	NEG	NEG	POS	POS	POS	POS	NEG	NEG	POS	POS	NEG	POS	POS	NEG	NEG	POS	NEG
2013_PA75_Z4058	NEG	NEG	POS	POS	POS	POS	NEG	NEG	POS	POS	NEG	POS	POS	NEG	NEG	POS	NEG
**CC5-MRSA-V [ACME+]**																	
2012_373_Z3433 (1204-17312)	NEG	NEG	POS	POS	POS	POS	NEG	NEG	POS	POS	NEG	NEG	NEG	NEG	NEG	NEG	NEG
**CC6-MSSA**																	
2012_173_Z3450 (98A/12)	POS	POS	POS	POS	POS	POS	NEG	AMB	POS	POS	POS	POS	POS	NEG	POS	NEG	AMB
2012_176_Z3498	POS	POS	POS	POS	POS	POS	NEG	NEG	POS	POS	AMB	POS	POS	NEG	POS	NEG	NEG
2012_181_Z3452	POS	POS	POS	POS	POS	POS	NEG	NEG	POS	POS	AMB	POS	POS	NEG	POS	NEG	NEG
2012_259_Z3546 (3279/12)	POS	POS	POS	POS	POS	POS	NEG	NEG	POS	POS	AMB	POS	POS	NEG	POS	NEG	NEG
2012_264_Z3551 (3407B/12)	POS	POS	POS	POS	POS	POS	NEG	NEG	POS	POS	AMB	POS	POS	NEG	POS	NEG	NEG
2012_273_Z3560 (3844/12)	POS	POS	POS	POS	POS	POS	NEG	NEG	POS	POS	AMB	POS	POS	NEG	POS	NEG	NEG
2012_312_Z3599 (6815/12)	POS	POS	POS	POS	POS	POS	NEG	NEG	POS	POS	NEG	POS	POS	NEG	POS	NEG	NEG
2012_338_Z3625	POS	POS	POS	POS	POS	POS	NEG	NEG	POS	POS	NEG	POS	POS	NEG	POS	NEG	NEG
2012_352_Z3639	POS	POS	POS	POS	POS	POS	NEG	NEG	POS	POS	NEG	POS	POS	NEG	POS	NEG	NEG
2012_368_Z3428	POS	POS	POS	POS	POS	POS	NEG	NEG	POS	POS	NEG	POS	POS	NEG	POS	NEG	NEG
2013_PA12_Z3995	POS	POS	POS	POS	POS	POS	NEG	NEG	POS	POS	NEG	POS	POS	NEG	POS	NEG	NEG
2013_PA28_Z4011	POS	POS	POS	POS	POS	POS	NEG	NEG	POS	POS	NEG	POS	POS	NEG	POS	NEG	NEG
**CC6-MSSA [PVL+]**																	
2012_375_Z3435	POS	POS	POS	POS	POS	POS	NEG	NEG	POS	POS	AMB	POS	POS	NEG	POS	NEG	NEG
2013_PA77_Z4060	POS	POS	POS	POS	POS	POS	NEG	NEG	POS	POS	NEG	POS	POS	NEG	POS	NEG	NEG
**CC7-MSSA**																	
2012_248_Z3532 (2864/12)	POS	NEG	POS	POS	POS	POS	NEG	POS	POS	POS	AMB	POS	POS	NEG	NEG	NEG	NEG
2012_265_Z3552 (3518/12)	POS	AMB	POS	POS	POS	POS	NEG	POS	POS	POS	AMB	POS	POS	NEG	POS	NEG	NEG
2013_PA53_Z4036	POS	NEG	POS	POS	POS	POS	NEG	POS	POS	POS	NEG	POS	POS	NEG	NEG	NEG	NEG
2013_PA83_Z4066	POS	NEG	POS	POS	POS	POS	NEG	POS	POS	POS	NEG	POS	POS	NEG	NEG	NEG	NEG
**CC8-MSSA**																	
2012_243_Z3528 (2587/12)	NEG	NEG	POS	POS	POS	POS	NEG	POS	POS	POS	AMB	POS	POS	POS	NEG	NEG	NEG
2012_344_Z3631 (8605/12)	NEG	NEG	POS	POS	POS	POS	NEG	POS	POS	POS	AMB	POS	POS	POS	NEG	NEG	NEG
2012_346_Z3633 (8074/12)	NEG	NEG	POS	POS	POS	POS	NEG	POS	POS	POS	AMB	POS	POS	POS	NEG	NEG	NEG
2012_255_Z3538 (3205B/12)	NEG	NEG	POS	POS	POS	POS	NEG	POS	POS	POS	AMB	POS	POS	POS	NEG	NEG	NEG
2012_280_Z3567 (4601/12)	NEG	NEG	POS	POS	POS	POS	NEG	POS	POS	POS	NEG	POS	POS	POS	NEG	NEG	NEG
Trinidad&Tobago_2012_267 (3642/12)	NEG	NEG	POS	POS	POS	POS	NEG	POS	POS	POS	NEG	POS	POS	POS	NEG	NEG	NEG
2012_365_Z3425 (1204-15838)	NEG	NEG	POS	POS	POS	POS	NEG	POS	POS	POS	AMB	POS	POS	POS	NEG	NEG	NEG
2012_296_Z3583 (5118/12)	NEG	NEG	POS	POS	POS	POS	NEG	POS	POS	POS	NEG	POS	POS	POS	NEG	NEG	NEG
2012_318_Z3605 (6960/12)	NEG	NEG	POS	POS	POS	POS	NEG	POS	POS	POS	NEG	POS	POS	POS	NEG	NEG	NEG
2012_306_Z3593 (5861/12)	NEG	NEG	POS	POS	POS	POS	NEG	POS	POS	POS	AMB	POS	POS	POS	NEG	NEG	NEG
2013_PA37_Z4020	NEG	NEG	POS	POS	POS	POS	NEG	POS	POS	POS	NEG	POS	POS	POS	NEG	NEG	NEG
2013_PA38_Z4021	NEG	NEG	POS	POS	POS	POS	NEG	AMB	POS	POS	NEG	POS	POS	POS	NEG	NEG	NEG
2013_PA52_Z4035	NEG	NEG	POS	POS	POS	POS	NEG	POS	POS	POS	NEG	POS	POS	POS	NEG	NEG	NEG
**CC8-MSSA [PVL+]**																	
2012_156_Z3489 (25917/11)	NEG	NEG	POS	POS	POS	POS	NEG	POS	POS	POS	NEG	POS	POS	POS	NEG	NEG	NEG
2012_159_Z3492 (26310/11)	NEG	NEG	POS	POS	POS	POS	NEG	POS	POS	POS	NEG	POS	POS	POS	NEG	NEG	NEG
2012_161_Z3445 (26430/11)	NEG	NEG	POS	POS	POS	POS	NEG	POS	POS	POS	POS	POS	POS	POS	NEG	NEG	NEG
2012_162_Z3512 (26433/11)	NEG	NEG	POS	POS	POS	POS	NEG	POS	POS	POS	NEG	POS	POS	POS	NEG	NEG	NEG
2012_169_Z3495 (27194/11)	NEG	NEG	POS	POS	POS	POS	NEG	POS	POS	POS	AMB	POS	POS	POS	NEG	NEG	NEG
2012_170_Z3496 (35/12)	NEG	NEG	POS	POS	POS	POS	NEG	POS	POS	POS	NEG	POS	POS	POS	NEG	NEG	NEG
2012_171_Z3448 (47/12)	NEG	NEG	POS	POS	POS	POS	NEG	POS	POS	POS	NEG	POS	POS	POS	NEG	NEG	NEG
2012_177_Z3499 (184/12)	NEG	NEG	POS	POS	POS	POS	NEG	POS	POS	POS	AMB	POS	POS	POS	NEG	NEG	NEG
2012_179_Z3515 (231/12)	NEG	NEG	POS	POS	POS	POS	NEG	POS	POS	POS	AMB	POS	POS	POS	NEG	NEG	NEG
2012_185_Z3502 (437/12)	NEG	NEG	POS	POS	POS	POS	NEG	POS	POS	POS	NEG	POS	POS	POS	NEG	NEG	NEG
2012_191_Z3456 (563/12)	NEG	NEG	POS	POS	POS	POS	NEG	POS	POS	POS	AMB	POS	POS	POS	NEG	NEG	NEG
2012_194_Z3459	NEG	NEG	POS	POS	POS	POS	NEG	POS	POS	POS	AMB	POS	POS	POS	NEG	NEG	NEG
2012_200_Z3460 (820/12)	NEG	NEG	POS	POS	POS	POS	NEG	POS	POS	POS	NEG	POS	POS	POS	NEG	NEG	NEG
2012_207_Z3467 (1010/12)	NEG	NEG	POS	POS	POS	POS	NEG	POS	POS	POS	AMB	POS	POS	POS	NEG	NEG	NEG
2012_212_Z3471 (1261/12)	NEG	NEG	POS	POS	POS	POS	NEG	POS	POS	POS	NEG	POS	POS	POS	NEG	NEG	NEG
2012_220_Z3477 (1466/12)	NEG	NEG	POS	POS	POS	POS	NEG	POS	POS	POS	NEG	POS	POS	POS	NEG	NEG	NEG
2012_224_Z3481 (1522/12)	NEG	NEG	POS	POS	POS	POS	NEG	POS	POS	POS	NEG	POS	POS	POS	NEG	NEG	NEG
2012_226_Z3483 (1662/12)	NEG	NEG	POS	POS	POS	POS	NEG	POS	POS	POS	NEG	POS	POS	POS	NEG	NEG	NEG
2012_242_Z3527 (2581/12)	NEG	NEG	POS	POS	POS	POS	NEG	POS	POS	POS	NEG	POS	POS	POS	NEG	NEG	NEG
2012_245_Z3529 (2687/12)	NEG	NEG	POS	POS	POS	POS	NEG	POS	POS	POS	AMB	POS	POS	POS	NEG	NEG	NEG
2012_253_Z3536 (3074/12)	NEG	NEG	POS	POS	POS	POS	NEG	POS	POS	POS	AMB	POS	POS	POS	NEG	NEG	NEG
2012_258_Z3545 (3231/12)	NEG	NEG	POS	POS	POS	POS	NEG	POS	POS	POS	AMB	POS	POS	POS	NEG	NEG	NEG
2012_261_Z3548 (3298/12)	POS	POS	POS	POS	POS	POS	NEG	POS	POS	POS	NEG	POS	POS	POS	NEG	NEG	NEG
2012_270_Z3557 (3798/12)	NEG	NEG	POS	POS	POS	POS	NEG	POS	POS	POS	AMB	POS	POS	POS	NEG	NEG	NEG
2012_274_Z3561 (3861/12)	NEG	NEG	POS	POS	POS	POS	NEG	POS	POS	POS	NEG	POS	POS	POS	NEG	NEG	NEG
2012_282_Z3569 (4698/12)	NEG	NEG	POS	POS	POS	POS	NEG	POS	POS	POS	AMB	POS	POS	POS	NEG	NEG	NEG
2012_286_Z3573 (4805/12)	NEG	NEG	POS	POS	POS	POS	NEG	POS	POS	POS	NEG	POS	POS	POS	NEG	NEG	NEG
2012_293_Z3580 (5007/12)	NEG	NEG	POS	POS	POS	POS	NEG	POS	POS	POS	NEG	POS	POS	POS	NEG	NEG	NEG
2012_294_Z3581	NEG	NEG	POS	POS	POS	POS	NEG	POS	POS	POS	NEG	POS	POS	POS	NEG	NEG	NEG
2012_307_Z3594 (5918/12)	NEG	NEG	POS	POS	POS	POS	NEG	POS	POS	POS	AMB	POS	POS	POS	NEG	NEG	NEG
2012_333_Z3620 (8101/12)	NEG	NEG	POS	POS	POS	POS	NEG	POS	POS	POS	NEG	POS	POS	POS	NEG	NEG	NEG
2012_343_Z3630 (8195/12)	NEG	NEG	POS	POS	POS	POS	NEG	POS	POS	POS	AMB	POS	POS	POS	NEG	NEG	NEG
2012_349_Z3636 (7999A/12)	NEG	NEG	POS	POS	POS	POS	NEG	POS	POS	POS	AMB	POS	POS	POS	NEG	NEG	NEG
2012_353_Z3640 (8575/12)	NEG	NEG	POS	POS	POS	POS	NEG	POS	POS	POS	AMB	POS	POS	POS	NEG	NEG	NEG
2012_354_Z3641 (8408/12)	NEG	NEG	POS	POS	POS	POS	NEG	POS	POS	POS	AMB	POS	POS	POS	NEG	NEG	NEG
2012_355_Z3642 (1112-58348)	NEG	NEG	POS	POS	POS	POS	NEG	POS	POS	POS	NEG	POS	POS	POS	NEG	NEG	NEG
2012_357_Z3417 (1202-6360)	NEG	NEG	POS	POS	POS	POS	NEG	POS	POS	POS	AMB	POS	POS	POS	NEG	NEG	NEG
2012_364_Z3424 (1112-60417)	NEG	NEG	POS	POS	POS	POS	NEG	POS	POS	POS	NEG	POS	POS	POS	NEG	NEG	NEG
2012_376_Z3436 (6256)	NEG	NEG	POS	POS	POS	POS	NEG	POS	POS	POS	AMB	POS	POS	POS	NEG	NEG	NEG
2012_383_Z3443	AMB	AMB	POS	POS	POS	POS	NEG	POS	POS	POS	NEG	POS	POS	POS	NEG	NEG	NEG
2013_PA01_Z3984	NEG	NEG	POS	POS	POS	POS	NEG	POS	POS	POS	NEG	POS	POS	POS	NEG	NEG	NEG
2013_PA08_Z3991	NEG	NEG	POS	POS	POS	POS	NEG	POS	POS	POS	NEG	POS	POS	POS	NEG	NEG	NEG
2013_PA10_Z3993	NEG	NEG	POS	POS	POS	POS	NEG	POS	POS	POS	NEG	POS	POS	POS	NEG	NEG	NEG
2013_PA13_Z3996	NEG	NEG	POS	POS	POS	POS	NEG	POS	POS	POS	NEG	POS	POS	POS	NEG	NEG	NEG
2013_PA14_Z3997	NEG	NEG	POS	POS	POS	POS	NEG	POS	POS	POS	NEG	POS	POS	POS	NEG	NEG	NEG
2013_PA17_Z4000	NEG	NEG	POS	POS	POS	POS	NEG	POS	POS	POS	NEG	POS	POS	POS	NEG	NEG	NEG
2013_PA26_Z4009	NEG	NEG	POS	POS	POS	POS	NEG	POS	POS	POS	NEG	POS	POS	POS	NEG	NEG	NEG
2013_PA27_Z4010	NEG	NEG	POS	POS	POS	POS	NEG	POS	POS	POS	NEG	POS	POS	POS	NEG	NEG	NEG
2013_PA42_Z4025	NEG	NEG	POS	POS	POS	POS	NEG	POS	POS	POS	NEG	POS	POS	POS	NEG	NEG	NEG
2013_PA43_Z4026	NEG	NEG	POS	POS	POS	POS	NEG	POS	POS	POS	NEG	POS	POS	POS	NEG	NEG	NEG
2013_PA45_Z4028	NEG	NEG	POS	POS	POS	POS	NEG	POS	POS	POS	NEG	POS	POS	POS	NEG	NEG	NEG
2013_PA56_Z4039	NEG	NEG	POS	POS	POS	POS	NEG	POS	POS	POS	NEG	POS	POS	POS	NEG	NEG	NEG
2013_PA58_Z4041	NEG	NEG	POS	POS	POS	POS	NEG	POS	POS	POS	NEG	POS	POS	POS	NEG	NEG	NEG
2013_PA64_Z4047	NEG	NEG	POS	POS	POS	POS	NEG	POS	POS	POS	NEG	POS	POS	POS	NEG	NEG	NEG
2013_PA66_Z4049	NEG	NEG	POS	POS	POS	POS	NEG	POS	POS	POS	NEG	POS	POS	POS	NEG	NEG	NEG
2013_PA67_Z4050	NEG	NEG	POS	POS	POS	POS	NEG	POS	POS	POS	NEG	POS	POS	POS	NEG	NEG	NEG
2013_PA69_Z4052	NEG	NEG	POS	POS	POS	POS	NEG	POS	POS	POS	NEG	POS	POS	POS	NEG	NEG	NEG
2013_PA72_Z4055	NEG	NEG	POS	POS	POS	POS	NEG	POS	POS	POS	NEG	POS	POS	POS	NEG	NEG	NEG
2013_PA76_Z4059	NEG	NEG	POS	POS	POS	POS	NEG	POS	POS	POS	NEG	POS	POS	POS	NEG	NEG	NEG
2013_PA80_Z4063	NEG	NEG	POS	POS	POS	POS	NEG	POS	POS	POS	NEG	POS	POS	POS	NEG	NEG	NEG

Strain / Isolates	bone sialoprotein-binding protein							clumping factor A					clumping factor B				
	bbp (total)	bbp (consensus)	bbp (COL+MW2)	bbp (MRSA252)	bbp (Mu50)	bbp (RF122)	bbp (ST45)	clfA (total)	clfA (consensus)	clfA (COL+RF122)	clfA (MRSA252)	clfA (Mu50+MW2)	clfB (total)	clfB (consensus)	clfB (COL+Mu50)	clfB (MW2)	clfB (RF122)
**ST8-MRSA-IV, USA300**																	
2012_158_Z3491 (26049A/11)	POS	POS	POS	NEG	AMB	AMB	NEG	POS	POS	AMB	POS	AMB	POS	POS	POS	NEG	NEG
2012_165_Z3513 (26656/11)	POS	POS	POS	NEG	AMB	NEG	NEG	POS	POS	AMB	POS	AMB	POS	POS	POS	NEG	NEG
2012_214_Z3473 (1322/12)	POS	POS	POS	NEG	NEG	NEG	NEG	POS	POS	AMB	AMB	POS	POS	POS	POS	NEG	NEG
2012_227_Z3484 (1688/12)	POS	POS	POS	NEG	AMB	AMB	NEG	POS	POS	AMB	POS	AMB	POS	POS	POS	NEG	NEG
2012_263_Z3550 (3355/12)	POS	POS	POS	NEG	AMB	NEG	NEG	POS	POS	AMB	POS	AMB	POS	POS	POS	NEG	NEG
2012_266_Z3553	POS	POS	POS	NEG	AMB	NEG	NEG	POS	POS	AMB	POS	AMB	POS	POS	POS	NEG	NEG
2012_284_Z3571 (4729/12)	POS	POS	POS	NEG	AMB	NEG	NEG	POS	POS	AMB	POS	AMB	POS	POS	POS	NEG	NEG
2012_298_Z3585 (5203/12)	POS	POS	POS	NEG	AMB	NEG	NEG	POS	POS	AMB	POS	AMB	POS	POS	POS	NEG	NEG
2012_304_Z3591 (5818/12)	POS	POS	POS	NEG	AMB	NEG	NEG	POS	POS	AMB	POS	AMB	POS	POS	POS	NEG	NEG
Z3595_TT308 - {EFC74B77-3925-484F-A4CF-A1F7DC	POS	POS	POS	NEG	AMB	NEG	NEG	POS	POS	AMB	AMB	POS	POS	POS	POS	NEG	NEG
2012_369_Z3429 (1112-58547)	POS	POS	POS	NEG	AMB	NEG	NEG	POS	POS	AMB	AMB	POS	POS	POS	POS	NEG	NEG
2012_374_Z3434 (1203-13527)	POS	POS	POS	NEG	NEG	NEG	NEG	POS	POS	AMB	POS	AMB	POS	POS	POS	NEG	NEG
2013_PA06_Z3989	POS	POS	POS	NEG	AMB	NEG	NEG	POS	POS	POS	AMB	AMB	POS	POS	POS	NEG	NEG
2013_PA55_Z4038	POS	POS	POS	NEG	AMB	NEG	NEG	POS	POS	AMB	AMB	POS	POS	POS	POS	NEG	NEG
2013_PA71_Z4054	POS	POS	POS	NEG	AMB	NEG	NEG	POS	POS	POS	AMB	AMB	POS	POS	POS	NEG	NEG
**ST8-MRSA-IV [PVL+/ACME+], US**																	
2012_180_Z3501 (232/12)	POS	POS	POS	NEG	NEG	NEG	NEG	POS	POS	AMB	AMB	POS	POS	POS	POS	NEG	NEG
2013_PA68_Z4051	POS	POS	POS	NEG	AMB	NEG	NEG	POS	POS	POS	AMB	AMB	POS	POS	POS	NEG	NEG
**ST72-MSSA**																	
2012_174_Z3451 (102/12)	POS	POS	POS	NEG	AMB	NEG	NEG	POS	POS	POS	AMB	AMB	POS	POS	NEG	NEG	POS
2012_223_Z3480 (1506A/12)	POS	POS	AMB	NEG	POS	NEG	NEG	POS	POS	AMB	AMB	POS	POS	POS	NEG	NEG	POS
2012_236_Z3523 (2086/12)	POS	POS	AMB	NEG	POS	NEG	NEG	POS	POS	AMB	POS	AMB	POS	POS	NEG	NEG	POS
2012_239_Z3525 (2346/12)	POS	POS	AMB	NEG	POS	NEG	NEG	POS	POS	AMB	POS	AMB	POS	POS	NEG	NEG	POS
2012_252_Z3535 (3030/12)	POS	POS	AMB	NEG	POS	NEG	NEG	POS	POS	AMB	POS	AMB	POS	POS	NEG	NEG	POS
2012_271_Z3558 (3799/12)	POS	POS	AMB	NEG	POS	NEG	NEG	POS	POS	AMB	POS	AMB	POS	POS	NEG	NEG	POS
2012_276_Z3563	POS	POS	AMB	NEG	POS	NEG	NEG	POS	POS	AMB	AMB	POS	POS	POS	NEG	NEG	POS
2012_300_Z3587 (5638/12)	POS	POS	NEG	NEG	POS	NEG	NEG	POS	POS	AMB	POS	AMB	POS	POS	NEG	NEG	POS
2012_316_Z3603 (6941/12)	POS	POS	AMB	NEG	POS	NEG	NEG	POS	POS	AMB	POS	AMB	POS	POS	NEG	NEG	POS
2012_319_Z3606 (7019/12)	POS	POS	AMB	NEG	POS	NEG	NEG	POS	POS	AMB	POS	AMB	POS	POS	NEG	NEG	POS
2012_371_Z3431 (1204-17577)	POS	POS	AMB	NEG	POS	NEG	NEG	POS	POS	AMB	POS	AMB	POS	POS	NEG	NEG	POS
2013_PA05_Z3988	POS	POS	NEG	NEG	POS	NEG	NEG	POS	POS	POS	AMB	AMB	POS	POS	NEG	NEG	POS
2013_PA65_Z4048	POS	POS	NEG	NEG	POS	NEG	NEG	POS	POS	POS	AMB	AMB	POS	POS	NEG	NEG	NEG
2013_PA73_Z4056	POS	POS	AMB	NEG	POS	NEG	NEG	POS	POS	AMB	AMB	POS	POS	POS	NEG	NEG	POS
**ST72-MSSA-SCCfus**																	
2013_PA16_Z3999	POS	POS	NEG	NEG	POS	NEG	NEG	POS	POS	POS	AMB	AMB	POS	POS	NEG	NEG	POS
**ST72-MSSA-SCCfus/kdp**																	
2012_361_Z3421 (1201-1299)	POS	POS	AMB	NEG	POS	NEG	NEG	POS	POS	AMB	POS	AMB	POS	POS	NEG	NEG	POS
**ST239-MRSA-III&SCCmer**																	
2012_157_Z3490	POS	POS	POS	NEG	AMB	NEG	NEG	POS	POS	AMB	POS	AMB	POS	POS	NEG	NEG	POS
2012_168_Z3494 (27129/11)	POS	POS	POS	NEG	AMB	NEG	NEG	POS	POS	AMB	POS	AMB	POS	POS	NEG	NEG	POS
2012_195_Z3508 (619/12)	POS	POS	POS	NEG	NEG	NEG	NEG	POS	POS	AMB	AMB	POS	POS	POS	NEG	NEG	POS
2012_222_Z3479 (1489/12)	POS	POS	POS	NEG	NEG	NEG	NEG	POS	POS	AMB	AMB	POS	POS	POS	NEG	NEG	POS
2012_233_Z3540 (1943/12)	POS	POS	POS	NEG	AMB	NEG	NEG	POS	POS	POS	AMB	AMB	POS	POS	NEG	NEG	POS
2012_237_Z3541	POS	POS	POS	NEG	AMB	NEG	NEG	POS	POS	AMB	AMB	POS	POS	POS	NEG	NEG	POS
2012_262_Z3549	POS	POS	POS	NEG	AMB	NEG	NEG	POS	POS	POS	AMB	AMB	POS	POS	NEG	NEG	POS
2012_275_Z3562 (3893/12)	POS	POS	POS	NEG	AMB	NEG	NEG	POS	POS	AMB	POS	AMB	POS	POS	NEG	NEG	POS
2012_291_Z3578	POS	POS	POS	NEG	NEG	NEG	NEG	POS	POS	AMB	POS	AMB	POS	POS	NEG	NEG	POS
2012_314_Z3601 (6939/12)	POS	POS	POS	NEG	AMB	NEG	NEG	POS	POS	AMB	POS	AMB	POS	POS	AMB	NEG	POS
2012323_Z3610	POS	POS	POS	NEG	AMB	NEG	NEG	POS	POS	POS	AMB	AMB	POS	POS	NEG	NEG	POS
2012330_Z3617 (7605/12)	POS	POS	POS	NEG	AMB	NEG	NEG	POS	POS	AMB	POS	AMB	POS	POS	NEG	NEG	POS
2012332_Z3619 (8076/12)	POS	POS	POS	NEG	NEG	NEG	NEG	POS	POS	AMB	POS	AMB	POS	POS	NEG	NEG	POS
2012339_Z3626 (8558/12)	POS	POS	POS	NEG	NEG	NEG	NEG	POS	POS	AMB	POS	AMB	POS	POS	NEG	NEG	POS
2012340_Z3627 (8523/12)	POS	POS	POS	NEG	NEG	NEG	NEG	POS	POS	AMB	POS	AMB	POS	POS	NEG	NEG	POS
2012348_Z3635	POS	POS	POS	NEG	NEG	NEG	NEG	POS	POS	AMB	POS	AMB	POS	POS	NEG	NEG	POS
2012350_Z3637	POS	POS	POS	NEG	AMB	NEG	NEG	POS	POS	AMB	POS	AMB	POS	POS	NEG	NEG	POS
2012_370_Z3430 (1112-52177)	POS	POS	POS	NEG	AMB	NEG	NEG	POS	POS	AMB	POS	AMB	POS	POS	NEG	NEG	POS
2012_378_Z3438 (4139)	POS	POS	POS	NEG	NEG	NEG	NEG	POS	POS	AMB	POS	AMB	POS	POS	NEG	NEG	POS
2013_PA21_Z4004	POS	POS	POS	NEG	NEG	NEG	NEG	POS	POS	POS	AMB	AMB	POS	POS	NEG	NEG	NEG
2013_PA22_Z4005	POS	POS	POS	NEG	AMB	NEG	NEG	POS	POS	AMB	AMB	POS	POS	POS	NEG	NEG	POS
2013_PA24_Z4007	POS	POS	POS	NEG	NEG	NEG	NEG	POS	POS	POS	AMB	AMB	POS	POS	NEG	NEG	POS
2013_PA31_Z4014	POS	POS	POS	NEG	AMB	NEG	NEG	POS	POS	POS	AMB	AMB	POS	POS	NEG	NEG	POS
2013_PA35_Z4018	POS	POS	POS	NEG	NEG	NEG	NEG	POS	POS	POS	AMB	AMB	POS	POS	NEG	NEG	NEG
2013_PA81_Z4064	POS	POS	POS	NEG	AMB	NEG	NEG	POS	POS	POS	AMB	AMB	POS	POS	NEG	NEG	POS
**ST239-MRSA-III+ccrA/B4**																	
2012_254_Z3537 (3124A/12)	POS	POS	POS	NEG	NEG	NEG	NEG	POS	POS	POS	AMB	AMB	POS	POS	NEG	NEG	POS
2013_PA25_Z4008	POS	POS	POS	NEG	AMB	NEG	NEG	POS	POS	POS	AMB	AMB	POS	POS	NEG	NEG	POS
**ST834-MSSA**																	
2012_268_Z3555 (3693/12)	POS	POS	POS	NEG	NEG	NEG	AMB	POS	POS	POS	AMB	AMB	POS	POS	NEG	NEG	POS
**CC12-MSSA**																	
2012_210_Z3470 (1177/12)	NEG	NEG	NEG	NEG	NEG	NEG	NEG	POS	POS	POS	AMB	AMB	POS	POS	NEG	NEG	POS
2012_230_Z3487 (1797/12)	NEG	NEG	NEG	NEG	NEG	NEG	NEG	POS	POS	AMB	AMB	POS	POS	POS	NEG	NEG	POS
2013_PA18_Z4001	NEG	NEG	NEG	NEG	NEG	NEG	NEG	POS	POS	POS	AMB	AMB	POS	POS	NEG	NEG	POS
**CC15-MSSA**																	
2012_160_Z3444 (26386/11)	POS	POS	AMB	NEG	POS	NEG	NEG	POS	POS	NEG	NEG	POS	POS	POS	NEG	AMB	POS
2012_167_Z3514 (27010/11)	POS	POS	AMB	NEG	POS	NEG	NEG	POS	POS	NEG	NEG	POS	POS	POS	NEG	AMB	POS
2012_184_Z3455 (376/12)	POS	POS	POS	NEG	AMB	NEG	NEG	POS	POS	NEG	NEG	POS	POS	POS	NEG	AMB	POS
2012_205_Z3465 (939/12)	POS	POS	AMB	NEG	POS	NEG	NEG	POS	POS	NEG	NEG	POS	POS	POS	NEG	AMB	POS
2012_231_Z3519 (1831/12)	POS	POS	AMB	NEG	POS	NEG	NEG	POS	POS	NEG	NEG	POS	POS	POS	NEG	AMB	POS
2012_249_Z3533 (2907/12)	POS	POS	AMB	NEG	POS	NEG	NEG	POS	POS	NEG	NEG	POS	POS	POS	NEG	AMB	POS
2012_305_Z3592 (5839/12)	POS	POS	AMB	NEG	POS	NEG	NEG	POS	POS	NEG	NEG	POS	POS	POS	NEG	POS	AMB
2012_313_Z3600 (6857/12)	POS	POS	AMB	NEG	POS	NEG	NEG	POS	POS	NEG	NEG	POS	POS	POS	NEG	AMB	POS
2012_345_Z3632 (7966B/12)	POS	POS	AMB	NEG	POS	NEG	NEG	POS	POS	NEG	NEG	POS	POS	POS	NEG	AMB	POS
2013_PA33_Z4016	POS	POS	NEG	NEG	POS	NEG	NEG	POS	POS	NEG	NEG	POS	POS	POS	NEG	POS	AMB
**CC20-MSSA**																	
2012_240_Z3526 (2354/12)	POS	POS	NEG	NEG	POS	NEG	NEG	POS	POS	NEG	NEG	POS	POS	POS	NEG	NEG	POS
**CC30-MSSA**																	
2012_155_Z3488 (25905A/11)	POS	POS	AMB	POS	NEG	NEG	NEG	POS	POS	POS	AMB	NEG	POS	POS	NEG	NEG	POS
**CC30-MSSA [PVL+]**																	
2012_164_Z3447 (26574/11)	POS	POS	POS	AMB	NEG	NEG	NEG	POS	POS	AMB	POS	AMB	POS	POS	AMB	NEG	POS
2012_166_Z3493 (26988A/11)	POS	POS	POS	AMB	NEG	NEG	NEG	POS	POS	POS	AMB	NEG	POS	POS	NEG	NEG	POS
2012_178_Z3500 (197/12)	POS	POS	AMB	POS	NEG	NEG	NEG	POS	POS	POS	AMB	NEG	POS	POS	NEG	NEG	POS
2012_183_Z3454	POS	POS	POS	AMB	NEG	NEG	NEG	POS	POS	POS	AMB	NEG	POS	POS	NEG	NEG	POS
2012_186_Z3503 (444/12)	POS	POS	AMB	POS	NEG	NEG	NEG	POS	POS	POS	AMB	NEG	POS	POS	NEG	NEG	POS
2012_189_Z3506 (542/12)	POS	POS	AMB	POS	NEG	NEG	NEG	POS	POS	POS	AMB	NEG	POS	POS	NEG	NEG	POS
2012_192_Z3457 (606/12)	POS	POS	AMB	POS	NEG	NEG	NEG	POS	POS	AMB	POS	NEG	POS	POS	NEG	NEG	POS
2012_196_Z3509 (707/12)	POS	POS	AMB	POS	NEG	NEG	NEG	POS	POS	POS	AMB	AMB	POS	POS	NEG	NEG	POS
2012_218_Z3476 (1432/12)	POS	POS	AMB	POS	NEG	NEG	NEG	POS	POS	POS	AMB	NEG	POS	POS	NEG	NEG	POS
2012_232_Z3520 (1892/12)	POS	POS	POS	AMB	NEG	NEG	NEG	POS	POS	POS	AMB	AMB	POS	POS	AMB	NEG	POS
2012_278_Z3565 (4397/12)	POS	POS	AMB	POS	NEG	NEG	NEG	POS	POS	POS	AMB	AMB	POS	POS	NEG	NEG	POS
2012_285_Z3572 (4802/12)	POS	POS	AMB	POS	NEG	NEG	NEG	POS	POS	POS	AMB	NEG	POS	POS	NEG	NEG	POS
2012_287_Z3574 (4906/12)	POS	POS	AMB	POS	NEG	NEG	NEG	POS	POS	POS	AMB	NEG	POS	POS	NEG	NEG	POS
2012_295_Z3582 (5116/12)	POS	POS	AMB	POS	NEG	NEG	NEG	POS	POS	POS	AMB	NEG	POS	POS	NEG	NEG	POS
2012_326_Z3613 (7321/12)	POS	POS	AMB	POS	NEG	NEG	AMB	POS	POS	POS	AMB	NEG	POS	POS	NEG	NEG	POS
2012_356_Z3643 (1204-15897)	POS	POS	NEG	POS	NEG	NEG	NEG	POS	POS	POS	AMB	NEG	POS	POS	NEG	NEG	POS
2012_366_Z3426 (1204-16124)	POS	POS	POS	AMB	NEG	NEG	NEG	POS	POS	AMB	POS	AMB	POS	POS	AMB	NEG	POS
2012_372_Z3432 (1205-19821)	POS	POS	AMB	POS	NEG	NEG	NEG	POS	POS	POS	AMB	NEG	POS	POS	NEG	NEG	POS
2012_380_Z3440 (4141)	POS	POS	AMB	POS	NEG	NEG	NEG	POS	POS	AMB	POS	AMB	POS	POS	AMB	NEG	POS
2013_PA20_Z4003	POS	POS	NEG	POS	NEG	NEG	NEG	POS	POS	POS	AMB	NEG	POS	POS	NEG	NEG	POS
2013_PA36_Z4019	POS	POS	NEG	POS	NEG	NEG	NEG	POS	POS	AMB	POS	NEG	POS	POS	NEG	NEG	NEG
2013_PA46_Z4029	POS	POS	NEG	POS	NEG	NEG	NEG	POS	POS	AMB	POS	NEG	POS	POS	NEG	NEG	NEG
2013_PA50_Z4033	POS	POS	NEG	POS	NEG	NEG	NEG	POS	POS	AMB	POS	NEG	POS	POS	NEG	NEG	NEG
2013_PA63_Z4046	POS	POS	NEG	POS	NEG	NEG	NEG	POS	POS	POS	AMB	NEG	POS	POS	NEG	NEG	NEG
2013_PA78_Z4061	POS	POS	NEG	POS	NEG	NEG	NEG	POS	POS	POS	AMB	NEG	POS	POS	NEG	NEG	POS
2013_PA79_Z4062	POS	POS	NEG	POS	NEG	NEG	NEG	POS	POS	POS	AMB	NEG	POS	POS	NEG	NEG	POS
**CC45-MSSA**																	
2012_172_Z3449 (49/12)	POS	POS	POS	NEG	AMB	NEG	AMB	POS	POS	POS	NEG	AMB	POS	POS	AMB	POS	AMB
2012_208_Z3468 (1011/12)	POS	POS	AMB	NEG	NEG	NEG	POS	POS	POS	POS	NEG	AMB	POS	POS	NEG	AMB	POS
2012_235_Z3522 (2085/12)	POS	POS	AMB	NEG	AMB	NEG	POS	POS	POS	POS	NEG	AMB	POS	POS	NEG	POS	AMB
2012_290_Z3577 (4921/12)	POS	POS	NEG	NEG	NEG	NEG	POS	POS	POS	POS	NEG	AMB	POS	POS	NEG	POS	AMB
2012_311_Z3598 (6182/12)	NEG	NEG	NEG	NEG	NEG	NEG	NEG	POS	POS	AMB	NEG	POS	POS	POS	NEG	AMB	POS
Z3604_TT317 - {DC558C18-2959-4049-B8F3-507365	NEG	NEG	NEG	NEG	NEG	NEG	NEG	POS	POS	AMB	NEG	POS	POS	POS	NEG	POS	AMB
2012_363_Z3423 (1112-61058)	POS	POS	AMB	NEG	AMB	NEG	POS	POS	POS	POS	NEG	AMB	POS	POS	AMB	POS	AMB
2013_PA19_Z4002	POS	POS	NEG	NEG	AMB	NEG	POS	POS	POS	AMB	NEG	POS	POS	POS	NEG	POS	AMB
2013_PA54_Z4037	POS	POS	AMB	NEG	AMB	NEG	POS	POS	POS	POS	NEG	AMB	POS	POS	NEG	POS	AMB
2013_PA82_Z4065	NEG	NEG	NEG	NEG	NEG	NEG	NEG	POS	POS	AMB	NEG	POS	POS	POS	NEG	POS	AMB
**CC97-MSSA**																	
2012_182_Z3453 (279A/12)	POS	POS	AMB	NEG	POS	NEG	NEG	POS	POS	AMB	POS	AMB	POS	POS	NEG	NEG	POS
2012_187_Z3504	POS	POS	AMB	NEG	POS	NEG	NEG	POS	POS	AMB	AMB	POS	POS	POS	NEG	NEG	POS
2012_197_Z3510 (757/12)	POS	POS	AMB	NEG	POS	NEG	NEG	POS	POS	AMB	AMB	POS	POS	POS	NEG	NEG	POS
2012_199_Z3516 (785/12)	POS	POS	AMB	NEG	POS	NEG	NEG	POS	POS	AMB	POS	AMB	POS	POS	NEG	NEG	POS
2013_PA15_Z3998	POS	POS	NEG	NEG	POS	NEG	NEG	POS	POS	POS	AMB	AMB	POS	POS	NEG	NEG	POS
**CC101-MSSA**																	
2012_283_Z3570 (4725/12)	POS	POS	POS	AMB	NEG	NEG	NEG	POS	POS	AMB	AMB	POS	POS	POS	NEG	NEG	POS
2012_247_Z3531 (2788/12)	POS	POS	POS	AMB	NEG	NEG	NEG	POS	POS	AMB	AMB	POS	POS	POS	NEG	NEG	POS
2012_279_Z3566 (4506/12)	POS	POS	POS	AMB	NEG	NEG	NEG	POS	POS	AMB	POS	AMB	POS	POS	NEG	NEG	POS

Strain / Isolates	collagen-binding adhesin	cell wall associated fibronectin-binding protein	cell surface elastin binding protein					enolase	fibrinogen binding protein (19 kDa)		fibronectin-binding protein A					
	cna	ebh (consensus)	ebpS (total)	ebpS_probe 612	ebpS_probe 614	ebpS (01-1111)	ebpS (COL)	eno	fib	fib (MRSA252)	fnbA (total)	fnbA (consensus)	fnbA (COL)	fnbA (MRSA252)	fnbA (Mu50+MW2)	fnbA (RF122)
**ST8-MRSA-IV, USA300**																
2012_158_Z3491 (26049A/11)	NEG	POS	POS	POS	POS	NEG	POS	POS	POS	NEG	POS	POS	POS	NEG	NEG	NEG
2012_165_Z3513 (26656/11)	NEG	POS	POS	POS	POS	NEG	POS	POS	POS	NEG	POS	POS	POS	NEG	NEG	NEG
2012_214_Z3473 (1322/12)	NEG	POS	POS	POS	POS	NEG	POS	POS	POS	NEG	POS	POS	POS	NEG	NEG	NEG
2012_227_Z3484 (1688/12)	NEG	POS	POS	POS	POS	NEG	POS	POS	POS	NEG	POS	POS	POS	NEG	NEG	NEG
2012_263_Z3550 (3355/12)	NEG	POS	POS	POS	POS	NEG	POS	POS	POS	AMB	POS	POS	POS	NEG	NEG	NEG
2012_266_Z3553	NEG	POS	POS	POS	POS	NEG	POS	POS	POS	AMB	POS	POS	POS	NEG	NEG	NEG
2012_284_Z3571 (4729/12)	NEG	POS	POS	POS	POS	NEG	POS	POS	POS	NEG	POS	POS	POS	NEG	NEG	NEG
2012_298_Z3585 (5203/12)	NEG	POS	POS	POS	POS	NEG	POS	POS	POS	AMB	POS	POS	POS	NEG	NEG	NEG
2012_304_Z3591 (5818/12)	NEG	POS	POS	POS	POS	NEG	POS	POS	POS	NEG	POS	POS	POS	NEG	NEG	NEG
Z3595_TT308 - {EFC74B77-3925-484F-A4CF-A1F7DC	NEG	POS	POS	POS	POS	NEG	POS	POS	POS	NEG	POS	POS	POS	NEG	NEG	NEG
2012_369_Z3429 (1112-58547)	NEG	POS	POS	POS	POS	NEG	POS	POS	POS	NEG	POS	POS	POS	NEG	NEG	NEG
2012_374_Z3434 (1203-13527)	NEG	POS	POS	POS	POS	NEG	POS	POS	POS	AMB	POS	POS	POS	NEG	NEG	NEG
2013_PA06_Z3989	NEG	POS	POS	POS	POS	NEG	POS	POS	POS	NEG	POS	POS	POS	NEG	NEG	NEG
2013_PA55_Z4038	NEG	POS	POS	POS	POS	NEG	POS	POS	POS	NEG	POS	POS	POS	NEG	NEG	NEG
2013_PA71_Z4054	NEG	POS	POS	POS	POS	NEG	POS	POS	POS	NEG	POS	POS	POS	NEG	NEG	NEG
**ST8-MRSA-IV [PVL+/ACME+], US**																
2012_180_Z3501 (232/12)	NEG	POS	POS	POS	POS	NEG	POS	POS	POS	NEG	POS	POS	POS	NEG	NEG	NEG
2013_PA68_Z4051	NEG	POS	POS	POS	POS	NEG	POS	POS	POS	NEG	POS	POS	POS	NEG	NEG	NEG
**ST72-MSSA**																
2012_174_Z3451 (102/12)	NEG	POS	POS	POS	POS	NEG	NEG	POS	POS	NEG	POS	POS	POS	NEG	NEG	NEG
2012_223_Z3480 (1506A/12)	NEG	POS	POS	POS	POS	NEG	NEG	POS	POS	NEG	POS	POS	POS	NEG	NEG	NEG
2012_236_Z3523 (2086/12)	NEG	POS	POS	POS	POS	NEG	NEG	POS	POS	AMB	POS	POS	POS	NEG	NEG	NEG
2012_239_Z3525 (2346/12)	NEG	POS	POS	POS	POS	NEG	NEG	POS	POS	AMB	POS	POS	POS	NEG	NEG	NEG
2012_252_Z3535 (3030/12)	NEG	POS	POS	POS	POS	NEG	NEG	POS	POS	AMB	POS	POS	POS	NEG	NEG	NEG
2012_271_Z3558 (3799/12)	NEG	POS	POS	POS	POS	POS	NEG	POS	POS	AMB	POS	POS	POS	NEG	NEG	NEG
2012_276_Z3563	NEG	POS	POS	POS	POS	NEG	NEG	POS	POS	NEG	POS	POS	POS	NEG	NEG	NEG
2012_300_Z3587 (5638/12)	NEG	POS	POS	POS	POS	NEG	NEG	POS	POS	NEG	POS	POS	POS	NEG	NEG	NEG
2012_316_Z3603 (6941/12)	NEG	POS	POS	POS	POS	NEG	NEG	POS	POS	AMB	POS	POS	POS	NEG	NEG	NEG
2012_319_Z3606 (7019/12)	NEG	POS	POS	POS	POS	NEG	NEG	POS	POS	NEG	POS	POS	POS	NEG	NEG	NEG
2012_371_Z3431 (1204-17577)	NEG	POS	POS	POS	POS	NEG	NEG	POS	POS	NEG	POS	POS	POS	NEG	NEG	NEG
2013_PA05_Z3988	NEG	POS	POS	POS	POS	NEG	NEG	POS	POS	NEG	POS	POS	POS	NEG	NEG	NEG
2013_PA65_Z4048	NEG	POS	POS	POS	POS	NEG	NEG	POS	POS	NEG	POS	POS	POS	NEG	NEG	NEG
2013_PA73_Z4056	NEG	POS	POS	POS	POS	NEG	NEG	POS	POS	NEG	POS	POS	POS	NEG	NEG	NEG
**ST72-MSSA-SCCfus**																
2013_PA16_Z3999	NEG	POS	POS	POS	POS	NEG	NEG	POS	POS	NEG	POS	POS	POS	NEG	NEG	NEG
**ST72-MSSA-SCCfus/kdp**																
2012_361_Z3421 (1201-1299)	NEG	POS	POS	POS	POS	NEG	NEG	POS	POS	NEG	POS	POS	POS	NEG	NEG	NEG
**ST239-MRSA-III&SCCmer**																
2012_157_Z3490	POS	POS	POS	POS	POS	NEG	POS	POS	POS	NEG	POS	POS	POS	NEG	NEG	NEG
2012_168_Z3494 (27129/11)	POS	POS	POS	POS	POS	NEG	POS	POS	POS	NEG	POS	POS	POS	NEG	NEG	NEG
2012_195_Z3508 (619/12)	POS	POS	POS	POS	POS	NEG	POS	POS	POS	AMB	POS	POS	POS	NEG	NEG	NEG
2012_222_Z3479 (1489/12)	POS	POS	POS	POS	POS	NEG	POS	POS	POS	NEG	POS	POS	POS	NEG	NEG	NEG
2012_233_Z3540 (1943/12)	POS	POS	POS	POS	POS	NEG	POS	POS	POS	NEG	POS	POS	POS	NEG	NEG	NEG
2012_237_Z3541	POS	POS	POS	POS	POS	NEG	POS	POS	POS	NEG	POS	POS	POS	NEG	NEG	NEG
2012_262_Z3549	POS	POS	POS	POS	POS	NEG	POS	POS	POS	AMB	POS	POS	POS	NEG	NEG	NEG
2012_275_Z3562 (3893/12)	POS	POS	POS	POS	POS	NEG	POS	POS	POS	NEG	POS	POS	POS	NEG	NEG	NEG
2012_291_Z3578	POS	POS	POS	POS	POS	NEG	POS	POS	POS	NEG	POS	POS	POS	NEG	NEG	NEG
2012_314_Z3601 (6939/12)	POS	POS	POS	POS	POS	NEG	POS	POS	POS	AMB	POS	POS	POS	NEG	NEG	NEG
2012323_Z3610	POS	POS	POS	POS	POS	NEG	POS	POS	POS	NEG	POS	POS	POS	NEG	NEG	NEG
2012330_Z3617 (7605/12)	POS	POS	POS	POS	POS	NEG	POS	POS	POS	AMB	POS	POS	POS	NEG	NEG	NEG
2012332_Z3619 (8076/12)	POS	POS	POS	POS	POS	NEG	POS	POS	POS	AMB	POS	POS	POS	NEG	NEG	NEG
2012339_Z3626 (8558/12)	POS	POS	POS	POS	POS	NEG	POS	POS	POS	NEG	POS	POS	POS	NEG	NEG	NEG
2012340_Z3627 (8523/12)	POS	POS	POS	POS	POS	NEG	POS	POS	POS	NEG	POS	POS	POS	NEG	NEG	NEG
2012348_Z3635	POS	POS	POS	POS	POS	NEG	POS	POS	POS	AMB	POS	POS	POS	NEG	NEG	NEG
2012350_Z3637	POS	POS	POS	POS	POS	NEG	POS	POS	POS	AMB	POS	POS	POS	NEG	NEG	NEG
2012_370_Z3430 (1112-52177)	POS	POS	POS	POS	POS	NEG	POS	POS	POS	NEG	POS	POS	POS	NEG	NEG	NEG
2012_378_Z3438 (4139)	POS	POS	POS	POS	POS	NEG	POS	POS	POS	AMB	POS	POS	POS	NEG	NEG	NEG
2013_PA21_Z4004	POS	POS	POS	POS	POS	NEG	POS	POS	POS	NEG	POS	POS	POS	NEG	NEG	NEG
2013_PA22_Z4005	POS	POS	POS	POS	POS	NEG	POS	POS	POS	NEG	POS	POS	POS	NEG	NEG	NEG
2013_PA24_Z4007	POS	POS	POS	POS	POS	NEG	POS	POS	POS	NEG	POS	POS	POS	NEG	NEG	NEG
2013_PA31_Z4014	POS	POS	POS	POS	POS	NEG	POS	POS	POS	NEG	POS	POS	POS	NEG	NEG	NEG
2013_PA35_Z4018	POS	POS	POS	POS	POS	NEG	POS	POS	POS	NEG	POS	POS	POS	NEG	NEG	NEG
2013_PA81_Z4064	POS	POS	POS	POS	POS	NEG	POS	POS	POS	NEG	POS	POS	POS	NEG	NEG	NEG
**ST239-MRSA-III+ccrA/B4**																
2012_254_Z3537 (3124A/12)	POS	POS	POS	POS	POS	NEG	POS	POS	POS	NEG	POS	POS	POS	NEG	NEG	NEG
2013_PA25_Z4008	POS	POS	POS	POS	POS	NEG	POS	POS	POS	NEG	POS	POS	POS	NEG	NEG	NEG
**ST834-MSSA**																
2012_268_Z3555 (3693/12)	NEG	POS	POS	POS	POS	NEG	NEG	POS	POS	NEG	POS	POS	NEG	POS	NEG	NEG
**CC12-MSSA**																
2012_210_Z3470 (1177/12)	POS	POS	POS	POS	POS	NEG	NEG	POS	POS	NEG	POS	POS	NEG	NEG	NEG	NEG
2012_230_Z3487 (1797/12)	POS	POS	POS	POS	POS	NEG	NEG	POS	POS	NEG	POS	POS	NEG	NEG	NEG	NEG
2013_PA18_Z4001	POS	POS	POS	POS	POS	NEG	NEG	POS	POS	NEG	POS	POS	NEG	NEG	NEG	NEG
**CC15-MSSA**																
2012_160_Z3444 (26386/11)	NEG	POS	POS	POS	POS	NEG	POS	POS	POS	NEG	POS	POS	POS	NEG	NEG	NEG
2012_167_Z3514 (27010/11)	NEG	POS	POS	POS	POS	NEG	POS	POS	POS	AMB	POS	POS	POS	NEG	NEG	NEG
2012_184_Z3455 (376/12)	NEG	POS	POS	POS	POS	NEG	POS	POS	POS	AMB	POS	POS	POS	NEG	NEG	NEG
2012_205_Z3465 (939/12)	NEG	POS	POS	POS	POS	NEG	POS	POS	POS	AMB	POS	POS	POS	NEG	NEG	NEG
2012_231_Z3519 (1831/12)	NEG	POS	POS	POS	POS	NEG	POS	POS	POS	NEG	POS	POS	POS	NEG	NEG	NEG
2012_249_Z3533 (2907/12)	NEG	POS	POS	POS	POS	NEG	POS	POS	POS	AMB	POS	POS	POS	NEG	NEG	NEG
2012_305_Z3592 (5839/12)	NEG	POS	POS	POS	POS	NEG	POS	POS	POS	NEG	POS	POS	POS	NEG	NEG	NEG
2012_313_Z3600 (6857/12)	NEG	POS	POS	POS	POS	NEG	POS	POS	POS	NEG	POS	POS	POS	NEG	NEG	NEG
2012_345_Z3632 (7966B/12)	NEG	POS	POS	POS	POS	NEG	POS	POS	POS	AMB	POS	POS	POS	NEG	NEG	NEG
2013_PA33_Z4016	NEG	POS	POS	POS	POS	NEG	POS	POS	POS	NEG	POS	POS	POS	NEG	NEG	NEG
**CC20-MSSA**																
2012_240_Z3526 (2354/12)	NEG	POS	POS	POS	POS	NEG	POS	POS	POS	AMB	POS	POS	NEG	POS	NEG	NEG
**CC30-MSSA**																
2012_155_Z3488 (25905A/11)	POS	POS	POS	POS	POS	NEG	NEG	POS	NEG	POS	POS	POS	NEG	POS	NEG	NEG
**CC30-MSSA [PVL+]**																
2012_164_Z3447 (26574/11)	POS	POS	POS	POS	POS	NEG	NEG	POS	NEG	POS	POS	POS	NEG	POS	NEG	AMB
2012_166_Z3493 (26988A/11)	POS	POS	POS	POS	POS	NEG	NEG	POS	NEG	POS	POS	POS	NEG	POS	NEG	NEG
2012_178_Z3500 (197/12)	POS	POS	POS	POS	POS	NEG	NEG	POS	NEG	POS	POS	POS	NEG	POS	NEG	NEG
2012_183_Z3454	POS	POS	POS	POS	POS	NEG	NEG	POS	NEG	POS	POS	POS	NEG	POS	NEG	NEG
2012_186_Z3503 (444/12)	POS	POS	POS	POS	POS	NEG	NEG	POS	NEG	POS	POS	POS	NEG	POS	NEG	NEG
2012_189_Z3506 (542/12)	POS	POS	POS	POS	POS	NEG	NEG	POS	NEG	POS	POS	POS	NEG	POS	NEG	NEG
2012_192_Z3457 (606/12)	POS	POS	POS	POS	POS	NEG	NEG	POS	NEG	POS	POS	POS	NEG	POS	NEG	NEG
2012_196_Z3509 (707/12)	POS	POS	POS	POS	POS	NEG	NEG	POS	NEG	POS	POS	POS	NEG	POS	NEG	NEG
2012_218_Z3476 (1432/12)	POS	POS	POS	POS	POS	NEG	NEG	POS	NEG	POS	POS	POS	NEG	POS	NEG	NEG
2012_232_Z3520 (1892/12)	POS	POS	POS	POS	POS	NEG	NEG	POS	NEG	POS	POS	POS	NEG	POS	NEG	NEG
2012_278_Z3565 (4397/12)	POS	POS	POS	POS	POS	NEG	NEG	POS	NEG	POS	POS	POS	NEG	POS	NEG	NEG
2012_285_Z3572 (4802/12)	POS	POS	POS	POS	POS	NEG	NEG	POS	NEG	POS	POS	POS	NEG	POS	NEG	NEG
2012_287_Z3574 (4906/12)	POS	POS	POS	POS	POS	NEG	NEG	POS	NEG	POS	POS	POS	NEG	POS	NEG	NEG
2012_295_Z3582 (5116/12)	POS	POS	POS	POS	POS	NEG	NEG	POS	NEG	POS	POS	POS	NEG	POS	NEG	NEG
2012_326_Z3613 (7321/12)	POS	POS	POS	POS	POS	NEG	NEG	POS	NEG	POS	POS	POS	NEG	POS	NEG	NEG
2012_356_Z3643 (1204-15897)	POS	POS	POS	POS	POS	NEG	NEG	POS	NEG	POS	POS	POS	NEG	POS	NEG	NEG
2012_366_Z3426 (1204-16124)	POS	POS	POS	POS	POS	NEG	NEG	POS	NEG	POS	POS	POS	NEG	POS	NEG	NEG
2012_372_Z3432 (1205-19821)	POS	POS	POS	POS	POS	NEG	NEG	POS	NEG	POS	POS	POS	NEG	POS	NEG	NEG
2012_380_Z3440 (4141)	POS	POS	POS	POS	POS	NEG	NEG	POS	NEG	POS	POS	POS	NEG	POS	NEG	NEG
2013_PA20_Z4003	POS	POS	POS	POS	POS	NEG	NEG	POS	NEG	POS	POS	POS	NEG	POS	NEG	NEG
2013_PA36_Z4019	POS	POS	POS	POS	AMB	POS	NEG	NEG	POS	NEG	POS	POS	POS	NEG	POS	NEG	NEG
2013_PA46_Z4029	POS	POS	POS	POS	POS	NEG	NEG	POS	NEG	POS	POS	POS	NEG	POS	NEG	NEG
2013_PA50_Z4033	POS	POS	POS	POS	POS	NEG	NEG	POS	NEG	POS	POS	POS	NEG	POS	NEG	NEG
2013_PA63_Z4046	POS	POS	POS	POS	POS	NEG	NEG	POS	NEG	POS	POS	POS	NEG	POS	NEG	NEG
2013_PA78_Z4061	POS	POS	POS	POS	POS	NEG	NEG	POS	NEG	POS	POS	POS	NEG	POS	NEG	NEG
2013_PA79_Z4062	POS	POS	POS	POS	POS	NEG	NEG	POS	NEG	POS	POS	POS	NEG	POS	NEG	NEG
**CC45-MSSA**																
2012_172_Z3449 (49/12)	POS	POS	POS	NEG	POS	POS	NEG	POS	NEG	POS	POS	POS	NEG	NEG	POS	NEG
2012_208_Z3468 (1011/12)	POS	POS	POS	NEG	POS	POS	NEG	POS	NEG	POS	POS	POS	NEG	NEG	POS	NEG
2012_235_Z3522 (2085/12)	POS	POS	POS	NEG	POS	POS	NEG	POS	NEG	POS	POS	POS	NEG	NEG	POS	NEG
2012_290_Z3577 (4921/12)	POS	POS	POS	NEG	POS	POS	NEG	POS	NEG	POS	POS	POS	NEG	NEG	POS	NEG
2012_311_Z3598 (6182/12)	POS	POS	POS	NEG	POS	POS	NEG	POS	NEG	POS	NEG	NEG	NEG	NEG	NEG	NEG
Z3604_TT317 - {DC558C18-2959-4049-B8F3-507365	POS	POS	POS	NEG	POS	POS	NEG	POS	NEG	POS	POS	POS	NEG	NEG	POS	NEG
2012_363_Z3423 (1112-61058)	POS	POS	POS	NEG	POS	POS	NEG	POS	NEG	POS	NEG	NEG	NEG	NEG	NEG	NEG
2013_PA19_Z4002	POS	POS	POS	NEG	POS	POS	NEG	POS	NEG	POS	NEG	NEG	NEG	NEG	NEG	NEG
2013_PA54_Z4037	POS	POS	POS	NEG	POS	POS	POS	POS	NEG	POS	POS	POS	NEG	NEG	POS	NEG
2013_PA82_Z4065	POS	POS	POS	NEG	POS	POS	NEG	POS	NEG	POS	POS	POS	NEG	NEG	POS	NEG
**CC97-MSSA**																
2012_182_Z3453 (279A/12)	NEG	POS	POS	POS	POS	NEG	NEG	POS	POS	AMB	POS	POS	NEG	NEG	NEG	NEG
2012_187_Z3504	NEG	POS	POS	POS	POS	NEG	NEG	POS	POS	NEG	POS	POS	NEG	NEG	NEG	NEG
2012_197_Z3510 (757/12)	NEG	POS	POS	POS	POS	NEG	NEG	POS	POS	AMB	POS	POS	NEG	NEG	NEG	NEG
2012_199_Z3516 (785/12)	NEG	POS	POS	POS	POS	NEG	NEG	POS	POS	AMB	POS	POS	NEG	NEG	NEG	NEG
2013_PA15_Z3998	NEG	POS	POS	POS	POS	NEG	NEG	POS	POS	NEG	POS	POS	NEG	NEG	NEG	NEG
**CC101-MSSA**																
2012_283_Z3570 (4725/12)	NEG	POS	POS	POS	POS	NEG	NEG	POS	POS	NEG	POS	POS	NEG	POS	NEG	NEG
2012_247_Z3531 (2788/12)	NEG	POS	POS	POS	POS	NEG	NEG	POS	POS	NEG	POS	POS	NEG	POS	NEG	NEG
2012_279_Z3566 (4506/12)	NEG	POS	POS	POS	POS	NEG	NEG	POS	POS	NEG	POS	POS	NEG	POS	NEG	NEG

Strain / Isolates	bone sialoprotein-binding protein							clumping factor A					clumping factor B					collagen-binding adhesin	cell wall associated fibronectin-binding protein	cell surface elastin binding protein					enolase	fibrinogen binding protein (19 kDa)		fibronectin-binding protein A					
	bbp (total)	bbp (consensus)	bbp (COL+MW2)	bbp (MRSA252)	bbp (Mu50)	bbp (RF122)	bbp (ST45)	clfA (total)	clfA (consensus)	clfA (COL+RF122)	clfA (MRSA252)	clfA (Mu50+MW2)	clfB (total)	clfB (consensus)	clfB (COL+Mu50)	clfB (MW2)	clfB (RF122)	cna	ebh (consensus)	ebpS (total)	ebpS_probe 612	ebpS_probe 614	ebpS (01-1111)	ebpS (COL)	eno	fib	fib (MRSA252)	fnbA (total)	fnbA (consensus)	fnbA (COL)	fnbA (MRSA252)	fnbA (Mu50+MW2)	fnbA (RF122)
**CC121-MSSA**																																	
2012_163_Z3446 (26495/11)	POS	POS	POS	AMB	NEG	NEG	NEG	POS	POS	NEG	NEG	POS	POS	POS	NEG	NEG	POS	POS	POS	POS	NEG	POS	POS	AMB	POS	POS	NEG	POS	POS	NEG	NEG	NEG	NEG
2012_204_Z3464 (938/12)	POS	POS	AMB	POS	NEG	NEG	NEG	POS	POS	NEG	NEG	POS	POS	POS	AMB	NEG	POS	POS	POS	POS	NEG	POS	POS	NEG	POS	POS	AMB	POS	POS	NEG	NEG	NEG	NEG
2012_225_Z3482 (1563/12)	POS	POS	AMB	POS	NEG	NEG	NEG	POS	POS	NEG	NEG	POS	POS	POS	NEG	NEG	POS	POS	POS	POS	NEG	POS	POS	POS	POS	POS	NEG	POS	POS	NEG	NEG	NEG	NEG
2012_228_Z3485 (1700B/12)	POS	POS	AMB	POS	NEG	NEG	NEG	POS	POS	NEG	NEG	POS	POS	POS	NEG	NEG	POS	POS	POS	POS	NEG	POS	POS	AMB	POS	POS	NEG	POS	POS	NEG	NEG	NEG	NEG
2012_229_Z3486 (1737/12)	POS	POS	AMB	POS	NEG	NEG	NEG	POS	POS	NEG	NEG	POS	POS	POS	NEG	NEG	POS	POS	POS	POS	NEG	POS	POS	AMB	POS	POS	NEG	POS	POS	NEG	NEG	NEG	NEG
2012_302_Z3589 (5795/12)	POS	POS	NEG	POS	NEG	NEG	NEG	POS	POS	NEG	NEG	POS	POS	POS	NEG	NEG	POS	POS	POS	POS	NEG	POS	POS	NEG	POS	POS	NEG	POS	POS	NEG	NEG	NEG	NEG
2012_359_Z3419	POS	POS	NEG	POS	NEG	NEG	NEG	POS	POS	NEG	NEG	POS	POS	POS	NEG	NEG	POS	POS	POS	POS	NEG	POS	POS	NEG	POS	POS	NEG	POS	POS	NEG	NEG	NEG	NEG
2012_362_Z3422 (1201-996)	POS	POS	POS	AMB	NEG	NEG	NEG	POS	POS	NEG	NEG	POS	POS	POS	NEG	NEG	POS	POS	POS	POS	NEG	POS	POS	AMB	POS	POS	AMB	POS	POS	NEG	NEG	NEG	NEG
2012_367_Z3427 (1204-16413)	POS	POS	POS	AMB	NEG	NEG	NEG	POS	POS	NEG	NEG	POS	POS	POS	NEG	NEG	POS	POS	POS	POS	NEG	POS	POS	POS	POS	POS	NEG	POS	POS	NEG	NEG	NEG	NEG
2013_PA09_Z3992	POS	POS	AMB	POS	NEG	NEG	NEG	POS	POS	NEG	NEG	POS	POS	POS	NEG	NEG	POS	POS	POS	POS	NEG	POS	POS	AMB	POS	POS	NEG	POS	POS	NEG	NEG	NEG	NEG
2013_PA59_Z4042	POS	POS	NEG	POS	NEG	NEG	NEG	POS	POS	NEG	NEG	POS	POS	POS	NEG	NEG	POS	POS	POS	POS	NEG	POS	POS	NEG	POS	POS	NEG	POS	POS	NEG	NEG	NEG	NEG
2013_PA60_Z4043	POS	POS	NEG	POS	NEG	NEG	NEG	POS	POS	NEG	NEG	POS	POS	POS	NEG	NEG	NEG	POS	POS	POS	NEG	POS	POS	NEG	POS	POS	NEG	POS	POS	NEG	NEG	NEG	NEG
**CC152-MSSA [PVL+]**																																	
2012_190_Z3507 (549/12)	POS	POS	POS	NEG	AMB	NEG	NEG	POS	POS	AMB	POS	AMB	POS	POS	NEG	POS	AMB	POS	POS	POS	POS	POS	NEG	NEG	POS	NEG	NEG	POS	POS	NEG	NEG	NEG	NEG
2012_201_Z3461 (823/12)	POS	POS	POS	NEG	AMB	NEG	AMB	POS	POS	POS	AMB	AMB	POS	POS	NEG	POS	AMB	POS	POS	POS	POS	POS	NEG	NEG	POS	NEG	NEG	POS	POS	NEG	NEG	NEG	NEG
2012_209_Z3469 (1116/12)	POS	POS	POS	NEG	AMB	NEG	NEG	POS	POS	POS	AMB	AMB	POS	POS	NEG	AMB	POS	POS	POS	POS	POS	POS	NEG	NEG	POS	NEG	NEG	POS	POS	NEG	NEG	NEG	NEG
2012_211_Z3517 (1238/12)	POS	POS	POS	NEG	NEG	NEG	NEG	POS	POS	AMB	AMB	POS	POS	POS	NEG	POS	AMB	POS	POS	POS	POS	POS	NEG	NEG	POS	NEG	NEG	POS	POS	NEG	NEG	NEG	NEG
2012_213_Z3472 (1320/12)	POS	POS	POS	NEG	NEG	NEG	NEG	POS	POS	POS	AMB	AMB	POS	POS	NEG	POS	AMB	POS	POS	POS	POS	POS	NEG	NEG	POS	NEG	NEG	POS	POS	NEG	NEG	NEG	NEG
2012_221_Z3478	POS	POS	POS	NEG	AMB	NEG	NEG	POS	POS	POS	AMB	AMB	POS	POS	NEG	POS	AMB	POS	POS	POS	POS	POS	NEG	NEG	POS	NEG	NEG	POS	POS	NEG	NEG	NEG	NEG
2012_234_Z3521 (1981/12)	POS	POS	POS	NEG	NEG	NEG	NEG	POS	POS	AMB	POS	AMB	POS	POS	NEG	POS	AMB	POS	POS	POS	POS	POS	NEG	NEG	POS	NEG	NEG	POS	POS	NEG	NEG	NEG	NEG
2012_238_Z3524 (2199/12)	POS	POS	POS	NEG	NEG	NEG	NEG	POS	POS	AMB	POS	AMB	POS	POS	NEG	POS	AMB	POS	POS	POS	POS	POS	NEG	NEG	POS	NEG	NEG	POS	POS	NEG	NEG	NEG	NEG
2012_260_Z3547 (3290/12)	POS	POS	POS	NEG	AMB	NEG	NEG	POS	POS	POS	AMB	AMB	POS	POS	NEG	POS	AMB	POS	POS	POS	POS	POS	NEG	NEG	POS	NEG	NEG	POS	POS	NEG	NEG	NEG	NEG
2012_272_Z3559 (3802/12)	POS	POS	POS	NEG	NEG	NEG	NEG	POS	POS	AMB	POS	AMB	POS	POS	NEG	POS	AMB	POS	POS	POS	POS	POS	NEG	NEG	POS	NEG	NEG	POS	POS	NEG	NEG	NEG	NEG
2012_289_Z3576 (4912/12)	POS	POS	POS	NEG	NEG	NEG	NEG	POS	POS	AMB	POS	AMB	POS	POS	NEG	POS	AMB	POS	POS	POS	POS	POS	NEG	NEG	POS	NEG	NEG	POS	POS	NEG	NEG	NEG	NEG
2012_309_Z3596	POS	POS	POS	NEG	AMB	NEG	NEG	POS	POS	AMB	POS	AMB	POS	POS	NEG	POS	AMB	POS	POS	POS	POS	POS	NEG	NEG	POS	NEG	NEG	POS	POS	NEG	NEG	NEG	NEG
2012_322_Z3609 (7178/12)	POS	POS	POS	NEG	NEG	NEG	NEG	POS	POS	POS	AMB	AMB	POS	POS	NEG	POS	AMB	POS	POS	POS	POS	POS	NEG	NEG	POS	NEG	NEG	POS	POS	NEG	NEG	NEG	NEG
2012_325_Z3612 (7254/12)	POS	POS	POS	NEG	AMB	NEG	AMB	POS	POS	AMB	POS	AMB	POS	POS	NEG	POS	AMB	POS	POS	POS	POS	POS	NEG	NEG	POS	NEG	NEG	POS	POS	NEG	NEG	NEG	NEG
2012_328_Z3615 (7456/12)	POS	POS	POS	NEG	AMB	NEG	NEG	POS	POS	AMB	POS	AMB	POS	POS	NEG	POS	AMB	POS	POS	POS	POS	POS	NEG	NEG	POS	NEG	NEG	POS	POS	NEG	NEG	NEG	NEG
2012_334_Z3621 (8267/12)	POS	POS	POS	NEG	AMB	NEG	NEG	POS	POS	AMB	POS	AMB	POS	POS	NEG	POS	AMB	POS	POS	POS	POS	POS	NEG	NEG	POS	NEG	NEG	POS	POS	NEG	NEG	NEG	NEG
2012_360_Z3420 (1201-4047)	POS	POS	POS	NEG	NEG	NEG	NEG	POS	POS	AMB	POS	AMB	POS	POS	AMB	POS	AMB	POS	POS	POS	POS	POS	NEG	NEG	POS	NEG	NEG	POS	POS	NEG	NEG	NEG	NEG
2012_382_Z3442 (4086)	POS	POS	POS	NEG	NEG	NEG	NEG	POS	POS	AMB	AMB	POS	POS	POS	NEG	POS	AMB	POS	POS	POS	POS	POS	NEG	NEG	POS	NEG	NEG	POS	POS	NEG	NEG	NEG	NEG
2013_PA02_Z3985	POS	POS	POS	NEG	NEG	NEG	NEG	POS	POS	POS	AMB	AMB	POS	POS	NEG	POS	AMB	POS	POS	POS	POS	POS	NEG	NEG	POS	NEG	NEG	POS	POS	NEG	NEG	NEG	NEG
2013_PA03_Z3986	POS	POS	POS	NEG	NEG	NEG	NEG	POS	POS	POS	AMB	AMB	POS	POS	NEG	POS	AMB	POS	POS	POS	POS	POS	NEG	NEG	POS	NEG	NEG	POS	POS	NEG	NEG	NEG	NEG
2013_PA07_Z3990	POS	POS	POS	NEG	NEG	NEG	NEG	POS	POS	POS	AMB	AMB	POS	POS	NEG	AMB	POS	POS	POS	POS	POS	POS	NEG	NEG	POS	NEG	NEG	POS	POS	NEG	NEG	NEG	NEG
2013_PA34_Z4017	POS	POS	POS	NEG	NEG	NEG	NEG	POS	POS	POS	AMB	AMB	POS	POS	NEG	POS	AMB	POS	POS	POS	POS	POS	NEG	NEG	POS	NEG	NEG	POS	POS	NEG	NEG	NEG	NEG
2013_PA40_Z4023	POS	POS	POS	NEG	NEG	NEG	NEG	POS	POS	POS	AMB	AMB	POS	POS	NEG	POS	AMB	POS	POS	POS	POS	POS	NEG	NEG	POS	NEG	NEG	POS	POS	NEG	NEG	NEG	NEG
2013_PA48_Z4031	POS	POS	POS	NEG	NEG	NEG	NEG	POS	POS	POS	AMB	AMB	POS	POS	NEG	POS	AMB	POS	POS	POS	POS	POS	NEG	NEG	POS	NEG	NEG	POS	POS	NEG	NEG	NEG	NEG
2013_PA49_Z4032	POS	POS	POS	NEG	NEG	NEG	NEG	POS	POS	POS	AMB	AMB	POS	POS	NEG	POS	AMB	POS	POS	POS	POS	POS	NEG	NEG	POS	NEG	NEG	POS	POS	NEG	NEG	NEG	NEG
2013_PA51_Z4034	POS	POS	POS	NEG	NEG	NEG	NEG	POS	POS	AMB	AMB	POS	POS	POS	NEG	POS	AMB	POS	POS	POS	POS	POS	NEG	NEG	POS	NEG	NEG	POS	POS	NEG	NEG	NEG	NEG
2013_PA70_Z4053	POS	POS	POS	NEG	NEG	NEG	NEG	POS	POS	POS	AMB	AMB	POS	POS	NEG	POS	AMB	POS	POS	POS	POS	POS	NEG	NEG	POS	NEG	NEG	POS	POS	NEG	NEG	NEG	NEG
2013_PA84_Z4067	POS	POS	POS	NEG	NEG	NEG	NEG	POS	POS	POS	AMB	AMB	POS	POS	NEG	POS	AMB	POS	NEG	POS	POS	POS	NEG	NEG	POS	NEG	NEG	POS	POS	NEG	NEG	NEG	NEG
**CC188-MSSA**																																	
2012_206_Z3466 (968/12)	POS	POS	POS	NEG	AMB	NEG	NEG	POS	POS	NEG	NEG	POS	POS	POS	NEG	NEG	POS	POS	POS	POS	POS	POS	NEG	POS	POS	POS	POS	POS	POS	NEG	NEG	NEG	NEG
2012_251_Z3534 (3026/12)	POS	POS	POS	NEG	NEG	NEG	NEG	POS	POS	NEG	NEG	POS	POS	POS	NEG	NEG	POS	POS	POS	POS	POS	POS	NEG	POS	POS	POS	AMB	POS	POS	NEG	NEG	NEG	NEG
2012_320_Z3607 (7144/12)	POS	POS	POS	NEG	AMB	NEG	NEG	POS	POS	NEG	NEG	POS	POS	POS	NEG	NEG	POS	POS	POS	POS	POS	POS	NEG	POS	POS	POS	NEG	POS	POS	NEG	NEG	NEG	NEG
2012_321_Z3608 (7147/12)	POS	POS	POS	NEG	AMB	NEG	NEG	POS	POS	NEG	NEG	POS	POS	POS	NEG	NEG	POS	POS	POS	POS	POS	POS	NEG	POS	POS	POS	NEG	POS	POS	NEG	NEG	NEG	NEG
2012_358_Z3418 (1202-6332)	POS	POS	POS	NEG	AMB	NEG	NEG	POS	POS	NEG	NEG	POS	POS	POS	NEG	NEG	POS	POS	POS	POS	POS	POS	NEG	POS	POS	POS	AMB	POS	POS	NEG	NEG	NEG	NEG
**CC398-MSSA**																																	
2013_PA11_Z3994	POS	POS	AMB	NEG	AMB	NEG	POS	POS	POS	POS	AMB	AMB	POS	POS	NEG	POS	AMB	POS	POS	POS	NEG	POS	POS	POS	POS	NEG	POS	POS	POS	NEG	NEG	NEG	NEG
2013_PA57_Z4040	POS	POS	AMB	NEG	AMB	NEG	POS	POS	POS	POS	AMB	AMB	POS	POS	NEG	POS	AMB	POS	POS	POS	NEG	POS	POS	POS	POS	NEG	POS	POS	POS	NEG	NEG	NEG	NEG
**ST1852-MSSA**																																	
2012_277_Z3564 (4050/12)	POS	POS	POS	NEG	AMB	NEG	NEG	POS	POS	NEG	NEG	POS	POS	POS	NEG	NEG	POS	POS	POS	POS	POS	POS	NEG	POS	POS	POS	POS	POS	POS	NEG	NEG	NEG	NEG
**CC1223/1594-MSSA**																																	
2012_337_Z3624 (8070B/12)	POS	POS	NEG	NEG	NEG	NEG	NEG	POS	NEG	NEG	POS	NEG	POS	POS	POS	NEG	NEG	NEG	NEG	NEG	NEG	NEG	NEG	NEG	POS	NEG	NEG	POS	POS	NEG	NEG	NEG	NEG
**MSSA related to ST2250/2277**																																	
2012_203_Z3463 (829/12)	POS	POS	NEG	NEG	POS	NEG	AMB	POS	NEG	NEG	POS	NEG	POS	POS	POS	NEG	NEG	NEG	NEG	NEG	NEG	NEG	NEG	NEG	POS	POS	POS	POS	POS	POS	NEG	NEG	NEG
2012_269_Z3556 (3769A/12)	POS	POS	NEG	NEG	NEG	NEG	POS	POS	NEG	NEG	POS	NEG	POS	AMB	POS	NEG	NEG	NEG	NEG	NEG	NEG	NEG	NEG	NEG	POS	NEG	NEG	POS	POS	POS	NEG	NEG	NEG
2012_292_Z3579	POS	POS	NEG	NEG	NEG	NEG	NEG	POS	NEG	NEG	POS	NEG	POS	AMB	POS	NEG	NEG	NEG	NEG	NEG	NEG	NEG	NEG	NEG	POS	NEG	NEG	POS	POS	POS	NEG	NEG	NEG
2012_299_Z3586 (5487/12)	NEG	NEG	NEG	NEG	NEG	NEG	NEG	POS	NEG	NEG	POS	NEG	POS	AMB	POS	NEG	NEG	NEG	NEG	NEG	NEG	NEG	NEG	NEG	POS	NEG	NEG	POS	POS	POS	NEG	NEG	NEG
2012_301_Z3588 (5717/12)	POS	POS	NEG	NEG	NEG	NEG	NEG	POS	NEG	NEG	POS	NEG	POS	AMB	POS	NEG	NEG	NEG	NEG	NEG	NEG	NEG	NEG	NEG	POS	NEG	NEG	POS	POS	POS	NEG	NEG	NEG
**ST2596dlv-MSSA**																																	
2012_310_Z3597 (6034/12)	POS	POS	NEG	NEG	NEG	NEG	NEG	POS	NEG	NEG	POS	NEG	POS	POS	POS	NEG	NEG	POS	NEG	NEG	NEG	NEG	NEG	NEG	POS	NEG	NEG	POS	POS	NEG	POS	NEG	NEG

Strain / Isolates	fibronectin-binding protein B							Major histocompatibility complex class II analog protein (=Extracellular adherence protein, eap)				Staphylococcus aureus surface protein G				Ser-Asp rich fibrinogen-/bone sialoprotein-binding protein C							Ser-Asp rich fibrinogen-/bone sialoprotein-binding protein D					van Willebrand factor binding protein					
	fnbB (total)	fnbB (COL)	fnbB (COL+Mu50+MW2)	fnbB (Mu50)	fnbB (MW2)	fnbB (ST15)	fnbB (ST45-2)	map (total)	map (COL)	map (MRSA252)	map (Mu50+MW2)	sasG (total)	sasG (COL+Mu50)	sasG (MW2)	sasG (other than MRSA252+RF122)	sdrC (total)	sdrC (consensus)	sdrC (B1)	sdrC (COL)	sdrC (Mu50)	sdrC (MW2+MRSA252+RF122)	sdrC (other than MRSA252+RF122)	sdrD (total)	sdrD (consensus)	sdrD (COL+MW2)	sdrD (Mu50)	sdrD (other)	vwb (total)	vwb (consensus)	vwb (COL+MW2)	vwb (MRSA252)	vwb (Mu50)	vwb (RF122)
**CC1-MSSA**																																	
2012_257_Z3544 (3216/12)	POS	NEG	POS	NEG	AMB	NEG	NEG	POS	POS	NEG	POS	POS	NEG	POS	POS	POS	POS	AMB	NEG	NEG	POS	POS	POS	POS	POS	NEG	NEG	POS	POS	POS	NEG	NEG	NEG
2012_281_Z3568 (4696/12)	POS	NEG	AMB	NEG	POS	NEG	NEG	POS	AMB	NEG	POS	POS	NEG	POS	POS	POS	POS	NEG	NEG	NEG	POS	POS	POS	POS	POS	NEG	NEG	POS	POS	POS	NEG	NEG	NEG
2012_324_Z3611 (7180/12)	POS	NEG	AMB	NEG	POS	NEG	NEG	POS	POS	AMB	POS	POS	NEG	POS	POS	POS	POS	NEG	NEG	NEG	POS	POS	POS	POS	POS	NEG	NEG	POS	POS	POS	NEG	NEG	NEG
2012_327_Z3614 (7347/12)	POS	NEG	AMB	NEG	POS	NEG	NEG	POS	POS	AMB	POS	POS	NEG	POS	POS	POS	POS	NEG	NEG	NEG	POS	POS	POS	POS	POS	NEG	NEG	POS	POS	POS	NEG	NEG	NEG
2012_351_Z3638 (8273B/12)	POS	NEG	AMB	NEG	POS	NEG	NEG	POS	POS	NEG	POS	POS	NEG	POS	POS	POS	POS	NEG	NEG	NEG	POS	POS	POS	POS	POS	NEG	NEG	POS	POS	POS	NEG	NEG	NEG
**CC5-MSSA**																																	
2012_188_Z3505 (503/12)	POS	NEG	AMB	POS	NEG	AMB	NEG	POS	POS	NEG	POS	POS	POS	NEG	POS	POS	POS	NEG	NEG	POS	NEG	POS	POS	POS	NEG	POS	NEG	POS	POS	NEG	NEG	POS	NEG
2012_219_Z3518 (1437/12)	POS	NEG	AMB	POS	NEG	AMB	NEG	POS	POS	NEG	POS	POS	POS	NEG	POS	POS	POS	NEG	NEG	POS	AMB	POS	POS	POS	NEG	POS	NEG	POS	POS	NEG	NEG	POS	NEG
2012_329_Z3616 (7577/12)	POS	NEG	AMB	POS	NEG	AMB	NEG	POS	POS	AMB	POS	POS	POS	NEG	POS	POS	POS	NEG	NEG	POS	AMB	POS	POS	POS	NEG	POS	NEG	POS	POS	NEG	NEG	POS	NEG
2012_377_Z3437 (6242)	POS	NEG	AMB	POS	NEG	AMB	NEG	POS	POS	NEG	POS	POS	POS	NEG	POS	POS	POS	NEG	NEG	POS	POS	POS	POS	POS	NEG	POS	NEG	POS	POS	NEG	NEG	POS	NEG
2013_PA04_Z3987	POS	NEG	AMB	POS	NEG	AMB	NEG	POS	POS	NEG	POS	POS	POS	NEG	POS	POS	POS	NEG	NEG	POS	NEG	POS	POS	POS	NEG	POS	NEG	POS	POS	NEG	NEG	POS	NEG
2013_PA32_Z4015	POS	NEG	AMB	POS	NEG	AMB	NEG	POS	POS	NEG	POS	POS	POS	NEG	POS	POS	POS	NEG	NEG	POS	NEG	POS	POS	POS	NEG	POS	NEG	POS	POS	NEG	NEG	POS	NEG
2013_PA41_Z4024	POS	NEG	AMB	POS	NEG	NEG	NEG	POS	AMB	NEG	POS	POS	POS	NEG	POS	POS	POS	NEG	NEG	POS	NEG	POS	POS	POS	NEG	POS	NEG	POS	POS	NEG	NEG	POS	NEG
2013_PA44_Z4027	POS	NEG	AMB	POS	NEG	AMB	NEG	POS	AMB	NEG	POS	POS	POS	NEG	POS	POS	POS	NEG	NEG	POS	NEG	POS	POS	POS	NEG	POS	NEG	POS	POS	NEG	NEG	POS	NEG
**CC5-MSSA [PVL+]**																																	
2012_175_Z3497 (112/12)	POS	NEG	AMB	POS	NEG	AMB	NEG	POS	POS	NEG	POS	POS	POS	NEG	POS	POS	POS	NEG	NEG	POS	AMB	POS	POS	POS	NEG	POS	NEG	POS	POS	NEG	NEG	POS	NEG
2012_193_Z3458 (617/12)	POS	NEG	AMB	POS	NEG	AMB	NEG	POS	POS	NEG	POS	POS	POS	NEG	POS	POS	POS	AMB	NEG	POS	AMB	POS	POS	POS	NEG	POS	NEG	POS	POS	NEG	NEG	POS	NEG
2012_215_Z3474 (1341/12)	POS	NEG	AMB	POS	NEG	AMB	NEG	POS	POS	NEG	POS	POS	POS	NEG	POS	POS	POS	NEG	NEG	POS	NEG	POS	POS	POS	NEG	POS	NEG	POS	POS	NEG	NEG	POS	NEG
2012_216_Z3475 (1370/12)	POS	NEG	AMB	POS	NEG	AMB	NEG	POS	POS	NEG	POS	POS	POS	NEG	POS	POS	POS	NEG	NEG	POS	NEG	POS	POS	POS	NEG	POS	NEG	POS	POS	NEG	NEG	POS	NEG
2012_297_Z3584 (5140/12)	POS	NEG	AMB	POS	NEG	NEG	NEG	POS	POS	NEG	POS	POS	POS	NEG	POS	POS	POS	NEG	NEG	POS	NEG	POS	POS	POS	NEG	POS	NEG	POS	POS	NEG	NEG	POS	NEG
2012_331_Z3618 (7644/12)	POS	NEG	AMB	POS	NEG	AMB	NEG	POS	POS	NEG	POS	POS	POS	NEG	POS	POS	POS	NEG	NEG	POS	AMB	POS	POS	POS	NEG	POS	NEG	POS	POS	NEG	NEG	POS	NEG
2012_379_Z3439 (4126)	POS	NEG	AMB	POS	NEG	AMB	NEG	POS	POS	NEG	POS	POS	POS	NEG	POS	POS	POS	NEG	NEG	POS	NEG	POS	POS	POS	NEG	POS	NEG	POS	POS	NEG	NEG	POS	NEG
2012_381_Z3441	POS	NEG	AMB	POS	NEG	AMB	NEG	POS	POS	AMB	POS	POS	POS	NEG	POS	POS	POS	AMB	NEG	POS	AMB	POS	POS	POS	NEG	POS	NEG	POS	POS	NEG	NEG	POS	NEG
2013_PA39_Z4022	POS	NEG	AMB	POS	NEG	AMB	NEG	POS	POS	NEG	POS	POS	POS	NEG	POS	POS	POS	NEG	NEG	POS	NEG	POS	POS	POS	NEG	POS	NEG	POS	POS	NEG	NEG	POS	NEG
2013_PA47_Z4030	POS	NEG	AMB	POS	NEG	NEG	NEG	POS	NEG	NEG	POS	POS	POS	NEG	POS	POS	POS	NEG	NEG	POS	NEG	POS	POS	POS	NEG	POS	NEG	POS	POS	NEG	NEG	POS	NEG
2013_PA61_I_Z4044	POS	NEG	AMB	POS	NEG	AMB	NEG	POS	POS	NEG	POS	POS	POS	NEG	POS	POS	POS	NEG	NEG	POS	NEG	POS	POS	POS	NEG	POS	NEG	POS	POS	NEG	NEG	POS	NEG
2013_PA62_I_Z4045	POS	NEG	AMB	POS	NEG	AMB	NEG	POS	POS	NEG	POS	POS	POS	NEG	POS	POS	POS	NEG	NEG	POS	NEG	POS	POS	POS	NEG	POS	NEG	POS	POS	NEG	NEG	POS	NEG
2013_PA74_Z4057	POS	NEG	AMB	POS	NEG	AMB	NEG	POS	POS	NEG	POS	POS	POS	NEG	POS	POS	POS	NEG	NEG	POS	NEG	POS	POS	POS	NEG	POS	NEG	POS	POS	NEG	NEG	POS	NEG
2013_PA75_Z4058	POS	NEG	AMB	POS	NEG	NEG	NEG	POS	POS	NEG	POS	POS	POS	NEG	POS	POS	POS	NEG	NEG	POS	NEG	POS	POS	POS	NEG	POS	NEG	POS	POS	NEG	NEG	POS	NEG
**CC5-MRSA-V [ACME+]**																																	
2012_373_Z3433 (1204-17312)	POS	NEG	AMB	POS	NEG	AMB	NEG	POS	POS	AMB	POS	POS	POS	NEG	POS	POS	POS	AMB	NEG	POS	AMB	POS	POS	POS	NEG	POS	NEG	POS	POS	NEG	NEG	POS	NEG
**CC6-MSSA**																																	
2012_173_Z3450 (98A/12)	POS	NEG	AMB	POS	NEG	AMB	NEG	POS	POS	AMB	POS	POS	NEG	POS	POS	POS	POS	AMB	POS	NEG	POS	POS	POS	POS	NEG	POS	NEG	POS	POS	NEG	POS	NEG	NEG
2012_176_Z3498	POS	NEG	AMB	POS	NEG	NEG	NEG	POS	AMB	NEG	POS	POS	NEG	POS	POS	POS	POS	NEG	POS	NEG	AMB	POS	POS	POS	NEG	POS	NEG	POS	POS	NEG	POS	NEG	NEG
2012_181_Z3452	POS	NEG	AMB	POS	NEG	NEG	NEG	POS	AMB	NEG	POS	POS	NEG	POS	POS	POS	POS	NEG	POS	NEG	AMB	POS	POS	POS	NEG	POS	NEG	POS	POS	NEG	POS	NEG	NEG
2012_259_Z3546 (3279/12)	POS	NEG	AMB	POS	NEG	NEG	NEG	POS	POS	NEG	POS	POS	NEG	POS	POS	POS	POS	AMB	POS	NEG	AMB	POS	POS	POS	NEG	POS	NEG	POS	POS	NEG	POS	NEG	NEG
2012_264_Z3551 (3407B/12)	POS	NEG	AMB	POS	NEG	NEG	NEG	POS	POS	NEG	POS	POS	NEG	POS	POS	POS	POS	NEG	POS	NEG	NEG	POS	POS	POS	NEG	POS	NEG	POS	POS	NEG	POS	NEG	NEG
2012_273_Z3560 (3844/12)	POS	NEG	AMB	POS	NEG	NEG	NEG	POS	POS	NEG	POS	POS	NEG	POS	POS	POS	POS	NEG	POS	NEG	NEG	POS	POS	POS	NEG	POS	NEG	POS	POS	NEG	POS	NEG	NEG
2012_312_Z3599 (6815/12)	POS	NEG	AMB	POS	NEG	NEG	NEG	POS	AMB	NEG	POS	POS	NEG	POS	POS	POS	POS	NEG	POS	NEG	NEG	POS	POS	POS	NEG	POS	NEG	POS	POS	NEG	POS	NEG	NEG
2012_338_Z3625	POS	NEG	AMB	POS	NEG	NEG	NEG	POS	AMB	NEG	POS	POS	NEG	POS	POS	POS	POS	NEG	POS	NEG	NEG	POS	POS	POS	NEG	POS	NEG	POS	POS	NEG	POS	NEG	NEG
2012_352_Z3639	POS	NEG	AMB	POS	NEG	NEG	NEG	POS	POS	NEG	POS	POS	NEG	POS	POS	POS	POS	NEG	POS	NEG	NEG	POS	POS	POS	NEG	POS	NEG	POS	POS	NEG	POS	NEG	NEG
2012_368_Z3428	POS	NEG	AMB	POS	NEG	NEG	NEG	POS	AMB	NEG	POS	POS	NEG	POS	POS	POS	POS	NEG	POS	NEG	NEG	POS	POS	POS	NEG	POS	NEG	POS	POS	NEG	POS	NEG	NEG
2013_PA12_Z3995	POS	NEG	AMB	POS	NEG	NEG	NEG	POS	NEG	NEG	POS	POS	NEG	POS	POS	POS	POS	NEG	POS	NEG	NEG	POS	POS	POS	NEG	POS	NEG	POS	POS	NEG	POS	NEG	NEG
2013_PA28_Z4011	POS	NEG	AMB	POS	NEG	NEG	NEG	POS	NEG	NEG	POS	POS	NEG	POS	POS	POS	POS	NEG	POS	NEG	NEG	POS	POS	POS	NEG	POS	NEG	POS	POS	NEG	POS	NEG	NEG
**CC6-MSSA [PVL+]**																																	
2012_375_Z3435	POS	NEG	AMB	POS	NEG	NEG	NEG	POS	AMB	NEG	POS	POS	NEG	POS	POS	POS	POS	NEG	POS	NEG	AMB	POS	POS	POS	NEG	POS	NEG	POS	POS	NEG	POS	NEG	NEG
2013_PA77_Z4060	POS	NEG	AMB	POS	NEG	NEG	NEG	POS	AMB	NEG	POS	POS	NEG	POS	POS	POS	POS	NEG	POS	NEG	NEG	POS	POS	POS	NEG	POS	NEG	POS	POS	NEG	POS	NEG	NEG
**CC7-MSSA**																																	
2012_248_Z3532 (2864/12)	POS	NEG	AMB	NEG	NEG	NEG	POS	POS	AMB	NEG	POS	NEG	NEG	NEG	NEG	POS	POS	NEG	POS	NEG	NEG	POS	POS	POS	NEG	POS	NEG	POS	POS	NEG	NEG	NEG	NEG
2012_265_Z3552 (3518/12)	POS	NEG	AMB	AMB	NEG	NEG	POS	POS	POS	NEG	POS	POS	NEG	AMB	POS	POS	POS	AMB	POS	NEG	AMB	POS	NEG	AMB	NEG	AMB	NEG	POS	POS	NEG	AMB	NEG	NEG
2013_PA53_Z4036	POS	NEG	AMB	NEG	NEG	NEG	POS	POS	POS	NEG	POS	NEG	NEG	NEG	NEG	POS	POS	NEG	POS	NEG	NEG	POS	POS	POS	NEG	POS	NEG	POS	POS	NEG	NEG	POS	NEG
2013_PA83_Z4066	POS	NEG	AMB	NEG	NEG	NEG	POS	POS	AMB	NEG	POS	NEG	NEG	NEG	NEG	POS	POS	NEG	POS	NEG	NEG	POS	POS	POS	NEG	POS	NEG	POS	POS	NEG	NEG	AMB	NEG
**CC8-MSSA**																																	
2012_243_Z3528 (2587/12)	POS	POS	AMB	AMB	NEG	NEG	NEG	POS	POS	NEG	AMB	POS	POS	NEG	POS	POS	POS	NEG	POS	NEG	NEG	POS	POS	POS	POS	NEG	NEG	POS	POS	POS	NEG	NEG	NEG
2012_344_Z3631 (8605/12)	POS	AMB	AMB	POS	NEG	NEG	NEG	POS	POS	NEG	AMB	POS	POS	NEG	POS	POS	POS	AMB	POS	NEG	NEG	POS	POS	POS	POS	NEG	NEG	POS	POS	POS	NEG	NEG	NEG
2012_346_Z3633 (8074/12)	POS	AMB	AMB	POS	NEG	NEG	NEG	POS	POS	NEG	NEG	POS	POS	NEG	POS	POS	POS	AMB	POS	NEG	NEG	POS	NEG	NEG	NEG	NEG	NEG	POS	POS	POS	NEG	NEG	NEG
2012_255_Z3538 (3205B/12)	POS	POS	AMB	AMB	NEG	NEG	NEG	POS	POS	NEG	POS	POS	POS	NEG	POS	POS	POS	AMB	POS	NEG	NEG	POS	POS	POS	POS	NEG	NEG	POS	POS	POS	NEG	NEG	NEG
2012_280_Z3567 (4601/12)	POS	AMB	AMB	POS	NEG	NEG	NEG	POS	POS	NEG	POS	POS	POS	NEG	POS	POS	POS	AMB	POS	NEG	NEG	POS	POS	POS	POS	NEG	NEG	POS	POS	POS	NEG	NEG	NEG
Trinidad&Tobago_2012_267 (3642/12)	POS	POS	AMB	NEG	NEG	NEG	NEG	POS	POS	NEG	AMB	POS	POS	NEG	POS	POS	POS	NEG	POS	NEG	NEG	POS	POS	POS	POS	NEG	NEG	POS	POS	POS	NEG	NEG	NEG
2012_365_Z3425 (1204-15838)	POS	AMB	AMB	POS	NEG	NEG	NEG	POS	POS	NEG	POS	POS	POS	NEG	POS	POS	POS	AMB	POS	NEG	AMB	POS	POS	POS	POS	NEG	NEG	POS	POS	POS	NEG	NEG	NEG
2012_296_Z3583 (5118/12)	POS	AMB	AMB	POS	NEG	NEG	NEG	POS	POS	NEG	AMB	POS	POS	NEG	POS	POS	POS	NEG	POS	NEG	NEG	POS	POS	POS	POS	NEG	NEG	POS	POS	POS	NEG	NEG	NEG
2012_318_Z3605 (6960/12)	POS	POS	AMB	AMB	NEG	NEG	NEG	POS	POS	NEG	POS	POS	POS	NEG	POS	POS	POS	NEG	POS	NEG	NEG	AMB	POS	POS	POS	NEG	NEG	POS	POS	POS	NEG	NEG	NEG
2012_306_Z3593 (5861/12)	POS	AMB	AMB	POS	NEG	NEG	NEG	POS	POS	NEG	POS	POS	POS	NEG	POS	POS	POS	AMB	POS	NEG	AMB	POS	NEG	NEG	NEG	NEG	NEG	POS	POS	POS	NEG	NEG	NEG
2013_PA37_Z4020	POS	POS	AMB	AMB	NEG	NEG	NEG	POS	POS	NEG	AMB	POS	POS	NEG	POS	POS	POS	NEG	POS	NEG	NEG	POS	POS	POS	POS	NEG	NEG	POS	POS	POS	NEG	NEG	NEG
2013_PA38_Z4021	POS	POS	AMB	NEG	NEG	NEG	NEG	POS	POS	NEG	NEG	POS	POS	NEG	POS	POS	POS	NEG	POS	NEG	NEG	POS	POS	POS	POS	NEG	NEG	POS	POS	POS	NEG	NEG	NEG
2013_PA52_Z4035	POS	POS	AMB	NEG	NEG	NEG	NEG	POS	POS	NEG	NEG	POS	POS	NEG	POS	POS	POS	NEG	POS	NEG	NEG	POS	POS	POS	POS	NEG	NEG	POS	POS	POS	NEG	NEG	NEG
**CC8-MSSA [PVL+]**																																	
2012_156_Z3489 (25917/11)	POS	AMB	AMB	POS	NEG	NEG	NEG	POS	POS	NEG	AMB	POS	POS	NEG	POS	POS	POS	NEG	POS	NEG	NEG	POS	POS	POS	POS	NEG	NEG	POS	POS	POS	NEG	NEG	NEG
2012_159_Z3492 (26310/11)	POS	AMB	AMB	POS	NEG	NEG	NEG	POS	POS	NEG	POS	POS	POS	NEG	POS	POS	POS	NEG	POS	NEG	AMB	POS	POS	POS	POS	NEG	NEG	POS	POS	POS	NEG	NEG	NEG
2012_161_Z3445 (26430/11)	POS	AMB	AMB	POS	NEG	NEG	AMB	POS	POS	NEG	POS	POS	POS	NEG	POS	POS	POS	AMB	POS	NEG	POS	POS	POS	POS	POS	NEG	NEG	POS	POS	POS	NEG	NEG	NEG
2012_162_Z3512 (26433/11)	POS	AMB	AMB	POS	NEG	NEG	NEG	POS	POS	NEG	POS	POS	POS	NEG	POS	POS	POS	NEG	POS	NEG	NEG	POS	POS	POS	POS	NEG	NEG	POS	POS	POS	NEG	NEG	NEG
2012_169_Z3495 (27194/11)	POS	AMB	AMB	POS	NEG	NEG	NEG	POS	POS	NEG	AMB	POS	POS	NEG	POS	POS	POS	AMB	POS	NEG	AMB	POS	POS	POS	POS	NEG	NEG	POS	POS	POS	NEG	NEG	NEG
2012_170_Z3496 (35/12)	POS	AMB	AMB	POS	NEG	NEG	NEG	POS	POS	NEG	AMB	POS	POS	NEG	POS	POS	POS	NEG	POS	NEG	AMB	POS	POS	POS	POS	NEG	NEG	POS	POS	POS	NEG	NEG	NEG
2012_171_Z3448 (47/12)	POS	AMB	AMB	POS	NEG	NEG	NEG	POS	POS	NEG	POS	POS	POS	NEG	POS	POS	POS	NEG	POS	NEG	AMB	POS	POS	POS	POS	NEG	NEG	POS	POS	POS	NEG	NEG	NEG
2012_177_Z3499 (184/12)	POS	AMB	AMB	POS	NEG	NEG	NEG	POS	POS	NEG	AMB	POS	POS	NEG	POS	POS	POS	AMB	POS	NEG	AMB	POS	POS	POS	POS	NEG	NEG	POS	POS	POS	NEG	NEG	NEG
2012_179_Z3515 (231/12)	POS	AMB	AMB	POS	NEG	NEG	NEG	POS	POS	NEG	POS	POS	POS	NEG	POS	POS	POS	AMB	POS	NEG	AMB	POS	POS	POS	POS	NEG	NEG	POS	POS	POS	NEG	NEG	NEG
2012_185_Z3502 (437/12)	POS	POS	AMB	AMB	NEG	NEG	NEG	POS	POS	NEG	AMB	POS	POS	NEG	POS	POS	POS	NEG	POS	NEG	NEG	POS	POS	POS	POS	NEG	NEG	POS	POS	POS	NEG	NEG	NEG
2012_191_Z3456 (563/12)	POS	AMB	AMB	POS	NEG	NEG	NEG	POS	POS	NEG	AMB	POS	POS	NEG	POS	POS	POS	AMB	POS	NEG	NEG	POS	POS	POS	POS	NEG	NEG	POS	POS	POS	NEG	NEG	NEG
2012_194_Z3459	POS	AMB	AMB	POS	NEG	NEG	NEG	POS	POS	NEG	AMB	POS	POS	NEG	POS	POS	POS	AMB	POS	NEG	AMB	POS	POS	POS	POS	NEG	NEG	POS	POS	POS	NEG	NEG	NEG
2012_200_Z3460 (820/12)	POS	AMB	AMB	POS	NEG	NEG	NEG	POS	POS	NEG	POS	POS	POS	NEG	POS	POS	POS	NEG	POS	NEG	AMB	POS	POS	POS	POS	NEG	NEG	POS	POS	POS	NEG	NEG	NEG
2012_207_Z3467 (1010/12)	POS	AMB	AMB	POS	NEG	NEG	NEG	POS	POS	NEG	AMB	POS	POS	NEG	POS	POS	POS	AMB	POS	NEG	NEG	POS	POS	POS	POS	NEG	NEG	POS	POS	POS	NEG	NEG	NEG
2012_212_Z3471 (1261/12)	POS	POS	AMB	AMB	NEG	NEG	NEG	POS	POS	NEG	NEG	POS	POS	NEG	POS	POS	POS	NEG	POS	NEG	NEG	POS	POS	POS	POS	NEG	NEG	POS	POS	POS	NEG	NEG	NEG
2012_220_Z3477 (1466/12)	POS	POS	AMB	AMB	NEG	NEG	NEG	POS	POS	NEG	AMB	POS	POS	NEG	POS	POS	POS	NEG	POS	NEG	NEG	POS	POS	POS	POS	NEG	NEG	POS	POS	POS	NEG	NEG	NEG
2012_224_Z3481 (1522/12)	POS	POS	AMB	AMB	NEG	NEG	NEG	POS	POS	NEG	AMB	POS	POS	NEG	POS	POS	POS	NEG	POS	NEG	NEG	POS	POS	POS	POS	NEG	NEG	POS	POS	POS	NEG	NEG	NEG
2012_226_Z3483 (1662/12)	POS	AMB	AMB	POS	NEG	NEG	NEG	POS	POS	NEG	POS	POS	POS	NEG	POS	POS	POS	NEG	POS	NEG	AMB	POS	POS	POS	POS	NEG	NEG	POS	POS	POS	NEG	NEG	NEG
2012_242_Z3527 (2581/12)	POS	AMB	AMB	POS	NEG	NEG	NEG	POS	POS	NEG	AMB	POS	POS	NEG	POS	POS	POS	NEG	POS	NEG	AMB	POS	POS	POS	POS	NEG	NEG	POS	POS	POS	NEG	NEG	NEG
2012_245_Z3529 (2687/12)	POS	AMB	AMB	POS	NEG	NEG	NEG	POS	POS	NEG	POS	POS	POS	NEG	POS	POS	POS	AMB	POS	NEG	NEG	POS	POS	POS	POS	NEG	NEG	POS	POS	POS	NEG	NEG	NEG
2012_253_Z3536 (3074/12)	POS	POS	AMB	AMB	NEG	NEG	NEG	POS	POS	NEG	POS	POS	POS	NEG	POS	POS	POS	AMB	POS	NEG	AMB	POS	POS	POS	POS	NEG	NEG	POS	POS	POS	NEG	NEG	NEG
2012_258_Z3545 (3231/12)	POS	AMB	AMB	POS	NEG	NEG	NEG	POS	POS	NEG	POS	POS	POS	NEG	POS	POS	POS	AMB	POS	NEG	AMB	POS	POS	POS	POS	NEG	NEG	POS	POS	POS	NEG	NEG	NEG
2012_261_Z3548 (3298/12)	POS	AMB	AMB	POS	NEG	NEG	NEG	POS	POS	NEG	POS	POS	POS	NEG	POS	POS	POS	AMB	POS	NEG	AMB	POS	POS	POS	POS	NEG	NEG	POS	POS	POS	NEG	NEG	NEG
2012_270_Z3557 (3798/12)	POS	POS	AMB	AMB	NEG	NEG	NEG	POS	POS	NEG	POS	POS	POS	POS	POS	POS	POS	AMB	POS	NEG	NEG	POS	POS	POS	POS	NEG	AMB	POS	POS	POS	NEG	NEG	NEG
2012_274_Z3561 (3861/12)	POS	POS	AMB	AMB	NEG	NEG	NEG	POS	POS	NEG	AMB	POS	POS	NEG	POS	POS	POS	NEG	POS	NEG	NEG	POS	POS	POS	POS	NEG	NEG	POS	POS	POS	NEG	NEG	NEG
2012_282_Z3569 (4698/12)	POS	AMB	AMB	POS	NEG	NEG	NEG	POS	POS	NEG	AMB	POS	POS	NEG	POS	POS	POS	NEG	POS	NEG	NEG	POS	POS	POS	POS	NEG	NEG	POS	POS	POS	NEG	NEG	NEG
2012_286_Z3573 (4805/12)	POS	AMB	AMB	POS	NEG	NEG	NEG	POS	POS	NEG	AMB	POS	POS	NEG	POS	POS	POS	AMB	POS	NEG	NEG	POS	POS	POS	POS	NEG	NEG	POS	POS	POS	NEG	NEG	NEG
2012_293_Z3580 (5007/12)	POS	POS	AMB	AMB	NEG	NEG	NEG	POS	POS	NEG	POS	POS	POS	NEG	POS	POS	POS	NEG	POS	NEG	NEG	POS	POS	POS	POS	NEG	NEG	POS	POS	POS	NEG	NEG	NEG
2012_294_Z3581	POS	AMB	AMB	POS	NEG	NEG	NEG	POS	POS	NEG	AMB	POS	POS	NEG	POS	POS	POS	AMB	POS	NEG	NEG	POS	POS	POS	POS	NEG	NEG	POS	POS	POS	NEG	NEG	NEG
2012_307_Z3594 (5918/12)	POS	AMB	AMB	POS	NEG	NEG	NEG	POS	POS	NEG	POS	POS	POS	NEG	POS	POS	POS	AMB	POS	NEG	NEG	POS	POS	POS	POS	NEG	NEG	POS	POS	POS	NEG	NEG	NEG
2012_333_Z3620 (8101/12)	POS	AMB	AMB	POS	NEG	NEG	NEG	POS	POS	NEG	POS	POS	POS	NEG	POS	POS	POS	NEG	POS	NEG	AMB	POS	POS	POS	POS	NEG	NEG	POS	POS	POS	NEG	NEG	NEG
2012_343_Z3630 (8195/12)	POS	AMB	AMB	POS	NEG	NEG	NEG	POS	POS	NEG	POS	POS	POS	NEG	POS	POS	POS	NEG	POS	NEG	NEG	POS	POS	POS	POS	NEG	NEG	POS	POS	POS	NEG	NEG	NEG
2012_349_Z3636 (7999A/12)	POS	POS	AMB	AMB	NEG	NEG	NEG	POS	POS	NEG	POS	POS	POS	NEG	POS	POS	POS	AMB	POS	NEG	NEG	POS	POS	POS	POS	NEG	NEG	POS	POS	POS	NEG	NEG	NEG
2012_353_Z3640 (8575/12)	POS	AMB	AMB	POS	NEG	NEG	NEG	POS	POS	NEG	POS	POS	POS	NEG	POS	POS	POS	AMB	POS	NEG	AMB	POS	POS	POS	POS	NEG	NEG	POS	POS	POS	NEG	NEG	NEG
2012_354_Z3641 (8408/12)	POS	AMB	AMB	POS	NEG	NEG	NEG	POS	POS	NEG	POS	POS	POS	NEG	POS	POS	POS	AMB	POS	NEG	AMB	POS	POS	POS	POS	NEG	NEG	POS	POS	POS	NEG	NEG	NEG
2012_355_Z3642 (1112-58348)	POS	POS	AMB	AMB	NEG	NEG	NEG	POS	POS	NEG	AMB	POS	POS	NEG	POS	POS	POS	NEG	POS	NEG	NEG	POS	POS	POS	POS	NEG	NEG	POS	POS	POS	NEG	NEG	NEG
2012_357_Z3417 (1202-6360)	POS	AMB	AMB	POS	NEG	NEG	NEG	POS	POS	NEG	POS	POS	POS	NEG	POS	POS	POS	AMB	POS	NEG	AMB	POS	POS	POS	POS	NEG	NEG	POS	POS	POS	NEG	NEG	NEG
2012_364_Z3424 (1112-60417)	POS	AMB	AMB	POS	NEG	NEG	NEG	POS	POS	NEG	POS	POS	POS	NEG	POS	POS	POS	NEG	POS	NEG	AMB	POS	POS	POS	POS	NEG	NEG	POS	POS	POS	NEG	NEG	NEG
2012_376_Z3436 (6256)	POS	AMB	AMB	POS	NEG	NEG	NEG	POS	POS	NEG	POS	POS	POS	NEG	POS	POS	POS	AMB	POS	NEG	POS	POS	POS	POS	POS	NEG	NEG	POS	POS	POS	NEG	NEG	NEG
2012_383_Z3443	POS	POS	AMB	AMB	NEG	NEG	NEG	POS	POS	NEG	POS	POS	POS	NEG	POS	POS	POS	NEG	POS	NEG	AMB	POS	POS	POS	POS	NEG	NEG	POS	POS	POS	NEG	NEG	NEG
2013_PA01_Z3984	POS	POS	AMB	AMB	NEG	NEG	NEG	POS	POS	NEG	AMB	POS	POS	NEG	POS	POS	POS	NEG	POS	NEG	NEG	POS	POS	POS	POS	NEG	NEG	POS	POS	POS	NEG	NEG	NEG
2013_PA08_Z3991	POS	POS	AMB	AMB	NEG	NEG	NEG	POS	POS	NEG	POS	POS	POS	NEG	POS	POS	POS	NEG	POS	NEG	NEG	POS	POS	POS	POS	NEG	NEG	POS	POS	POS	NEG	NEG	NEG
2013_PA10_Z3993	POS	POS	AMB	AMB	NEG	NEG	NEG	POS	POS	NEG	AMB	POS	POS	NEG	POS	POS	POS	NEG	POS	NEG	NEG	POS	POS	POS	POS	NEG	NEG	POS	POS	POS	NEG	NEG	NEG
2013_PA13_Z3996	POS	POS	AMB	AMB	NEG	NEG	NEG	POS	POS	NEG	AMB	POS	POS	NEG	POS	POS	POS	NEG	POS	NEG	NEG	POS	POS	POS	POS	NEG	NEG	POS	POS	POS	NEG	NEG	NEG
2013_PA14_Z3997	POS	POS	AMB	AMB	NEG	NEG	NEG	POS	POS	NEG	AMB	POS	POS	NEG	POS	POS	POS	NEG	POS	NEG	NEG	POS	POS	POS	POS	NEG	NEG	POS	POS	POS	NEG	NEG	NEG
2013_PA17_Z4000	POS	POS	AMB	AMB	NEG	NEG	NEG	POS	POS	NEG	NEG	POS	POS	NEG	POS	POS	POS	NEG	POS	NEG	NEG	POS	POS	POS	POS	NEG	NEG	POS	POS	POS	NEG	NEG	NEG
2013_PA26_Z4009	POS	POS	AMB	NEG	NEG	NEG	NEG	POS	POS	NEG	NEG	POS	POS	NEG	POS	POS	POS	NEG	POS	NEG	NEG	POS	POS	POS	POS	NEG	NEG	POS	POS	POS	NEG	NEG	NEG
2013_PA27_Z4010	POS	POS	AMB	AMB	NEG	NEG	NEG	POS	POS	NEG	AMB	POS	POS	NEG	POS	POS	POS	NEG	POS	NEG	NEG	POS	POS	POS	POS	NEG	NEG	POS	POS	POS	NEG	NEG	NEG
2013_PA42_Z4025	POS	POS	AMB	AMB	NEG	NEG	NEG	POS	POS	NEG	NEG	POS	POS	NEG	POS	POS	POS	NEG	POS	NEG	NEG	POS	POS	POS	POS	NEG	NEG	POS	POS	POS	NEG	NEG	NEG
2013_PA43_Z4026	POS	POS	AMB	NEG	NEG	NEG	NEG	POS	POS	NEG	NEG	POS	POS	NEG	POS	POS	POS	NEG	POS	NEG	NEG	POS	POS	POS	POS	NEG	NEG	POS	POS	POS	NEG	NEG	NEG
2013_PA45_Z4028	POS	POS	AMB	AMB	NEG	NEG	NEG	POS	POS	NEG	NEG	POS	POS	NEG	POS	POS	POS	NEG	POS	NEG	NEG	POS	POS	POS	POS	NEG	NEG	POS	POS	POS	NEG	NEG	NEG
2013_PA56_Z4039	POS	POS	AMB	AMB	NEG	NEG	NEG	POS	POS	NEG	NEG	POS	POS	NEG	POS	POS	POS	NEG	POS	NEG	NEG	POS	POS	POS	POS	NEG	NEG	POS	POS	POS	NEG	NEG	NEG
2013_PA58_Z4041	POS	POS	AMB	AMB	NEG	NEG	NEG	POS	POS	NEG	POS	POS	POS	NEG	POS	POS	POS	NEG	POS	NEG	AMB	POS	POS	POS	POS	NEG	NEG	POS	POS	POS	NEG	NEG	NEG
2013_PA64_Z4047	POS	POS	AMB	AMB	NEG	NEG	NEG	POS	POS	NEG	AMB	POS	POS	NEG	POS	POS	POS	NEG	POS	NEG	NEG	POS	POS	POS	POS	NEG	NEG	POS	POS	POS	NEG	NEG	NEG
2013_PA66_Z4049	POS	POS	AMB	AMB	NEG	NEG	NEG	POS	POS	NEG	NEG	POS	POS	NEG	POS	POS	POS	NEG	POS	NEG	NEG	POS	POS	POS	POS	NEG	NEG	POS	POS	POS	NEG	NEG	NEG
2013_PA67_Z4050	POS	POS	AMB	AMB	NEG	NEG	NEG	POS	POS	NEG	AMB	POS	POS	NEG	POS	POS	POS	NEG	POS	NEG	NEG	POS	POS	POS	POS	NEG	NEG	POS	POS	POS	NEG	NEG	NEG
2013_PA69_Z4052	POS	AMB	POS	AMB	NEG	NEG	NEG	POS	POS	NEG	NEG	POS	POS	NEG	POS	POS	POS	NEG	POS	NEG	NEG	POS	POS	POS	POS	NEG	NEG	POS	POS	POS	NEG	NEG	NEG
2013_PA72_Z4055	POS	POS	AMB	AMB	NEG	NEG	NEG	POS	POS	NEG	AMB	POS	POS	NEG	POS	POS	POS	NEG	POS	NEG	NEG	POS	POS	POS	POS	NEG	NEG	POS	POS	POS	NEG	NEG	NEG
2013_PA76_Z4059	POS	POS	AMB	AMB	NEG	NEG	NEG	POS	POS	NEG	AMB	POS	POS	NEG	POS	POS	POS	NEG	POS	NEG	NEG	POS	POS	POS	POS	NEG	NEG	POS	POS	POS	NEG	NEG	NEG
2013_PA80_Z4063	POS	POS	AMB	AMB	NEG	NEG	NEG	POS	POS	NEG	AMB	POS	POS	NEG	POS	POS	POS	NEG	POS	NEG	NEG	POS	POS	POS	POS	NEG	NEG	POS	POS	POS	NEG	NEG	NEG

Strain / Isolates	fibronectin-binding protein B							Major histocompatibility complex class II analog protein (=Extracellular adherence protein, eap)				Staphylococcus aureus surface protein G				Ser-Asp rich fibrinogen-/bone sialoprotein-binding protein C							Ser-Asp rich fibrinogen-/bone sialoprotein-binding protein D					van Willebrand factor binding protein					
	fnbB (total)	fnbB (COL)	fnbB (COL+Mu50+MW2)	fnbB (Mu50)	fnbB (MW2)	fnbB (ST15)	fnbB (ST45-2)	map (total)	map (COL)	map (MRSA252)	map (Mu50+MW2)	sasG (total)	sasG (COL+Mu50)	sasG (MW2)	sasG (other than MRSA252+RF122)	sdrC (total)	sdrC (consensus)	sdrC (B1)	sdrC (COL)	sdrC (Mu50)	sdrC (MW2+MRSA252+RF122)	sdrC (other than MRSA252+RF122)	sdrD (total)	sdrD (consensus)	sdrD (COL+MW2)	sdrD (Mu50)	sdrD (other)	vwb (total)	vwb (consensus)	vwb (COL+MW2)	vwb (MRSA252)	vwb (Mu50)	vwb (RF122)
**ST8-MRSA-IV, USA300**																																	
2012_158_Z3491 (26049A/11)	POS	AMB	AMB	POS	NEG	NEG	NEG	POS	POS	NEG	AMB	POS	POS	NEG	POS	POS	POS	NEG	POS	NEG	NEG	POS	POS	POS	POS	NEG	NEG	POS	POS	POS	NEG	NEG	NEG
2012_165_Z3513 (26656/11)	POS	AMB	AMB	POS	NEG	NEG	NEG	POS	POS	NEG	AMB	POS	POS	NEG	POS	POS	POS	NEG	POS	NEG	AMB	POS	POS	POS	POS	NEG	NEG	POS	POS	POS	NEG	NEG	NEG
2012_214_Z3473 (1322/12)	POS	POS	AMB	AMB	NEG	NEG	NEG	POS	POS	NEG	NEG	POS	POS	NEG	POS	POS	POS	NEG	POS	NEG	NEG	POS	POS	POS	POS	NEG	NEG	POS	POS	POS	NEG	NEG	NEG
2012_227_Z3484 (1688/12)	POS	AMB	AMB	POS	NEG	NEG	NEG	POS	POS	NEG	POS	POS	POS	NEG	POS	POS	POS	NEG	POS	NEG	AMB	POS	POS	POS	POS	NEG	NEG	POS	POS	POS	NEG	NEG	NEG
2012_263_Z3550 (3355/12)	POS	AMB	AMB	POS	NEG	NEG	NEG	POS	POS	NEG	POS	POS	POS	NEG	POS	POS	POS	AMB	POS	NEG	AMB	POS	POS	POS	POS	NEG	NEG	POS	POS	POS	NEG	NEG	NEG
2012_266_Z3553	POS	AMB	AMB	POS	NEG	NEG	NEG	POS	POS	NEG	POS	POS	POS	NEG	POS	POS	POS	AMB	POS	NEG	AMB	POS	POS	POS	POS	NEG	NEG	POS	POS	POS	NEG	NEG	NEG
2012_284_Z3571 (4729/12)	POS	POS	AMB	AMB	NEG	NEG	NEG	POS	POS	NEG	AMB	POS	POS	NEG	POS	POS	POS	NEG	POS	NEG	NEG	POS	POS	POS	POS	NEG	NEG	POS	POS	POS	NEG	NEG	NEG
2012_298_Z3585 (5203/12)	POS	AMB	AMB	POS	NEG	NEG	NEG	POS	POS	NEG	AMB	POS	POS	NEG	POS	POS	POS	NEG	POS	NEG	NEG	POS	POS	POS	POS	NEG	NEG	POS	POS	POS	NEG	NEG	NEG
2012_304_Z3591 (5818/12)	POS	POS	AMB	AMB	NEG	NEG	NEG	POS	POS	NEG	AMB	POS	POS	NEG	POS	POS	POS	NEG	POS	NEG	NEG	POS	POS	POS	POS	NEG	NEG	POS	POS	POS	NEG	NEG	NEG
Z3595_TT308 - (EFC74B77-3925-484F-A4CF-A1F7DC	POS	POS	AMB	AMB	NEG	NEG	NEG	POS	POS	NEG	NEG	POS	POS	NEG	POS	POS	POS	NEG	POS	NEG	NEG	POS	POS	POS	POS	NEG	NEG	POS	POS	POS	NEG	NEG	NEG
2012_369_Z3429 (1112-58547)	POS	AMB	AMB	POS	NEG	NEG	NEG	POS	POS	NEG	POS	POS	POS	NEG	POS	POS	POS	NEG	POS	NEG	AMB	POS	POS	POS	POS	NEG	NEG	POS	POS	POS	NEG	NEG	NEG
2012_374_Z3434 (1203-13527)	POS	AMB	AMB	POS	NEG	NEG	NEG	POS	POS	NEG	POS	POS	POS	NEG	POS	POS	POS	AMB	POS	NEG	AMB	POS	POS	POS	POS	NEG	NEG	POS	POS	POS	NEG	NEG	NEG
2013_PA06_Z3989	POS	POS	AMB	AMB	NEG	NEG	NEG	POS	POS	NEG	AMB	POS	POS	NEG	POS	POS	POS	NEG	POS	NEG	NEG	POS	POS	POS	POS	NEG	NEG	POS	POS	POS	NEG	NEG	NEG
2013_PA55_Z4038	POS	POS	AMB	AMB	NEG	NEG	NEG	POS	POS	NEG	AMB	POS	POS	NEG	POS	POS	POS	NEG	POS	NEG	NEG	POS	POS	POS	POS	NEG	NEG	POS	POS	POS	NEG	NEG	NEG
2013_PA71_Z4054	POS	POS	AMB	AMB	NEG	NEG	NEG	POS	POS	NEG	NEG	POS	POS	NEG	POS	POS	POS	NEG	POS	NEG	NEG	POS	POS	POS	POS	NEG	NEG	POS	POS	POS	NEG	NEG	NEG
**ST8-MRSA-IV [PVL+/ACME+], US**																																	
2012_180_Z3501 (232/12)	POS	POS	AMB	AMB	NEG	NEG	NEG	POS	POS	NEG	AMB	POS	POS	NEG	POS	POS	POS	NEG	POS	NEG	NEG	POS	POS	POS	POS	NEG	NEG	POS	POS	POS	NEG	NEG	NEG
2013_PA68_Z4051	POS	POS	AMB	AMB	NEG	NEG	NEG	POS	POS	NEG	AMB	POS	POS	NEG	POS	POS	POS	NEG	POS	NEG	NEG	POS	POS	POS	POS	NEG	NEG	POS	POS	POS	NEG	NEG	NEG
**ST72-MSSA**																																	
2012_174_Z3451 (102/12)	POS	NEG	AMB	POS	NEG	NEG	NEG	POS	POS	NEG	POS	POS	NEG	POS	POS	POS	POS	NEG	NEG	POS	POS	POS	POS	POS	NEG	POS	NEG	POS	POS	NEG	NEG	NEG	NEG
2012_223_Z3480 (1506A/12)	POS	NEG	POS	AMB	NEG	NEG	NEG	POS	AMB	NEG	POS	POS	NEG	POS	POS	POS	POS	NEG	NEG	POS	POS	POS	POS	POS	NEG	POS	NEG	POS	POS	NEG	NEG	NEG	NEG
2012_236_Z3523 (2086/12)	POS	NEG	AMB	POS	NEG	NEG	NEG	POS	AMB	NEG	POS	POS	NEG	POS	POS	POS	POS	NEG	NEG	POS	POS	POS	POS	POS	NEG	POS	NEG	POS	POS	NEG	NEG	NEG	NEG
2012_239_Z3525 (2346/12)	POS	NEG	AMB	POS	NEG	NEG	NEG	POS	AMB	NEG	POS	POS	NEG	POS	POS	POS	POS	NEG	NEG	POS	POS	POS	POS	POS	NEG	POS	NEG	POS	POS	NEG	NEG	NEG	NEG
2012_252_Z3535 (3030/12)	POS	NEG	AMB	POS	NEG	NEG	NEG	POS	POS	AMB	POS	POS	NEG	POS	POS	POS	POS	AMB	NEG	POS	POS	POS	POS	POS	NEG	POS	NEG	POS	POS	NEG	NEG	NEG	NEG
2012_271_Z3558 (3799/12)	POS	NEG	AMB	POS	NEG	NEG	NEG	POS	AMB	NEG	POS	POS	NEG	POS	POS	POS	POS	NEG	NEG	POS	POS	POS	POS	POS	POS	POS	NEG	POS	POS	NEG	NEG	NEG	NEG
2012_276_Z3563	POS	NEG	AMB	POS	NEG	NEG	NEG	POS	POS	NEG	POS	POS	NEG	POS	POS	POS	POS	NEG	NEG	POS	POS	POS	POS	POS	NEG	POS	NEG	POS	POS	NEG	NEG	NEG	NEG
2012_300_Z3587 (5638/12)	POS	NEG	AMB	POS	NEG	NEG	NEG	POS	AMB	NEG	POS	POS	NEG	POS	POS	POS	POS	NEG	NEG	POS	POS	POS	POS	POS	NEG	POS	NEG	POS	POS	NEG	NEG	NEG	NEG
2012_316_Z3603 (6941/12)	POS	NEG	AMB	POS	NEG	NEG	NEG	POS	AMB	NEG	POS	POS	NEG	POS	POS	POS	POS	NEG	NEG	POS	POS	POS	POS	POS	NEG	POS	NEG	POS	POS	NEG	NEG	NEG	NEG
2012_319_Z3606 (7019/12)	POS	NEG	AMB	POS	NEG	NEG	NEG	POS	AMB	NEG	POS	POS	NEG	POS	POS	POS	POS	NEG	NEG	POS	POS	POS	POS	POS	NEG	POS	NEG	POS	POS	NEG	NEG	NEG	NEG
2012_371_Z3431 (1204-17577)	POS	NEG	AMB	POS	NEG	NEG	NEG	POS	POS	NEG	POS	POS	NEG	POS	POS	POS	POS	NEG	NEG	POS	POS	POS	POS	POS	NEG	POS	NEG	POS	POS	NEG	NEG	NEG	NEG
2013_PA05_Z3988	POS	NEG	POS	AMB	NEG	NEG	NEG	POS	AMB	NEG	POS	POS	NEG	POS	POS	POS	POS	NEG	NEG	POS	POS	POS	POS	POS	NEG	POS	NEG	POS	POS	NEG	NEG	NEG	NEG
2013_PA65_Z4048	POS	NEG	POS	NEG	NEG	NEG	NEG	POS	NEG	NEG	POS	POS	NEG	POS	POS	POS	POS	NEG	NEG	POS	AMB	POS	POS	POS	NEG	POS	NEG	POS	POS	NEG	NEG	NEG	NEG
2013_PA73_Z4056	POS	NEG	POS	AMB	NEG	NEG	NEG	POS	AMB	NEG	POS	POS	NEG	POS	POS	POS	POS	NEG	NEG	POS	POS	POS	POS	POS	NEG	POS	NEG	POS	POS	NEG	NEG	NEG	NEG
**ST72-MSSA-SCCfus**																																	
2013_PA16_Z3999	POS	NEG	POS	AMB	NEG	NEG	NEG	POS	NEG	NEG	POS	POS	NEG	POS	POS	POS	POS	NEG	NEG	POS	POS	POS	POS	POS	NEG	POS	NEG	POS	POS	NEG	NEG	NEG	NEG
**ST72-MSSA-SCCfus/kdp**																																	
2012_361_Z3421 (1201-1299)	POS	NEG	AMB	POS	NEG	NEG	NEG	POS	NEG	NEG	POS	POS	NEG	POS	POS	POS	POS	NEG	NEG	POS	POS	POS	POS	POS	NEG	POS	NEG	POS	POS	NEG	NEG	NEG	NEG
**ST239-MRSA-III&SCCmer**																																	
2012_157_Z3490	POS	AMB	AMB	POS	NEG	NEG	NEG	POS	POS	NEG	AMB	POS	POS	NEG	POS	POS	POS	NEG	POS	NEG	AMB	POS	POS	POS	POS	NEG	NEG	POS	POS	POS	NEG	NEG	NEG
2012_168_Z3494 (27129/11)	POS	AMB	AMB	POS	NEG	NEG	NEG	POS	POS	NEG	AMB	POS	POS	NEG	POS	POS	POS	NEG	POS	NEG	NEG	POS	POS	POS	POS	NEG	NEG	POS	POS	POS	NEG	NEG	NEG
2012_195_Z3508 (619/12)	POS	POS	AMB	AMB	NEG	NEG	NEG	POS	POS	NEG	AMB	POS	POS	NEG	POS	POS	POS	NEG	POS	NEG	NEG	POS	POS	POS	POS	NEG	NEG	POS	POS	POS	NEG	NEG	NEG
2012_222_Z3479 (1489/12)	POS	POS	AMB	AMB	NEG	NEG	NEG	POS	POS	NEG	AMB	POS	POS	NEG	POS	POS	POS	NEG	POS	NEG	NEG	POS	POS	POS	POS	NEG	NEG	POS	POS	POS	NEG	NEG	NEG
2012_233_Z3540 (1943/12)	POS	POS	AMB	AMB	NEG	NEG	NEG	POS	POS	NEG	AMB	POS	POS	NEG	POS	POS	POS	NEG	POS	NEG	NEG	POS	POS	POS	POS	NEG	NEG	POS	POS	POS	NEG	NEG	NEG
2012_237_Z3541	POS	AMB	AMB	POS	NEG	NEG	NEG	POS	POS	NEG	AMB	POS	POS	NEG	POS	POS	POS	NEG	POS	NEG	NEG	POS	POS	POS	POS	NEG	NEG	POS	POS	POS	NEG	NEG	NEG
2012_262_Z3549	POS	AMB	AMB	POS	NEG	NEG	NEG	POS	POS	NEG	POS	POS	POS	NEG	POS	POS	POS	AMB	POS	NEG	AMB	POS	POS	POS	POS	NEG	NEG	POS	POS	POS	NEG	NEG	NEG
2012_275_Z3562 (3893/12)	POS	AMB	AMB	POS	NEG	NEG	NEG	POS	POS	NEG	POS	POS	POS	NEG	POS	POS	POS	AMB	POS	NEG	NEG	POS	POS	POS	POS	NEG	NEG	POS	POS	POS	NEG	NEG	NEG
2012_291_Z3578	POS	AMB	AMB	POS	NEG	NEG	NEG	POS	POS	NEG	AMB	POS	POS	NEG	POS	POS	POS	NEG	POS	NEG	NEG	POS	POS	POS	POS	NEG	NEG	POS	POS	POS	NEG	NEG	NEG
2012_314_Z3601 (6939/12)	POS	AMB	AMB	POS	NEG	NEG	NEG	POS	POS	NEG	AMB	POS	POS	NEG	POS	POS	POS	NEG	POS	NEG	NEG	POS	POS	POS	POS	NEG	NEG	POS	POS	POS	NEG	NEG	NEG
2012323_Z3610	POS	AMB	AMB	POS	NEG	NEG	NEG	POS	POS	NEG	POS	POS	POS	NEG	POS	POS	POS	NEG	POS	NEG	AMB	POS	POS	POS	POS	NEG	NEG	POS	POS	POS	NEG	NEG	NEG
2012330_Z3617 (7605/12)	POS	AMB	AMB	POS	NEG	NEG	NEG	POS	POS	NEG	POS	POS	POS	NEG	POS	POS	POS	AMB	POS	NEG	AMB	POS	POS	POS	POS	NEG	NEG	POS	POS	POS	NEG	NEG	NEG
2012332_Z3619 (8076/12)	POS	AMB	AMB	POS	NEG	NEG	NEG	POS	POS	NEG	POS	POS	POS	NEG	POS	POS	POS	NEG	POS	NEG	NEG	POS	POS	POS	POS	NEG	NEG	POS	POS	POS	NEG	NEG	NEG
2012339_Z3626 (8558/12)	POS	AMB	AMB	POS	NEG	NEG	NEG	POS	POS	NEG	AMB	POS	POS	NEG	POS	POS	POS	NEG	POS	NEG	NEG	POS	POS	POS	POS	NEG	NEG	POS	POS	POS	NEG	NEG	NEG
2012340_Z3627 (8523/12)	POS	AMB	AMB	POS	NEG	NEG	NEG	POS	POS	NEG	AMB	POS	POS	NEG	POS	POS	POS	NEG	POS	NEG	NEG	POS	POS	POS	POS	NEG	NEG	POS	POS	POS	NEG	NEG	NEG
2012348_Z3635	POS	POS	AMB	AMB	NEG	NEG	NEG	POS	POS	NEG	POS	POS	POS	NEG	POS	POS	POS	AMB	POS	NEG	NEG	POS	POS	POS	POS	NEG	NEG	POS	POS	POS	NEG	NEG	NEG
2012350_Z3637	POS	POS	AMB	AMB	NEG	NEG	NEG	POS	POS	NEG	POS	POS	POS	NEG	POS	POS	POS	NEG	POS	NEG	NEG	POS	POS	POS	POS	NEG	NEG	POS	POS	POS	NEG	NEG	NEG
2012_370_Z3430 (1112-52177)	POS	AMB	AMB	POS	NEG	NEG	NEG	POS	POS	NEG	POS	POS	POS	NEG	POS	POS	POS	NEG	POS	NEG	AMB	POS	POS	POS	POS	NEG	NEG	POS	POS	POS	NEG	NEG	NEG
2012_378_Z3438 (4139)	POS	AMB	AMB	POS	NEG	NEG	NEG	POS	POS	NEG	AMB	POS	POS	NEG	POS	POS	POS	NEG	POS	NEG	NEG	POS	POS	POS	POS	NEG	NEG	POS	POS	POS	NEG	NEG	NEG
2013_PA21_Z4004	POS	POS	AMB	NEG	NEG	NEG	NEG	POS	POS	NEG	NEG	POS	POS	NEG	POS	POS	POS	NEG	POS	NEG	NEG	POS	POS	POS	POS	NEG	NEG	POS	POS	POS	NEG	NEG	NEG
2013_PA22_Z4005	POS	POS	AMB	AMB	NEG	NEG	NEG	POS	POS	NEG	POS	POS	POS	NEG	POS	POS	POS	NEG	POS	NEG	NEG	POS	POS	POS	POS	NEG	NEG	POS	POS	POS	NEG	NEG	NEG
2013_PA24_Z4007	POS	POS	AMB	NEG	NEG	NEG	NEG	POS	POS	NEG	NEG	POS	POS	NEG	POS	POS	POS	NEG	POS	NEG	NEG	POS	POS	POS	POS	NEG	NEG	POS	POS	POS	NEG	NEG	NEG
2013_PA31_Z4014	POS	POS	AMB	AMB	NEG	NEG	NEG	POS	POS	NEG	AMB	POS	POS	NEG	POS	POS	POS	NEG	POS	NEG	NEG	POS	POS	POS	POS	NEG	NEG	POS	POS	POS	NEG	NEG	NEG
2013_PA35_Z4018	POS	POS	AMB	NEG	NEG	NEG	NEG	POS	POS	NEG	NEG	POS	POS	NEG	POS	POS	POS	NEG	POS	NEG	NEG	POS	POS	POS	POS	NEG	NEG	POS	POS	POS	NEG	NEG	NEG
2013_PA81_Z4064	POS	POS	AMB	AMB	NEG	NEG	NEG	POS	POS	NEG	AMB	POS	POS	NEG	POS	POS	POS	NEG	POS	NEG	NEG	POS	POS	POS	POS	NEG	NEG	POS	POS	POS	NEG	NEG	NEG
**ST239-MRSA-III+ccrA/B4**																																	
2012_254_Z3537 (3124A/12)	POS	POS	AMB	AMB	NEG	NEG	NEG	POS	POS	NEG	AMB	POS	POS	NEG	POS	POS	POS	NEG	POS	NEG	NEG	POS	POS	POS	POS	NEG	NEG	POS	POS	POS	NEG	NEG	NEG
2013_PA25_Z4008	POS	POS	AMB	AMB	NEG	NEG	NEG	POS	POS	NEG	AMB	POS	POS	NEG	POS	POS	POS	NEG	POS	NEG	NEG	POS	POS	POS	POS	NEG	NEG	POS	POS	POS	NEG	NEG	NEG
**ST834-MSSA**																																	
2012_268_Z3555 (3693/12)	POS	NEG	AMB	AMB	NEG	POS	NEG	POS	POS	NEG	POS	POS	NEG	POS	POS	POS	POS	NEG	NEG	POS	POS	POS	POS	POS	NEG	NEG	POS	POS	POS	NEG	NEG	NEG	NEG
**CC12-MSSA**																																	
2012_210_Z3470 (1177/12)	POS	NEG	AMB	NEG	NEG	NEG	POS	POS	POS	NEG	POS	NEG	NEG	NEG	NEG	POS	POS	NEG	POS	NEG	NEG	POS	NEG	NEG	NEG	NEG	NEG	POS	POS	NEG	NEG	NEG	NEG
2012_230_Z3487 (1797/12)	POS	NEG	AMB	NEG	NEG	NEG	POS	POS	POS	NEG	POS	NEG	NEG	NEG	NEG	POS	POS	NEG	POS	NEG	NEG	POS	NEG	NEG	NEG	NEG	NEG	POS	POS	NEG	NEG	NEG	NEG
2013_PA18_Z4001	POS	NEG	AMB	NEG	NEG	NEG	NEG	POS	POS	NEG	POS	NEG	NEG	NEG	NEG	POS	POS	NEG	POS	NEG	NEG	POS	NEG	NEG	NEG	NEG	NEG	POS	POS	NEG	NEG	NEG	NEG
**CC15-MSSA**																																	
2012_160_Z3444 (26386/11)	POS	NEG	AMB	AMB	NEG	POS	NEG	POS	POS	NEG	POS	POS	NEG	POS	POS	POS	POS	NEG	POS	NEG	AMB	POS	POS	POS	NEG	POS	NEG	POS	POS	NEG	NEG	NEG	NEG
2012_167_Z3514 (27010/11)	POS	NEG	AMB	AMB	NEG	POS	NEG	POS	POS	NEG	POS	POS	NEG	POS	POS	POS	POS	NEG	POS	NEG	AMB	POS	POS	POS	NEG	POS	NEG	POS	POS	NEG	NEG	NEG	NEG
2012_184_Z3455 (376/12)	POS	NEG	AMB	AMB	NEG	POS	NEG	POS	POS	NEG	POS	POS	NEG	POS	POS	POS	POS	NEG	POS	NEG	AMB	POS	POS	POS	NEG	POS	NEG	POS	POS	NEG	NEG	NEG	NEG
2012_205_Z3465 (939/12)	POS	NEG	AMB	AMB	NEG	POS	NEG	POS	POS	NEG	POS	POS	NEG	POS	POS	POS	POS	NEG	POS	NEG	NEG	POS	POS	POS	NEG	POS	NEG	POS	POS	NEG	NEG	NEG	NEG
2012_231_Z3519 (1831/12)	POS	NEG	POS	AMB	NEG	AMB	NEG	POS	POS	NEG	POS	POS	NEG	POS	POS	POS	POS	NEG	POS	NEG	AMB	POS	POS	POS	NEG	POS	NEG	POS	POS	NEG	NEG	NEG	NEG
2012_249_Z3533 (2907/12)	POS	NEG	AMB	AMB	NEG	POS	NEG	POS	POS	NEG	POS	POS	NEG	POS	POS	POS	POS	NEG	POS	NEG	NEG	POS	POS	POS	NEG	POS	NEG	POS	POS	NEG	NEG	NEG	NEG
2012_305_Z3592 (5839/12)	POS	NEG	AMB	AMB	NEG	POS	NEG	POS	POS	NEG	POS	POS	NEG	POS	POS	POS	POS	NEG	POS	NEG	NEG	POS	POS	POS	NEG	POS	NEG	POS	POS	NEG	NEG	NEG	NEG
2012_313_Z3600 (6857/12)	POS	NEG	AMB	POS	NEG	AMB	NEG	POS	POS	NEG	POS	POS	NEG	POS	POS	POS	POS	NEG	POS	NEG	NEG	POS	POS	POS	NEG	POS	NEG	POS	POS	NEG	NEG	NEG	NEG
2012_345_Z3632 (79668/12)	POS	NEG	AMB	AMB	NEG	POS	NEG	POS	POS	NEG	POS	POS	NEG	POS	POS	POS	POS	NEG	POS	NEG	NEG	POS	POS	POS	NEG	POS	NEG	POS	POS	NEG	NEG	NEG	NEG
2013_PA33_Z4016	POS	NEG	AMB	POS	NEG	AMB	NEG	POS	AMB	NEG	POS	POS	NEG	POS	POS	POS	POS	NEG	POS	NEG	NEG	POS	POS	POS	NEG	POS	NEG	POS	POS	NEG	NEG	NEG	NEG
**CC20-MSSA**																																	
2012_240_Z3526 (2354/12)	POS	NEG	AMB	AMB	NEG	POS	NEG	POS	AMB	NEG	POS	POS	POS	NEG	POS	POS	POS	AMB	NEG	POS	POS	POS	POS	POS	NEG	NEG	POS	POS	POS	NEG	NEG	POS	NEG
**CC30-MSSA**																																	
2012_155_Z3488 (25905A/11)	NEG	NEG	NEG	NEG	NEG	NEG	NEG	NEG	NEG	NEG	NEG	NEG	NEG	NEG	NEG	POS	POS	NEG	NEG	NEG	POS	NEG	POS	POS	NEG	NEG	POS	POS	POS	NEG	POS	NEG	NEG
**CC30-MSSA [PVL+]**																																	
2012_164_Z3447 (26574/11)	POS	NEG	AMB	POS	NEG	AMB	NEG	POS	AMB	POS	AMB	NEG	NEG	NEG	NEG	POS	POS	NEG	NEG	NEG	POS	NEG	POS	POS	NEG	NEG	POS	POS	POS	NEG	POS	NEG	NEG
2012_166_Z3493 (26988A/11)	POS	NEG	AMB	POS	NEG	NEG	NEG	POS	NEG	POS	NEG	NEG	NEG	NEG	NEG	POS	POS	NEG	NEG	NEG	POS	NEG	POS	POS	NEG	NEG	POS	POS	POS	NEG	POS	NEG	NEG
2012_178_Z3500 (197/12)	NEG	NEG	NEG	NEG	NEG	NEG	NEG	POS	NEG	POS	NEG	NEG	NEG	NEG	NEG	POS	POS	NEG	NEG	NEG	NEG	NEG	POS	POS	NEG	NEG	POS	POS	POS	NEG	POS	NEG	NEG
2012_183_Z3454	POS	NEG	AMB	POS	NEG	NEG	NEG	POS	NEG	POS	NEG	NEG	NEG	NEG	NEG	POS	POS	NEG	NEG	NEG	AMB	NEG	POS	POS	NEG	NEG	POS	POS	POS	NEG	POS	NEG	NEG
2012_186_Z3503 (444/12)	POS	NEG	AMB	POS	NEG	NEG	NEG	POS	NEG	POS	NEG	NEG	NEG	NEG	NEG	POS	POS	NEG	NEG	NEG	AMB	NEG	POS	POS	NEG	NEG	POS	POS	POS	NEG	POS	NEG	NEG
2012_189_Z3506 (542/12)	POS	NEG	AMB	POS	NEG	NEG	NEG	POS	NEG	POS	NEG	NEG	NEG	NEG	NEG	POS	POS	NEG	NEG	NEG	POS	NEG	POS	POS	NEG	NEG	POS	POS	POS	NEG	POS	NEG	NEG
2012_192_Z3457 (606/12)	POS	NEG	AMB	POS	NEG	NEG	NEG	POS	NEG	POS	NEG	NEG	NEG	NEG	NEG	POS	POS	NEG	NEG	NEG	AMB	NEG	POS	POS	NEG	NEG	POS	POS	POS	NEG	POS	NEG	NEG
2012_196_Z3509 (707/12)	POS	NEG	AMB	POS	NEG	NEG	NEG	POS	NEG	POS	NEG	NEG	NEG	NEG	NEG	POS	POS	NEG	NEG	NEG	POS	NEG	POS	POS	NEG	NEG	POS	POS	POS	NEG	POS	NEG	NEG
2012_218_Z3476 (1432/12)	POS	NEG	AMB	POS	NEG	NEG	NEG	POS	NEG	POS	NEG	NEG	NEG	NEG	NEG	POS	POS	NEG	NEG	NEG	POS	NEG	POS	POS	NEG	NEG	POS	POS	POS	NEG	POS	NEG	NEG
2012_232_Z3520 (1892/12)	POS	NEG	AMB	POS	NEG	NEG	NEG	POS	NEG	POS	NEG	NEG	NEG	NEG	NEG	POS	POS	NEG	NEG	NEG	POS	NEG	POS	POS	NEG	NEG	POS	POS	POS	NEG	POS	NEG	NEG
2012_278_Z3565 (4397/12)	POS	NEG	AMB	POS	NEG	NEG	NEG	POS	NEG	POS	NEG	NEG	NEG	NEG	NEG	POS	POS	NEG	NEG	NEG	POS	NEG	POS	POS	NEG	NEG	POS	POS	POS	NEG	POS	NEG	NEG
2012_285_Z3572 (4802/12)	POS	NEG	AMB	POS	NEG	NEG	NEG	POS	NEG	POS	NEG	NEG	NEG	NEG	NEG	POS	POS	NEG	NEG	NEG	POS	NEG	POS	POS	NEG	NEG	POS	POS	POS	NEG	POS	NEG	NEG
2012_287_Z3574 (4906/12)	POS	NEG	AMB	POS	NEG	NEG	NEG	POS	NEG	POS	NEG	NEG	NEG	NEG	NEG	POS	POS	NEG	NEG	NEG	POS	NEG	POS	POS	NEG	NEG	POS	POS	POS	NEG	POS	NEG	NEG
2012_295_Z3582 (5116/12)	POS	NEG	AMB	POS	NEG	NEG	NEG	POS	NEG	POS	NEG	NEG	NEG	NEG	NEG	POS	POS	NEG	NEG	NEG	POS	NEG	POS	POS	NEG	NEG	POS	POS	POS	NEG	POS	NEG	NEG
2012_326_Z3613 (7321/12)	POS	NEG	AMB	POS	NEG	NEG	NEG	POS	NEG	POS	AMB	NEG	NEG	NEG	NEG	POS	POS	NEG	NEG	NEG	POS	NEG	POS	POS	NEG	NEG	POS	POS	POS	NEG	POS	NEG	NEG
2012_356_Z3643 (1204-15897)	POS	NEG	AMB	POS	NEG	NEG	NEG	POS	NEG	POS	NEG	NEG	NEG	NEG	NEG	POS	POS	NEG	NEG	NEG	POS	NEG	POS	POS	NEG	NEG	POS	POS	POS	NEG	POS	NEG	NEG
2012_366_Z3426 (1204-16124)	POS	NEG	AMB	POS	NEG	NEG	NEG	POS	AMB	POS	AMB	NEG	NEG	NEG	NEG	POS	POS	NEG	NEG	NEG	POS	NEG	POS	POS	NEG	NEG	POS	POS	POS	NEG	POS	NEG	NEG
2012_372_Z3432 (1205-19821)	POS	NEG	AMB	POS	NEG	NEG	NEG	POS	NEG	POS	NEG	NEG	NEG	NEG	NEG	POS	POS	NEG	NEG	NEG	AMB	NEG	POS	POS	NEG	NEG	POS	POS	POS	NEG	POS	NEG	NEG
2012_380_Z3440 (4141)	POS	NEG	AMB	POS	NEG	NEG	NEG	POS	NEG	POS	NEG	NEG	NEG	NEG	NEG	POS	POS	NEG	NEG	NEG	POS	NEG	POS	POS	NEG	NEG	POS	POS	POS	NEG	POS	NEG	NEG
2013_PA20_Z4003	POS	NEG	AMB	POS	NEG	NEG	NEG	POS	NEG	POS	NEG	NEG	NEG	NEG	NEG	POS	POS	NEG	NEG	NEG	NEG	NEG	POS	POS	NEG	NEG	POS	POS	POS	NEG	POS	NEG	NEG
2013_PA36_Z4019	POS	NEG	AMB	POS	NEG	NEG	NEG	POS	NEG	POS	NEG	NEG	NEG	NEG	NEG	POS	POS	NEG	NEG	NEG	NEG	NEG	NEG	AMB	NEG	NEG	AMB	POS	POS	NEG	POS	NEG	NEG
2013_PA46_Z4029	POS	NEG	AMB	POS	NEG	NEG	NEG	POS	NEG	POS	NEG	NEG	NEG	NEG	NEG	POS	POS	NEG	NEG	NEG	NEG	NEG	POS	POS	NEG	NEG	POS	POS	POS	NEG	POS	NEG	NEG
2013_PA50_Z4033	POS	NEG	AMB	POS	NEG	NEG	NEG	POS	NEG	POS	NEG	NEG	NEG	NEG	NEG	POS	POS	NEG	NEG	NEG	NEG	NEG	POS	POS	NEG	NEG	POS	POS	POS	NEG	POS	NEG	NEG
2013_PA63_Z4046	NEG	NEG	NEG	NEG	NEG	NEG	NEG	POS	NEG	POS	NEG	NEG	NEG	NEG	NEG	POS	POS	NEG	NEG	NEG	AMB	NEG	POS	POS	NEG	NEG	POS	POS	POS	NEG	POS	NEG	NEG
2013_PA78_Z4061	POS	NEG	AMB	POS	NEG	NEG	NEG	POS	NEG	POS	NEG	NEG	NEG	NEG	NEG	POS	POS	NEG	NEG	NEG	AMB	NEG	POS	POS	NEG	NEG	POS	POS	POS	NEG	POS	NEG	NEG
2013_PA79_Z4062	POS	NEG	AMB	POS	NEG	NEG	NEG	POS	NEG	POS	NEG	NEG	NEG	NEG	NEG	POS	POS	NEG	NEG	NEG	POS	NEG	POS	POS	NEG	NEG	POS	POS	POS	NEG	POS	NEG	NEG
**CC45-MSSA**																																	
2012_172_Z3449 (49/12)	POS	NEG	AMB	AMB	NEG	POS	NEG	POS	AMB	POS	AMB	NEG	NEG	NEG	NEG	POS	POS	POS	AMB	NEG	POS	NEG	POS	POS	POS	NEG	NEG	POS	POS	NEG	POS	NEG	NEG
2012_208_Z3468 (1011/12)	POS	NEG	AMB	POS	NEG	AMB	NEG	POS	NEG	POS	NEG	NEG	NEG	NEG	NEG	POS	POS	POS	NEG	NEG	NEG	NEG	POS	POS	POS	NEG	NEG	POS	AMB	NEG	POS	NEG	NEG
2012_235_Z3522 (2085/12)	POS	NEG	POS	AMB	NEG	AMB	NEG	POS	NEG	POS	NEG	NEG	NEG	NEG	NEG	POS	POS	POS	NEG	NEG	POS	NEG	POS	POS	POS	NEG	NEG	POS	POS	NEG	POS	NEG	NEG
2012_290_Z3577 (4921/12)	POS	NEG	POS	AMB	NEG	AMB	NEG	POS	NEG	POS	NEG	NEG	NEG	NEG	NEG	POS	POS	POS	NEG	NEG	AMB	NEG	POS	POS	POS	NEG	NEG	POS	AMB	NEG	POS	NEG	NEG
2012_311_Z3598 (6182/12)	POS	NEG	AMB	POS	NEG	AMB	NEG	POS	NEG	POS	NEG	NEG	NEG	NEG	NEG	POS	POS	POS	NEG	NEG	AMB	NEG	NEG	NEG	NEG	NEG	NEG	POS	AMB	NEG	POS	NEG	NEG
Z3604_TT317 - (DC558C18-2959-4049-B8F3-507365	NEG	NEG	NEG	NEG	NEG	NEG	NEG	POS	NEG	POS	NEG	NEG	NEG	NEG	NEG	POS	POS	POS	NEG	NEG	NEG	NEG	POS	POS	POS	NEG	NEG	POS	AMB	NEG	POS	NEG	NEG
2012_363_Z3423 (1112-61058)	POS	NEG	AMB	POS	NEG	AMB	NEG	POS	AMB	POS	AMB	NEG	NEG	NEG	NEG	POS	POS	POS	NEG	NEG	AMB	NEG	POS	POS	POS	NEG	NEG	POS	POS	NEG	POS	NEG	NEG
2013_PA19_Z4002	POS	NEG	AMB	POS	NEG	AMB	NEG	POS	NEG	POS	NEG	NEG	NEG	NEG	NEG	POS	POS	POS	NEG	NEG	AMB	NEG	POS	POS	POS	NEG	NEG	POS	AMB	NEG	POS	NEG	NEG
2013_PA54_Z4037	POS	NEG	AMB	POS	NEG	AMB	NEG	POS	AMB	POS	AMB	NEG	NEG	NEG	NEG	POS	POS	POS	AMB	NEG	POS	NEG	POS	POS	POS	NEG	NEG	POS	POS	NEG	POS	NEG	NEG
2013_PA82_Z4065	POS	NEG	AMB	POS	NEG	AMB	NEG	NEG	NEG	#ZAHL!	NEG	NEG	NEG	NEG	NEG	POS	POS	POS	NEG	NEG	NEG	NEG	NEG	NEG	NEG	NEG	NEG	POS	AMB	NEG	POS	NEG	NEG
**CC97-MSSA**																																	
2012_182_Z3453 (279A/12)	POS	NEG	AMB	POS	NEG	NEG	NEG	POS	POS	AMB	POS	POS	POS	NEG	POS	POS	POS	AMB	NEG	POS	POS	POS	POS	POS	NEG	POS	NEG	POS	POS	NEG	NEG	NEG	POS
2012_187_Z3504	POS	NEG	AMB	POS	NEG	NEG	NEG	POS	POS	NEG	POS	POS	POS	NEG	POS	POS	POS	NEG	NEG	POS	POS	POS	POS	POS	NEG	POS	NEG	POS	POS	NEG	NEG	NEG	POS
2012_197_Z3510 (757/12)	POS	NEG	AMB	POS	NEG	NEG	NEG	POS	POS	NEG	POS	POS	POS	NEG	POS	POS	POS	NEG	NEG	POS	POS	POS	POS	POS	NEG	POS	NEG	POS	POS	NEG	NEG	NEG	POS
2012_199_Z3516 (785/12)	POS	NEG	AMB	POS	NEG	NEG	NEG	POS	POS	AMB	POS	POS	POS	NEG	POS	POS	POS	AMB	NEG	POS	POS	POS	POS	POS	NEG	POS	NEG	POS	POS	NEG	NEG	NEG	POS
2013_PA15_Z3998	POS	NEG	AMB	POS	NEG	NEG	NEG	POS	POS	NEG	POS	POS	POS	NEG	POS	POS	POS	NEG	NEG	POS	POS	POS	POS	POS	NEG	POS	NEG	POS	POS	NEG	NEG	NEG	POS
**CC101-MSSA**																																	
2012_283_Z3570 (4725/12)	POS	NEG	POS	AMB	NEG	NEG	NEG	POS	AMB	NEG	POS	NEG	NEG	NEG	NEG	POS	POS	NEG	NEG	POS	NEG	POS	POS	POS	POS	NEG	NEG	POS	POS	NEG	NEG	NEG	NEG
2012_247_Z3531 (2788/12)	POS	NEG	POS	AMB	NEG	NEG	NEG	POS	NEG	NEG	POS	NEG	NEG	NEG	NEG	POS	POS	NEG	NEG	POS	NEG	POS	POS	POS	POS	NEG	NEG	POS	POS	NEG	NEG	NEG	NEG
2012_279_Z3566 (4506/12)	POS	NEG	POS	AMB	NEG	NEG	NEG	POS	POS	NEG	POS	NEG	NEG	NEG	NEG	POS	POS	AMB	NEG	POS	AMB	POS	POS	POS	POS	NEG	NEG	POS	POS	NEG	NEG	NEG	NEG

Strain / Isolates	fibronectin-binding protein B							Major histocompatibility complex class II analog protein (=Extracellular adherence protein, eap)				Staphylococcus aureus surface protein G				Ser-Asp rich fibrinogen-/bone sialoprotein-binding protein C							Ser-Asp rich fibrinogen-/bone sialoprotein-binding protein D					van Willebrand factor binding protein					
	fnbB (total)	fnbB (COL)	fnbB (COL+Mu50+MW2)	fnbB (Mu50)	fnbB (MW2)	fnbB (ST15)	fnbB (ST45-2)	map (total)	map (COL)	map (MRSA252)	map (Mu50+MW2)	sasG (total)	sasG (COL+Mu50)	sasG (MW2)	sasG (other than MRSA252+RF122)	sdrC (total)	sdrC (consensus)	sdrC (B1)	sdrC (COL)	sdrC (Mu50)	sdrC (MW2+MRSA252+RF122)	sdrC (other than MRSA252+RF122)	sdrD (total)	sdrD (consensus)	sdrD (COL+MW2)	sdrD (Mu50)	sdrD (other)	vwb (total)	vwb (consensus)	vwb (COL+MW2)	vwb (MRSA252)	vwb (Mu50)	vwb (RF122)
**CC121-MSSA**																																	
2012_163_Z3446 (26495/11)	NEG	NEG	NEG	NEG	NEG	NEG	NEG	POS	POS	NEG	POS	NEG	NEG	AMB	NEG	POS	POS	NEG	POS	NEG	AMB	NEG	POS	POS	NEG	POS	NEG	POS	POS	POS	NEG	NEG	NEG
2012_204_Z3464 (938/12)	POS	NEG	NEG	POS	NEG	NEG	NEG	POS	POS	NEG	POS	NEG	NEG	AMB	NEG	POS	POS	NEG	POS	NEG	NEG	NEG	POS	POS	NEG	POS	NEG	POS	POS	POS	NEG	NEG	NEG
2012_225_Z3482 (1563/12)	NEG	NEG	NEG	NEG	NEG	NEG	NEG	POS	POS	NEG	POS	NEG	NEG	AMB	NEG	POS	POS	NEG	POS	NEG	AMB	NEG	POS	POS	NEG	POS	NEG	POS	POS	POS	NEG	NEG	NEG
2012_228_Z3485 (1700B/12)	NEG	NEG	NEG	NEG	NEG	NEG	NEG	POS	POS	NEG	POS	NEG	NEG	AMB	NEG	POS	POS	NEG	POS	NEG	AMB	NEG	POS	POS	NEG	POS	NEG	POS	POS	POS	NEG	NEG	NEG
2012_229_Z3486 (1737/12)	NEG	NEG	NEG	NEG	NEG	NEG	NEG	POS	POS	NEG	POS	NEG	NEG	AMB	NEG	POS	POS	NEG	POS	NEG	NEG	NEG	POS	POS	NEG	POS	NEG	POS	POS	POS	NEG	NEG	NEG
2012_302_Z3589 (5795/12)	NEG	NEG	NEG	NEG	NEG	NEG	NEG	POS	POS	NEG	POS	NEG	NEG	NEG	NEG	POS	POS	NEG	POS	NEG	NEG	NEG	POS	POS	NEG	POS	NEG	POS	POS	POS	NEG	NEG	NEG
2012_359_Z3419	NEG	NEG	NEG	NEG	NEG	NEG	NEG	POS	POS	NEG	POS	NEG	NEG	AMB	NEG	POS	POS	NEG	POS	NEG	NEG	NEG	POS	POS	NEG	AMB	NEG	POS	POS	POS	NEG	NEG	NEG
2012_362_Z3422 (1201-996)	POS	NEG	NEG	POS	NEG	NEG	NEG	POS	POS	NEG	POS	NEG	NEG	NEG	NEG	POS	POS	NEG	POS	NEG	AMB	NEG	POS	POS	NEG	POS	NEG	POS	POS	POS	NEG	NEG	NEG
2012_367_Z3427 (1204-16413)	NEG	NEG	NEG	NEG	NEG	NEG	NEG	POS	POS	NEG	POS	NEG	NEG	NEG	NEG	POS	POS	NEG	POS	NEG	AMB	NEG	POS	POS	NEG	POS	NEG	POS	POS	POS	NEG	NEG	NEG
2013_PA09_Z3992	NEG	NEG	NEG	NEG	NEG	NEG	NEG	POS	POS	NEG	POS	NEG	NEG	NEG	NEG	POS	POS	NEG	POS	NEG	NEG	NEG	POS	POS	NEG	AMB	NEG	POS	POS	POS	NEG	NEG	NEG
2013_PA59_Z4042	NEG	NEG	NEG	NEG	NEG	NEG	NEG	POS	POS	NEG	POS	NEG	NEG	NEG	NEG	POS	POS	NEG	POS	NEG	NEG	NEG	POS	POS	NEG	AMB	NEG	POS	POS	POS	NEG	NEG	NEG
2013_PA60_Z4043	NEG	NEG	NEG	NEG	NEG	NEG	NEG	POS	POS	NEG	NEG	NEG	NEG	NEG	NEG	POS	POS	NEG	POS	NEG	NEG	NEG	POS	POS	NEG	NEG	NEG	POS	POS	POS	NEG	NEG	NEG
**CC152-MSSA [PVL+]**																																	
2012_190_Z3507 (549/12)	POS	NEG	AMB	POS	NEG	NEG	NEG	NEG	NEG	NEG	NEG	NEG	NEG	NEG	NEG	NEG	AMB	NEG	NEG	NEG	NEG	NEG	POS	POS	POS	NEG	NEG	POS	POS	NEG	NEG	NEG	NEG
2012_201_Z3461 (823/12)	POS	NEG	POS	AMB	NEG	NEG	NEG	NEG	NEG	NEG	NEG	NEG	NEG	AMB	NEG	NEG	NEG	NEG	NEG	NEG	NEG	NEG	POS	POS	POS	NEG	NEG	POS	POS	NEG	NEG	NEG	NEG
2012_209_Z3469 (1116/12)	POS	NEG	POS	AMB	NEG	NEG	NEG	NEG	NEG	NEG	NEG	NEG	NEG	NEG	NEG	NEG	NEG	NEG	NEG	NEG	NEG	NEG	POS	POS	POS	NEG	NEG	POS	POS	NEG	NEG	NEG	NEG
2012_211_Z3517 (1238/12)	POS	NEG	AMB	POS	NEG	NEG	NEG	NEG	NEG	NEG	NEG	NEG	NEG	AMB	NEG	NEG	NEG	NEG	NEG	NEG	NEG	NEG	POS	POS	POS	NEG	NEG	POS	POS	NEG	NEG	NEG	NEG
2012_213_Z3472 (1320/12)	POS	NEG	POS	AMB	NEG	NEG	NEG	NEG	NEG	NEG	NEG	NEG	NEG	NEG	NEG	NEG	NEG	NEG	NEG	NEG	NEG	NEG	POS	POS	POS	NEG	NEG	POS	POS	NEG	NEG	NEG	NEG
2012_221_Z3478	POS	NEG	POS	AMB	NEG	NEG	NEG	NEG	NEG	NEG	NEG	NEG	NEG	NEG	NEG	NEG	NEG	NEG	NEG	NEG	NEG	NEG	POS	POS	POS	NEG	NEG	POS	POS	NEG	NEG	NEG	NEG
2012_234_Z3521 (1981/12)	POS	NEG	AMB	POS	NEG	NEG	NEG	NEG	NEG	NEG	NEG	NEG	NEG	AMB	NEG	NEG	NEG	NEG	NEG	NEG	NEG	NEG	POS	POS	POS	NEG	NEG	POS	POS	NEG	NEG	NEG	NEG
2012_238_Z3524 (2199/12)	POS	NEG	AMB	POS	NEG	NEG	NEG	NEG	NEG	NEG	NEG	NEG	NEG	NEG	NEG	NEG	NEG	NEG	NEG	NEG	NEG	NEG	POS	POS	POS	NEG	NEG	POS	POS	NEG	NEG	NEG	NEG
2012_260_Z3547 (3290/12)	POS	NEG	AMB	POS	NEG	NEG	NEG	NEG	NEG	NEG	NEG	NEG	NEG	NEG	NEG	NEG	NEG	NEG	NEG	NEG	NEG	NEG	POS	POS	POS	NEG	NEG	POS	POS	NEG	NEG	NEG	NEG
2012_272_Z3559 (3802/12)	POS	NEG	AMB	POS	NEG	NEG	NEG	NEG	NEG	NEG	NEG	NEG	NEG	AMB	NEG	NEG	NEG	NEG	NEG	NEG	NEG	NEG	POS	POS	POS	NEG	NEG	POS	POS	NEG	NEG	NEG	NEG
2012_289_Z3576 (4912/12)	POS	NEG	AMB	POS	NEG	NEG	NEG	NEG	NEG	NEG	NEG	NEG	NEG	NEG	NEG	NEG	NEG	NEG	NEG	NEG	NEG	NEG	POS	POS	POS	NEG	NEG	POS	POS	NEG	NEG	NEG	NEG
2012_309_Z3596	POS	NEG	AMB	POS	NEG	NEG	NEG	NEG	NEG	NEG	NEG	NEG	NEG	AMB	NEG	NEG	NEG	NEG	NEG	NEG	NEG	NEG	POS	POS	POS	NEG	NEG	POS	POS	NEG	NEG	NEG	NEG
2012_322_Z3609 (7178/12)	POS	NEG	POS	AMB	NEG	NEG	NEG	NEG	NEG	NEG	NEG	NEG	NEG	NEG	NEG	NEG	NEG	NEG	NEG	NEG	NEG	NEG	POS	POS	POS	NEG	NEG	POS	POS	NEG	NEG	NEG	NEG
2012_325_Z3612 (7254/12)	POS	NEG	AMB	POS	NEG	NEG	NEG	NEG	NEG	NEG	NEG	NEG	NEG	AMB	NEG	NEG	NEG	NEG	NEG	NEG	NEG	NEG	POS	POS	POS	NEG	NEG	POS	POS	NEG	NEG	NEG	NEG
2012_328_Z3615 (7456/12)	POS	NEG	AMB	POS	NEG	NEG	NEG	NEG	NEG	NEG	NEG	NEG	NEG	AMB	NEG	NEG	NEG	NEG	NEG	NEG	NEG	NEG	POS	POS	POS	NEG	NEG	POS	POS	NEG	NEG	NEG	NEG
2012_334_Z3621 (8267/12)	POS	NEG	AMB	POS	NEG	NEG	NEG	NEG	NEG	NEG	NEG	NEG	NEG	AMB	NEG	NEG	NEG	NEG	NEG	NEG	NEG	NEG	POS	POS	POS	NEG	NEG	POS	POS	NEG	NEG	NEG	NEG
2012_360_Z3420 (1201-4047)	POS	NEG	AMB	POS	NEG	NEG	NEG	NEG	NEG	NEG	NEG	NEG	NEG	AMB	NEG	NEG	NEG	NEG	NEG	NEG	NEG	NEG	POS	POS	POS	NEG	NEG	POS	POS	NEG	NEG	NEG	NEG
2012_382_Z3442 (4086)	POS	NEG	AMB	POS	NEG	NEG	NEG	NEG	NEG	NEG	NEG	NEG	NEG	NEG	NEG	NEG	NEG	NEG	NEG	NEG	NEG	NEG	POS	POS	POS	NEG	NEG	POS	POS	NEG	NEG	NEG	NEG
2013_PA02_Z3985	POS	NEG	AMB	POS	NEG	NEG	NEG	NEG	NEG	NEG	NEG	NEG	NEG	NEG	NEG	NEG	NEG	NEG	NEG	NEG	NEG	NEG	POS	POS	POS	NEG	NEG	POS	POS	NEG	NEG	NEG	NEG
2013_PA03_Z3986	POS	NEG	AMB	POS	NEG	NEG	NEG	NEG	NEG	NEG	NEG	NEG	NEG	NEG	NEG	NEG	NEG	NEG	NEG	NEG	NEG	NEG	POS	POS	POS	NEG	NEG	POS	POS	NEG	NEG	NEG	NEG
2013_PA07_Z3990	POS	NEG	AMB	POS	NEG	NEG	NEG	NEG	NEG	NEG	NEG	NEG	NEG	NEG	NEG	NEG	NEG	NEG	NEG	NEG	NEG	NEG	POS	POS	POS	NEG	NEG	POS	POS	NEG	NEG	NEG	NEG
2013_PA34_Z4017	POS	NEG	POS	AMB	NEG	NEG	NEG	NEG	NEG	NEG	NEG	NEG	NEG	NEG	NEG	NEG	NEG	NEG	NEG	NEG	NEG	NEG	POS	AMB	POS	NEG	NEG	POS	POS	NEG	NEG	NEG	NEG
2013_PA40_Z4023	POS	NEG	POS	AMB	NEG	NEG	NEG	NEG	NEG	NEG	NEG	NEG	NEG	NEG	NEG	NEG	NEG	NEG	NEG	NEG	NEG	NEG	POS	AMB	POS	NEG	NEG	POS	POS	NEG	NEG	NEG	NEG
2013_PA48_Z4031	POS	NEG	POS	AMB	NEG	NEG	NEG	NEG	NEG	NEG	NEG	NEG	NEG	NEG	NEG	NEG	NEG	NEG	NEG	NEG	NEG	NEG	POS	POS	POS	NEG	NEG	POS	POS	NEG	NEG	NEG	NEG
2013_PA49_Z4032	POS	NEG	POS	AMB	NEG	NEG	NEG	NEG	NEG	NEG	NEG	NEG	NEG	NEG	NEG	NEG	NEG	NEG	NEG	NEG	NEG	NEG	POS	AMB	POS	NEG	NEG	POS	POS	NEG	NEG	NEG	NEG
2013_PA51_Z4034	POS	NEG	POS	AMB	NEG	NEG	NEG	NEG	NEG	NEG	NEG	NEG	NEG	NEG	NEG	NEG	NEG	NEG	NEG	NEG	NEG	NEG	POS	POS	POS	NEG	NEG	POS	POS	NEG	NEG	NEG	NEG
2013_PA70_Z4053	POS	NEG	POS	AMB	NEG	NEG	NEG	NEG	NEG	NEG	NEG	NEG	NEG	NEG	NEG	NEG	NEG	NEG	NEG	NEG	NEG	NEG	POS	POS	POS	NEG	NEG	POS	POS	NEG	NEG	NEG	NEG
2013_PA84_Z4067	POS	NEG	AMB	POS	NEG	NEG	NEG	NEG	NEG	NEG	NEG	NEG	NEG	NEG	NEG	NEG	NEG	NEG	NEG	NEG	NEG	NEG	POS	POS	POS	NEG	NEG	POS	POS	NEG	NEG	NEG	NEG
**CC188-MSSA**																																	
2012_206_Z3466 (968/12)	POS	NEG	POS	AMB	NEG	AMB	NEG	POS	POS	NEG	POS	NEG	NEG	NEG	NEG	POS	POS	NEG	POS	NEG	AMB	NEG	POS	POS	NEG	POS	NEG	POS	POS	NEG	POS	NEG	NEG
2012_251_Z3534 (3026/12)	POS	NEG	AMB	POS	NEG	AMB	NEG	POS	POS	NEG	POS	NEG	NEG	NEG	NEG	POS	POS	NEG	POS	NEG	NEG	NEG	POS	POS	NEG	POS	NEG	POS	POS	NEG	POS	NEG	NEG
2012_320_Z3607 (7144/12)	POS	NEG	AMB	POS	NEG	AMB	NEG	POS	POS	NEG	POS	NEG	NEG	NEG	NEG	POS	POS	NEG	POS	NEG	NEG	NEG	POS	POS	NEG	POS	NEG	POS	POS	NEG	POS	NEG	NEG
2012_321_Z3608 (7147/12)	POS	NEG	AMB	POS	NEG	AMB	NEG	POS	POS	NEG	POS	NEG	NEG	NEG	NEG	POS	POS	NEG	POS	NEG	NEG	NEG	POS	POS	NEG	POS	NEG	POS	POS	NEG	POS	NEG	NEG
2012_358_Z3418 (1202-6332)	POS	NEG	POS	AMB	NEG	AMB	NEG	POS	POS	NEG	POS	NEG	NEG	NEG	NEG	POS	POS	NEG	POS	NEG	AMB	NEG	POS	POS	NEG	POS	NEG	POS	POS	NEG	POS	NEG	NEG
**CC398-MSSA**																																	
2013_PA11_Z3994	POS	NEG	POS	AMB	NEG	NEG	NEG	POS	POS	POS	NEG	NEG	NEG	NEG	NEG	POS	POS	NEG	NEG	POS	POS	NEG	POS	POS	NEG	AMB	NEG	POS	POS	NEG	NEG	NEG	NEG
2013_PA57_Z4040	POS	NEG	POS	NEG	NEG	NEG	NEG	POS	POS	POS	NEG	NEG	NEG	NEG	NEG	POS	POS	NEG	NEG	POS	POS	NEG	POS	POS	NEG	NEG	NEG	POS	POS	NEG	NEG	NEG	NEG
**ST1852-MSSA**																																	
2012_277_Z3564 (4050/12)	POS	NEG	AMB	POS	NEG	NEG	NEG	POS	NEG	NEG	POS	NEG	NEG	NEG	NEG	POS	POS	AMB	NEG	NEG	POS	POS	POS	POS	NEG	NEG	POS	POS	POS	NEG	NEG	NEG	NEG
**CC1223/1594-MSSA**																																	
2012_337_Z3624 (8070B/12)	POS	NEG	POS	AMB	NEG	AMB	NEG	NEG	NEG	NEG	NEG	NEG	NEG	NEG	NEG	POS	POS	NEG	NEG	POS	AMB	NEG	POS	POS	NEG	NEG	POS	POS	POS	NEG	NEG	NEG	NEG
**MSSA related to ST2250/2277**																																	
2012_203_Z3463 (829/12)	POS	NEG	AMB	POS	NEG	AMB	NEG	NEG	NEG	NEG	NEG	POS	NEG	POS	NEG	POS	POS	NEG	NEG	NEG	NEG	NEG	POS	POS	NEG	NEG	POS	POS	POS	AMB	NEG	NEG	NEG
2012_269_Z3556 (3769A/12)	POS	NEG	NEG	POS	NEG	AMB	NEG	NEG	NEG	NEG	NEG	POS	NEG	POS	NEG	POS	POS	NEG	NEG	NEG	NEG	NEG	POS	POS	NEG	NEG	POS	POS	POS	NEG	NEG	NEG	NEG
2012_292_Z3579	POS	NEG	POS	AMB	NEG	AMB	NEG	NEG	NEG	NEG	NEG	POS	NEG	POS	NEG	POS	POS	NEG	NEG	NEG	NEG	NEG	POS	POS	NEG	NEG	POS	POS	POS	NEG	NEG	NEG	NEG
2012_299_Z3586 (5487/12)	POS	NEG	NEG	POS	NEG	NEG	NEG	NEG	NEG	NEG	NEG	POS	NEG	POS	NEG	POS	POS	NEG	NEG	NEG	NEG	NEG	NEG	NEG	NEG	NEG	NEG	NEG	NEG	NEG	NEG	NEG	NEG
2012_301_Z3588 (5717/12)	POS	NEG	NEG	POS	NEG	NEG	NEG	NEG	NEG	NEG	NEG	POS	NEG	POS	NEG	POS	POS	NEG	NEG	NEG	NEG	NEG	POS	POS	NEG	NEG	POS	NEG	NEG	NEG	NEG	NEG	NEG
**ST2596dlv-MSSA**																																	
2012_310_Z3597 (6034/12)	POS	NEG	POS	AMB	NEG	NEG	NEG	NEG	NEG	NEG	NEG	NEG	NEG	NEG	NEG	POS	POS	NEG	NEG	NEG	AMB	NEG	POS	POS	NEG	NEG	POS	POS	POS	NEG	NEG	NEG	NEG

Strain / Isolates	immunodominant antigen B		defensin resistance protein		transferrin-binding protein			hypothetical protein, similar to integral membrane protein LmrP				type I site-specific deoxyribo-nuclease subunit, 1st locus	type I site-specific deoxyribonuclease subunit, 2nd locus				type I site-specific deoxyribonuclease subunit, 3rd locus					type I site-specific deoxyribonuclease subunit, unknown locus			hypothetical protein, located next to serine protease operon	Unspecific efflux/trans-porter	hypothetical protein	hyaluronate lyase, first / second locus			hyaluronate lyase, second locus				
	isaB	isaB (MRSA252)	mprF (COL+MW2)	mprF (Mu50+MRSA252)	isdA (consensus)	isdA (MRSA252)	isdA (other than MRSA252)	lmrP (other than RF122)_probe1	lmrP (other than RF122)_probe2	lmrP (RF122)_probe1	lmrP (RF122)_probe2	hsdS1 (RF122)	hsdS2 (Mu50+N315+COL+USA300+NCTC8325)	hsdS2 (MW2+MSSA476)	hsdS2 (RF122)	hsdS2 (MRSA252)	hsdS3 (all other than RF122+MRSA252)	hsdS3 (COL+USA300+NCTC8325+MW2+MSSA476+RF122)	hsdS3 (Mu50+N315)	hsdS3 (CC151+MRSA252)	hsdS3 (MRSA252)	hsdSx (CC25)	hsdSx (CC15)	hsdSx (etd)	Q2FXC0	Q2YUB3	Q7A4X2	hysA1 (MRSA252)	hysA1 (MRSA252+RF122) and/or hysA2 (consensus)	hysA1 (MRSA252+RF122) and/or hysA2 (COL+USA300)	hysA2 (all other than MRSA252)	hysA2 (COL+USA300+NCTC8325)	hysA2 (all other than COL+USA300+NCTC8325)	hysA2 (all other than COL+USA300+NCTC8325)	hysA2 (MRSA252)
**CC1-MSSA**																																			
2012_257_Z3544 (3216/12)	POS	AMB	POS	AMB	POS	AMB	POS	POS	POS	NEG	NEG	NEG	NEG	POS	NEG	NEG	POS	POS	NEG	NEG	NEG	POS	NEG	NEG	POS	POS	NEG	NEG	POS	NEG	POS	NEG	POS	POS	NEG
2012_281_Z3568 (4696/12)	POS	AMB	AMB	POS	POS	NEG	POS	POS	POS	NEG	NEG	NEG	NEG	POS	NEG	NEG	POS	POS	NEG	NEG	NEG	POS	NEG	NEG	POS	NEG	NEG	NEG	POS	NEG	POS	NEG	POS	POS	NEG
2012_324_Z3611 (7180/12)	POS	AMB	POS	AMB	POS	AMB	POS	POS	POS	NEG	NEG	NEG	NEG	POS	NEG	NEG	POS	POS	NEG	NEG	NEG	POS	AMB	NEG	POS	AMB	NEG	NEG	POS	NEG	POS	NEG	POS	POS	NEG
2012_327_Z3614 (7347/12)	POS	AMB	AMB	POS	POS	AMB	POS	POS	POS	NEG	NEG	NEG	NEG	POS	NEG	NEG	POS	POS	NEG	NEG	NEG	POS	NEG	NEG	POS	AMB	NEG	NEG	POS	NEG	POS	NEG	POS	POS	NEG
2012_351_Z3638 (82738/12)	POS	AMB	AMB	POS	POS	AMB	POS	POS	POS	NEG	NEG	NEG	NEG	POS	NEG	NEG	POS	POS	NEG	NEG	NEG	POS	AMB	NEG	POS	AMB	NEG	NEG	POS	NEG	POS	NEG	POS	POS	NEG
**CC5-MSSA**																																			
2012_188_Z3505 (503/12)	POS	AMB	POS	AMB	POS	AMB	POS	POS	POS	NEG	NEG	NEG	POS	NEG	NEG	NEG	POS	NEG	POS	NEG	NEG	POS	NEG	NEG	NEG	AMB	POS	NEG	POS	NEG	POS	NEG	POS	POS	NEG
2012_219_Z3518 (1437/12)	POS	AMB	POS	AMB	POS	AMB	POS	POS	POS	NEG	NEG	NEG	POS	NEG	NEG	NEG	POS	NEG	POS	NEG	NEG	POS	AMB	NEG	NEG	AMB	POS	NEG	POS	NEG	POS	NEG	POS	POS	NEG
2012_329_Z3616 (7577/12)	POS	AMB	POS	AMB	POS	AMB	POS	POS	POS	NEG	NEG	NEG	NEG	NEG	NEG	NEG	POS	NEG	POS	AMB	NEG	POS	AMB	NEG	NEG	POS	POS	NEG	POS	NEG	POS	NEG	POS	POS	NEG
2012_377_Z3437 (6242)	POS	AMB	POS	AMB	POS	NEG	POS	AMB	POS	NEG	NEG	NEG	NEG	NEG	NEG	NEG	POS	NEG	POS	NEG	NEG	POS	NEG	NEG	NEG	NEG	POS	NEG	POS	NEG	POS	NEG	POS	POS	AMB
2013_PA04_Z3987	AMB	POS	AMB	POS	POS	NEG	POS	POS	POS	NEG	NEG	POS	POS	NEG	NEG	NEG	POS	NEG	POS	NEG	NEG	POS	NEG	NEG	NEG	NEG	POS	NEG	POS	NEG	POS	NEG	POS	POS	NEG
2013_PA32_Z4015	POS	AMB	POS	AMB	POS	AMB	POS	POS	POS	NEG	NEG	NEG	POS	AMB	NEG	NEG	POS	NEG	POS	NEG	NEG	POS	AMB	NEG	NEG	AMB	POS	NEG	POS	NEG	POS	NEG	POS	POS	NEG
2013_PA41_Z4024	AMB	POS	AMB	POS	POS	NEG	POS	POS	POS	NEG	NEG	NEG	POS	NEG	NEG	NEG	POS	NEG	POS	NEG	NEG	POS	NEG	NEG	NEG	NEG	POS	NEG	POS	NEG	POS	NEG	POS	POS	NEG
2013_PA44_Z4027	AMB	POS	AMB	POS	POS	NEG	POS	POS	POS	NEG	NEG	NEG	POS	NEG	NEG	NEG	POS	NEG	POS	NEG	NEG	POS	NEG	NEG	NEG	NEG	POS	NEG	POS	NEG	POS	NEG	POS	POS	NEG
**CC5-MSSA [PVL+]**																																			
2012_175_Z3497 (112/12)	POS	AMB	POS	AMB	POS	AMB	POS	POS	POS	NEG	NEG	NEG	POS	NEG	NEG	NEG	POS	NEG	POS	NEG	NEG	POS	NEG	NEG	NEG	AMB	POS	NEG	POS	NEG	POS	NEG	POS	POS	NEG
2012_193_Z3458 (617/12)	POS	AMB	POS	AMB	POS	AMB	POS	POS	POS	NEG	NEG	NEG	POS	NEG	NEG	NEG	POS	NEG	POS	NEG	NEG	POS	NEG	NEG	NEG	AMB	POS	NEG	POS	NEG	POS	NEG	POS	POS	NEG
2012_215_Z3474 (1341/12)	POS	AMB	POS	AMB	POS	NEG	POS	POS	POS	NEG	NEG	NEG	POS	NEG	NEG	NEG	POS	NEG	POS	NEG	NEG	POS	NEG	NEG	NEG	AMB	POS	NEG	POS	NEG	POS	NEG	POS	POS	AMB
2012_216_Z3475 (1370/12)	POS	AMB	POS	AMB	POS	NEG	POS	POS	POS	NEG	NEG	NEG	POS	NEG	NEG	NEG	POS	NEG	POS	NEG	NEG	POS	NEG	NEG	NEG	NEG	POS	NEG	POS	NEG	POS	NEG	POS	POS	POS
2012_297_Z3584 (5140/12)	POS	AMB	POS	AMB	POS	NEG	POS	POS	POS	NEG	NEG	NEG	POS	NEG	NEG	NEG	POS	NEG	POS	NEG	NEG	POS	NEG	NEG	NEG	AMB	POS	NEG	POS	NEG	POS	NEG	POS	POS	NEG
2012_331_Z3618 (7644/12)	POS	AMB	POS	AMB	POS	AMB	POS	POS	POS	NEG	NEG	NEG	POS	NEG	NEG	NEG	POS	NEG	POS	NEG	NEG	POS	AMB	NEG	NEG	AMB	POS	NEG	POS	NEG	POS	NEG	POS	POS	NEG
2012_379_Z3439 (4126)	POS	AMB	POS	AMB	POS	AMB	POS	POS	POS	NEG	NEG	NEG	POS	NEG	NEG	NEG	POS	NEG	POS	NEG	NEG	POS	NEG	NEG	NEG	NEG	POS	NEG	POS	NEG	POS	NEG	POS	POS	NEG
2012_381_Z3441	POS	AMB	POS	AMB	POS	AMB	POS	POS	POS	AMB	NEG	NEG	POS	NEG	NEG	NEG	POS	NEG	POS	AMB	NEG	POS	NEG	NEG	NEG	POS	POS	NEG	POS	NEG	POS	NEG	POS	POS	POS
2013_PA39_Z4022	AMB	POS	POS	AMB	POS	NEG	POS	POS	POS	NEG	NEG	NEG	POS	NEG	NEG	NEG	POS	NEG	POS	NEG	NEG	POS	NEG	NEG	NEG	NEG	POS	NEG	POS	NEG	POS	NEG	POS	POS	NEG
2013_PA47_Z4030	POS	AMB	AMB	POS	POS	NEG	POS	POS	POS	NEG	NEG	NEG	POS	NEG	NEG	NEG	POS	NEG	POS	NEG	NEG	POS	NEG	NEG	NEG	NEG	POS	NEG	POS	NEG	POS	NEG	POS	POS	NEG
2013_PA61_I_Z4044	POS	AMB	POS	AMB	POS	AMB	POS	POS	POS	NEG	NEG	NEG	POS	NEG	NEG	NEG	POS	NEG	POS	NEG	NEG	POS	AMB	NEG	NEG	NEG	POS	NEG	POS	NEG	POS	NEG	POS	POS	AMB
2013_PA62_I_Z4045	AMB	POS	POS	AMB	POS	NEG	POS	POS	POS	NEG	NEG	NEG	POS	NEG	NEG	NEG	POS	NEG	POS	NEG	NEG	POS	NEG	NEG	NEG	NEG	POS	NEG	POS	NEG	POS	NEG	POS	POS	AMB
2013_PA74_Z4057	AMB	POS	POS	AMB	POS	NEG	POS	POS	POS	NEG	NEG	NEG	POS	NEG	NEG	NEG	POS	NEG	POS	NEG	NEG	POS	NEG	NEG	NEG	NEG	POS	NEG	POS	NEG	POS	NEG	POS	POS	NEG
2013_PA75_Z4058	POS	AMB	AMB	POS	POS	AMB	POS	POS	POS	NEG	NEG	NEG	POS	NEG	NEG	NEG	POS	NEG	POS	NEG	NEG	POS	NEG	NEG	NEG	NEG	POS	NEG	POS	NEG	POS	NEG	POS	POS	NEG
**CC5-MRSA-V [ACME+]**																																			
2012_373_Z3433 (1204-17312)	POS	AMB	POS	AMB	POS	AMB	POS	POS	POS	NEG	NEG	NEG	NEG	NEG	NEG	NEG	POS	NEG	POS	NEG	NEG	POS	AMB	NEG	NEG	AMB	POS	NEG	POS	NEG	POS	NEG	POS	POS	NEG
**CC6-MSSA**																																			
2012_173_Z3450 (98A/12)	POS	AMB	POS	AMB	POS	AMB	POS	POS	POS	NEG	NEG	NEG	AMB	NEG	NEG	NEG	POS	POS	NEG	POS	NEG	POS	NEG	NEG	POS	POS	NEG	NEG	POS	NEG	AMB	NEG	POS	POS	POS
2012_176_Z3498	POS	AMB	POS	AMB	POS	AMB	POS	POS	POS	NEG	NEG	NEG	NEG	NEG	NEG	NEG	POS	POS	NEG	POS	NEG	POS	NEG	NEG	POS	AMB	NEG	NEG	POS	NEG	NEG	NEG	POS	POS	NEG
2012_181_Z3452	POS	AMB	POS	AMB	POS	AMB	POS	POS	POS	NEG	NEG	NEG	NEG	NEG	NEG	NEG	POS	POS	NEG	POS	NEG	POS	AMB	NEG	POS	POS	NEG	NEG	POS	NEG	NEG	NEG	POS	POS	NEG
2012_259_Z3546 (3279/12)	POS	AMB	POS	AMB	POS	AMB	POS	POS	POS	NEG	NEG	NEG	NEG	NEG	NEG	NEG	POS	POS	NEG	POS	NEG	POS	AMB	NEG	POS	POS	NEG	NEG	POS	NEG	NEG	NEG	POS	POS	AMB
2012_264_Z3551 (34078/12)	POS	AMB	POS	AMB	POS	AMB	POS	POS	POS	NEG	NEG	NEG	NEG	NEG	NEG	NEG	POS	POS	NEG	POS	NEG	POS	AMB	NEG	POS	AMB	NEG	NEG	POS	NEG	NEG	NEG	POS	POS	AMB
2012_273_Z3560 (3844/12)	POS	AMB	POS	AMB	POS	AMB	POS	POS	POS	NEG	NEG	NEG	NEG	NEG	NEG	NEG	POS	POS	NEG	POS	NEG	POS	AMB	NEG	POS	POS	NEG	NEG	POS	NEG	NEG	NEG	POS	POS	NEG
2012_312_Z3599 (6815/12)	POS	AMB	POS	AMB	POS	AMB	POS	POS	POS	NEG	NEG	NEG	NEG	NEG	NEG	NEG	POS	POS	NEG	POS	NEG	POS	NEG	NEG	POS	AMB	NEG	NEG	POS	NEG	NEG	NEG	POS	POS	NEG
2012_338_Z3625	POS	AMB	POS	AMB	POS	AMB	POS	POS	POS	NEG	NEG	NEG	NEG	NEG	NEG	NEG	POS	POS	NEG	POS	NEG	POS	NEG	NEG	POS	AMB	NEG	NEG	POS	NEG	NEG	NEG	POS	POS	NEG
2012_352_Z3639	POS	AMB	POS	AMB	POS	AMB	POS	POS	POS	NEG	NEG	NEG	NEG	NEG	NEG	NEG	POS	POS	NEG	POS	NEG	POS	AMB	NEG	POS	AMB	NEG	NEG	POS	NEG	NEG	NEG	POS	POS	AMB
2012_368_Z3428	POS	AMB	POS	AMB	POS	AMB	POS	POS	POS	NEG	NEG	NEG	NEG	NEG	NEG	NEG	POS	POS	NEG	POS	NEG	POS	AMB	NEG	POS	NEG	NEG	NEG	POS	NEG	NEG	NEG	POS	POS	NEG
2013_PA12_Z3995	POS	AMB	POS	AMB	POS	NEG	POS	POS	POS	NEG	NEG	NEG	NEG	NEG	NEG	NEG	POS	POS	NEG	POS	NEG	POS	AMB	NEG	POS	NEG	NEG	NEG	POS	NEG	NEG	NEG	POS	POS	NEG
2013_PA28_Z4011	POS	AMB	POS	AMB	POS	NEG	POS	POS	POS	NEG	NEG	NEG	NEG	NEG	NEG	NEG	POS	POS	NEG	POS	NEG	POS	AMB	NEG	POS	NEG	NEG	NEG	POS	NEG	NEG	NEG	POS	POS	NEG
**CC6-MSSA [PVL+]**																																			
2012_375_Z3435	POS	AMB	POS	AMB	POS	AMB	POS	POS	POS	NEG	NEG	NEG	NEG	NEG	NEG	NEG	POS	POS	NEG	POS	NEG	POS	AMB	NEG	POS	AMB	NEG	NEG	POS	NEG	NEG	NEG	POS	POS	NEG
2013_PA77_Z4060	AMB	POS	POS	AMB	POS	NEG	POS	POS	POS	NEG	NEG	NEG	NEG	NEG	NEG	NEG	POS	POS	NEG	POS	NEG	POS	AMB	NEG	POS	NEG	NEG	NEG	POS	NEG	NEG	NEG	POS	POS	NEG
**CC7-MSSA**																																			
2012_248_Z3532 (2864/12)	POS	AMB	POS	AMB	POS	AMB	POS	POS	POS	NEG	NEG	NEG	NEG	NEG	NEG	NEG	NEG	NEG	NEG	NEG	NEG	POS	NEG	POS	NEG	AMB	POS	NEG	POS	NEG	AMB	NEG	POS	POS	AMB
2012_265_Z3552 (3518/12)	POS	AMB	AMB	POS	POS	AMB	POS	POS	POS	NEG	NEG	NEG	NEG	NEG	NEG	NEG	AMB	POS	NEG	POS	NEG	POS	NEG	POS	NEG	POS	POS	NEG	POS	NEG	AMB	NEG	POS	POS	AMB
2013_PA53_Z4036	POS	AMB	POS	AMB	POS	AMB	POS	POS	POS	NEG	NEG	NEG	NEG	NEG	NEG	NEG	NEG	NEG	NEG	NEG	NEG	POS	AMB	POS	NEG	AMB	POS	NEG	POS	NEG	POS	NEG	POS	POS	AMB
2013_PA83_Z4066	AMB	POS	AMB	POS	POS	NEG	POS	POS	POS	NEG	NEG	NEG	NEG	NEG	NEG	NEG	NEG	NEG	NEG	NEG	NEG	POS	NEG	POS	NEG	AMB	POS	NEG	POS	NEG	AMB	NEG	POS	POS	NEG
**CC8-MSSA**																																			
2012_243_Z3528 (2587/12)	POS	AMB	POS	AMB	POS	AMB	POS	POS	POS	NEG	NEG	NEG	POS	NEG	NEG	NEG	NEG	NEG	NEG	NEG	NEG	POS	NEG	NEG	NEG	AMB	NEG	NEG	POS	POS	POS	POS	NEG	NEG	AMB
2012_344_Z3631 (8605/12)	POS	AMB	AMB	POS	POS	AMB	POS	POS	POS	NEG	NEG	NEG	POS	NEG	NEG	NEG	POS	POS	NEG	NEG	NEG	POS	NEG	NEG	POS	AMB	NEG	NEG	POS	POS	POS	POS	NEG	NEG	NEG
2012_346_Z3633 (8074/12)	POS	AMB	AMB	POS	POS	AMB	POS	POS	POS	NEG	NEG	NEG	POS	NEG	NEG	NEG	POS	POS	NEG	NEG	NEG	POS	NEG	NEG	POS	AMB	NEG	NEG	POS	POS	POS	POS	NEG	NEG	AMB
2012_255_Z3538 (32058/12)	POS	AMB	AMB	POS	POS	AMB	POS	POS	POS	NEG	NEG	NEG	POS	NEG	NEG	NEG	POS	POS	NEG	NEG	NEG	POS	NEG	NEG	POS	POS	NEG	NEG	POS	POS	POS	POS	NEG	NEG	AMB
2012_280_Z3567 (4601/12)	POS	AMB	AMB	POS	POS	AMB	POS	POS	POS	NEG	NEG	NEG	POS	NEG	NEG	NEG	POS	POS	NEG	NEG	NEG	POS	NEG	NEG	POS	AMB	NEG	NEG	POS	POS	POS	POS	NEG	NEG	NEG
Trinidad&Tobago_2012_267 (3642/12)	POS	AMB	POS	AMB	POS	NEG	POS	POS	POS	NEG	NEG	NEG	POS	NEG	NEG	NEG	POS	POS	NEG	NEG	NEG	POS	NEG	NEG	POS	NEG	NEG	NEG	POS	POS	POS	POS	NEG	NEG	AMB
2012_365_Z3425 (1204-15838)	POS	AMB	AMB	POS	POS	AMB	POS	POS	POS	NEG	NEG	NEG	POS	NEG	NEG	NEG	POS	POS	NEG	NEG	NEG	POS	NEG	NEG	POS	AMB	NEG	NEG	POS	POS	POS	POS	NEG	NEG	AMB
2012_296_Z3583 (5118/12)	POS	AMB	AMB	POS	POS	NEG	POS	POS	POS	NEG	NEG	NEG	POS	NEG	NEG	NEG	POS	POS	NEG	NEG	NEG	POS	NEG	NEG	POS	AMB	NEG	NEG	POS	POS	POS	POS	NEG	NEG	NEG
2012_318_Z3605 (6960/12)	AMB	POS	AMB	POS	POS	AMB	POS	POS	POS	NEG	NEG	NEG	POS	NEG	NEG	NEG	POS	POS	NEG	NEG	NEG	POS	NEG	NEG	POS	AMB	NEG	NEG	POS	POS	POS	POS	NEG	NEG	NEG
2012_306_Z3593 (5861/12)	POS	AMB	POS	AMB	POS	AMB	POS	POS	POS	NEG	NEG	NEG	POS	NEG	NEG	NEG	POS	POS	NEG	NEG	NEG	POS	NEG	NEG	POS	NEG	NEG	NEG	POS	POS	POS	POS	NEG	NEG	AMB
2013_PA37_Z4020	AMB	POS	POS	AMB	POS	AMB	POS	POS	POS	NEG	NEG	NEG	POS	NEG	NEG	NEG	POS	POS	NEG	NEG	NEG	POS	NEG	NEG	POS	AMB	NEG	NEG	POS	POS	POS	POS	NEG	NEG	POS
2013_PA38_Z4021	POS	AMB	POS	AMB	POS	NEG	POS	POS	POS	NEG	NEG	NEG	POS	NEG	NEG	NEG	POS	POS	NEG	NEG	NEG	POS	NEG	NEG	AMB	NEG	NEG	NEG	POS	POS	POS	POS	NEG	NEG	NEG
2013_PA52_Z4035	POS	AMB	POS	AMB	POS	NEG	POS	POS	POS	NEG	NEG	NEG	POS	NEG	NEG	NEG	POS	POS	NEG	NEG	NEG	POS	NEG	NEG	POS	NEG	NEG	NEG	POS	POS	POS	POS	NEG	NEG	NEG
**CC8-MSSA [PVL+]**																																			
2012_156_Z3489 (25917/11)	POS	AMB	POS	AMB	POS	AMB	POS	POS	POS	NEG	NEG	NEG	POS	NEG	NEG	NEG	NEG	NEG	NEG	NEG	NEG	POS	NEG	NEG	NEG	AMB	NEG	NEG	POS	POS	POS	POS	NEG	NEG	NEG
2012_159_Z3492 (26310/11)	POS	AMB	AMB	POS	POS	AMB	POS	POS	POS	NEG	NEG	NEG	POS	NEG	NEG	NEG	AMB	NEG	NEG	NEG	NEG	POS	NEG	NEG	NEG	AMB	NEG	NEG	POS	POS	POS	POS	NEG	NEG	NEG
2012_161_Z3445 (26430/11)	POS	AMB	POS	AMB	POS	AMB	POS	POS	POS	NEG	NEG	NEG	POS	NEG	NEG	NEG	AMB	NEG	NEG	NEG	NEG	POS	NEG	NEG	NEG	POS	NEG	NEG	POS	POS	POS	POS	NEG	NEG	AMB
2012_162_Z3512 (26433/11)	POS	AMB	POS	AMB	POS	AMB	POS	POS	POS	NEG	NEG	NEG	POS	NEG	NEG	NEG	NEG	NEG	NEG	NEG	NEG	POS	NEG	NEG	NEG	AMB	NEG	NEG	POS	POS	POS	POS	NEG	NEG	NEG
2012_169_Z3495 (27194/11)	POS	AMB	POS	AMB	POS	AMB	POS	POS	POS	NEG	NEG	NEG	POS	NEG	NEG	NEG	NEG	NEG	NEG	NEG	NEG	POS	NEG	NEG	NEG	AMB	NEG	NEG	POS	POS	POS	POS	NEG	NEG	NEG
2012_170_Z3496 (35/12)	POS	AMB	POS	AMB	POS	AMB	POS	POS	POS	NEG	NEG	NEG	POS	NEG	NEG	NEG	NEG	NEG	NEG	NEG	NEG	POS	NEG	NEG	NEG	AMB	NEG	NEG	POS	POS	POS	POS	NEG	NEG	NEG
2012_171_Z3448 (47/12)	POS	AMB	POS	AMB	POS	AMB	POS	POS	POS	NEG	NEG	NEG	POS	NEG	NEG	NEG	NEG	NEG	NEG	NEG	NEG	POS	NEG	NEG	NEG	AMB	NEG	NEG	POS	POS	POS	POS	NEG	NEG	NEG
2012_177_Z3499 (184/12)	POS	AMB	POS	AMB	POS	AMB	POS	POS	POS	NEG	NEG	NEG	POS	NEG	NEG	NEG	NEG	NEG	NEG	NEG	NEG	POS	NEG	NEG	NEG	AMB	NEG	NEG	POS	POS	POS	POS	NEG	NEG	AMB
2012_179_Z3515 (231/12)	POS	AMB	POS	AMB	POS	AMB	POS	POS	POS	NEG	NEG	NEG	POS	NEG	NEG	NEG	NEG	NEG	NEG	NEG	NEG	POS	NEG	NEG	NEG	AMB	NEG	NEG	POS	POS	POS	POS	NEG	NEG	AMB
2012_185_Z3502 (437/12)	POS	AMB	POS	AMB	POS	NEG	POS	POS	POS	NEG	NEG	NEG	POS	NEG	NEG	NEG	NEG	NEG	NEG	NEG	NEG	POS	NEG	NEG	NEG	NEG	NEG	NEG	POS	POS	POS	POS	NEG	NEG	NEG
2012_191_Z3456 (563/12)	POS	AMB	POS	AMB	POS	AMB	POS	POS	POS	NEG	NEG	NEG	POS	NEG	NEG	NEG	NEG	NEG	NEG	NEG	NEG	POS	NEG	NEG	NEG	AMB	NEG	NEG	POS	POS	POS	POS	NEG	NEG	AMB
2012_194_Z3459	POS	AMB	POS	AMB	POS	AMB	POS	POS	POS	NEG	NEG	NEG	POS	NEG	NEG	NEG	NEG	NEG	NEG	NEG	NEG	NEG	NEG	NEG	NEG	AMB	NEG	NEG	POS	POS	POS	POS	NEG	NEG	AMB
2012_200_Z3460 (820/12)	POS	AMB	POS	AMB	POS	AMB	POS	POS	POS	NEG	NEG	NEG	POS	NEG	NEG	NEG	AMB	NEG	NEG	NEG	NEG	POS	AMB	NEG	NEG	POS	NEG	NEG	POS	POS	POS	POS	NEG	NEG	POS
2012_207_Z3467 (1010/12)	POS	AMB	POS	AMB	POS	AMB	POS	POS	POS	NEG	NEG	NEG	POS	NEG	NEG	NEG	NEG	NEG	NEG	NEG	NEG	POS	NEG	NEG	NEG	AMB	NEG	NEG	POS	POS	POS	POS	NEG	NEG	POS
2012_212_Z3471 (1261/12)	POS	AMB	POS	AMB	POS	NEG	POS	POS	POS	NEG	NEG	NEG	POS	NEG	NEG	NEG	NEG	NEG	NEG	NEG	NEG	POS	NEG	NEG	NEG	NEG	NEG	NEG	POS	POS	POS	POS	NEG	NEG	NEG
2012_220_Z3477 (1466/12)	POS	AMB	POS	AMB	POS	NEG	POS	POS	POS	NEG	NEG	NEG	POS	NEG	NEG	NEG	NEG	NEG	NEG	NEG	NEG	POS	NEG	NEG	NEG	NEG	NEG	NEG	POS	POS	POS	POS	NEG	NEG	AMB
2012_224_Z3481 (1522/12)	POS	AMB	POS	AMB	POS	NEG	POS	POS	POS	NEG	NEG	NEG	POS	NEG	NEG	NEG	NEG	NEG	NEG	NEG	NEG	POS	NEG	NEG	NEG	NEG	NEG	NEG	POS	POS	POS	POS	NEG	NEG	AMB
2012_226_Z3483 (1662/12)	POS	AMB	POS	AMB	POS	AMB	POS	POS	POS	NEG	NEG	NEG	POS	NEG	NEG	NEG	NEG	NEG	NEG	NEG	NEG	POS	NEG	NEG	NEG	POS	NEG	NEG	POS	POS	POS	POS	NEG	NEG	NEG
2012_242_Z3527 (2581/12)	POS	AMB	POS	AMB	POS	AMB	POS	POS	POS	NEG	NEG	NEG	POS	NEG	NEG	NEG	NEG	NEG	NEG	NEG	NEG	POS	NEG	NEG	NEG	AMB	NEG	NEG	POS	POS	POS	POS	NEG	NEG	NEG
2012_245_Z3529 (2687/12)	POS	AMB	POS	AMB	POS	AMB	POS	POS	POS	NEG	NEG	NEG	POS	NEG	NEG	NEG	NEG	NEG	NEG	NEG	NEG	POS	NEG	NEG	NEG	AMB	NEG	NEG	POS	POS	POS	POS	NEG	NEG	NEG
2012_253_Z3536 (3074/12)	POS	AMB	POS	AMB	POS	AMB	POS	POS	POS	NEG	NEG	NEG	POS	NEG	NEG	NEG	NEG	NEG	NEG	NEG	NEG	POS	NEG	NEG	NEG	AMB	NEG	NEG	POS	POS	POS	POS	NEG	NEG	AMB
2012_258_Z3545 (3231/12)	POS	AMB	POS	AMB	POS	AMB	POS	POS	POS	NEG	NEG	NEG	POS	NEG	NEG	NEG	NEG	NEG	NEG	NEG	NEG	POS	AMB	NEG	NEG	NEG	NEG	NEG	POS	POS	POS	POS	NEG	NEG	AMB
2012_261_Z3548 (3298/12)	POS	AMB	POS	AMB	POS	AMB	POS	POS	POS	POS	POS	NEG	POS	NEG	NEG	NEG	NEG	NEG	NEG	POS	POS	POS	NEG	POS	NEG	POS	NEG	NEG	POS	POS	POS	POS	NEG	NEG	AMB
2012_270_Z3557 (3798/12)	POS	AMB	POS	AMB	POS	AMB	POS	POS	POS	NEG	NEG	NEG	POS	NEG	POS	NEG	NEG	NEG	NEG	NEG	NEG	POS	NEG	NEG	NEG	POS	NEG	POS	POS	POS	POS	POS	POS	POS	NEG
2012_274_Z3561 (3861/12)	POS	AMB	POS	AMB	POS	AMB	POS	POS	POS	NEG	NEG	NEG	POS	NEG	NEG	NEG	NEG	NEG	NEG	NEG	NEG	POS	NEG	NEG	NEG	AMB	NEG	NEG	POS	POS	POS	POS	NEG	NEG	NEG
2012_282_Z3569 (4698/12)	POS	AMB	POS	AMB	POS	NEG	POS	POS	POS	NEG	NEG	NEG	POS	NEG	NEG	NEG	NEG	NEG	NEG	NEG	NEG	POS	NEG	NEG	NEG	AMB	NEG	NEG	POS	POS	POS	POS	NEG	NEG	AMB
2012_286_Z3573 (4805/12)	POS	AMB	POS	AMB	POS	NEG	POS	POS	POS	NEG	NEG	NEG	POS	NEG	NEG	NEG	NEG	NEG	NEG	NEG	NEG	POS	NEG	NEG	NEG	AMB	NEG	NEG	POS	POS	POS	POS	NEG	NEG	NEG
2012_293_Z3580 (5007/12)	POS	AMB	POS	AMB	POS	NEG	POS	POS	POS	NEG	NEG	NEG	POS	NEG	NEG	NEG	NEG	NEG	NEG	NEG	NEG	POS	NEG	NEG	NEG	AMB	NEG	NEG	POS	POS	POS	POS	NEG	NEG	NEG
2012_294_Z3581	POS	AMB	POS	AMB	POS	NEG	POS	POS	POS	NEG	NEG	NEG	POS	NEG	NEG	NEG	NEG	NEG	NEG	NEG	NEG	POS	NEG	NEG	NEG	AMB	NEG	NEG	POS	POS	POS	POS	NEG	NEG	NEG
2012_307_Z3594 (5918/12)	POS	AMB	AMB	POS	POS	AMB	POS	POS	POS	NEG	NEG	NEG	POS	NEG	NEG	NEG	NEG	NEG	NEG	NEG	NEG	POS	AMB	NEG	NEG	POS	NEG	NEG	POS	POS	POS	POS	NEG	NEG	NEG
2012_333_Z3620 (8101/12)	POS	AMB	POS	AMB	POS	AMB	POS	POS	POS	NEG	NEG	NEG	POS	NEG	NEG	NEG	NEG	NEG	NEG	NEG	NEG	POS	NEG	NEG	NEG	AMB	NEG	NEG	POS	POS	POS	POS	NEG	NEG	NEG
2012_343_Z3630 (8195/12)	POS	AMB	POS	AMB	POS	AMB	POS	POS	POS	NEG	NEG	NEG	POS	NEG	NEG	NEG	NEG	NEG	NEG	NEG	NEG	POS	NEG	NEG	NEG	AMB	NEG	NEG	POS	POS	POS	POS	NEG	NEG	NEG
2012_349_Z3636 (7999A/12)	POS	AMB	AMB	POS	POS	AMB	POS	POS	POS	NEG	NEG	NEG	POS	NEG	NEG	NEG	NEG	NEG	NEG	NEG	NEG	POS	NEG	NEG	NEG	AMB	NEG	NEG	POS	POS	POS	POS	NEG	NEG	AMB
2012_353_Z3640 (8575/12)	POS	AMB	POS	AMB	POS	AMB	POS	POS	POS	NEG	NEG	NEG	POS	NEG	NEG	NEG	POS	POS	NEG	NEG	NEG	POS	NEG	NEG	POS	AMB	NEG	NEG	POS	POS	POS	POS	NEG	NEG	AMB
2012_354_Z3641 (8408/12)	POS	AMB	POS	AMB	POS	AMB	POS	POS	POS	NEG	NEG	NEG	POS	NEG	NEG	NEG	AMB	NEG	NEG	NEG	NEG	POS	NEG	NEG	NEG	POS	NEG	NEG	POS	POS	POS	POS	NEG	NEG	AMB
2012_355_Z3642 (1112-58348)	AMB	POS	AMB	POS	POS	NEG	POS	POS	POS	NEG	NEG	NEG	POS	NEG	NEG	NEG	NEG	NEG	NEG	NEG	NEG	POS	NEG	NEG	NEG	NEG	NEG	NEG	POS	POS	POS	POS	NEG	NEG	NEG
2012_357_Z3417 (1202-6360)	POS	AMB	POS	AMB	POS	AMB	POS	POS	POS	NEG	NEG	NEG	POS	AMB	NEG	NEG	AMB	NEG	NEG	NEG	NEG	POS	NEG	NEG	NEG	POS	NEG	NEG	POS	POS	POS	POS	NEG	NEG	AMB
2012_364_Z3424 (1112-60417)	POS	AMB	POS	AMB	POS	NEG	POS	POS	POS	NEG	NEG	NEG	POS	NEG	NEG	NEG	AMB	NEG	NEG	NEG	NEG	POS	NEG	NEG	NEG	NEG	NEG	NEG	POS	POS	POS	POS	NEG	NEG	NEG
2012_376_Z3436 (6256)	POS	AMB	POS	AMB	POS	AMB	POS	POS	POS	NEG	NEG	NEG	POS	NEG	NEG	NEG	AMB	NEG	NEG	NEG	NEG	POS	NEG	NEG	NEG	AMB	NEG	NEG	POS	POS	POS	POS	NEG	NEG	POS
2012_383_Z3443	AMB	POS	POS	AMB	POS	AMB	POS	POS	POS	POS	POS	NEG	POS	NEG	NEG	NEG	AMB	NEG	NEG	NEG	NEG	POS	NEG	POS	NEG	POS	NEG	NEG	POS	POS	POS	POS	NEG	NEG	NEG
2013_PA01_Z3984	AMB	POS	POS	AMB	POS	AMB	POS	POS	POS	NEG	NEG	NEG	POS	AMB	NEG	NEG	NEG	NEG	NEG	NEG	NEG	POS	NEG	NEG	NEG	NEG	NEG	NEG	POS	POS	POS	POS	NEG	NEG	AMB
2013_PA08_Z3991	AMB	POS	POS	AMB	POS	AMB	POS	POS	POS	NEG	NEG	NEG	POS	AMB	NEG	NEG	NEG	NEG	NEG	NEG	NEG	POS	NEG	NEG	NEG	NEG	NEG	NEG	POS	POS	POS	POS	NEG	NEG	AMB
2013_PA10_Z3993	AMB	POS	POS	AMB	POS	AMB	POS	POS	POS	NEG	NEG	NEG	POS	NEG	NEG	NEG	NEG	NEG	NEG	NEG	NEG	POS	NEG	NEG	NEG	NEG	NEG	NEG	POS	POS	POS	POS	NEG	NEG	AMB
2013_PA13_Z3996	POS	AMB	POS	AMB	POS	AMB	POS	POS	POS	NEG	NEG	NEG	POS	NEG	NEG	NEG	NEG	NEG	NEG	NEG	NEG	POS	NEG	NEG	NEG	NEG	NEG	NEG	POS	POS	POS	POS	NEG	NEG	NEG
2013_PA14_Z3997	AMB	POS	POS	AMB	POS	AMB	POS	POS	POS	NEG	NEG	NEG	POS	NEG	NEG	NEG	NEG	NEG	NEG	NEG	NEG	POS	NEG	NEG	NEG	NEG	NEG	NEG	POS	POS	POS	POS	NEG	NEG	NEG
2013_PA17_Z4000	AMB	POS	POS	AMB	POS	NEG	POS	POS	POS	NEG	NEG	NEG	POS	NEG	NEG	NEG	NEG	NEG	NEG	NEG	NEG	POS	NEG	NEG	NEG	NEG	NEG	NEG	POS	POS	POS	POS	NEG	NEG	NEG
2013_PA26_Z4009	AMB	POS	POS	AMB	POS	NEG	POS	POS	POS	NEG	NEG	NEG	POS	NEG	NEG	NEG	NEG	NEG	NEG	NEG	NEG	AMB	NEG	NEG	NEG	NEG	NEG	NEG	POS	POS	POS	POS	NEG	NEG	AMB
2013_PA27_Z4010	AMB	POS	POS	AMB	POS	NEG	POS	POS	POS	NEG	NEG	NEG	POS	NEG	NEG	NEG	NEG	NEG	NEG	NEG	NEG	POS	NEG	NEG	NEG	AMB	NEG	NEG	POS	POS	POS	POS	NEG	NEG	AMB
2013_PA42_Z4025	AMB	POS	POS	AMB	POS	NEG	POS	POS	POS	NEG	NEG	NEG	POS	NEG	NEG	NEG	NEG	NEG	NEG	NEG	NEG	POS	NEG	NEG	NEG	NEG	NEG	NEG	POS	POS	POS	POS	NEG	NEG	NEG
2013_PA43_Z4026	POS	AMB	POS	AMB	POS	NEG	POS	POS	POS	NEG	NEG	NEG	POS	NEG	NEG	NEG	NEG	NEG	NEG	NEG	NEG	POS	NEG	NEG	NEG	NEG	NEG	NEG	POS	POS	POS	POS	NEG	NEG	NEG
2013_PA45_Z4028	POS	AMB	POS	AMB	POS	NEG	POS	POS	POS	NEG	NEG	NEG	POS	NEG	NEG	NEG	NEG	NEG	NEG	NEG	NEG	POS	NEG	NEG	NEG	AMB	NEG	NEG	POS	POS	POS	POS	NEG	NEG	NEG
2013_PA56_Z4039	AMB	POS	POS	AMB	POS	NEG	POS	POS	POS	NEG	NEG	NEG	POS	NEG	NEG	NEG	NEG	NEG	NEG	NEG	NEG	POS	NEG	NEG	NEG	NEG	NEG	NEG	POS	POS	POS	POS	NEG	NEG	NEG
2013_PA58_Z4041	POS	AMB	AMB	POS	POS	AMB	POS	POS	POS	NEG	NEG	NEG	POS	NEG	NEG	NEG	AMB	NEG	NEG	NEG	NEG	POS	NEG	NEG	NEG	AMB	NEG	NEG	POS	POS	POS	POS	NEG	NEG	AMB
2013_PA64_Z4047	POS	AMB	POS	AMB	POS	AMB	POS	POS	POS	NEG	NEG	NEG	POS	NEG	NEG	NEG	NEG	NEG	NEG	NEG	NEG	POS	NEG	NEG	NEG	AMB	NEG	NEG	POS	POS	POS	POS	NEG	NEG	NEG
2013_PA66_Z4049	AMB	POS	POS	AMB	POS	NEG	POS	POS	POS	NEG	NEG	NEG	POS	NEG	NEG	NEG	NEG	NEG	NEG	NEG	NEG	POS	NEG	NEG	NEG	NEG	NEG	NEG	POS	POS	POS	POS	NEG	NEG	AMB
2013_PA67_Z4050	AMB	POS	POS	AMB	POS	AMB	POS	POS	POS	NEG	NEG	NEG	POS	NEG	NEG	NEG	NEG	NEG	NEG	NEG	NEG	POS	NEG	NEG	NEG	AMB	NEG	NEG	POS	POS	POS	POS	NEG	NEG	NEG
2013_PA69_Z4052	AMB	POS	POS	AMB	POS	NEG	POS	POS	POS	NEG	NEG	NEG	POS	NEG	NEG	NEG	NEG	NEG	NEG	NEG	NEG	POS	NEG	NEG	NEG	NEG	NEG	NEG	POS	POS	POS	POS	NEG	NEG	NEG
2013_PA72_Z4055	POS	AMB	POS	AMB	POS	AMB	POS	POS	POS	NEG	NEG	NEG	POS	NEG	NEG	NEG	NEG	NEG	NEG	NEG	NEG	POS	NEG	NEG	NEG	NEG	NEG	NEG	POS	POS	POS	POS	NEG	NEG	NEG
2013_PA76_Z4059	AMB	POS	POS	AMB	POS	AMB	POS	POS	POS	NEG	NEG	NEG	POS	NEG	NEG	NEG	NEG	NEG	NEG	NEG	NEG	POS	NEG	NEG	NEG	AMB	NEG	NEG	POS	POS	POS	POS	NEG	NEG	NEG
2013_PA80_Z4063	AMB	POS	POS	AMB	POS	NEG	POS	POS	POS	NEG	NEG	NEG	POS	NEG	NEG	NEG	NEG	NEG	NEG	NEG	NEG	POS	NEG	NEG	NEG	NEG	NEG	NEG	POS	POS	POS	POS	NEG	NEG	NEG

Strain / Isolates	immunodominant antigen B		defensin resistance protein		transferrin-binding protein			hypothetical protein, similar to integral membrane protein LmrP				type I site-specific deoxyribo-nuclease subunit, 1st locus	type I site-specific deoxyribonuclease subunit, 2nd locus				type I site-specific deoxyribonuclease subunit, 3rd locus
	isaB	isaB (MRSA252)	mprF (COL+MW2)	mprF (Mu50+MRSA252)	isdA (consensus)	isdA (MRSA252)	isdA (other than MRSA252)	lmrP (other than RF122)_probe1	lmrP (other than RF122)_probe2	lmrP (RF122)_probe1	lmrP (RF122)_probe2	hsdS1 (RF122)	hsdS2 (Mu50+N315+COL+USA300+NCTC8325	hsdS2 (MW2+MSSA476)	hsdS2 (RF122)	hsdS2 (MRSA252)	hsdS3 (all other than RF122+ MRSA252)
**ST8-MRSA-IV, USA300**																	
2012_158_Z3491 (26049A/11)	POS	AMB	AMB	POS	POS	AMB	POS	POS	POS	NEG	NEG	NEG	POS	NEG	NEG	NEG	POS
2012_165_Z3513 (26656/11)	POS	AMB	POS	AMB	POS	AMB	POS	POS	POS	NEG	NEG	NEG	POS	NEG	NEG	NEG	POS
2012_214_Z3473 (1322/12)	POS	AMB	POS	AMB	POS	NEG	POS	POS	POS	NEG	NEG	NEG	POS	NEG	NEG	NEG	POS
2012_227_Z3484 (1688/12)	POS	AMB	AMB	POS	POS	AMB	POS	POS	POS	NEG	NEG	NEG	POS	NEG	NEG	NEG	POS
2012_263_Z3550 (3355/12)	POS	AMB	AMB	POS	POS	AMB	POS	POS	POS	NEG	NEG	NEG	POS	NEG	NEG	NEG	POS
2012_266_Z3553	POS	AMB	AMB	POS	POS	AMB	POS	POS	POS	NEG	NEG	NEG	POS	NEG	NEG	NEG	POS
2012_284_Z3571 (4729/12)	POS	AMB	POS	AMB	POS	NEG	POS	POS	POS	NEG	NEG	NEG	POS	NEG	NEG	NEG	POS
2012_298_Z3585 (5203/12)	POS	AMB	AMB	POS	POS	NEG	POS	POS	POS	NEG	NEG	NEG	POS	NEG	NEG	NEG	POS
2012_304_Z3591 (5818/12)	POS	AMB	AMB	POS	POS	NEG	POS	POS	POS	NEG	NEG	NEG	POS	NEG	NEG	NEG	POS
Z3595_TT308 - {EFC74B77-3925-484F-A4CF-A1F7DC	AMB	POS	AMB	POS	POS	AMB	POS	POS	POS	NEG	NEG	NEG	POS	NEG	NEG	NEG	POS
2012_369_Z3429 (1112-58547)	POS	AMB	POS	AMB	POS	NEG	POS	POS	POS	NEG	NEG	NEG	POS	NEG	NEG	NEG	POS
2012_374_Z3434 (1203-13527)	POS	AMB	AMB	POS	POS	AMB	POS	POS	POS	NEG	NEG	NEG	POS	NEG	NEG	NEG	POS
2013_PA06_Z3989	AMB	POS	POS	AMB	POS	NEG	POS	POS	POS	NEG	NEG	NEG	POS	NEG	NEG	NEG	POS
2013_PA55_Z4038	POS	AMB	POS	AMB	POS	AMB	POS	POS	POS	NEG	NEG	NEG	POS	NEG	NEG	NEG	POS
2013_PA71_Z4054	AMB	POS	POS	AMB	POS	NEG	POS	POS	POS	NEG	NEG	NEG	POS	NEG	NEG	NEG	POS
**ST8-MRSA-IV [PVL+/ACME+], US**																	
2012_180_Z3501 (232/12)	POS	AMB	POS	AMB	POS	NEG	POS	POS	POS	NEG	NEG	NEG	POS	NEG	NEG	NEG	POS
2013_PA68_Z4051	POS	AMB	POS	AMB	POS	NEG	POS	POS	POS	NEG	NEG	NEG	POS	NEG	NEG	NEG	POS
**ST72-MSSA**																	
2012_174_Z3451 (102/12)	POS	AMB	AMB	POS	POS	AMB	POS	POS	POS	NEG	NEG	NEG	NEG	NEG	NEG	NEG	POS
2012_223_Z3480 (1506A/12)	POS	AMB	POS	AMB	POS	NEG	POS	POS	POS	NEG	NEG	NEG	NEG	NEG	NEG	NEG	POS
2012_236_Z3523 (2086/12)	POS	AMB	POS	AMB	POS	AMB	POS	POS	POS	NEG	NEG	NEG	NEG	NEG	NEG	NEG	POS
2012_239_Z3525 (2346/12)	POS	AMB	POS	AMB	POS	AMB	POS	POS	POS	NEG	NEG	NEG	NEG	NEG	NEG	NEG	POS
2012_252_Z3535 (3030/12)	POS	AMB	POS	AMB	POS	AMB	POS	POS	POS	NEG	NEG	NEG	NEG	NEG	NEG	NEG	POS
2012_271_Z3558 (3799/12)	POS	AMB	POS	AMB	POS	AMB	POS	POS	POS	NEG	NEG	NEG	NEG	NEG	NEG	NEG	POS
2012_276_Z3563	POS	AMB	POS	AMB	POS	AMB	POS	POS	POS	NEG	NEG	NEG	NEG	NEG	NEG	NEG	POS
2012_300_Z3587 (5638/12)	POS	AMB	AMB	POS	POS	NEG	POS	POS	POS	NEG	NEG	NEG	NEG	NEG	NEG	NEG	POS
2012_316_Z3603 (6941/12)	POS	AMB	AMB	POS	POS	AMB	POS	POS	POS	NEG	NEG	NEG	NEG	NEG	NEG	NEG	POS
2012_319_Z3606 (7019/12)	POS	AMB	AMB	POS	POS	AMB	POS	POS	POS	NEG	NEG	NEG	NEG	NEG	NEG	NEG	POS
2012_371_Z3431 (1204-17577)	POS	AMB	AMB	POS	POS	AMB	POS	POS	POS	NEG	NEG	NEG	NEG	NEG	NEG	NEG	POS
2013_PA05_Z3988	AMB	POS	POS	AMB	POS	NEG	POS	POS	POS	NEG	NEG	NEG	NEG	NEG	NEG	NEG	POS
2013_PA65_Z4048	POS	AMB	POS	AMB	POS	NEG	POS	POS	POS	NEG	NEG	NEG	NEG	NEG	NEG	NEG	AMB
2013_PA73_Z4056	AMB	POS	POS	AMB	POS	NEG	POS	POS	POS	NEG	NEG	NEG	NEG	NEG	NEG	NEG	POS
**ST72-MSSA-SCCfus**																	
2013_PA16_Z3999	AMB	POS	POS	AMB	POS	NEG	POS	POS	POS	NEG	NEG	NEG	NEG	NEG	NEG	NEG	POS
**ST72-MSSA-SCCfus/kdp**																	
2012_361_Z3421 (1201-1299)	POS	AMB	POS	AMB	POS	AMB	POS	POS	POS	NEG	NEG	NEG	NEG	NEG	NEG	NEG	POS
**ST239-MRSA-III&SCCmer**																	
2012_157_Z3490	NEG	POS	POS	AMB	POS	AMB	POS	POS	POS	NEG	NEG	NEG	POS	NEG	NEG	NEG	POS
2012_168_Z3494 (27129/11)	NEG	POS	POS	AMB	POS	AMB	POS	POS	POS	NEG	NEG	NEG	POS	NEG	NEG	NEG	POS
2012_195_Z3508 (619/12)	NEG	POS	AMB	POS	POS	AMB	POS	POS	POS	NEG	NEG	NEG	POS	NEG	NEG	NEG	POS
2012_222_Z3479 (1489/12)	NEG	POS	POS	AMB	POS	AMB	POS	POS	POS	NEG	NEG	NEG	POS	NEG	NEG	NEG	POS
2012_233_Z3540 (1943/12)	NEG	POS	POS	AMB	POS	AMB	POS	POS	POS	NEG	NEG	NEG	POS	NEG	NEG	NEG	POS
2012_237_Z3541	NEG	POS	POS	AMB	POS	AMB	POS	POS	POS	NEG	NEG	NEG	POS	NEG	NEG	NEG	POS
2012_262_Z3549	AMB	POS	POS	AMB	POS	AMB	POS	POS	POS	NEG	NEG	NEG	POS	NEG	NEG	NEG	POS
2012_275_Z3562 (3893/12)	AMB	POS	POS	AMB	POS	AMB	POS	POS	POS	NEG	NEG	NEG	POS	NEG	NEG	NEG	POS
2012_291_Z3578	NEG	POS	POS	AMB	POS	NEG	POS	POS	POS	NEG	NEG	NEG	POS	NEG	NEG	NEG	POS
2012_314_Z3601 (6939/12)	AMB	POS	AMB	POS	POS	AMB	POS	POS	POS	NEG	NEG	NEG	POS	NEG	NEG	NEG	POS
2012323_Z3610	AMB	POS	POS	AMB	POS	AMB	POS	POS	POS	NEG	NEG	NEG	POS	NEG	NEG	NEG	POS
2012330_Z3617 (7605/12)	AMB	POS	POS	AMB	POS	AMB	POS	POS	POS	NEG	NEG	NEG	POS	NEG	NEG	NEG	POS
2012332_Z3619 (8076/12)	AMB	POS	POS	AMB	POS	AMB	POS	POS	POS	NEG	NEG	NEG	POS	NEG	NEG	NEG	POS
2012339_Z3626 (8558/12)	NEG	POS	POS	AMB	POS	AMB	POS	POS	POS	NEG	NEG	NEG	POS	NEG	NEG	NEG	POS
2012340_Z3627 (8523/12)	NEG	POS	POS	AMB	POS	AMB	POS	POS	POS	NEG	NEG	NEG	POS	NEG	NEG	NEG	POS
2012348_Z3635	AMB	POS	AMB	POS	POS	AMB	POS	POS	POS	NEG	NEG	NEG	POS	NEG	NEG	NEG	POS
2012350_Z3637	NEG	POS	POS	AMB	POS	AMB	POS	POS	POS	NEG	NEG	NEG	POS	NEG	NEG	NEG	POS
2012_370_Z3430 (1112-52177)	NEG	POS	AMB	POS	POS	AMB	POS	POS	POS	NEG	NEG	NEG	POS	NEG	NEG	NEG	POS
2012_378_Z3438 (4139)	NEG	POS	AMB	POS	POS	AMB	POS	POS	POS	NEG	NEG	NEG	POS	NEG	NEG	NEG	POS
2013_PA21_Z4004	NEG	POS	POS	AMB	POS	NEG	POS	POS	POS	NEG	NEG	NEG	POS	NEG	NEG	NEG	POS
2013_PA22_Z4005	AMB	POS	AMB	POS	POS	AMB	POS	POS	POS	NEG	NEG	NEG	POS	NEG	NEG	NEG	POS
2013_PA24_Z4007	NEG	POS	POS	AMB	POS	NEG	POS	POS	POS	NEG	NEG	NEG	POS	NEG	NEG	NEG	POS
2013_PA31_Z4014	NEG	POS	POS	AMB	POS	NEG	POS	POS	POS	NEG	NEG	NEG	POS	NEG	NEG	NEG	POS
2013_PA35_Z4018	NEG	POS	POS	AMB	POS	NEG	POS	POS	POS	NEG	NEG	NEG	POS	NEG	NEG	NEG	POS
2013_PA81_Z4064	NEG	POS	POS	AMB	POS	NEG	POS	POS	POS	NEG	NEG	NEG	POS	NEG	NEG	NEG	POS
**ST239-MRSA-III+ccrA/B4**																	
2012_254_Z3537 (3124A/12)	NEG	POS	POS	AMB	POS	NEG	POS	POS	POS	NEG	NEG	NEG	POS	NEG	NEG	NEG	POS
2013_PA25_Z4008	NEG	POS	POS	AMB	POS	NEG	POS	POS	POS	NEG	NEG	NEG	POS	NEG	NEG	NEG	POS
**ST834-MSSA**																	
2012_268_Z3555 (3693/12)	POS	AMB	AMB	POS	POS	NEG	POS	POS	POS	NEG	NEG	NEG	NEG	POS	NEG	NEG	POS
**CC12-MSSA**																	
2012_210_Z3470 (1177/12)	POS	AMB	POS	AMB	POS	NEG	POS	POS	POS	NEG	NEG	NEG	POS	NEG	NEG	NEG	POS
2012_230_Z3487 (1797/12)	POS	AMB	POS	AMB	POS	AMB	POS	POS	POS	NEG	NEG	NEG	POS	NEG	NEG	NEG	POS
2013_PA18_Z4001	AMB	POS	POS	AMB	POS	AMB	POS	POS	POS	NEG	NEG	NEG	POS	NEG	NEG	NEG	POS
**CC15-MSSA**																	
2012_160_Z3444 (26386/11)	AMB	POS	AMB	POS	POS	AMB	POS	POS	POS	NEG	NEG	NEG	NEG	NEG	NEG	NEG	POS
2012_167_Z3514 (27010/11)	POS	AMB	POS	AMB	POS	AMB	POS	POS	POS	NEG	NEG	NEG	NEG	NEG	NEG	NEG	POS
2012_184_Z3455 (376/12)	POS	AMB	AMB	POS	POS	AMB	POS	POS	POS	NEG	NEG	NEG	NEG	NEG	NEG	NEG	POS
2012_205_Z3465 (939/12)	POS	AMB	AMB	POS	POS	POS	AMB	POS	POS	NEG	NEG	NEG	NEG	NEG	NEG	NEG	POS
2012_231_Z3519 (1831/12)	POS	AMB	POS	AMB	POS	AMB	POS	POS	POS	NEG	NEG	NEG	NEG	NEG	NEG	NEG	POS
2012_249_Z3533 (2907/12)	POS	AMB	POS	AMB	POS	POS	AMB	POS	POS	NEG	NEG	NEG	NEG	NEG	NEG	NEG	POS
2012_305_Z3592 (5839/12)	POS	AMB	AMB	POS	POS	POS	AMB	POS	POS	NEG	NEG	NEG	NEG	NEG	NEG	NEG	POS
2012_313_Z3600 (6857/12)	POS	AMB	AMB	POS	POS	POS	AMB	POS	POS	NEG	NEG	NEG	NEG	NEG	NEG	NEG	POS
2012_345_Z3632 (7966B/12)	POS	AMB	AMB	POS	POS	POS	AMB	POS	POS	NEG	NEG	NEG	NEG	NEG	NEG	NEG	POS
2013_PA33_Z4016	POS	AMB	POS	AMB	POS	POS	NEG	POS	POS	NEG	NEG	NEG	NEG	NEG	NEG	NEG	AMB
**CC20-MSSA**																	
2012_240_Z3526 (2354/12)	POS	AMB	POS	AMB	POS	AMB	POS	POS	POS	NEG	NEG	NEG	NEG	POS	NEG	NEG	AMB
**CC30-MSSA**																	
2012_155_Z3488 (25905A/11)	NEG	POS	POS	AMB	POS	POS	AMB	POS	POS	NEG	NEG	NEG	NEG	NEG	NEG	POS	NEG
**CC30-MSSA [PVL+]**																	
2012_164_Z3447 (26574/11)	AMB	POS	POS	AMB	POS	POS	AMB	POS	POS	NEG	NEG	NEG	NEG	NEG	NEG	POS	POS
2012_166_Z3493 (26988A/11)	AMB	POS	POS	AMB	POS	POS	AMB	POS	POS	NEG	NEG	NEG	NEG	NEG	NEG	POS	NEG
2012_178_Z3500 (197/12)	NEG	POS	POS	AMB	POS	POS	NEG	POS	POS	NEG	NEG	NEG	NEG	NEG	NEG	POS	NEG
2012_183_Z3454	NEG	POS	POS	AMB	POS	POS	AMB	POS	POS	NEG	NEG	NEG	NEG	NEG	NEG	POS	AMB
2012_186_Z3503 (444/12)	NEG	POS	POS	AMB	POS	POS	NEG	POS	POS	NEG	NEG	NEG	NEG	NEG	NEG	POS	NEG
2012_189_Z3506 (542/12)	NEG	POS	POS	AMB	POS	POS	NEG	POS	POS	NEG	NEG	NEG	NEG	NEG	NEG	POS	AMB
2012_192_Z3457 (606/12)	NEG	POS	POS	AMB	POS	POS	NEG	POS	POS	NEG	NEG	NEG	NEG	NEG	NEG	POS	AMB
2012_196_Z3509 (707/12)	NEG	POS	POS	AMB	POS	POS	NEG	POS	POS	NEG	NEG	NEG	NEG	NEG	NEG	POS	POS
2012_218_Z3476 (1432/12)	NEG	POS	POS	AMB	POS	POS	NEG	POS	POS	NEG	NEG	NEG	NEG	NEG	NEG	POS	NEG
2012_232_Z3520 (1892/12)	AMB	POS	POS	AMB	POS	POS	AMB	POS	POS	NEG	NEG	NEG	NEG	NEG	NEG	POS	NEG
2012_278_Z3565 (4397/12)	AMB	POS	POS	AMB	POS	POS	AMB	POS	POS	NEG	NEG	NEG	NEG	NEG	NEG	POS	NEG
2012_285_Z3572 (4802/12)	NEG	POS	POS	AMB	POS	POS	NEG	POS	POS	NEG	NEG	NEG	NEG	NEG	NEG	POS	NEG
2012_287_Z3574 (4906/12)	NEG	POS	POS	AMB	POS	POS	NEG	POS	POS	NEG	NEG	NEG	NEG	NEG	NEG	POS	NEG
2012_295_Z3582 (5116/12)	NEG	POS	POS	AMB	POS	POS	NEG	POS	POS	NEG	NEG	NEG	NEG	NEG	NEG	POS	NEG
2012_326_Z3613 (7321/12)	NEG	POS	POS	AMB	POS	POS	AMB	POS	POS	NEG	NEG	NEG	NEG	NEG	NEG	POS	AMB
2012_356_Z3643 (1204-15897)	NEG	POS	POS	AMB	POS	POS	NEG	POS	POS	NEG	NEG	NEG	NEG	NEG	NEG	POS	NEG
2012_366_Z3426 (1204-16124)	AMB	POS	POS	AMB	POS	POS	AMB	POS	POS	NEG	NEG	NEG	NEG	NEG	NEG	POS	AMB
2012_372_Z3432 (1205-19821)	NEG	POS	POS	AMB	POS	POS	AMB	POS	POS	NEG	NEG	NEG	NEG	NEG	NEG	POS	AMB
2012_380_Z3440 (4141)	NEG	POS	POS	AMB	POS	POS	AMB	POS	POS	NEG	NEG	NEG	NEG	NEG	NEG	POS	AMB
2013_PA20_Z4003	NEG	POS	AMB	POS	POS	POS	NEG	POS	POS	NEG	NEG	NEG	NEG	NEG	NEG	POS	NEG
2013_PA36_Z4019	NEG	POS	AMB	POS	POS	POS	NEG	POS	POS	NEG	NEG	NEG	NEG	NEG	NEG	AMB	NEG
2013_PA46_Z4029	NEG	POS	AMB	POS	POS	POS	NEG	POS	POS	NEG	NEG	NEG	NEG	NEG	NEG	POS	NEG
2013_PA50_Z4033	NEG	POS	AMB	POS	POS	POS	NEG	POS	POS	NEG	NEG	NEG	NEG	NEG	NEG	POS	NEG
2013_PA63_Z4046	NEG	POS	POS	AMB	POS	POS	NEG	POS	POS	NEG	NEG	NEG	NEG	NEG	NEG	POS	NEG
2013_PA78_Z4061	NEG	POS	AMB	POS	POS	POS	NEG	POS	POS	NEG	NEG	NEG	NEG	NEG	NEG	POS	NEG
2013_PA79_Z4062	NEG	POS	AMB	POS	POS	POS	NEG	POS	POS	NEG	NEG	NEG	NEG	NEG	NEG	POS	NEG
**CC45-MSSA**																	
2012_172_Z3449 (49/12)	AMB	POS	POS	AMB	POS	AMB	POS	POS	POS	NEG	NEG	NEG	NEG	NEG	NEG	POS	NEG
2012_208_Z3468 (1011/12)	NEG	POS	NEG	POS	POS	POS	AMB	POS	POS	NEG	NEG	NEG	NEG	NEG	NEG	POS	NEG
2012_235_Z3522 (2085/12)	AMB	POS	AMB	POS	POS	AMB	POS	POS	POS	NEG	NEG	NEG	NEG	NEG	NEG	POS	NEG
2012_290_Z3577 (4921/12)	NEG	POS	AMB	POS	POS	POS	AMB	POS	POS	NEG	NEG	NEG	NEG	NEG	NEG	POS	NEG
2012_311_Z3598 (6182/12)	NEG	POS	AMB	POS	POS	POS	AMB	POS	POS	NEG	NEG	NEG	NEG	NEG	NEG	POS	NEG
Z3604_TT317 - {DC558C18-2959-4049-B8F3-507365	NEG	POS	AMB	POS	POS	POS	AMB	POS	POS	NEG	NEG	NEG	NEG	NEG	NEG	POS	NEG
2012_363_Z3423 (1112-61058)	AMB	POS	AMB	POS	POS	AMB	POS	POS	POS	NEG	NEG	NEG	NEG	NEG	NEG	POS	NEG
2013_PA19_Z4002	NEG	POS	AMB	POS	POS	POS	AMB	POS	POS	NEG	NEG	NEG	NEG	NEG	NEG	POS	NEG
2013_PA54_Z4037	AMB	POS	AMB	POS	POS	POS	AMB	POS	POS	NEG	NEG	NEG	NEG	NEG	NEG	POS	NEG
2013_PA82_Z4065	NEG	POS	AMB	POS	POS	POS	AMB	POS	POS	NEG	NEG	NEG	NEG	NEG	NEG	POS	NEG
**CC97-MSSA**																	
2012_182_Z3453 (279A/12)	POS	AMB	POS	AMB	POS	AMB	POS	POS	POS	NEG	NEG	NEG	NEG	NEG	NEG	NEG	POS
2012_187_Z3504	POS	AMB	POS	AMB	POS	NEG	POS	POS	POS	NEG	NEG	NEG	NEG	NEG	NEG	NEG	AMB
2012_197_Z3510 (757/12)	POS	AMB	AMB	POS	POS	AMB	POS	POS	POS	NEG	NEG	NEG	NEG	NEG	NEG	NEG	POS
2012_199_Z3516 (785/12)	POS	AMB	POS	AMB	POS	AMB	POS	POS	POS	NEG	NEG	NEG	NEG	NEG	NEG	NEG	POS
2013_PA15_Z3998	AMB	POS	POS	AMB	POS	AMB	POS	POS	POS	NEG	NEG	NEG	NEG	NEG	NEG	NEG	POS
**CC101-MSSA**																	
2012_283_Z3570 (4725/12)	POS	AMB	POS	AMB	POS	POS	AMB	POS	POS	NEG	NEG	NEG	POS	NEG	NEG	NEG	POS
2012_247_Z3531 (2788/12)	POS	AMB	POS	AMB	POS	POS	AMB	POS	POS	NEG	NEG	NEG	POS	NEG	NEG	NEG	POS
2012_279_Z3566 (4506/12)	POS	AMB	POS	AMB	POS	POS	AMB	POS	POS	NEG	NEG	NEG	POS	NEG	NEG	NEG	POS

Strain / Isolates	type I site-specific deoxyribonuclease subunit, 3rd locus				type I site-specific deoxyribonuclease subunit, unknown locus			hypothetical protein, located next to serine protease operon	Unspecific efflux/trans-porter	hypothetical protein	hyaluronate lyase, first / second locus			hyaluronate lyase, second locus				
	hsdS3 (COL+USA300+NCTC8325+MW2+MSSA476+RF122)	hsdS3 (Mu50+N315)	hsdS3 (CC51+MRSA252)	hsdS3 (MRSA252)	hsdSx (CC25)	hsdSx (CC15)	hsdSx (etd)	Q2FXC0	Q2YUB3	Q7A4X2	hysA1 (MRSA252)	hysA1 (MRSA252+RF122) and/or hysA2 (consensus)	hysA1 (MRSA252+RF122) and/or hysA2 (COL+USA300)	hysA2 (all other than MRSA252)	hysA2 (COL+USA300+NCTC8325)	hysA2 (all other than COL+USA300+NCTC8325)	hysA2 (all other than COL+USA300+NCTC8325)	hysA2 (MRSA252)
**ST8-MRSA-IV, USA300**																		
2012_158_Z3491 (26049A/11)	POS	NEG	NEG	NEG	POS	NEG	NEG	POS	AMB	NEG	NEG	POS	POS	POS	POS	NEG	NEG	NEG
2012_165_Z3513 (26656/11)	POS	NEG	NEG	NEG	POS	NEG	NEG	POS	AMB	NEG	NEG	POS	POS	POS	POS	NEG	NEG	NEG
2012_214_Z3473 (1322/12)	POS	NEG	NEG	NEG	POS	NEG	NEG	POS	NEG	NEG	NEG	POS	POS	POS	POS	NEG	NEG	AMB
2012_227_Z3484 (1688/12)	POS	NEG	NEG	NEG	POS	NEG	NEG	POS	POS	NEG	NEG	POS	POS	POS	POS	NEG	NEG	AMB
2012_263_Z3550 (3355/12)	POS	NEG	NEG	NEG	POS	NEG	NEG	POS	POS	NEG	NEG	POS	POS	POS	POS	NEG	NEG	NEG
2012_266_Z3553	POS	NEG	NEG	NEG	POS	NEG	NEG	POS	POS	NEG	NEG	POS	POS	POS	POS	NEG	NEG	NEG
2012_284_Z3571 (4729/12)	POS	NEG	NEG	NEG	POS	NEG	NEG	POS	NEG	NEG	NEG	POS	POS	POS	POS	NEG	NEG	NEG
2012_298_Z3585 (5203/12)	POS	NEG	NEG	NEG	POS	NEG	NEG	POS	AMB	NEG	NEG	POS	POS	POS	POS	NEG	NEG	NEG
2012_304_Z3591 (5818/12)	POS	NEG	NEG	NEG	POS	NEG	NEG	POS	AMB	NEG	NEG	POS	POS	POS	POS	NEG	NEG	NEG
Z3595_TT308 - {EFC74B77-3925-484F-A4CF-A1F7DC	POS	NEG	NEG	NEG	POS	NEG	NEG	POS	NEG	NEG	NEG	POS	POS	POS	POS	NEG	NEG	POS
2012_369_Z3429 (1112-58547)	POS	NEG	NEG	NEG	POS	NEG	NEG	POS	NEG	NEG	NEG	POS	POS	POS	POS	NEG	NEG	NEG
2012_374_Z3434 (1203-13527)	POS	NEG	NEG	NEG	POS	NEG	NEG	POS	AMB	NEG	NEG	POS	POS	POS	POS	NEG	NEG	POS
2013_PA06_Z3989	POS	NEG	NEG	NEG	POS	NEG	NEG	POS	AMB	NEG	NEG	POS	POS	POS	POS	NEG	NEG	AMB
2013_PA55_Z4038	POS	NEG	NEG	NEG	POS	NEG	NEG	POS	#ZAHL!	NEG	NEG	POS	POS	POS	POS	NEG	NEG	NEG
2013_PA71_Z4054	POS	NEG	NEG	NEG	POS	NEG	NEG	POS	NEG	NEG	NEG	POS	POS	POS	POS	NEG	NEG	NEG
**ST8-MRSA-IV [PVL+/ACME+], US**																		
2012_180_Z3501 (232/12)	POS	NEG	NEG	NEG	POS	NEG	NEG	POS	NEG	NEG	NEG	POS	POS	POS	POS	NEG	NEG	NEG
2013_PA68_Z4051	POS	NEG	NEG	NEG	POS	NEG	NEG	POS	AMB	NEG	NEG	POS	POS	POS	POS	NEG	NEG	NEG
**ST72-MSSA**																		
2012_174_Z3451 (102/12)	NEG	NEG	POS	NEG	POS	NEG	NEG	NEG	AMB	POS	NEG	POS	NEG	POS	NEG	POS	POS	AMB
2012_223_Z3480 (1506A/12)	NEG	NEG	POS	NEG	POS	NEG	NEG	NEG	AMB	POS	NEG	POS	NEG	POS	NEG	POS	POS	AMB
2012_236_Z3523 (2086/12)	NEG	NEG	POS	NEG	POS	NEG	NEG	NEG	AMB	POS	NEG	POS	NEG	POS	NEG	POS	POS	AMB
2012_239_Z3525 (2346/12)	NEG	NEG	POS	NEG	POS	AMB	NEG	NEG	AMB	POS	NEG	POS	NEG	POS	NEG	POS	POS	AMB
2012_252_Z3535 (3030/12)	NEG	NEG	POS	NEG	POS	NEG	NEG	NEG	POS	POS	NEG	POS	NEG	POS	NEG	POS	POS	AMB
2012_271_Z3558 (3799/12)	NEG	NEG	POS	NEG	POS	NEG	NEG	NEG	POS	POS	NEG	POS	NEG	POS	NEG	POS	POS	AMB
2012_276_Z3563	NEG	NEG	POS	NEG	POS	NEG	NEG	NEG	AMB	POS	NEG	POS	NEG	POS	NEG	POS	POS	NEG
2012_300_Z3587 (5638/12)	NEG	NEG	POS	NEG	POS	NEG	NEG	NEG	AMB	POS	NEG	POS	NEG	POS	NEG	POS	POS	NEG
2012_316_Z3603 (6941/12)	NEG	NEG	POS	NEG	POS	NEG	NEG	NEG	AMB	POS	NEG	POS	NEG	POS	NEG	POS	POS	NEG
2012_319_Z3606 (7019/12)	NEG	NEG	POS	NEG	POS	NEG	NEG	NEG	AMB	POS	NEG	POS	NEG	POS	NEG	POS	POS	NEG
2012_371_Z3431 (1204-17577)	NEG	NEG	POS	NEG	POS	AMB	NEG	NEG	AMB	POS	NEG	POS	NEG	POS	NEG	POS	POS	NEG
2013_PA05_Z3988	NEG	NEG	POS	NEG	POS	NEG	NEG	NEG	AMB	POS	NEG	POS	NEG	POS	NEG	POS	POS	AMB
2013_PA65_Z4048	NEG	NEG	POS	NEG	POS	NEG	NEG	NEG	NEG	POS	NEG	POS	NEG	POS	NEG	POS	POS	NEG
2013_PA73_Z4056	NEG	NEG	POS	NEG	POS	NEG	NEG	NEG	AMB	POS	NEG	POS	NEG	POS	NEG	POS	POS	NEG
**ST72-MSSA-SCCfus**																		
2013_PA16_Z3999	NEG	NEG	POS	NEG	POS	NEG	NEG	NEG	NEG	POS	NEG	POS	NEG	POS	NEG	POS	POS	AMB
**ST72-MSSA-SCCfus/kdp**																		
2012_361_Z3421 (1201-1299)	NEG	NEG	POS	NEG	POS	NEG	NEG	NEG	AMB	POS	NEG	POS	NEG	POS	NEG	POS	POS	POS
**ST239-MRSA-III&SCCmer**																		
2012_157_Z3490	POS	NEG	NEG	NEG	POS	NEG	NEG	POS	AMB	NEG	NEG	POS	POS	POS	POS	NEG	NEG	NEG
2012_168_Z3494 (27129/11)	POS	NEG	NEG	NEG	POS	NEG	NEG	POS	NEG	NEG	NEG	POS	POS	POS	POS	NEG	NEG	NEG
2012_195_Z3508 (619/12)	POS	NEG	NEG	NEG	POS	NEG	NEG	POS	AMB	NEG	NEG	POS	POS	POS	POS	NEG	NEG	NEG
2012_222_Z3479 (1489/12)	POS	NEG	NEG	NEG	POS	NEG	NEG	POS	NEG	NEG	NEG	POS	POS	POS	POS	NEG	NEG	AMB
2012_233_Z3540 (1943/12)	POS	NEG	NEG	NEG	POS	NEG	NEG	POS	AMB	NEG	NEG	POS	POS	POS	POS	NEG	NEG	NEG
2012_237_Z3541	POS	NEG	NEG	NEG	POS	NEG	NEG	POS	AMB	NEG	NEG	POS	POS	POS	POS	NEG	NEG	NEG
2012_262_Z3549	POS	NEG	NEG	NEG	POS	NEG	NEG	POS	POS	NEG	NEG	POS	POS	POS	POS	NEG	NEG	NEG
2012_275_Z3562 (3893/12)	POS	NEG	NEG	NEG	POS	NEG	NEG	POS	POS	NEG	NEG	POS	POS	POS	POS	NEG	NEG	NEG
2012_291_Z3578	POS	NEG	NEG	NEG	POS	NEG	NEG	POS	AMB	NEG	NEG	POS	POS	POS	POS	NEG	NEG	NEG
2012_314_Z3601 (6939/12)	POS	NEG	NEG	NEG	POS	NEG	NEG	POS	AMB	NEG	NEG	POS	POS	POS	POS	NEG	NEG	AMB
2012323_Z3610	POS	NEG	NEG	NEG	POS	NEG	NEG	POS	AMB	NEG	NEG	POS	POS	POS	POS	NEG	NEG	NEG
2012330_Z3617 (7605/12)	POS	NEG	NEG	NEG	POS	NEG	NEG	POS	AMB	NEG	NEG	POS	POS	POS	POS	NEG	NEG	NEG
2012332_Z3619 (8076/12)	POS	NEG	NEG	NEG	POS	NEG	NEG	POS	AMB	NEG	NEG	POS	POS	POS	POS	NEG	NEG	NEG
2012339_Z3626 (8558/12)	POS	NEG	NEG	NEG	POS	NEG	NEG	POS	AMB	NEG	NEG	POS	POS	POS	POS	NEG	NEG	NEG
2012340_Z3627 (8523/12)	POS	NEG	NEG	NEG	POS	NEG	NEG	POS	AMB	NEG	NEG	POS	POS	POS	POS	NEG	NEG	NEG
2012348_Z3635	POS	NEG	NEG	NEG	POS	NEG	NEG	POS	AMB	NEG	NEG	POS	POS	POS	POS	NEG	NEG	NEG
2012350_Z3637	POS	NEG	NEG	NEG	POS	NEG	NEG	POS	AMB	NEG	NEG	POS	POS	POS	POS	NEG	NEG	AMB
2012_370_Z3430 (1112-52177)	POS	NEG	NEG	NEG	POS	NEG	NEG	POS	AMB	NEG	NEG	POS	POS	POS	POS	NEG	NEG	NEG
2012_378_Z3438 (4139)	POS	NEG	NEG	NEG	POS	NEG	NEG	POS	AMB	NEG	NEG	POS	POS	POS	POS	NEG	NEG	AMB
2013_PA21_Z4004	POS	NEG	NEG	NEG	POS	NEG	NEG	POS	NEG	NEG	NEG	POS	POS	POS	POS	NEG	NEG	NEG
2013_PA22_Z4005	POS	NEG	NEG	NEG	POS	NEG	NEG	POS	POS	NEG	NEG	POS	POS	POS	POS	NEG	NEG	AMB
2013_PA24_Z4007	POS	NEG	NEG	NEG	POS	NEG	NEG	POS	NEG	NEG	NEG	POS	POS	POS	POS	NEG	NEG	NEG
2013_PA31_Z4014	POS	NEG	NEG	NEG	POS	NEG	NEG	POS	NEG	NEG	NEG	POS	POS	POS	POS	NEG	NEG	AMB
2013_PA35_Z4018	POS	NEG	NEG	NEG	POS	NEG	NEG	POS	NEG	NEG	NEG	POS	POS	POS	POS	NEG	NEG	NEG
2013_PA81_Z4064	POS	NEG	NEG	NEG	POS	NEG	NEG	POS	NEG	NEG	NEG	POS	POS	POS	POS	NEG	NEG	NEG
**ST239-MRSA-III+ccrA/B4**																		
2012_254_Z3537 (3124A/12)	POS	NEG	NEG	NEG	POS	NEG	NEG	POS	NEG	NEG	NEG	POS	POS	POS	POS	NEG	NEG	NEG
2013_PA25_Z4008	POS	NEG	NEG	NEG	POS	NEG	NEG	POS	NEG	NEG	NEG	POS	POS	POS	POS	NEG	NEG	AMB
**ST834-MSSA**																		
2012_268_Z3555 (3693/12)	NEG	NEG	NEG	NEG	NEG	NEG	POS	NEG	NEG	NEG	NEG	POS	NEG	NEG	NEG	POS	POS	NEG
**CC12-MSSA**																		
2012_210_Z3470 (1177/12)	NEG	NEG	POS	NEG	POS	NEG	NEG	NEG	NEG	NEG	NEG	POS	NEG	NEG	NEG	POS	POS	NEG
2012_230_Z3487 (1797/12)	NEG	NEG	POS	NEG	POS	AMB	NEG	NEG	POS	NEG	NEG	POS	NEG	NEG	NEG	POS	POS	NEG
2013_PA18_Z4001	NEG	NEG	POS	NEG	POS	AMB	NEG	NEG	AMB	NEG	NEG	POS	NEG	NEG	NEG	POS	POS	NEG
**CC15-MSSA**																		
2012_160_Z3444 (26386/11)	NEG	NEG	NEG	NEG	POS	POS	NEG	NEG	AMB	NEG	NEG	POS	POS	POS	POS	POS	POS	NEG
2012_167_Z3514 (27010/11)	NEG	NEG	NEG	NEG	POS	POS	NEG	NEG	AMB	NEG	NEG	POS	POS	POS	POS	POS	POS	NEG
2012_184_Z3455 (376/12)	NEG	NEG	NEG	NEG	POS	POS	NEG	NEG	AMB	NEG	NEG	POS	POS	POS	POS	POS	POS	NEG
2012_205_Z3465 (939/12)	NEG	NEG	NEG	NEG	POS	POS	NEG	NEG	POS	NEG	NEG	POS	POS	POS	POS	POS	POS	AMB
2012_231_Z3519 (1831/12)	NEG	NEG	NEG	NEG	POS	POS	NEG	NEG	NEG	NEG	NEG	POS	POS	POS	POS	POS	POS	NEG
2012_249_Z3533 (2907/12)	NEG	NEG	NEG	NEG	POS	POS	NEG	NEG	AMB	NEG	NEG	POS	POS	POS	POS	POS	POS	AMB
2012_305_Z3592 (5839/12)	NEG	NEG	NEG	NEG	POS	POS	NEG	NEG	AMB	NEG	NEG	POS	POS	POS	POS	POS	POS	AMB
2012_313_Z3600 (6857/12)	NEG	NEG	NEG	NEG	POS	POS	NEG	NEG	AMB	NEG	NEG	POS	POS	POS	POS	POS	POS	NEG
2012_345_Z3632 (7966B/12)	NEG	NEG	NEG	NEG	POS	POS	NEG	NEG	AMB	NEG	NEG	POS	POS	POS	POS	POS	POS	NEG
2013_PA33_Z4016	NEG	NEG	NEG	NEG	POS	POS	NEG	NEG	NEG	NEG	NEG	POS	POS	POS	POS	POS	POS	NEG
**CC20-MSSA**																		
2012_240_Z3526 (2354/12)	NEG	NEG	NEG	NEG	AMB	AMB	NEG	NEG	AMB	POS	NEG	POS	NEG	AMB	NEG	POS	POS	NEG
**CC30-MSSA**																		
2012_155_Z3488 (25905A/11)	NEG	NEG	POS	POS	POS	NEG	NEG	NEG	AMB	POS	POS	POS	POS	NEG	POS	POS	POS	POS
**CC30-MSSA [PVL+]**																		
2012_164_Z3447 (26574/11)	NEG	NEG	POS	POS	POS	NEG	NEG	NEG	POS	POS	POS	POS	POS	NEG	POS	POS	POS	POS
2012_166_Z3493 (26988A/11)	NEG	NEG	POS	POS	POS	AMB	NEG	NEG	AMB	POS	POS	POS	POS	NEG	POS	POS	POS	POS
2012_178_Z3500 (197/12)	NEG	NEG	POS	POS	POS	NEG	NEG	NEG	NEG	POS	POS	POS	POS	NEG	POS	POS	POS	POS
2012_183_Z3454	NEG	NEG	POS	POS	POS	NEG	NEG	NEG	AMB	POS	POS	POS	POS	NEG	POS	POS	POS	POS
2012_186_Z3503 (444/12)	NEG	NEG	POS	POS	POS	NEG	NEG	NEG	NEG	POS	POS	POS	POS	NEG	POS	POS	POS	POS
2012_189_Z3506 (542/12)	NEG	NEG	POS	POS	POS	NEG	NEG	NEG	NEG	POS	POS	POS	POS	NEG	POS	POS	POS	POS
2012_192_Z3457 (606/12)	NEG	NEG	POS	POS	POS	NEG	NEG	NEG	AMB	POS	POS	POS	POS	NEG	POS	POS	POS	POS
2012_196_Z3509 (707/12)	NEG	NEG	POS	POS	POS	AMB	NEG	NEG	AMB	POS	POS	POS	POS	NEG	POS	POS	POS	POS
2012_218_Z3476 (1432/12)	NEG	NEG	POS	POS	POS	NEG	NEG	NEG	NEG	POS	POS	POS	POS	NEG	POS	POS	POS	POS
2012_232_Z3520 (1892/12)	NEG	NEG	POS	POS	POS	AMB	NEG	NEG	AMB	POS	POS	POS	POS	NEG	POS	POS	POS	POS
2012_278_Z3565 (4397/12)	NEG	NEG	POS	POS	POS	NEG	NEG	NEG	POS	POS	POS	POS	POS	NEG	POS	POS	POS	POS
2012_285_Z3572 (4802/12)	NEG	NEG	POS	POS	POS	NEG	NEG	NEG	AMB	POS	POS	POS	POS	NEG	POS	POS	POS	POS
2012_287_Z3574 (4906/12)	NEG	NEG	POS	POS	POS	NEG	NEG	NEG	AMB	POS	POS	POS	POS	NEG	POS	POS	POS	POS
2012_295_Z3582 (5116/12)	NEG	NEG	POS	POS	POS	NEG	NEG	NEG	AMB	POS	POS	POS	POS	NEG	POS	POS	POS	POS
2012_326_Z3613 (7321/12)	NEG	NEG	POS	POS	POS	AMB	NEG	NEG	AMB	POS	POS	POS	POS	NEG	POS	POS	POS	POS
2012_356_Z3643 (1204-15897)	NEG	NEG	POS	POS	POS	NEG	NEG	NEG	NEG	POS	POS	POS	POS	NEG	POS	POS	POS	POS
2012_366_Z3426 (1204-16124)	NEG	NEG	POS	POS	POS	AMB	NEG	NEG	AMB	POS	POS	POS	POS	NEG	POS	POS	POS	POS
2012_372_Z3432 (1205-19821)	NEG	NEG	POS	POS	POS	NEG	NEG	NEG	AMB	POS	POS	POS	POS	NEG	POS	POS	POS	POS
2012_380_Z3440 (4141)	NEG	NEG	POS	POS	POS	NEG	NEG	NEG	AMB	POS	POS	POS	POS	NEG	POS	POS	POS	POS
2013_PA20_Z4003	NEG	NEG	POS	POS	POS	NEG	NEG	NEG	NEG	POS	POS	POS	POS	NEG	POS	POS	POS	POS
2013_PA36_Z4019	NEG	NEG	POS	AMB	NEG	NEG	NEG	NEG	NEG	POS	POS	POS	POS	NEG	POS	POS	AMB	POS
2013_PA46_Z4029	NEG	NEG	POS	POS	AMB	NEG	NEG	NEG	NEG	POS	POS	POS	POS	NEG	POS	POS	POS	POS
2013_PA50_Z4033	NEG	NEG	POS	POS	AMB	NEG	NEG	NEG	NEG	POS	POS	POS	POS	NEG	POS	POS	POS	POS
2013_PA63_Z4046	NEG	NEG	POS	POS	POS	NEG	NEG	NEG	NEG	POS	POS	POS	POS	NEG	POS	POS	POS	POS
2013_PA78_Z4061	NEG	NEG	POS	POS	POS	NEG	NEG	NEG	NEG	POS	POS	POS	POS	NEG	POS	POS	POS	POS
2013_PA79_Z4062	NEG	NEG	POS	POS	POS	NEG	NEG	NEG	NEG	POS	POS	POS	POS	NEG	POS	POS	POS	POS
**CC45-MSSA**																		
2012_172_Z3449 (49/12)	NEG	NEG	NEG	NEG	POS	NEG	NEG	NEG	AMB	POS	NEG	POS	NEG	POS	NEG	POS	NEG	NEG
2012_208_Z3468 (1011/12)	NEG	NEG	NEG	NEG	POS	NEG	NEG	NEG	NEG	POS	NEG	POS	NEG	POS	NEG	POS	NEG	NEG
2012_235_Z3522 (2085/12)	NEG	NEG	NEG	NEG	POS	AMB	NEG	NEG	AMB	POS	NEG	POS	NEG	POS	NEG	POS	NEG	NEG
2012_290_Z3577 (4921/12)	NEG	NEG	NEG	NEG	POS	NEG	NEG	NEG	NEG	POS	NEG	POS	NEG	POS	NEG	POS	NEG	NEG
2012_311_Z3598 (6182/12)	NEG	NEG	NEG	NEG	POS	NEG	NEG	NEG	NEG	POS	NEG	POS	NEG	AMB	NEG	POS	NEG	NEG
Z3604_TT317 - {DC558C18-2959-4049-B8F3-507365	NEG	NEG	NEG	NEG	POS	NEG	NEG	NEG	NEG	POS	NEG	POS	NEG	NEG	NEG	POS	NEG	NEG
2012_363_Z3423 (1112-61058)	NEG	NEG	NEG	NEG	POS	AMB	NEG	NEG	AMB	POS	NEG	POS	NEG	AMB	NEG	POS	NEG	NEG
2013_PA19_Z4002	NEG	NEG	NEG	NEG	POS	NEG	NEG	NEG	NEG	POS	NEG	POS	NEG	AMB	NEG	POS	NEG	NEG
2013_PA54_Z4037	NEG	NEG	NEG	NEG	POS	AMB	NEG	NEG	AMB	POS	NEG	POS	NEG	POS	NEG	POS	NEG	NEG
2013_PA82_Z4065	NEG	NEG	NEG	NEG	AMB	NEG	NEG	NEG	NEG	POS	NEG	POS	NEG	NEG	NEG	POS	NEG	NEG
**CC97-MSSA**																		
2012_182_Z3453 (279A/12)	NEG	NEG	NEG	NEG	POS	NEG	NEG	NEG	AMB	POS	NEG	POS	NEG	NEG	NEG	POS	POS	NEG
2012_187_Z3504	NEG	NEG	NEG	NEG	POS	NEG	NEG	NEG	AMB	POS	NEG	POS	NEG	NEG	NEG	POS	POS	NEG
2012_197_Z3510 (757/12)	NEG	NEG	NEG	NEG	POS	NEG	NEG	NEG	AMB	POS	NEG	POS	NEG	NEG	NEG	POS	POS	NEG
2012_199_Z3516 (785/12)	NEG	NEG	NEG	NEG	POS	AMB	NEG	NEG	AMB	POS	NEG	POS	NEG	AMB	NEG	POS	POS	AMB
2013_PA15_Z3998	NEG	NEG	NEG	NEG	POS	AMB	NEG	NEG	AMB	POS	NEG	POS	NEG	AMB	NEG	POS	POS	NEG
**CC101-MSSA**																		
2012_283_Z3570 (4725/12)	NEG	NEG	NEG	NEG	POS	NEG	POS	POS	AMB	NEG	POS	POS	POS	NEG	POS	POS	POS	NEG
2012_247_Z3531 (2788/12)	NEG	NEG	NEG	NEG	POS	NEG	POS	POS	AMB	NEG	POS	POS	POS	NEG	POS	POS	POS	NEG
2012_279_Z3566 (4506/12)	NEG	NEG	NEG	NEG	POS	NEG	POS	POS	POS	NEG	POS	POS	POS	NEG	POS	POS	POS	NEG

Strain / Isolates	immunodominant antigen B		defensin resistance protein		transferrin-binding protein			hypothetical protein, similar to integral membrane protein LmrP				type I site-specific deoxyribo-nuclease subunit, 1st locus	type I site-specific deoxyribonuclease subunit, 2nd locus				type I site-specific deoxyribonuclease subunit, 3rd locus					type I site-specific deoxyribonuclease subunit, unknown locus			hypothetical protein, located next to serine protease operon	Unspecific efflux/trans-porter	hypothetical protein	hyaluronate lyase, first / second locus			hyaluronate lyase, second locus				
	isaB	isaB (MRSA252)	mprF (COL+MW2)	mprF (Mu50+MRSA252)	isdA (consensus)	isdA (MRSA252)	isdA (other than MRSA252)	lmrP (other than RF122)_probe1	lmrP (other than RF122)_probe2	lmrP (RF122)_probe1	lmrP (RF122)_probe2	hsdS1 (RF122)	hsdS2 (Mu50+N315+COL+USA300+NCTC8325	hsdS2 (MW2+MSSA476)	hsdS2 (RF122)	hsdS2 (MRSA252)	hsdS3 (all other than RF122+ MRSA252)	hsdS3 (COL+USA300+ NCTC8325+MW2+MSSA476+ RF122)	hsdS3 (Mu50+N315)	hsdS3 (CC51+ MRSA252)	hsdS3 (MRSA252)	hsdSx (CC25)	hsdSx (CC15)	hsdSx (etd)	Q2FXC0	Q2YUB3	Q7A4X2	hysA1 (MRSA252)	hysA1 (MRSA252+RF122) and/or hysA2 (consensus)	hysA1 (MRSA252+RF122) and/or hysA2 (COL+USA300)	hysA2 (all other than MRSA252)	hysA2 (COL+USA300+ NCTC8325)	hysA2 (all other than COL+USA300+ NCTC8325)	hysA2 (all other than COL+USA300+ NCTC8325)	hysA2 (MRSA252)
**CC121-MSSA**																																			
2012_163_Z3446 (26495/11)	POS	AMB	POS	AMB	POS	AMB	POS	NEG	NEG	POS	POS	NEG	POS	NEG	NEG	NEG	AMB	NEG	NEG	POS	NEG	POS	AMB	NEG	NEG	AMB	POS	NEG	POS	POS	POS	POS	NEG	NEG	NEG
2012_204_Z3464 (938/12)	POS	AMB	POS	AMB	POS	AMB	POS	NEG	NEG	POS	POS	NEG	POS	NEG	NEG	NEG	AMB	NEG	NEG	POS	NEG	POS	NEG	NEG	NEG	POS	POS	NEG	POS	POS	POS	POS	NEG	NEG	NEG
2012_225_Z3482 (1563/12)	POS	AMB	POS	AMB	POS	AMB	POS	NEG	AMB	POS	POS	NEG	POS	NEG	NEG	NEG	AMB	NEG	NEG	POS	NEG	POS	NEG	NEG	NEG	POS	POS	NEG	POS	POS	POS	POS	NEG	NEG	NEG
2012_228_Z3485 (1700B/12)	POS	AMB	POS	AMB	POS	AMB	POS	NEG	NEG	POS	POS	NEG	POS	NEG	NEG	NEG	NEG	NEG	NEG	POS	NEG	POS	NEG	NEG	NEG	POS	POS	NEG	POS	POS	POS	POS	NEG	NEG	NEG
2012_229_Z3486 (1737/12)	POS	AMB	POS	AMB	POS	AMB	POS	NEG	NEG	POS	POS	NEG	POS	NEG	NEG	NEG	NEG	NEG	NEG	POS	NEG	POS	AMB	NEG	NEG	POS	POS	NEG	POS	POS	POS	POS	NEG	NEG	NEG
2012_302_Z3589 (5795/12)	POS	AMB	AMB	POS	POS	NEG	POS	NEG	NEG	POS	POS	NEG	POS	NEG	NEG	NEG	NEG	NEG	NEG	POS	NEG	POS	NEG	NEG	NEG	AMB	POS	NEG	POS	POS	NEG	POS	NEG	NEG	NEG
2012_359_Z3419	AMB	POS	POS	AMB	POS	AMB	POS	NEG	NEG	POS	POS	NEG	POS	NEG	NEG	NEG	NEG	NEG	NEG	POS	NEG	POS	AMB	NEG	NEG	NEG	POS	NEG	POS	POS	AMB	POS	NEG	NEG	NEG
2012_362_Z3422 (1201-996)	POS	AMB	POS	AMB	POS	AMB	POS	NEG	NEG	POS	POS	NEG	POS	NEG	NEG	NEG	AMB	NEG	NEG	POS	NEG	POS	NEG	NEG	NEG	AMB	POS	NEG	POS	POS	AMB	POS	NEG	NEG	NEG
2012_367_Z3427 (1204-16413)	POS	AMB	POS	AMB	POS	AMB	POS	NEG	NEG	POS	POS	NEG	POS	NEG	NEG	NEG	AMB	NEG	NEG	POS	NEG	POS	NEG	NEG	NEG	AMB	POS	NEG	POS	POS	AMB	POS	NEG	NEG	NEG
2013_PA09_Z3992	POS	AMB	POS	AMB	POS	AMB	POS	NEG	NEG	POS	POS	NEG	POS	NEG	NEG	NEG	NEG	NEG	NEG	POS	NEG	POS	NEG	NEG	NEG	NEG	POS	NEG	POS	POS	NEG	POS	NEG	NEG	NEG
2013_PA59_Z4042	POS	AMB	POS	AMB	POS	AMB	POS	NEG	NEG	POS	POS	NEG	POS	NEG	NEG	NEG	NEG	NEG	NEG	POS	NEG	POS	NEG	NEG	NEG	NEG	POS	NEG	POS	POS	NEG	POS	NEG	NEG	NEG
2013_PA60_Z4043	POS	AMB	POS	AMB	POS	NEG	POS	NEG	NEG	POS	POS	NEG	POS	NEG	NEG	NEG	NEG	NEG	NEG	POS	NEG	POS	NEG	NEG	NEG	NEG	POS	NEG	POS	POS	NEG	POS	NEG	NEG	NEG
**CC152-MSSA [PVL+]**																																			
2012_190_Z3507 (549/12)	POS	AMB	POS	NEG	POS	POS	NEG	NEG	NEG	POS	POS	NEG	POS	NEG	NEG	NEG	POS	NEG	NEG	POS	POS	POS	NEG	POS	NEG	NEG	NEG	NEG	POS	POS	NEG	POS	NEG	NEG	POS
2012_201_Z3461 (823/12)	POS	AMB	POS	NEG	POS	POS	NEG	NEG	NEG	POS	POS	NEG	POS	NEG	NEG	NEG	POS	NEG	NEG	POS	POS	POS	AMB	POS	NEG	NEG	NEG	NEG	POS	POS	NEG	POS	NEG	NEG	POS
2012_209_Z3469 (1116/12)	AMB	POS	POS	NEG	POS	NEG	NEG	NEG	NEG	POS	POS	NEG	POS	NEG	NEG	NEG	POS	NEG	NEG	POS	POS	POS	NEG	POS	NEG	NEG	NEG	NEG	POS	POS	NEG	POS	NEG	NEG	POS
2012_211_Z3517 (1238/12)	AMB	POS	POS	NEG	POS	NEG	NEG	NEG	NEG	POS	POS	NEG	POS	NEG	NEG	NEG	POS	NEG	NEG	POS	POS	POS	AMB	POS	NEG	NEG	NEG	NEG	POS	POS	NEG	POS	NEG	NEG	POS
2012_213_Z3472 (1320/12)	AMB	POS	POS	NEG	POS	NEG	NEG	NEG	NEG	POS	POS	NEG	POS	NEG	NEG	NEG	POS	NEG	NEG	POS	POS	POS	NEG	POS	NEG	NEG	NEG	NEG	POS	POS	NEG	POS	NEG	NEG	POS
2012_221_Z3478	AMB	POS	POS	NEG	POS	NEG	NEG	NEG	NEG	POS	POS	NEG	POS	NEG	NEG	NEG	POS	NEG	NEG	POS	POS	POS	NEG	POS	NEG	NEG	NEG	NEG	POS	POS	NEG	POS	NEG	NEG	POS
2012_234_Z3521 (1981/12)	POS	AMB	POS	NEG	POS	POS	NEG	NEG	AMB	POS	POS	NEG	POS	NEG	NEG	NEG	POS	NEG	NEG	POS	POS	POS	AMB	POS	NEG	AMB	NEG	NEG	POS	POS	NEG	POS	NEG	NEG	POS
2012_238_Z3524 (2199/12)	POS	AMB	POS	NEG	POS	NEG	NEG	NEG	NEG	POS	POS	NEG	POS	NEG	NEG	NEG	POS	NEG	NEG	POS	POS	POS	NEG	POS	NEG	AMB	NEG	NEG	POS	POS	NEG	POS	NEG	NEG	POS
2012_260_Z3547 (3290/12)	POS	AMB	POS	AMB	POS	POS	NEG	NEG	AMB	POS	POS	NEG	POS	NEG	NEG	NEG	POS	NEG	NEG	POS	POS	POS	NEG	POS	NEG	POS	NEG	NEG	POS	POS	NEG	POS	NEG	NEG	POS
2012_272_Z3559 (3802/12)	POS	AMB	POS	NEG	POS	POS	NEG	NEG	AMB	POS	POS	NEG	POS	NEG	NEG	NEG	AMB	NEG	NEG	POS	POS	POS	AMB	NEG	NEG	AMB	NEG	NEG	POS	POS	NEG	POS	NEG	NEG	POS
2012_289_Z3576 (4912/12)	POS	AMB	POS	NEG	POS	NEG	NEG	NEG	NEG	POS	POS	NEG	POS	NEG	NEG	NEG	POS	NEG	NEG	POS	POS	POS	NEG	POS	NEG	NEG	NEG	NEG	POS	POS	NEG	POS	NEG	NEG	POS
2012_309_Z3596	POS	AMB	POS	NEG	POS	NEG	NEG	NEG	NEG	POS	POS	NEG	POS	NEG	NEG	NEG	POS	NEG	NEG	POS	POS	POS	NEG	POS	NEG	AMB	NEG	NEG	POS	POS	NEG	POS	NEG	NEG	POS
2012_322_Z3609 (7178/12)	AMB	POS	POS	NEG	POS	NEG	NEG	NEG	NEG	POS	POS	NEG	POS	NEG	NEG	NEG	AMB	NEG	NEG	POS	POS	AMB	NEG	POS	NEG	NEG	NEG	NEG	POS	POS	NEG	POS	NEG	NEG	POS
2012_325_Z3612 (7254/12)	POS	AMB	POS	NEG	POS	POS	NEG	NEG	NEG	POS	POS	NEG	POS	NEG	NEG	NEG	POS	NEG	NEG	POS	POS	POS	AMB	NEG	NEG	AMB	NEG	NEG	POS	POS	NEG	POS	NEG	NEG	POS
2012_328_Z3615 (7456/12)	POS	AMB	POS	NEG	POS	POS	NEG	NEG	NEG	POS	POS	NEG	POS	NEG	NEG	NEG	POS	NEG	NEG	POS	POS	POS	AMB	POS	NEG	AMB	NEG	NEG	POS	POS	NEG	POS	NEG	NEG	POS
2012_334_Z3621 (8267/12)	POS	AMB	POS	NEG	POS	POS	NEG	NEG	NEG	POS	POS	NEG	POS	NEG	NEG	NEG	POS	NEG	NEG	POS	POS	POS	NEG	POS	NEG	AMB	NEG	NEG	POS	POS	NEG	POS	NEG	NEG	POS
2012_360_Z3420 (1201-4047)	POS	AMB	POS	NEG	POS	POS	NEG	NEG	AMB	POS	POS	NEG	POS	NEG	NEG	NEG	POS	NEG	NEG	POS	POS	POS	AMB	POS	NEG	AMB	NEG	NEG	POS	POS	NEG	POS	NEG	NEG	POS
2012_382_Z3442 (4086)	POS	AMB	POS	NEG	POS	POS	NEG	NEG	NEG	POS	POS	NEG	POS	AMB	NEG	NEG	POS	NEG	NEG	POS	POS	POS	NEG	POS	NEG	NEG	NEG	NEG	POS	POS	NEG	POS	NEG	NEG	POS
2013_PA02_Z3985	AMB	POS	POS	NEG	POS	NEG	NEG	NEG	NEG	POS	POS	NEG	POS	AMB	NEG	NEG	POS	NEG	NEG	POS	POS	POS	NEG	POS	NEG	NEG	NEG	NEG	POS	POS	NEG	POS	NEG	NEG	POS
2013_PA03_Z3986	AMB	POS	POS	NEG	POS	NEG	NEG	NEG	NEG	POS	POS	NEG	POS	NEG	NEG	NEG	AMB	NEG	NEG	POS	POS	POS	NEG	POS	NEG	NEG	NEG	NEG	POS	POS	NEG	POS	NEG	NEG	POS
2013_PA07_Z3990	AMB	POS	POS	NEG	POS	NEG	NEG	NEG	NEG	POS	POS	NEG	POS	NEG	NEG	NEG	POS	NEG	NEG	POS	POS	POS	AMB	POS	NEG	NEG	NEG	NEG	POS	POS	NEG	POS	NEG	NEG	POS
2013_PA34_Z4017	POS	AMB	POS	NEG	POS	NEG	NEG	NEG	NEG	POS	POS	NEG	POS	NEG	NEG	NEG	AMB	NEG	NEG	POS	POS	POS	NEG	POS	NEG	NEG	NEG	NEG	POS	AMB	NEG	POS	NEG	NEG	AMB
2013_PA40_Z4023	POS	AMB	POS	NEG	POS	NEG	NEG	NEG	NEG	POS	POS	NEG	POS	NEG	NEG	NEG	AMB	NEG	NEG	POS	POS	AMB	NEG	POS	NEG	NEG	NEG	NEG	POS	AMB	NEG	POS	NEG	NEG	AMB
2013_PA48_Z4031	AMB	POS	POS	NEG	POS	NEG	NEG	NEG	NEG	POS	POS	NEG	POS	NEG	NEG	NEG	POS	NEG	NEG	POS	POS	POS	NEG	POS	NEG	NEG	NEG	NEG	POS	POS	NEG	POS	NEG	NEG	POS
2013_PA49_Z4032	POS	AMB	POS	NEG	POS	NEG	NEG	NEG	NEG	POS	POS	NEG	POS	NEG	NEG	NEG	NEG	NEG	NEG	POS	POS	AMB	NEG	POS	NEG	NEG	NEG	NEG	POS	NEG	NEG	POS	NEG	NEG	NEG
2013_PA51_Z4034	AMB	POS	POS	NEG	POS	NEG	NEG	NEG	NEG	POS	POS	NEG	POS	AMB	NEG	NEG	POS	NEG	NEG	POS	POS	POS	NEG	POS	NEG	NEG	NEG	NEG	POS	POS	NEG	POS	NEG	NEG	POS
2013_PA70_Z4053	AMB	POS	POS	NEG	POS	NEG	NEG	NEG	NEG	POS	POS	NEG	POS	NEG	NEG	NEG	AMB	NEG	NEG	POS	POS	POS	NEG	POS	NEG	NEG	NEG	NEG	POS	AMB	NEG	POS	NEG	NEG	POS
2013_PA84_Z4067	AMB	POS	POS	NEG	POS	NEG	NEG	NEG	NEG	POS	POS	NEG	POS	NEG	NEG	NEG	AMB	NEG	NEG	POS	POS	POS	NEG	POS	NEG	NEG	NEG	NEG	POS	POS	NEG	POS	NEG	NEG	POS
**CC188-MSSA**																																			
2012_206_Z3466 (968/12)	POS	AMB	AMB	POS	POS	AMB	POS	POS	POS	NEG	NEG	NEG	NEG	POS	NEG	NEG	POS	NEG	NEG	POS	NEG	POS	AMB	NEG	NEG	POS	NEG	NEG	POS	NEG	POS	NEG	POS	POS	POS
2012_251_Z3534 (3026/12)	POS	AMB	POS	AMB	POS	AMB	POS	POS	POS	NEG	NEG	NEG	NEG	POS	NEG	NEG	POS	NEG	NEG	POS	NEG	POS	NEG	NEG	NEG	AMB	NEG	NEG	POS	NEG	POS	NEG	POS	POS	NEG
2012_320_Z3607 (7144/12)	POS	AMB	AMB	POS	POS	AMB	POS	POS	POS	NEG	NEG	NEG	NEG	POS	NEG	NEG	POS	NEG	NEG	POS	NEG	POS	NEG	NEG	NEG	AMB	NEG	NEG	POS	NEG	POS	NEG	POS	POS	NEG
2012_321_Z3608 (7147/12)	AMB	POS	AMB	POS	POS	AMB	POS	POS	POS	NEG	NEG	NEG	NEG	POS	NEG	NEG	POS	NEG	NEG	POS	NEG	POS	AMB	NEG	NEG	AMB	NEG	NEG	POS	NEG	POS	NEG	POS	POS	NEG
2012_358_Z3418 (1202-6332)	POS	AMB	AMB	POS	POS	AMB	POS	POS	POS	NEG	NEG	NEG	NEG	POS	NEG	NEG	POS	NEG	NEG	POS	NEG	POS	AMB	NEG	NEG	POS	NEG	NEG	POS	NEG	POS	NEG	POS	POS	AMB
**CC398-MSSA**																																			
2013_PA11_Z3994	NEG	POS	NEG	POS	POS	POS	AMB	POS	POS	NEG	NEG	NEG	NEG	NEG	NEG	NEG	NEG	NEG	NEG	NEG	NEG	NEG	POS	NEG	NEG	NEG	NEG	NEG	POS	POS	NEG	POS	NEG	NEG	AMB
2013_PA57_Z4040	NEG	POS	NEG	POS	POS	POS	NEG	POS	POS	NEG	NEG	NEG	NEG	NEG	NEG	NEG	NEG	NEG	NEG	NEG	NEG	AMB	POS	NEG	NEG	NEG	NEG	NEG	POS	POS	NEG	POS	NEG	NEG	NEG
**ST1852-MSSA**																																			
2012_277_Z3564 (4050/12)	POS	AMB	POS	AMB	POS	AMB	POS	POS	POS	NEG	NEG	NEG	POS	NEG	NEG	NEG	AMB	NEG	NEG	POS	NEG	POS	NEG	NEG	NEG	POS	POS	NEG	POS	NEG	AMB	NEG	POS	POS	POS
**CC1223/1594-MSSA**																																			
2012_337_Z3624 (8070B/12)	AMB	POS	POS	AMB	NEG	NEG	NEG	NEG	POS	NEG	NEG	NEG	NEG	NEG	NEG	NEG	POS	NEG	NEG	NEG	NEG	POS	NEG	POS	NEG	NEG	POS	NEG	POS	NEG	POS	NEG	POS	POS	NEG
**MSSA related to ST2250/2277**																																			
2012_203_Z3463 (829/12)	POS	AMB	POS	AMB	NEG	NEG	POS	NEG	POS	NEG	NEG	NEG	NEG	NEG	POS	NEG	NEG	NEG	NEG	NEG	NEG	AMB	NEG	NEG	NEG	AMB	NEG	POS	POS	NEG	NEG	POS	POS	POS	NEG
2012_269_Z3556 (3769A/12)	AMB	POS	POS	AMB	NEG	NEG	NEG	NEG	POS	NEG	NEG	NEG	NEG	NEG	POS	NEG	NEG	NEG	NEG	NEG	NEG	NEG	NEG	NEG	NEG	NEG	NEG	POS	POS	NEG	NEG	NEG	POS	POS	NEG
2012_292_Z3579	AMB	POS	POS	AMB	NEG	NEG	NEG	NEG	POS	NEG	NEG	NEG	NEG	NEG	POS	NEG	NEG	NEG	NEG	NEG	NEG	NEG	NEG	NEG	NEG	NEG	NEG	POS	POS	NEG	NEG	NEG	POS	POS	NEG
2012_299_Z3586 (5487/12)	AMB	POS	POS	AMB	NEG	NEG	NEG	NEG	POS	NEG	NEG	NEG	NEG	NEG	POS	NEG	NEG	NEG	NEG	NEG	NEG	NEG	NEG	NEG	NEG	NEG	NEG	POS	POS	NEG	NEG	NEG	POS	POS	NEG
2012_301_Z3588 (5717/12)	AMB	POS	POS	AMB	NEG	NEG	NEG	NEG	POS	NEG	NEG	NEG	NEG	NEG	POS	NEG	NEG	NEG	NEG	NEG	NEG	NEG	NEG	NEG	NEG	NEG	NEG	POS	POS	NEG	NEG	NEG	POS	POS	NEG
**ST2596dlv-MSSA**																																			
2012_310_Z3597 (6034/12)	AMB	POS	AMB	POS	NEG	NEG	NEG	NEG	POS	NEG	NEG	POS	NEG	NEG	NEG	NEG	NEG	NEG	NEG	NEG	NEG	NEG	NEG	NEG	NEG	NEG	NEG	POS	POS	POS	POS	POS	POS	POS	NEG
